# The neddylation of the RNA-dependent RNA polymerase 3D of Coxsackievirus B3 promotes viral replication

**DOI:** 10.1128/jvi.01535-25

**Published:** 2025-10-31

**Authors:** Siwei Li, Yanyan Dong, Xuexuan Wang, Danxiang Feng, Tian Luan, Ziyuan Wang, Lexun Lin, Yang Chen, Yao Wang, Yanru Fei, Yan Wang, Zhaohua Zhong, Wenran Zhao

**Affiliations:** 1 Department of Cell Biology, The School of Basic Medical Sciences, Harbin Medical University 34707, Harbin, China; 2 Teaching Center of Pathogenic Biology, The School of Basic Medical Sciences, Harbin Medical University 34707, Harbin, China; 3 Department of Microbiology, The School of Basic Medical Sciences, Harbin Medical University 34707, Harbin, China; Loyola University Chicago - Health Sciences Campus, Maywood, Illinois, USA

**Keywords:** Coxsackievirus B, RNA-dependent RNA polymerase 3D, posttranslational modification, NEDD8, tripartite motif-containing 4

## Abstract

The pathogenesis of Coxsackievirus B3 (CVB3), the causative pathogen for severe diseases such as myocarditis, pancreatitis, and meningitis, remains largely unknown. Neddylation, the covalent modification of proteins through the conjugation of NEDD8, a ubiquitin-like molecule, plays critical roles in regulating cellular activities. Our previous study demonstrated that 3D^pol^, the RNA-dependent RNA polymerase of CVB3, was modified by ubiquitin. Since NEDD8 is highly homologous to ubiquitin in both the sequence and structure, we hypothesized that 3D^pol^ may also undergo neddylation. Here, we demonstrated that 3D^pol^ of CVB3 is modified by NEDD8. Proteomics and immunoprecipitation studies identified that NEDD8 expression was upregulated in CVB3-infected cells, and 3D^pol^ was neddylated. Through the overexpression or knockdown of the subunit of NEDD8-activating enzyme or NEDD8, we show that neddylation promoted CVB3 replication. Furthermore, the overexpression of NEDD8-specific protease 1 completely blocked 3D^pol^ neddylation and suppressed viral replication. The neddylation sites of 3D^pol^, K261, and K457 were identified by mutagenesis studies. We further showed that neddylation enhanced 3D^pol^ stability. In contrast, when the neddylation sites were mutated (K261R, K457R, or K261/457R), the 3D^pol^ degradation rate was significantly accelerated. Moreover, the growth kinetics of the recombinant viruses containing the mutated 3D^pol^ at the neddylation sites was markedly decreased. Through analyzing the 3D^pol^ interacting proteins with mass spectrometry, we identified that TRIM4 is the E3 ligase, which upregulates 3D^pol^ neddylation. TRIM4 knockdown markedly suppressed viral replication and almost completely blocked 3D^pol^ neddylation. In contrast, TRIM4 overexpression promoted CVB3 replication. Taken together, this study demonstrated that 3D^pol^ neddylation facilitates CVB3 replication.

**IMPORTANCE:**

CVB3 infection is commonly related to the inflammatory disease of the heart, which may develop to dilated cardiomyopathy and heart failure. Neddylation is a process in which the ubiquitin-like molecule NEDD8 is covalently linked to the specific lysine residues of the target proteins. Increasing evidence has shown that the neddylation of either host or viral proteins may alter viral replication. In this study, we demonstrated that 3D^pol^, the RNA-dependent RNA polymerase of CVB3, is modified by NEDD8 at its lysine residues 261 and 457, and the neddylation process is mediated by the E3 ligase TRIM4. Neddylation enhances the stability of 3D^pol^ and facilitates viral replication, while viruses with mutated 3D^pol^, which cannot be neddylated, showed decreased replication capacity. Our findings not only add novel insights for understanding the pathogenesis of CVB3 but also identify that targeting neddylation could be a potential antiviral strategy for the treatment of CVB3 infection.

## INTRODUCTION

Small RNA viruses are widespread in nature, and some of them, such as enterovirus A71 (EV-A71), Coxsackievirus group A (CVA), or Coxsackievirus group B (CVB), can cause severe illnesses. CVBs are single-stranded, positive-sense, non-enveloped RNA viruses, which belong to the *Enterovirus* genus of *Picornaviridae* family (1). There are six known serotypes of CVB (CVB1 ~6), which can cause a variety of diseases ranging from the common cold to viral myocarditis, pancreatitis, and meningitis (2, 3). CVB1, CVB3, and CVB5 are cardiophilic in nature (4), while CVB3 has been shown as the primary causative pathogen of myocarditis, dilated cardiomyopathy, and even heart failure (5–7).

CVB3, with a genome of 7.4 kb (8), consists of a single open reading frame (ORF) and two untranslated regions (UTRs) located at the ends of the viral genome, the 5’UTR and 3’UTR (1, 9). The ORF is translated into a single polyprotein, which is cleaved by viral proteases 2A^pro^, 3C^pro^, and 3CD^pro^ into four structural proteins (VP1–4) and seven nonstructural proteins (2A^pro^, 2B, 2C, 3A, 3B, 3C^pro^, and 3D^pol^) (1, 10). 3D^pol^ is the RNA-dependent RNA polymerase (RdRp), which mediates viral genomic replication (11, 12). To replicate viral RNA, CVB3 uses its genomic RNA, a positive-sense, single-stranded RNA (+ssRNA), as a template to synthesize a complementary negative-stranded RNA (-ssRNA), which is then used as the template to produce the genomic RNA for progeny viruses (13).

The posttranslational modifications (PTMs) of proteins, such as phosphorylation, glycosylation, methylation, and ubiquitination, play critical roles in regulating the stability, enzymatic activity, and subcellular localization of the substrate proteins (14, 15). PTMs are also exploited by viruses to facilitate viral replication or to inhibit cellular defense (15). Neddylation is a process of PTMs, in which substrate proteins are covalently modified by neural precursor cell-expressed developmentally downregulated protein 8 (NEDD8), a highly conserved ubiquitin-like protein with 59% amino acid identity to ubiquitin (Ub) (16, 17). Similar to ubiquitination, neddylation also requires an enzymatic cascade containing NEDD8-activating enzyme (NAE), NEDD8-conjugating enzyme E2, and substrate-specific NEDD8 E3 ligases (18, 19). There is only one NAE (NAE1/UBA3 heterodimer) (20) and two NEDD8-conjugating enzymes (UBC12/UBE2M or UBE2F) (21, 22). Mature NEDD8 forms a thioester bond with the cysteine active site in UBA3 in an ATP-dependent manner catalyzed by NAE1 (20, 22). Subsequently, NEDD8 is transferred to the cysteine active site of the NEDD8-conjugating enzyme (21, 22). Finally, in the presence of E3 ligases, the glycine at the C-terminal of NEDD8 forms an isopeptide bond with the specific lysine (K) residue on the substrate protein (19, 23). Unlike ubiquitination, there are a limited number of NEDD8 E3 ligases identified, and all of them are also E3 Ub ligases (24). In addition, like most of the cellular biochemical reactions, neddylation is a reversible process, which is mediated by deneddylases, such as COP9 signalosome complex and NEDD8-specific protease 1 (NEDP1). Through deneddylation, NEDD8 is removed from the target protein, allowing NEDD8 to participate in the next cycle of neddylation (23).

Previous studies showed that neddylation is involved in the life cycle of some viruses including hepatitis B virus (HBV), influenza A virus (IAV), and EV-A71 (25–28). However, it is unknown whether or not neddylation also participates in CVB infection. Our previous study demonstrated that the 3D^pol^ of CVB3 contains the ubiquitination site at its K220, which upregulates the degradation of 3D^pol^ (29). Since NEDD8 shows high homology to Ub in both the sequence and structure, we hypothesized that 3D^pol^ of CVB3 might also be modified by neddylation.

In this study, we demonstrated that 3D^pol^ of CVB3 is neddylated at K261 and K457. The neddylation of 3D^pol^ is mediated by the E3 ligase tripartite motif 4 (TRIM4). We further show that neddylation of 3D^pol^ increases its stability, which in turn promotes viral replication. Our study adds novel insights for understanding the pathogenesis of CVB3 infection.

## RESULTS

### Viral protein 3D^pol^ is neddylated

**Fig 1:**
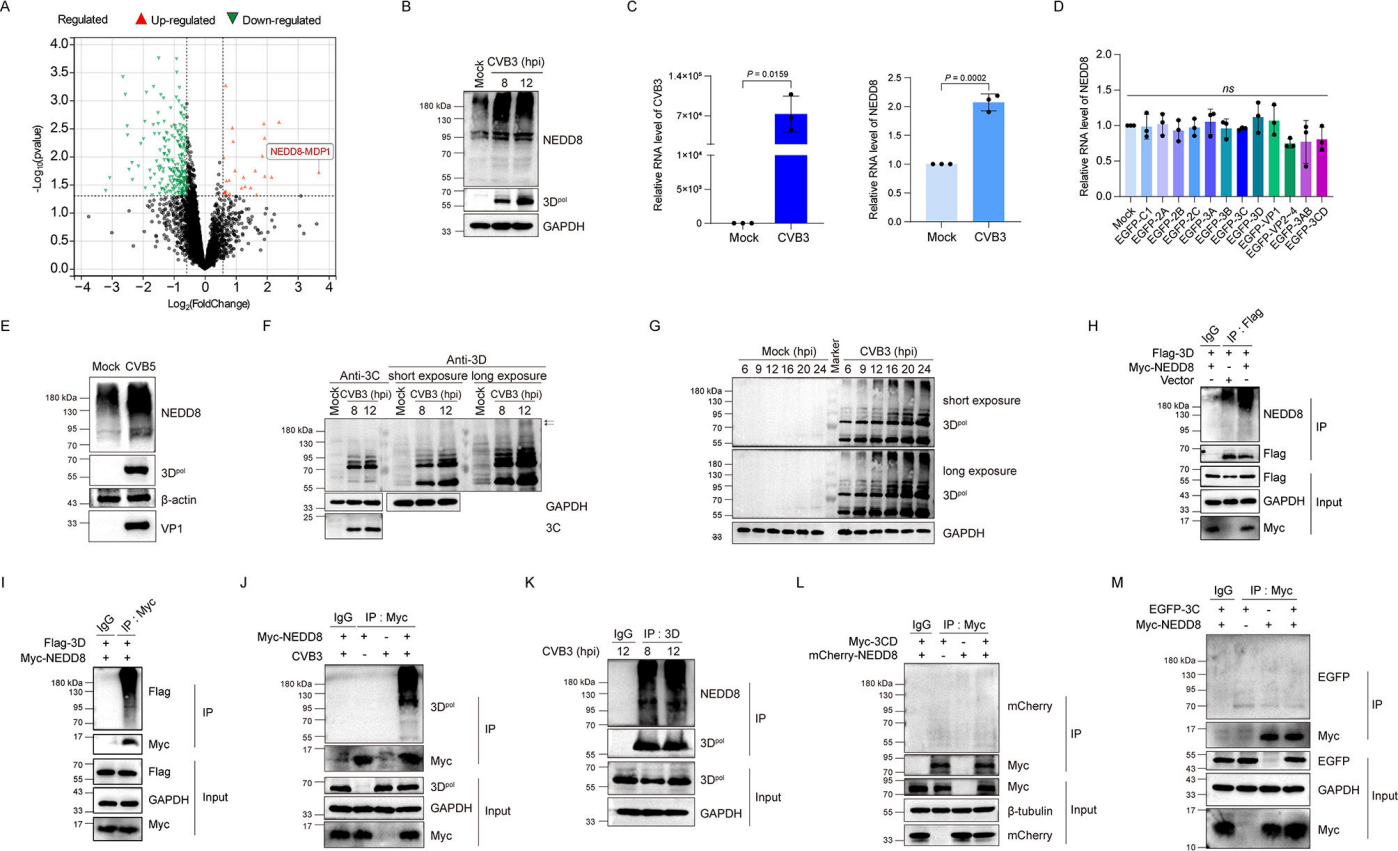
CVB3 3D^pol^ is neddylated. (**A**) HeLa cells were mock-infected or infected with CVB3 (MOI = 1) for 24 h. Total cellular proteins were extracted and analyzed by proteomics. The differentially expressed genes were displayed by a volcanic diagram. (**B**) HeLa cells were infected with CVB3 (MOI = 10) for 8 or 12 h. Cells were collected and subjected to immunoblotting with anti-NEDD8 antibody to analyze the cellular neddylation status. (**C**) HeLa cells were infected with CVB3 (MOI = 10), and total cellular RNA was extracted at 8 h post-infection. The mRNA level of CVB3 and NEDD8 was detected by RT-qPCR. (**D**) HEK293T cells were transfected with the constructs expressing different viral proteins for 24 h. Total RNA was extracted and analyzed by RT-qPCR to determine the mRNA abundance of NEDD8. (**E**) HeLa cells were infected with CVB5 (MOI = 5) for 12 h. Cells were collected and subjected to immunoblotting with anti-NEDD8 antibody to analyze the cellular neddylation status. (**F**) HeLa cells were infected with CVB3 (MOI = 10) for 8 or 12 h, and viral protein 3D^pol^ and 3C were determined by immunoblotting with anti-3D or anti-3C antibody. (**G**) HeLa cells were mock-infected or infected with CVB3 (MOI = 1) and collected at the indicated time points. Viral protein 3D^pol^ was determined by immunoblotting with the anti-3D antibody. (**H, I**) HEK293T cells were transfected with the indicated plasmids for 48 h. Total cellular proteins were extracted and subjected to denatured Co-IP with anti-Flag antibody (**H**), anti-Myc antibody (**I**), or mouse IgG. The precipitated proteins were separated by SDS-PAGE and probed with the indicated specific antibodies. (**J**) HEK293T cells were transfected with the construct expressing Myc-NEDD8 for 36 h and then infected with CVB3 (MOI = 10) for 8 h. The cell lysates were subjected to denatured Co-IP with anti-Myc antibody or mouse IgG. The precipitated proteins were separated by SDS-PAGE and probed with the indicated specific antibodies. (**K**) HeLa cells were infected with CVB3 (MOI = 10) for 8 or 12 h. The cell lysates were subjected to denatured Co-IP with the anti-3D antibody or mouse IgG. The precipitated proteins were separated by SDS-PAGE and probed with the indicated specific antibodies. (**L, M**) HEK293T cells were transfected with the indicated plasmids for 48 h. Total cellular proteins were extracted and subjected to denatured Co-IP with anti-Myc antibody or mouse IgG. Neddylated 3CD was determined by immunoblotting with anti-mCherry antibody (**L**), and the neddylated 3C was determined by immunoblotting with the anti-EGFP antibody (**M**). Three independent experiments were performed, and representative results were presented. hpi: hours post-infection.

To study how CVB3 infection would impair host protein synthesis, we analyzed the protein expression profile of CVB3-infected cells by mass spectrometry (MS). We found that NEDD8 (Gene name: NEDD8-MDP1; Gene ID: 100528064) was one of the proteins that was significantly upregulated in CVB3-infected cells (Fig. 1A; Table S1). To evaluate the correlation between CVB3 infection and neddylation, the overall neddylated proteins and NEDD8 expression were determined (Fig. 1B and C). We show that CVB3 infection enhanced neddylation and NEDD8 expression (Fig. 1B and C). To show whether individual viral protein exerts the capability to impact neddylation, we determined the expression of NEDD8 in the cells expressing viral mature proteins or viral precursors (Fig. 1D). We show that none of the viral proteins or viral precursor proteins was able to alter NEDD8 expression, suggesting that the upregulated NEDD8 and neddylation is the consequence of viral replication. We further wondered whether upregulation of neddylation is shared by other types of CVB or not. To this end, we determined the neddylation status of the cells infected with CVB5. We show that neddylation was significantly enhanced by CVB5 infection (Fig. 1E), further implicating the importance of the upregulated neddylation in CVB infection.

Our previous study demonstrated that 3D^pol^ of CVB3 is ubiquitinated (29). Since Ub and NEDD8 are highly homologous, we hypothesized that viral 3D^pol^ might be neddylated. To validate this hypothesis, we first observed if there were 3D^pol^ molecules with high molecular weight (MW) when cell lysates were analyzed by immunoblotting. 3D^pol^ of CVB3 is usually migrating at around 58 kDa in the SDS-PAGE gel. With the covalent modification of NEDD8 or Ub, the MW of 3D^pol^ would become much higher than 58 kDa. As shown in Fig. 1F, 3D^pol^ with an MW higher than 180 kDa appeared at 12 h of post-infection (indicated by arrows), while there was no viral 3C^pro^ with an MW higher than 180 kDa. Proteins migrating between >70 and ~110 kDa showed similar patterns revealed by either anti-3C and anti-3D (Fig. 1F), indicating that these proteins contain both 3C^pro^ and 3D^pol^, which are likely viral precursors derived from P3 (3CD, 3BCD, and 3ABCD). Since 3C^pro^-containing protein molecules did not appear at high MW (Fig. 1F), we proposed that it is 3D^pol^ rather than 3C^pro^ or the precursor of 3D^pol,^ which is under PTM. To further reveal that 3D^pol^ contains modification during virus infection, cells were infected with CVB3, and 3D^pol^ was analyzed at different time points of post-infection. We found that 3D^pol^ with high MW was accumulating as viral infection continues (Fig. 1G).

To demonstrate whether or not 3D^pol^ is modified by NEDD8, denatured Co-IP was carried out for the cells overexpressing 3D^pol^ (Fig. 1H and I) or infected with CVB3 (Fig. 1J and K). We show that 3D^pol^ of CVB3, when ectopically expressed, was neddylated (Fig. 1H and I). To avoid the artificial effects of NEDD8 overexpression, the neddylation of 3D^pol^ was determined in the natural process of virus infection (Fig. 1K). We demonstrated that 3D^pol^ was neddylated in CVB3-infected cells (Fig. 1J and K). Furthermore, we confirmed that neither 3 CD nor 3C^pro^ was neddylated (Fig. 1L and M). Collectively, these results demonstrate that 3D^pol^ of CVB3 is neddylated.

### Neddylation facilitates CVB3 replication

**Fig 2:**
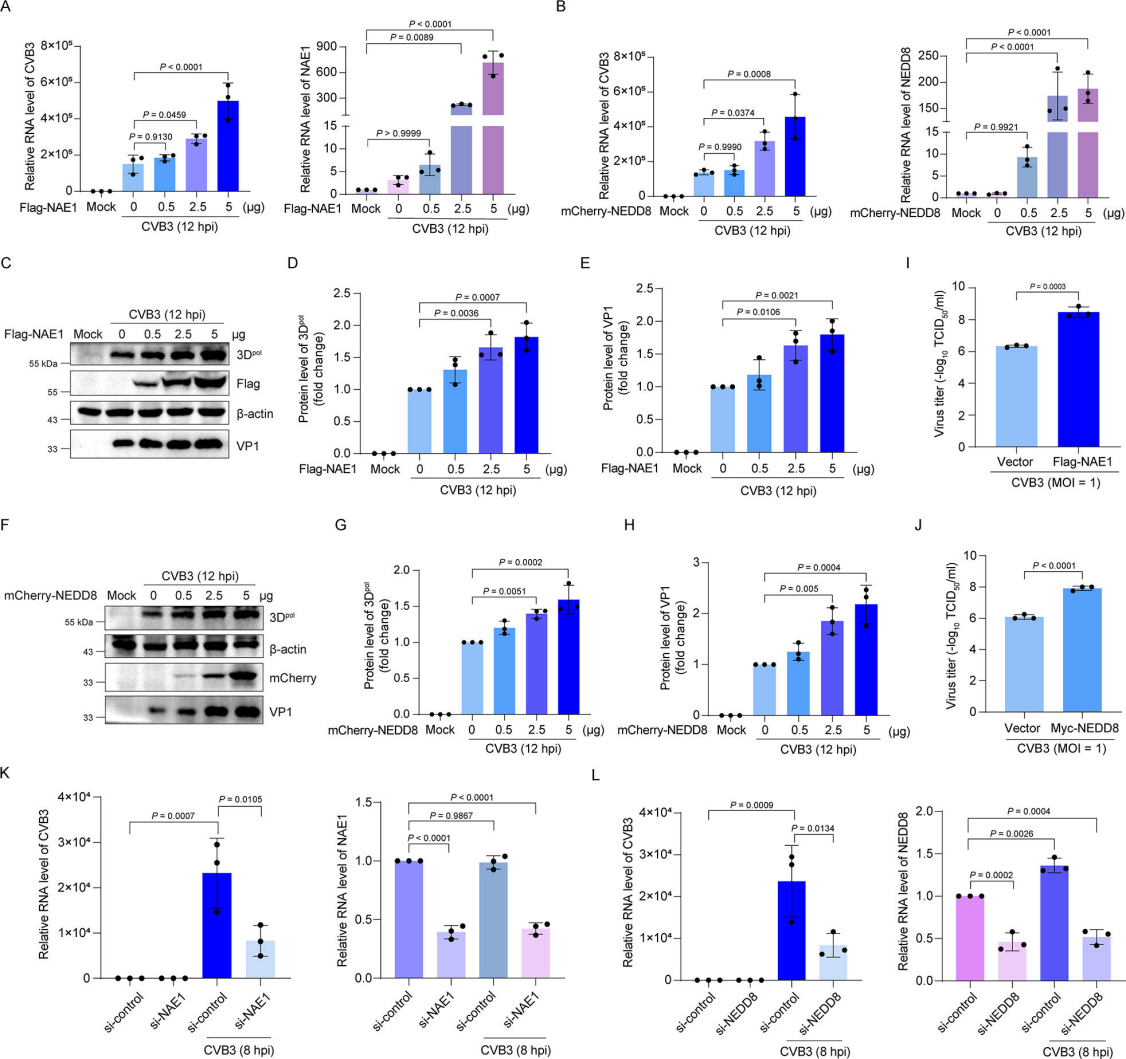
Neddylation of 3D^pol^ promotes CVB3 replication. (**A–H**) HEK293T cells were transfected with the construct expressing Flag-NAE1 (**A, C–E**) or mCherry-NEDD8 (**B, F–H**) at the indicated concentrations for 36 h, and then cells were infected with CVB3 (MOI = 1) for 12 h. The cell lysates were harvested and subjected to RT-qPCR (**A, B**) and immunoblotting (**C–H**), respectively. (**I, J**) HEK293T cells were transfected with the construct expressing Flag-NAE1 (**I**) or Myc-NEDD8 (**J**) for 36 h and then infected with CVB3 (MOI = 1) for 12 h. Cells were collected and subjected to freeze-thaw three times to release viral particles, which were determined by TCID_50_ assay. (**K, L**) HEK293T cells were transfected with control siRNA, NAE1 siRNA (**K**), or NEDD8 siRNA (**L**) for 24 h and then infected with CVB3 (MOI = 10) for 8 h. The cells were harvested for RT-qPCR. Three independent experiments were performed, and representative results were presented. Quantitative analysis of the immunoblotting results was carried out by ImageJ. hpi: hours post-infection.

With the identification that 3D^pol^ is neddylated, we further determined how neddylation would impact viral replication. To this end, CVB3 replication was observed in the cells overexpressing NAE1, the subunit of the NAE, or NEDD8 (Fig. 2A through J). We show that overexpression of NAE1 or NEDD8 obviously led to the increased levels of viral RNA in a dose-dependent manner (Fig. 2A and B). Similarly, levels of viral proteins 3D^pol^ and VP1 were also increased in the cells overexpressing NAE1 or NEDD8 (Fig. 2C through H). Moreover, NAE1 or NEDD8 overexpression significantly increased the virus yield (Fig. 2I and J). In contrast, knockdown of NAE1 or NEDD8 resulted in markedly decreased viral RNA levels (Fig. 2K and L). Collectively, these data demonstrate that neddylation facilitates CVB3 replication.

### Deneddylation inhibits CVB3 replication

**Fig 3:**
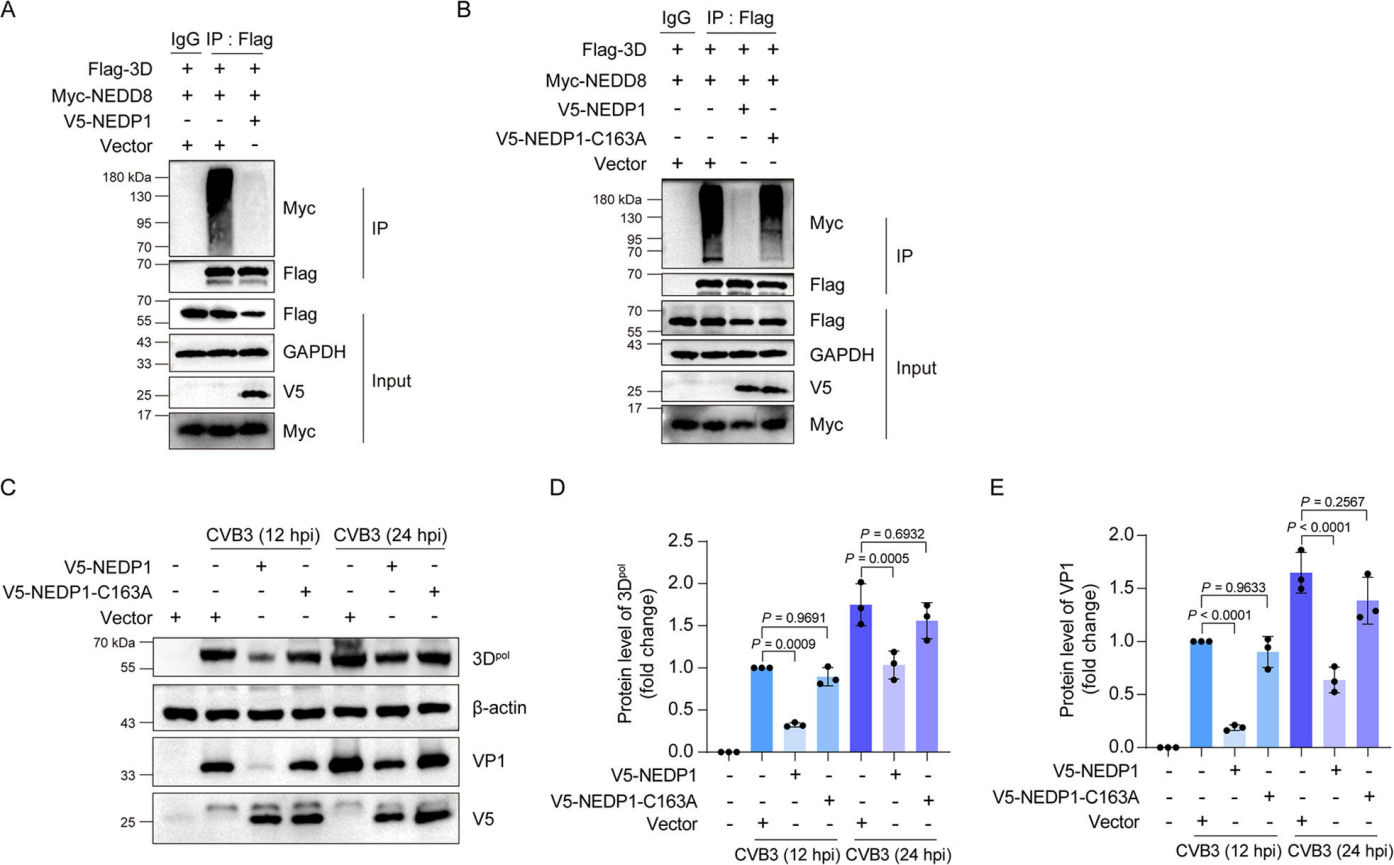
Deneddylation inhibits CVB3 replication. (**A, B**) HEK293T cells were transfected with the indicated plasmids for 48 h. Total cellular proteins were extracted and subjected to denatured Co-IP with anti-Flag antibody or mouse IgG. The precipitated proteins were separated by SDS-PAGE and probed with the indicated specific antibodies. (**C–E**) HEK293T cells were transfected with the indicated plasmids for 36 h, and then the cells were infected with CVB3 (MOI = 1) for 12 or 24 h. The total cellular proteins were extracted and detected by immunoblotting. Three independent experiments were performed, and representative results were presented. Quantitative analysis of the immunoblotting results was carried out by ImageJ. hpi: hours post-infection.

Neddylation is reversed by deneddylation, the process in which NEDD8 is removed from the neddylated substrate protein. NEDP1, a NEDD8-specific protease, mediates the removal of NEDD8 from a wide variety of substrates (30). To further verify that neddylation is utilized to enhance CVB3 replication, we determined how deneddylation would impact CVB3 replication. To this end, the neddylation of 3D^pol^ of CVB3 was determined in the cells with or without NEDP1 overexpression. As shown in Fig. 3, overexpression of NEDP1 (Fig. 3A), but not the mutated NEDP1 (without enzymatic activity) (Fig. 3B), completely blocked the neddylation of 3D^pol^. Moreover, the overexpression of NEDP1, but not the mutated NEDP1, resulted in a significant decrease in viral protein 3D^pol^ and VP1 (Fig. 3C through E). These results further demonstrate that 3D^pol^ of CVB3 is neddylated, and neddylation enhances viral replication.

### The 3D^pol^ of CVB3 is neddylated at K261 and K457

**Fig 4:**
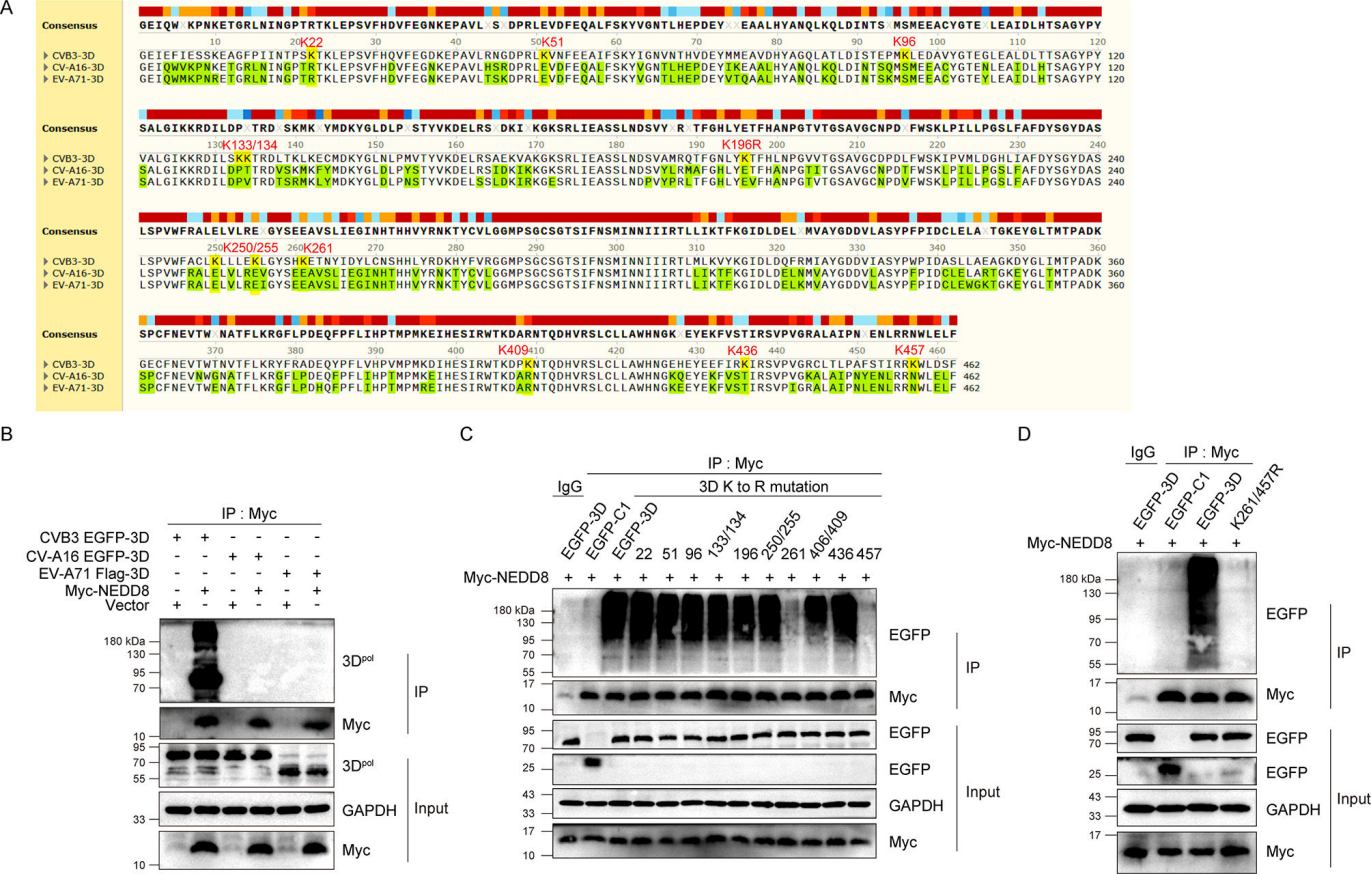
3D^pol^ of CVB3 was neddylated at K261 and K457. (**A**) The amino acid sequence alignment of 3D^pol^ of CVB3, CV-A16, and EV-A71. The lysine (**K**) residues in the CVB3 3D^pol^ that are absent in the other two viruses are highlighted as yellow. (**B**) HEK293T cells were transfected with the indicated plasmids for 48 h. Total cellular proteins were extracted and subjected to denatured Co-IP with the anti-Myc antibody. The precipitated proteins were separated by SDS-PAGE and probed with the indicated specific antibodies (*n* = 3). (**C, D**) HEK293T cells were transfected with the indicated plasmids for 48 h. Total cellular proteins were extracted and subjected to denatured Co-IP with the anti-Myc antibody or mouse IgG. The precipitated proteins were separated by SDS-PAGE and probed with the indicated specific antibodies (*n* = 3).

With the finding that neddylation of 3D^pol^ facilitates CVB3 replication, we wondered which specific lysine residue in 3D^pol^ is neddylated. Before answering this question, we asked if other species of enteroviruses also contain 3D^pol^ neddylation since the amino acid sequences of 3D^pol^ among enteroviruses share more than 50% homology. To this end, we compared the 3D^pol^ amino acid sequence of CVB3 (GenBank: U57056.1), CV-A16 (GenBank: U05876.1), and EV-A71 (Genbank: U22521.1) (Fig. 4A), and the neddylation status of the 3D^pol^ from these viruses was determined with Co-IP analysis (Fig. 4B). We show that neddylation was identified only in the 3D^pol^ of CVB3, implicating that the neddylation sites of 3D^pol^ of CVB3 are not shared by the 3D^pol^ of either CV-A16 or EV-A71.

According to the amino acid sequence of 3D^pol^ (Fig. 4A), we identified 12 K residues (K22, 51, 96, 133, 134, 196, 250, 255, 261, 409, 436, and 457), which exclusively occur in 3D^pol^ of CVB3. To determine the neddylation site of 3D^pol^, constructs expressing 3D^pol^ mutations were generated, in which K was mutated to arginine (R). The neddylation of the mutated 3D^pol^ was determined by Co-IP (Fig. 4C). We show that when 3D^pol^ was mutated at K261 or K457, 3D^pol^ neddylation was significantly inhibited. When both K261 and K457 were mutated, 3D^pol^ neddylation was completely blocked (Fig. 4D). These data demonstrate that NEDD8 modification of 3D^pol^ occurs at K261 and K457.

### Neddylation enhances 3D^pol^ stability

**Fig 5:**
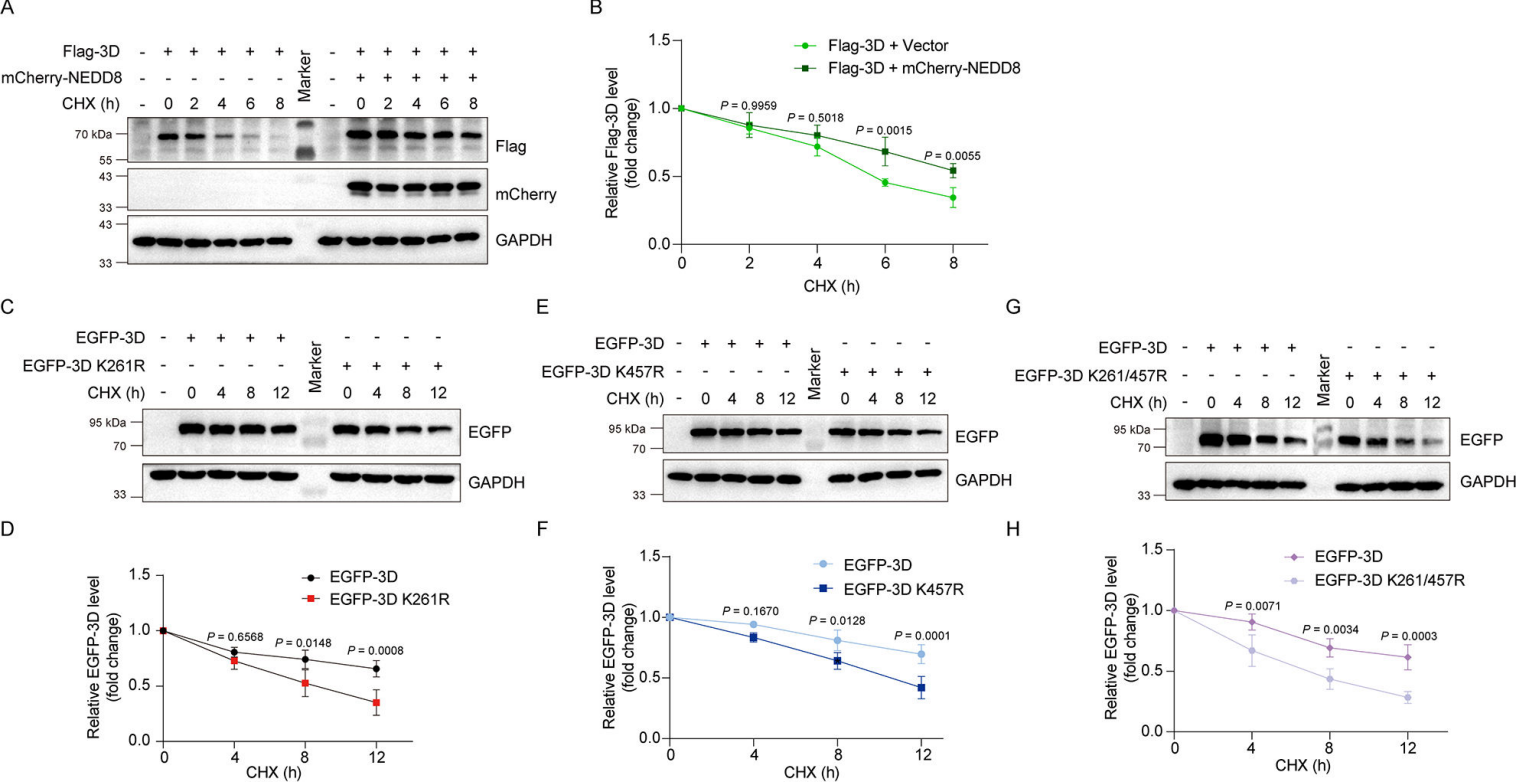
Neddylation enhances 3D^pol^ stability. (**A, B**) HEK293T cells were transfected with the construct expressing Flag-3D together with or without mCherry-NEDD8 for 36 h and then treated with CHX (100 nM) for the indicated time points. Cells were harvested and subjected to immunoblotting. (**C–H**) HEK293T cells were transfected with the construct expressing EGFP-3D or EGFP-3D-K261R, -K457R, and -K261/457R for 36 h. The cells were treated with CHX (100 nM) for the indicated time points. The cell lysates were collected and subjected to immunoblotting. Three independent experiments were performed, and representative results were presented. Quantitative analysis of the immunoblotting results was carried out by ImageJ. CHX: cycloheximide.

Our previous study has shown that the ubiquitination of 3D^pol^ of CVB3 promotes its degradation through proteasomal activity (29). However, CVB3 replication is not disrupted in spite of the proteasomal degradation of 3D^pol^, suggesting that a counteractive mechanism might be involved to maintain the stability of viral proteins. Since the conjugation of NEDD8 often induces a conformational change of its targets, and hence their biochemical properties (31), we raised the question of how neddylation would impact the stability of 3D^pol^. To this end, cycloheximide (CHX) chase assay was used to measure the degradation rate of 3D^pol^ or the mutant 3D^pol^ at K261, K457, and K261/457. As shown in Fig. 5, 3D^pol^ degradation was significantly inhibited in the cells overexpressing NEDD8 (Fig. 5A and B), indicating neddylation enhances 3D^pol^ stability. In contrast, 3D^pol^ with mutation at either K261 or K457 or both (K261/457) showed significantly accelerated degradation (Fig. 5C through H), demonstrating that neddylation promotes 3D^pol^ stability.

### Viral replication is impaired by the mutations at the neddylation sites of 3D^pol^ of CVB3

**Fig 6:**
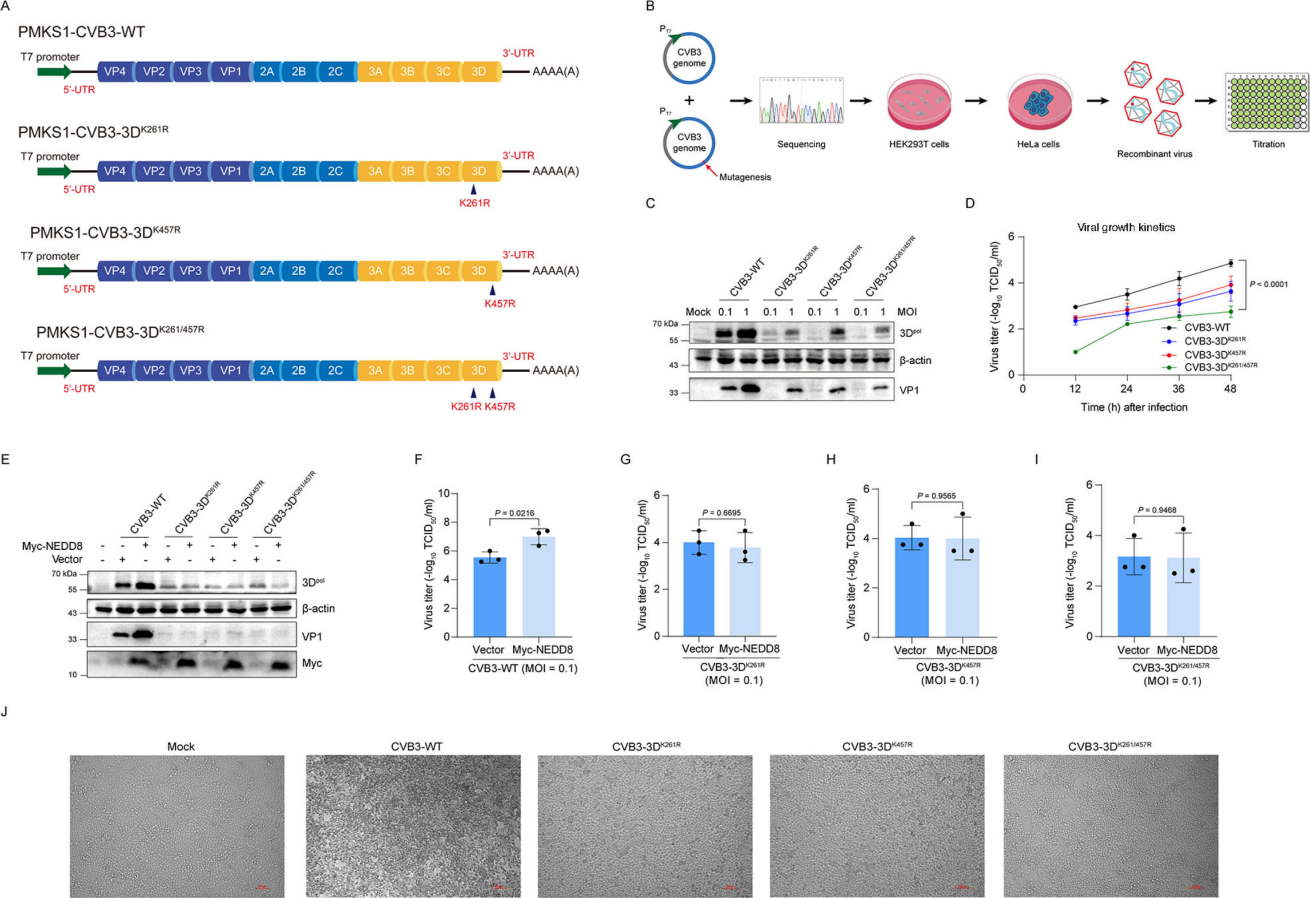
Abolishment of 3D^pol^ neddylation reduces CVB3 replication. (**A**) Schematic diagram shows the construction of the T7 promoter-driven infectious clone of CVB3. The indicated mutations were separately introduced into pMKS1-CVB3-WT, the construct containing the whole length of CVB3 genomic cDNA. (**B**) Schematic diagram shows rescue of wild-type and mutant CVB3 viruses. (**C**) HeLa cells were infected with wild-type or mutant viruses at different MOI (0.1 and 1) for 24 h. The total cellular proteins were extracted and analyzed by immunoblotting (*n* = 3). (**D**) HeLa cells were infected with wild-type or mutant CVB3 (MOI = 0.1). The cells were subjected to three freeze-thaw cycles at different time points post-infection, and the virus titers were calculated by TCID_50_ (*n* = 3). (**E–I**) HEK293T cells were transfected with the construct expressing Myc-NEDD8 or empty vector for 36 h and then infected with CVB3-WT, CVB3-3D^K261R^, CVB3-3D^K457R^, and CVB3-3D^K261/457R^, respectively (MOI = 0.1). The cells were harvested at 24 h post-infection for immunoblotting (E; *n*= 3) and titration of virus yield (F–I; *n* = 3). (**J**) HeLa cells were infected with CVB3-WT, CVB3-3D^K261R^, CVB3-3D^K457R^, and CVB3-3D^K261/457R^, respectively (MOI = 0.1) for 48 h. Cytopathic effect induced by viral infection was viewed with microscopy. Representative images were provided. Bar = 100 µm. WT: wild-type.

To further reveal that neddylation of 3D^pol^ is used by CVB3 as a strategy to facilitate viral replication, we generated recombinant CVB3 viruses that contain mutated 3D^pol^ at its neddylation sites. We compared the viral growth kinetics and viral replication between wild-type CVB3 and the mutated CVB3. As shown in Fig. 6, recombinant viruses were generated based on pMKS1, which contains the cDNA of the entire CVB3 genome (Fig. 6A). The constructs of the recombinant CVB3 were sequenced. Viruses were recovered by transfecting HEK293T cells with these constructs. The virus titer was determined with TCID_50_ assay using HeLa cells (Fig. 6B). HeLa cells were infected with wild-type or mutant CVB3 at various MOI (0.1 and 1) for 24 h, and viral protein 3D^pol^ and VP1 were determined (Fig. 6C). Viral growth kinetics was determined by infecting cells with 0.1 MOI of wild-type or mutant CVB3. The cell culture was harvested at various time points of post-infection, and virus titers were determined with TCID_50_ assay (Fig. 6D). We show that the replication of mutated CVB3 was impaired, represented by the significantly decreased levels of 3D^pol^, VP1, and viral growth kinetics (Fig. 6C and D).

Furthermore, overexpression of NEDD8 significantly increased the levels of 3D^pol^ and VP1 in the cells infected with wild-type CVB3 (Fig. 6E), while NEDD8 overexpression did not alter both 3D^pol^ and VP1 levels in the cells infected with mutated viruses (Fig. 6E), demonstrating that 3D^pol^ neddylation promotes viral replication. Similarly, NEDD8 overexpression significantly increased the production of viral particles of wild-type CVB3 (Fig. 6F), while the viral particle production of the recombinant viruses was not significantly altered (Fig. 6G through I).

It should be noted that for the cells without NEDD8 expression, the virus yield of wild-type CVB3 was up to 10^6^ (Fig. 6F), while the yield of mutant CVB3 declined to 10^4^ (Fig. 6G and H), demonstrating that both neddylation sites in 3D^pol^ are indispensable for the efficient replication of CVB3. When cells were infected with the recombinant CVB3, which lost the two neddylation sites of 3D^pol^, the virus yield further declined (Fig. 6I). Collectively, these data confirmed that the neddylation of 3D^pol^ promotes CVB3 replication.

To further show the replication difference between the wild-type CVB3 and the mutated CVB3, the cytopathic effect (CPE) of the viruses was visualized (Fig. 6J). We observed that the CPE of the wild-type CVB3 was obvious, while CVB3 with 3D^pol^ mutation at K261 or K457 or K261/K457 showed significantly reduced CPE. Collectively, these data further demonstrated that 3D^pol^ neddylation facilitates the replication of CVB3.

### E3 ligase TRIM4 upregulates 3D^pol^ neddylation

**Fig 7:**
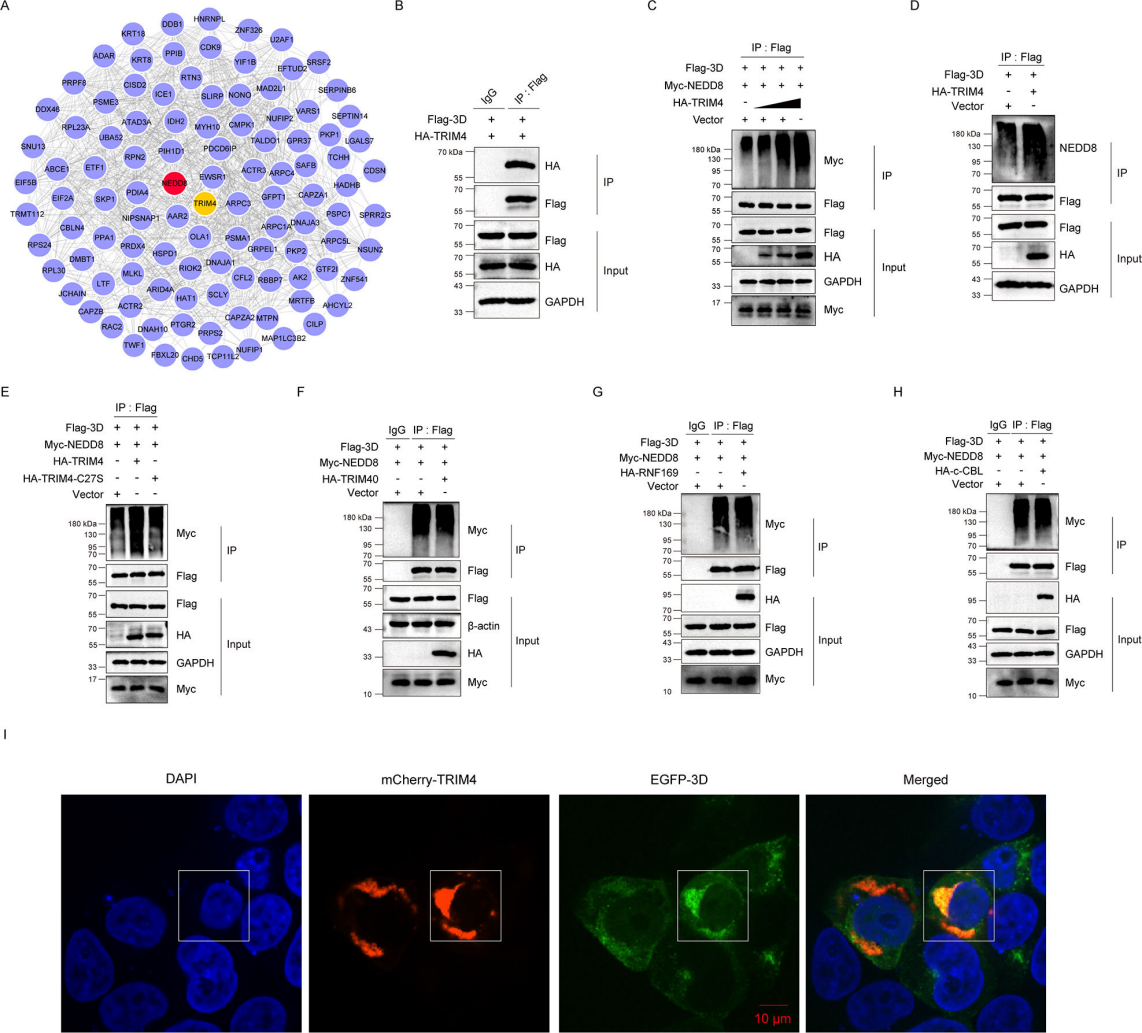
E3 ligase TRIM4 upregulates 3D^pol^ neddylation. (**A**) HEK293T cells were transfected with the construct expressing Flag-3D for 36 h, followed by CVB3 infection (MOI = 5) or mock-infection for 18 h. Total cellular proteins were extracted and subjected to denatured Co-IP with anti-Flag antibody. The proteins precipitated with 3D^pol^ were analyzed by mass spectrometry (MS). The proteins that were identified by MS were displayed by a network diagram (drawn with Cytoscape 3.7.0). (**B–H**) HEK293T cells were transfected with the indicated plasmids for 48 h. Total cellular proteins were extracted and subjected to denatured Co-IP with anti-Flag antibody or mouse IgG. The precipitated proteins were separated by SDS-PAGE and probed with the indicated specific antibodies (*n* = 3). (**I**) HEK293T cells were cultured to 50% confluency and were co-transfected with pEGFP-3D and pmCherry-TRIM4 for 36 h. Cells were vitalized with confocal microscopy. Nuclei were stained with DAPI. Bar = 10 µm.

To further understand how neddylation is exploited by CVB3 to promote 3D^pol^ stability, we carried out Co-IP to obtain the proteins that are interacting with 3D^pol^, and these 3D^pol^-interacting proteins were analyzed by MS. To this end, cells were transfected with pFlag-3D for 36 h and then infected with CVB3 for 18 h, followed by Co-IP assay to obtain cellular proteins that are co-precipitated with 3D^pol^. These proteins were analyzed by MS. Among the 3D^pol^-interacting proteins identified by MS (Fig. 7A; Table S2), we paid special attention to the Ub E3 ligases since all the known NEDD8 E3 ligases are Ub E3 ligases. To validate the role of the E3 ligases identified by MS in 3D^pol^ neddylation, Co-IP was carried out. We show that TRIM4 interacted with 3D^pol^ and enhanced 3D^pol^ neddylation in a dose-dependent manner (Fig. 7B through D). To evaluate the functional involvement of TRIM4 in 3D^pol^ neddylation, cells were transfected with the construct expressing the mutant TRIM4 (TRIM4-C27S), which lost its E3 ligase activity (32). We found that compared to the wild-type TRIM4, the mutant TRIM4 displayed weakened capability to enhance 3D^pol^ neddylation (Fig. 7E), suggesting that the E3 ligase activity of TRIM4 is indispensable for mediating 3D^pol^ neddylation.

Although the observations above show that TRIM4 mediates 3D^pol^ neddylation, we cannot exclude that there are other E3 ligases that also contribute to this process. To this end, we determined the impact of TRIM40 and RNF169 on 3D^pol^ NEDDylation. It has been shown that TRIM40 suppresses antiviral response through mediating the ubiquitination of RIG-I (33), indicating that TRIM40 might be exploited by viruses. RING finger proteins (RNF) show functional flexibility as the E3 ligases for ubiquitination, SUMOylation, and ISGylation (34), implicating the possible involvement of RNFs in neddylation. Therefore, we tested the correlation of these E3 ligases, TRIM40 and RNF169, with 3D^pol^ neddylation (Fig. 7F and G). In addition, E3 ligase c-CBL was selected as a negative control (Fig. 7H). Our results showed that overexpression of TRIM40, RNF169, or c-CBL did not alter the levels of neddylated Flag-3D (Fig. 7F through H).

To further study the interaction of 3D^pol^ and TRIM4, HEK293T cells were co-transfected with constructs expressing EGFP-3D and mCherry-TRIM4. The result showed that 3D^pol^ and TRIM4 were colocalized in the cytoplasm (Fig. 7I). Overall, these observations demonstrate that TRIM4, which interacts with and promotes 3D^pol^ neddylation, likely functions as the E3 ligase of NEDD8-conjugation.

### TRIM4 promotes CVB3 replication

**Fig 8:**
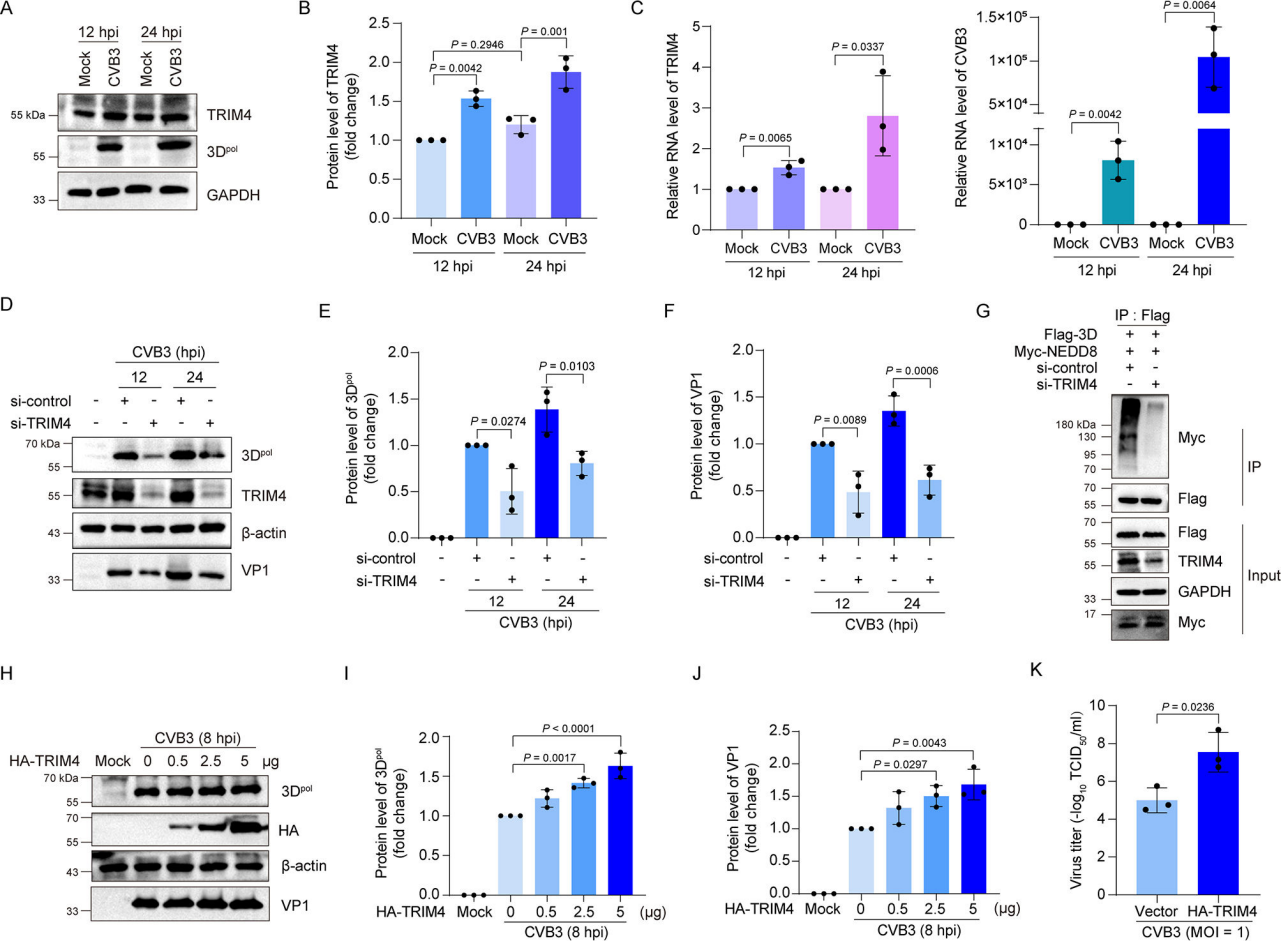
TRIM4 promotes CVB3 replication. (**A–C**) HEK293T cells were mock-infected or infected with CVB3 (MOI = 1) for 12 or 24 h. The cell lysates were harvested and subjected to immunoblotting (**A, B**) and RT-qPCR (**C**), respectively. (**D–F**) HEK293T cells were transfected with control siRNA or TRIM4 siRNA for 24 h and then infected with CVB3 (MOI = 1) for 12 or 24 h. The cells were harvested for immunoblotting. (**G**) HEK293T cells were transfected with control siRNA or TRIM4 siRNA for 24 h and then transfected with plasmids expressing Flag-3D and Myc-NEDD8 for 24 h. The cell lysates were subjected to immunoprecipitation with anti-Flag antibody. The precipitated proteins were resolved by SDS-PAGE and probed with the indicated specific antibodies. (**H–J**) HEK293T cells were transfected with the plasmids expressing HA-TRIM4 at increasing quantity (0.5, 2.5, and 5 µg) for 36 h, and then the cells were infected with CVB3 (MOI = 10) for 8 h. Total cellular proteins were extracted and analyzed by immunoblotting. (**K**) HEK293T cells were transfected with pHA-TRIM4 plasmid for 36 h and then infected with CVB3 (MOI = 1) for 24 h. Cells were collected and subjected to TCID_50_ assay to determine virus yield. Three independent experiments were performed, and representative results were presented. Quantitative analysis of the immunoblotting results was carried out by ImageJ. hpi: hours post-infection.

It has been reported that TRIM4 is required for EV-A71 replication with an unknown mechanism (35), and our results suggest that TRIM4 also plays a role in CVB3 infection. To show how TRIM4 would influence CVB3 infection, viral replication was determined in the context of either knockdown or overexpression of TRIM4. As shown in Fig. 8, changes in TRIM4 expression were determined in CVB3-infected cells (Fig. 8A through C). We found that, compared with the mock-infected cells, virus infection induced a significant increase in TRIM4 expression (Fig. 8A through C). Furthermore, in the cells with TRIM4 knockdown, viral protein 3D^pol^ and VP1 were markedly reduced (Fig. 8D through F), indicating that TRIM4 plays a critical role for the efficient replication of CVB3. Moreover, knockdown of TRIM4 almost completely blocked 3D^pol^ neddylation (Fig. 8G), demonstrating that TRIM4 is essential for 3D^pol^ neddylation.

The impact of TRIM4 on virus infection was further determined in the cells with TRIM4 overexpression. We found that overexpression of TRIM4 promoted the levels of viral proteins 3D^pol^ and VP1 in a dose-dependent manner (Fig. 8H through J). Evidently, increased virus yield was also observed in the cells overexpressing TRIM4 (Fig. 8K). Collectively, these data demonstrate that TRIM4 promotes CVB3 replication through mediating 3D^pol^ neddylation.

## DISCUSSION

Viruses optimize the cellular environment for efficient replication. NEDD8, one of the ubiquitin-like molecules, shares structural similarity with Ub. Neddylation, the covalent linkage of NEDD8 with the specific lysine residue of the substrate proteins, plays an important role in regulating the stability, subcellular localization, and function of proteins (23). Our previous study showed that the 3D^pol^ of CVB3 is ubiquitinated at K220, leading to upregulated degradation of 3D^pol^ through proteasomal activity (29). However, whether or not 3D^pol^ of CVB3 is modified by covalent linkage with NEDD8, which shares up to 60% sequence identity to Ub, remains unknown. This study shows that 3D^pol^ of CVB3 is neddylated at both K261 and K457 mediated by E3 ligase TRIM4. Neddylation enhances 3D^pol^ stability and promotes viral replication.

PTM is a strategy used by viruses to facilitate the viral life cycle and to optimize the host environment. Studies have implicated that neddylation plays essential roles in the life cycle of viruses such as HBV, IAV, and EV-A71 (25–28, 36). For instance, the neddylated X protein (HBx) of HBV exhibited enhanced stability and chromatin localization, which in turn promoted viral replication and HBV-induced tumor growth (25). In contrast, the neddylation of PB2 and M1 protein of IAV negatively regulates viral replication (26, 27). Similarly, it was reported that the capsid protein VP2 of EV-A71 is neddylated, leading to the reduced stability of VP2 and inhibited viral replication (28). Pharmacological inhibition of neddylation with MLN4924, the selective inhibitor of NAE, enhanced EV-A71 replication. However, another study obtained a contradictory conclusion that neddylation promoted EV-A71 replication (37). Nonetheless, according to these reports and our previous study, in which the ubiquitination of 3D^pol^ of CVB3 has been demonstrated (29), we postulated that 3D^pol^ might also be modified by neddylation.

During the infection of enteroviruses, multiple host proteins are involved in every step of the viral life cycle (38). We previously demonstrated that TRIM56 mediates the ubiquitination of 3D^pol^ and promotes 3D^pol^ proteasomal degradation (29). In spite of this, viral replication is not compromised, suggesting that a counteractive strategy might exist to ensure the stability of 3D^pol^ and the successful replication of CVB3. Importantly, our proteomics study using liquid chromatography−tandem mass spectrometry (LC-MS) identified that NEDD8 level was increased significantly in CVB3-infected cells (Table S1). A subsequent verification study demonstrated that it is the overall impact of CVB3 replication rather than the expression of individual viral protein that upregulated NEDD8 expression. Concerning that NEDD8 is homologous to Ub in structure and sequence and that neddylation often stabilizes substrate proteins, we hypothesized that 3D^pol^ might be neddylated. Co-IP analysis identified that 3D^pol^ is covalently conjugated to NEDD8 in CVB3-infected cells. Furthermore, we demonstrated that overexpression of either NAE1 or NEDD8 significantly elevated the virus titer, while knockdown of NAE1 or NEDD8 inhibited viral replication. Moreover, the CVB3 growth rate was significantly decreased when 3D^pol^ lost its neddylation sites at K261 and/or K457. These data demonstrated that 3D^pol^ neddylation facilitates CVB3 replication.

PTM affects almost all aspects of protein function (39). Similar to ubiquitination, neddylation is the conjugation of NEDD8 with the specific lysine residue of the substrate protein via an isopeptide bond (40). Neddylation often changes the stability, intracellular localization, and enzymatic function of the substrate protein (23). Protein stability is crucial for the normal function of the cell, and abnormally activated proteasomal degradation often leads to diseases (41). 3D^pol^, as the RdRp of CVB3, determines the synthesis rate of the viral genome and subsequent translation of viral proteins. Therefore, it is not surprising that the stability of 3D^pol^ is maintained by PTM. We previously demonstrated that TRIM56 mediates the ubiquitination and proteasomal degradation of 3D^pol^ (29). With the identification that 3D^pol^ of CVB3 is also neddylated, we wondered how these different modification processes interact in regulating the stability of 3D^pol^. To get an initial understanding of this issue, we determined the ubiquitination of 3D^pol^ in the context of NEDD8 overexpression (Fig. S1). Our data show that the ubiquitination of 3D^pol^ was declined along with the increased expression of NEDD8 in a dose-dependent pattern. These data are consistent with the favorable effect of the upregulated expression of NEDD8 on CVB3 replication, while an in-depth study is needed to reveal how neddylation is interfering with the ubiquitination of 3D^pol^.

It has been reported that neddylation promotes the nuclear import of the transforming growth factor-β (TGF-β)-activated kinase 1 (TAK1) (42). In addition, it has been reported that 3D^pol^ and its precursor 3 CD of poliovirus are capable of entering the nucleus in virus-infected cells, where the viral 3C^pro^, matured from 3 CD, may cleave nuclear proteins (43). Therefore, to further investigate the impact of 3D^pol^ neddylation on the host cell as well as virus, we observed the cellular localization of the neddylated 3D^pol^ (Fig. S2). We found that, although a small portion of 3D^pol^ is located in the nucleus in a dotted pattern, the neddylated 3D^pol^ is primarily localized in the cytoplasm. This finding implies that neddylation might be critical for 3D^pol^ to function as RdRp since the genome replication of enterovirus occurs in the cytoplasm.

Due to the sequence and structural homology of 3D^pol^ among enteroviruses (44), we initially supposed that other enteroviruses might also contain 3D^pol^ neddylation. It was reported that the 3D^pol^ of EV-A71 contains phosphorylation at S184, which is essential for viral replication (45). However, we found that 3D^pol^ neddylation was absent in either CV-A16 and EV-A71, suggesting that the neddylation site is unique for CVB3 3D^pol^. Through comparing the amino acid sequences of these enteroviruses, we identified 12 lysine residues that are found only in CVB3. Subsequent analysis demonstrated that neddylation occurs at K261 and K457 of CVB3 3D^pol^. We also show that the mutated CVB3, which lost its two neddylation sites in 3D^pol^, exhibited much slower growth kinetics than the wild-type virus. Even with one mutated neddylation site, virus yield was significantly reduced, demonstrating that both neddylation sites of 3D^pol^ are critical for viral replication. In addition, we found that CVB5 infection upregulates neddylation. Sequence alignment shows that there is more than 95% identity in the 3D^pol^ sequence of different types of CVB, while K261 and K457 are well conserved (Fig. S3). These data imply that 3D^pol^ neddylation is shared by all types of CVB.

To further consolidate that neddylation facilitates CVB replication, MLN4924 was used to show the impact of neddylation on virus replication (Fig. S4). We found that MLN4924 suppressed the replication of both wild-type and mutated CVB3 (CVB3-3D^K261R^ or CVB3-3D^K457R^), further demonstrating that neddylation of 3D^pol^ is critical for CVB replication.

This study demonstrated that TRIM4 is the E3 ligase that mediates the neddylation of 3D^pol^ of CVB3. To search for the E3 ligase that mediates the neddylation of 3D^pol^, an MS study was carried out to identify the proteins that interact with 3D^pol^ in CVB3-infected cells (Table S2). Until now, all the known E3 ligases that mediate the final step of neddylation are Ub E3 ligases. Therefore, we paid special attention to the E3 ligases in the MS data. We confirmed that TRIM4 specifically interacts with and enhances 3D^pol^ neddylation, while other E3 ligases do not. Moreover, E3 ligase-deficient TRIM4 failed to upregulate 3D^pol^ neddylation. These data show that the promoting effect of TRIM4 on 3D^pol^ neddylation depends on its E3 ligase activity. In addition, we demonstrated that NEDP1, the highly conserved deneddylation cysteine protease, completely blocked 3D^pol^ neddylation, while the mutated NEDP1, which lacks the enzymatic activity, did not. However, further study is needed to characterize the intrinsic role of the NEDP1 during CVB3 infection. Overall, these observations implicate that the key enzymes involved in neddylation, including NAE, NEDD8-conjugating enzyme E2, TRIM4, and NEDP1, are critical for CVB infection.

The interaction and the colocalization of TRIM4 and 3D^pol^ were confirmed in this study. However, which domain of TRIM4 is interacting with 3D^pol^ was not determined. It is also unclear whether or not the interaction between 3D^pol^ and TRIM4 is facilitated by other cellular or viral proteins. Our MS analysis identified up to hundreds of proteins that are interacting with 3D^pol^ in CVB3-infected cells (Table S2). Except for the ribosomal proteins required for viral translation, which are almost co-existing with viral genome synthesis at the same intracellular space, other proteins identified by MS may also be involved in the stability, modification, or localization of 3D^pol^. Therefore, further in-depth study is needed.

Here, we identified the neddylation of 3D^pol^ of CVB. However, it is unknown whether or not neddylation also occurs in other proteins of CVB. It has been demonstrated that VP2 of EV-A71 is neddylated at the lysine residue at position 69 (28). Through comparing the amino acid sequence of VP2 between CVB3 and EV-A71, we found that K69 is unique for the VP2 of EV-A71 (Fig. S5). However, the presence or absence of neddylation for other proteins of CVB still needs further investigation.

In summary, this study demonstrated that the 3D^pol^ protein of CVB3 can be modified by NEDD8 at its lysine residues 261 and 457. The neddylation of 3D^pol^, which is mediated by the E3 ligase TRIM4, promotes its stability and facilitates CVB3 replication. Our findings not only revealed a novel mechanism involved in CVB3 pathogenesis but also suggest that targeting the process of neddylation might be a potential antiviral strategy against CVB3 infection.

## MATERIALS AND METHODS

### Cell culture

HeLa and HEK293T cells were maintained and cultured in Dulbecco’s modified Eagle medium (DMEM, Gibco) supplemented with 10% (vol/vol) fetal bovine serum (FBS, Bioindustry, Israel) and 1% antibiotics (100 µg/mL penicillin and 100 µg/mL streptomycin) at 37°C in a 5% CO_2_ incubator and passaged every 2 days. After virus inoculation, cells were maintained in the medium containing 2% FBS.

### Virus

CVB3 Woodruff strain was kindly provided by the Scrips Research Institute (San Diego, USA). CVB5 was provided by Professor Shen of Jiangsu University, Zhenjiang, China. To amplify viruses, HeLa cells were infected with CVB3 or CVB5 for 24 h. Cell cultures were subjected to three freeze-thaw cycles and centrifuged at 12,000 × *g* (Thermo Fisher, Waltham, MA) for 5 min to obtain the virus stock solution, which was stored at −80°C. The virus titer was determined by the 50% tissue culture infective dose (TCID_50_) assay as described previously (46). In this study, the TCID_50_ of CVB3 was 1 × 10^-6.5^/mL. TCID_50_ of CVB5 was 1 × 10^−5^/mL.

### Chemical and antibodies

MLN4924 (Abmole, Shanghai, China) was dissolved in DMSO to prepare the stock solution (100 mM) and stored at −80°C. The working solution of MLN4924 was prepared with DMEM and used only once to avoid inactivation. Antibodies against NEDD8, GAPDH, β-actin, β-tubulin, Myc, Flag, HA, EGFP, and mCherry were purchased from Proteintech (Wuhan, China). Antibodies against V5, horseradish peroxidase (HRP)-conjugated goat anti-mouse IgG and HRP-conjugated goat anti-rabbit IgG were purchased from Servicebio (Wuhan, China). Antibody against TRIM4 was purchased from CUSABIO (Wuhan, China). Polyclonal antibodies against 3D^pol^, 3C, and VP1 of CVB3 were prepared in our laboratory.

### Plasmids and siRNAs

The plasmids expressing Myc-NEDD8, mCherry-NEDD8, Flag-NAE1, HA-TRIM4, HA-Ub, and V5-NEDP1 were purchased from Miaoling (Wuhan, China). Plasmids pMKS1-CVB3, pEGFP-C1, pEGFP-2A, pEGFP-2B, pEGFP-2C, pEGFP-3A, pEGFP-3B, pEGFP-3C, pEGFP-3D, pEGFP-VP1, and pEGFP-VP2 ~4 were kindly provided by Professor Zhaohua Zhong, Department of Microbiology, Harbin Medical University. The plasmids expressing EGFP-3D K22R, -K51R, -K96R, -K133/134R, -K196R, -K250/255R, -K261R, -K406/409R, -K436, -K457R, -K261/457R, V5-NEDP1-C163A, and HA-TRIM4-C27S were generated by site-specific mutagenesis with PrimeSTAR Max Premix (Takara, Beijing, China) by following the manufacturer’s directions. The plasmids expressing Flag-3D of CVB3, Flag-3D of EV-A71, EGFP-3D of CV-A16, and Myc-3CD were constructed based on pcDNA3.1-EGFP. The plasmid expressing mCherry-TRIM4 was constructed based on pcDNA3.1-mCherry. NEDD8 siRNA was purchased from Ribobio Technology (Guangzhou, China). The other siRNAs were synthesized by GenePharma (Shanghai, China). The siRNA sequences are listed in Table 1.

**TABLE 1:** siRNA sequences

siRNA	Nucleotide sequence (5’→3’)
si-Control	
Sense Antisense	UUCUCCGAACGUGUCACGUTT ACGUGACACGUUCGGAGAATT
si-NAE1	
Sense Antisense	GCUCGUGCCUUAAAGGAAUTT AUUCCUUUAAGGCACGAGCTT
si-NEDD8	
Sense Antisense	CAGUCCUUCACCUGGUGUUTT AACACCAGGUGAAGGACUGTT
si-TRIM4	
Sense Antisense	GGAAGUUGAGAGUAGAGAUTT AUCUCUACUCUCAACUUCCTT

### Transfection

HEK293T cells were cultured in 6-well plates to 50% ~ 60% confluence 24 h prior to transfection. The transfection mix was prepared with plasmids and polyethyleneimine (PEI) (Polysciences, USA) in the ratio of 1 : 2.5 dissolved in 1 mL of DMEM, and cells were incubated with the mixture for 6 h at 37°C. After transfection, cells were cultured in fresh medium for 36 to 48 h and then harvested for immunoblotting and RT-qPCR. siRNA (25 nmol) was transfected into cells with X-tremeGene siRNA Transfection Reagent, according to the instructions recommended by the manufacturer. Cells were harvested at 24 h after transfection for further analysis.

### Cytotoxicity assay

HEK293T cells were plated into 96-well plates and cultured to 80% of confluence. Cells were treated with various concentrations of MLN4924 for 24 h. The cell viability was determined with the methylthiazolydiphenyl-tetrazolium bromide (MTT) (Yeasen, Shanghai, China) according to the protocol recommended by the provider. The MTT solution was diluted with DMEM to a concentration of 10% and then added to each well of the culture plate, which was measured by a microplate reader Epoch2 (BioTek) at 570 nm.

### Proteasomal degradation assay

MG132 (Selleck, Shanghai, China) was dissolved in DMSO to prepare the stock solution (10 mM), which was stored at −80°C. The fresh working solution of MG132 (10 µM) was prepared with DMEM. Six hours before the endpoint of the culture, cells were treated with MG132. Cells were harvested for further analysis.

### Cycloheximide chase assay

Cycloheximide (CHX) (Abmole, Shanghai, China) was dissolved in DMSO to 10 mM stock solutions (stored at −80°C). The working concentration of CHX was 100 nM, which was diluted with DMEM and used only once to avoid inactivation. HEK293T cells were used to carry out CHX chase assay after transfection. The cell lysates were prepared at different time points after CHX treatment and analyzed by immunoblotting.

### RNA extraction, reverse transcription, and real-time quantitative PCR

Total RNA was extracted by TRIzol (Yeasen, Shanghai) according to the protocol recommended by the providers, and the concentration of RNA was quantified by NanoDrop 2000 (Thermo Fisher). The reverse transcription system was prepared with 1,000 ng RNA, 1 µL of gDNA remover, and 4 µL of 5 × Trans Script All-in-One Super Mix (TransGen, Beijing, China) and was made up to 20 µL with RNase-free sterile water. The mixture was carried out at 50°C for 5 min, followed by heating at 85°C for 2 min to create cDNA. Twenty microliters of quantitative PCR system consisted of 1 µL of cDNA, 0.4 µL of forward and reverse primers (10 µM), 10 µL of 2 × TransStart Top Green qPCR Super Mix (*Trans* Gen, Beijing, China), and 8.2 µL RNase-free sterile water.

Quantitative PCR was performed in LightCycler 96 (Roche, Basel, Switzerland) for 45 cycles. Each amplification cycle contained denaturation at 94°C for 5 s, annealing at 58°C for 15 s, and extension at 72°C for 1 min. Relative RNA quantity was calculated using the 2^-ΔΔCT^ method and normalized to the quantity of GAPDH. Primers were synthesized by Comate Bio (Jilin, China). The sequences of primers for PCR are listed in Table 2.

**TABLE 2:** Primer sequences

Primer	Sequence (5’→3’)
CVB3 forward	GCACACACCCTCAAACCAGA
CVB3 reverse	ATGAAACACGGACACCCAAAG
GAPDH forward (Homo)	CTGGGCTACACTGAGCACC
GAPDH reverse (Homo)	AAGTGGTCGTTGAGGGCAATG
NAE1 forward (Homo)	CCTGTTCGAGGCACAATTCCTG
NAE1 reverse (Homo)	TTACCCACAGCGGCAGCATCTT
NEDD8 forward (Homo)	GTGAAGACGCTGACCGGAAAGG
NEDD8 reverse (Homo)	CCTCCACACGCTCCTTGATTCG

### Immunoblotting

Cells were cultured in 6 or 12-well plates to 80% confluency and lysed by using RIPA lysis buffer (Beyotime, Beijing, China) containing 1% protease inhibitor PMSF (Beyotime) on ice for 20 min and then treated with ultrasound under the power of 120W for 8 s, repeated three times. The cell lysates were centrifuged at 12,000 × *g* at 4°C for 15 min to collect the supernatant, and the concentration of protein samples was detected by using a BCA protein assay kit (Beyotime). Proteins were separated on 10% or 12.5% sodium dodecyl sulfate-polyacrylamide gel (SDS-PAGE). After electrophoresis, proteins were transferred from gel to polyvinylidene difluoride (PVDF) membranes (Millipore, USA), which were blocked with skimmed milk for 1 h and then incubated with the indicated primary antibody at 4°C overnight. The membranes were washed three times with 0.5% Tween-20 in TBST and incubated with the secondary antibody (anti-mouse or anti-rabbit IgG) for 1 h at room temperature. Finally, the blots were visualized by Tanon 5200 Chemiluminescent Imaging System (Biotanon, Shanghai, China) and analyzed by ImageJ.

### Immunoprecipitation

To explore the interaction between two ectopic expression proteins, HEK293T cells were transfected with the indicated plasmids for 36 or 48 h and then washed twice with cold PBS. The cell lysates were collected with NP40 lysis buffer (Beyotime) containing 1% protease inhibitor PMSF (Beyotime). Soluble lysates were incubated on ice for 30 min and then centrifuged at 12,000 × *g* at 4°C for 15 min to collect the supernatant. After determining the protein concentration, the samples were incubated with anti-Flag or anti-Myc magnetic beads (MCE, Shanghai, China) at room temperature for 2 h with rotation. The control cell lysates were incubated with mouse IgG (Beyotime) at 4°C overnight with rotation, followed by incubation of protein A/G agarose beads (MCE). The precipitated mixture was placed on a magnetic stand to remove the supernatant, and the protein-magnetic bead mixture was washed with IP washing buffer 10 times by gently pipetting. Finally, the beads were incubated with 100 µL elution buffer at 100°C for 10 min. The precipitated proteins were collected for immunoblotting.

To identify the linkage between NEDD8 and 3D of CVB3, HeLa cells were infected with CVB3 (MOI = 10) for 8 or 12 h and collected with NP40 lysis buffer (Beyotime) containing 1% protease inhibitor PMSF (Beyotime). The cell lysates were incubated with 4 µg of 3D polyclonal antibody or mouse IgG (Beyotime) at 4°C overnight on a rotating mixer, followed by incubation of protein A/G agarose beads (MCE). The rest of the steps are the same as above.

### Fluorescence microscopy

HEK293T cells were grown on coverslips in 6-well plates and transfected with 1 µg of plasmid (pEGFP-3D, pmCherry-TRIM4 or pmCherry-NEDD8) using PEI according to the manufacturer’s protocol for 36 h. To visualize cells with fluorescence microscopy, cells were fixed with 4% paraformaldehyde for 20 min at room temperature. Nuclei were stained using 4’,6’-diamidino-2-phenylindole (DAPI) for 15 min. Coverslips were mounted with FluorSave (Calbiochem). Images were acquired with a Cell Voyager 1000 (Yokogawa, Japan) confocal laser scanning microscope.

### Mass spectrometry

HeLa cells were infected or mock-infected with CVB3 (MOI = 1) for 24 h. The cells were washed with cold PBS three times, and the cell lysates were prepared with NP40 lysis buffer (Beyotime) supplemented with 1% protease inhibitor PMSF (Beyotime). Cell lysates were analyzed by nanoscale liquid chromatography coupled to tandem mass spectrometry (EASY-nLC 1200, Thermo Fisher).

HEK293T cells were transfected with the constructing plasmid Flag-3D for 36 h, followed by CVB3 infection (MOI = 5) or mock-infection. At 18 h post-infection, the cells were lysed in NP40 lysis buffer (containing 1% PMSF) on ice for 30 min and then centrifuged at 12,000 × *g* for 15 min at 4℃. The supernatants were incubated with anti-Flag magnetic beads (MCE) for 2 h on a rotating mixer, and then the protein-bead mixture was washed with washing buffer for 10 times. The precipitated proteins were removed from the agarose beads and then trypsinized. The digested peptides were analyzed by HPLC-MS/MS.

### Production of wild-type and mutant CVB3 viruses

Full-length genomic cDNA of CVB3 was synthesized and cloned in the pMKS1 vector by GenScript (Nanjing, China), yielding plasmid pMKS1-CVB3-WT. A T7 promoter was placed at the 5’ end for *in vitro* transcription. The point mutations were introduced into this plasmid using PrimeSTAR Max Premix (Takara, Beijing, China), and the desired sequence alterations were confirmed by DNA sequencing. To recover CVB3 viruses, the plasmids containing the wild-type or mutant CVB3 genomic cDNA were transfected into HEK293T cells using PEI. The CVB3 viruses were collected 3 days post-transfection and propagated in HeLa cells. The wild-type or mutant CVB3 viruses were collected by three freeze-thaw cycles and titrated by TCID_50_ assay.

### Viral growth kinetics

HeLa cells were infected with wild-type CVB3 or mutant virus CVB3-3D^K261R^, CVB3-3D^K457R^, and CVB3-3D^K261/457R^ at an MOI of 0.1. At 1 h post-infection, the cells were cultured in fresh DMEM with 2% FBS at 37°C for 12, 24, 36, or 48 h. The supernatants were subjected to three freeze-thaw cycles, and virus titers were calculated by TCID_50_ assay.

### Statistical analysis

All experiments were repeated three times. Data were analyzed by Graphpad Prism 8, and the quantitative data were presented as mean ± SD. Student’s *t* test, one-way ANOVA, and two-way ANOVA were used for statistical analyses. *P* < 0.05 is considered statistically significant.

## Data Availability

Data in the study are openly available at https://data.mendeley.com/datasets/czn5zyj6zy/2.

## SUPPLEMENTAL MATERIAL

Supplemental figures jvi.01535-25-s0001.docx Figures S1 to S5.

Table S1 jvi.01535-25-s0002.pdf Data of mass spectrometry for CVB3-infected cells.

Table S2 jvi.01535-25-s0003.pdf Data of mass spectrometry for the proteins precipitated with 3D^pol^.

ASM does not own the copyrights to Supplemental Material that may be linked to, or accessed through, an article. The authors have granted ASM a non-exclusive, world-wide license to publish the Supplemental Material files. Please contact the corresponding author directly for reuse.

## References

[1] Garmaroudi FS, Marchant D, Hendry R, Luo H, Yang D, Ye X, Shi J, McManus BM. 2015. Coxsackievirus B3 replication and pathogenesis. Future Microbiol 10:629–653. doi:10.2217/fmb.15.5 25865198

[2] Weng S, Zhu R, Wu Y, Xia N, Xu L, Cheng T. 2025. Research progress and application prospects of animal models of group B Coxsackievirus infections. Emerg Microbes Infect 14:2441391. doi:10.1080/22221751.2024.2441391 39665300 PMC11703136

[3] Kim KS, Hufnagel G, Chapman NM, Tracy S. 2001. The group B coxsackieviruses and myocarditis. Rev Med Virol 11:355–368. doi:10.1002/rmv.326 11746998

[4] Gauntt CJ, Paque RE, Trousdale MD, Gudvangen RJ, Barr DT, Lipotich GJ, Nealon TJ, Duffey PS. 1983. Temperature-sensitive mutant of coxsackievirus B3 establishes resistance in neonatal mice that protects them during adolescence against coxsackievirus B3-induced myocarditis. Infect Immun 39:851–864. doi:10.1128/iai.39.2.851-864.1983 6299950 PMC348027

[5] Yang Q, Yan D, Song Y, Zhu S, He Y, Han Z, Wang D, Ji T, Zhang Y, Xu W. 2022. Whole-genome analysis of coxsackievirus B3 reflects its genetic diversity in China and worldwide. Virol J 19:69. doi:10.1186/s12985-022-01796-0 35436962 PMC9014606

[6] Bouin A, Gretteau P-A, Wehbe M, Renois F, N’Guyen Y, Lévêque N, Vu MN, Tracy S, Chapman NM, Bruneval P, Fornes P, Semler BL, Andreoletti L. 2019. Enterovirus persistence in cardiac cells of patients with idiopathic dilated cardiomyopathy is linked to 5' terminal genomic RNA-deleted viral populations with viral-encoded proteinase activities. Circulation 139:2326–2338. doi:10.1161/CIRCULATIONAHA.118.035966 30755025 PMC6517084

[7] Gaaloul I, Riabi S, Harrath R, Hunter T, Hamda KB, Ghzala AB, Huber S, Aouni M. 2014. Coxsackievirus B detection in cases of myocarditis, myopericarditis, pericarditis and dilated cardiomyopathy in hospitalized patients. Mol Med Rep 10:2811–2818. doi:10.3892/mmr.2014.2578 25241846 PMC4227425

[8] Muckelbauer JK, Kremer M, Minor I, Diana G, Dutko FJ, Groarke J, Pevear DC, Rossmann MG. 1995. The structure of coxsackievirus B3 at 3.5 A resolution. Structure 3:653–667. doi:10.1016/s0969-2126(01)00201-5 8591043

[9] Bouin A, Nguyen Y, Wehbe M, Renois F, Fornes P, Bani-Sadr F, Metz D, Andreoletti L. 2016. Major persistent 5' terminally deleted Coxsackievirus B3 populations in human endomyocardial tissues. Emerg Infect Dis 22:1488–1490. doi:10.3201/eid2208.160186 27434549 PMC4982168

[10] Huber SA, Gauntt CJ, Sakkinen P. 1998. Enteroviruses and myocarditis: viral pathogenesis through replication, cytokine induction, and immunopathogenicity. Adv Virus Res 51:35–80. doi:10.1016/s0065-3527(08)60783-6 9891585

[11] Song QQ, Luo XN, Shi BT, Liu M, Song J, Xia D, Xia ZQ, Wang WJ, Yao HL, Han J. 2022. Exploration of IRES elements within the ORF of the Coxsackievirus B3 genome. Biomed Environ Sci 35:322–333. doi:10.3967/bes2022.043 35473896

[12] Chen Y, Li X, Han F, Ji B, Li Y, Yan J, Wang M, Fan J, Zhang S, Lu L, Zou P. 2024. The nucleoside analog 4’-fluorouridine suppresses the replication of multiple enteroviruses by targeting 3D polymerase. Antimicrob Agents Chemother 68:e0005424. doi:10.1128/aac.00054-24 38687016 PMC11620493

[13] Bedard KM, Semler BL. 2004. Regulation of picornavirus gene expression. Microbes Infect 6:702–713. doi:10.1016/j.micinf.2004.03.001 15158778

[14] Xu Y, Wu W, Han Q, Wang Y, Li C, Zhang P, Xu H. 2019. Post-translational modification control of RNA-binding protein hnRNPK function. Open Biol 9:180239. doi:10.1098/rsob.180239 30836866 PMC6451366

[15] Kumar R, Mehta D, Mishra N, Nayak D, Sunil S. 2020. Role of host-mediated post-translational modifications (PTMs) in RNA virus pathogenesis. Int J Mol Sci 22:323. doi:10.3390/ijms22010323 33396899 PMC7796338

[16] van der Veen AG, Ploegh HL. 2012. Ubiquitin-like proteins. Annu Rev Biochem 81:323–357. doi:10.1146/annurev-biochem-093010-153308 22404627

[17] Kamitani T, Kito K, Nguyen HP, Yeh ET. 1997. Characterization of NEDD8, a developmentally down-regulated ubiquitin-like protein. J Biol Chem 272:28557–28562. doi:10.1074/jbc.272.45.28557 9353319

[18] Kamada S. 2013. Inhibitor of apoptosis proteins as E3 ligases for ubiquitin and NEDD8. Biomol Concepts 4:161–171. doi:10.1515/bmc-2012-0036 25436573

[19] Zhou L, Zhang W, Sun Y, Jia L. 2018. Protein neddylation and its alterations in human cancers for targeted therapy. Cell Signal 44:92–102. doi:10.1016/j.cellsig.2018.01.009 29331584 PMC5829022

[20] Walden H, Podgorski MS, Huang DT, Miller DW, Howard RJ, Minor DL, Holton JM, Schulman BA. 2003. The structure of the APPBP1-UBA3-NEDD8-ATP complex reveals the basis for selective ubiquitin-like protein activation by an E1. Mol Cell 12:1427–1437. doi:10.1016/s1097-2765(03)00452-0 14690597

[21] Huang DT, Paydar A, Zhuang M, Waddell MB, Holton JM, Schulman BA. 2005. Structural basis for recruitment of Ubc12 by an E2 binding domain in NEDD8’s E1. Mol Cell 17:341–350. doi:10.1016/j.molcel.2004.12.020 15694336

[22] Gong L, Yeh ET. 1999. Identification of the activating and conjugating enzymes of the NEDD8 conjugation pathway. J Biol Chem 274:12036–12042. doi:10.1074/jbc.274.17.12036 10207026

[23] Zhao Y, Morgan MA, Sun Y. 2014. Targeting Neddylation pathways to inactivate cullin-RING ligases for anticancer therapy. Antioxid Redox Signal 21:2383–2400. doi:10.1089/ars.2013.5795 24410571 PMC4241876

[24] Enchev RI, Schulman BA, Peter M. 2015. Protein neddylation: beyond cullin-RING ligases. Nat Rev Mol Cell Biol 16:30–44. doi:10.1038/nrm3919 25531226 PMC5131867

[25] Liu N, Zhang J, Yang X, Jiao T, Zhao X, Li W, Zhu J, Yang P, Jin J, Peng J, Li Z, Ye X. 2017. HDM2 promotes NEDDylation of hepatitis B virus HBx to enhance its stability and function. J Virol 91:e00340-17. doi:10.1128/JVI.00340-17 28592528 PMC5533936

[26] Zhang T, Ye Z, Yang X, Qin Y, Hu Y, Tong X, Lai W, Ye X. 2017. NEDDylation of PB2 reduces its stability and blocks the replication of influenza A virus. Sci Rep 7:43691. doi:10.1038/srep43691 28252002 PMC5333077

[27] Li Y, Chai W, Min J, Ye Z, Tong X, Qi D, Liu W, Luo E, Li J, Ye X. 2020. Neddylation of M1 negatively regulates the replication of influenza A virus. J Gen Virol 101:1242–1250. doi:10.1099/jgv.0.001503 33016861

[28] Wang H, Zhong M, Cui B, Yan H, Wu S, Wang K, Li Y. 2022. Neddylation of Enterovirus 71 VP2 protein reduces its stability and restricts viral replication. J Virol 96:e0059822. doi:10.1128/jvi.00598-22 35510863 PMC9131864

[29] Wang Y, Dong Y, Luan T, Chen Y, Lin L, Li S, Feng D, Wei J, Fei Y, Wang G, Pan J, Wang Y, Zhong Z, Zhao W. 2024. TRIM56 restricts Coxsackievirus B infection by mediating the ubiquitination of viral RNA-dependent RNA polymerase 3D. PLoS Pathog 20:e1012594. doi:10.1371/journal.ppat.1012594 39348396 PMC11476688

[30] Mendoza HM, Shen LN, Botting C, Lewis A, Chen J, Ink B, Hay RT. 2003. NEDP1, a highly conserved cysteine protease that deNEDDylates Cullins. J Biol Chem 278:25637–25643. doi:10.1074/jbc.M212948200 12730221

[31] Rabut G, Peter M. 2008. Function and regulation of protein neddylation. “Protein modifications: beyond the usual suspects” review series. EMBO Rep 9:969–976. doi:10.1038/embor.2008.183 18802447 PMC2572130

[32] Yan J, Li Q, Mao AP, Hu MM, Shu HB. 2014. TRIM4 modulates type I interferon induction and cellular antiviral response by targeting RIG-I for K63-linked ubiquitination. J Mol Cell Biol 6:154–163. doi:10.1093/jmcb/mju005 24755855

[33] Pan Q, Xie Y, Zhang Y, Guo X, Wang J, Liu M, Zhang XL. 2024. EGFR core fucosylation, induced by hepatitis C virus, promotes TRIM40-mediated-RIG-I ubiquitination and suppresses interferon-I antiviral defenses. Nat Commun 15:652. doi:10.1038/s41467-024-44960-6 38253527 PMC10803816

[34] Cai C, Tang YD, Zhai J, Zheng C. 2022. The RING finger protein family in health and disease. Signal Transduct Target Ther 7:300. doi:10.1038/s41392-022-01152-2 36042206 PMC9424811

[35] Li Y, Jian X, Yin P, Zhu G, Zhang L. 2019. Elucidating the host interactome of Enterovirus A71 2C reveals viral dependency factors. Front Microbiol 10:636. doi:10.3389/fmicb.2019.00636 31001221 PMC6454016

[36] Shizhen Zhang , Qing Yu , Zhijian Li , Yongchao Zhao， Yi Sun S, Yu Q, Li Z, Zhao Y, Sun Y. 2024. Protein neddylation and its role in health and diseases. Signal Transduct Target Ther 1:708–716. doi:10.1177/1947601910382898 PMC10995212 38575611

[37] Zhang Z, Guo H, Wang J, Li Y, Gao Y, Liu Q, Niu J, Wei W. 2021. Inhibition of the neddylation pathway suppresses Enterovirus replication. Virol Sin 36:1664–1667. doi:10.1007/s12250-021-00427-2 34351571 PMC8692535

[38] Huan C, Qu X, Li Z. 2022. Host restrictive factors are the emerging storm troopers against Enterovirus: a mini-review. Front Immunol 13:910780. doi:10.3389/fimmu.2022.910780 35603180 PMC9114347

[39] Lee JM, Hammarén HM, Savitski MM, Baek SH. 2023. Control of protein stability by post-translational modifications. Nat Commun 14:201. doi:10.1038/s41467-023-35795-8 36639369 PMC9839724

[40] Baek K, Scott DC, Schulman BA. 2021. NEDD8 and ubiquitin ligation by cullin-RING E3 ligases. Curr Opin Struct Biol 67:101–109. doi:10.1016/j.sbi.2020.10.007 33160249 PMC8096640

[41] Wang T, Jiang J, Zhang X, Ke X, Qu Y. 2024. Ubiquitin-like modification dependent proteasomal degradation and disease therapy. Trends Mol Med 30:1061–1075. doi:10.1016/j.molmed.2024.05.005 38851992

[42] Li S, Fang W, Cui Y, Shi H, Chen J, Li L, Zhang L, Zhang X. 2020. Neddylation promotes protein translocation between the cytoplasm and nucleus. Biochem Biophys Res Commun 529:991–997. doi:10.1016/j.bbrc.2020.07.012 32819610

[43] Sharma R, Raychaudhuri S, Dasgupta A. 2004. Nuclear entry of poliovirus protease-polymerase precursor 3CD: implications for host cell transcription shut-off. Virology (Auckl) 320:195–205. doi:10.1016/j.virol.2003.10.020 15016543

[44] Gruez A, Selisko B, Roberts M, Bricogne G, Bussetta C, Jabafi I, Coutard B, De Palma AM, Neyts J, Canard B. 2008. The crystal structure of coxsackievirus B3 RNA-dependent RNA polymerase in complex with its protein primer VPg confirms the existence of a second VPg binding site on Picornaviridae polymerases. J Virol 82:9577–9590. doi:10.1128/JVI.00631-08 18632861 PMC2546979

[45] Lin D, Dong X, Xiao X, Xiang Z, Lei X, Wang J. 2023. Proteomic and phosphoproteomic analysis of responses to enterovirus A71 infection reveals novel targets for antiviral and viral replication. Antiviral Res 220:105761. doi:10.1016/j.antiviral.2023.105761 37992763

[46] Wang Y, Zhao S, Chen Y, Wang Y, Wang T, Wo X, Dong Y, Zhang J, Xu W, Qu C, Feng X, Wu X, Wang Y, Zhong Z, Zhao W. 2020. N-Acetyl cysteine effectively alleviates Coxsackievirus B-Induced myocarditis through suppressing viral replication and inflammatory response. Antiviral Res 179:104699. doi:10.1016/j.antiviral.2019.104699 31883926

