# Supplementary material for: The neddylation of the RNA-dependent RNA polymerase 3D of Coxsackievirus B3 promotes viral replication
Source: J Virol. 2025 Oct 31;99(11):e01535-25. doi: 10.1128/jvi.01535-25 (PMC12646006; doi:10.1128/jvi.01535-25)

**The neddylation of the RNA-dependent RNA polymerase 3D of Coxsackievirus B3 promotes viral replication**

Siwei Li^a^, Yanyan Dong^a^, Xuexuan Wang^a^, Danxiang Feng^a^, Tian Luan^a^, Ziyuan Wang^a^, Lexun Lin^b^, Yang Chen^b^, Yao Wang^a^, Yanru Fei^c^, Yan Wang^c^, Zhaohua Zhong^c*^, Wenran Zhao^a*^

^a^Department of Cell Biology, The School of Basic Medical Sciences, Harbin Medical University, 157 Baojian Road, Harbin 150081, China

^b^Teaching Center of Pathogenic Biology, The School of Basic Medical Sciences, Harbin Medical University, 157 Baojian Road, Harbin 150081, China

^c^Department of Microbiology, School of Basic Medical Sciences, Harbin Medical University, 157 Baojian Road, Harbin 150081, China

*Correspondence should be addressed to:

Professor Wenran Zhao

Department of Cell Biology, School of Basic Medical Sciences, Harbin Medical University, 157 Baojian Road, Harbin 150081, China

E-mail: [zhaowr@hrbmu.edu.cn](mailto:zhaowr@hrbmu.edu.cn)

Professor Zhaohua Zhong

Department of Microbiology, School of Basic Medical Sciences, Harbin Medical University, 157 Baojian Road, Harbin 150081, China

E-mail: [zhongzh@hrbmu.edu.cn](mailto:zhongzh@hrbmu.edu.cn)

**Supplementary Materials**

**Fig. S1. The neddylation of viral 3D^pol^ inhibits its ubiquitination.** HEK293T cells were transfected with the indicated plasmids together with increasing amount of the plasmid expressing Myc-NEDD8 (1 μg and 2 μg) for 24 h, followed by the treatment of MG132 for 6 h. Total cellular proteins were extracted and subjected to denatured Co-IP with anti-Flag antibody. The precipitated proteins were separated by SDS-PAGE and probed with the indicated specific antibodies. Three independent experiments were performed and representative results were presented.

**
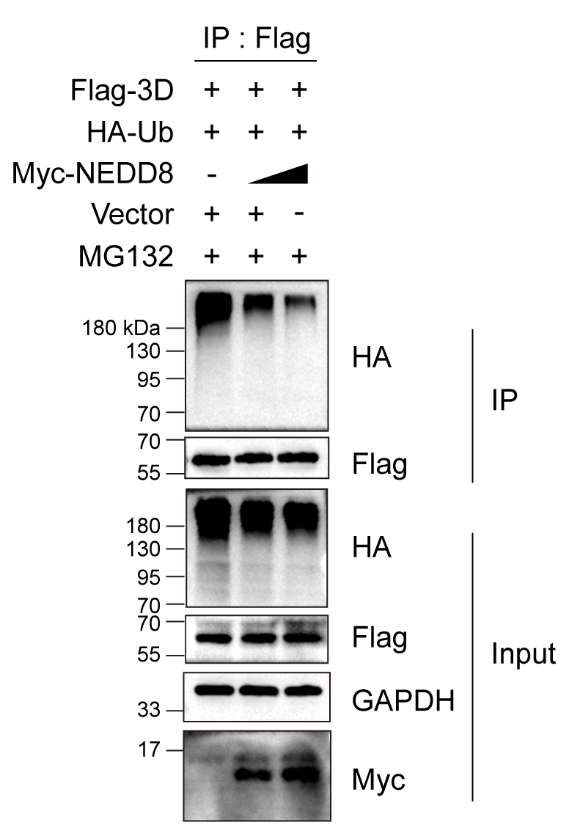
**

**Fig. S2. The cytoplasmic localization of the neddylated 3D^pol^**. HEK293T cells were cultured to 50% confluency. Cells were transfected with pEGFP-3D together with or without pmCherry-NEDD8 for 36 h. Cells were fixed and examined by immunofluorescent microscopy. Nuclei were stained with DAPI. Bar = 10 μm.

**
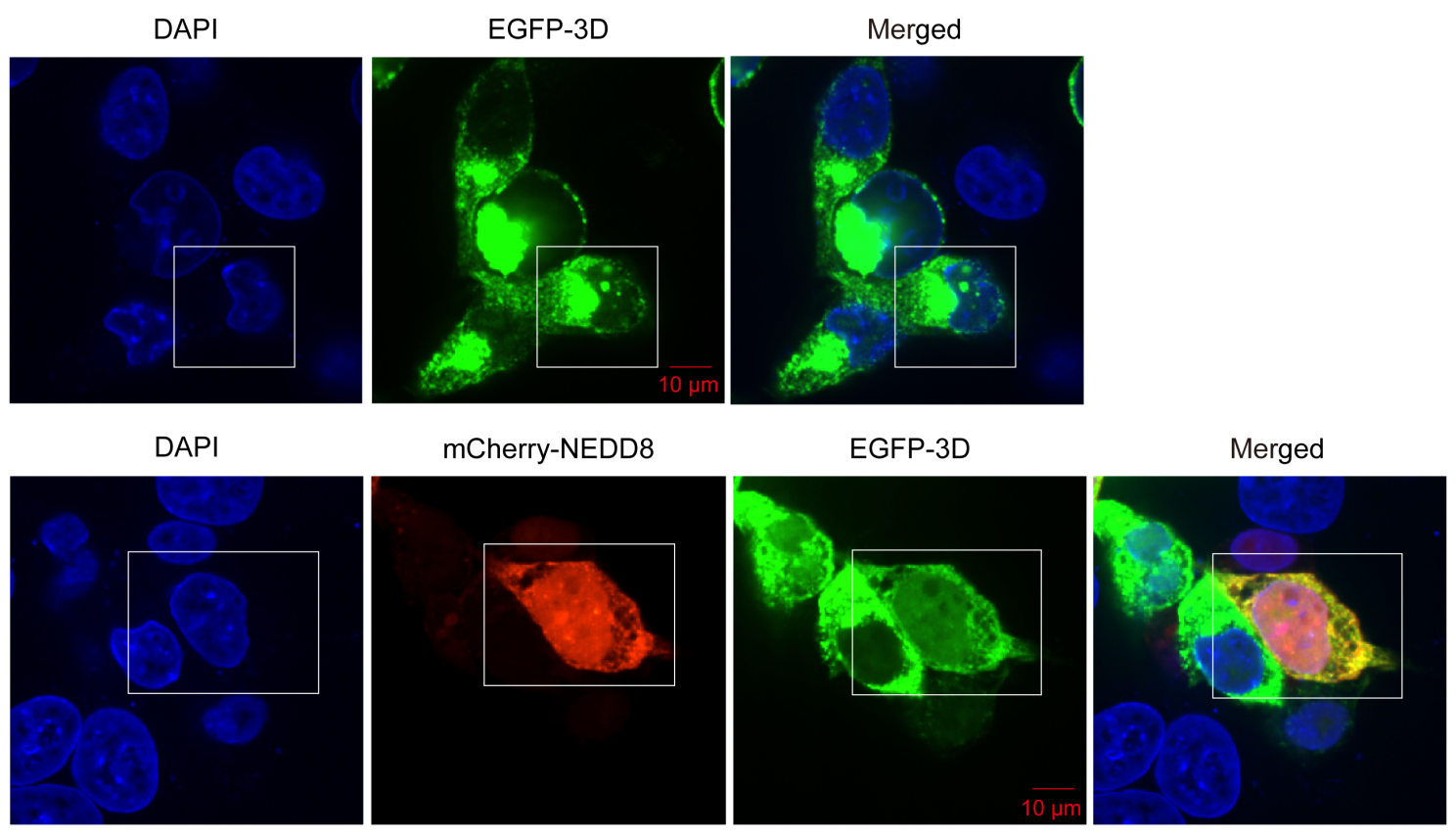
**

**Fig. S3. The neddylation sites of 3D^pol^ in CVB is well conserved.** The amino acid sequence alignment of 3D^pol^ of CVB1~6. The lysine (K) residues at 261 and 457 are highlighted as yellow.

**
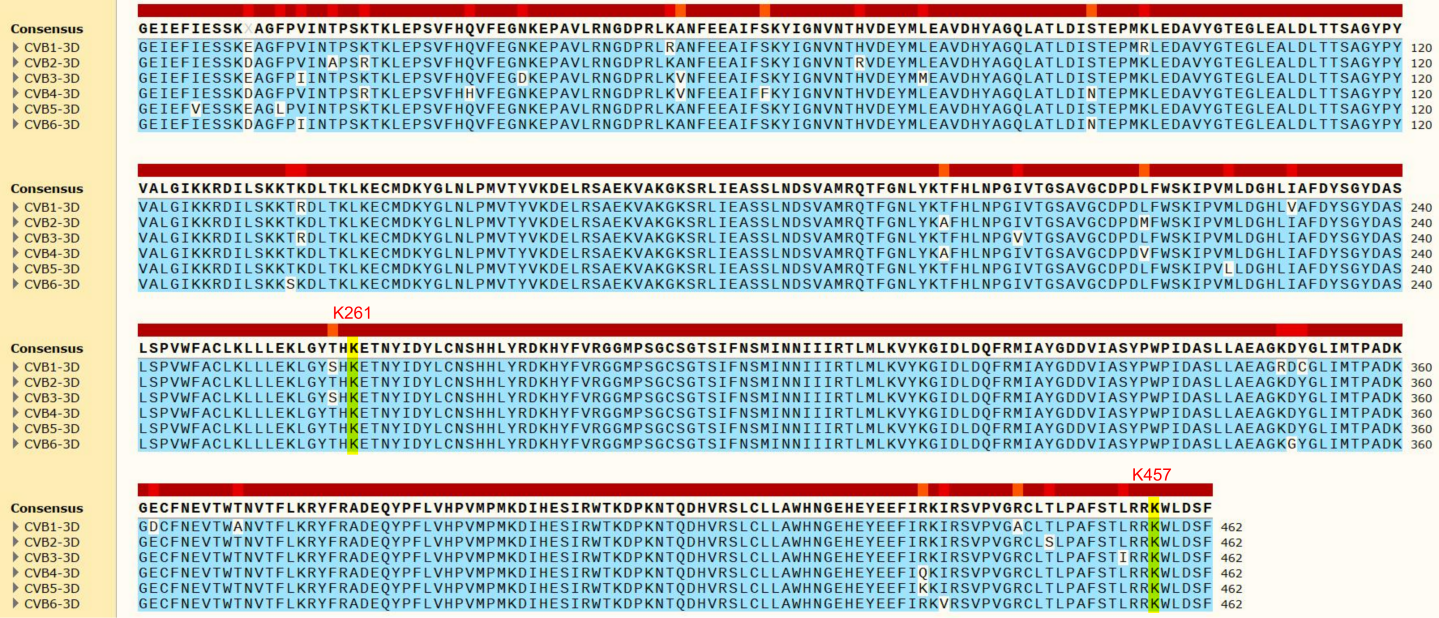
**

**Fig. S4. MLN4924 suppresses CVB3 replication.** (A) HEK293T cells were cultured in 96-well plates for 24 h. Cells were treated with MLN4924 at various concentrations (0.1 μM, 1 μM, 10 μM, 100 μM and 1 mM) for 24 h. Cell viability was measured using MTT assay. Cell viability curves were drawn by Graphpad software (*n* = 8). (B-D) HEK293T cells were infected with CVB3 (MOI = 1) and treated with MLN4924 at the indicated concentration for 24 h. Total proteins were extracted and subjected to immunoblotting (*n* = 3). (E-G) HEK293T cells were infected with wild type or mutant CVB3 (MOI = 1) with or without the treatment of MLN4924 (9 μM) for 24 h. Cells were harvested and subjected to immunoblotting (*n* = 3). Quantitative analysis of the immunoblotting results was carried out by ImageJ. MLN: MLN4924. WT: wild type. hpi: hours post-infection.

**
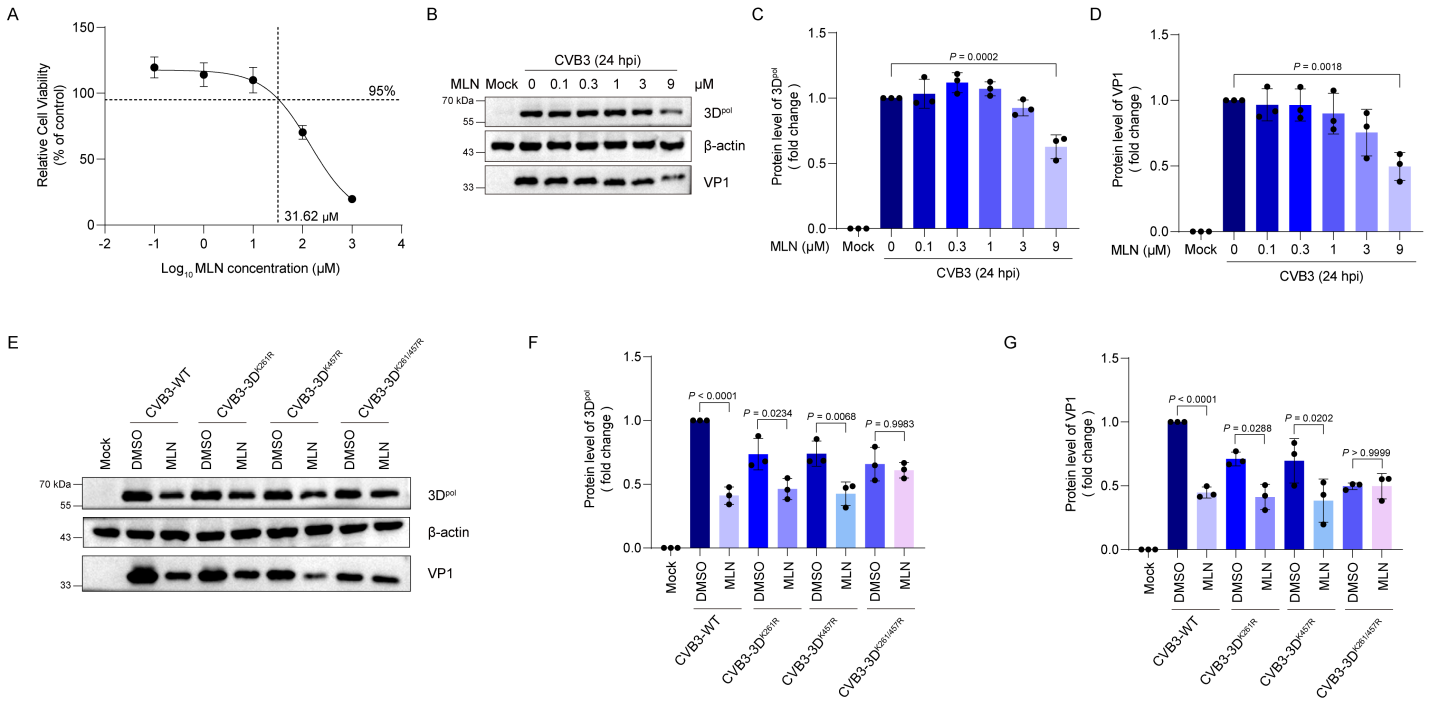
**

**Fig. S5. The alignment of the VP2 amino acid sequences of CVB3 and EV-A71**. The residues at position 69 are highlighted as yellow.


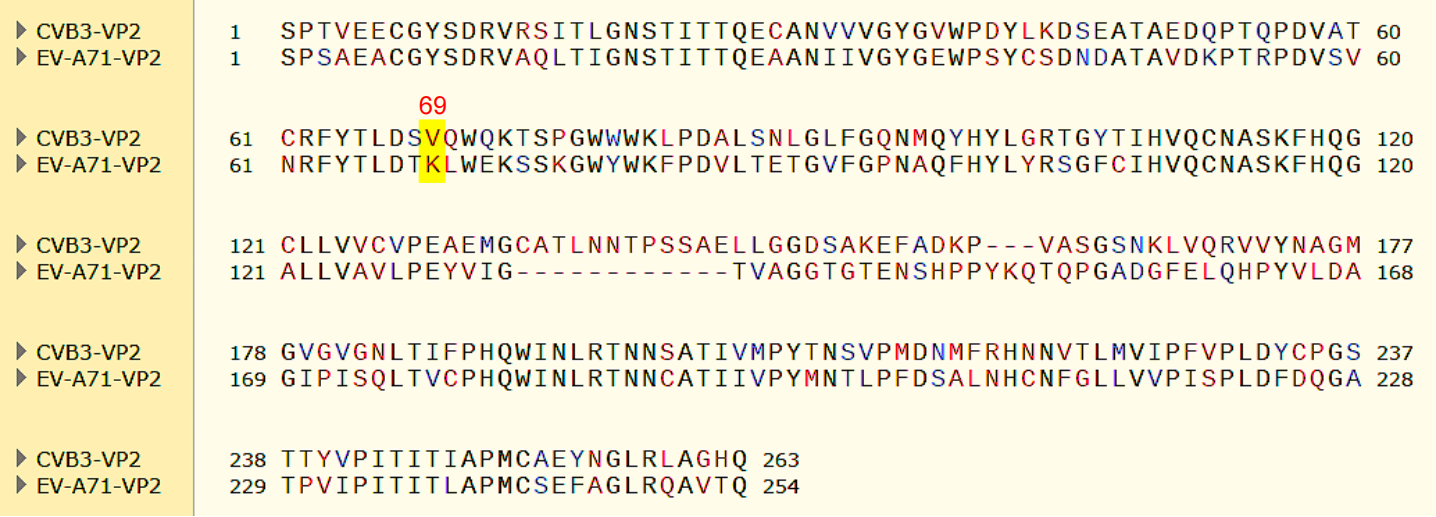

Supplement: Supplemental figures — Figures S1 to S5. [file jvi.01535-25-s0001.docx]
