# Supplementary material for: The neddylation of the RNA-dependent RNA polymerase 3D of Coxsackievirus B3 promotes viral replication
Source: J Virol. 2025 Oct 31;99(11):e01535-25. doi: 10.1128/jvi.01535-25 (PMC12646006; doi:10.1128/jvi.01535-25)
Supplement: Table S1 — Data of mass spectrometry for CVB3-infected cells. [file jvi.01535-25-s0002.pdf]

Table S1. Data of mass spectrometry for CVB3-infected cells. HeLa cells were infected or mock-infected with CVB3 at MOI of 1 for 24 h. Culture medium was aspirated and cells were washed with ice-cold PBS. Cell lysates were prepared with NP40 lysis buffer supplemented with 1% protease inhibitor. Cell lysates were analyzed by nanoscale liquid chromatography coupled to tandem mass spectrometry. Protein abundance which is normalized to GAPDH was provided.

GeneSymbol	Description	NC1_ibaq	NC1.1_ibaq	NC2_ibaq	NC2.1_ibaq	NC3_ibaq	NC3.1_ibaq	CVB1_ibaq	CVB2_ibaq	CVB3_ibaq
1-Mar	MOSC domain-containing protein 1, mitochondrial precursor [Homo sapiens]	347254.0506	214022.0974	343203.3327	534399.3666	308191.9071	1157195.457	1097669.568	292091.3089	1287198.254
10-Sep	septin-10 isoform 1 [Homo sapiens]	690418.3141	1079770.039	1160423.119	1172540.937	1268841.519	1166999.723	1119203.168	994918.523	1100074.771
11-Sep	septin-11 [Homo sapiens]	1179403.839	1377861.924	1828435.775	1200594.392	1968396.662	1317894.891	1278868.973	1235630.141	1672392.557
15-Sep	15 kDa selenoprotein isoform 1 precursor [Homo sapiens]	931203.6272	389987.1617	620271.9957	1021993.408	2035548.943	861866.0048	1060202.741	1410944.56	811162.9333
2-Mar	MOSC domain-containing protein 2, mitochondrial precursor [Homo sapiens]	121677.2525	0	180783.2099	133303.1372	208217.3576	167588.4238	170797.0501	97464.37974	48093.98013
2-Sep	septin-2 [Homo sapiens]	3484361.354	5112033.968	6020792.986	4754098.703	5649924.664	5672962.939	4660253.023	4380777.953	4813677.719
5-Mar	E3 ubiquitin-protein ligase MARCH5 [Homo sapiens]	516588.3677	759818.8645	350531.4027	708914.4255	938615.8065	631982.3774	719396.363	585121.7212	328917.6086
6-Mar	E3 ubiquitin-protein ligase MARCH6 isoform 1 [Homo sapiens]	109485.3105	197691.8571	0	98299.64055	0	116227.3286	144412.4551	79901.74369	148948.1504
6-Sep	septin-6 isoform B [Homo sapiens]	898364.2099	578462.5283	1059774.126	926853.1335	1067932.912	990858.3476	865424.5298	696992.8723	801805.5681
7-Sep	septin-7 isoform 1 [Homo sapiens]	2987234.988	3283180.81	3856919.963	3435290.564	3962203.886	3526258.675	3178485.093	2754152.127	3307756.256
8-Sep	septin-8 isoform a [Homo sapiens]	183353.0013	77495.82412	327670.4562	237658.2864	313336.4402	183154.9711	214703.6935	147693.1254	135759.768
9-Sep	septin-9 isoform a [Homo sapiens]	1710581.755	2056883.244	2369750.385	2229165.221	2531251.75	2238341.568	2226548.162	1852753.439	2073343.506
AAAS	aladin isoform 1 [Homo sapiens]	508197.8786	688107.2335	638032.9379	615979.5601	564237.0366	623249.3696	653309.305	550493.118	836374.446
AACS	acetoacetyl-CoA synthetase [Homo sapiens]	271862.5753	362342.9986	323389.4426	307430.9406	398949.2584	339546.5708	359160.3103	277357.9955	382350.6998
AAGAB	alpha- and gamma-adaptin-binding protein p34 isoform 1 [Homo sapiens]	345094.5833	326614.2679	297601.0139	553067.1806	376693.6825	609707.7738	571476.4286	460711.0565	0
AAK1	AP2-associated protein kinase 1 [Homo sapiens]	62486.44328	26824.38354	70475.44166	80313.71494	71541.50923	65945.53852	62805.20941	58541.64207	37268.76063
AAMP	angio-associated migratory cell protein [Homo sapiens]	249136.8582	422075.9761	541001.012	554305.9813	471543.9417	621851.3409	305275.7174	225514.1212	489564.717
AAR2	protein AAR2 homolog [Homo sapiens]	523271.8565	770905.7631	1009021.117	588941.0886	850740.6874	727596.1535	631719.5065	640590.7336	809630.2079
AARS	alanine--tRNA ligase, cytoplasmic [Homo sapiens]	2341269.51	2405585.881	4159353.168	2392876.249	4511662.613	2991377.949	2725142.08	2010274.279	2508745.907
AARS2	alanine--tRNA ligase, mitochondrial [Homo sapiens]	206919.7892	196218.4104	289286.7087	212053.1027	282024.772	230510.3726	218097.2916	179539.0971	242150.9796
AASDHPPT	L-aminoadipate-semialdehyde dehydrogenase-phosphopantetheinyl transferase	1062383.219	1026950.695	1447450.947	1202356.625	927577.5246	1248392.087	1123237.964	995469.52	898684.7715
AATF	protein AATF [Homo sapiens]	372792.1989	350889.0501	492422.3055	434471.2492	517103.6936	545940.6836	389780.9901	315541.3158	382404.7729
ABCB10	ATP-binding cassette sub-family B member 10, mitochondrial [Homo sapiens]	65018.27687	84308.46567	83770.31676	89255.13616	102522.4591	88560.86004	119249.1118	54307.93081	265880.1841
ABCB6	ATP-binding cassette sub-family B member 6, mitochondrial [Homo sapiens]	201236.6074	216748.5799	210973.3428	266931.6689	177671.6729	239779.7285	244940.5852	167970.7931	152016.9057
ABCB7	ATP-binding cassette sub-family B member 7, mitochondrial isoform 1 [Homo s	225418.847	287621.1915	286926.588	286636.8326	337319.8177	262078.2051	204508.0017	251094.2196	267985.535
ABCC1	multidrug resistance-associated protein 1 [Homo sapiens]	66813.18861	84003.14291	65646.14246	106249.9957	25973.73251	75753.6118	97033.94422	71060.1495	75805.04836
ABCC2	canalicular multispecific organic anion transporter 1 [Homo sapiens]	43579.74554	32320.20883	55854.16071	0	45102.30357	72448.53274	0	0	0
ABCC4	multidrug resistance-associated protein 4 isoform 1 [Homo sapiens]	21807.16099	0	18747.25551	22592.88235	98674.74632	0	0	0	0
ABCD1	ATP-binding cassette sub-family D member 1 [Homo sapiens]	292652.4652	292126.3856	405738.6028	288761.3838	434771.8261	303916.813	304833.5046	246194.4613	342582.4295
ABCD3	ATP-binding cassette sub-family D member 3 isoform a [Homo sapiens]	840189.5894	890481.6394	1148090.114	883975.6803	1445365.039	941727.0535	848658.1525	779271.7842	972320.3701
ABCE1	ATP-binding cassette sub-family E member 1 [Homo sapiens]	2204988.573	2459202.035	2952604.934	2378006.62	3002843.293	2531735.959	2497514.536	2139234.107	2409745.607
ABCF1	ATP-binding cassette sub-family F member 1 isoform a [Homo sapiens]	1592514.474	1830406.123	2098607.436	1886464.368	1997563.313	2016941.606	1962355.018	1591107.042	2050969.072
ABCF2	ATP-binding cassette sub-family F member 2 isoform b [Homo sapiens]	1241826.59	1753403.561	2088322.044	1606571.932	2196802.529	1722449.87	1561875.42	1382096.284	1571153.477
ABCF3	ATP-binding cassette sub-family F member 3 [Homo sapiens]	200954.1171	254701.786	273264.3596	253438.529	308047.5854	257153.0033	260121.6778	249281.5047	233421.4332
ABHD10	mycophenolic acid acyl-glucuronide esterase, mitochondrial isoform 1 precu	1195057.286	2103149.339	1694198.642	2075353.501	1663170.09	2248264.148	2289497.125	1748900.396	2276089.401
ABHD11	alpha/beta hydrolase domain-containing protein 11 isoform 1 [Homo sapiens]	655758.5316	462730.709	833028.0707	697403.5935	791429.7103	753330.0555	628915.2079	186568.6937	349033.0052
ABHD12	monoacylglycerol lipase ABHD12 isoform b [Homo sapiens]	271455.8492	246699.8486	284898.9983	281848.5246	286504.9587	263170.5455	306813.3872	186896.0754	286209.6161
ABHD14B	alpha/beta hydrolase domain-containing protein 14B isoform 1 [Homo sapiens	8280038.527	3886313.019	11147267.22	10189794.46	12386443.5	10926976.25	10016682.56	8807031.044	11176799.46
ABHD16A	abhydrolase domain-containing protein 16A isoform a [Homo sapiens]	149010.2856	201204.9318	203100.3704	226822.3683	158571.9548	212108.2611	220198.6341	167265.5647	196528.646
ABHD5	1-acylglycerol-3-phosphate O-acyltransferase ABHD5 [Homo sapiens]	0	0	0	0	0	0	133162.974	78784.131	53270.091
ABI1	abl interactor 1 isoform a [Homo sapiens]	243762.806	319517.9628	322677.5913	480516.7916	625393.2255	680880.7329	662169.4127	687430.769	259797.6419
ABI2	abl interactor 2 [Homo sapiens]	0	0	0	0	0	0	0	0	0
ABL2	Abelson tyrosine-protein kinase 2 isoform b [Homo sapiens]	90054.87645	0	78272.4652	0	84427.11949	95609.85383	59062.61717	0	90654.47362
ABLIM1	actin-binding LIM protein 1 isoform a [Homo sapiens]	36073.18841	0	63540.73081	61756.44047	15922.9883	0	32547.10765	32113.7476	35598.93366
ABR	active breakpoint cluster region-related protein isoform a [Homo sapiens]	30273.96948	0	35167.09157	50691.50145	46372.54762	47424.7173	15125.01753	0	58170.56395
ABRACL	costars family protein ABRACL [Homo sapiens]	10505827.1	5766129.403	11784720.53	5885965.023	7002537.558	7258196.494	10023175.16	10429847.42	5447440.081
ABT1	activator of basal transcription 1 [Homo sapiens]	136167.2527	0	339172.5692	0	243036.3789	338955.2019	192238.1408	226550.7688	203264.6549
ACAA1	3-ketoacyl-CoA thiolase, peroxisomal isoform a [Homo sapiens]	770349.6859	526168.4595	769752.8746	677527.0656	750088.212	752962.4972	728726.2725	683263.1394	816210.9268
ACAA2	3-ketoacyl-CoA thiolase, mitochondrial [Homo sapiens]	2385974.69	2434965.208	3837933.223	3581012.226	4241253.134	3707173.786	3211951.424	2510245.745	3259286.912
ACACA	acetyl-CoA carboxylase 1 isoform 1 [Homo sapiens]	227307.6315	225665.0118	308303.2983	238106.9548	302497.9937	231085.8216	232348.6062	184862.6415	0
ACAD10	acyl-CoA dehydrogenase family member 10 isoform a [Homo sapiens]	8521.056471	0	15022.57647	9208.645882	16402.8625	13474.01647	8899.327647	0	10519.05441
ACAD11	acyl-CoA dehydrogenase family member 11 [Homo sapiens]	0	0	0	0	0	0	54726.46636	36529.46138	0
ACAD8	isobutyryl-CoA dehydrogenase, mitochondrial [Homo sapiens]	89395.35895	0	114601.3864	95609.26974	0	137394.7615	130692.0843	65135.78125	0
ACAD9	acyl-CoA dehydrogenase family member 9, mitochondrial [Homo sapiens]	620092.6969	613177.3146	794457.1134	612277.9913	801167.861	643754.5252	643889.0028	562200.3213	653158.5012
ACADM	medium-chain specific acyl-CoA dehydrogenase, mitochondrial isoform b precu	1355280.185	1437065.598	1669831.072	1430427.714	1623997.656	1483116.295	1629151.663	1291520.128	1496108.168
ACADS	short-chain specific acyl-CoA dehydrogenase, mitochondrial precursor [Homo	254336.0603	210732.1812	279821.9345	211232.1026	365958.1297	198939.1386	249532.4241	182596.0065	327775.7617
ACADSB	short/branched chain specific acyl-CoA dehydrogenase, mitochondrial precurs	81663.62752	182956.2846	151368.6239	101218.9161	203494.5848	202543.7207	110198.62	86030.19718	96514.07785
ACADVL	very long-chain specific acyl-CoA dehydrogenase, mitochondrial isoform 3 [	2679862.571	3114948.702	3520380.02	3083485.754	3633242.918	3347603.834	3283167.602	2637544.957	3063860.601
ACAP2	arf-GAP with coiled-coil, ANK repeat and PH domain-containing protein 2 [H	162342.1502	125595.3167	197894.0513	136775.1453	155899.2498	190386.9954	154939.2863	135991.6872	143687.1549
ACAT1	acetyl-CoA acetyltransferase, mitochondrial precursor [Homo sapiens]	5251746.959	6288320.127	6504778.828	6352956.683	7090627.102	6563481.146	6965692.841	5294186.176	6368557.196
ACAT2	acetyl-CoA acetyltransferase, cytosolic [Homo sapiens]	2560457.623	2957877.885	3645792.036	3077370.428	3622106.574	3307879.535	3068112.339	2732787.519	3130299.734
ACBD3	Golgi resident protein GCP60 [Homo sapiens]	718569.527	714557.3	900474.2689	781255.4724	901113.9184	775153.6536	762044.9541	649939.1842	902479.2816
ACBD5	acyl-CoA-binding domain-containing protein 5 isoform 1 [Homo sapiens]	304281.0766	265428.4294	471110.0338	317649.9035	467987.0674	436751.1802	313958.2669	366725.1504	391598.051
ACBD6	acyl-CoA-binding domain-containing protein 6 [Homo sapiens]	63115.38438	388709.7671	310749.7758	652156.6138	367378.0108	852367.97	0	959468.44	197652.8613
ACD	adrenocortical dysplasia protein homolog isoform 1 [Homo sapiens]	0	127903.1313	109442.4234	98721.09219	102268.1631	0	0	0	0
ACIN1	apoptotic chromatin condensation inducer in the nucleus isoform 1 [Homo sa	332485.2951	470345.9131	542746.8383	440533.4216	116960.3669	465525.4457	471163.0157	139778.9367	526031.9687
ACLY	ATP-citrate synthase isoform 1 [Homo sapiens]	2724717.714	2825599.951	3792807.479	3031773.748	4024349.77	3214567.684	2941275.24	2445232.068	3039894.565
ACO1	cytoplasmic aconitate hydratase [Homo sapiens]	596019.3722	663168.8526	637862.507	719555.945	876236.0096	719032.7715	682583.0732	395425.0079	584989.7755
ACO2	aconitate hydratase, mitochondrial precursor [Homo sapiens]	903264.9142	1036611.313	913156.2899	821850.3875	1010941.338	961153.3448	996692.8878	725459.2611	1039725.949
ACOT1	acyl-coenzyme A thioesterase 1 [Homo sapiens]	0	0	0	0	0	0	1233637.806	0	796751.2752
ACOT13	acyl-coenzyme A thioesterase 13 isoform 1 [Homo sapiens]	6386714.704	6744323.423	7190319.394	6760346.358	6811263.325	9638626.038	7820975.723	6150664.891	7880188.094
ACOT2	acyl-coenzyme A thioesterase 2, mitochondrial [Homo sapiens]	0	0	0	0	0	0	0	0	0
ACOT7	cytosolic acyl coenzyme A thioester hydrolase isoform hBACHb [Homo sapiens	1086253.687	1029548.096	1421364.94	1312003.398	1143258.854	1407677.953	1043875.843	927179.156	973684.2321
ACOT8	acyl-coenzyme A thioesterase 8 [Homo sapiens]	257262.5326	90292.214	263573.9517	0	252198.5937	66697.88084	304657.2102	0	42285.79864
ACOT9	acyl-coenzyme A thioesterase 9, mitochondrial isoform a precursor [Homo sa	1137535.507	1379506.245	1419611.701	1107312.364	1412172.03	1146649.677	1198477.448	1214799.807	1261333.279
ACOX1	peroxisomal acyl-coenzyme A oxidase 1 isoform a [Homo sapiens]	885289.0432	126227.1144	1297075.508	249694.1049	494231.5	1486400.209	0	934776.0047	1007299.554
ACP1	low molecular weight phosphotyrosine protein phosphatase isoform c [Homo sa	3005413.278	4229857.061	4480114.981	3057422.03	2951326.657	4741347.027	3678322.782	3396310.31	4126804.969
ACP1	low molecular weight phosphotyrosine protein phosphatase isoform b [Homo sa	3005413.278	4229857.061	4480114.981	3057422.03	2951326.657	4741347.027	3678322.782	3396310.31	4126804.969
ACP2	lysosomal acid phosphatase isoform 1 precursor [Homo sapiens]	310018.5522	326628.8355	112521.9142	256629.0346	212916.0728	354235.8512	349964.681	210211.2781	293914.8004
ACP6	lysophosphatidic acid phosphatase type 6 precursor [Homo sapiens]	145731.8488	128244.0382	117051.102	159847.9913	173985.6608	129840.0356	197703.2045	213607.5878	153711.197
ACSF3	acyl-CoA synthetase family member 3, mitochondrial precursor [Homo sapiens	136126.6225	103701.9687	153178.7747	174846.099	254048.1816	179919.9955	319343.5404	230796.9183	211891.4221
ACSL1	long-chain-fatty-acid--CoA ligase 1 [Homo sapiens]	80652.87643	53364.14805	95055.62688	135219.9295	114187.8609	94488.85105	66514.91759	67845.50425	95908.42199
ACSL3	long-chain-fatty-acid--CoA ligase 3 [Homo sapiens]	699615.1239	688705.4731	854297.4561	747294.2936	1047392.404	694996.4848	816094.8333	699947.4973	852706.1439
ACSL4	long-chain-fatty-acid--CoA ligase 4 isoform 2 [Homo sapiens]	780929.1087	944092.4431	1043637.198	1018772.25	1069733.151	1015013.667	1062665.491	922232.6696	1039028.462
ACSS2	acetyl-coenzyme A synthetase, cytoplasmic isoform 2 [Homo sapiens]	65717.94138	35997.48959	43832.2876	34323.41743	39549.14914	34993.35467	45341.0498	0	71091.88008
ACTA1	actin, alpha skeletal muscle [Homo sapiens]	107662475.8	89578804.16	0	106548637.7	25230093.33	114167695.5	140379502.5	116448928.4	136082139.3
ACTB	actin, cytoplasmic 1 [Homo sapiens]	136391804.9	165040417.5	157487604.6	153149417.7	180024355.1	176552348	158329039.2	144887137.9	168845405.6
ACTBL2	beta-actin-like protein 2 [Homo sapiens]	840126.9368	967054.9474	891904.8609	820108.106	60250961.92	659875.0973	1456931.365	1083556.625	125162.6297
ACTG1	actin, cytoplasmic 2 [Homo sapiens]	32567342.98	41372827.17	41223565.09	38939450.12	42594475.08	43643530.05	41786792.07	34340107.63	38362394.06
ACTL6A	actin-like protein 6A isoform 1 [Homo sapiens]	1775072.485	2095352.204	2674724.734	2298485.452	2702863.024	2458974.85	2135819.136	1880203.556	2128107.918
ACTN1	alpha-actinin-1 isoform b [Homo sapiens]	2503489.807	2524112.439	3233203.081	2677636.81	3427347.936	2819568.656	2773589.036	2436668.061	2767379.156
ACTN4	alpha-actinin-4 [Homo sapiens]	5911149.624	5766248.422	8173220.181	6116259.141	7857415.976	6450455.681	6376081.105	6405563.73	7845540.008
ACTR10	actin-related protein 10 [Homo sapiens]	243457.6943	194890.0938	409889.3476	290023.9532	391302.5164	488778.5745	417804.707	354932.1222	354312.2061
ACTR1A	alpha-centractin [Homo sapiens]	2532015.461	3281174.476	3960087.835	3408371.739	4126331.946	3869739.748	3564372.605	2468697.639	3118867.739
ACTR1B	beta-centractin [Homo sapiens]	1472948.315	1626286.32	1704291.303	2043111.593	1735167.918	1496404.082	1359039.352	1017062.076	1758518.897
ACTR2	actin-related protein 2 isoform b [Homo sapiens]	4490352.81	5330889.876	5975322.368	5367728.164	6214666.013	5354328.747	5424859.918	4270661.056	5462838.692
ACTR3	actin-related protein 3 isoform 1 [Homo sapiens]	4234966.06	4637229.384	5521468.038	4707544.238	4909933.593	4842673.304	4651541.035	4352646.633	5285287.891
ACTR5	actin-related protein 5 [Homo sapiens]	27409.71305	0	58700.92059	28509.51755	57611.12788	81330.03419	0	0	0
ACTR8	actin-related protein 8 [Homo sapiens]	0	0	0	0	0	0	36444.19062	0	73372.108
ACY1	aminoacylase-1 isoform a [Homo sapiens]	994864.8908	1006829.187	1567312.715	1105088.545	1301956.353	1154216.522	1040607.084	818045.6624	900469.734
ACYP1	acylphosphatase-1 isoform a [Homo sapiens]	1571505.361	1533913.711	896662.2083	1131076.627	3043925.058	1538544.62	1319780.422	1155816.338	1616966.719
ACYP2	acylphosphatase-2 [Homo sapiens]	0	0	0	0	0	0	1282045.615	0	1167633.457
ADA	adenosine deaminase [Homo sapiens]	272107.3841	329705.1633	532053.0394	207366.1599	442270.591	295640.8427	265416.182	204194.1605	333289.0652
ADAM10	disintegrin and metalloproteinase domain-containing protein 10 precursor [	94613.77519	110653.0925	324481.9887	107866.1166	123306.8524	129893.8424	75571.23096	41141.32679	119218.2489
ADAM15	disintegrin and metalloproteinase domain-containing protein 15 isoform 6 pr	222510.94	235408.6263	392989.5268	68948.02315	341515.4501	253727.9066	149263.1493	169060.5435	19321924.3
ADAM17	disintegrin and metalloproteinase domain-containing protein 17 preproprote	0	0	0	0	0	0	0	41288.46269	24073.87052
ADAM9	disintegrin and metalloproteinase domain-containing protein 9 precursor [H	148114.4825	192945.1346	172067.7998	172708.9713	175229.5496	175896.0736	183011.7181	90718.84977	154400.0853
ADAR	double-stranded RNA-specific adenosine deaminase isoform a [Homo sapiens]	242001.5208	239524.7138	424660.4867	248671.0978	343694.5188	375327.0961	462390.5885	227943.4165	411603.0201
ADAR	double-stranded RNA-specific adenosine deaminase isoform b [Homo sapiens]	242001.5208	239524.7138	424660.4867	248671.0978	343694.5188	375327.0961	462390.5885	227943.4165	411603.0201
ADARB1	double-stranded RNA-specific editase 1 isoform 2 [Homo sapiens]	35733.54143	62851.88163	74750.24432	47343.26705	47121.63981	12887.76409	0	0	0
ADCK4	uncharacterized aarF domain-containing protein kinase 4 isoform a [Homo sa	59899.27648	131235.235	100641.9471	5944.534597	75431.92378	108605.4899	33849.07222	42635.82541	128530.5612
ADD1	alpha-adducin isoform b [Homo sapiens]	165537.3872	194993.5417	224656.5946	173628.8172	225026.793	202702.7578	167338.3787	104423.6065	109901.5921
ADD3	gamma-adducin isoform a [Homo sapiens]	151982.4889	0	124796.4991	119786.3447	300481.4119	60575.16664	0	53299.45927	82539.67814
ADH5	alcohol dehydrogenase class-3 [Homo sapiens]	1113893.474	1843203.479	2640302.646	2086814.361	2099733.761	1776090.357	2392509.642	1489116.518	1961915.364
ADI1	1,2-dihydroxy-3-keto-5-methylthiopentene dioxygenase [Homo sapiens]	2932382.103	2485370.468	3332528.826	2772373.336	3586783.431	3012487.52	2666640.109	2694079.913	3175185.942
ADK	adenosine kinase isoform b [Homo sapiens]	3287804.323	3152849.629	4585966.422	4768097.011	4314567.239	4885926.247	4814061.965	2371565.361	2989970.207
ADNP	activity-dependent neuroprotector homeobox protein [Homo sapiens]	102153.1438	147729.217	205873.8477	159107.7427	209279.796	135071.2685	157176.611	110815.5954	120043.0236
ADPGK	ADP-dependent glucokinase precursor [Homo sapiens]	717232.2473	637706.6439	739257.627	527660.933	986857.2672	766787.8572	749783.1516	562098.862	916637.291
ADPRHL2	poly(ADP-ribose) glycohydrolase ARH3 [Homo sapiens]	493416.9709	511192.9438	635878.7064	554458.717	750893.851	552730.4491	472739.8141	475161.1054	300196.8972

ADRBK1	beta-adrenergic receptor kinase 1 [Homo sapiens]	78373.67	97265.79167	0	0	59419.52127	85632.14423	0	55103.02503	64180.55543
ADRM1	proteasomal ubiquitin receptor ADRM1 precursor [Homo sapiens]	3138527.597	3791515.672	3605141.587	3406442.364	4125934.713	3290375.24	3349895.473	2217463.223	2708980.345
ADSL	adenylosuccinate lyase isoform a [Homo sapiens]	1982192.854	1728830.746	2604699.043	1690202.244	2647586.217	2030294.691	1960508.351	1413378.094	1537754.401
ADSS	adenylosuccinate synthetase isozyme 2 [Homo sapiens]	3095965.974	3690232.892	3734994.219	3466315.868	4157127.809	3430655.271	3495604.175	2928821.978	3990207.65
AEBP2	zinc finger protein AEBP2 isoform b [Homo sapiens]	12983.70104	9596.846338	43948.88396	26411.84992	48293.85828	0	0	0	0
AFAP1	actin filament-associated protein 1 isoform A [Homo sapiens]	37392.99767	26493.59375	65453.04276	33375.73707	49568.42362	0	0	0	0
AFF4	AF4/FMR2 family member 4 [Homo sapiens]	22582.03468	15561.82612	11837.07176	19510.56698	0	17936.5805	24025.65281	0	19025.6303
AFG3L2	AFG3-like protein 2 [Homo sapiens]	577885.8462	558827.3519	610489.5052	567703.662	553198.5943	594401.3101	588360.8415	523107.7809	623371.6689
AGA	N(4)-(beta-N-acetylglucosaminyl)-L-asparaginase isoform 1 preproprotein [Ho	136219.2477	450462.028	790063.0467	345839.2052	0	338752.7037	396702.7504	225970.4555	394905.2418
AGAP3	arf-GAP with GTPase, ANK repeat and PH domain-containing protein 3 isoform	32362.57464	51883.6744	0	61526.29381	31120.69201	0	26750.31229	17205.37543	13061.11856
AGFG1	arf-GAP domain and FG repeat-containing protein 1 isoform 1 [Homo sapiens]	377659.5327	477537.8272	812624.2031	646330.5209	2246584.174	1478289.996	339154.4551	391526.4004	467874.1312
AGFG2	arf-GAP domain and FG repeat-containing protein 2 [Homo sapiens]	252579.2705	482640.1045	572297.3293	473984.2705	522114.2545	450059.8886	379637.3955	221591.6103	615090.8159
AGK	acylglycerol kinase, mitochondrial precursor [Homo sapiens]	595680.3603	520826.6702	794163.0552	742879.4374	851120.3945	899683.0209	735612.9227	587784.0119	385614.8558
AGL	glycogen debranching enzyme isoform 1 [Homo sapiens]	168212.4132	131844.7082	165175.0982	136426.0465	234395.4129	127598.8386	116077.3073	104041.6069	138129.5293
AGMAT	agmatinase, mitochondrial precursor [Homo sapiens]	1320036.861	1438540.307	1863479.708	1255687.229	1928163.733	1509301.349	1560835.432	1424073.006	1600262.742
AGPAT1	1-acyl-sn-glycerol-3-phosphate acyltransferase alpha [Homo sapiens]	480831.9658	532222.4078	809065.0806	443966.8981	548659.2342	666958.665	512730.4505	398157.8794	490219.0642
AGPAT2	1-acyl-sn-glycerol-3-phosphate acyltransferase beta isoform a precursor [Ho	0	0	0	0	0	0	97600.95263	0	235180.304
AGPAT3	1-acyl-sn-glycerol-3-phosphate acyltransferase gamma [Homo sapiens]	0	70474.67347	90550.16327	0	75472.48299	41384.94048	0	0	0
AGPAT5	1-acyl-sn-glycerol-3-phosphate acyltransferase epsilon [Homo sapiens]	82664.38667	113651.5487	72082.19667	95019.05167	115149.4909	147062.5279	118155.395	102767.0013	62178.55979
AGPAT6	glycerol-3-phosphate acyltransferase 6 [Homo sapiens]	0	0	0	0	0	0	129360.2853	97367.19908	0
AGPS	alkyldihydroxyacetonephosphate synthase, peroxisomal precursor [Homo sapien	1264726.239	1601833.889	1670934.407	1230778.244	1744042.87	1685588.189	1724578.82	1465815.137	1697111.118
AGRN	agrin precursor [Homo sapiens]	24303.38914	20219.90105	41988.40052	17466.23406	26431.93898	13656.78137	16045.8371	0	47998.33184
AGTRAP	type-1 angiotensin II receptor-associated protein isoform a [Homo sapiens]	1793206.667	2927344.179	2613489.846	3113006.436	3086488.61	3538369.269	2921999.897	1640244.367	2902047.885
AHCTF1	protein ELYS [Homo sapiens]	90203.64016	75974.38065	108765.0994	98895.88593	144803.8087	90458.04161	96486.82697	73599.68681	103364.1913
AHCY	adenosylhomocysteinase isoform 1 [Homo sapiens]	11329306.74	10334218.94	15190840.06	11473651.36	14626635.64	16754173.46	14883491.61	9620558.591	12717889.9
AHCYL1	putative adenosylhomocysteinase 2 isoform a [Homo sapiens]	1664868.03	1706523.716	2171245.133	1773086.189	2231614.294	1744158.542	847958.0819	1524087.317	1761545.941
AHNAK	neuroblast differentiation-associated protein AHNAK isoform 1 [Homo sapiens	789515.2507	973710.274	989050.9981	893842.5828	1005351.487	970242.6553	867018.2251	823242.3219	904989.7507
AHNAK2	protein AHNAK2 [Homo sapiens]	8898.344462	0	2567.701335	17354.15252	3446.528194	1799.819146	12823.58249	12559.49973	0
AHSA1	activator of 90 kDa heat shock protein ATPase homolog 1 [Homo sapiens]	6056518.732	7142210.69	8458931.739	7445755.93	8309595.746	7418749.521	7467585.641	5111406.361	7448308.113
AIDA	axin interactor, dorsalization-associated protein [Homo sapiens]	26640.76296	145218.8111	259724.0899	206799.3741	72359.13056	206992.215	269658.7745	199531.0019	51759.15031
AIF1L	allograft inflammatory factor 1-like isoform 3 [Homo sapiens]	597145.3992	478494.4484	689778.8306	648895.7952	835257.4729	615721.6295	295480.5941	207229.5602	956435.674
AIFM1	apoptosis-inducing factor 1, mitochondrial isoform 1 precursor [Homo sapien	2036758.859	2569635.9	2664944.336	2480802.331	2818661.161	2716320.752	2651984.196	1908200.198	2302328.196
AIFM2	apoptosis-inducing factor 2 [Homo sapiens]	574023.2431	693113.7966	529932.8486	652688.9104	757773.9104	679728.3931	683833.7099	498831.2388	690704.5628
AIM1	absent in melanoma 1 protein [Homo sapiens]	8767.888038	4348.583402	0	5531.29553	0	8433.235513	0	0	0
AIMP1	aminoacyl tRNA synthase complex-interacting multifunctional protein 1 isofo	2903606.514	3460324.62	2267982.522	2607187.001	3872773.83	3446172.497	3174013.829	2072476.594	3491280.875
AIMP2	aminoacyl tRNA synthase complex-interacting multifunctional protein 2 [Homo	5500366.719	6819830.734	7651394.91	6230934.623	7216372.821	6545863.541	6406517.11	5175308.596	6692853.259
AIP	AH receptor-interacting protein [Homo sapiens]	1250364.545	1881293.377	1534504.439	1042721.694	1796865.287	1201025.7	1001518.653	1114819.099	1302348.337
AK1	adenylate kinase isoenzyme 1 [Homo sapiens]	7423755.74	8368453.99	9770075.342	8310373.18	9559580.573	9634906.961	8545512.766	7423366.278	8404564.286
AK2	adenylate kinase 2, mitochondrial isoform a [Homo sapiens]	9070458.654	8349746.165	11063066.65	9367641.964	12036572.26	9412970.455	9434828.709	6205887.539	8943313.676
AK3	GTP:AMP phosphotransferase AK3, mitochondrial isoform a [Homo sapiens]	1041196.434	1264935.6	1362132.888	1329592.802	1277803.908	1208026.419	1261857.691	915382.804	1279272.461
AKAP1	A-kinase anchor protein 1, mitochondrial precursor [Homo sapiens]	185537.4068	346999.179	310062.4033	274992.0758	208598.5683	246206.1588	235785.2192	189257.4073	231163.463
AKAP12	A-kinase anchor protein 12 isoform 1 [Homo sapiens]	0	0	0	0	0	0	0	0	0
AKAP13	A-kinase anchor protein 13 isoform 1 [Homo sapiens]	15457.53787	36812.87099	26885.61107	26293.02684	46667.97687	36918.07421	35397.80006	220329.1629	32971.62581
AKAP17A	A-kinase anchor protein 17A isoform 1 [Homo sapiens]	0	0	0	0	0	0	23063.6974	28711.27079	35400.34832
AKAP8	A-kinase anchor protein 8 [Homo sapiens]	916306.7534	669853.1618	856162.5663	223013.9661	918492.8735	140571.4528	102682.2944	206618.1024	151604.4195
AKAP8L	A-kinase anchor protein 8-like [Homo sapiens]	238469.2764	236831.7916	360107.8565	322508.0978	384123.7753	385895.7709	251186.62	245808.1533	230423.2436
AKAP9	A-kinase anchor protein 9 isoform 2 [Homo sapiens]	2152.431893	20085.91237	3606.246845	7534.922428	4246.460195	4589.800741	1699.379063	3966.333942	6429.410223
AKR1A1	alcohol dehydrogenase [NADP(+)] [Homo sapiens]	17701835.92	17098118.61	21316488.88	19517108.43	21857570.03	19591516.19	19645031.22	16989926.55	20078427.32
AKR1B1	aldose reductase [Homo sapiens]	35375199.15	36976656.47	44081220.45	39946904.71	44317968.46	40488636.75	40979302.63	32463707.01	43506888.86
AKR1C1	aldo-keto reductase family 1 member C1 [Homo sapiens]	0	287120.1181	200986.148	0	207222.5104	80991.57813	138043.842	397490.2951	109361.1465
AKR1C3	aldo-keto reductase family 1 member C3 isoform 1 [Homo sapiens]	2650750.176	3330641.05	3416020.765	2611337.943	3842836.979	3342613.419	2622113.692	1964624.999	2618493.652
AKR7A2	aflatoxin B1 aldehyde reductase member 2 [Homo sapiens]	1953118.585	1895449.743	2065707.43	2141537.309	2435736.172	1803962.615	2109755.083	1678249.631	1887939.801
AKT1	RAC-alpha serine/threonine-protein kinase [Homo sapiens]	128390.533	91955.31164	868229.1863	0	828145.5858	487588.2922	0	0	0
AKT1S1	proline-rich AKT1 substrate 1 isoform b [Homo sapiens]	2017617.095	1508987.775	2580308.504	1746788.325	2671271.89	2017962.556	2181305.913	1545302.169	1552745.952
AKT2	RAC-beta serine/threonine-protein kinase isoform 1 [Homo sapiens]	283527.079	229431.5979	319615.8926	184418.1011	270796.6943	200740.6248	198508.2157	202135.3873	219135.1131
ALAD	delta-aminolevulinic acid dehydratase [Homo sapiens]	0	0	0	0	0	0	0	154408.3244	150679.53
ALB	serum albumin preproprotein [Homo sapiens]	4382191.601	926276.7332	1818124.966	693095.3472	1249176.505	907403.172	1266031.152	1044694.456	1430687.333
ALCAM	CD166 antigen isoform 1 precursor [Homo sapiens]	0	551646.9954	738142.8147	524358.9825	540274.2862	571575.2065	778092.8364	626532.7189	845863.8664
ALDH16A1	aldehyde dehydrogenase family 16 member A1 isoform 1 [Homo sapiens]	506641.8613	598595.2885	589669.6106	602279.0038	645433.3243	1566596.42	398672.3363	1631965.77	608528.0502
ALDH18A1	delta-1-pyrroline-5-carboxylate synthase isoform 1 [Homo sapiens]	1879016.805	2105433.784	2557118.677	2111111.918	2539316.731	2266328.32	2347767.779	1894363.12	2243777.964
ALDH1B1	aldehyde dehydrogenase X, mitochondrial precursor [Homo sapiens]	473164.6431	662495.2379	720792.3894	717809.3525	985612.6621	724351.1768	784198.2798	640238.1682	763023.9525
ALDH1L2	mitochondrial 10-formyltetrahydrofolate dehydrogenase precursor [Homo sapie	58467.65222	37384.4574	63735.47408	38838.26047	56935.11164	54487.97425	48697.99816	35101.29332	41143.87296
ALDH2	aldehyde dehydrogenase, mitochondrial isoform 1 precursor [Homo sapiens]	1488530.927	1543459.532	2038987.996	1735838.08	1975524.099	1860537.277	1864129.949	1657875.034	1603371.715
ALDH3A2	fatty aldehyde dehydrogenase isoform 1 [Homo sapiens]	699782.4	1087929.598	929293.9704	1011888.593	1054666.605	1066617.944	749310.8267	976102.8859	991624.7961
ALDH3B1	aldehyde dehydrogenase family 3 member B1 isoform a [Homo sapiens]	0	64970.97239	0	127048.6016	128201.7423	194883.8083	0	0	0
ALDH4A1	delta-1-pyrroline-5-carboxylate dehydrogenase, mitochondrial isoform a prec	35235.33687	112750.77	20393.1115	0	35336.13307	0	41915.11737	0	83176.64202
ALDH5A1	succinate-semialdehyde dehydrogenase, mitochondrial isoform 1 precursor [Ho	220052.8409	334016.7479	350679.5819	212501.7138	347026.6181	280951.2563	281300.2186	183934.1532	451563.063
ALDH7A1	alpha-aminoadipic semialdehyde dehydrogenase isoform 2 [Homo sapiens]	1816859.432	2334036.479	2323753.664	2234043.4	2511155.593	2359733.908	2501867.723	1937825.723	2632302.908
ALDH9A1	4-trimethylaminobutyraldehyde dehydrogenase [Homo sapiens]	2159811.769	2375822.805	2542000.273	2453824.814	2621539.122	2690322.836	2552170.297	2152045.068	2329811.171
ALDOA	fructose-bisphosphate aldolase A isoform 1 [Homo sapiens]	74808212.19	67432884.67	84131350.35	75080748.35	90710108.36	72475571.96	67297998.98	62915299.11	78915224.77
ALDOC	fructose-bisphosphate aldolase C [Homo sapiens]	6395715.766	7350603.783	9447900.643	7475895.028	9838103.371	8184869.531	8363469.797	6995923.427	9152696.119
ALG1	chitobiosyldiphosphodolichol beta-mannosyltransferase [Homo sapiens]	399933.8586	406930.9753	524175.2937	441490.331	603839.6109	436240.0615	542780.9552	373296.1747	459320.5085
ALG10	dol-P-Glc:Glc(2)Man(9)GlcNAc(2)-PP-Dol alpha-1,2-glucosyltransferase [Homo	0	0	0	0	0	0	0	0	0
ALG11	GDP-Man:Man(3)GlcNAc(2)-PP-Dol alpha-1,2-mannosyltransferase [Homo sapiens]	0	163356.2468	45025.86218	0	84635.60897	173200.633	0	0	0
ALG13	UDP-N-acetylglucosamine transferase subunit ALG13 homolog isoform 1 [Homo s	238123.0512	0	148022.2556	223620.481	0	213093.429	245405.4218	206128.027	0
ALG13	UDP-N-acetylglucosamine transferase subunit ALG13 homolog isoform 2 [Homo s	238123.0512	0	148022.2556	223620.481	0	213093.429	245405.4218	206128.027	0
ALG14	UDP-N-acetylglucosamine transferase subunit ALG14 homolog [Homo sapiens]	46348.0487	73532.4513	122114.5503	0	0	98945.39286	100797.6356	62162.27597	99817.7013
ALG2	alpha-1,3/1,6-mannosyltransferase ALG2 [Homo sapiens]	297133.3137	69799.85948	246650.3824	248469	226024.5212	310644.3431	306875.7925	219346.6512	206992.6593
ALG3	dol-P-Man:Man(5)GlcNAc(2)-PP-Dol alpha-1,3-mannosyltransferase isoform a [H	0	435539.7686	0	298409.6855	71216.29933	524246.8678	445160.2924	0	448206.1302
ALG5	dolichyl-phosphate beta-glucosyltransferase isoform 1 [Homo sapiens]	716353.4055	948212.6966	1026625.466	711693.5041	960507.0193	678190.827	817303.5303	865446.6448	1242239.329
ALG6	dolichyl pyrophosphate Man9GlcNAc2 alpha-1,3-glucosyltransferase precursor	0	0	0	0	0	0	136887.3794	98881.20105	0
ALG8	probable dolichyl pyrophosphate Glc1Man9GlcNAc2 alpha-1,3-glucosyltransfera	0	0	75926.08166	65723.97241	191174.7513	1052542.669	65953.74224	702787.3055	0
ALG9	alpha-1,2-mannosyltransferase ALG9 isoform a [Homo sapiens]	0	0	0	0	0	0	0	146744.1187	151611.3268
ALKBH5	RNA demethylase ALKBH5 [Homo sapiens]	152109.2853	232135.0449	254730.41	262436.9228	229155.4218	236490.1651	197202.1607	55624.79805	291145.7693
ALPI	intestinal-type alkaline phosphatase precursor [Homo sapiens]	4855914.19	4839303.682	5416639.729	5185987.866	6557661.051	5355109.056	4187019.171	3229921.086	4153460.39
ALPP	alkaline phosphatase, placental type preproprotein [Homo sapiens]	16339924.85	17818335.06	22510341.73	18895070.91	23661536.45	19863484.6	16894518.27	13827214.93	16634221.23
ALYREF	THO complex subunit 4 [Homo sapiens]	5115592.91	4996284.932	7123274.85	6457020.111	6837294.115	6586242.218	6213097.503	5391646.802	5987563.509
AMDHD2	putative N-acetylglucosamine-6-phosphate deacetylase isoform 2 [Homo sapien	80050.53546	379824.1567	236380.8443	137505.4699	1042870.81	145574.2131	404955.5534	112399.5707	202592.3989
AMFR	E3 ubiquitin-protein ligase AMFR [Homo sapiens]	40375.95641	56323.07903	31203.36731	109315.9897	25829.54648	19326.67308	50863.17372	51414.21282	7731.446795
AMMECR1L	AMMECR1-like protein [Homo sapiens]	54625.37418	54660.45312	85129.89364	68586.77083	90615.61298	66766.07402	39639.49808	0	48193.25205
AMOTL2	angiomotin-like protein 2 [Homo sapiens]	0	0	0	0	0	0	14154.8007	0	23702.89628
AMPD2	AMP deaminase 2 isoform 1 [Homo sapiens]	249479.7197	250968.788	548122.4289	339299.4263	287506.334	330524.7623	411792.9986	233257.046	298862.6866
ANAPC1	anaphase-promoting complex subunit 1 [Homo sapiens]	92702.12195	76578.8746	84277.97488	79190.7034	81994.81098	57002.52758	75243.17981	62635.09082	108737.5449
ANAPC10	anaphase-promoting complex subunit 10 isoform 2 [Homo sapiens]	0	0	0	0	0	0	666430.2957	0	150015.2835
ANAPC15	anaphase-promoting complex subunit 15 isoform a [Homo sapiens]	1404824.75	1236301.643	1430853.268	1325167.384	0	1440745.214	1456441.116	1138090.768	1293818.911
ANAPC2	anaphase-promoting complex subunit 2 [Homo sapiens]	32363.80486	81944.73865	106161.7947	104570.0226	80838.29116	92799.85552	43416.86019	72546.3652	86273.00166
ANAPC4	anaphase-promoting complex subunit 4 [Homo sapiens]	35913.69204	110291.6468	125170.625	126922.9498	136985.5303	47282.05104	52319.81301	75761.6263	100283.694
ANAPC5	anaphase-promoting complex subunit 5 isoform a [Homo sapiens]	145980.0326	160515.8811	139912.076	149117.5638	192719.4131	200963.6466	109874.4255	50857.31257	128981.3494
ANAPC7	anaphase-promoting complex subunit 7 isoform a [Homo sapiens]	325692.4155	431018.9276	417055.1955	344085.4238	424925.5928	442713.9486	341659.7571	285915.5281	354957.8763
ANKFY1	ankyrin repeat and FYVE domain-containing protein 1 isoform 3 [Homo sapiens	239718.1127	268891.091	329893.7656	262566.8282	1484111.52	1863142.555	2049854.748	1597893.393	318264.3915
ANKH	progressive ankylosis protein homolog [Homo sapiens]	135038.7948	68126.28723	160309.0142	110851.6694	142171.4592	92935.49028	30834.10816	48490.60062	64085.54433
ANKHD1-EIF4EBP3	ANKHD1-EIF4EBP3 protein [Homo sapiens]	69111.80755	81087.92523	72009.82698	73303.07684	100222.1029	82480.30306	61659.4087	45657.07229	53972.21584
ANKLE2	ankyrin repeat and LEM domain-containing protein 2 [Homo sapiens]	63257.89657	52695.83595	47964.18291	66765.73395	70134.3037	64551.72489	35999.24044	26128.11126	43774.99202
ANKMY2	ankyrin repeat and MYND domain-containing protein 2 [Homo sapiens]	197260.08	337757.2143	290762.7943	189317.5333	474303.0529	194926.0522	150466.5643	148956.3538	129862.5729
ANKRD12	ankyrin repeat domain-containing protein 12 isoform 1 [Homo sapiens]	0	0	0	0	0	0	320371.1316	0	213996.8035
ANKRD13D	ankyrin repeat domain-containing protein 13D [Homo sapiens]	0	0	0	0	0	0	47595.08057	36799.21209	0
ANKRD17	ankyrin repeat domain-containing protein 17 isoform a [Homo sapiens]	41913.9046	60337.34303	71912.35058	48924.00434	126911.2059	60302.19533	48194.19119	78537.05097	70260.15077
ANKRD28	serine/threonine-protein phosphatase 6 regulatory ankyrin repeat subunit A	180571.2814	138255.5804	170363.0301	168739.8706	105127.1854	97409.6961	129723.0444	156025.712	245555.1183
ANKRD40	ankyrin repeat domain-containing protein 40 [Homo sapiens]	142085.4569	0	347235.8347	200870.7231	354861.8125	142323.9762	0	0	0
ANKRD52	serine/threonine-protein phosphatase 6 regulatory ankyrin repeat subunit C	41274.2546	54288.0689	71126.73353	68146.81793	49374.88203	40521.73737	45787.7405	28459.99323	65762.72983
ANKRD54	ankyrin repeat domain-containing protein 54 [Homo sapiens]	190129.554	152808.3175	443824.7214	224350.9477	224602.6949	191685.5821	163645.9277	0	133404.2086
ANKS1A	ankyrin repeat and SAM domain-containing protein 1A [Homo sapiens]	61565.95658	61341.8675	46637.6239	50444.59875	46446.32658	0	49773.05353	19265.67344	51396.35918
ANKZF1	ankyrin repeat and zinc finger domain-containing protein 1 [Homo sapiens]	66758.3913	66853.61306	82676.68059	78717.57977	94854.72792	48791.98007	59540.48453	56180.82186	70940.37736
ANLN	actin-binding protein anillin [Homo sapiens]	102061.6418	112006.0476	119130.4286	136182.741	144040.309	117681.0044	111350.3972	78115.63099	88782.00432
ANO10	anoctamin-10 isoform 1 [Homo sapiens]	54564.90702	130474.4225	199336.7306	120002.5617	85662.79204	146979.2667	105274.7072	33194.90222	158681.6619
ANO4	anoctamin-4 [Homo sapiens]	0	0	0	0	0	0	1157604.348	0	941819.2167
ANO6	anoctamin-6 isoform d [Homo sapiens]	126059.8844	49525.43293	87350.52786	42126.9956	170281.3915	43481.24487	32140.69062	66312.32689	152858.3862
ANP32A	acidic leucine-rich nuclear phosphoprotein 32 family member A [Homo sapiens	13499974.41	16069165.86	17041648.13	15110334.99	18771863.29	16928486.11	16969269.49	14157360.19	16274198.67
ANP32B	acidic leucine-rich nuclear phosphoprotein 32 family member B [Homo sapiens	18654437.38	21159113.71	21466480.3	19498487.78	21421461	20393754.57	19118124.47	18209079.71	21946787.21
ANP32E	acidic leucine-rich nuclear phosphoprotein 32 family member E isoform 1 [Ho	7297799.205	10317698.76	10132157.28	11065434.21	9660414.521	10000430.96	11153312.12	10342565.07	6369642.785

ANP32E	acidic leucine-rich nuclear phosphoprotein 32 family member E isoform 3 [Ho	7297799.205	10317698.76	10132157.28	11065434.21	9660414.521	10000430.96	11153312.12	10342565.07	6369642.785
ANPEP	aminopeptidase N precursor [Homo sapiens]	0	74509.76839	25004.01672	0	124289.2266	38318.30341	49634.44284	22469.76421	100336.2504
ANTXR1	anthrax toxin receptor 1 isoform 1 precursor [Homo sapiens]	0	0	0	0	0	0	55389.02637	0	75821.84314
ANXA1	annexin A1 [Homo sapiens]	48020143.43	25371396.54	63450081.9	50797913.77	58388618.1	56396467.95	25990950.61	46947003.9	28126293.95
ANXA11	annexin A11 isoform 1 [Homo sapiens]	3195144.12	3367431.734	4291605.624	3718586.155	4080276.529	3794201.39	3509525.855	2912587.952	3307539.324
ANXA2	annexin A2 isoform 2 [Homo sapiens]	116243717.3	124088466.2	153523151.3	123349408.6	150960583.4	133895976.7	132936362.4	111197799.6	136134979
ANXA3	annexin A3 [Homo sapiens]	4121236.159	5223479.465	5473109.589	4923708.52	5418563.921	5139905.691	5463670.553	4362375.88	5981959.276
ANXA4	annexin A4 [Homo sapiens]	1660004.203	2019840.637	1681606.219	2233744.648	2048666.323	2413683.198	1947056.228	1527460.052	2071987.125
ANXA5	annexin A5 [Homo sapiens]	12335416.18	15508120.41	16903974.47	14559656.94	16310268.21	17235061.86	18147273.81	15125077.15	17451511.89
ANXA6	annexin A6 isoform 1 [Homo sapiens]	717323.3105	1070617.976	1235840.125	980022.2867	1066138.71	1005036.976	1119725.214	786524.9308	855438.8832
ANXA7	annexin A7 isoform 2 [Homo sapiens]	2773825.406	3380690.46	4344613.415	2902355.762	3487636.42	3483245.261	3171807.665	2514681.872	3280708.866
ANXA8	annexin A8 isoform 2 [Homo sapiens]	1196478.46	93477.08156	363129.3191	0	172467.617	0	0	0	0
ANXA8L2	annexin A8-like protein 2 [Homo sapiens]	0	1270441.25	1258888.403	1082477.519	1176536.409	1200543.261	1118430.431	1178988.825	1384307.243
AP1B1	AP-1 complex subunit beta-1 isoform b [Homo sapiens]	655733.342	0	843372.0405	42550.21481	862493.5805	0	389086.0755	281154.4499	678293.588
AP1B1	AP-1 complex subunit beta-1 isoform a [Homo sapiens]	655733.342	0	843372.0405	42550.21481	862493.5805	0	389086.0755	281154.4499	678293.588
AP1G1	AP-1 complex subunit gamma-1 isoform a [Homo sapiens]	661806.4687	756005.5452	505158.5217	695314.5658	805779.726	760435.5875	670434.3762	659404.0633	697775.1897
AP1G2	AP-1 complex subunit gamma-like 2 [Homo sapiens]	14070.65421	0	33735.65421	5179.855605	60323.54981	0	0	0	0
AP1M1	AP-1 complex subunit mu-1 isoform 1 [Homo sapiens]	1046325.407	0	1469727.477	1225720.266	1448475.412	0	1329994.836	1058181.106	0
AP1S1	AP-1 complex subunit sigma-1A [Homo sapiens]	1326503.253	1372592.496	1717274.588	1568996.714	1553882.04	1333405.555	1499899.103	694027.8622	1805279.551
AP2A1	AP-2 complex subunit alpha-1 isoform 1 [Homo sapiens]	568033.6668	598082.8491	1707463.116	1431489.349	1729219.512	1368954.057	558667.3792	446665.3162	621739.8491
AP2A2	AP-2 complex subunit alpha-2 isoform 1 [Homo sapiens]	324006.7529	405080.5666	453777.117	379541.878	453205.8378	366086.0062	365310.0204	264321.56	317034.0319
AP2B1	AP-2 complex subunit beta isoform a [Homo sapiens]	1271086.177	1374482.714	1095721.897	1237999.751	1832990.657	1396465.358	1298809.188	1133618.577	1231487.573
AP2M1	AP-2 complex subunit mu isoform a [Homo sapiens]	1349597.453	1287053.298	1403214.952	1084030.798	1298568.048	1364155.286	1271258.604	1106463.326	1132579.44
AP2S1	AP-2 complex subunit sigma isoform AP17 [Homo sapiens]	1808372.561	2093418.064	3283840.345	2002979.491	3411782.2	1441421.855	2060105.233	913470.0784	2462989.182
AP3B1	AP-3 complex subunit beta-1 isoform 1 [Homo sapiens]	185299.9623	315152.7629	487155.0282	413691.2853	513790.0438	405757.6981	396140.7	289245.7704	408853.0723
AP3D1	AP-3 complex subunit delta-1 isoform 3 [Homo sapiens]	321626.1065	287775.5841	276325.9899	355173.0415	439287.9197	375069.3256	321343.0576	313886.3457	518711.8852
AP3M1	AP-3 complex subunit mu-1 [Homo sapiens]	980980.937	1266894.129	1101423.561	1294197.651	1575357.527	1340834.959	1110489.156	984421.8425	1136006.284
AP3S1	AP-3 complex subunit sigma-1 [Homo sapiens]	1083674.919	692176.5	1490436.545	1000423.264	973434.623	1648109.623	2111612.113	775477.0007	4203555.703
AP3S2	AP-3 complex subunit sigma-2 [Homo sapiens]	0	0	0	0	0	0	789231.8629	715943.7258	760096.5171
AP4B1	AP-4 complex subunit beta-1 isoform 1 [Homo sapiens]	0	0	0	0	0	0	51190.38476	84222.79647	53325.91777
AP4M1	AP-4 complex subunit mu-1 [Homo sapiens]	131845.9619	0	110928.3689	0	173042.5686	80153.01829	177102.8506	111051.5488	0
AP4S1	AP-4 complex subunit sigma-1 isoform 2 [Homo sapiens]	0	0	0	0	0	0	387181.2166	0	328409.1161
AP5B1	AP-5 complex subunit beta-1 [Homo sapiens]	22485.82449	22429.20306	0	0	17276.97271	18515.41939	0	13166.40663	15178.98061
AP5S1	AP-5 complex subunit sigma-1 [Homo sapiens]	137124.9667	0	169588.51	134936.71	0	105100.0567	120064.5	94821.87084	0
AP5Z1	AP-5 complex subunit zeta-1 [Homo sapiens]	22407.23403	35056.14232	35451.06865	34868.50246	38277.88664	35989.19736	27948.2082	0	35279.67874
APAF1	apoptotic protease-activating factor 1 isoform c [Homo sapiens]	20949.67603	15738.73825	25257.36552	11572.68022	23321.25798	19744.11123	15799.93503	7663.245193	7635.543681
APBB1IP	amyloid beta A4 precursor protein-binding family B member 1-interacting pro	12152.69034	38743.68636	0	32393.94318	0	49290.85227	0	0	0
APC	adenomatous polyposis coli protein isoform b [Homo sapiens]	3290.018003	5481.863518	7402.275473	8701.086844	3761.76957	2721.680312	4020.485725	0	11323.11834
APEH	acylamino-acid-releasing enzyme [Homo sapiens]	1640628.139	1668016.649	2052287.955	1691010.674	2073551.806	1572319.005	1480680.999	1254614.782	1678444.84
APEX1	DNA-(apurinic or apyrimidinic site) lyase [Homo sapiens]	6870555.492	8651294.561	5536389.902	5967718.762	10835970.64	6484303.965	6206281.497	5088278.488	9002953.068
APEX2	DNA-(apurinic or apyrimidinic site) lyase 2 isoform 1 [Homo sapiens]	85584.74569	0	123168.1751	114802.8299	0	125955.552	0	0	0
APH1A	gamma-secretase subunit APH-1A isoform 1 [Homo sapiens]	198957.0862	0	132912.2457	177152.6962	0	215174.2112	0	237141.7069	179588.5
API5	apoptosis inhibitor 5 isoform b [Homo sapiens]	992728.7555	1101616.134	1352189.375	1140329.064	0	1137469.519	1116100.173	932288.101	1145169.169
API5	apoptosis inhibitor 5 isoform a [Homo sapiens]	992728.7555	1101616.134	1352189.375	1140329.064	0	1137469.519	1116100.173	932288.101	1145169.169
APIP	methylthioribulose-1-phosphate dehydratase [Homo sapiens]	613066.6863	589840.1863	670455.955	648416.1604	909232.8264	699447.5275	468729.5522	519738.4465	674314.0108
APLP2	amyloid-like protein 2 isoform 1 precursor [Homo sapiens]	107400.8856	102812.3744	133762.3858	118029.5719	136300.0905	144138.6602	34319.81084	55601.27114	0
APMAP	adipocyte plasma membrane-associated protein [Homo sapiens]	1001802.142	1089362.341	1250843.275	1046332.204	1334315.146	1062016.517	1185660.193	1008069.949	1164907.522
APOA1	apolipoprotein A-I preproprotein [Homo sapiens]	0	0	0	0	0	0	0	0	0
APOA1BP	NAD(P)H-hydrate epimerase precursor [Homo sapiens]	9926871.577	10804346.2	12726940.67	8793638.997	12669050.7	11784269.51	10636734	9343470.328	10661420.56
APOB	apolipoprotein B-100 precursor [Homo sapiens]	23811.38221	25184.41531	35542.56286	17214.55792	43463.49484	19214.8993	28876.0028	26057.36612	51898.59386
APOBEC3B	DNA dC-_dU-editing enzyme APOBEC-3B isoform a [Homo sapiens]	634118.7951	530262.2134	793579.7256	423594.3559	667965.2254	382959.0364	403592.0992	1017404.053	401807.272
APOBEC3C	DNA dC-_dU-editing enzyme APOBEC-3C [Homo sapiens]	627887.6514	620537.5194	1195929.863	1057567.796	1365853.599	1046998.773	650875.2077	756744.5634	877642.8979
APOE	apolipoprotein E precursor [Homo sapiens]	0	0	0	0	0	0	311737.3208	148640.6556	0
APOL2	apolipoprotein L2 [Homo sapiens]	401521.7387	331168.8358	671377.4127	553708.0837	599366.2044	467732.2606	549013.0463	255511.2063	437796.4878
APOO	apolipoprotein O precursor [Homo sapiens]	1460455.946	2292442.887	2306883.879	1778436.161	2922676.859	2354330.614	2346088.699	2013077.997	2811097.627
APOOL	apolipoprotein O-like precursor [Homo sapiens]	119487.4454	321256.3406	163677.6632	612764.8634	0	95223.77259	389762.2272	58698.25926	163851.7259
APP	amyloid beta A4 protein isoform a precursor [Homo sapiens]	200594.2994	131313.1078	181238.6895	132623.988	54418.11752	147228.2524	48221.71066	0	97538.8893
APPL1	DCC-interacting protein 13-alpha [Homo sapiens]	103781.9049	108782.5514	185222.0543	109798.4535	230491.9396	101946.0313	116785.6817	136910.3456	130526.531
APRT	adenine phosphoribosyltransferase isoform a [Homo sapiens]	9675814.739	10858177.69	11228106.73	11934180.2	13685420.05	11886443.91	11275841.12	10885767.25	12084390.79
APTX	aprataxin isoform e [Homo sapiens]	61781.175	181551.0219	43447.72656	136275.325	76822.67188	155709.4653	150765.9391	89004.2875	149418.8984
AQR	intron-binding protein aquarius [Homo sapiens]	319627.1746	254488.0134	379574.1879	368039.8384	414228.8959	272808.1334	321700.8582	199650.0631	312890.6246
ARAF	serine/threonine-protein kinase A-Raf isoform 1 [Homo sapiens]	162445.9023	185499.3503	234793.7901	150880.0693	202149.2502	0	0	139575.8367	162787.4953
ARAF	serine/threonine-protein kinase A-Raf isoform 2 [Homo sapiens]	162445.9023	185499.3503	234793.7901	150880.0693	202149.2502	0	0	139575.8367	162787.4953
ARAP1	arf-GAP with Rho-GAP domain, ANK repeat and PH domain-containing protein 1	184824.9337	114538.3997	107603.9992	95770.86005	114991.201	116567.6058	106190.8642	98458.80251	114482.4077
ARCN1	coatomer subunit delta isoform 1 [Homo sapiens]	2411990.716	2573396.688	3105197.495	2741495.794	3225837.797	2875175.534	2816269.453	2464700.313	2830065.352
ARF1	ADP-ribosylation factor 1 [Homo sapiens]	810065.5185	34227677.34	1144872.979	48014.23152	724566.8892	320023.0431	333597.9569	29968994.83	33962492.72
ARF3	ADP-ribosylation factor 3 [Homo sapiens]	718998.8048	0	1019500.305	280512.5479	561037.3356	304214.8493	0	0	0
ARF4	ADP-ribosylation factor 4 [Homo sapiens]	16835560.78	18478546.62	21116982.42	18639382.38	23873224	19603412.04	18754194.09	14771045.71	20272213.91
ARF5	ADP-ribosylation factor 5 [Homo sapiens]	840472.9164	2070049.145	804831.7171	1379033.692	1476496.981	1484349.661	2046111.678	1640538.029	2238617.572
ARF6	ADP-ribosylation factor 6 [Homo sapiens]	2013010.078	2955780.044	2700189.969	2726784.146	2991986.9	2820239.259	3071833.37	2415867.885	3605838.948
ARFGAP1	ADP-ribosylation factor GTPase-activating protein 1 isoform a [Homo sapiens	313567.3789	798939.3933	753613.2342	729095.9235	686690.7671	950889.8416	503492.9933	426017.651	712043.698
ARFGAP2	ADP-ribosylation factor GTPase-activating protein 2 isoform 1 [Homo sapien	284359.1952	427796.3712	468025.2689	369292.7494	599657.5205	311509.2405	352561.5984	293620.1838	435693.284
ARFGAP3	ADP-ribosylation factor GTPase-activating protein 3 isoform 1 [Homo sapien	65383.46732	30096.86737	79385.96451	80192.59507	0	88270.49333	0	0	0
ARFGEF1	brefeldin A-inhibited guanine nucleotide-exchange protein 1 [Homo sapiens]	21694.68241	13977.68738	47098.47627	43519.35595	46680.02649	72897.93303	106513.1453	40132.44559	19712.19052
ARFGEF2	brefeldin A-inhibited guanine nucleotide-exchange protein 2 [Homo sapiens]	9820.975009	24791.5901	0	28842.99361	18661.32602	0	0	14877.90936	16950.74617
ARFIP1	arfaptin-1 isoform 2 [Homo sapiens]	200013.9751	147782.6201	230297.6931	249930.195	221303.1903	189523.4661	193922.2395	189922.9975	213408.0541
ARFIP1	arfaptin-1 isoform 1 [Homo sapiens]	200013.9751	147782.6201	230297.6931	249930.195	221303.1903	189523.4661	193922.2395	189922.9975	213408.0541
ARFIP2	arfaptin-2 isoform 1 [Homo sapiens]	394099.5242	357490.9504	394481.6767	269004.3018	485591.9933	301053.9216	304376.5211	187907.4177	176309.8218
ARFRP1	ADP-ribosylation factor-related protein 1 isoform a [Homo sapiens]	223600.6676	311649.5681	251636.3692	297244.0559	311959.5412	173344.2039	273023.9647	224027.0294	258555.9912
ARG1	arginase-1 isoform 1 [Homo sapiens]	0	0	0	0	0	0	121507.125	74000.82469	0
ARG2	arginase-2, mitochondrial precursor [Homo sapiens]	124956.2212	0	65357.50221	0	58622.42423	213828.823	0	0	0
ARHGAP1	rho GTPase-activating protein 1 [Homo sapiens]	2012767.467	2084231.15	2562153.052	2076329.093	2745886.049	2172359.621	2061259.894	1877689.297	1858895.704
ARHGAP11A	rho GTPase-activating protein 11A isoform 1 [Homo sapiens]	0	0	0	0	0	0	14204.90037	10340.72846	0
ARHGAP12	rho GTPase-activating protein 12 isoform 1 [Homo sapiens]	0	11841.1834	23659.64336	30682.88949	0	30819.71169	25314.91684	0	48260.96328
ARHGAP17	rho GTPase-activating protein 17 isoform 1 [Homo sapiens]	176308.7843	275468.6144	344667.3345	267301.7592	256920.5491	282819.3808	272745.5367	221253.1319	201509.1371
ARHGAP18	rho GTPase-activating protein 18 [Homo sapiens]	0	0	96175.55355	57184.73198	93278.49915	69079.63993	69633.57863	47992.87819	78820.96389
ARHGAP21	rho GTPase-activating protein 21 [Homo sapiens]	0	7884.790211	4780.449775	4891.1	0	4895.534827	0	0	0
ARHGAP33	rho GTPase-activating protein 33 isoform 1 [Homo sapiens]	0	0	0	0	0	0	0	0	0
ARHGAP35	rho GTPase-activating protein 35 [Homo sapiens]	144065.6641	8482.018688	150294.1267	31178.91534	93760.03872	35340.56436	13475.39522	30320.35642	63942.88796
ARHGAP40	rho GTPase-activating protein 40 [Homo sapiens]	259787.0708	363881.0735	369020.5	362993.9651	234137.7243	355367.0349	317660.9954	309331.932	352148.3015
ARHGAP5	rho GTPase-activating protein 5 isoform a [Homo sapiens]	1615997.107	11781.64488	30063.09832	38882.42605	19829.79549	0	11335.76685	12921.04932	18033.61884
ARHGDIA	rho GDP-dissociation inhibitor 1 isoform a [Homo sapiens]	13517155.29	15683234.84	17767878.85	16224296.03	19875507.17	11761926.7	15450909.34	14635649.71	18654561.76
ARHGDIB	rho GDP-dissociation inhibitor 2 [Homo sapiens]	89935.25749	0	159814.3711	0	176247.763	119325.0489	0	137726.0469	87000.4526
ARHGDIG	rho GDP-dissociation inhibitor 3 [Homo sapiens]	0	0	0	0	0	0	198907.4799	0	162475.4383
ARHGEF1	rho guanine nucleotide exchange factor 1 isoform 1 [Homo sapiens]	161837.7601	231288.8394	241843.916	230361.9162	343348.2034	239972.8617	199577.3537	167815.8344	305746.4538
ARHGEF10	rho guanine nucleotide exchange factor 10 [Homo sapiens]	26323.25977	27435.8766	67769.62241	18677.71976	36883.32852	59951.20197	26723.44641	31841.20866	24189.09157
ARHGEF16	rho guanine nucleotide exchange factor 16 [Homo sapiens]	142279.8431	114199.9504	93987.53925	152911.8127	185452.0179	182143.7619	126255.8636	72088.93902	135742.6649
ARHGEF2	rho guanine nucleotide exchange factor 2 isoform 1 [Homo sapiens]	328582.8609	337905.106	454117.3404	343003.93	432169.13	379874.6166	331120.3598	268553.8258	366107.9188
ARHGEF7	rho guanine nucleotide exchange factor 7 isoform a [Homo sapiens]	0	34140.50896	33520.81027	57035.28835	52826.68575	50294.231	37088.00425	0	40666.19198
ARHGEF7	rho guanine nucleotide exchange factor 7 isoform c [Homo sapiens]	0	34140.50896	33520.81027	57035.28835	52826.68575	50294.231	37088.00425	0	40666.19198
ARID1A	AT-rich interactive domain-containing protein 1A isoform a [Homo sapiens]	76527.54093	73203.47412	98745.76711	66262.271	97785.99535	63219.19053	36499.41339	54792.5	87342.14298
ARID2	AT-rich interactive domain-containing protein 2 [Homo sapiens]	40560.80801	23257.69589	0	7210.575655	55127.72994	11482.20868	12687.36697	0	21866.15503
ARID4B	AT-rich interactive domain-containing protein 4B isoform 1 [Homo sapiens]	0	0	0	0	0	0	22274.36552	18208.54133	30476.58718
ARIH1	E3 ubiquitin-protein ligase ARIH1 [Homo sapiens]	297267.7471	386261.8893	300854.8979	346800.8325	305174.8771	360487.3009	308930.5072	310146.456	321515.0189
ARIH2	E3 ubiquitin-protein ligase ARIH2 [Homo sapiens]	439782.603	302679.5928	649488.9995	524352.4778	636229.2571	494856.9427	552924.0934	397501.3714	336883.3934
ARL1	ADP-ribosylation factor-like protein 1 [Homo sapiens]	4022781.784	3781767.427	5304837.391	3673988.686	5411186.844	4457024.336	4023640.589	3125650.703	3907633.473
ARL2	ADP-ribosylation factor-like protein 2 isoform 1 [Homo sapiens]	1073617.734	1247891.944	1227346.651	1291764.03	1294257.958	1282641.385	1017597.778	911063.7577	1010159.587
ARL2BP	ADP-ribosylation factor-like protein 2-binding protein [Homo sapiens]	384308.6833	0	614965.2222	1145504.378	505198.6389	0	0	0	0
ARL3	ADP-ribosylation factor-like protein 3 [Homo sapiens]	2199487.869	2074902.417	2467887.283	2836591.006	2396999.135	2968111.879	2943389.39	2253790.444	3011983.485
ARL6	ADP-ribosylation factor-like protein 6 [Homo sapiens]	307282.3689	145697.4939	146949.6616	0	0	1049040.836	172528.5748	0	469666.5809
ARL6IP1	ADP-ribosylation factor-like protein 6-interacting protein 1 [Homo sapiens]	1701210.918	848285.1882	3429264.017	1765244.558	1987063.792	1614042.22	1319812.814	1151600.206	1754997.788
ARL6IP4	ADP-ribosylation factor-like protein 6-interacting protein 4 isoform 1 [Hom	177971.2436	198113.6043	166871.1096	206450.3583	332849.9682	284363.4699	113537.2126	120918.1661	168097.2941
ARL6IP5	PRA1 family protein 3 [Homo sapiens]	7447853.995	8307043.889	9627127.678	7845265.025	9300279.1	8598792.308	5960734.425	7244283.611	7613969.386
ARL6IP6	ADP-ribosylation factor-like protein 6-interacting protein 6 [Homo sapiens]	45428.73269	1005795.179	976918.0212	0	1086023.062	0	0	0	0
ARL8B	ADP-ribosylation factor-like protein 8B [Homo sapiens]	2712221.063	3466563.707	2474272.492	3701528.224	1587111.715	3865155.613	3776345.469	3186553.021	4475644.46
ARMC1	armadillo repeat-containing protein 1 [Homo sapiens]	922702.192	1006120.763	1499646.542	824376.8007	1056730.026	1269765.857	909570.9608	820156.5392	828495.5572
ARMC10	armadillo repeat-containing protein 10 isoform a [Homo sapiens]	409444.7952	224460.2455	456302.6476	173102.872	434239.8381	434655.8881	41256.55357	153365.5333	0
ARMC6	armadillo repeat-containing protein 6 isoform 1 [Homo sapiens]	566003.806	595741.7719	751823.7602	682382.1684	807848.5626	753760.6725	729083.8269	600641.5146	726004.5351
ARMC8	armadillo repeat-containing protein 8 isoform 2 [Homo sapiens]	96965.24967	81780.2716	108486.5759	99539.49091	130728.3402	0	101442.7625	110799.8875	0
ARMCX3	armadillo repeat-containing X-linked protein 3 [Homo sapiens]	397198.5463	340326.7883	422179.1045	553486.9739	487245.5911	540170.3178	585638.4959	215823.9681	711858.0407

ARNT	aryl hydrocarbon receptor nuclear translocator isoform 1 [Homo sapiens]	35745.62853	0	91979.05645	47989.99899	67755.17867	53759.92137	0	0	0
ARPC1A	actin-related protein 2/3 complex subunit 1A isoform 1 [Homo sapiens]	253594.026	274744.044	336069.5388	221383.1285	387470.523	197283.6474	287699.0593	238470.9589	257342.2108
ARPC1B	actin-related protein 2/3 complex subunit 1B [Homo sapiens]	2823856.371	2570890.013	3098536.592	2867942.764	3336993.959	2994135.403	2260978.052	2213291.709	2387332.691
ARPC2	actin-related protein 2/3 complex subunit 2 [Homo sapiens]	3623288.837	3844439.221	4298964.402	3846952.291	4590795.842	3928146.103	3491389.729	2675733.857	3709074.089
ARPC3	actin-related protein 2/3 complex subunit 3 [Homo sapiens]	3659573.975	2238134.855	2094758.48	3263385.204	1691632.704	3394284.629	3360175.551	3064232.613	2012585.216
ARPC4	actin-related protein 2/3 complex subunit 4 isoform c [Homo sapiens]	3267512.715	0	3097404.878	3343581.695	4394367.691	0	0	2525907.39	3834351.895
ARPC4	actin-related protein 2/3 complex subunit 4 isoform a [Homo sapiens]	3267512.715	0	3097404.878	3343581.695	4394367.691	0	0	2525907.39	3834351.895
ARPC5	actin-related protein 2/3 complex subunit 5 isoform 2 [Homo sapiens]	2862049.07	0	3774481.204	2294366.32	2484951.734	0	1882451.72	0	611702.6145
ARPC5	actin-related protein 2/3 complex subunit 5 isoform 1 [Homo sapiens]	2862049.07	0	3774481.204	2294366.32	2484951.734	0	1882451.72	0	611702.6145
ARPC5L	actin-related protein 2/3 complex subunit 5-like protein [Homo sapiens]	2591279.623	2620086.201	2614250.033	3094567.519	2773393.08	2118762.512	2472557.597	2670674.575	3633684.717
ARPP19	cAMP-regulated phosphoprotein 19 [Homo sapiens]	913267.8417	1067415.606	1660911.008	1148170.776	1273946.942	1062913.601	984092.4833	1010492.767	1215644.544
ARRB1	beta-arrestin-1 isoform A [Homo sapiens]	275870.4535	587523.5078	559826.0073	613427.3246	646646.8626	568476.8464	635229.8514	401026.9399	611502.924
ARRB2	beta-arrestin-2 isoform 3 [Homo sapiens]	0	0	0	0	0	0	118472.0217	76988.73505	48189.68563
ARSA	arylsulfatase A isoform a precursor [Homo sapiens]	0	0	122521.3087	196801.7543	193855.9424	230436.5261	182769.5546	50229.2837	117702.7217
ASAH1	acid ceramidase isoform b [Homo sapiens]	0	0	0	0	0	0	97895.35	0	297309.2906
ASB6	ankyrin repeat and SOCS box protein 6 isoform 1 [Homo sapiens]	149285.3201	123861.5165	169894.2976	138453.5655	158641.1122	167153.5844	101758.5831	0	134402.7549
ASB9	ankyrin repeat and SOCS box protein 9 isoform 1 [Homo sapiens]	1344514.558	1072452.789	768213.174	1095771.419	1360574.395	1187592.767	1019167.493	461515.9695	329452.9535
ASCC1	activating signal cointegrator 1 complex subunit 1 isoform b [Homo sapiens]	0	472478.2791	314580.125	369738.8601	0	483989.8245	0	265230.5918	281173.014
ASCC2	activating signal cointegrator 1 complex subunit 2 isoform 1 [Homo sapiens]	200538.3426	230363.9435	256144.6382	202114.4661	230432.747	173561.7556	176377.6186	140649.0949	258544.8425
ASCC3	activating signal cointegrator 1 complex subunit 3 isoform a [Homo sapiens]	82989.98291	79837.40168	100340.8623	165264.9422	255722.1523	224874.4421	72386.68327	63654.53521	66940.524
ASF1A	histone chaperone ASF1A [Homo sapiens]	1092061.84	1093643.289	999183.9468	1225573.114	996305.7262	1297224.494	886689.4468	581856.1949	954192.3777
ASF1B	histone chaperone ASF1B [Homo sapiens]	2123202.75	1290369.243	3559095.793	2004050.451	3864546.937	2418189.615	2027227.909	1199247.199	2031175.492
ASH1L	histone-lysine N-methyltransferase ASH1L [Homo sapiens]	1345.441543	6507.723841	0	10651.25731	0	8344.304466	0	0	0
ASH2L	set1/Ash2 histone methyltransferase complex subunit ASH2 isoform a [Homo sa	154762.3857	146684.7181	95716.31202	156484.8229	107826.9301	244021.4425	174624.2739	133268.5222	219867.2441
ASL	argininosuccinate lyase isoform 1 [Homo sapiens]	104470.5756	106730.6802	133493.6003	110932.8877	62989.09302	173066.0727	76213.88262	134346.7791	0
ASMTL	N-acetylserotonin O-methyltransferase-like protein isoform 1 [Homo sapiens]	167978.0488	173940.235	368487.8879	90757.59793	1604351.502	189080.9159	260851.2742	124289.9198	182924.6466
ASNA1	ATPase ASNA1 [Homo sapiens]	2470827.807	2280650.848	3722075.839	3185586.63	4011399.27	3187302.956	2624603.48	2565774.586	2927400.046
ASNS	asparagine synthetase [glutamine-hydrolyzing] isoform a [Homo sapiens]	1076482.388	1394817.637	1417118.651	1361930.441	1446794.679	1541132.993	1472584.721	1244060.839	1596499.215
ASNSD1	asparagine synthetase domain-containing protein 1 [Homo sapiens]	0	0	0	0	0	0	34427.14857	34081.7541	30316.46734
ASPH	aspartyl/asparaginyl beta-hydroxylase isoform a [Homo sapiens]	1443604.722	846928.2434	2007766.802	1894067.188	2046531.47	2052982.103	2119069.701	718082.0358	1976696.337
ASPSCR1	tether containing UBX domain for GLUT4 isoform 2 [Homo sapiens]	22379.55558	0	30291.82319	25891.02863	0	18102.95247	12400.31219	10175.58674	21631.03822
ASRGL1	isoaspartyl peptidase/L-asparaginase [Homo sapiens]	199232.2397	255155	271928.1262	212778.0267	204871.7627	209454.1974	0	250135.068	217627.8535
ASS1	argininosuccinate synthase [Homo sapiens]	29593808.7	29738563.4	37670127.76	30900254.57	38298417.93	32387642.51	26987897.99	22446808.63	28300759.22
ASUN	protein asunder homolog [Homo sapiens]	167516.727	329965.032	177525.1521	198728.2932	222707.4237	165703.6108	134197.9193	108251.2865	99013.25027
ASXL2	putative Polycomb group protein ASXL2 [Homo sapiens]	0	0	0	0	0	0	1770.225292	0	9088.415187
ATAD1	ATPase family AAA domain-containing protein 1 [Homo sapiens]	456274.5618	646144.1333	612710.5332	825511.0673	610498.4278	989753.5477	886166.9902	453320.5971	840946.0072
ATAD2	ATPase family AAA domain-containing protein 2 [Homo sapiens]	38490.34338	52444.81008	47885.40182	51152.5417	65449.63109	44681.36555	22064.3396	0	41685.19314
ATAD3A	ATPase family AAA domain-containing protein 3A isoform 2 [Homo sapiens]	0	0	0	0	0	0	1509523.675	1197844.664	1539281.97
ATAD3B	ATPase family AAA domain-containing protein 3B [Homo sapiens]	183434.3566	157231.6271	230403.5452	232090.5308	211077.0393	226062.2713	198800.8029	91979.92705	206663.7962
ATE1	arginyl-tRNA--protein transferase 1 isoform 2 [Homo sapiens]	120578.6402	76570.7602	159082.6518	130807.5906	195357.9786	104733.361	133541.4592	168349.5931	0
ATF1	cyclic AMP-dependent transcription factor ATF-1 [Homo sapiens]	1507213.487	1096016.893	1311608.07	1665355.78	1408085.104	1335222.767	783671.3233	1497875.073	737166.7467
ATF6B	cyclic AMP-dependent transcription factor ATF-6 beta isoform a [Homo sapien	136926.0101	138971.7115	157692.3901	110632.6962	156273.0897	83549.63323	0	55213.73839	41274.0796
ATF7	cyclic AMP-dependent transcription factor ATF-7 isoform 2 [Homo sapiens]	121652.6618	83218.17409	0	101291.7289	222165.7752	277688.2403	216594.6291	162531.515	0
ATG13	autophagy-related protein 13 isoform 3 [Homo sapiens]	63863.30303	60547.39792	109011.272	76118.34291	73251.45661	93465.19091	83505.79442	59369.08395	40262.27803
ATG2A	autophagy-related protein 2 homolog A [Homo sapiens]	6492.265442	16865.92551	19802.22371	9727.725689	19458.01169	0	9221.06584	20340.38773	13843.99214
ATG3	ubiquitin-like-conjugating enzyme ATG3 [Homo sapiens]	3039719.983	3074972.078	3584047.714	3323110.166	3503459.056	3548853.421	3308131.329	2830728.361	3300557.146
ATG4B	cysteine protease ATG4B isoform a [Homo sapiens]	138188.8919	237535.1212	111561.1313	275650.6734	308476.3403	280182.3487	235057.1702	0	304600.7626
ATG5	autophagy protein 5 [Homo sapiens]	67809.07857	77251.25238	170320.3923	92763.95714	188753.3857	138420.1822	133321.3926	100543.0256	94026.4872
ATG7	ubiquitin-like modifier-activating enzyme ATG7 isoform a [Homo sapiens]	305909.5975	209241.3644	641622.6577	470504.8124	625280.6411	519764.7129	504184.9929	379387.3418	473528.3498
ATG9A	autophagy-related protein 9A [Homo sapiens]	54065.57494	50266.19386	164031.2301	132648.1726	134051.8208	116947.1685	0	159737.8964	67524.85458
ATIC	bifunctional purine biosynthesis protein PURH [Homo sapiens]	5025878.102	5468234.912	6575006.442	5532387.08	6504098.42	5888248.637	5388878.243	4791314.912	5092576.168
ATL2	atlastin-2 isoform 2 [Homo sapiens]	420070.8125	333630.0707	990794.0098	520080.2294	509260.5373	417292.5695	377398.854	233669.5482	536810.6074
ATL3	atlastin-3 [Homo sapiens]	3426171.943	3711634.356	4468158.079	4137498.999	4497633.088	4221618.949	4303827.613	3543824.494	3937401.437
ATM	serine-protein kinase ATM [Homo sapiens]	0	0	0	0	0	0	11797.17014	0	4443.22133
ATN1	atrophin-1 [Homo sapiens]	3594.946834	0	12352.9982	5059.785294	12594.9346	11466.65335	7113.571786	0	15584.60433
ATOX1	copper transport protein ATOX1 [Homo sapiens]	9452566.46	9508409.01	12322594.65	7427486.55	11224328.26	10141960.28	6853998.043	4818718.96	9971053.013
ATP11C	probable phospholipid-transporting ATPase IG isoform a [Homo sapiens]	37324.01332	96181.42365	130177.8623	120505.3751	92815.31393	84472.05726	122315.8274	130227.3887	237276.0972
ATP13A1	probable cation-transporting ATPase 13A1 [Homo sapiens]	82088.43893	165445.8045	77459.28451	220413.045	129890.9843	101646.8559	110206.5585	84637.88705	97710.19939
ATP13A3	probable cation-transporting ATPase 13A3 [Homo sapiens]	0	0	0	0	0	0	42782.93915	32591.62401	35645.19629
ATP1A1	sodium/potassium-transporting ATPase subunit alpha-1 isoform a [Homo sapien	2782008.415	3389701.835	3686447.561	3154738.738	3884211.851	3341933.695	3336828.78	2714986.958	3685456.363
ATP1B1	sodium/potassium-transporting ATPase subunit beta-1 [Homo sapiens]	1616656.658	2860631.891	2662911.629	2853618.49	952643.5949	1862867.955	1973495.177	2589814.584	2932902.838
ATP1B3	sodium/potassium-transporting ATPase subunit beta-3 [Homo sapiens]	6388812.007	7696432.394	8348851.391	8194874.874	9076034.297	8014721.483	6958412.258	5780993.737	7616176.55
ATP2A2	sarcoplasmic/endoplasmic reticulum calcium ATPase 2 isoform b [Homo sapiens	1974053.172	2374717.14	2345136.783	2343700.163	2741707.155	2466397.117	2272653.876	2067396.568	2630987.18
ATP2B1	plasma membrane calcium-transporting ATPase 1 isoform 1b [Homo sapiens]	186236.8114	223765.9306	180952.3402	226586.0769	282148.0454	186841.4015	236719.186	193521.4366	234133.4679
ATP2B4	plasma membrane calcium-transporting ATPase 4 isoform 4b [Homo sapiens]	87141.01136	49328.28173	139638.7103	43480.85439	100855.2892	38648.24677	66588.50795	45610.41705	23451.3182
ATP2C1	calcium-transporting ATPase type 2C member 1 isoform 2a [Homo sapiens]	171319.67	172883.9833	181209.6636	148962.9427	216942.5917	606190.091	518939.4259	94798.41423	119590.8043
ATP5A1	ATP synthase subunit alpha, mitochondrial isoform a precursor [Homo sapiens	11732649.88	13285376.53	15690570.65	12776825.07	16554029.38	13718462.77	13762682.97	10988318.98	14243168.88
ATP5B	ATP synthase subunit beta, mitochondrial precursor [Homo sapiens]	19965703.49	20596080.66	27117559.86	22160592.11	27137915.01	20612443.58	21394482.81	17324181.87	23166030.95
ATP5C1	ATP synthase subunit gamma, mitochondrial isoform L (liver) precursor [Homo	4784520.097	3528810.44	6345369.608	5471149.852	6192213.033	5308300.065	5477027.865	4924468.122	5061685.61
ATP5D	ATP synthase subunit delta, mitochondrial precursor [Homo sapiens]	9902096.967	9795346.998	12862431.23	8774518.611	14210023.01	8822358.182	8977092.751	9761669.076	12482030.52
ATP5E	ATP synthase subunit epsilon, mitochondrial [Homo sapiens]	0	0	0	0	0	0	77636.22581	0	42338.82561
ATP5F1	ATP synthase subunit b, mitochondrial precursor [Homo sapiens]	6310875.959	6229000.355	7563411.088	6992715.437	5173503.173	6299109.673	6034429.248	4562844.315	5836283.93
ATP5H	ATP synthase subunit d, mitochondrial isoform a [Homo sapiens]	10189341.67	8577839.937	9483591.577	9102115.889	9011015.214	7959801.668	7790020.568	10023370.14	7236935.362
ATP5I	ATP synthase subunit e, mitochondrial [Homo sapiens]	7273896.316	9351629.745	10218223.86	8971440.921	9435542.955	10269271.23	8138089.689	7823546.297	11209685.47
ATP5J	ATP synthase-coupling factor 6, mitochondrial isoform b precursor [Homo sap	6882121.025	7091375.25	6019446.841	10223304.82	5482262.361	10277501.12	8534960.375	9053573.086	10905288.08
ATP5J2	ATP synthase subunit f, mitochondrial isoform 2a [Homo sapiens]	6128845.366	6845625.695	7549993.024	6879733.841	8508140.341	6781958.051	6961060.39	0	7661718.488
ATP5J2-PTCD1	ATP5J2-PTCD1 fusion protein [Homo sapiens]	131782.9381	66965.73295	105329.3238	117750.2778	112501.3746	96305.36946	105694.9794	1227778.156	62530.06349
ATP5L	ATP synthase subunit g, mitochondrial [Homo sapiens]	10780575.63	21458599.96	8935104.264	21866502.76	10527128.35	23826348	21808526.19	0	13745294.62
ATP5O	ATP synthase subunit O, mitochondrial precursor [Homo sapiens]	7642132.757	8862539.397	10500756.37	8906776.569	10924651.23	8393723.344	9305132.796	7289744.641	10418715.29
ATP5SL	ATP synthase subunit s-like protein isoform 1 [Homo sapiens]	296993.5273	0	379294.6626	381302.7718	370788.835	0	0	117069.3859	316608.2816
ATP6	ATP synthase F0 subunit 6 [Homo sapiens]	9787612.667	11656567.97	12326804.53	11498568.88	16905117.22	11193182.23	0	9990214.508	13511411.03
ATP6AP1	V-type proton ATPase subunit S1 precursor [Homo sapiens]	562839.7411	710767.5498	642782.0225	654609.4387	891299.532	598558.8389	368814.1066	465963.8457	349515.826
ATP6AP2	renin receptor precursor [Homo sapiens]	723615.697	717769.7706	814055.378	647803.7144	1063880.564	707575.6773	550269.8886	552623.625	607804.0205
ATP6V0A1	V-type proton ATPase 116 kDa subunit a isoform 1 isoform a [Homo sapiens]	117860.866	115095.7161	84266.673	82689.80863	113841.001	83537.66065	121859.993	79446.73529	102387.6546
ATP6V0A2	V-type proton ATPase 116 kDa subunit a isoform 2 [Homo sapiens]	69952.50895	109748.64	87539.12211	78665.16015	118368.2182	68006.13273	89980.81593	65982.70847	102055.0491
ATP6V0C	V-type proton ATPase 16 kDa proteolipid subunit [Homo sapiens]	1941063.537	0	5564517.122	4288410.833	3059367.829	5034616.778	5908546.096	3182198.759	4597474.704
ATP6V0D1	V-type proton ATPase subunit d 1 [Homo sapiens]	1262588.96	1230204.902	1811978.123	1302040.729	1661633.851	1527070.048	1439429.239	1329600.633	1108068.831
ATP6V1A	V-type proton ATPase catalytic subunit A [Homo sapiens]	3135057.064	3773859.602	4460914.922	3624963.877	3980398.39	3706265.048	3677882.837	3041442.518	3809572.453
ATP6V1B2	V-type proton ATPase subunit B, brain isoform [Homo sapiens]	725823.3918	804548.1511	1013858.355	868249.3659	854610.1835	919593.1401	1086186.747	752055.2297	771836.2786
ATP6V1C1	V-type proton ATPase subunit C 1 [Homo sapiens]	758695.2474	788820.0156	1057560.661	892103.1335	1113166.325	943432.3295	744781.5867	612999.8711	952124.6538
ATP6V1D	V-type proton ATPase subunit D [Homo sapiens]	1514807.408	1845022.334	675303.2954	1668716.487	552291.5746	752500.2276	744859.592	563834.0827	533415.8455
ATP6V1E1	V-type proton ATPase subunit E 1 isoform a [Homo sapiens]	372755.658	1493508.595	724730.1157	580868.4564	822225.4052	868518.2552	517265.7896	593891.0722	611083.5652
ATP6V1F	V-type proton ATPase subunit F isoform 2 [Homo sapiens]	1386124.846	1759424.513	1745947.07	1668655.041	1430185.528	1635400.473	1859626.05	1430722.755	1450607.138
ATP6V1G1	V-type proton ATPase subunit G 1 [Homo sapiens]	2178017.838	3023936.924	3116374.824	2529882.017	3290561.603	1774475.324	2045212.274	2167411.891	3012805.198
ATP6V1H	V-type proton ATPase subunit H isoform 1 [Homo sapiens]	220995.2956	452759.5581	364912.7557	212972.4978	309053.8799	382196.5197	164880.7428	242753.5789	356948.9583
ATPAF1	ATP synthase mitochondrial F1 complex assembly factor 1 isoform 1 precursor	543199.0102	523458.7896	273033.3741	680119.7821	562054.9031	668584.3413	510293.2084	396549.2021	540104.4315
ATPAF2	ATP synthase mitochondrial F1 complex assembly factor 2 [Homo sapiens]	567232.4645	738902.9487	715338.2331	319629.7319	763305.5976	654755.0131	615762.5822	515109.2917	706628.8958
ATR	serine/threonine-protein kinase ATR [Homo sapiens]	29461.46011	20622.29286	40668.78106	28285.86845	35907.33881	193288.6524	23706.71126	23901.16338	182549.6413
ATRIP	ATR-interacting protein isoform 1 [Homo sapiens]	32298.13688	67345.81608	528005.8973	370244.9382	0	696666.6008	0	0	0
ATRX	transcriptional regulator ATRX isoform 1 [Homo sapiens]	45925.77813	60726.49556	20768.77698	28069.09035	50202.62078	17051.59486	37750.92614	19688.42806	50874.99479
ATXN10	ataxin-10 isoform 1 [Homo sapiens]	2122960.141	2166963.745	2756401.84	2015772.255	2934757.048	2073445.393	2031108.178	1677097.567	2171800.076
ATXN2	ataxin-2 [Homo sapiens]	157735.9423	140701.8717	127156.4265	169958.7868	156934.9439	144636.3261	146270.0862	112975.1426	157558.7046
ATXN2L	ataxin-2-like protein isoform C [Homo sapiens]	320300.8573	418396.553	0	370797.9715	0	361861.9078	323225.0043	284783.8988	397947.226
ATXN2L	ataxin-2-like protein isoform A [Homo sapiens]	320300.8573	418396.553	0	370797.9715	0	361861.9078	323225.0043	284783.8988	397947.226
AUP1	ancient ubiquitous protein 1 [Homo sapiens]	639458.8483	595347.6656	772291.7225	615577.4518	894795.0697	720251.1408	681808.2432	562480.0845	724412.2634
AURKA	aurora kinase A [Homo sapiens]	213769.8867	160319.7196	276246.0405	212539.1411	180199.2293	250910.7222	377869.4638	683289.3175	110146.2298
AURKB	aurora kinase B isoform 1 [Homo sapiens]	300440.5282	197119.5892	217576.5593	415025.4438	191156.5154	120567.6576	179783.7702	66885.92306	87957.18471
AVEN	cell death regulator Aven [Homo sapiens]	121414.5488	287796.7047	159902.3542	98519.24616	131148.637	130177.4212	185489.2586	153795.2654	0
AVL9	late secretory pathway protein AVL9 homolog [Homo sapiens]	0	0	0	0	0	0	35353.23089	28940.44842	0
AXL	tyrosine-protein kinase receptor UFO isoform 1 precursor [Homo sapiens]	75123.16198	108331.7803	99604.98419	30285.17011	50772.77101	81253.26024	45354.34601	33006.27218	94967.25859
B2M	beta-2-microglobulin precursor [Homo sapiens]	90509.0535	9968695.637	271974.7746	0	267954.5522	0	0	0	0
B3GALT6	beta-1,3-galactosyltransferase 6 [Homo sapiens]	0	0	0	0	0	0	89121.35274	125719.3314	0
B3GAT3	galactosylgalactosylxylosylprotein 3-beta-glucuronosyltransferase 3 [Homo s	0	306062.8819	333541.6772	294452.9685	391477.7461	345025.5739	0	0	0
B3GNT1	N-acetyllactosaminide beta-1,3-N-acetylglucosaminyltransferase [Homo sapien	0	0	0	0	0	0	110804.9926	68094.72353	0
B4GALT1	beta-1,4-galactosyltransferase 1 [Homo sapiens]	627105.404	1233149.258	1377416.466	1496942.206	1506106.819	1450057.901	1223220.95	1098043.998	1117711.907
B4GALT3	beta-1,4-galactosyltransferase 3 [Homo sapiens]	120290.0188	27256.31849	137222.3887	76205.33562	125980.0103	71372.77705	149237.2886	77476.62274	100022.8853
BABAM1	BRISC and BRCA1-A complex member 1 [Homo sapiens]	307597.4551	289731.2998	251555	313691.9025	240897.5875	306046.4433	271136.7372	68855.87377	516798.9568
BAG1	BAG family molecular chaperone regulator 1 isoform BAG-1L [Homo sapiens]	33094.5777	311410.8193	651161.897	317570.7432	425832.6951	483360.9531	282603.6634	297649.9579	277781.8539
BAG2	BAG family molecular chaperone regulator 2 [Homo sapiens]	7552445.771	7942841.682	10505078.61	8004112.359	10801188.9	7632597.342	8446217.942	7081487.656	8954084.323
BAG3	BAG family molecular chaperone regulator 3 [Homo sapiens]	664359.3332	788969.9283	936610.1478	938624.98	1029054.716	979469.1054	817451.5883	571797.4576	841785.5385

BAG4	BAG family molecular chaperone regulator 4 isoform 1 [Homo sapiens]	106766.6574	57413.52778	128473.0046	78969.86111	101142.3438	83530.36921	0	48241.648	79785.06134
BAG5	BAG family molecular chaperone regulator 5 isoform a [Homo sapiens]	237370.1205	257787.9915	322297.082	198218.3672	302242.3556	179056.0528	287011.8918	193273.3069	233259.971
BAG6	large proline-rich protein BAG6 isoform a [Homo sapiens]	1110876.081	1236367.585	1576266.049	1325717.062	1615486.983	1380439.833	1070718.477	849573.3353	1026865.604
BAIAP2	brain-specific angiogenesis inhibitor 1-associated protein 2 isoform 2 [Hor	174322.0129	395568.7135	254438.8893	432588.7021	289970.9302	467245.3473	430962.2344	195552.6349	152928.4308
BAIAP2L1	brain-specific angiogenesis inhibitor 1-associated protein 2-like protein 1	141390.3022	120189.6667	185952.3359	170431.6847	143558.5755	166491.2879	121874.8153	124818.4334	206967.7387
BAK1	bcl-2 homologous antagonist/killer [Homo sapiens]	0	925267.3679	451498.9364	301819.934	427209.3774	947105.4434	254515.23	0	1086490.17
BANF1	barrier-to-autointegration factor [Homo sapiens]	44892352.85	44131207.96	54240584.55	44904178.81	56493558.89	47619760.09	46620539.79	44556400.23	47004093.3
BASP1	brain acid soluble protein 1 [Homo sapiens]	36862547.23	34128764.78	55259616.35	36372065.77	59215370.3	37536614.43	32029440.05	20373483.43	44663892.6
BAX	apoptosis regulator BAX isoform beta [Homo sapiens]	2210484.671	2945720.024	5032753.51	2916456.472	3591040.302	2861368.603	2806252.567	2376584.967	3034907.238
BAZ1A	bromodomain adjacent to zinc finger domain protein 1A isoform a [Homo sapie	150573.8139	158383.0963	91035.4705	93227.87613	96243.50716	116491.8361	120275.9312	113192.4528	142113.7677
BAZ1B	tyrosine-protein kinase BAZ1B [Homo sapiens]	190846.5715	210177.4693	245503.0851	227088.5046	246014.1524	241457.9674	228922.9342	185060.362	508927.825
BAZ2A	bromodomain adjacent to zinc finger domain protein 2A [Homo sapiens]	50600.16214	49709.9112	36234.64983	51409.89699	135251.2294	31161.5655	43258.29081	20907.88842	63393.64223
BBX	HMG box transcription factor BBX isoform 1 [Homo sapiens]	0	0	0	0	0	0	32969.82452	26664.74591	0
BCAP31	B-cell receptor-associated protein 31 isoform a [Homo sapiens]	3157206.988	3211887.746	2831339.783	3281349.852	3231481.612	2973192.864	3263614.872	2586155.588	2330335.519
BCAR1	breast cancer anti-estrogen resistance protein 1 isoform 1 [Homo sapiens]	255492.0611	188681.327	323366.9874	188307.2362	343809.3587	241617.9775	232615.5921	205385.9682	187193.3427
BCAR3	breast cancer anti-estrogen resistance protein 3 isoform 1 [Homo sapiens]	59401.62704	79757.23856	72241.42049	70734.89595	81897.32101	139950.1092	119276.379	109422.292	121052.6093
BCAS2	pre-mRNA-splicing factor SPF27 [Homo sapiens]	4212426.233	4620714.71	5298399.349	4605125.69	5475555.376	5178008.778	4555662.728	3484918.194	4765644.852
BCAT2	branched-chain-amino-acid aminotransferase, mitochondrial isoform a precurs	706270.1435	1007904.595	707424.2068	989275.3156	788322.517	797470.066	1057778.741	574049.3905	1059546.414
BCCIP	BRCA2 and CDKN1A-interacting protein isoform BCCIPbeta [Homo sapiens]	2524635.427	2273951.598	3519583.784	2823182.619	3347951.885	2942630.673	2794776.593	2404647.052	2466705.41
BCKDHA	2-oxoisovalerate dehydrogenase subunit alpha, mitochondrial isoform 1 precu	58537.51591	142154.02	0	64627.75941	408006.2301	125455.0533	55709.97485	50044.54586	0
BCKDHB	2-oxoisovalerate dehydrogenase subunit beta, mitochondrial precursor [Homo	128637.4803	274490.0396	226340.5438	176071.8993	225652.998	122433.6424	198629.4385	100867.2668	291304.5888
BCL10	B-cell lymphoma/leukemia 10 [Homo sapiens]	202145.7245	0	1093891.291	640543.7755	0	569897.5918	0	149585.6327	401446.6633
BCL2L1	bcl-2-like protein 1 isoform 1 [Homo sapiens]	0	0	0	0	0	0	297457.0795	0	368938.0748
BCL2L12	bcl-2-like protein 12 isoform 1 [Homo sapiens]	313343.5694	0	0	74582.93319	613178.5486	85372.10417	0	0	0
BCL2L13	bcl-2-like protein 13 isoform a [Homo sapiens]	1010078.022	1103175.398	1355116.615	1056192.359	1340968.133	1093436.941	965553.2462	1057370.906	980642.5966
BCL7A	B-cell CLL/lymphoma 7 protein family member A isoform a [Homo sapiens]	72842.23911	0	73098.27739	59034.67925	96683.58743	59000.20821	43475.58602	49391.15967	0
BCL7C	B-cell CLL/lymphoma 7 protein family member C [Homo sapiens]	178082.8503	0	290388.1715	0	359094.3663	222801.8576	218183.4041	157626.1003	131852.7413
BCL9L	B-cell CLL/lymphoma 9-like protein [Homo sapiens]	79694.11295	89779.73813	74311.37741	87580.15601	0	76986.51826	49663.25069	91625.57231	79930.3422
BCLAF1	bcl-2-associated transcription factor 1 isoform 2 [Homo sapiens]	79979.66574	48751.23269	106883.3537	346061.8269	367738.8294	398888.8063	323664.4394	177231.0707	401694.0901
BCLAF1	bcl-2-associated transcription factor 1 isoform 1 [Homo sapiens]	79979.66574	48751.23269	106883.3537	346061.8269	367738.8294	398888.8063	323664.4394	177231.0707	401694.0901
BCR	breakpoint cluster region protein isoform 1 [Homo sapiens]	32386.00812	60087.37349	64056.68928	50636.41118	55320.54839	43883.97679	49806.8856	35115.14833	39161.10009
BCS1L	mitochondrial chaperone BCS1 [Homo sapiens]	260467.8769	126822.9992	608176.6198	223499.4638	335810.5567	287323.4339	318325.1165	281182.9578	192799.7022
BDH1	D-beta-hydroxybutyrate dehydrogenase, mitochondrial precursor [Homo sapiens	0	112539.9643	319770.8268	106038.6946	0	88917.86207	39306.96161	0	94001.75714
BEND3	BEN domain-containing protein 3 [Homo sapiens]	0	0	0	0	0	0	55175.09943	0	20714.51133
BET1	BET1 homolog [Homo sapiens]	1481965.927	2773654.31	2151572.745	1643598.923	672652.744	3193697.579	3185247.274	2522937.155	3373309.593
BET1L	BET1-like protein isoform 1 [Homo sapiens]	317976.4804	0	543295.6275	443084.7353	0	509631.0957	431019.8627	342114.3465	0
BICC1	protein bicaudal C homolog 1 [Homo sapiens]	44192.04322	34714.20383	49444.19365	73965.2504	101387.7642	68073.49426	123222.4748	0	35303.49107
BICD2	protein bicaudal D homolog 2 isoform 1 [Homo sapiens]	86168.04652	114716.145	83204.41268	114411.3215	52409.34407	190956.1113	117819.7957	47794.94566	48119.07879
BID	BH3-interacting domain death agonist isoform 1 [Homo sapiens]	2442556.115	2182534.395	3199733.536	2515715.312	3193690.532	2693364.948	2342662.293	2126235.519	2150138.974
BIN1	myc box-dependent-interacting protein 1 isoform 1 [Homo sapiens]	713448.6369	731320.1456	1028603.706	778696.1092	980680.8533	804749.5682	755548.8503	719825.9718	708526.9713
BIRC6	baculoviral IAP repeat-containing protein 6 [Homo sapiens]	21729.70352	31116.17851	30938.20505	22101.32867	31967.71748	35583.55239	31269.14603	23993.70108	25531.25746
BIVM-ERCC5	BIVM-ERCC5 protein [Homo sapiens]	9156.138761	6185.732658	14626.23391	13998.98764	19032.37196	8021.520359	9734.677002	22618.20722	0
BLM	Bloom syndrome protein [Homo sapiens]	151005.6278	135387.529	98363.5224	157873.218	91330.42828	143316.2953	209868.9526	144478.2517	126481.8957
BLMH	bleomycin hydrolase [Homo sapiens]	2674961.612	2799363.692	3415845.379	2987005.318	3675345.988	3137057.039	2896810.713	2726078.024	3003909.147
BLOC1S1	biogenesis of lysosome-related organelles complex 1 subunit 1 [Homo sapiens	1113574.705	537687.4186	1184292.659	764551.303	870833.5176	0	0	0	0
BLOC1S2	biogenesis of lysosome-related organelles complex 1 subunit 2 [Homo sapiens	1323246.319	1254420.64	1347825.205	1411304.911	1243328.833	1681235.62	1863397.974	1495811.57	1155376.631
BLOC1S3	biogenesis of lysosome-related organelles complex 1 subunit 3 [Homo sapiens	261530.3505	124572.0862	411680.0491	421465.2959	651153.8291	0	0	0	0
BLVRA	biliverdin reductase A precursor [Homo sapiens]	2460256.565	2477220.224	3260558.752	2459303.602	3137534.234	2725230.376	2401212.594	2144292.368	2701522.332
BLVRB	flavin reductase (NADPH) [Homo sapiens]	8993852.851	9762245.306	11472749.96	7449476.763	11277799.31	10547474.03	9080406.697	8025890.937	10947305.45
BMP2K	BMP-2-inducible protein kinase isoform a [Homo sapiens]	0	0	0	0	0	0	9659.556647	0	12043.95308
BMS1	ribosome biogenesis protein BMS1 homolog [Homo sapiens]	155123.0975	174093.6989	190732.6002	152117.2849	191717.1309	188863.5192	151973.6492	111879.6158	201478.1601
BNIP1	vesicle transport protein SEC20 isoform BNIP1-b [Homo sapiens]	92823.40948	326587.9617	221563.5948	232313.3836	252918.2694	219496.5221	343992.9871	29188.81008	186245.6741
BNIP2	BCL2/adenovirus E1B 19 kDa protein-interacting protein 2 [Homo sapiens]	83198.60312	162953.8079	138033.744	172438.3981	231753.829	155529.861	116836.3847	100560.7961	224074.3041
BOD1L1	biorientation of chromosomes in cell division protein 1-like 1 [Homo sapier	15250.08007	10169.99208	19861.71057	16056.0165	22885.91636	18567.12918	19243.38042	6280.078229	14058.96362
BOLA2B	bolA-like protein 2 [Homo sapiens]	15773734.55	12943806.39	16181034.8	16656538.83	18668686.46	11307117.56	14533742.95	11008125.37	17158169.23
BOLA3	bolA-like protein 3 isoform 1 [Homo sapiens]	1948586.913	2047373.944	1896026.7	1766685.815	2125323.53	1745650.597	1944938.765	1192360.389	2071026.944
BOP1	ribosome biogenesis protein BOP1 [Homo sapiens]	862322.62	768806.6446	1047478.547	719941.2772	1184238.133	1059306.449	903905.0232	610833.0242	1002246.122
BPHL	valacyclovir hydrolase precursor [Homo sapiens]	157308.1924	270635.918	210741.4597	146572.2734	0	0	177169.5977	129688.6602	264658.4602
BPIFB6	BPI fold-containing family B member 6 precursor [Homo sapiens]	0	0	0	0	0	0	1723997.086	1331014.72	0
BPNT1	3'(2'),5'-bisphosphate nucleotidase 1 [Homo sapiens]	1461306.184	1792556.289	1810628.5	2853729.73	2315908.195	1805215.012	1572306.365	1481136.383	1922225.741
BPTF	nucleosome-remodeling factor subunit BPTF isoform 1 [Homo sapiens]	22536.49566	54131.74665	26135.81617	22534.95434	49718.96831	17566.94334	25056.57227	19713.3828	10605.25264
BRAP	BRCA1-associated protein [Homo sapiens]	108732.8845	264668.6865	177501.3129	171996.6946	233526.5762	295676.0348	298713.0149	151941.4212	159351.6786
BRAT1	BRCA1-associated ATM activator 1 [Homo sapiens]	237897.0754	244393.4248	295814.1725	277416.525	280711.5847	290910.9607	257875.5693	221043.8895	256998.69
BRCC3	lys-63-specific deubiquitinase BRCC36 isoform 1 [Homo sapiens]	358213.4521	382451.4809	454474.1987	319829.0323	476952.6129	385045.7761	330612.8156	122607.7214	320919.5588
BRD2	bromodomain-containing protein 2 isoform 2 [Homo sapiens]	177402.0543	114523.6436	275233.2734	220048.602	315561.0668	236552.7498	138831.3155	146089.5591	236926.8391
BRD4	bromodomain-containing protein 4 isoform long [Homo sapiens]	126124.7465	132005.9626	222400.0613	154492.6247	228878.7559	145012.7925	120912.264	82653.88322	136498.1737
BRD8	bromodomain-containing protein 8 isoform 2 [Homo sapiens]	50962.31823	56282.43449	36735.08435	33802.61207	0	0	0	17273.36223	40818.45161
BRE	BRCA1-A complex subunit BRE isoform 1 [Homo sapiens]	0	0	0	0	0	0	272041.2261	450697.2543	0
BRE	BRCA1-A complex subunit BRE isoform 2 [Homo sapiens]	0	0	0	0	0	0	272041.2261	450697.2543	0
BRI3BP	BRI3-binding protein precursor [Homo sapiens]	1322676.526	1775010.486	916241.4881	1218139.665	1061992.728	1459575.019	1625605.46	1429340.165	1122225.293
BRIX1	ribosome biogenesis protein BRX1 homolog [Homo sapiens]	11128203.28	9708901.553	14141929.54	10094832.94	14139306.77	12026690.83	10178343.55	9719756.827	11038692.31
BRK1	protein BRICK1 [Homo sapiens]	0	0	0	0	0	0	5045563.1	0	5678568.17
BROX	BRO1 domain-containing protein BROX [Homo sapiens]	669831.4913	271473.0805	825314.9581	672073.1517	874408.1419	716133.2122	618002.4686	479664.75	337431.157
BRWD1	bromodomain and WD repeat-containing protein 1 isoform A [Homo sapiens]	16688.9039	0	13853.50833	12209.49412	0	21347.65034	0	0	0
BSDC1	BSD domain-containing protein 1 isoform a [Homo sapiens]	173600.6821	262223.8857	168507.3509	240821.9625	173219.3031	310279.0386	249757.15	249173.7821	229540.1018
BSG	basigin isoform 1 precursor [Homo sapiens]	3113395.515	3616494.047	2824730.45	4137676.402	2827334.345	2698190.397	1917336.587	4386228.368	3950751.999
BSG	basigin isoform 3 [Homo sapiens]	3113395.515	3616494.047	2824730.45	4137676.402	2827334.345	2698190.397	1917336.587	4386228.368	3950751.999
BST2	bone marrow stromal antigen 2 [Homo sapiens]	1340223.022	3844525.398	2099193.664	1329642.065	2153074.281	1479076.633	1200523.168	1019207.285	1259110.344
BTAF1	TATA-binding protein-associated factor 172 [Homo sapiens]	33468.38347	40597.12892	57375.12216	33699.65493	50981.80582	39037.21944	17579.18831	49980.6264	59970.49166
BTF3	transcription factor BTF3 isoform A [Homo sapiens]	23852781.47	27347553.52	30117197.4	30110090.8	28561799.68	31063443.84	27516476.88	24255224.48	26716239.61
BTF3L4	transcription factor BTF3 homolog 4 isoform 1 [Homo sapiens]	2490382.798	2075552.229	2525476.153	2836028.255	2829703.106	2139523.912	1402228.41	2882157.214	2307928.85
BTN3A2	butyrophilin subfamily 3 member A2 isoform a precursor [Homo sapiens]	81852.93693	0	124209.6418	97901.3287	0	101222.2287	0	73560.97744	39186.70674
BUB1	mitotic checkpoint serine/threonine-protein kinase BUB1 [Homo sapiens]	22977.19187	37024.03776	35348.95549	31885.25858	201578.7358	21719.52968	17434.58608	0	16758.09029
BUB1B	mitotic checkpoint serine/threonine-protein kinase BUB1 beta [Homo sapiens]	116737.8135	116268.7793	140456.1709	129460.9394	134385.7022	149198.1945	81274.36778	58592.09036	70061.92954
BUB3	mitotic checkpoint protein BUB3 isoform a [Homo sapiens]	4137520.399	4827949.308	6918545.228	4407737.631	6320197.256	4395664.588	5190372.534	4294462.524	5660748.432
BUD13	BUD13 homolog isoform 1 [Homo sapiens]	16283.92841	67247.13289	76684.76504	84208.28923	17219.96203	89901.80301	83785.50143	0	85016.65312
BUD31	protein BUD31 homolog [Homo sapiens]	686636.3892	849610.1687	982724.1409	837892.3381	935844.519	1067664.284	1067540.55	1080451.306	562184.6801
BYSL	bystin [Homo sapiens]	613109.2273	689806.0758	869646.3825	751359.682	893825.3675	748129.4139	747109.2175	648326.1892	784597.8232
BZRAP1	peripheral-type benzodiazepine receptor-associated protein 1 isoform a [Hor	0	0	0	0	0	0	98494.16439	216010.558	0
BZW1	basic leucine zipper and W2 domain-containing protein 1 isoform 2 [Homo sap	4497343.587	8433511.119	11436475.52	5069067.035	11156113.32	8825978.119	5486984.845	5175414.156	8855347.332
BZW2	basic leucine zipper and W2 domain-containing protein 2 [Homo sapiens]	3006718.488	2984937.634	3410096.261	3320646.51	3467126.262	3378933.671	3251498.699	2898072.042	3329568.574
C1orf137	erythroid differentiation-related factor 1 isoform 1 [Homo sapiens]	61779.02278	23095.42769	24371.02974	17656.85119	26477.5804	24827.91769	12831.55617	17898.89758	80032.91692
C1orf2	twinkle protein, mitochondrial isoform A [Homo sapiens]	38399.60326	60194.93638	86083.06767	17385.39382	53601.9553	48486.58943	42667.22581	57765.07348	28348.35573
C10orf76	UPF0668 protein C10orf76 [Homo sapiens]	0	84161.63527	138296.8108	112851.2759	108255.7044	113292.3767	88270.32432	57061.88401	106699.83
C11orf30	protein EMSY [Homo sapiens]	0	0	0	0	0	0	29696.44782	0	19876.00956
C11orf31	selenoprotein H [Homo sapiens]	0	0	0	0	0	0	614972.0164	624857.3689	0
C11orf54	ester hydrolase C11orf54 [Homo sapiens]	751207.3504	1642679.939	1007488.503	2884523.345	512104.2722	1269550.792	647765.6096	618715.1622	1792625.689
C11orf58	small acidic protein [Homo sapiens]	3773046.957	5828380.578	2134714.01	3987072.357	5378038.035	4042017.263	4070536.771	5454744.983	3953971.143
C11orf68	UPF0696 protein C11orf68 isoform 1 [Homo sapiens]	320417.5864	287860.0399	499703.8294	434809.1937	604201.8831	494186.3456	386358.091	269693.7847	269822.2181
C11orf73	protein Hikeshi [Homo sapiens]	2396332.15	8508650.795	3354464.183	2071998.185	3415515.305	2300239.843	1937688.455	1265402.027	2602173.679
C11orf83	UPF0723 protein C11orf83 precursor [Homo sapiens]	2711245.958	2020784.785	2861950.097	2340366.582	4531991.972	2376604.616	3029008.422	2484550.139	2958157.056
C11orf84	uncharacterized protein C11orf84 [Homo sapiens]	0	0	0	0	0	0	93258.12689	59255.49337	0
C12orf10	UPF0160 protein MYG1, mitochondrial precursor [Homo sapiens]	2345637.988	2086834.675	2822742.074	2687883.788	3159428.39	3004012.388	2737024.593	2341928.991	2775518.319
C12orf23	UPF0444 transmembrane protein C12orf23 [Homo sapiens]	5226336.942	5031918.558	5694802.547	4693771.15	6277602.636	6500603.686	6244311.672	5199589.533	7074974.556
C12orf29	uncharacterized protein C12orf29 [Homo sapiens]	197193.9957	133786.6212	0	85552.71685	434124.7346	214062.8788	123302.8842	0	223815.9838
C12orf43	uncharacterized protein C12orf43 [Homo sapiens]	362138.7056	388766.5066	0	386159.8785	722945.6589	390047.4159	348952.4065	416697.4439	522783.9626
C12orf44	autophagy-related protein 101 [Homo sapiens]	260272.1903	86412.68333	233393.8472	92240.66389	276436.0709	0	0	69198.6125	240520.3487
C12orf45	uncharacterized protein C12orf45 [Homo sapiens]	367859.4103	0	274799.0513	203822.0149	714029.6026	147096.3574	0	0	0
C12orf5	fructose-2,6-bisphosphatase TIGAR [Homo sapiens]	1270218.157	1438291.503	1590923.534	1418512.804	1557868.813	1573549.049	1431176.697	1188960.332	1548355.498
C12orf57	protein C10 [Homo sapiens]	2024176.69	2204840.837	2012556.752	1481407.691	2498118.394	1936906.128	1809733.104	985809.4324	2004041.221
C14orf142	uncharacterized protein C14orf142 [Homo sapiens]	2099554.401	2444653.664	1635605.81	3527749.186	1594647.922	820820.7586	1800292.75	2504850.136	2159887.379
C14orf166	UPF0568 protein C14orf166 [Homo sapiens]	4967985.644	5376752.632	6374709.26	5437638.429	6325826.584	5862381.386	5397003.014	4653454.99	5413360.645
C14orf169	bifunctional lysine-specific demethylase and histidyl-hydroxylase NO66 [Hor	125208.2419	86930.5	121529.3823	138143.2175	77715.78457	155437.0825	0	123461.6569	125995.9924
C14orf2	6.8 kDa mitochondrial proteolipid isoform 1 [Homo sapiens]	0	0	0	0	0	0	9510323.111	0	2340064.412
C15orf38-AP3S2	C15orf38-AP3S2 fusion protein [Homo sapiens]	586162.2234	511850.3576	688182.5434	661876.8678	796615.7771	677659.8614	600109.3819	443406.9456	423746.5365
C15orf40	UPF0235 protein C15orf40 isoform 1 [Homo sapiens]	0	0	0	0	0	0	97870.43116	226485.1884	0
C16orf13	UPF0585 protein C16orf13 isoform b [Homo sapiens]	151999.8142	103180.2298	271953.6252	232488.2533	289845.3535	223918.7273	64792.06155	0	259012.4194
C16orf13	UPF0585 protein C16orf13 isoform a [Homo sapiens]	151999.8142	103180.2298	271953.6252	232488.2533	289845.3535	223918.7273	64792.06155	0	259012.4194
C16orf62	UPF0505 protein C16orf62 [Homo sapiens]	20991.36262	0	599875.2362	41754.88043	19110.6632	38679.84935	45813.87077	30557.41727	49874.21502
C16orf80	UPF0468 protein C16orf80 [Homo sapiens]	779634.9796	824783.3365	696080.2766	623018.1977	2724043.151	777205.7048	611916.1068	1809558.49	2882805.56

C16orf87	UPF0547 protein C16orf87 [Homo sapiens]	28228.81601	50242.44944	54047.32269	0	0	40843.89747	30294.47612	0	33722.60674
C16orf91	protein CCSMST1 precursor [Homo sapiens]	0	0	0	0	0	0	0	0	0
C17orf49	chromatin complexes subunit BAP18 isoform 1 [Homo sapiens]	0	846648.8192	310848.2462	950980.7731	0	241205.4135	0	0	0
C17orf59	uncharacterized protein C17orf59 [Homo sapiens]	71690.09033	75720.54263	156403.9988	0	170792.4896	67410.74635	0	89038.38686	79961.31752
C17orf70	Fanconi anemia-associated protein of 100 kDa isoform b [Homo sapiens]	15579.9963	15914.93361	24356.60918	14205.08027	10225.64108	22287.18387	23261.37521	10402.87707	77655.06797
C17orf75	protein Njmu-R1 [Homo sapiens]	227608.3134	220222.1712	205679.7209	164207.9587	292390.8048	167298.2277	130852.542	122920.4101	245094.1408
C17orf85	uncharacterized protein C17orf85 [Homo sapiens]	42212.2334	80899.48573	263467.3822	276463.1971	249828.9917	139785.6949	204530.4839	26655.03542	177615.0231
C18orf25	uncharacterized protein C18orf25 isoform b [Homo sapiens]	0	41403.53366	54607.3602	47185.92834	106875.0363	43389.9797	0	272802.4436	154406.8428
C18orf25	uncharacterized protein C18orf25 isoform a [Homo sapiens]	0	41403.53366	54607.3602	47185.92834	106875.0363	43389.9797	0	272802.4436	154406.8428
C19orf10	UPF0556 protein C19orf10 precursor [Homo sapiens]	7684201.091	4971688.513	7563200.214	6014205.079	8121975.979	6446370.014	6750330.625	5085631.127	7222391.039
C19orf25	UPF0449 protein C19orf25 [Homo sapiens]	1124462.318	991303.8409	0	549097.5473	488444.6705	942943.4234	564463.5682	836655.9191	0
C19orf33	immortalization up-regulated protein [Homo sapiens]	978209.1284	30567528.97	37758849.26	31171962.11	35826259.79	43588517.05	28913297.68	33447909.03	44855285.47
C19orf43	uncharacterized protein C19orf43 [Homo sapiens]	648812.6239	751920.9453	1453137.907	460902.4588	1230785.096	936060.3253	566724.3286	203798.3283	1019294.937
C19orf52	uncharacterized protein C19orf52 [Homo sapiens]	590633.7441	660586.3289	108205.6762	143862.6183	0	455516.0906	338567.4989	0	344610.1201
C19orf70	protein QIL1 precursor [Homo sapiens]	3363609.09	2116865.515	4593676.138	2033751.258	4351257.733	2855639.333	3186101.628	2748299.574	2576401.303
C1orf112	uncharacterized protein C1orf112 [Homo sapiens]	0	0	0	0	0	0	33266.60351	42700.84934	47819.54694
C1orf122	uncharacterized protein C1orf122 isoform 1 [Homo sapiens]	0	1720437.106	0	380175.5144	1291197.841	1462906.452	1656487.279	1289779.799	1690772.894
C1orf123	UPF0587 protein C1orf123 [Homo sapiens]	239851.9242	1492083.25	1636711.476	0	1562049.78	0	849137.8333	0	956783.3561
C1orf27	protein odr-4 homolog isoform 1 [Homo sapiens]	260123.2736	90000.86596	295238.533	188246.0125	224675.5736	204125.8223	244030.8978	175971.9505	1028577.73
C1orf35	multiple myeloma tumor-associated protein 2 [Homo sapiens]	0	160107.4779	261336.0294	129963.0427	209097.1554	205575.8015	191681.4173	118743.4963	188076.6875
C1orf50	uncharacterized protein C1orf50 [Homo sapiens]	0	0	0	0	0	0	362032.1348	348577.875	0
C1orf52	UPF0690 protein C1orf52 [Homo sapiens]	0	109406.6818	1240291.273	139854.5909	0	171289.7958	0	0	0
C1orf68	skin-specific protein 32 [Homo sapiens]	0	0	0	0	0	0	0	0	0
C1orf85	lysosomal protein NCU-G1 isoform 1 precursor [Homo sapiens]	805914.4868	657898.7368	885137.7632	486723.5026	0	922354.5395	0	1268501.398	676276.8816
C1QBP	complement component 1 Q subcomponent-binding protein, mitochondrial precu	40096480.54	45220350.93	56032106.52	45517911.61	54922558.54	49406829.47	49714945.67	44228635.04	52001829.14
C1QTNF6	complement C1q tumor necrosis factor-related protein 6 [Homo sapiens]	0	0	0	0	0	0	0	136083.2474	451399.2751
C20orf112	uncharacterized protein C20orf112 isoform 1 [Homo sapiens]	0	0	0	0	0	0	43842.88617	0	44560.9117
C20orf24	uncharacterized protein C20orf24 isoform b [Homo sapiens]	604661.2814	456949.2343	505521.7102	590933.4432	833609.1932	712899.7955	0	0	0
C20orf27	UPF0687 protein C20orf27 isoform 1 [Homo sapiens]	627744.0699	616164.2819	663433.5584	156272.9078	632792.7668	111573.6485	149626.7365	160289.4943	781058.2508
C21orf33	ES1 protein homolog, mitochondrial isoform Ia precursor [Homo sapiens]	1137183.76	2732748.268	3093866.624	2831374.228	3378165.475	2962340.71	3357334.257	2484645.18	2974036.193
C21orf59	uncharacterized protein C21orf59 [Homo sapiens]	411861.5268	519518.8057	774049.5161	600114.1889	746558.762	578945.6115	520012.8459	503002.9106	337678.854
C2CD2	C2 domain-containing protein 2 isoform 1 precursor [Homo sapiens]	18324.24156	23578.49657	0	22140.17405	22509.02901	63757.32278	70678.17089	18373.95833	33444.19673
C2orf43	UPF0554 protein C2orf43 [Homo sapiens]	251471.1338	168043.4127	301483.2451	249599.9157	322657.4475	215547.5258	210972.1949	195711.689	179763.0209
C2orf47	uncharacterized protein C2orf47, mitochondrial precursor [Homo sapiens]	657942.549	555738.656	601750.6105	733839.8672	732159.907	819197.844	659629.1226	535243.9355	782513.7754
C2orf49	ashwin [Homo sapiens]	133989.2816	348088.7891	201909.3217	387249.5103	193707.9457	176654.1886	85287.97826	0	410181.1962
C3	complement C3 precursor [Homo sapiens]	24359.13524	28417.80337	32051.68944	13883.52128	39271.9042	24010.05368	7999.339169	6776.196183	29405.1626
C3orf17	uncharacterized protein C3orf17 [Homo sapiens]	53614.21536	60871.82397	112268.9979	41303.37536	77391.98408	50723.62438	50008.04262	0	61024.8221
C3orf33	uncharacterized protein C3orf33 [Homo sapiens]	0	0	0	0	0	0	148080.4964	72150.2375	0
C3orf37	UPF0361 protein C3orf37 [Homo sapiens]	357522.1819	344497.8293	380357.3576	370947.2683	512124.4818	239956.2015	216344.4535	298290.5141	1348443.843
C3orf38	uncharacterized protein C3orf38 [Homo sapiens]	0	0	0	0	0	0	0	0	0
C4orf27	UPF0609 protein C4orf27 [Homo sapiens]	197373.3193	65102.63674	175420.9765	195950.3444	191232.0839	145025.8948	122897.957	117351.7824	167202.2033
C4orf32	uncharacterized protein C4orf32 [Homo sapiens]	0	0	0	0	0	0	0	0	0
C5orf15	keratinocyte-associated transmembrane protein 2 precursor [Homo sapiens]	0	1032903.881	1538712.625	716135.4235	1623352.069	0	0	0	0
C5orf22	UPF0489 protein C5orf22 [Homo sapiens]	255942.3126	289268.8026	329051.69	124679.65	316170.3746	325750.696	91545.792	179448.4135	390867.97
C5orf24	UPF0461 protein C5orf24 [Homo sapiens]	0	0	0	0	0	0	74359.09299	0	214846.8052
C5orf28	transmembrane protein C5orf28 [Homo sapiens]	0	0	0	0	0	0	162185.7346	0	401338.6231
C5orf51	UPF0600 protein C5orf51 [Homo sapiens]	877171.2873	1102477.505	1365502.931	1085215.337	1221037.998	1133506.059	1031786.655	847520.154	1133341.781
C6orf211	UPF0364 protein C6orf211 [Homo sapiens]	487379.1047	415913.158	557574.722	162066.5257	579128.5428	458861.5557	256864.8046	401448.2399	371420.8446
C6orf47	uncharacterized protein C6orf47 [Homo sapiens]	0	0	0	0	0	0	237231.1571	0	289623.7672
C6orf89	bombesin receptor-activated protein C6orf89 [Homo sapiens]	0	0	0	0	0	0	68383.44	53549.476	0
C7orf26	uncharacterized protein C7orf26 [Homo sapiens]	145051.25	158109.8939	169668.3485	158344.1932	179896.116	0	93626.61053	112177.0814	146109.2538
C7orf50	uncharacterized protein C7orf50 [Homo sapiens]	682227.9533	768543.2615	892515.9728	692449.067	822060.0221	938027.1695	685394.6772	786986.059	789661.9359
C8orf33	UPF0488 protein C8orf33 [Homo sapiens]	804870.7347	802414.5306	1243726.86	534504.8571	1293085.883	1000545.98	891671.1505	69336.35141	589768.6071
C8orf82	UPF0598 protein C8orf82 [Homo sapiens]	328940.1724	322675.2615	513998.153	331767.8291	542206.1825	446793.4885	300506.6667	272625.0517	353794.5723
C9orf114	uncharacterized protein C9orf114 [Homo sapiens]	539188.6922	251145.8233	573444.5305	407874.5533	578776.0769	415100.2211	423395.3554	520249.5955	436192.5829
C9orf142	uncharacterized protein C9orf142 [Homo sapiens]	459119.2131	212013.1596	459991.806	190119.032	513127.3714	261659.3178	239029.6664	385898.6476	237855.8946
C9orf16	UPF0184 protein C9orf16 [Homo sapiens]	2547180.529	2413730.209	1909867.941	2537535.279	1624553.324	0	0	0	0
C9orf40	uncharacterized protein C9orf40 [Homo sapiens]	717665.7742	0	1011565.657	416837.0806	524570.7661	781841.1935	0	426787.7182	39766.09577
C9orf41	UPF0586 protein C9orf41 [Homo sapiens]	0	0	0	0	0	0	0	42794.0839	83574.61171
C9orf64	UPF0553 protein C9orf64 [Homo sapiens]	239670.7784	337316.4478	319548.3916	336533.7212	334698.2123	433921.5238	428626.7018	270336.6241	546373.0148
C9orf78	uncharacterized protein C9orf78 [Homo sapiens]	680007.9933	799236.6805	813627.9913	553540.1328	2174555.766	631694.7855	216915.6647	517216.0364	543944.2399
CAAP1	caspase activity and apoptosis inhibitor 1 isoform 1 [Homo sapiens]	88995.60687	129499.2634	116391.4905	72183.35603	97065.10938	108781.4063	81760.09375	0	106783.4241
CAB39	calcium-binding protein 39 [Homo sapiens]	736317.638	705654.0981	1516191.951	682391.4259	970678.1437	678859.0367	652697.4747	668960.9778	790085.7228
CACNA2D1	voltage-dependent calcium channel subunit alpha-2/delta-1 precursor [Homo s	25097.33333	47995.09384	28097.69952	33701.9719	46973.18849	40559.17307	47398.13989	27336.09413	57550.79807
CACTIN	cactin [Homo sapiens]	66770.66642	67947.30196	95857.61973	86791.35015	66664.95557	82564.11747	57598.55045	55595.80723	73153.9328
CACYBP	calcyclin-binding protein isoform 1 [Homo sapiens]	9523045.967	11299650.67	28003628.13	11595345.28	30953393.28	11518633.16	10810283.93	21648868.26	11009445.33
CAD	CAD protein [Homo sapiens]	1003881.549	1105363.017	1292363.763	1145209.028	1273066.206	1159206.763	1117260.64	975399.8562	1154210.251
CALCOCO2	calcium-binding and coiled-coil domain-containing protein 2 isoform 1 [Homo	138275.1364	147200.173	33011.05556	163347.2929	0	0	0	0	0
CALD1	caldesmon isoform 1 [Homo sapiens]	250632.973	234192.9952	343323.8538	278692.3484	309772.7311	288743.6674	227877.3989	218540.1043	267298.6572
CALM3	calmodulin [Homo sapiens]	59307468.86	69108957.06	80254496.84	68587849.49	79429550.55	73349253.41	72369095.39	67779739.96	74028158.57
CALML5	calmodulin-like protein 5 [Homo sapiens]	574654.9722	0	234850.9398	0	697212.3033	250677.2593	523025.5463	454222.0035	0
CALR	calreticulin precursor [Homo sapiens]	22735254.63	27990059.52	30610889.52	29827934.29	31550398.1	30247520.73	30869339.72	25690974.37	29873264.87
CALU	calumenin isoform c precursor [Homo sapiens]	1646215.513	1387376.484	1226517.476	1938454.768	2454410.697	2235819.692	2066504.941	1992722.455	1454302.02
CALU	calumenin isoform d precursor [Homo sapiens]	1646215.513	1387376.484	1226517.476	1938454.768	2454410.697	2235819.692	2066504.941	1992722.455	1454302.02
CAMK1	calcium/calmodulin-dependent protein kinase type 1 [Homo sapiens]	0	0	0	0	0	0	83149.8125	152903.9672	78941.2325
CAMK2G	calcium/calmodulin-dependent protein kinase type II subunit gamma isoform 1	129817.5368	173947.6135	227566.1397	146499.4945	330029.5737	199224.1841	167318.6555	206666.6753	327386.2624
CAMLG	calcium signal-modulating cyclophilin ligand [Homo sapiens]	202442.2367	197439.1814	313349.7654	124265.6642	185684.9334	145890.0497	203757.105	130750.8484	204788.2008
CAMSAP2	calmodulin-regulated spectrin-associated protein 2 [Homo sapiens]	64464.38815	52057.54858	88784.06193	77186.04016	61272.29388	73314.50752	66655.75108	46631.54966	161454.5306
CAND1	cullin-associated NEDD8-dissociated protein 1 [Homo sapiens]	1554626.674	1836961.332	2034163.969	1711747.857	1913780.116	2045083.366	1857610.38	1581447.853	2001227.049
CANT1	soluble calcium-activated nucleotidase 1 [Homo sapiens]	0	0	0	0	0	0	0	58639.44937	85239.60333
CANX	calnexin precursor [Homo sapiens]	10471630.77	13890275.05	13931237.7	12472171.19	14296275.43	13454086.63	13710806.57	11780636.2	15744009.67
CAP1	adenylyl cyclase-associated protein 1 [Homo sapiens]	6575199.505	7169982.637	8913793.647	7813667.774	8384923.005	8139655.068	7950977.442	6860997.779	6947804.711
CAP2	adenylyl cyclase-associated protein 2 [Homo sapiens]	441842.9602	463505.7145	262936.4138	537545.1883	528677.3753	601491.5113	532815.0214	474235.9579	350181.4136
CAPG	macrophage-capping protein isoform 1 [Homo sapiens]	4143330.164	5258169	5277024.773	5277357.143	4825253.135	4820288.062	4539105.563	4310379.522	4680700.888
CAPN1	calpain-1 catalytic subunit [Homo sapiens]	1066927.054	1225667.187	1445818.119	1204518.031	1512693.298	1328442.037	1190917.06	996488.262	1219887.13
CAPN2	calpain-2 catalytic subunit isoform 1 [Homo sapiens]	1954239.805	1978786.982	2470491.47	1973240.794	2380436.207	2006478.468	2033938.585	1805190.991	2492177.804
CAPN3	calpain-3 isoform a [Homo sapiens]	0	0	0	0	0	0	706297.8438	569444.6313	894039.3603
CAPN7	calpain-7 [Homo sapiens]	51936.57402	45284.73534	126439.6361	50952.09518	73098.02282	58077.05218	77721.3542	107961.0759	73290.79644
CAPNS1	calpain small subunit 1 [Homo sapiens]	10217658.15	9993984.958	12793870.67	9700841.932	11861636.72	10402653.91	9395021.574	8179746.09	10376830.8
CAPRIN1	caprin-1 isoform 1 [Homo sapiens]	5916482.04	5526024.89	8006817.92	5247023.31	7204341.46	6042928.535	4165862.567	4661855.845	5250622.41
CAPZA1	F-actin-capping protein subunit alpha-1 [Homo sapiens]	10718975.69	11581859.95	11559694.96	12226437.63	12895002.73	12417831.2	11536970.24	10260418.65	12750740.79
CAPZA2	F-actin-capping protein subunit alpha-2 [Homo sapiens]	2267155.22	2944790.901	3014134.781	2596606.755	3629115.631	3255764.847	3058400.072	1692524.37	2673314.368
CAPZB	F-actin-capping protein subunit beta isoform 1 [Homo sapiens]	6411929.34	6570927.438	8683456.24	8868674.14	9809303.562	8272536.562	8466373.843	6653889.162	8669452.595
CARD6	caspase recruitment domain-containing protein 6 [Homo sapiens]	98067.38814	28620.11275	62092.74069	88940.62335	92591.46278	81728.11124	71941.44045	86506.73697	10593.80056
CARHSP1	calcium-regulated heat stable protein 1 [Homo sapiens]	9484470.62	8931928.644	11964091.11	9817591.998	11700230.17	10216305.49	8263796.336	9026797.506	10162984.19
CARM1	histone-arginine methyltransferase CARM1 [Homo sapiens]	649772.2555	886703.8139	990483.2139	1260907.704	1434283.914	1197849.836	1150197.49	693514.2722	853516.4298
CARS	cysteine--tRNA ligase, cytoplasmic isoform c [Homo sapiens]	727172.5957	723839.5605	853460.7761	713494.3519	912746.4167	754721.698	729515.5594	477602.3568	757039.0314
CARS2	probable cysteine--tRNA ligase, mitochondrial [Homo sapiens]	482834.8929	530048.3322	570615.2893	582517.1132	599187.0907	468865.6657	543749.6654	491002.4332	529426.14
CASC3	protein CASC3 [Homo sapiens]	0	0	0	0	0	0	0	0	0
CASC4	protein CASC4 isoform a [Homo sapiens]	97394.18407	30341.58576	0	63423.83038	67298.27956	45876.59774	28525.61865	23784.56868	164490.7747
CASK	peripheral plasma membrane protein CASK isoform 1 [Homo sapiens]	0	0	0	0	0	0	48715.23246	25055.96562	0
CASP10	caspase-10 isoform 1 preproprotein [Homo sapiens]	0	0	0	0	0	0	24783.66572	0	31582.64141
CASP14	caspase-14 precursor [Homo sapiens]	0	0	0	0	0	0	133701.5586	0	542263.1582
CASP2	caspase-2 isoform 1 preproprotein [Homo sapiens]	262538.5898	196573.0191	335430.1138	273731.2407	363154.8082	287298.9182	253149.8447	226482.7236	223216.5412
CASP3	caspase-3 preproprotein [Homo sapiens]	294542.0466	421176.0873	291132.6698	509606.1768	413191.8631	323711.3733	399004.5396	246764.3571	518162.3824
CASP4	caspase-4 isoform alpha precursor [Homo sapiens]	226222.2375	241175.8325	210425.8211	267328.7089	230563.2025	259990.1596	214384.6691	174679.5056	236392.8642
CASP7	caspase-7 isoform e [Homo sapiens]	0	0	0	0	0	0	0	52536.82711	71151.9606
CASP8	caspase-8 isoform G precursor [Homo sapiens]	453731.6622	422798.819	549221.7396	470733.2509	575124.4009	506574.2622	512348.7275	432825.3215	0
CASP9	caspase-9 isoform alpha precursor [Homo sapiens]	204748.4091	282997.7553	253675.0083	254652.4929	367816.8892	302979.0018	0	0	0
CAST	calpastatin isoform f [Homo sapiens]	1382559.712	1370634.126	1846327.741	1578452.783	1951241.708	1696403.186	1400408.894	1366368.477	1409452.259
CAT	catalase [Homo sapiens]	1983018.574	2023317.786	2630794.667	2060476.616	2986721.826	2153635.182	1992868.549	2534358.742	2347247.524
CAV1	caveolin-1 isoform alpha [Homo sapiens]	1489633.49	1494097.292	807810.8871	1512826.463	0	1604031.224	1862938.352	1547047.423	0
CBFB	core-binding factor subunit beta isoform 2 [Homo sapiens]	969077.2109	1024531.136	950929.4201	949093.4062	1052595.374	921450.0028	834735.013	682129.0836	849975.5554
CBFB	core-binding factor subunit beta isoform 1 [Homo sapiens]	969077.2109	1024531.136	950929.4201	949093.4062	1052595.374	921450.0028	834735.013	682129.0836	849975.5554
CBL	E3 ubiquitin-protein ligase CBL [Homo sapiens]	92583.55581	35701.23888	157736.4898	100056.4331	183857.8022	89305.14628	77015.68172	72673.48594	0
CBR1	carbonyl reductase [NADPH] 1 [Homo sapiens]	9676185.44	10886816.8	10201288.34	11069094.22	12521060.63	11764098.72	11197701.04	9240901.578	12611018.73
CBS	cystathionine beta-synthase [Homo sapiens]	1484021.206	1462370.538	1896615.357	1720148.189	2125115.208	1553517.291	1464773.315	1202914.069	1579504.939
CBX1	chromobox protein homolog 1 [Homo sapiens]	1019299.82	956450.1724	1382169.838	1332037.506	1121152.572	1390728.875	1422748.207	1289284.761	1589380.253
CBX3	chromobox protein homolog 3 [Homo sapiens]	11066562.48	10182253.35	13920710.17	10161259.53	12860039.28	11237586.85	8265283.567	8936677.569	12543007.64
CBX4	E3 SUMO-protein ligase CBX4 [Homo sapiens]	0	0	0	0	0	0	30086.65388	9671.29843	0

CBX5	chromobox protein homolog 5 [Homo sapiens]	4882000.71	3590034.098	5852926.696	5095580.733	6243712.677	5074893.029	4901200.63	3982271.248	4464452.893
CBX8	chromobox protein homolog 8 [Homo sapiens]	104741.6588	106143.7719	112786.4064	93004.18774	133855.4872	99430.23574	18897.39408	0	83235.32437
CC2D1A	coiled-coil and C2 domain-containing protein 1A [Homo sapiens]	86458.81668	96379.0868	62611.71177	61012.4477	89782.53228	107278.2695	64580.02966	46360.66828	92675.50949
CC2D1B	coiled-coil and C2 domain-containing protein 1B [Homo sapiens]	59986.7071	96962.86686	125608.7415	68906.00518	115782.4712	88077.99136	60800.02686	39874.50814	62809.99195
CCAR1	cell division cycle and apoptosis regulator protein 1 [Homo sapiens]	420206.0066	395907.7352	357521.5142	314118.1877	402463.3518	363738.6955	283387.2665	399893.3531	382126.5304
CCBL2	kynurenine--oxoglutarate transaminase 3 isoform 1 [Homo sapiens]	376548.821	436189.8549	483998.8417	426579.4352	491428.9075	454555.6594	436809.0458	403463.6056	606373.6596
CCDC101	SAGA-associated factor 29 homolog [Homo sapiens]	94011.75315	130218.1298	241528.6038	106271.2506	299676.4442	139498.4111	125898.4976	75146.57584	163104.3538
CCDC104	coiled-coil domain-containing protein 104 [Homo sapiens]	0	0	0	0	0	0	0	0	0
CCDC12	coiled-coil domain-containing protein 12 1 [Homo sapiens]	152260.1391	410955.0106	0	0	176286.9509	302618.6355	0	0	0
CCDC124	coiled-coil domain-containing protein 124 [Homo sapiens]	1017298.215	833366.9397	1214055.725	1095084.281	1193216.037	1177461.51	1061624.082	969535.1228	763175.375
CCDC132	coiled-coil domain-containing protein 132 isoform a [Homo sapiens]	99855.44794	0	87897.88095	34677.48828	81930.57411	34190.73853	84814.74486	68706.45763	34328.67835
CCDC134	coiled-coil domain-containing protein 134 precursor [Homo sapiens]	400328.8512	236305.6721	333244.7381	264652.8949	415954.6012	142862.192	0	267998.6295	82695.15179
CCDC137	coiled-coil domain-containing protein 137 [Homo sapiens]	230353.7544	355879.7442	365967.5934	394890.8002	342111.093	366258.4331	288524.3176	123010.4197	311884.6069
CCDC22	coiled-coil domain-containing protein 22 [Homo sapiens]	137970.1133	167682.5735	147331.3741	127396.771	186487.4395	146444.2362	140376.4431	90316.40335	151754.0674
CCDC25	coiled-coil domain-containing protein 25 [Homo sapiens]	482911.8738	627177.9283	657652.4865	616375.1379	1268903.912	646107.3995	635014.6881	500638.7897	657607.588
CCDC43	coiled-coil domain-containing protein 43 isoform 1 [Homo sapiens]	1151014.769	668223.539	1293113.809	781320.227	1409370.222	665245.9317	0	1055158.14	602704.926
CCDC47	coiled-coil domain-containing protein 47 precursor [Homo sapiens]	1114429.513	1333481.404	1654846.708	1329233.419	1633412.387	1174105.238	1267577.84	1131567.559	1363894.673
CCDC50	coiled-coil domain-containing protein 50 short isoform [Homo sapiens]	0	190173.1	0	320351.6444	54830.62903	293484.0279	256381.6097	0	206927.1355
CCDC51	coiled-coil domain-containing protein 51 isoform 1 [Homo sapiens]	573982.3376	606005.85	750433.8062	703905.277	832312.0169	832812.8848	543163.9538	234411.4228	587269.5292
CCDC58	coiled-coil domain-containing protein 58 [Homo sapiens]	2508216.838	3221562.767	3548540.229	3008083.851	4139552.217	3088205.909	3061985.978	2191524.658	3628307.451
CCDC59	thyroid transcription factor 1-associated protein 26 [Homo sapiens]	0	141690.353	0	153850.368	319365.208	168041.006	0	0	0
CCDC6	coiled-coil domain-containing protein 6 [Homo sapiens]	433840.995	627821.7276	759067.0497	707503.8754	619953.3286	832277.8533	529465.3151	583241.802	550718.8899
CCDC86	coiled-coil domain-containing protein 86 [Homo sapiens]	381238.3946	320184.7328	789189.5486	969425.084	1208347.449	759562.832	455200.157	255976.6294	428844.1683
CCDC88A	girdin isoform 1 [Homo sapiens]	14282.62336	14403.37326	32510.45996	20265.65078	25127.89558	21023.17362	14530.82006	11269.36112	20391.58836
CCDC9	coiled-coil domain-containing protein 9 [Homo sapiens]	97837.1753	133958.3147	104108.6086	195631.7669	128173.4475	117454.4562	0	79018.39693	70884.82072
CCDC90B	coiled-coil domain-containing protein 90B, mitochondrial precursor [Homo sa	361767.9949	221340.9898	461222.6066	219880.2534	277949.9744	266469.0743	268369.4283	215329.9325	267474.0102
CCDC93	coiled-coil domain-containing protein 93 [Homo sapiens]	56480.03968	66483.0918	61603.07454	53870.70057	73336.95741	56199.83492	39834.63132	33458.64965	49099.8329
CCDC94	coiled-coil domain-containing protein 94 [Homo sapiens]	303231.5609	213972.7682	127403.088	207699.7864	261029.0841	238937.7391	214163.2312	0	271461.9928
CCDC97	coiled-coil domain-containing protein 97 [Homo sapiens]	68626.84221	434581.6414	308462.9522	305249.5646	537316.1735	253878.7939	214672.4529	358290.9303	187328.8258
CCNB1	G2/mitotic-specific cyclin-B1 [Homo sapiens]	367201.9122	380533.846	477450.038	403046.5473	501731.3634	344074.4752	209420.4024	244211.1538	202731.0858
CCNB1IP1	E3 ubiquitin-protein ligase CCNB1IP1 [Homo sapiens]	0	0	0	0	0	0	734817.0216	636775.5259	0
CCNB2	G2/mitotic-specific cyclin-B2 [Homo sapiens]	609632.7778	280468.3518	324184.7036	554754.8452	421192.6184	584430.1852	514715.911	130938.38	97462.82006
CCNC	cyclin-C isoform a [Homo sapiens]	0	0	0	0	0	0	114965.3914	382221.1204	0
CCNDBP1	cyclin-D1-binding protein 1 [Homo sapiens]	0	236374.0231	205118.7037	0	278406.0833	269244.2179	206641.6412	124119.6951	225060.1806
CCNH	cyclin-H isoform 1 [Homo sapiens]	151418.7921	216637.1965	295780.1244	83042.1844	251879.6544	65550.98382	129333.6137	0	51007.73272
CCNK	cyclin-K [Homo sapiens]	159399.8033	184253.7332	223058.1117	325849.6553	403742.863	157795.489	190165.9508	140460.5386	52723.3245
CCNL1	cyclin-L1 [Homo sapiens]	114115.7235	68648.02843	92196.426	35584.69412	78915.71357	49558.37843	27318.93529	0	76414.53725
CCNT1	cyclin-T1 isoform a [Homo sapiens]	104474.689	72294.40437	109879.5853	91043.95201	192866.8612	120601.2103	115157.3244	104252.2598	147796.1619
CCNY	cyclin-Y isoform 1 [Homo sapiens]	0	0	0	0	0	0	0	0	0
CCS	copper chaperone for superoxide dismutase [Homo sapiens]	0	524397.198	674889.6429	1071246.564	506363.8579	1394400.201	765314.124	843858.3687	1146274.146
CCT2	T-complex protein 1 subunit beta isoform 1 [Homo sapiens]	11021916.33	11624270.5	14609501.75	11514055.89	13969792.1	12356774.99	11468679.23	10215871.21	11576664.61
CCT3	T-complex protein 1 subunit gamma isoform a [Homo sapiens]	9893397.712	11576976.88	12589135.46	11749893.46	14133558.62	12118408.49	11211800.15	9960774.581	11851377.62
CCT4	T-complex protein 1 subunit delta isoform a [Homo sapiens]	9360081.837	11180943.16	13808096.01	11424985.24	12696710.68	11978224.08	11269073.85	10007506.42	12076860.24
CCT5	T-complex protein 1 subunit epsilon [Homo sapiens]	11397085.9	12003176.42	14867501.73	13425276.87	13851844.19	14111064.74	11764477.44	9326066.729	13339970.54
CCT6A	T-complex protein 1 subunit zeta isoform a [Homo sapiens]	9971509.429	10426032.12	13069767.03	10360094.88	12883467.9	11101588.41	9907577.212	8485426.963	10635607.99
CCT7	T-complex protein 1 subunit eta isoform a [Homo sapiens]	8536782.351	9246728.738	11360339.96	9535332.182	11611538.39	10062048.22	9069733.253	7771584.787	10009240.25
CCT8	T-complex protein 1 subunit theta [Homo sapiens]	12138626.23	13556227.95	16278608.79	13477226.09	16838308.83	13621347.62	13677990.41	11843223.24	14662342.98
CCZ1	vacuolar fusion protein CCZ1 homolog [Homo sapiens]	178712.738	126047.5863	357013.7221	160599.8207	242312.8205	69276.23706	99626.17807	133669.5151	279736.4353
CD109	CD109 antigen isoform 1 precursor [Homo sapiens]	36713.58312	87955.54853	46081.56635	38029.6122	39399.68323	65735.32062	0	19634.75251	77353.35987
CD151	CD151 antigen [Homo sapiens]	673406.4383	865055.9595	942398.3442	305416.3864	0	920174.5504	834980.6883	793615.8296	1827420.313
CD276	CD276 antigen isoform a precursor [Homo sapiens]	672135.8068	472070.3012	770650.1552	449567.8905	664909.8273	645642.6291	378128.3807	565533.4542	0
CD2AP	CD2-associated protein [Homo sapiens]	275567.0008	235704.3681	493021.1414	355137.072	505001.2739	387882.9811	336515.3029	307451.3508	352263.6723
CD2BP2	CD2 antigen cytoplasmic tail-binding protein 2 [Homo sapiens]	1119826.69	1733991.695	1346281.629	1698532.142	1635566.571	1965322.526	1431685.315	1322381.199	1404605.382
CD320	CD320 antigen isoform 1 precursor [Homo sapiens]	0	0	0	0	0	0	73794.06646	83171.38246	630269.3097
CD3EAP	DNA-directed RNA polymerase I subunit RPA34 [Homo sapiens]	455684.7288	646679.9867	638815.7218	316778.9718	606868.8152	545937.4515	555194.6508	473450.202	644266.9285
CD44	CD44 antigen isoform 4 precursor [Homo sapiens]	145574.8685	134701.2493	171659.4808	2959617.861	226141.8722	109576.4883	141311.7955	2793243.952	230496.0946
CD44	CD44 antigen isoform 1 precursor [Homo sapiens]	145574.8685	134701.2493	171659.4808	2959617.861	226141.8722	109576.4883	141311.7955	2793243.952	230496.0946
CD46	membrane cofactor protein isoform 2 precursor [Homo sapiens]	989901.109	910525.7507	1052148.396	675080.0716	1208566.84	611872.9235	602158.2315	881525.3724	1552924.68
CD47	leukocyte surface antigen CD47 isoform 1 precursor [Homo sapiens]	847760.2735	1217727.071	903820.6118	1065416.311	0	1141298.71	1309873.288	1184559.593	0
CD55	complement decay-accelerating factor isoform 2 precursor [Homo sapiens]	1871621.405	2342619.883	2331501.853	2058633.904	2010574.663	2200114.209	2159722.277	1804584.865	2433869.761
CD58	lymphocyte function-associated antigen 3 isoform 1 [Homo sapiens]	1131245.992	1596486.397	853075.2941	1634738.781	819351.8134	1663800.265	1585001.809	989431.1507	1592930.059
CD59	CD59 glycoprotein preproprotein [Homo sapiens]	26547558.76	27372822.87	35956716.46	27812090.67	31111696.17	28927180.4	28150458.72	9395192.657	31171270.49
CD63	CD63 antigen isoform A [Homo sapiens]	7679825.055	7306174.607	9575338.724	11888323.68	9668804.199	7815903.861	8283731.651	6965133.972	30485153.45
CD68	macrosialin isoform A precursor [Homo sapiens]	0	0	0	0	0	0	510533.8413	40475.09722	464765.0635
CD81	CD81 antigen [Homo sapiens]	1750284.587	1633894.154	1396695.322	1427119.279	0	942442.7031	2579252.092	1333006.894	892164.274
CD9	CD9 antigen [Homo sapiens]	415096.502	4077527.411	2059388.127	2128120.706	2204063.939	0	4284305.758	1983859.276	4311116.323
CD97	CD97 antigen isoform 1 preproprotein [Homo sapiens]	559256.6502	567284.0974	798338.4013	601978.8502	741549.503	582325.066	685753.7081	431760.3485	659912.6098
CDA	cytidine deaminase [Homo sapiens]	9156912.912	13325651.87	12396957.73	11224759.46	11908679.38	11999942.12	12804820.98	10428495.38	13096369.02
CDC123	cell division cycle protein 123 homolog [Homo sapiens]	1508473.706	1580531.672	2336033.592	1987934.413	2066250.545	2264543.54	1917621.677	1511574.326	2501971.86
CDC16	cell division cycle protein 16 homolog [Homo sapiens]	174090.5495	261850.1888	202915.4591	269399.8356	310995.6539	233433.5013	213065.3675	252134.3862	214941.3001
CDC20	cell division cycle protein 20 homolog [Homo sapiens]	0	58956.04023	221925.8012	170444.4123	179740.0838	99860.52339	0	0	0
CDC23	cell division cycle protein 23 homolog [Homo sapiens]	193046.3619	196517.6459	285981.7295	216520.4952	265160.5006	253773.0792	243594.9032	207598.0885	211762.0041
CDC26	anaphase-promoting complex subunit CDC26 [Homo sapiens]	0	689195.7616	788675	827305.8198	0	967850.4449	723638.6251	0	649568.9158
CDC27	cell division cycle protein 27 homolog isoform 1 [Homo sapiens]	305817.2047	243586.3959	337625.6472	358162.4554	414450.8411	402524.0088	357014.6811	210471.704	486360.9056
CDC34	ubiquitin-conjugating enzyme E2 R1 [Homo sapiens]	0	201784.5292	152584.4448	154090.3742	0	225957.8961	147179.5422	505565.3482	0
CDC37	hsp90 co-chaperone Cdc37 [Homo sapiens]	3985029.043	4400215.085	5437946.658	5363172.155	5530839.134	5487361.925	5283901.632	4410279.226	6789638.578
CDC40	pre-mRNA-processing factor 17 [Homo sapiens]	132523.4417	124841.3428	142043.4175	131185.5339	199949.194	149518.1324	135775.1272	123561.6261	153962.7944
CDC42	cell division control protein 42 homolog isoform 1 precursor [Homo sapiens	16573134.31	16427977.9	18595419.54	17293482.42	17829876.63	18251740.32	16816590.59	10727804.07	16879100.55
CDC42BPA	serine/threonine-protein kinase MRCK alpha isoform B [Homo sapiens]	16483.73301	14643.68153	18908.45406	12789.25043	23713.68689	21266.16644	17418.94105	11851.15569	16130.38766
CDC42BPB	serine/threonine-protein kinase MRCK beta [Homo sapiens]	13471.24887	30494.93718	34166.23056	31412.09475	27748.53988	39562.05014	27713.45296	4491.838325	21468.18394
CDC42EP1	cdc42 effector protein 1 [Homo sapiens]	548313.8934	830318.2227	778640.3869	651935.6778	635350.9413	1065626.022	815872.4206	363283.5028	901550.2069
CDC42EP4	cdc42 effector protein 4 [Homo sapiens]	196631.9065	261112.0334	312809.5264	160829.3467	333104.4994	272940.9468	210487.5434	266429.8192	250852.1806
CDC42SE2	CDC42 small effector protein 2 [Homo sapiens]	3275130.533	4856176.579	5898149.732	5454725.895	4420132.316	6013882.842	4853520.789	3220998.105	3981247.895
CDC45	cell division control protein 45 homolog isoform 1 [Homo sapiens]	91403.64156	129415.1778	191742.5107	113680.7266	185237.7783	161443.4326	76840.44138	125317.2546	77644.75404
CDC5L	cell division cycle 5-like protein [Homo sapiens]	629946.5792	737671.6158	939325.4475	743718.4519	878785.0686	831397.7597	651186.3626	526997.4525	739282.186
CDC7	cell division cycle 7-related protein kinase [Homo sapiens]	0	0	0	0	0	0	0	0	0
CDC73	parafibromin [Homo sapiens]	549009.9915	540018.7849	470269.5772	618551.5419	610708.1257	620521.7004	536104.614	454565.9301	486574.5548
CDCA2	cell division cycle-associated protein 2 [Homo sapiens]	15730.60298	21325.63497	22686.77083	19210.50819	39238.66162	22660.04628	27247.52857	9982.44256	25804.99564
CDCA3	cell division cycle-associated protein 3 [Homo sapiens]	0	0	0	0	0	0	0	0	0
CDCA7L	cell division cycle-associated 7-like protein isoform 1 [Homo sapiens]	28945.77693	0	28666.69826	6349.956833	0	18387.27355	0	0	0
CDIPT	CDP-diacylglycerol--inositol 3-phosphatidyltransferase [Homo sapiens]	1150702.408	1297528.797	1318314.296	606708.065	1538275.168	780416.725	545584	849613.265	1621570.757
CDK1	cyclin-dependent kinase 1 isoform 1 [Homo sapiens]	4014491.575	5592962.128	6446826.727	4881620.805	5669796.491	5418413.191	5015669.445	4269923.385	5965292.613
CDK11B	cyclin-dependent kinase 11B isoform 2 [Homo sapiens]	297198.4683	238494.6702	387165.372	365411.7421	361495.9499	273690.6109	316897.0712	229595.884	331750.2933
CDK12	cyclin-dependent kinase 12 isoform 1 [Homo sapiens]	292986.5597	25724.38294	57515.77661	33361.95958	16287.51015	31720.62487	22599.71061	18407.55081	13632.59768
CDK13	cyclin-dependent kinase 13 isoform 1 [Homo sapiens]	0	28709.2045	35223.30253	42483.22814	34307.84949	47239.50715	33482.66614	7633.054849	41245.18839
CDK16	cyclin-dependent kinase 16 isoform 3 [Homo sapiens]	21738.49111	75105.94004	0	35328.79332	0	20522.36433	46542.0789	27937.60315	17826.90244
CDK2	cyclin-dependent kinase 2 isoform 1 [Homo sapiens]	3288356.442	2438766.075	4424354.59	3574333.076	4559091.051	3830251.998	3592582.286	3141919.5	3932568.38
CDK2AP1	cyclin-dependent kinase 2-associated protein 1 isoform 1 [Homo sapiens]	2153443.063	1799735.226	2019785.03	2469163.298	2160467.997	2085517.089	2226785.577	1425754.052	349571.0388
CDK4	cyclin-dependent kinase 4 [Homo sapiens]	1318902.003	1884145.725	1908591.728	1667894.674	1092646.232	1693835.03	1743601.478	1303439.744	1326136.234
CDK5	cyclin-dependent kinase 5 isoform 1 [Homo sapiens]	523400.4896	518737.1758	786811.5016	554439.1504	645012.447	266570.7764	560298.1341	214855.1958	481835.0905
CDK5RAP2	CDK5 regulatory subunit-associated protein 2 isoform a [Homo sapiens]	5930.994249	8040.787076	14917.15678	6745.119249	10199.74924	8121.862591	0	0	0
CDK5RAP3	CDK5 regulatory subunit-associated protein 3 isoform b [Homo sapiens]	350993.0434	483180.5708	509708.2612	436155.4146	574929.8571	474434.9653	458647.1512	325582.9409	0
CDK6	cyclin-dependent kinase 6 [Homo sapiens]	452182.3066	540683.8098	829209.674	446637.0576	1099094.789	1040636.376	775526.1069	735633.8356	1007983.56
CDK7	cyclin-dependent kinase 7 [Homo sapiens]	308737.9947	341275.4565	521079.5943	510984.5933	513015.9981	439246.0966	589643.1269	465357.1987	419012.8063
CDK9	cyclin-dependent kinase 9 [Homo sapiens]	497752.2795	553149.3164	708607.7866	699280.6561	696512.3497	573540.5303	659638.5006	539913.6291	554627.1019
CDKAL1	threonylcarbamoyladenosine tRNA methylthiotransferase [Homo sapiens]	195957.6237	232733.021	238715.2029	405513.2038	284560.3076	250080.853	402138.2214	191352.6937	245441.8998
CDKN2A	cyclin-dependent kinase inhibitor 2A isoform p16gamma [Homo sapiens]	4543069.394	6546578.745	6460454.74	6485704.747	6636949.859	6176367.57	5896316.16	5103269.265	5461968.735
CDKN2A	cyclin-dependent kinase inhibitor 2A isoform p14ARF [Homo sapiens]	4543069.394	6546578.745	6460454.74	6485704.747	6636949.859	6176367.57	5896316.16	5103269.265	5461968.735
CDKN2AIP	CDKN2A-interacting protein [Homo sapiens]	1280700.054	348862.7432	350265.8317	328672.3861	405367.3797	319047.3131	283041.8082	230091.3627	350699.9004
CDKN2AIPNL	CDKN2AIP N-terminal-like protein [Homo sapiens]	0	0	0	0	0	0	0	0	0
CDKN2C	cyclin-dependent kinase 4 inhibitor C [Homo sapiens]	229157.7903	150693.4725	0	322845.9237	0	316235.9449	0	164810.7797	251925.2415
CDSN	corneodesmosin precursor [Homo sapiens]	540443.5907	672012.5637	0	330612.4485	959877.3113	0	674279.6508	184764.2328	919035.0478
CDT1	DNA replication factor Cdt1 [Homo sapiens]	14577.51267	16589.87217	23640.51033	0	0	19301.48204	0	0	0
CDV3	protein CDV3 homolog isoform b [Homo sapiens]	4230918.081	5562947.125	7016325.685	4602423.736	6811114.354	5147226.36	4821297.424	4580027.074	6358458.046
CDYL	chromodomain Y-like protein isoform a [Homo sapiens]	126250.2956	136309.2936	186111.88	174577.5654	155975.5775	125331.0064	135054.7709	106682.0691	173750.1694
CEBPB	CCAAT/enhancer-binding protein beta [Homo sapiens]	461991.2051	434663.1667	239259.2961	489765.9595	618244.9079	457994.5707	217066.6042	200028.9754	82141.04605
CEBPZ	CCAAT/enhancer-binding protein zeta [Homo sapiens]	251870.4301	277491.7851	339555.926	267260.4851	369693.814	329332.2219	270848.6726	219930.0985	294036.3498
CECR5	cat eye syndrome critical region protein 5 isoform 2 precursor [Homo sapien	1408876.193	972375.2874	979500.0615	1686206.314	1042089.68	1741939.855	1634677.24	1473224.836	768713.028
CELF1	CUGBP Elav-like family member 1 isoform 3 [Homo sapiens]	967771.213	593849.0909	1228099.695	553302.4783	340149.4072	630684.1322	465326.3623	337795.771	1104624.156
CELF1	CUGBP Elav-like family member 1 isoform 4 [Homo sapiens]	967771.213	593849.0909	1228099.695	553302.4783	340149.4072	630684.1322	465326.3623	337795.771	1104624.156

CENPB	major centromere autoantigen B [Homo sapiens]	0	94475.12058	123334.4666	90467.56283	112649.6545	78694.29188	62056.03995	81435.45649	42755.29276
CENPF	centromere protein F [Homo sapiens]	5467.08174	20350.22252	14956.86031	12479.18013	12603.67585	9828.956824	4510.192196	0	8572.546728
CENPH	centromere protein H [Homo sapiens]	0	0	0	0	0	0	0	0	0
CENPV	centromere protein V [Homo sapiens]	1169248.53	1122354.113	1352905.475	1141003.485	1118886.677	1194298.77	1159498.062	783364.3292	997152.3636
CEP170	centrosomal protein of 170 kDa isoform alpha [Homo sapiens]	75158.25268	97726.00497	82821.2919	90672.66566	99964.86837	94524.79877	106323.3879	107763.103	91517.86359
CEP41	centrosomal protein of 41 kDa isoform 1 [Homo sapiens]	11454.4896	18091.99897	13992.78063	16411.27132	13939.77566	0	12454.28699	0	19159.49617
CEP55	centrosomal protein of 55 kDa [Homo sapiens]	111388.3275	224696.4994	155063.3323	150908.0515	139835.3237	139548.9129	149762.0347	127911.9738	114701.1357
CEP85	centrosomal protein of 85 kDa [Homo sapiens]	0	0	0	0	0	0	85447.4783	30717.23524	0
CEP97	centrosomal protein of 97 kDa [Homo sapiens]	145648.4946	187209.4695	159171.0274	170925.985	147216.0666	202266.2979	93876.97218	232271.3397	102516.6514
CEPT1	choline/ethanolaminephosphotransferase 1 [Homo sapiens]	1070051.142	1295868.86	1353662.329	1448303.612	1463696.842	1536514.619	1444457.002	2226656.294	1491774.434
CERS2	ceramide synthase 2 [Homo sapiens]	3085280.672	1805668.718	4850752.4	3121319.282	4368294.293	3671419.857	3733091.314	2976875.341	3958115.24
CERS5	ceramide synthase 5 [Homo sapiens]	0	0	0	0	0	0	0	0	0
CETN2	centrin-2 [Homo sapiens]	820893.5552	802594.3533	1492905.601	1280350.94	1354133.913	1174649.775	1101537.938	843177.4393	840394.5558
CFDP1	craniofacial development protein 1 [Homo sapiens]	307190.443	0	432378.6829	411052.8691	457715.5067	0	334963.651	294091.7617	298102.094
CFL1	cofilin-1 [Homo sapiens]	108255800.4	136284907	122975864.8	102949899	131970983.4	135498046.4	118853779.4	122527647.1	142424705.5
CFL2	cofilin-2 isoform 1 [Homo sapiens]	3731342.137	4877411.946	4497404.155	3752794.218	3692386.842	5097176.25	3644236.117	2738244.177	5339883.083
CGGBP1	CGG triplet repeat-binding protein 1 [Homo sapiens]	269288.3086	160037.4359	0	159779.4599	0	201234.3878	280378.1312	186746.3588	186294.3858
CGREF1	cell growth regulator with EF hand domain protein 1 isoform a precursor [Ho	0	0	0	0	0	0	27568.37843	42712.77859	0
CHAC2	cation transport regulator-like protein 2 [Homo sapiens]	0	0	0	0	0	0	0	494791.033	1055337.357
CHAF1A	chromatin assembly factor 1 subunit A [Homo sapiens]	106078.2597	154839.3979	101346.5639	129921.766	114453.6883	109133.1021	113310.3179	64259.33745	144697.3941
CHAF1B	chromatin assembly factor 1 subunit B [Homo sapiens]	224714.9306	257495.0156	276759.5966	218089.4423	243204.4263	111094.2903	0	145020.6328	322472.7089
CHAMP1	chromosome alignment-maintaining phosphoprotein 1 [Homo sapiens]	153884.6851	149108.9319	130928.08	190267.5709	132664.4433	189562.7673	89382.83581	111465.2505	86816.33575
CHCHD10	coiled-coil-helix-coiled-coil-helix domain-containing protein 10, mitochon	2527750.739	4388429.943	1805797.841	5217509.218	2132092.034	5207030.909	3827020.182	4344084.466	3083314.557
CHCHD2	coiled-coil-helix-coiled-coil-helix domain-containing protein 2, mitochondi	6072462.907	6162687	8466295.641	8496677.702	8031889.393	8630757.617	5957081.707	4972388.532	4938781.244
CHCHD3	coiled-coil-helix-coiled-coil-helix domain-containing protein 3, mitochondi	1713049.945	1642044.905	2224759.1	1673798.069	2423779.841	1825565.318	1795715.507	1182127.933	1787073.242
CHCHD4	mitochondrial intermembrane space import and assembly protein 40 isoform 2	1392971.914	1492079.426	2403353.938	1403714.797	2045398.545	1260714.392	1368280.16	900619.6364	1728079.236
CHCHD5	coiled-coil-helix-coiled-coil-helix domain-containing protein 5 [Homo sapie	0	1258256.883	495502.9727	1860099.415	1295044.96	1093656.53	1608250.528	1621178.78	616969.2515
CHCHD6	coiled-coil-helix-coiled-coil-helix domain-containing protein 6, mitochondi	294802.1203	443499.9103	541651.6531	357789.0165	543865.9144	230282.2107	548887.7258	256428.1885	380270.1781
CHD1	chromodomain-helicase-DNA-binding protein 1 [Homo sapiens]	35628.2134	30322.69264	47585.42742	35986.84402	64179.72356	73615.96366	34945.73865	27145.67847	52873.30276
CHD2	chromodomain-helicase-DNA-binding protein 2 isoform 1 [Homo sapiens]	0	0	0	0	0	0	11156.11721	7012.652209	12925.66643
CHD3	chromodomain-helicase-DNA-binding protein 3 isoform 3 [Homo sapiens]	41594.55082	30943.1374	43339.8564	35184.70918	41643.51688	41006.5811	37804.41129	17549.79179	36527.84514
CHD4	chromodomain-helicase-DNA-binding protein 4 [Homo sapiens]	266664.1833	251884.088	329196.8627	249668.7831	410491.4065	309499.001	248238.7631	218064.8327	315062.2704
CHD5	chromodomain-helicase-DNA-binding protein 5 [Homo sapiens]	0	0	0	0	0	0	0	0	0
CHD8	chromodomain-helicase-DNA-binding protein 8 isoform 1 [Homo sapiens]	19214.16269	17029.68353	29480.55963	25528.92304	7356.522469	34132.16904	22883.29483	18928.44676	27544.92317
CHEK1	serine/threonine-protein kinase Chk1 isoform 1 [Homo sapiens]	166904.6682	117423.3121	174291.608	155568.0726	182967.8645	163816.6506	100210.5587	77902.06187	123746.9366
CHERP	calcium homeostasis endoplasmic reticulum protein [Homo sapiens]	524590.2625	665030.6125	566978.0635	428144.8049	650488.8957	621090.6819	645675.0813	573758.3556	672734.2431
CHID1	chitinase domain-containing protein 1 isoform b precursor [Homo sapiens]	130678.7453	0	122185.8547	111226.9305	220013.5063	151574.1547	102101.8953	0	150044.2219
CHKA	choline kinase alpha isoform a [Homo sapiens]	0	0	0	0	0	0	93287.37202	0	173342.4933
CHM	rab proteins geranylgeranyltransferase component A 1 isoform a [Homo sapier	152236.3744	121178.5767	61701.81159	123975.5117	76920.67391	96314.67411	126726.7569	146050.9555	269050.0966
CHMP1A	charged multivesicular body protein 1a isoform 2 [Homo sapiens]	397667.8017	533223.4138	562889.7414	565302.796	600034.1034	564139.5977	503458.5374	509397.5661	439232.8448
CHMP2A	charged multivesicular body protein 2a [Homo sapiens]	90894.79931	552790.0566	196871.4702	630213.2887	287735.6606	660807.4817	462418.1567	113501.8253	489111.0306
CHMP2B	charged multivesicular body protein 2b isoform 1 [Homo sapiens]	814778.7835	898993.017	281215.526	414794.595	108003.5938	592013.5357	673153.1121	881758.6007	901157.6875
CHMP4A	charged multivesicular body protein 4a [Homo sapiens]	1422095.408	1642434.354	1793360.142	1666973.042	2075404.548	1583502.697	1443230.106	1305291.16	1645538.157
CHMP4B	charged multivesicular body protein 4b [Homo sapiens]	1815700.405	2281809.122	2418445.253	1532067.849	2500296.608	2425924.366	1832518.256	1865026.648	2145139.351
CHMP5	charged multivesicular body protein 5 isoform 1 [Homo sapiens]	1000231.751	1395818.827	1051971.784	1721109.438	1136260.117	1646513.909	1092381.569	701181.4626	1670577.526
CHMP6	charged multivesicular body protein 6 [Homo sapiens]	179695.7644	153390.2414	137816.4713	0	190602.4713	0	0	0	0
CHMP7	charged multivesicular body protein 7 [Homo sapiens]	228129.6801	237793.2393	261644.3061	271031.5374	291814.1864	266550.9181	217277.5656	165947.4906	152123.6364
CHORDC1	cysteine and histidine-rich domain-containing protein 1 isoform a [Homo sa	3870020.581	4762269.439	5372416.74	4151032.243	5352573.524	5079533.928	4479095.982	3696498.469	5712444.496
CHP1	calcineurin B homologous protein 1 [Homo sapiens]	1048152.542	1295030.589	1398574.612	912120.9963	1269853.021	481727.1761	779871.2424	700589.5739	1273602.467
CHRAC1	chromatin accessibility complex protein 1 [Homo sapiens]	2049954.427	2572749.507	2966777.42	2393623.069	1710893.55	2327292.242	2313825.658	1993761.507	2970738.582
CHST14	carbohydrate sulfotransferase 14 [Homo sapiens]	91294.4569	89356.76281	106829.4223	52607.43494	239032.4327	131124.8904	113701.2251	0	30015.26608
CHST3	carbohydrate sulfotransferase 3 [Homo sapiens]	99859.21108	62611.07783	0	69754.36175	92805.9366	164052.6014	126206.5991	66443.50472	81331.51887
CHTF18	chromosome transmission fidelity protein 18 homolog [Homo sapiens]	43028.86474	42892.00105	50406.0699	65018.36099	75112.32516	55691.47183	46078.99513	29112.73309	40992.05335
CHTOP	chromatin target of PRMT1 protein isoform 2 [Homo sapiens]	1522937.672	1467698.727	1363774.536	1203171.613	2238517.158	1267774.49	1661200.574	910396.5389	1686355.657
CHUK	inhibitor of nuclear factor kappa-B kinase subunit alpha [Homo sapiens]	114588.8566	62059.99104	81985.09677	0	100508.1147	0	84207.94086	35812.70072	30711.16779
CHURC1-FNTB	CHURC1-FNTB protein isoform 1 [Homo sapiens]	90399.36034	88057.99664	0	97207.46396	53890.01741	124371.0872	86036.72819	96151.35698	0
CIAO1	probable cytosolic iron-sulfur protein assembly protein CIAO1 [Homo sapiens	1424018.108	1796728.393	2275557.293	1226044.321	1864500.22	1592609.057	1875110.821	1395800.976	1958701.729
CIAPIN1	anamorsin [Homo sapiens]	1276045.361	1505566.524	1578589.654	2387088.847	1266350.256	1164830.465	941630.7425	610640.4944	1549509.907
CIB1	calcium and integrin-binding protein 1 isoform a [Homo sapiens]	1311239.065	705385.4451	793506.8163	802504.9036	732698.6777	810291.7595	523107.4699	108843.8404	540082.753
CIRBP	cold-inducible RNA-binding protein [Homo sapiens]	4202333.517	5013409.598	5810111.01	5012164.73	5756499.979	4993909.853	4721820.687	3990876.533	4795150.757
CIRH1A	cirhin [Homo sapiens]	712692.505	795088.7622	1001264.788	890220.9992	1045147.518	903630.1824	853871.1399	569777.1256	886767.2239
CISD1	CDGSH iron-sulfur domain-containing protein 1 [Homo sapiens]	8675864	8911637.758	11294509.65	7625440.292	11716719.13	6614316.068	7704689.98	5543190.138	11559291.93
CISD2	CDGSH iron-sulfur domain-containing protein 2 [Homo sapiens]	2803928.024	3295150.682	3740258.616	3917553.813	4216891.05	3991635.353	3687193.335	3347113.959	3651889.337
CIT	citron Rho-interacting kinase isoform 1 [Homo sapiens]	15799.84545	30353.46609	9199.511198	17118.30339	5971.190994	2129.756641	5898.236003	2711.476791	7768.558513
CIZ1	cip1-interacting zinc finger protein isoform 5 [Homo sapiens]	14418.25475	72308.67651	33297.88734	13576.89286	29815.33757	25538.5814	24999.87947	0	25091.90459
CKAP2	cytoskeleton-associated protein 2 isoform 2 [Homo sapiens]	105708.6283	34204.22501	51341.23043	63859.87754	102135.2464	62766.84893	48476.79855	35847.45072	0
CKAP4	cytoskeleton-associated protein 4 [Homo sapiens]	1726882.413	1916373.957	2154547.965	1815376.017	2492234.948	1834479.036	1949101.547	1668145.61	2008245.509
CKAP5	cytoskeleton-associated protein 5 isoform a [Homo sapiens]	128358.1499	174609.9942	157273.4907	105554.073	181874.0603	185360.1595	119873.7853	112752.6941	152794.5537
CKB	creatine kinase B-type [Homo sapiens]	3323336.816	3936955.278	4210974.071	4002352.938	4395095.917	4353719.682	2906068.36	2537999.22	2658209.859
CKS1B	cyclin-dependent kinases regulatory subunit 1 [Homo sapiens]	3970767.185	5793086.079	1694586.21	4263792.25	2643983.109	4624409.95	4902984.038	4221132.665	3927538.482
CKS2	cyclin-dependent kinases regulatory subunit 2 [Homo sapiens]	3867087.847	3996649.75	3641185.242	3855982.467	3719075.372	3756613.022	2955282.444	2446418.814	3340498.397
CLASP1	CLIP-associating protein 1 isoform 1 [Homo sapiens]	79759.80965	80326.87143	105373.9936	93510.87914	92428.04128	102219.5711	87912.71272	81882.11998	69686.58516
CLASP2	CLIP-associating protein 2 isoform 1 [Homo sapiens]	102898.391	93547.2826	622099.6317	81634.12959	93124.51891	540103.2206	70571.00778	51918.44281	93561.56994
CLASRP	CLK4-associating serine/arginine rich protein isoform 1 [Homo sapiens]	0	0	0	0	0	0	0	0	0
CLCC1	chloride channel CLIC-like protein 1 isoform 1 precursor [Homo sapiens]	210821.1997	258323.8214	214043.8359	223739.956	167221.8772	247829.5731	233594.6635	134432.0275	291751.3581
CLCN7	H(+)/Cl(-) exchange transporter 7 isoform a [Homo sapiens]	0	0	0	0	0	0	0	15193.75352	47921.47708
CLEC2B	C-type lectin domain family 2 member B [Homo sapiens]	693022.7083	582582.3448	770963.2667	527533.4792	749221.973	577959.8657	601041.9083	627220.6343	810796.0215
CLGN	calmegin precursor [Homo sapiens]	0	57914.98224	59051.29095	120583.2357	61867.29297	0	0	0	0
CLIC1	chloride intracellular channel protein 1 [Homo sapiens]	30713629.01	34801495.95	42240760.16	36839401.8	44255035.49	39149422.46	37027981.61	33006574.78	37536928.99
CLIC4	chloride intracellular channel protein 4 [Homo sapiens]	1739851.529	2215491.677	2665845.376	2272431.063	2822842.086	2473171.493	2443338.316	1919859.383	2339360.924
CLINT1	clathrin interactor 1 isoform 1 [Homo sapiens]	1272722.228	1266323.95	1497113.597	978994.0866	1544117.066	686880.3791	897929.5578	960842.0738	987774.8877
CLIP1	CAP-Gly domain-containing linker protein 1 isoform b [Homo sapiens]	103269.1501	129976.4826	129808.9557	101653.7252	131526.1978	0	98078.9553	103147.486	125819.5064
CLK3	dual specificity protein kinase CLK3 isoform a [Homo sapiens]	0	122721.4347	0	73142.70933	18764.11519	72977.17147	59376.61279	0	76992.78304
CLMP	CXADR-like membrane protein precursor [Homo sapiens]	0	0	0	0	0	0	0	0	0
CLN3	battenin [Homo sapiens]	233285.7677	264056.8404	515324.442	0	547528.0046	256191.5583	466479.494	328377.0422	551321.5301
CLN6	ceroid-lipofuscinosis neuronal protein 6 [Homo sapiens]	568730.8043	1508113.642	1243574.323	1736133.75	1157567.421	1805406.757	1418332.533	1256052.204	1371379.428
CLNS1A	methylosome subunit pICln [Homo sapiens]	10651590.66	11455017.63	13981433.71	13227888.54	13537593.42	12276402.17	10781854.38	9284902.229	12068496.57
CLP1	polyribonucleotide 5'-hydroxyl-kinase Clp1 isoform 1 [Homo sapiens]	194422.2147	356736.1949	109318.0186	0	85437.24534	0	352566.6677	0	338177.4232
CLPB	caseinolytic peptidase B protein homolog isoform 2 [Homo sapiens]	0	754738.8841	781923.9299	676920.4169	906627.1581	798504.6127	696368.2753	523183.1828	955560.1964
CLPP	putative ATP-dependent Clp protease proteolytic subunit, mitochondrial pre	3397474.528	3722472.58	3953434.668	3354913.36	4334408.989	3513140.019	3574030.885	3051305.476	3865095.143
CLPTM1	cleft lip and palate transmembrane protein 1 [Homo sapiens]	562113.421	694518.4462	764756.3349	666555.8323	929773.8113	512064.8959	498489.3072	436379.3562	749250.4703
CLPTM1L	cleft lip and palate transmembrane protein 1-like protein [Homo sapiens]	1697971.618	1600808.937	2286289.516	1909458.235	2162886.618	1974425.476	1846839.719	1453165.514	1874356.342
CLPX	ATP-dependent Clp protease ATP-binding subunit clpX-like, mitochondrial pre	563604.0088	647645.5232	664983.5029	635890.3985	743779.0331	622769.8669	638369.2063	510901.9386	557959.9283
CLSPN	claspin isoform 1 [Homo sapiens]	13788.01171	11046.90836	24555.61842	8405.782718	29810.96241	12389.40543	5639.428925	6204.837406	18740.64439
CLTA	clathrin light chain A isoform a [Homo sapiens]	4432041.714	5695328.819	5428048.379	7300742.329	8228577.82	5757618.294	5530486.899	4910241.424	5477894.016
CLTB	clathrin light chain B isoform b [Homo sapiens]	2593827.205	2933572.872	5012073.41	4436146.654	3309460.085	2980579.85	2927227.306	3023360.624	4649519.331
CLTC	clathrin heavy chain 1 [Homo sapiens]	3037813.508	3230951.898	4306237.267	3453787.137	4116880.01	3553221.851	3520079.383	2949600.913	3565920.447
CLTCL1	clathrin heavy chain 2 isoform 1 [Homo sapiens]	10432.83604	0	21869.1599	155344.7932	0	156665.558	0	0	0
CMAS	N-acylneuraminate cytidylyltransferase [Homo sapiens]	300795.006	343941.8057	377742.3303	228741.8357	325665.3204	360036.1554	331703.1852	194419.6297	328196.7241
CMBL	carboxymethylenebutenolidase homolog [Homo sapiens]	4408721.897	4558759.686	5208870.057	5296450.959	5803169.389	5303716.716	3911800.977	4123993.697	5271807.79
CMC1	COX assembly mitochondrial protein homolog [Homo sapiens]	221768.9877	842709.5738	0	413129.6025	0	503594.0492	496942.6721	549725.3934	258346.6639
CMC2	COX assembly mitochondrial protein 2 homolog [Homo sapiens]	2322781.146	2821457.502	1683819.93	2826886.446	2074670.707	2272910.366	0	1634769.012	2789435.163
CMIP	C-Maf-inducing protein isoform C-Mip [Homo sapiens]	74386.00373	37806.32526	75548.66325	45931.75437	82769.80597	69077.67071	78245.28243	40030.25776	0
CMPK1	UMP-CMP kinase isoform a [Homo sapiens]	6237766.613	6573282.476	8961233.191	7124906.783	8741463.534	7318868.762	6746209.19	6153014.796	6747182.049
CMSS1	protein CMSS1 isoform 1 [Homo sapiens]	652001.0633	881134.3407	1224630.027	1099918.604	1296279.879	1148441.624	760109.9185	691377.2889	900751.4141
CNBP	cellular nucleic acid-binding protein isoform 3 [Homo sapiens]	4336118.245	5024496.172	6616864.992	4894800.711	4869935.713	5418076.442	5127866.925	3222530.182	5576150.134
CNBP	cellular nucleic acid-binding protein isoform 2 [Homo sapiens]	4336118.245	5024496.172	6616864.992	4894800.711	4869935.713	5418076.442	5127866.925	3222530.182	5576150.134
CNDP2	cytosolic non-specific dipeptidase isoform 1 [Homo sapiens]	1034828.295	1081450.517	1415590.805	1166237.322	1497613.715	1181037.021	1099275.311	941442.7611	1198014.649
CNIH4	protein cornichon homolog 4 isoform 1 [Homo sapiens]	12376071.28	11973980.17	16604165.55	12608146.72	18667368.55	13912334.02	14330403.1	11523333.69	2804855.345
CNN2	calponin-2 isoform a [Homo sapiens]	3578105.24	3398419.881	3987702.454	3829166.383	3577222.787	3581923.664	4115869.21	3284067.18	3779129.613
CNN3	calponin-3 [Homo sapiens]	340614.5627	601792.3127	445132.2039	532756.854	455595.2227	126546.0393	465822.5572	390265.7927	637123.3186
CNNM3	metal transporter CNNM3 isoform 1 precursor [Homo sapiens]	72333.16848	53138.76979	87124.17029	51092.357	85687.2037	68309.06329	60109.1142	50313.62193	23422.47454
CNO	biogenesis of lysosome-related organelles complex 1 subunit 4 [Homo sapiens	0	0	0	0	0	0	103415.487	231663.5764	287045.6883
CNOT1	CCR4-NOT transcription complex subunit 1 isoform a [Homo sapiens]	159457.4831	153392.3941	200572.8924	137623.2965	233385.2429	185192.2851	0	99983.44931	136410.4394
CNOT10	CCR4-NOT transcription complex subunit 10 isoform 3 [Homo sapiens]	105630.2603	92651.91292	0	130107.6317	146129.0311	250734.5034	234831.3248	199936.4619	174682.7269
CNOT11	CCR4-NOT transcription complex subunit 11 [Homo sapiens]	256836.8649	228749.7625	339721.5755	269443.0508	348840.0107	395545.5513	265760.6495	224419.2245	284662.2227
CNOT2	CCR4-NOT transcription complex subunit 2 [Homo sapiens]	286348.7256	0	355228.6803	267119.4099	387640.5232	335934.1809	265506.5068	88111.88526	208713.4368
CNOT3	CCR4-NOT transcription complex subunit 3 [Homo sapiens]	177394.4146	258280.979	138262.5905	244420.5117	273004.6089	291059.4686	280030.2372	820801.893	153218.2336
CNOT7	CCR4-NOT transcription complex subunit 7 isoform 1 [Homo sapiens]	257997.6092	629134.0078	414124.7787	672635.4138	544725.1437	917237.8205	837090.5079	420624.1523	960418.9354
CNOT8	CCR4-NOT transcription complex subunit 8 [Homo sapiens]	117074.1709	289205.3944	0	351533.5778	176755.2583	338235.225	240412.2711	203296.6611	355805.816

CNP	2',3'-cyclic-nucleotide 3'-phosphodiesterase [Homo sapiens]	833767.045	820462.9431	1082435.058	842846.8849	1191968.849	967335.3381	862910.7853	721343.3427	851200.4427
CNPY2	protein canopy homolog 2 isoform 1 precursor [Homo sapiens]	5917742.634	7999972.442	7634021.768	8608587.369	5809151.432	8746867.892	9242466.849	8335315.737	8578996.351
CNPY3	protein canopy homolog 3 precursor [Homo sapiens]	372725.2325	397387.1029	530513.6107	252918.4269	285023.2224	251257.0305	274171.0276	334416.0224	694501.1695
CNPY4	protein canopy homolog 4 precursor [Homo sapiens]	115425.3333	263779.6001	243327.3118	0	479852.4254	300866.957	219630.7245	249726.3482	250040.4173
COA3	cytochrome c oxidase assembly protein 3 homolog, mitochondrial [Homo sapier	1828701.418	1731600.469	1219609.536	1823939.602	841553.9246	1740818.939	1914885.615	37224.67415	1292024.242
COA4	cytochrome c oxidase assembly factor 4 homolog, mitochondrial [Homo sapien:	2362137.319	1717060.608	1867509.278	3749527.453	3411475.583	2362663.212	2612480.233	3508435.369	2695561.256
COA6	cytochrome c oxidase assembly factor 6 homolog isoform 1 [Homo sapiens]	3497503.756	4551600.463	4270185.694	4435248.789	4618283.25	4121151.113	4103003.802	3666650.778	4572485.789
COASY	bifunctional coenzyme A synthase isoform c [Homo sapiens]	1578389.227	1297700.444	2018036.336	1577269.54	1961180.101	1620945.158	1504855.34	1298775.508	1332172.512
COBL	protein cordon-bleu [Homo sapiens]	14779.84472	20546.66338	34269.98178	23396.85565	40308.82895	19113.06503	12258.17529	13898.37702	7417.229318
COG1	conserved oligomeric Golgi complex subunit 1 [Homo sapiens]	0	0	0	0	0	0	0	70872.49847	31590.4929
COG2	conserved oligomeric Golgi complex subunit 2 isoform 1 [Homo sapiens]	58805.50319	64761.65472	63134.29697	47019.87785	78012.79697	56535.16329	46526.57208	40493.64007	77279.02769
COG3	conserved oligomeric Golgi complex subunit 3 [Homo sapiens]	79663.11417	101705.1004	246856.3492	110010.1774	138611.8188	83444.52813	113937.655	86977.86967	204615.2958
COG4	conserved oligomeric Golgi complex subunit 4 isoform 1 [Homo sapiens]	133197.709	116540.2534	143342.2603	135365.29	200707.0163	109310.9045	118570.7535	97910.27068	69676.943
COG5	conserved oligomeric Golgi complex subunit 5 isoform 1 [Homo sapiens]	94737.27636	109479.9434	107180.9865	162998.9769	120745.7857	111151.0015	128997.8516	113752.5338	101253.1016
COG6	conserved oligomeric Golgi complex subunit 6 isoform 1 [Homo sapiens]	104504.1624	123959.1893	28092.4223	132201.2485	108571.2466	83514.47937	108667.6516	88570.16441	129301.7883
COG7	conserved oligomeric Golgi complex subunit 7 [Homo sapiens]	80287.50591	114967.0513	88057.65462	83757.29385	78710.28223	59075.95514	139099.0655	47498.59008	130924.8649
COG8	conserved oligomeric Golgi complex subunit 8 [Homo sapiens]	103876.6063	102964.6034	89512.1269	142073.0478	89779.76	140015.1664	121114.8582	126668.5225	97774.8875
COIL	coilin [Homo sapiens]	256082.8906	446122.9556	420816.7041	313827.5995	431421.2111	468033.1984	468777.1449	396870.0878	432532.486
COL12A1	collagen alpha-1(XII) chain long isoform precursor [Homo sapiens]	14340.87688	8111.788334	16358.17095	14301.48457	15929.6602	11532.5367	12124.74381	7550.059109	11423.58209
COL3A1	collagen alpha-1(III) chain preproprotein [Homo sapiens]	72809.51635	55821.56665	47092.15397	44764.02759	81457.19353	85472.31167	58400.47344	0	66605.23995
COL4A2	collagen alpha-2(IV) chain preproprotein [Homo sapiens]	0	24677.51271	46709.36403	45656.14373	54006.87355	48110.30971	41853.98574	32696.43627	39373.68929
COL4A3BP	collagen type IV alpha-3-binding protein isoform 3 [Homo sapiens]	105136.292	11019.97337	156669.9075	95560.79316	237242.8892	108202.4868	213131.3514	98625.61598	48754.66065
COL7A1	collagen alpha-1(VII) chain precursor [Homo sapiens]	17261.43114	13215.20278	25493.6342	18528.43944	26419.99104	12183.40297	15695.98807	15257.20124	18894.20855
COMMD1	COMM domain-containing protein 1 [Homo sapiens]	511185.5894	560376.5236	0	483849.8478	1111573	647570.7946	488146.1377	388160.2319	678254.1486
COMMD10	COMM domain-containing protein 10 [Homo sapiens]	156103.5232	220593.7053	314850.4568	317509.0864	344054.4938	337055.821	0	206374.6049	263999.3611
COMMD2	COMM domain-containing protein 2 [Homo sapiens]	364440.7055	1092990.021	350147.7979	790205.7134	196893.8459	1367865.456	992223.1393	813824.4977	569198.7016
COMMD3	COMM domain-containing protein 3 [Homo sapiens]	947799.7917	707697.9028	824871.7118	549522.0046	843313.309	686676.5243	683759.066	0	841870.066
COMMD3-BMI1	COMMD3-BMI1 read-through protein [Homo sapiens]	0	0	0	0	0	0	48423.06217	276269.7712	59156.28571
COMMD4	COMM domain-containing protein 4 [Homo sapiens]	523008.0628	649496.8816	925742.3487	774219.7654	917356.9912	517896.5438	147456.9013	389485.8307	670228.0909
COMMD6	COMM domain-containing protein 6 isoform a [Homo sapiens]	648896.2669	1055854.709	973986.4777	1341650.686	1654694.971	1386490.027	1437533.368	1173666.893	983479.018
COMMD7	COMM domain-containing protein 7 isoform 1 [Homo sapiens]	347585.0076	257089.0947	389752.805	304203.5682	327065.0985	122617.9195	0	294440.0795	243556.6364
COMMD8	COMM domain-containing protein 8 [Homo sapiens]	463881.3097	809361.7357	820137.0413	650153.8825	911252.5236	833713.9639	963385.4291	527805.204	670981.3134
COMMD9	COMM domain-containing protein 9 isoform 1 [Homo sapiens]	2506864.636	2742657.551	3168763.701	2733954.615	2913223.568	1542767.755	2074114.261	1367371.989	2396135.504
COMT	catechol O-methyltransferase isoform MB-COMT [Homo sapiens]	7098619.101	7236641.356	7612526.229	7286365.369	8294788.308	7746934.3	7714718.717	6330279.312	6887039.158
COMTD1	catechol O-methyltransferase domain-containing protein 1 [Homo sapiens]	415490.1079	307392.3453	598999.4633	423146.1397	547407.1786	459056.4348	307394.2156	161379.3855	419776.0131
COPA	coatomer subunit alpha isoform 1 [Homo sapiens]	1695894.343	1513761.437	2115991.805	1455701.508	1977743.932	1666374.003	1599276.186	1274441.985	1474613.159
COPB1	coatomer subunit beta [Homo sapiens]	1486631.895	1650993.556	2075359.925	1603424.964	2142303.886	1774982.17	1779794.381	1544196.916	1794239.005
COPB2	coatomer subunit beta' [Homo sapiens]	2017624.807	2157934.663	2424704.367	2267390.11	2426071.767	2354751.803	2152703.858	1848018.107	2098408.923
COPE	coatomer subunit epsilon isoform a [Homo sapiens]	4365433.468	5945514.792	6114797.917	5218607.68	5839822.989	5351793.012	4180081.664	4336059.906	5606888.526
COPG1	coatomer subunit gamma-1 [Homo sapiens]	1727979.548	1871613.31	2270456.184	1833066.144	2133780.535	1850798.76	1750650.476	1458439.558	1950209.736
COPG2	coatomer subunit gamma-2 [Homo sapiens]	91173.58113	61411.80956	80838.34091	102349.9892	81881.62618	130395.3117	158606.6808	30727.56524	120699.2846
COPRS	coordinator of PRMT5 and differentiation stimulator [Homo sapiens]	629151.0872	594099.1163	0	347868.9856	0	458579.4651	144710.9477	275541.6628	0
COPS2	COP9 signalosome complex subunit 2 isoform 1 [Homo sapiens]	936727.0095	1095103.245	1276973.057	1034760.099	1356044.534	920617.4	650144.45	687778.8037	1160315.483
COPS3	COP9 signalosome complex subunit 3 isoform 1 [Homo sapiens]	1755041.633	1530128.517	1766342.376	1517930.939	2305989.828	1325266.101	1524906.66	1543858.648	1881845.794
COPS4	COP9 signalosome complex subunit 4 isoform 1 [Homo sapiens]	1295289.778	1423387.476	2036865.741	1755307.047	2007451.131	1733056.625	1533844.053	1129582.965	1622098.65
COPS5	COP9 signalosome complex subunit 5 [Homo sapiens]	1601231.632	1236654.488	1533754.329	1466478.718	1467558.949	1651876.858	1234721.874	1363785.296	1337276.376
COPS6	COP9 signalosome complex subunit 6 [Homo sapiens]	1538280.635	1797356.489	1742542.452	1964943.859	1844853.236	2034097.826	1932259.371	1281214.036	1992636.859
COPS7A	COP9 signalosome complex subunit 7a [Homo sapiens]	1076587.398	1155574.95	1045692.692	1442678.186	815135.5153	1343749.95	1488788.37	827111.1732	1111504.612
COPS7B	COP9 signalosome complex subunit 7b [Homo sapiens]	426302.8059	10552762.02	466100.5084	9444418.772	403109.2128	509579.1911	8989909.682	7848268.019	529549.8094
COPS8	COP9 signalosome complex subunit 8 isoform 1 [Homo sapiens]	4781645.286	5125592.653	5533363.205	5117346.632	5446159.249	5259084.075	4979279.5	4089140.447	6274474.754
COPZ1	coatomer subunit zeta-1 isoform 1 [Homo sapiens]	9499883.695	10546387.9	11637328.56	10574349.55	10573851.84	10791092.85	7101231.057	9118580.33	10192235.89
COQ3	hexaprenyldihydroxybenzoate methyltransferase, mitochondrial [Homo sapiens]	0	0	0	0	0	0	11441681.23	0	2560172.36
COQ5	2-methoxy-6-polyprenyl-1,4-benzoquinol methylase, mitochondrial precursor	39452.16119	368224.6222	0	29118.09728	0	23955.01562	0	0	0
COQ9	ubiquinone biosynthesis protein COQ9, mitochondrial precursor [Homo sapien:	235451.2146	259478.6679	295047.1489	275991.6091	344054.253	321094.6703	276236.4722	146846.2463	303360.9196
CORO1B	coronin-1B [Homo sapiens]	1471973.139	1561549.434	1832494.05	1499388.818	1775050.375	1536391.833	1409625.318	1227123.78	1454690.817
CORO1C	coronin-1C isoform a [Homo sapiens]	1465445.466	2100259.42	2211084.209	2033135.174	2258968.773	2051769.897	1796874.929	1746374.827	2439376.798
CORO2A	coronin-2A [Homo sapiens]	0	0	0	0	0	0	0	0	0
CORO7-PAM16	CORO7-PAM16 protein [Homo sapiens]	362083.6785	344955.6562	704428.894	309968.0244	571635.5487	274295.2599	269430.3356	244286.9604	212216.724
COTL1	coactosin-like protein [Homo sapiens]	11755841.33	14184119.48	15087202.93	10744938.07	15085048.67	15776901.88	15171727.27	12014939.92	10536077.56
COX1	cytochrome c oxidase subunit I [Homo sapiens]	5347461.765	2338253.629	3705671.553	3612759.47	3837623.148	4563203.242	0	1104278.794	2091457.206
COX11	cytochrome c oxidase assembly protein COX11, mitochondrial isoform 1 [Homo	222408.535	326307.3717	475361.7235	417078.6249	535644.6239	351297.8507	218403.0618	0	205101.3667
COX15	cytochrome c oxidase assembly protein COX15 homolog isoform 1 [Homo sapien:	648022.4149	592045.3791	747327.7465	622244.442	1045682.108	529298.2154	641127.3556	495576.1571	640919.4377
COX17	cytochrome c oxidase copper chaperone [Homo sapiens]	13151294.34	11306155.09	15652542.13	13111530.97	9260555.619	12317463.69	11339694.5	11488502.75	13176480.27
COX2	cytochrome c oxidase subunit II [Homo sapiens]	19726383.08	22427148.21	25708158.21	22999385.18	29545421.62	24344427.81	23974144.64	18724767.28	23364083.35
COX20	cytochrome c oxidase protein 20 homolog [Homo sapiens]	4010206.154	3156890.396	5597609.308	2518992.894	4264890.89	2528312.217	3220100.337	2334551.338	3861612.592
COX4I1	cytochrome c oxidase subunit 4 isoform 1, mitochondrial precursor [Homo sap	13218958.55	15361821.65	16129422.7	14819177.45	15846369.12	14827241.86	13972732.65	11761894.37	14744458.64
COX5A	cytochrome c oxidase subunit 5A, mitochondrial precursor [Homo sapiens]	8661183.044	6476603.924	12686174.61	11741791.14	11548531.07	13238343.19	13444546.84	12718339.44	14729064.26
COX5B	cytochrome c oxidase subunit 5B, mitochondrial precursor [Homo sapiens]	9613190.225	11651919.76	9994565.67	10987632.93	10771228.26	11255598.53	11796150.98	9947274.52	12042919.12
COX6A1	cytochrome c oxidase subunit 6A1, mitochondrial [Homo sapiens]	8003505.7	7189218.118	11855861.39	3206867.961	9235243.079	5683925.153	6830732.232	0	9931002.797
COX6B1	cytochrome c oxidase subunit 6B1 [Homo sapiens]	19765434	20536820.22	25399993.98	23680589.79	25629280.38	20888742.17	21591067.13	17722862.66	22264632.34
COX6C	cytochrome c oxidase subunit 6C proprotein [Homo sapiens]	1898006.653	2414946.938	894453.0889	1481522.249	685786.9464	1541032.293	1700475.572	2197827.365	0
COX7A2	cytochrome c oxidase subunit 7A2, mitochondrial precursor [Homo sapiens]	12670897.79	17581883.64	15918912.69	17592489.24	17099652.13	18762257.98	17926023.7	11193156.93	16652718.54
COX7A2L	cytochrome c oxidase subunit 7A-related protein, mitochondrial [Homo sapier	2614092.286	2589177.686	3247343.033	2340587.349	3442737.812	2719371.451	2245404.038	2048445.541	2194021.513
COX7C	cytochrome c oxidase subunit 7C, mitochondrial precursor [Homo sapiens]	8646925.688	0	9822305.813	9043495.844	11227516.38	9104269.9	10067660.07	7292531.259	10500543.46
CPD	carboxypeptidase D isoform 1 precursor [Homo sapiens]	58112.89208	1009578.905	58934.62072	46286.24983	56105.41857	562756.3894	31861.68113	0	60603.92516
CPLX1	complexin-1 [Homo sapiens]	457049.9141	397468.5147	620522.5285	467887.2096	587401.0441	497103.0359	0	0	0
CPNE1	copine-1 isoform b [Homo sapiens]	1156185.681	1237122.565	1519703.958	1264012.645	0	0	1275609.837	845541.6325	0
CPNE1	copine-1 isoform a [Homo sapiens]	1156185.681	1237122.565	1519703.958	1264012.645	0	0	1275609.837	845541.6325	0
CPNE3	copine-3 [Homo sapiens]	1620122.349	1533630.783	2060178.125	1738502.559	1788100.961	1878281.549	1762001.151	1486386.984	1888305.067
CPNE8	copine-8 [Homo sapiens]	240107.6304	296137.4203	364952.2883	376852.2934	160114.7251	276064.8132	323766.1094	261122.8499	228115.5201
CPOX	coproporphyrinogen-III oxidase, mitochondrial precursor [Homo sapiens]	216155.4122	216168.6489	269742.449	253673.9929	278074.0149	285853.4996	299926.0355	215120.3679	145569.785
CPPED1	calcineurin-like phosphoesterase domain-containing protein 1 isoform a [Hor	0	401927.983	489841.8547	0	603831.7001	279153.6557	0	147071.3996	439735.4961
CPS1	carbamoyl-phosphate synthase [ammonia], mitochondrial isoform a precursor	5882814.094	6541832.986	7547198.446	6475198.505	8644879.433	7656930.263	7049337.527	5553636.286	5625507.591
CPSF1	cleavage and polyadenylation specificity factor subunit 1 [Homo sapiens]	107894.533	89040.47777	103521.8492	107748.2446	127035.429	80248.70717	66621.49589	58959.60672	72161.12237
CPSF2	cleavage and polyadenylation specificity factor subunit 2 [Homo sapiens]	235074.2038	1342206.006	291860.7961	277941.9223	302099.3413	255089.3951	267050.8682	221478.5309	223860.6834
CPSF3	cleavage and polyadenylation specificity factor subunit 3 [Homo sapiens]	332822.0252	309334.0127	375736.284	339359.6303	381664.6482	316545.0958	317979.138	251064.488	308645.3336
CPSF3L	integrator complex subunit 11 isoform 1 [Homo sapiens]	0	0	0	0	0	0	109467.3472	107767.3739	0
CPSF4	cleavage and polyadenylation specificity factor subunit 4 isoform 1 [Homo s	278364.7944	516403.2581	268109.4484	110326.406	401690.6824	171153.5115	246754.0155	102987.8287	891852.4587
CPSF6	cleavage and polyadenylation specificity factor subunit 6 [Homo sapiens]	1467877.737	2318353.067	2723887.077	2357220.256	2566482.404	2349850.663	2305783.956	1975877.112	2884192.501
CPSF7	cleavage and polyadenylation specificity factor subunit 7 isoform 1 [Homo s	713427.9272	793685.8897	958782.0164	804329.4128	1098684.885	766315.6415	838288.7349	683931.0354	871961.7492
CPT1A	carnitine O-palmitoyltransferase 1, liver isoform isoform 1 [Homo sapiens]	1434247.982	1500267.494	2015032.141	1487271.079	2190564.527	1545624.719	1398631.888	1003377.199	1553293.809
CPT2	carnitine O-palmitoyltransferase 2, mitochondrial precursor [Homo sapiens]	266433.4111	256456.803	1219753.806	411788.1692	921113.6518	301883.3533	388217.196	230399.2648	290684.727
CRADD	death domain-containing protein CRADD [Homo sapiens]	111408.2717	0	165984.7374	177458.2464	179546.1304	0	0	153348.0354	49844.95109
CRCP	DNA-directed RNA polymerase III subunit RPC9 isoform a [Homo sapiens]	188538.6607	224054.7549	360694.8684	268181.6198	233428.8981	344858.0628	305932.4321	196511.7325	256794.7012
CREB1	cyclic AMP-responsive element-binding protein 1 isoform B [Homo sapiens]	0	0	0	0	0	0	425718.0487	0	317024.5844
CREBBP	CREB-binding protein isoform a [Homo sapiens]	2419.123963	51791.48279	1338.458368	0	0	24377.30857	0	8621.660468	3062.306878
CREG1	protein CREG1 precursor [Homo sapiens]	1900849.388	2702580.744	3697029.612	2462158.664	3161567.164	2307093.388	2034908.367	4685993.379	3026269.358
CRELD2	cysteine-rich with EGF-like domain protein 2 isoform a precursor [Homo sap:	391278.2435	382585.6793	228965.0465	178909.9085	222024.7529	419122.6243	408370.7785	416926.609	401567.1699
CRIP1	cysteine-rich protein 1 [Homo sapiens]	25409305.4	13903686.54	26759070.36	9958924.844	9151417.597	9373338.778	5856317.336	9916209.358	6124691.158
CRIP2	cysteine-rich protein 2 isoform 2 [Homo sapiens]	309542.662	411661.2466	476917.0671	210494.3549	366834.8148	449288.9288	209876.3924	0	378064.25
CRK	adapter molecule crk isoform a [Homo sapiens]	1751548.948	1787673.832	2003605.51	1814586.134	1916316.458	1939371.228	1863169.111	1522158.132	1781515.729
CRKL	crk-like protein [Homo sapiens]	2569753.965	2827387.315	2773380.27	2218934.896	3360261.878	2982858.007	2919029.776	2464800.305	2949886.907
CRLF3	cytokine receptor-like factor 3 [Homo sapiens]	246760.4118	147093.0109	448385.0987	255401.9199	417313.1911	286945.0064	293929.5448	265130.5239	200994.4124
CRNKL1	crooked neck-like protein 1 [Homo sapiens]	58451.91266	95010.59053	126031.5102	82100.2618	114052.0377	114688.5085	100173.1877	67107.02614	83365.53496
CRTAP	cartilage-associated protein precursor [Homo sapiens]	812921.6739	829289.7609	862926.9398	903222.3466	857016.7683	918022.195	741422.7422	563184.0902	819788.9217
CRTC3	CREB-regulated transcription coactivator 3 isoform a [Homo sapiens]	576325.7233	422800.2956	252360.9167	479485.6981	1402677.384	284692.6619	465848.3396	168916.1855	106262.3176
CRYZ	quinone oxidoreductase isoform a [Homo sapiens]	829985.3413	916919.2835	1158136.202	966630.4488	1446751.698	700379.281	601629.3155	891345.8868	1188902.311
CRYZL1	quinone oxidoreductase-like protein 1 [Homo sapiens]	165581.3095	159284.4266	452311.7242	210227.8542	430991.2242	212461.2503	235251.4098	288727.7315	124923.2202
CS	citrate synthase, mitochondrial precursor [Homo sapiens]	6033571.451	7168754.519	8263660.706	7169705.938	7775558.963	7486479.975	7713626.525	6585981.4	8224700.194
CSDE1	cold shock domain-containing protein E1 isoform 4 [Homo sapiens]	18965.99731	1726994.511	2108897.76	13799.088	0	0	0	1484150.226	1741196.259
CSE1L	exportin-2 isoform 1 [Homo sapiens]	3084560.187	3626727.294	4030116.947	3687888.509	3903345.681	3561427.063	3522203.381	2880447.763	4011597.813
CSK	tyrosine-protein kinase CSK [Homo sapiens]	776634.1016	945485.6218	711664.4164	958550.036	1063387.145	960819.2661	921533.5238	820182.9646	1021386.225
CSNK1A1	casein kinase I isoform alpha isoform 2 [Homo sapiens]	742598.8006	836790.1822	0	906369.0688	1147619.931	0	896061.6083	786405.307	809413.2318
CSNK1D	casein kinase I isoform delta isoform 1 [Homo sapiens]	0	0	0	0	0	0	343570.8109	242906.2111	0
CSNK1E	casein kinase I isoform epsilon [Homo sapiens]	279054.9182	224608.0135	291674.4584	0	416938.7265	0	0	77022.42082	270753.5355
CSNK1G3	casein kinase I isoform gamma-3 isoform 4 [Homo sapiens]	0	47676.77941	53859.21324	84208.07873	0	50973.88848	35201.42678	0	55149.0999
CSNK2A1	casein kinase II subunit alpha isoform a [Homo sapiens]	2267983.687	2467562.796	2807464.818	2612645.637	3083334.586	2695428.619	2533853.192	2077315.173	2407252.29
CSNK2A2	casein kinase II subunit alpha' [Homo sapiens]	889169.1333	1111889.61	1472016.714	1468857.235	1534717.128	1625545.954	1418126.265	1236216.801	1729079.629
CSNK2B	casein kinase II subunit beta [Homo sapiens]	6411757.626	6582967.671	6400487.402	5332967.892	7947922.183	6193541.515	6163703.265	5145252.845	7213000.297
CSPG4	chondroitin sulfate proteoglycan 4 precursor [Homo sapiens]	0	21509.1042	31090.06048	22250.7925	26162.02548	44516.15113	35349.1564	11367.9571	17397.16552

CSRP1	cysteine and glycine-rich protein 1 isoform 1 [Homo sapiens]	9616332.943	11252451.45	13077919.82	10843995.65	11672275.99	10680069.63	10535839.27	6491915.641	12787106.21
CSTB	cystatin-B [Homo sapiens]	39394834	37758295.5	52216813.18	45335354.18	44092090.05	45561180.13	38451596.66	40498684.45	42121275.47
CSTF1	cleavage stimulation factor subunit 1 [Homo sapiens]	615657.2045	874802.6382	869701.4986	914990.1683	995952.6782	918155.7296	863278.8394	608103.0704	927698.7042
CSTF2	cleavage stimulation factor subunit 2 [Homo sapiens]	1086901.958	1015756.6	1390781.985	1332397.557	1322648.292	1281592.216	982306.4574	944176.8847	1057347.172
CSTF3	cleavage stimulation factor subunit 3 isoform 1 [Homo sapiens]	405135.0009	426498.8539	524292.857	468852.9619	536594.9217	410144.4864	360440.4712	376605.0477	444623.5022
CTAGE5	cTAGE family member 5 isoform 1 [Homo sapiens]	126037.4143	118599.5936	144335.8997	113820.2354	156173.4749	121979.3452	109231.8937	0	116501.9243
CTBP1	C-terminal-binding protein 1 isoform 1 [Homo sapiens]	876069.0878	877737.7163	1277790.255	1146685.359	1301240.536	1158002.956	1113111.629	789887.0694	1111396.152
CTBP2	C-terminal-binding protein 2 isoform 2 [Homo sapiens]	275249.8095	380524.7322	357181.3832	246210.5928	454630.2174	338582.2326	291684.6362	367797.5817	271667.3178
CTCF	transcriptional repressor CTCF isoform 1 [Homo sapiens]	43887.66359	22770.09615	49462.61608	281469.5211	35116.99359	180152.6137	103113.5331	233330.5137	0
CTDP1	RNA polymerase II subunit A C-terminal domain phosphatase isoform 1 [Homo s	0	100021.5822	114775.4579	90963.98845	113526.858	87209.65353	87676.72011	73603.76087	96065.0163
CTDSPL2	CTD small phosphatase-like protein 2 [Homo sapiens]	0	0	0	0	0	0	80525.70621	24894.24223	67352.50282
CTH	cystathionine gamma-lyase isoform 1 [Homo sapiens]	287076.9337	392872.9257	0	338413.5929	441630.0512	365167.9643	257605.905	206691.252	269166.3472
CTNNA1	catenin alpha-1 [Homo sapiens]	729731.8501	855365.0151	1059990.491	722638.0955	967879.0584	847490.2838	497720.4788	472255.3125	598396.3836
CTNNAL1	alpha-catulin [Homo sapiens]	116193.336	358671.8095	150033.1677	81859.24974	130815.3589	67957.86317	89897.31209	77939.12663	85083.68703
CTNNB1	catenin beta-1 [Homo sapiens]	145914.907	58845.36139	85773.78764	87448.64945	202291.0907	111857.0862	17743.37738	40813.69011	8033.836764
CTNNBL1	beta-catenin-like protein 1 [Homo sapiens]	450006.4546	516509.2546	601439.2123	451265.5053	583010.9652	508422.4463	513543.5885	428037.2583	549257.9683
CTNND1	catenin delta-1 isoform 1ABC [Homo sapiens]	564710.3208	627512.2727	686143.8427	621037.1933	677459.0246	643807.4543	544685.905	483637.4891	596981.0201
CTNND2	catenin delta-2 [Homo sapiens]	0	0	0	0	0	0	0	0	0
CTPS1	CTP synthase 1 [Homo sapiens]	2234894.064	2615608.333	3051297.619	2401837.052	3271150.961	2587791.473	2456046.114	2054918.952	2880752.953
CTPS2	CTP synthase 2 [Homo sapiens]	187000.6229	250043.0389	278870.4332	274419.5782	403588.369	278389.4056	269147.8933	224433.6476	268998.4011
CTR9	RNA polymerase-associated protein CTR9 homolog [Homo sapiens]	160975.7952	232005.0178	240333.1794	194094.5507	225021.5425	194282.9784	110954.041	188798.6486	178054.7827
CTSA	lysosomal protective protein isoform a precursor [Homo sapiens]	319728.2167	585785.0599	303851.411	154594.0747	221394.3108	311780.7821	515155.7966	504683.3403	141851.1458
CTSB	cathepsin B preproprotein [Homo sapiens]	1078142.352	630256.0031	755927.5579	630034.6923	1237004.169	1140609.095	887537.5741	599844.5194	724054.0833
CTSC	dipeptidyl peptidase 1 isoform a preproprotein [Homo sapiens]	1827480.01	1782629.507	1732515.634	1792937.274	1768061.282	1374556.512	1898018.232	956535.9678	1901662.239
CTSD	cathepsin D preproprotein [Homo sapiens]	2751961.421	3029199.06	3313380.393	3181035.796	3308494.621	2893073.621	2806113.417	2120117.833	2878384.035
CTSL1	cathepsin L1 isoform 1 preproprotein [Homo sapiens]	1647932.415	901626.9639	1021841.919	1210998.148	1022247.285	996351.1604	1342102.229	704947.8811	832604.4725
CTSZ	cathepsin Z preproprotein [Homo sapiens]	610407.6647	115468.6693	594589.03	525887.449	217321.9896	207560.1608	198563.1432	303348.8229	0
CTTN	src substrate cortactin isoform a [Homo sapiens]	3079117.398	3503036.971	3833908.462	3674425.37	4014275.555	3809934.196	3491765.364	3215374.401	2073137.437
CTTNBP2NL	CTTNBP2 N-terminal-like protein [Homo sapiens]	88824.2808	89102.41304	81653.05072	92481.85326	87834.61413	46497.4271	0	67888.15036	69444.73406
CTU2	cytoplasmic tRNA 2-thiolation protein 2 isoform 1 [Homo sapiens]	46667.09631	0	70177.23361	70749.56831	0	92838.85383	45535.9071	0	73000.0847
CUL1	cullin-1 [Homo sapiens]	241959.2269	306094.524	379077.722	258203.3589	382180.5269	334581.6754	315180.2874	231991.5841	381034.6737
CUL2	cullin-2 isoform a [Homo sapiens]	482834.1927	617077.4717	624116.3819	450011.8622	614653.0962	539588.3705	556289.5905	476610.4908	496718.5917
CUL3	cullin-3 isoform 3 [Homo sapiens]	145373.3182	255120.5385	180112.1775	209140.7278	259700.1251	210359.6397	2786588.332	164379.1742	225613.6782
CUL4A	cullin-4A isoform 1 [Homo sapiens]	466566.9137	503117.0934	571599.5773	518771.3896	590037.8765	503412.5757	500784.2279	367394.7896	479287.6675
CUL4B	cullin-4B isoform 1 [Homo sapiens]	375252.6913	313605.4691	503312.5389	285016.9079	535220.3292	303644.9797	287923.6968	259014.6431	351397.7673
CUL5	cullin-5 [Homo sapiens]	115800.5189	115314.6053	148355.1105	129676.3311	143534.8201	253499.8524	202229.7242	94476.68615	206191.2774
CUTA	protein CutA isoform 1 [Homo sapiens]	20040054.25	19723060.46	25932935.05	23782929.93	23338879.56	25797156.65	21985947.73	17831176.82	23720371.6
CUTC	copper homeostasis protein cutC homolog [Homo sapiens]	518659.707	851486.4236	1184549.541	1170797.747	1220715.266	883625.1188	769929.2935	564874.613	1392402.514
CUX1	protein CASP isoform d [Homo sapiens]	14936.71174	22254.11509	20834.02236	25876.16144	29928.94871	31430.0335	16666.61547	31423.0938	17712.56208
CWC15	spliceosome-associated protein CWC15 homolog [Homo sapiens]	446248.7623	870313.1705	668087.1066	455146.1967	0	525858.4713	435990.8689	924535.3811	638297.5799
CWC22	pre-mRNA-splicing factor CWC22 homolog [Homo sapiens]	113773.4204	73250.34444	135186.949	100676.3	134333.5751	75713.18853	85583.97045	93833.64307	105175.4623
CWC27	peptidyl-prolyl cis-trans isomerase CWC27 homolog [Homo sapiens]	278113.8829	429422.7016	294927.2356	150392.514	416250.8272	296417.075	276045.9569	349514.7727	239667.2719
CWF19L1	CWF19-like protein 1 [Homo sapiens]	274782.2766	285430.1736	284231.2927	306289.0606	333803.3502	311780.7821	294436.185	258005.8405	299399.7237
CXADR	coxsackievirus and adenovirus receptor isoform 1 precursor [Homo sapiens]	527265.4191	600719.6855	558215.5683	493709.5408	591504.7009	577033.9365	540399.0689	486864.7507	187766.7182
CXorf38	uncharacterized protein CXorf38 [Homo sapiens]	362680.4969	429548.9988	520084.6086	534559.4553	462969.0035	625524.6316	424021.021	349014.572	617716.8427
CXorf56	UPF0428 protein CXorf56 isoform 1 [Homo sapiens]	300196.7277	440492.125	242445.7936	175523.1183	368041.8123	234424.5436	132188.2132	67718.62946	78908.36384
CXXC1	cpG-binding protein isoform 1 [Homo sapiens]	0	0	0	0	0	0	18141.91774	0	23054.08589
CYB561D2	cytochrome b561 domain-containing protein 2 [Homo sapiens]	1488570.62	1519326.811	702555.2308	1213903.092	284989.1515	1467168.438	1661999.212	521966.1827	1005710.899
CYB5A	cytochrome b5 isoform 1 [Homo sapiens]	6161603.109	6354105.795	7770184.914	5901778.863	7034298.574	6400829.012	6444203.491	5617061.623	5712061.621
CYB5B	cytochrome b5 type B [Homo sapiens]	22689848.6	22681114	30669951.85	24822729.83	33133384.79	27227169.48	27931795.02	25789097.75	31602654.83
CYB5R1	NADH-cytochrome b5 reductase 1 [Homo sapiens]	560478.2306	651850.7866	1017388.709	713829.0866	1032069.338	534402.7386	600338.796	469037.2528	928588.796
CYB5R3	NADH-cytochrome b5 reductase 3 isoform 3 [Homo sapiens]	4842122.429	5368573.051	6558110.929	4665645.78	6240763.319	5542806.363	5267011.876	3533773.566	5023748.485
CYBA	cytochrome b-245 light chain [Homo sapiens]	1006201.213	1693678.363	970881.7903	2170994.629	0	1577362.435	1092188.546	0	1064564.866
CYC1	cytochrome c1, heme protein, mitochondrial [Homo sapiens]	3233729.691	3738641.131	4374695.936	3785105.263	4706830.589	3546868.764	4059246.339	3167173.383	3997322.396
CYCS	cytochrome c [Homo sapiens]	22629612.27	27640634.83	32279784.32	24086563.31	33467724.58	26604321.58	28680765.75	22609310.56	30029089.08
CYFIP1	cytoplasmic FMR1-interacting protein 1 isoform a [Homo sapiens]	293495.8864	289198.7215	374865.5303	296628.2495	322438.5344	351273.1948	274508.439	226418.9476	295756.1837
CYP20A1	cytochrome P450 20A1 [Homo sapiens]	0	0	0	0	0	0	12300.50801	37862.26143	23494.01524
CYP51A1	lanosterol 14-alpha demethylase isoform 1 precursor [Homo sapiens]	689986.9251	1233147.932	704535.9302	786764.405	1272329.166	708531.9145	697597.6436	859046.1687	1369133.187
CYTB	cytochrome b [Homo sapiens]	828509.4509	1558239.92	0	709436.2355	539498.6071	776653.2054	1410476.607	1048351.589	1903848.277
DAB2	disabled homolog 2 isoform 1 [Homo sapiens]	159026.2567	124698.56	129634.2957	136306.8535	134670.4163	45784.45291	167304.236	134663.7371	128965.1891
DAD1	dolichyl-diphosphooligosaccharide--protein glycosyltransferase subunit DAD1	16526339.37	25868905.7	19899676.44	19559487.02	22512404.64	20114060.65	7976368.364	16822119.58	37286221.52
DAG1	dystroglycan preproprotein [Homo sapiens]	351137.4603	285137.5929	607685.9151	270550.7714	0	62468.89583	32195.52885	289164.3205	72950.41439
DAGLB	sn1-specific diacylglycerol lipase beta isoform 1 [Homo sapiens]	266437.1396	312220.9912	356191.1319	262603.456	335808.3937	358791.8385	323350.9306	231181.0234	338423.0604
DAK	bifunctional ATP-dependent dihydroxyacetone kinase/FAD-AMP lyase (cyclizing	473022.3727	722267.0092	624881.9321	606856.5446	659966.7326	644807.9772	641701.8522	506143.3665	638215.1668
DAP3	28S ribosomal protein S29, mitochondrial isoform 1 [Homo sapiens]	981552.0107	1625502.41	1613485.645	1267886.85	1767301.326	1380379.514	1269188.29	1106105.336	1197817.968
DAPK3	death-associated protein kinase 3 [Homo sapiens]	0	0	0	0	0	0	66222.3354	42481.26733	72793.06807
DARS	aspartate--tRNA ligase, cytoplasmic [Homo sapiens]	2534269.108	2690137.515	3571263.485	2849344.112	3713812.988	2830705.677	2672049.024	2414836.503	2781980.721
DARS2	aspartate--tRNA ligase, mitochondrial [Homo sapiens]	221116.8941	267788.0153	262732.9483	280626.9424	327604.3916	287617.3833	287944.5169	243023.0685	286312.8586
DAXX	death domain-associated protein 6 isoform b [Homo sapiens]	38913.25287	242783.8466	216996.6684	349533.1614	91318.59992	226179.5787	221761.1436	79823.14057	252485.6925
DAZAP1	DAZ-associated protein 1 isoform b [Homo sapiens]	5386288.484	5369985.641	6686767.786	4915861.653	6601698.502	5535932.433	5467073.277	4016989.542	5245737.837
DBI	acyl-CoA-binding protein isoform 4 [Homo sapiens]	11951935.93	17534447.16	10905853.69	30602012.77	16642060.04	29116758.22	19141501.34	10566561.91	12714460.83
DBN1	drebrin isoform a [Homo sapiens]	1437456.06	1710465.679	1147377.461	1388235.972	1943702.798	1447060.922	1664626.813	1394285.458	1829389.679
DBNL	drebrin-like protein isoform c [Homo sapiens]	2320593.975	2564652.099	124748.4477	2565933.999	3149066.024	2757352.96	2370039.085	1922381.547	2808884.752
DBR1	lariat debranching enzyme [Homo sapiens]	287368.5876	345375.3009	909755.5216	791376.0551	902658.1714	778962.2027	1952805.255	1001031.364	930336.1773
DBT	lipoamide acyltransferase component of branched-chain alpha-keto acid dehyo	0	121774.1933	0	286986.7318	95008.82807	253281.6269	257136.4429	112560.5706	130754.0465
DCAF11	DDB1- and CUL4-associated factor 11 isoform 1 [Homo sapiens]	0	0	0	0	0	0	16223.24192	0	447934.8488
DCAF13	DDB1- and CUL4-associated factor 13 [Homo sapiens]	219147.3501	259061.1417	190380.1344	272875.4315	397719.0394	277475.5088	235386.3062	174603.1935	168606.8017
DCAF16	DDB1- and CUL4-associated factor 16 [Homo sapiens]	275068.7929	487082.6607	356932.2179	488236.6809	248874.0714	459724.4679	393209.6321	372789.1821	472104.9357
DCAF7	DDB1- and CUL4-associated factor 7 [Homo sapiens]	699268.288	356271.7549	951251.8178	815134.7532	772337.8633	857355.308	794365.8145	617834.518	717611.5784
DCAF8	DDB1- and CUL4-associated factor 8 [Homo sapiens]	299255.7787	286041.6526	285751.3285	290105.3198	307019.9111	319654.2963	332855.5862	253461.5403	162431.1579
DCAKD	dephospho-CoA kinase domain-containing protein [Homo sapiens]	201893.5319	0	240114.0907	218979.451	217510.0245	0	0	204672.8235	212843.902
DCD	dermcidin preproprotein [Homo sapiens]	7301224.716	2806620.386	0	1823010.874	265416.1995	3763138.059	5144765.67	2981271.968	4715001.409
DCK	deoxycytidine kinase [Homo sapiens]	319276.9813	579998.1395	503731.2789	0	647212.7883	274035.5936	316324.2194	0	158673.4753
DCP1A	mRNA-decapping enzyme 1A [Homo sapiens]	281388.3303	252805.7414	232005.3833	263155.1914	406121.6384	304718.5624	253813.0338	102864.505	257843.3187
DCPS	m7GpppX diphosphatase [Homo sapiens]	1529217.88	1686956.771	2039087.282	1726093.013	2052690.211	1745211.287	1451987.242	1471711.176	1725997.983
DCTD	deoxycytidylate deaminase isoform a [Homo sapiens]	504193.345	351534.2804	777851.0874	471075.4511	855949.9257	690858.2562	665142.7939	331531.1769	652773.9342
DCTN1	dynactin subunit 1 isoform 1 [Homo sapiens]	148117.9087	177601.8684	198838.6693	178329.9578	208630.9025	189656.6234	163043.1775	129202.7036	0
DCTN1	dynactin subunit 1 isoform 3 [Homo sapiens]	148117.9087	177601.8684	198838.6693	178329.9578	208630.9025	189656.6234	163043.1775	129202.7036	0
DCTN2	dynactin subunit 2 isoform 1 [Homo sapiens]	2615290.418	2715175.247	3327617.969	2845050.582	3242498.282	3178464.908	2579815.437	2356054.975	2471513.988
DCTN3	dynactin subunit 3 isoform 1 [Homo sapiens]	744090.4886	569951.6664	836151.7122	508445.6809	102525.3421	955049.0762	897151.2338	400266.8537	446834.5263
DCTN4	dynactin subunit 4 isoform a [Homo sapiens]	336427.0283	656664.4467	480537.9665	416641.163	471965.9685	445131.2939	253293.4157	274345.9939	677007.6957
DCTN5	dynactin subunit 5 isoform 1 [Homo sapiens]	0	0	0	0	0	0	0	0	0
DCTPP1	dCTP pyrophosphatase 1 [Homo sapiens]	4096776.217	3560748.902	4530170.194	4221655.705	3424734.619	3720863.89	3490613.61	3200976.844	3681666.438
DCUN1D1	DCN1-like protein 1 [Homo sapiens]	679062.4977	1702696.29	1491654.869	616446.729	833437.0625	1122044.368	889881.2126	654691.715	746387.4716
DCUN1D5	DCN1-like protein 5 [Homo sapiens]	487025.2932	1104340.496	1255218.221	1134219.691	1168197.802	826821.8417	1167386.231	635455.6421	1352117.163
DCXR	L-xylulose reductase isoform 1 [Homo sapiens]	424311.7724	647524.3353	598428.1491	696797.2819	549436.1742	644657.5221	869621.7762	723799.4755	601139.8755
DDA1	DET1- and DDB1-associated protein 1 [Homo sapiens]	530500.0686	526268.5	458448.7843	374499.9878	715158.9706	492065.3137	326335.152	216649.8861	536504.1324
DDAH1	N(G),N(G)-dimethylarginine dimethylaminohydrolase 1 isoform 1 [Homo sapiens	399415.6434	646410.2387	677837.5229	634023.807	677317.6904	665764.9685	715929.7384	569016.5255	500923.2192
DDAH2	N(G),N(G)-dimethylarginine dimethylaminohydrolase 2 [Homo sapiens]	287819.847	263569.2696	439328.0983	410810.6042	542178.4896	453366.1022	297610.375	180862.5118	493153.1927
DDB1	DNA damage-binding protein 1 [Homo sapiens]	1569970.88	1755041.925	2045602.256	1964419.65	2091708.117	1886948.053	1850811.221	1522400.71	1688573.228
DDB2	DNA damage-binding protein 2 [Homo sapiens]	415099.955	265537.4715	443683.6518	252603.1096	640061.1583	346063.9177	317056.3377	178886.5744	204524.2512
DDI2	protein DDI1 homolog 2 [Homo sapiens]	178217.1914	427265.279	282008.2109	287612.7466	503401.9524	465242.9467	424235.8373	474451.8571	421443.3289
DDOST	dolichyl-diphosphooligosaccharide--protein glycosyltransferase 48 kDa subun	4136668.961	4826545.426	5281450.856	5202993.833	5469958.714	5647613.649	5251810.887	4224950.776	4804020.031
DDR2	discoidin domain-containing receptor 2 precursor [Homo sapiens]	0	0	0	0	0	0	491858.2748	0	504804.2557
DDRGK1	DDRGK domain-containing protein 1 precursor [Homo sapiens]	672486.6405	651530.0525	729494.1524	691782.4583	995642.7628	744763.1051	611220.6405	419479.4465	667493.5439
DDT	D-dopachrome decarboxylase [Homo sapiens]	45247005.07	42273816.62	52314524.43	50495324	57122380.9	51358014.17	74071295.69	44859363.74	46478510.14
DDX1	ATP-dependent RNA helicase DDX1 [Homo sapiens]	2203795.462	1910948.445	2885430.184	2141831.131	2752377.238	2344370.609	2069471.814	1467035.949	1834225.567
DDX10	probable ATP-dependent RNA helicase DDX10 [Homo sapiens]	133779.5086	134259.9211	120293.4905	139675.6474	218843.9057	128880.3215	121347.9652	84961.14608	151181.2787
DDX17	probable ATP-dependent RNA helicase DDX17 isoform 3 [Homo sapiens]	3223163.034	3457919.089	4094320.463	3349406.692	4379095.2	3503895.8	3184430.991	2753182.385	3562342.568
DDX18	ATP-dependent RNA helicase DDX18 [Homo sapiens]	2374033.259	1238630.694	3259391.12	1245786.893	1746715.034	1361228.897	1240449.09	1100856.96	1173095.684
DDX19A	ATP-dependent RNA helicase DDX19A [Homo sapiens]	454641.0388	635218.816	666165.2825	330935.7124	886859.2194	878101.585	721947.9408	505440.1141	696453.2121
DDX19B	ATP-dependent RNA helicase DDX19B isoform 1 [Homo sapiens]	279977.6541	426688.9768	453467.8442	364536.5389	435071.6853	422752.4962	396466.0025	326095.505	338168.119
DDX20	probable ATP-dependent RNA helicase DDX20 [Homo sapiens]	200275.4768	281940.8525	281943.7799	248017.2617	331627.5059	236119.4797	178927.4516	210705.5106	277448.1853
DDX21	nucleolar RNA helicase 2 isoform 1 [Homo sapiens]	3226445.78	3851560.627	3906431.405	3822946.182	4061963.984	3985351.107	4028102.818	3493610.92	4408585.303
DDX23	probable ATP-dependent RNA helicase DDX23 [Homo sapiens]	417096.6741	511251.8319	586922.9927	569132.6389	549524.9863	608357.2168	574053.2024	505911.0363	508972.974
DDX24	ATP-dependent RNA helicase DDX24 [Homo sapiens]	245818.4699	245635.6195	318262.2177	265675.4296	334452.1345	290397.806	264998.5688	189452.4072	274310.2294
DDX27	probable ATP-dependent RNA helicase DDX27 [Homo sapiens]	648569.1419	701554.5	610548.5033	652902.4843	606024.6113	704948.341	771302.2749	443512.2044	780260.4237
DDX31	probable ATP-dependent RNA helicase DDX31 isoform 1 [Homo sapiens]	43108.3453	38593.1263	92502.29457	64322.18842	140958.8682	72250.92313	69722.3314	25411.76122	95668.85287
DDX39A	ATP-dependent RNA helicase DDX39A [Homo sapiens]	3603060.606	4420990.532	5192767.977	4173133.197	5421002.101	4557641.606	4229184.023	3527387.152	4792386.534
DDX39B	spliceosome RNA helicase DDX39B [Homo sapiens]	5573169.158	5798515.833	7379948.994	6367069.557	7694718.356	6461533.224	6061594.552	4991366.775	6148986.971

DDX3X	ATP-dependent RNA helicase DDX3X isoform 1 [Homo sapiens]	6214786.3	5901824.449	8063634.136	6503420.645	7931005.328	6546259.714	6119775.718	5494612.92	5811407.78
DDX41	probable ATP-dependent RNA helicase DDX41 [Homo sapiens]	52243.41566	77942.63227	212074.549	73962.97612	72591.72612	66815.3101	83257.4458	75230.92091	99536.00087
DDX42	ATP-dependent RNA helicase DDX42 [Homo sapiens]	384507.8306	474641.1408	495345.6571	366990.4992	548814.3406	375307.3411	401875.8979	442153.4877	392812.8461
DDX46	probable ATP-dependent RNA helicase DDX46 [Homo sapiens]	647413.6435	619200.4497	792513.3251	625866.5409	840339.7946	650645.0838	618361.0669	528581.4305	1007052.75
DDX47	probable ATP-dependent RNA helicase DDX47 isoform 1 [Homo sapiens]	849534.5255	823418.1755	1043176.171	876834.4404	1118794.9	808583.9436	872290.1734	785279.1527	902793.7686
DDX49	probable ATP-dependent RNA helicase DDX49 [Homo sapiens]	201860.0455	336444.278	293467.097	150135.3431	275073.723	271494.3092	231016.9903	167085.8002	239070.7818
DDX5	probable ATP-dependent RNA helicase DDX5 [Homo sapiens]	5906774.951	8976905.203	8035362.011	6520699.797	8145756.406	8922458.508	6942865.18	5917468.444	9476897.974
DDX50	ATP-dependent RNA helicase DDX50 [Homo sapiens]	233960.4787	211292.4536	279163.8274	271642.214	256718.8959	255162.0973	217968.3673	231197.2559	244307.4521
DDX51	ATP-dependent RNA helicase DDX51 [Homo sapiens]	52054.93892	90873.06224	87549.139	40451.18478	132617.3102	114273.1685	42255.47759	150438.1916	119991.4117
DDX52	probable ATP-dependent RNA helicase DDX52 [Homo sapiens]	137110.6464	271461.7986	283269.4865	288775.1576	186252.5212	295921.5483	197932.6961	132181.6433	299381.3218
DDX54	ATP-dependent RNA helicase DDX54 isoform 1 [Homo sapiens]	296485.6139	259947.2582	359714.53	253966.1307	350923.0012	358397.0702	259102.3381	272301.8837	329581.0378
DDX55	ATP-dependent RNA helicase DDX55 [Homo sapiens]	183925.7666	219860.2793	347461.5688	125357.333	242542.1836	226826.0809	188970.4097	85658.97583	195025.8034
DDX56	probable ATP-dependent RNA helicase DDX56 isoform 1 [Homo sapiens]	483355.5226	537279.7704	738620.9309	511881.3665	726526.0991	469862.333	454019.2087	338854.1583	498920.0848
DDX58	probable ATP-dependent RNA helicase DDX58 [Homo sapiens]	0	0	0	0	0	0	20310.51855	24564.88373	23468.70714
DDX59	probable ATP-dependent RNA helicase DDX59 [Homo sapiens]	72819.23452	261512.784	152073.2513	217744.4206	170183.0261	212255.9785	144024.6233	43613.01752	0
DDX6	probable ATP-dependent RNA helicase DDX6 [Homo sapiens]	1762728.374	1714017.609	2292451.947	1847192.838	2536908.503	1893678.387	1590725.929	1356250.739	1834831.911
DDX60	probable ATP-dependent RNA helicase DDX60 [Homo sapiens]	6044.945078	19533.02355	41314.45168	6725.050776	24875.26596	25094.35877	12430.9805	12900.50285	16107.5282
DECR1	2,4-dienoyl-CoA reductase, mitochondrial precursor [Homo sapiens]	873483.9992	468292.9687	816308.7031	555304.3033	497687.9696	654287.1735	717270.6331	837332.7922	836858.1938
DECR2	peroxisomal 2,4-dienoyl-CoA reductase [Homo sapiens]	328356.7009	622173.5036	258969.1718	248442.1121	239049.3645	240854.0374	311026.4206	523494.3411	988796.1308
DEGS1	sphingolipid delta(4)-desaturase DES1 [Homo sapiens]	896047.8649	560952.2	886854.3562	1148657.453	1422337.017	460398.6842	488055.3921	817172.2579	1450102.979
DEK	protein DEK isoform 1 [Homo sapiens]	3852025.193	4859682.145	5225885.136	4576793.814	5051632.936	4534659.638	4607381.841	3946636.468	5030418.45
DENND4C	DENN domain-containing protein 4C [Homo sapiens]	14046.98052	21769.21835	23344.70959	15033.98638	45299.72106	15949.36324	26097.0113	18629.11958	18822.25952
DENND5B	DENN domain-containing protein 5B [Homo sapiens]	0	28555.90805	0	20716.51884	20882.26288	26906.00094	0	0	0
DENR	density-regulated protein [Homo sapiens]	2827152.09	1917290.439	3704387.78	2809582.801	3267381.175	3344059.16	3065557.77	2555387.416	1903871.288
DEPDC7	DEP domain-containing protein 7 isoform 1 [Homo sapiens]	94301.9844	61234.94968	152847.8108	95827.12153	142025.568	93992.81222	57712.77676	44946.90972	45609.10048
DERA	putative deoxyribose-phosphate aldolase [Homo sapiens]	495349.9382	735093.9465	733814.0218	691106.7719	777883.8801	556560.5993	592823.3438	514438.1468	1194357.392
DERL1	derlin-1 isoform a [Homo sapiens]	1032756.254	503746.5282	1074442.629	1807778.577	1567632.717	776672.507	1557097.73	1106521.95	530413.1338
DERL2	derlin-2 [Homo sapiens]	1465937.763	1797126.193	2406232.737	1628699.158	1980989.868	2405187.702	1410635.264	1662744.974	0
DESI1	desumoylating isopeptidase 1 [Homo sapiens]	3779519.734	3489808.878	5475352.171	3370531.795	5710983.266	4778150.276	3284393.415	2920647.156	3761849.92
DFFA	DNA fragmentation factor subunit alpha isoform 1 [Homo sapiens]	309833.8483	827089.1905	492232.9382	509998.1998	422103.592	368379.4081	427103.6742	302195.6066	772254.5975
DFFA	DNA fragmentation factor subunit alpha isoform 2 [Homo sapiens]	309833.8483	827089.1905	492232.9382	509998.1998	422103.592	368379.4081	427103.6742	302195.6066	772254.5975
DFNA5	non-syndromic hearing impairment protein 5 isoform a [Homo sapiens]	223840.8997	140570.5472	169590.7296	141949.8301	168555.3499	223723.4416	208432.0791	176151.0419	250036.5865
DGCR14	protein DGCR14 [Homo sapiens]	90927.83084	76865.7395	67220.60302	86230.51713	89349.61842	88679.09233	69630.69619	115984.2512	56272.12465
DGKE	diacylglycerol kinase epsilon [Homo sapiens]	137206.8998	124563.413	106562.1362	171622.0193	130318.4106	158544.6027	179589.6498	136424.6304	46385.27476
DHCR24	delta(24)-sterol reductase precursor [Homo sapiens]	923865.3299	1226446.897	1144563.966	1075736.987	1345962.838	1171694.013	1116807.223	980421.2348	1057761.391
DHCR7	7-dehydrocholesterol reductase [Homo sapiens]	1155631.844	1437248.811	3800661.969	3976705.598	1155726.493	1593308.518	4357336.385	1769618.235	1485464.74
DHDDS	dehydrodolichyl diphosphate synthase isoform 2 [Homo sapiens]	59241.42561	0	108863.2214	30015.30971	82672.73929	27212.55006	0	0	0
DHFR	dihydrofolate reductase [Homo sapiens]	2670219.038	2778689.498	2646637.579	3022549.065	3266253.017	3207943.208	2878743.265	2104735.016	2561584.415
DHODH	dihydroorotate dehydrogenase (quinone), mitochondrial [Homo sapiens]	185046.1624	169403.6947	306795.3539	252540.5045	253129.9799	251491.432	306248.994	196498.414	239372.4768
DHPS	deoxyhypusine synthase isoform a [Homo sapiens]	1092468.879	1086191.644	1330152.49	968827.7411	1261629.907	1087004.801	941825.7073	935658.225	1057030.246
DHRS1	dehydrogenase/reductase SDR family member 1 [Homo sapiens]	69496.47821	138869.6034	0	614694.2464	115327.25	376963.2613	264879.0416	240012.1178	205106.6034
DHRS11	dehydrogenase/reductase SDR family member 11 precursor [Homo sapiens]	0	0	0	0	0	0	0	0	0
DHRS4	dehydrogenase/reductase SDR family member 4 [Homo sapiens]	1179544.593	860937.8739	1219317.577	641313.7635	1222830.725	899623.8314	945366.9148	734833.8148	1096425.028
DHRS7	dehydrogenase/reductase SDR family member 7 precursor [Homo sapiens]	784940.4509	754048.6111	954003.5715	1010005.837	1172956.069	630948.5474	1017142.771	1156579.417	1036326.428
DHRS7B	dehydrogenase/reductase SDR family member 7B [Homo sapiens]	365569.8528	529026.5763	406102.6744	335300.5061	379692.2623	431612.1441	420396.0212	435376.5456	330937.8898
DHTKD1	probable 2-oxoglutarate dehydrogenase E1 component DHKTD1, mitochondrial [I	92133.15358	57751.50856	110681.0231	148723.7248	146337.9515	69796.78943	62680.03485	103792.2646	108794.2791
DHX15	putative pre-mRNA-splicing factor ATP-dependent RNA helicase DHX15 [Homo sa	2591759.128	2908173.97	3314744.873	2854022.592	3363524.832	3083247.107	2755238.629	2290649.919	2962888.069
DHX16	putative pre-mRNA-splicing factor ATP-dependent RNA helicase DHX16 isoform	367766.7482	375666.0425	478275.1316	372441.51	513477.9204	436815.5305	397290.692	327257.3684	375981.0115
DHX29	ATP-dependent RNA helicase DHX29 [Homo sapiens]	123442.3921	122668.7448	138784.8374	128072.1522	138962.2906	130122.0191	116139.5183	97976.66562	124980.4071
DHX30	putative ATP-dependent RNA helicase DHX30 isoform 1 [Homo sapiens]	464654.5553	565576.1852	622650.2453	503549.975	614264.0994	572852.0081	547089.8837	400009.2655	516962.5
DHX32	putative pre-mRNA-splicing factor ATP-dependent RNA helicase DHX32 [Homo sa	59819.15063	28074.96385	71833.70711	65430.76778	90017.61611	61471.76151	70811.84205	38747.14592	75374.6836
DHX33	putative ATP-dependent RNA helicase DHX33 isoform 1 [Homo sapiens]	111049.1717	0	100434.0205	0	57071.50948	110808.2743	106287.1992	0	46364.5842
DHX34	probable ATP-dependent RNA helicase DHX34 [Homo sapiens]	0	16113.62043	34617.99726	18633.57135	32843.05758	18776.38984	0	0	0
DHX36	probable ATP-dependent RNA helicase DHX36 isoform 1 [Homo sapiens]	410081.0295	399642.3604	547330.3235	436465.5261	537091.7393	678076.5811	427000.0681	426892.2302	442925.7043
DHX37	probable ATP-dependent RNA helicase DHX37 [Homo sapiens]	96804.17537	33059.2883	165386.7539	100574.0861	156044.5163	109656.3767	99981.09465	87669.86465	63193.10191
DHX38	pre-mRNA-splicing factor ATP-dependent RNA helicase PRP16 [Homo sapiens]	94805.50658	122446.9919	128478.9198	126063.5168	113482.0392	135154.8985	110697.3217	82823.28838	112595.3784
DHX40	probable ATP-dependent RNA helicase DHX40 isoform 1 [Homo sapiens]	85030.41784	83753.04461	98689.14396	85741.39677	60007.50317	96130.67416	74511.79705	65644.9807	76500.7802
DHX57	putative ATP-dependent RNA helicase DHX57 [Homo sapiens]	52181.31475	90926.57244	53337.64516	90576.38204	86798.97819	51896.9328	36996.26419	31113.72787	57827.9164
DHX8	ATP-dependent RNA helicase DHX8 [Homo sapiens]	221014.1408	177861.5211	241619.0899	178169.5138	239385.8386	174741.5671	131250.2484	113340.1831	120994.4499
DHX9	ATP-dependent RNA helicase A [Homo sapiens]	3620089.594	3881498.906	4623118.943	3803982.259	4683912.939	4057373.151	3747939.537	3310107.649	3761387.818
DIABLO	diablo homolog, mitochondrial isoform 1 precursor [Homo sapiens]	1710826.257	1628910.06	3143920.059	1313922.998	1577290.416	1705155.152	1593898.33	1417439.953	1701353.946
DIAPH1	protein diaphanous homolog 1 isoform 1 [Homo sapiens]	532618.0795	678741.4639	718127.9114	621554.2162	686563.9832	657696.1921	649115.9286	544496.7509	619701.3542
DIAPH2	protein diaphanous homolog 2 isoform 156 [Homo sapiens]	48489.01891	34558.63222	29146.55408	77027.85363	46700.40314	60397.79141	34840.02959	0	50185.08915
DIAPH3	protein diaphanous homolog 3 isoform a [Homo sapiens]	86257.89693	93759.25729	112260.5953	86693.43655	148577.1431	115732.9845	77430.88716	45402.95627	84574.88462
DICER1	endoribonuclease Dicer isoform 1 [Homo sapiens]	49325.55981	34964.45802	48773.36531	32355.01089	47629.74899	61919.12691	47307.03489	29762.02929	38594.95534
DIDO1	death-inducer obliterator 1 isoform c [Homo sapiens]	60652.04089	67042.24692	111824.5524	115717.6413	103991.3332	74572.536	92437.70052	103114.982	93639.00445
DIEXF	digestive organ expansion factor homolog [Homo sapiens]	188884.4508	238726.8122	285759.2471	203953.4842	261827.522	216013.8652	185364.1614	137740.7416	242783.9508
DIMT1	probable dimethyladenosine transferase [Homo sapiens]	605112.354	784380.9752	865584.143	767254.2181	966250.5087	789527.3282	796856.6812	713870.2168	866412.3517
DIP2A	disco-interacting protein 2 homolog A isoform a [Homo sapiens]	25878.00093	26254.13687	23126.98867	26899.57407	23590.3052	26153.12424	21265.03889	0	21003.04456
DIP2B	disco-interacting protein 2 homolog B [Homo sapiens]	109892.7244	116601.8583	140954.93	125950.2918	668658.1422	148514.0029	111893.7725	90373.1868	488614.4145
DIRC2	disrupted in renal carcinoma protein 2 [Homo sapiens]	307333.2551	0	533522.6122	373112.3878	500607.8942	449869.1837	456285.727	328276.4005	440599.9898
DIS3	exosome complex exonuclease RRP44 isoform a [Homo sapiens]	392754.1719	550259.2924	544200.6848	548479.419	518485.2294	590435.6253	493458.1152	472537.0266	490386.0544
DKC1	H/ACA ribonucleoprotein complex subunit 4 isoform 1 [Homo sapiens]	1513568.802	1515651.744	2026912.407	1719994.421	1780320.833	1748154.911	1739241.749	1244789.049	1602826.717
DLAT	dihydrolipoyllysine-residue acetyltransferase component of pyruvate dehydro	1237964.532	1109527.182	1624903.74	1501933.73	1756150.72	1554046.242	1545824.415	1338875.7	1513240.766
DLD	dihydrolipoyl dehydrogenase, mitochondrial precursor [Homo sapiens]	2636459.819	2425234.489	3282368.461	2792251.655	3290956.473	2803862.608	2784860.54	2199036.055	2721120.829
DLG1	disks large homolog 1 isoform 2 [Homo sapiens]	96166.96503	107050.8032	40834.83458	108927.0066	203543.6891	116952.0291	122549.6359	131660.6589	31753.22467
DLGAP4	disks large-associated protein 4 isoform a [Homo sapiens]	0	0	0	0	0	0	0	1963.168554	77016.06063
DLGAP5	disks large-associated protein 5 isoform a [Homo sapiens]	65771.48905	68366.93198	47793.7218	56557.76118	137613.8441	61566.44967	47668.29323	0	1467861.107
DLST	dihydrolipoyllysine-residue succinyltransferase component of 2-oxoglutarate	1713743.823	1993503.135	2218993.563	1941283.745	2164393.674	2492028.57	2094603.748	1744499.897	2036668.426
DMAP1	DNA methyltransferase 1-associated protein 1 [Homo sapiens]	63501.65573	75365.51724	81163.31091	117454.2625	160907.9623	126320.4052	149919.0463	96540.29957	4189410.138
DMD	dystrophin Dp427m isoform [Homo sapiens]	36670.21747	75218.57136	49616.9123	39974.65361	57448.07641	38781.75187	38587.21623	32693.39557	38684.63174
DNAAF2	protein kintoun isoform 1 [Homo sapiens]	24407.89461	17270.92892	0	0	26503.64114	39601.86059	12777.36448	42101.73735	0
DNAJA1	dnaJ homolog subfamily A member 1 [Homo sapiens]	3666027.714	3601446.386	4667430.529	4103054.882	4817384.148	4236494.183	3965258.383	3318131.315	3184944.703
DNAJA2	dnaJ homolog subfamily A member 2 [Homo sapiens]	1843541.758	1969435.034	2499553.417	2204214.877	2465187.067	1805245.981	1945673.147	1457354.459	2030721.617
DNAJA3	dnaJ homolog subfamily A member 3, mitochondrial isoform 1 [Homo sapiens]	794365.2462	0	991590.102	844981.401	998427.8593	974006.6754	936193.3206	758704.6276	509022.6447
DNAJA4	dnaJ homolog subfamily A member 4 isoform 1 [Homo sapiens]	250270.5162	928550.0434	340052.9614	924331.1112	304615.1083	741391.0668	102208.6517	724906.6702	779020.1698
DNAJB1	dnaJ homolog subfamily B member 1 [Homo sapiens]	1568851.494	2038991.099	1766568.902	1823657.226	2093444.354	2265884.927	2163972.619	1940911.238	1886927.455
DNAJB11	dnaJ homolog subfamily B member 11 precursor [Homo sapiens]	917093.6585	1147150.828	958531.7503	1300293.292	1170146.084	1047075.176	1219375.59	627312.3217	898567.1735
DNAJB12	dnaJ homolog subfamily B member 12 [Homo sapiens]	279496.1452	448787.4945	349733.7317	421913.8127	448720.3434	417206.1097	395230.1058	428571.8074	514020.1263
DNAJB4	dnaJ homolog subfamily B member 4 [Homo sapiens]	569971.4806	547703.6555	588821.8447	598726.9581	663175.318	431735.2247	367324.6398	417413.8716	600833.1685
DNAJB6	dnaJ homolog subfamily B member 6 isoform a [Homo sapiens]	410413.2762	718395.5919	0	726055.6397	0	693879.4559	564851.1489	499184.5432	0
DNAJC1	dnaJ homolog subfamily C member 1 precursor [Homo sapiens]	0	146122.1145	165891.9342	127273.3684	160006.9494	96179.35223	104970.3573	104152.6073	0
DNAJC10	dnaJ homolog subfamily C member 10 isoform 1 precursor [Homo sapiens]	96134.33832	134633.2391	154815.3167	152695.9636	167944.804	149079.0568	114526.7596	104477.0067	112214.5813
DNAJC11	dnaJ homolog subfamily C member 11 [Homo sapiens]	309352.6106	403002.0411	589218.2962	592359.8681	558137.6261	614616.9681	554634.1197	380425.4713	570586.1895
DNAJC13	dnaJ homolog subfamily C member 13 [Homo sapiens]	42299.44721	46317.12785	40229.0697	145488.8122	48244.31568	55870.75836	55010.83176	44620.256	27985.57906
DNAJC15	dnaJ homolog subfamily C member 15 [Homo sapiens]	0	0	0	0	0	0	0	170596.8846	229347.6923
DNAJC16	dnaJ homolog subfamily C member 16 precursor [Homo sapiens]	0	0	0	0	0	0	14988.24116	0	14733.39477
DNAJC17	dnaJ homolog subfamily C member 17 [Homo sapiens]	245365.5449	434465.4647	241368.222	528873.4065	559882.5449	382583.0262	520202.8267	500702.9812	527817.8894
DNAJC2	dnaJ homolog subfamily C member 2 isoform 1 [Homo sapiens]	288419.5884	311383.9036	350825.9612	352594.8594	332825.8693	362611.2197	339736.0394	266511.1359	310362.1331
DNAJC21	dnaJ homolog subfamily C member 21 isoform 2 [Homo sapiens]	0	0	0	0	0	0	191210.7299	105996.2994	273594.897
DNAJC21	dnaJ homolog subfamily C member 21 isoform 1 [Homo sapiens]	0	0	0	0	0	0	191210.7299	105996.2994	273594.897
DNAJC3	dnaJ homolog subfamily C member 3 precursor [Homo sapiens]	125796.7647	349899.5014	384604.3673	100339.1419	235281.5046	227307.3383	233258.461	120034.4027	163051.8699
DNAJC5	dnaJ homolog subfamily C member 5 [Homo sapiens]	1028238.841	1112973.678	1660560.153	1209538.483	1617624.915	663134.9459	638333.7161	1137492.1	1329924.892
DNAJC7	dnaJ homolog subfamily C member 7 isoform 1 [Homo sapiens]	1049931.3	1052630.531	1553329.537	1213972.723	1408762.149	1060594.727	1282867.481	1070786.416	1161866.686
DNAJC8	dnaJ homolog subfamily C member 8 [Homo sapiens]	1018095.359	961947.5993	1203100.326	905219.4148	1084206.701	711984.2373	676022.1784	803177.7592	591114.6013
DNAJC9	dnaJ homolog subfamily C member 9 [Homo sapiens]	1504503.812	1869189.823	1921337.612	1468552.433	1763142.458	1545378.973	1316881.582	1276710.576	1602404.335
DNASE2	deoxyribonuclease-2-alpha precursor [Homo sapiens]	938083.3958	549394.3047	1277669.779	769296.4988	1255493.276	571958.2135	999649.2891	819636.1432	810579.6554
DNLZ	DNL-type zinc finger protein [Homo sapiens]	0	0	0	0	0	0	0	0	0
DNM1L	dynamin-1-like protein isoform 1 [Homo sapiens]	212288.2982	0	237850.1873	256036.2351	0	265400.7	227876.388	179053.8927	264526.9367
DNM1L	dynamin-1-like protein isoform 4 [Homo sapiens]	212288.2982	0	237850.1873	256036.2351	0	265400.7	227876.388	179053.8927	264526.9367
DNM2	dynamin-2 isoform 1 [Homo sapiens]	409599.7202	402761.0584	475933.1003	214240.6909	469279.9419	431794.6126	224549.3395	331414.2618	221168.7668
DNM2	dynamin-2 isoform 2 [Homo sapiens]	409599.7202	402761.0584	475933.1003	214240.6909	469279.9419	431794.6126	224549.3395	331414.2618	221168.7668
DNMBP	dynamin-binding protein [Homo sapiens]	25766.17369	30293.23554	22738.25745	28071.46131	78543.65243	0	21255.2566	8950.646259	0
DNMT1	DNA (cytosine-5)-methyltransferase 1 isoform a [Homo sapiens]	293777.9872	336481.2581	416161.3164	323396.2044	380356.6826	345340.5197	286384.5224	228810.0318	318979.534
DNPEP	aspartyl aminopeptidase [Homo sapiens]	309686.7148	351026.47	240690.4221	354286.2131	389367.7668	346410.3716	367810.8652	411743.8412	495162.6304
DNPH1	2'-deoxynucleoside 5'-phosphate N-hydrolase 1 isoform 1 [Homo sapiens]	10490544.54	7868593.951	9537430.928	11851936.3	9286748.846	12922862.39	11058750.03	9351015	11500175.13
DNTTIP1	deoxynucleotidyltransferase terminal-interacting protein 1 [Homo sapiens]	180305.0802	101155.4584	224901.0433	167130.3103	179409.504	178108.1911	0	0	0
DNTTIP2	deoxynucleotidyltransferase terminal-interacting protein 2 [Homo sapiens]	289212.9185	259191.2643	426829.1654	363125.6542	380719.8508	386413.4309	360092.987	274378.6128	345846.9817
DOCK1	dedicator of cytokinesis protein 1 [Homo sapiens]	0	0	0	0	0	0	41063.4355	5587.076887	6664.355198
DOCK2	dedicator of cytokinesis protein 2 [Homo sapiens]	2207475.489	0	910766.996	33601299.28	48179874.79	118849.2572	38315014.57	0	42041131.14

DOCK5	dedicator of cytokinesis protein 5 [Homo sapiens]	26481.11097	191777.7879	42569.1643	21828.05295	36982.6225	38314.17785	25606.70328	21715.17844	52964.04538
DOCK7	dedicator of cytokinesis protein 7 isoform 1 [Homo sapiens]	11525.50101	9364.219021	30094.06336	14104.42024	26725.05429	12920.73637	0	0	0
DOHH	deoxyhypusine hydroxylase [Homo sapiens]	900750.8459	1069609.952	1558334.28	911130.3154	1485549.975	905665.4328	867488.5798	901828.2146	1156677.189
DOLPP1	dolichyldiphosphatase 1 isoform a [Homo sapiens]	971828.0492	1127093.478	1176908.631	0	1267996.443	1132865.082	1100111.534	691317.0349	1094589.717
DOM3Z	decapping and exoribonuclease protein [Homo sapiens]	0	0	0	0	0	0	45698.70192	0	193163.2657
DOPEY1	protein dopey-1 isoform b [Homo sapiens]	0	0	0	0	0	0	0	0	0
DPAGT1	UDP-N-acetylglucosamine--dolichyl-phosphate N-acetylglucosaminephosphotrans	0	0	0	0	0	0	132418.8198	137943.1356	0
DPCD	protein DPCD [Homo sapiens]	204746.9255	187404.2728	229514.7113	212240.0925	220630.14	178282.8863	253028.1975	220557.7975	160480.9625
DPF2	zinc finger protein ubi-d4 [Homo sapiens]	335826.0858	402758.3417	400549.5755	386791.8715	431681.5656	434671.9664	305807.2782	247077.123	465892.0818
DPH1	diphthamide biosynthesis protein 1 [Homo sapiens]	181747.8201	158626.2417	213412.2237	103753.7141	0	142220.9981	127627.3503	86056.86976	198930.3392
DPH2	diphthamide biosynthesis protein 2 isoform a [Homo sapiens]	241572.4988	150395.9006	310183.3744	250756.1366	331826.9897	131810.5694	249995.1541	221938.5233	246170.8063
DPH3	DPH3 homolog isoform 1 [Homo sapiens]	1216073.257	0	1762468.875	1291042.424	0	1334512.528	0	0	0
DPH5	diphthine synthase isoform a [Homo sapiens]	378932.2574	315406.4072	534197.889	311449.3218	415881.6048	332665.0933	418907.33	422438.2376	381989.2467
DPM1	dolichol-phosphate mannosyltransferase [Homo sapiens]	1495063.633	2274864.078	1909738.401	1830078.215	1883509.623	1806303.79	2032565.597	1981624.344	2496261.528
DPM3	dolichol-phosphate mannosyltransferase subunit 3 isoform 1 [Homo sapiens]	0	0	0	0	0	0	0	0	0
DPP3	dipeptidyl peptidase 3 isoform 1 [Homo sapiens]	1249395.916	1200520.624	1319201.676	1368513.922	1385351.998	1445445.263	1152856.163	973613.3765	1154248.496
DPP7	dipeptidyl peptidase 2 preproprotein [Homo sapiens]	100231.3106	98565.35524	114553.8195	70041.63054	104338.1908	89298.16367	140365.126	250537.6916	154946.5075
DPP9	dipeptidyl peptidase 9 [Homo sapiens]	166524.2856	161114.9016	277467.0135	230970.1062	239917.6935	214968.979	193046.3751	164176.3196	182027.1044
DPY30	protein dpy-30 homolog [Homo sapiens]	10180047.25	8839007.497	12139182.97	7272796.105	12104116.85	5649639.546	6903123.032	8185952.762	9335408.881
DPYSL2	dihydropyrimidinase-related protein 2 isoform 1 [Homo sapiens]	902888.2664	733830.1148	982610.8246	756862.0512	1119638.064	1024503.938	895301.8164	731647.9557	856681.002
DPYSL3	dihydropyrimidinase-related protein 3 isoform 1 [Homo sapiens]	183591.0477	100910.0927	192371.106	137100.014	190699.393	134688.0887	89298.9139	0	86791.64535
DR1	protein Dr1 [Homo sapiens]	2986336.577	3900160.495	5478780.946	0	5187349.238	2861516.791	3160565.229	2279623.141	4518998.268
DRAP1	dr1-associated corepressor [Homo sapiens]	1642326.282	2407364.163	2303338.507	2279559.651	2192547.368	2498253.549	2443280.992	1149112.035	2868853.51
DRG1	developmentally-regulated GTP-binding protein 1 [Homo sapiens]	1787485.396	2775220.749	2430354.518	2895487.996	2725369.052	3044832.325	1974903.439	1718447.646	2125523.526
DRG2	developmentally-regulated GTP-binding protein 2 [Homo sapiens]	721006.0082	878786.6434	890617.1987	942428.4013	1045729.792	920002.8352	857470.1874	675980.5906	1004599.033
DROSHA	ribonuclease 3 isoform 1 [Homo sapiens]	26595.37511	41156.21526	0	37919.46455	25223.37338	49850.75933	29205.5597	22172.46828	35932.52228
DSC1	desmocollin-1 isoform Dsc1a preproprotein [Homo sapiens]	96564.69879	52547.20889	73163.09111	26696.91748	333547.8673	94443.32216	79252.41039	164811.491	79497.06229
DSCC1	sister chromatid cohesion protein DCC1 [Homo sapiens]	46752.54779	0	59749.65441	0	225240.4118	137176.8125	0	0	0
DSCR3	Down syndrome critical region protein 3 [Homo sapiens]	232705.9237	147716.5681	278914.7849	197263.003	296113.8816	238923.0686	114704.4135	165829.3306	128647.149
DSG1	desmoglein-1 preproprotein [Homo sapiens]	370960.0913	252578.4927	196627.2663	184402.3864	629406.8559	351345.9052	231819.1911	263794.7788	192822.7585
DSG2	desmoglein-2 preproprotein [Homo sapiens]	421869.5158	442058.5369	466184.3786	389283.453	529015.7777	443209.4994	428076.9225	336277.6741	505657.589
DSN1	kinetochore-associated protein DSN1 homolog isoform 1 [Homo sapiens]	165168.9561	170167.2679	215103.9	76696.98134	84440.27369	253169.151	0	42069.43247	66929.5323
DSP	desmoplakin isoform I [Homo sapiens]	271652.2889	264053.7507	304112.4236	256036.4729	318582.0289	382961.2675	311803.9211	217276.9283	350640.7129
DST	bullous pemphigoid antigen 1 isoform 2 [Homo sapiens]	16830.94824	13554.30109	27333.34055	18311.58491	22342.84246	15421.4001	14738.07466	10408.36589	14630.6319
DSTN	destrin isoform a [Homo sapiens]	11094001.27	11485590.3	10199685.5	12831885.88	11573602.18	12978677.26	12423705.78	9835106.031	10739445.47
DTD1	D-tyrosyl-tRNA(Tyr) deacylase 1 [Homo sapiens]	771660.5227	0	1048675.051	1001304.406	1591228.415	998772.0745	937126.5812	540721.5936	0
DTL	denticleless protein homolog [Homo sapiens]	0	53396.06734	356757.9527	566823.2483	0	26674.42897	0	0	0
DTNBP1	dysbindin isoform a [Homo sapiens]	112484.1439	374506.5873	135471.4528	0	136800.2689	140201.4623	86960.00825	69247.7783	0
DTX3L	E3 ubiquitin-protein ligase DTX3L [Homo sapiens]	68266.70144	57595.78528	156869.3328	79194.0611	42308.60583	59543.58718	103247.1412	53760.71761	0
DTYMK	thymidylate kinase isoform 1 [Homo sapiens]	1675770.841	2057546.681	1710071.041	1672554.745	2177375.722	2075005.136	2220977.446	1674448.426	2093997.118
DUS1L	tRNA-dihydrouridine(16/17) synthase [NAD(P)(+)]-like [Homo sapiens]	58948.66626	59887.93414	99884.91133	0	95985.51601	0	0	80991.07207	63157.11453
DUS2L	tRNA-dihydrouridine(20) synthase [NAD(P)+]-like isoform 1 [Homo sapiens]	179013.0983	124232.4417	160369.7625	83202.50718	137211.4026	76889.97144	90018.71278	144952.2686	148583.2137
DUS3L	tRNA-dihydrouridine(47) synthase [NAD(P)(+)]-like isoform 1 [Homo sapiens]	166875.9115	478138.6168	280601.6341	253699.3193	240316.9735	312759.2492	278080.2582	245540.1557	496956.1252
DUSP12	dual specificity protein phosphatase 12 [Homo sapiens]	727595.1633	885375.8524	1096874.369	826257.0766	1078101.177	872374.5573	760842.2137	698677.1269	832165.7
DUSP3	dual specificity protein phosphatase 3 [Homo sapiens]	2275273.815	3567257.402	3324116.403	3457437.185	3692060.979	3311880.297	3652955.203	2416982.706	3321209.997
DUSP9	dual specificity protein phosphatase 9 [Homo sapiens]	448011.6227	498707.5366	607433.1613	344136.2473	457683.9052	589264.3152	219464.9798	214707.9637	378838.2851
DUT	deoxyuridine 5'-triphosphate nucleotidohydrolase, mitochondrial isoform 2	1631121.603	9779560.55	10690253.03	1445911.442	615024.3948	1710519.697	1500633.866	5180831.355	817427.6472
DUT	deoxyuridine 5'-triphosphate nucleotidohydrolase, mitochondrial isoform 1	1631121.603	9779560.55	10690253.03	1445911.442	615024.3948	1710519.697	1500633.866	5180831.355	817427.6472
DVL1	segment polarity protein dishevelled homolog DVL-1 [Homo sapiens]	0	0	0	0	0	0	15015.00825	0	13222.9741
DYNC1H1	cytoplasmic dynein 1 heavy chain 1 [Homo sapiens]	329901.9414	373451.3904	412386.0062	335401.3182	405612.5261	372163.8679	352035.6625	302612.8463	367758.6347
DYNC1I2	cytoplasmic dynein 1 intermediate chain 2 isoform 3 [Homo sapiens]	1535120.031	1374625.538	1791645.267	1569777.607	1817261.822	1700010.439	0	1081550.234	1249797.682
DYNC1LI1	cytoplasmic dynein 1 light intermediate chain 1 [Homo sapiens]	1553928.485	1594443.988	1976695.627	1777496.898	1999517.677	1724364.441	1683513.42	1556033.213	1743705.275
DYNC1LI2	cytoplasmic dynein 1 light intermediate chain 2 [Homo sapiens]	634305.0138	719492.1892	864186.1123	683044.2956	849281.8847	788025.6099	677819.1148	612274.4709	788707.3793
DYNLL1	dynein light chain 1, cytoplasmic [Homo sapiens]	31289090.8	31873837.34	47912750.66	35058159.66	46208999.06	18000096.23	17253741.09	36090291.03	32877328.11
DYNLL2	dynein light chain 2, cytoplasmic [Homo sapiens]	3329156.614	2964885.343	3273662.389	3461257.371	4477418.629	2945701.797	2853022.432	3841687.811	5520243.657
DYNLRB1	dynein light chain roadblock-type 1 [Homo sapiens]	12516379.49	10596160.49	11009010.16	9023182.6	16140805.99	38349133.97	16455876.82	9044314.189	15424663.01
DYNLT1	dynein light chain Tctex-type 1 [Homo sapiens]	2320809.995	2133760.559	2912093.615	2439775.345	2907295.041	2454120.708	2813545.6	2137954.434	2386718.045
DYNLT3	dynein light chain Tctex-type 3 [Homo sapiens]	4834689.371	4587282.032	5939694.046	1269715.161	6609058.361	5575857.389	5106875.85	4149725.629	9063371.529
DYRK1A	dual specificity tyrosine-phosphorylation-regulated kinase 1A isoform 1 [Ho	0	0	0	0	0	0	55496.89706	19323.67535	78522.95107
E2F4	transcription factor E2F4 [Homo sapiens]	0	0	0	0	0	0	0	0	0
EAF1	ELL-associated factor 1 [Homo sapiens]	0	0	0	0	0	0	219616.5928	0	371093.8479
EARS2	probable glutamate--tRNA ligase, mitochondrial [Homo sapiens]	142492.2295	61103.36657	180332.2567	167365.2301	145512.5181	191195.1594	154460.1824	97010.45652	0
EBNA1BP2	probable rRNA-processing protein EBP2 isoform 1 [Homo sapiens]	1909190.528	2271767.431	2720189.234	2664706.524	2836358.297	2685705.656	2567196.223	2189165.97	2597295.091
EBP	3-beta-hydroxysteroid-Delta(8),Delta(7)-isomerase [Homo sapiens]	9659393.044	24683541.89	18335064.81	27436977.5	22004855.43	18733318.2	17729601.53	18624301.91	18852492.04
ECD	protein SGT1 isoform 2 [Homo sapiens]	417670.7031	447032.2009	460685.4319	1138347.16	467383.4555	515523.8048	417458.5548	323517.6197	429572.0919
ECE1	endothelin-converting enzyme 1 isoform 1 [Homo sapiens]	128782.1382	148573.7953	174851.8875	180111.9802	178168.1984	165817.0982	132808.1415	140372.2727	107535.3632
ECE2	endothelin-converting enzyme 2 isoform A [Homo sapiens]	79545.88245	0	74256.51049	0	131346.333	123488.6923	87143.82517	0	107116.0284
ECH1	delta(3,5)-Delta(2,4)-dienoyl-CoA isomerase, mitochondrial precursor [Homo	3444845.708	3393643.778	4624269.961	2989381.298	4574740.362	3533703.695	3530817.671	2629418.428	3423671.797
ECHDC1	ethylmalonyl-CoA decarboxylase isoform 5 [Homo sapiens]	316721.6181	531643.9492	473842.5816	391054.4294	721622.5042	537187.9703	405763.617	0	378172.6536
ECHDC3	enoyl-CoA hydratase domain-containing protein 3, mitochondrial precursor [	391293.0647	235223.552	429679.8513	370021.1013	518717.903	317619.4003	0	134756.4009	247025.1444
ECHS1	enoyl-CoA hydratase, mitochondrial [Homo sapiens]	4165718.472	4727678.773	5805696.901	5219297.213	5654364.653	5628839.567	5124856.234	4209790.648	5283156.086
ECI1	enoyl-CoA delta isomerase 1, mitochondrial isoform 1 precursor [Homo sapier	2561398.365	3149307.999	4908780.792	3324924.912	3919858.969	3519284.126	3489008.585	3125551.814	4320041.173
ECI2	enoyl-CoA delta isomerase 2, mitochondrial isoform 2 [Homo sapiens]	2127205.553	2574002.744	2951644.068	3082419.792	3555411.857	3136146.653	2676248.822	2176397.324	2743230.471
ECSIT	evolutionarily conserved signaling intermediate in Toll pathway, mitochondr	549341.8361	425009.1424	1985169.259	435878.0005	428179.0719	439306.4924	453080.9074	1929461.11	403032.3083
EDC3	enhancer of mRNA-decapping protein 3 [Homo sapiens]	538115.8978	436474.7324	703991.0254	622392.2331	648915.6713	669346.326	572208.8315	500607.6814	616256.1702
EDC4	enhancer of mRNA-decapping protein 4 [Homo sapiens]	332312.0411	337470.1175	442425.4108	387738.2605	450988.2012	432587.6574	359530.3055	312652.7632	363900.0376
EDEM3	ER degradation-enhancing alpha-mannosidase-like protein 3 precursor [Homo s	0	0	33297.12778	51722.73454	41397.91032	30812.50873	0	0	0
EDF1	endothelial differentiation-related factor 1 isoform alpha [Homo sapiens]	6084772.243	6702648.853	7954979.354	8654895.011	5420057.011	7349960.754	5534191.262	5280988.261	6434372.677
EEA1	early endosome antigen 1 [Homo sapiens]	149976.6033	151310.9778	207652.6795	148228.0287	233160.0864	237467.0543	266957.274	139517.9453	156966.7047
EED	polycomb protein EED isoform a [Homo sapiens]	282986.5036	350680.6102	335500.5326	234695.8709	189600.2445	369682.4745	357979.8814	212905.1836	429879.4876
EEF1A1	elongation factor 1-alpha 1 [Homo sapiens]	127883333.3	133072914.7	113234340.6	109278348.2	128873658.2	150164414.4	127370569.6	130049855.2	147639785.5
EEF1A2	elongation factor 1-alpha 2 [Homo sapiens]	3430202.596	3597835.973	4977409.833	3598555.923	2515800.815	4421703.228	4008905.382	3177210.69	4006430.9
EEF1B2	elongation factor 1-beta [Homo sapiens]	49054075.85	60166078.89	67302368.02	57122006.56	68064032.89	59533436.6	59905985.82	53041948.43	62609513.1
EEF1D	elongation factor 1-delta isoform 2 [Homo sapiens]	1074046.56	1397719.681	1457723.08	1372558.377	1418549.216	1419258.981	1307797.775	7824285.287	11336125.14
EEF1D	elongation factor 1-delta isoform 4 [Homo sapiens]	1074046.56	1397719.681	1457723.08	1372558.377	1418549.216	1419258.981	1307797.775	7824285.287	11336125.14
EEF1D	elongation factor 1-delta isoform 5 [Homo sapiens]	1074046.56	1397719.681	1457723.08	1372558.377	1418549.216	1419258.981	1307797.775	7824285.287	11336125.14
EEF1E1	eukaryotic translation elongation factor 1 epsilon-1 isoform 1 [Homo sapier	8018213.979	7726113.894	9479543.094	9270016.536	9795826.628	9754629.255	9146566.334	7298045.201	6925889.755
EEF1G	elongation factor 1-gamma [Homo sapiens]	27077879.97	26651934.59	35950272.06	30942932.86	35537332.97	30142180.68	28831091.13	25176046.34	30416666.38
EEF2	elongation factor 2 [Homo sapiens]	21502290.82	22978245.49	24585075.92	23978456.23	25470433.1	25132464.7	23614192.44	19451794.67	25210734.05
EEFSEC	selenocysteine-specific elongation factor [Homo sapiens]	231068.5943	193214.3265	326708.1081	201527.7608	264394.0667	209370.1397	297160.0267	125402.9092	220017.2241
EFHD2	EF-hand domain-containing protein D2 [Homo sapiens]	2505223.564	2339753.506	3491464.811	2765555.185	3201225.912	2879623.618	2726874.569	2302038.558	2931753.568
EFR3A	protein EFR3 homolog A [Homo sapiens]	0	0	0	0	0	0	25517.29292	30711.87935	0
EFTUD1	elongation factor Tu GTP-binding domain-containing protein 1 isoform 1 [Hom	244631.4935	265656.2647	290739.5832	244362.3125	279021.8743	256187.6969	228345.6295	219248.2208	264414.1098
EFTUD2	116 kDa U5 small nuclear ribonucleoprotein component isoform a [Homo sapier	2144248.204	2350236.494	3020440.536	2396817.261	2867059.22	2382891.648	2296422.575	2032037.328	2688206.999
EGFR	epidermal growth factor receptor isoform a precursor [Homo sapiens]	564556.4288	618121.3793	780524.2738	551135.8113	769502.6411	690483.47	796018.4724	638013.6147	663142.3421
EGLN1	egl nine homolog 1 [Homo sapiens]	339132.9867	357842.3353	414777.2698	340048.8862	457330.3032	361372.2384	367298.4912	0	311521.954
EHBP1	EH domain-binding protein 1 isoform 1 [Homo sapiens]	19414.78736	24386.4153	24945.32297	18574.50182	0	19357.92046	28408.18089	0	23192.63321
EHBP1L1	EH domain-binding protein 1-like protein 1 [Homo sapiens]	62795.54234	36079.36987	79154.45058	53312.8634	112400.3248	61509.88178	51479.77921	38791.8192	58875.36043
EHD1	EH domain-containing protein 1 [Homo sapiens]	963812.9916	1166264.2	1227590.796	1078638.278	1378486.037	1055095.268	1085636.176	972765.6748	1002640.9
EHD2	EH domain-containing protein 2 [Homo sapiens]	259285.2917	295027.4129	350822.4762	340629.7621	429823.7926	693191.0089	626535.4116	588395.9138	301332.3025
EHD4	EH domain-containing protein 4 [Homo sapiens]	178822.3246	269790.2849	232460.5311	249721.3708	232403.8116	222785.9823	259092.9172	355593.0583	315890.1652
EHMT1	histone-lysine N-methyltransferase EHMT1 isoform 1 [Homo sapiens]	64635.11327	55328.0778	80518.34011	78684.09145	0	74411.84007	64285.41092	0	33821.93655
EHMT2	histone-lysine N-methyltransferase EHMT2 isoform a [Homo sapiens]	59484.76642	46756.20113	0	60612.70557	164187.4275	51384.77495	24824.88031	53526.58608	45480.20597
EI24	etoposide-induced protein 2.4 homolog isoform 1 [Homo sapiens]	217331.4951	163849.8482	644409.7098	267834.0943	627411.4796	241666.255	323156.7428	362765.8438	582936.9043
EIF1	eukaryotic translation initiation factor 1 [Homo sapiens]	6740509.374	12936889.75	10506866.05	9466722.572	10860140.92	7471528.178	8405545.426	13642653.31	8683727.663
EIF1AD	probable RNA-binding protein EIF1AD [Homo sapiens]	2397375.954	1247353.111	1199803.736	1291347.416	1136256.126	1271409.108	581316.454	1245155.331	
EIF1B	eukaryotic translation initiation factor 1b [Homo sapiens]	0	1236848.643	2198455.707	1054009.326	1996590.652	2413336.348	1751731.412	0	1576211.87
EIF2A	eukaryotic translation initiation factor 2A [Homo sapiens]	1891186.016	2386489.68	2430648.477	2106305.863	2377767.147	2431101.49	2368563.105	1962208.13	2815644.481
EIF2AK2	interferon-induced, double-stranded RNA-activated protein kinase isoform a	1098640.586	1297057.709	1466342.534	1323482.266	1493346.033	1301604.203	1291849.48	1074657.677	1340601.574
EIF2AK4	eukaryotic translation initiation factor 2-alpha kinase 4 [Homo sapiens]	22700.59636	26637.28444	33198.5679	29803.24673	31034.58556	43608.46546	40043.9962	25388.28388	27299.84118
EIF2B1	translation initiation factor eIF-2B subunit alpha [Homo sapiens]	2253620.752	2708691.815	3494240.415	2280347.11	3187392.402	2814756.774	2728781.235	2160620.351	2202082.81
EIF2B2	translation initiation factor eIF-2B subunit beta [Homo sapiens]	464765.8587	1000516.251	1197945.49	1052454.565	1169826.666	2939930.41	993570.5791	702131.5065	1260206.004
EIF2B3	translation initiation factor eIF-2B subunit gamma isoform 1 [Homo sapiens]	617523.8859	615266.2486	711895.5422	600258.7968	668763.5708	614897.5514	535988.3683	436916.243	895689.1827
EIF2B4	translation initiation factor eIF-2B subunit delta isoform 2 [Homo sapiens]	105884.3348	102952.4993	145517.8938	151307.7446	165874.4501	258990.65	227070.3442	185335.5614	144740.0534
EIF2B4	translation initiation factor eIF-2B subunit delta isoform 1 [Homo sapiens]	105884.3348	102952.4993	145517.8938	151307.7446	165874.4501	258990.65	227070.3442	185335.5614	144740.0534
EIF2B5	translation initiation factor eIF-2B subunit epsilon [Homo sapiens]	227123.5308	235936.8037	312196.1392	163577.633	289692.6948	154866.1572	237104.992	237506.8852	249519.3081
EIF2C1	protein argonaute-1 [Homo sapiens]	39316.91696	56281.03591	63473.84461	89253.16713	81287.19613	57176.6029	81047.66713	72454.11948	0
EIF2C2	protein argonaute-2 isoform 1 [Homo sapiens]	107644.819	156252.8036	133382.478	101560.7138	152409.5893	148864.7605	160906.6177	117851.8835	209941.1885
EIF2C3	protein argonaute-3 isoform a [Homo sapiens]	0	0	0	0	0	0	0	0	0
EIF2D	eukaryotic translation initiation factor 2D isoform 1 [Homo sapiens]	927044.5363	469128.9914	618349.8486	428501.1119	615590.6863	525816.7533	447575.1519	391655.7952	495625.7434

EIF2S1	eukaryotic translation initiation factor 2 subunit 1 [Homo sapiens]	7703851.947	6931149.053	9943776.107	7607164.027	10094117.67	7314703.413	7093733.86	6163906.375	7375269.373
EIF2S2	eukaryotic translation initiation factor 2 subunit 2 [Homo sapiens]	3457190.295	4498194.894	3790923.935	4059042.571	4462455.531	3570201.983	3442724.081	3205151.986	4412024.449
EIF2S3	eukaryotic translation initiation factor 2 subunit 3 [Homo sapiens]	8839544.085	10574612.19	11945213.15	9845165.266	11782703.93	10415529.43	9764831.346	7968679.867	10124478.22
EIF3A	eukaryotic translation initiation factor 3 subunit A [Homo sapiens]	904101.8905	850144.8647	1208260.122	1023016.174	1028651.255	1046855.689	995362.0312	842618.0027	913160.0442
EIF3B	eukaryotic translation initiation factor 3 subunit B [Homo sapiens]	3497223.311	4262299.511	4449401.19	3864488.311	4514420.667	4021478.381	3944652.241	2701044.02	4027996.749
EIF3C	eukaryotic translation initiation factor 3 subunit C [Homo sapiens]	1997835.625	2094695.216	2561194.729	2168914.475	1975095.954	2330105.582	2094365.086	1759190.643	1923597.764
EIF3D	eukaryotic translation initiation factor 3 subunit D [Homo sapiens]	2823931.463	2661404.663	3612130.325	3231312.875	3658795.829	3520365.064	3076755.515	2302485.187	2982570.626
EIF3E	eukaryotic translation initiation factor 3 subunit E [Homo sapiens]	3385385.949	3645982.52	4364827.897	3465423.585	4501710.579	3895935.705	3413911.764	2888276.593	3714870.745
EIF3F	eukaryotic translation initiation factor 3 subunit F [Homo sapiens]	9703895.583	12233808.11	13147812.11	12419095.92	13242735.67	12942025.92	13185099.82	10743123.72	12390675.96
EIF3G	eukaryotic translation initiation factor 3 subunit G [Homo sapiens]	4507304.351	4458610.833	5807244.852	3729999.146	6034975.046	4324662.391	3671148.368	3586604.67	4562627.595
EIF3H	eukaryotic translation initiation factor 3 subunit H [Homo sapiens]	2986802.002	3047345.364	3297229.925	3010203.858	4195070.547	3408034.857	2970765.529	2452108.837	3215650.974
EIF3I	eukaryotic translation initiation factor 3 subunit I [Homo sapiens]	6610292.83	7538595.721	8927317.525	7742488.649	8855788.34	7661887.096	7688424.704	5004276.964	8365602.394
EIF3J	eukaryotic translation initiation factor 3 subunit J [Homo sapiens]	6296397.076	6928423.097	8253401.168	6966354.754	9248342.958	7113139.72	6917166.891	5734204.233	7340046.35
EIF3K	eukaryotic translation initiation factor 3 subunit K [Homo sapiens]	8251845.957	9573548.257	11533538.59	9872437.767	11379368.73	10341381.55	10258284	8137320.309	9034928.149
EIF3L	eukaryotic translation initiation factor 3 subunit L isoform 1 [Homo sapien	2595435.18	2825607.06	3331263.67	2989312.202	3322706.195	3080176.588	3029038.045	2417753.125	2884592.657
EIF3M	eukaryotic translation initiation factor 3 subunit M [Homo sapiens]	3544304.863	4592843.847	4211417.92	4956664.936	4647010.255	5246899.567	4853547.476	3692263.915	4536914.884
EIF4A1	eukaryotic initiation factor 4A-I isoform 1 [Homo sapiens]	34117269.45	38790550.24	36815062.2	37223660.19	41810590.11	39592848.18	39573828.29	33456451.91	42183671.19
EIF4A2	eukaryotic initiation factor 4A-II [Homo sapiens]	714450.0171	643995.578	335287.9449	429653.417	484622.5244	445782.0854	321243.9141	235479.7163	765856.7024
EIF4A3	eukaryotic initiation factor 4A-III [Homo sapiens]	9153902.983	10094573.6	12597702.49	10101671.2	12701145.66	9605105.747	10003287.98	7708497	10472858.28
EIF4B	eukaryotic translation initiation factor 4B [Homo sapiens]	1950396.856	1797707.722	2689888.564	2220790.985	2591276.791	2183103.893	1955711.513	1845178.822	2515575.019
EIF4E	eukaryotic translation initiation factor 4E isoform 2 [Homo sapiens]	2909389.091	3251613.128	3365556.551	3344095.323	3390742.932	3220482.433	3458087.116	2796804.414	3376433.702
EIF4E2	eukaryotic translation initiation factor 4E type 2 [Homo sapiens]	350594.1907	359936.2451	650069.1715	736676.2685	323858.4723	576852.4677	653192.8426	0	163703.2694
EIF4EBP1	eukaryotic translation initiation factor 4E-binding protein 1 [Homo sapiens	0	0	0	0	0	0	0	0	0
EIF4EBP2	eukaryotic translation initiation factor 4E-binding protein 2 [Homo sapiens	867787.2121	592045.5152	435692.2273	886842.4697	704596.9242	645271.1555	662112.1136	82856.38448	669203.027
EIF4ENIF1	eukaryotic translation initiation factor 4E transporter isoform a [Homo sap	0	21533.09441	0	19743.51513	27854.45412	16838.14694	0	0	0
EIF4G1	eukaryotic translation initiation factor 4 gamma 1 isoform 6 [Homo sapiens]	633148.1425	0	736166.514	752208.3385	846730.9835	689315.543	0	557984.943	612997.6425
EIF4G1	eukaryotic translation initiation factor 4 gamma 1 isoform 2 [Homo sapiens]	633148.1425	0	736166.514	752208.3385	846730.9835	689315.543	0	557984.943	612997.6425
EIF4G2	eukaryotic translation initiation factor 4 gamma 2 isoform 1 [Homo sapiens]	814907.9306	914940.3644	1078724.158	979417.1385	966638.6631	988041.4982	800168.7987	774965.4683	953728.372
EIF4G3	eukaryotic translation initiation factor 4 gamma 3 isoform 1 [Homo sapiens]	306453.0049	302620.6578	445607.0306	355200.9919	372240.9145	348330.8478	271749.1985	233655.8052	289391.5586
EIF4H	eukaryotic translation initiation factor 4H isoform 1 [Homo sapiens]	7163146.523	8641129.352	9580750.523	7820887.75	9057889.07	7774344.286	5691736.091	4415349.792	8647644.492
EIF5	eukaryotic translation initiation factor 5 [Homo sapiens]	2937436.515	3284143.591	3754259.132	3195316.076	3560056.792	3323662.157	3272066.11	2771932.97	3366692.188
EIF5A	eukaryotic translation initiation factor 5A-1 isoform B [Homo sapiens]	100079742.5	121217891.7	125426545.2	115768787.6	122053812.4	122681446.7	123716534.9	93621000.54	147980624.4
EIF5B	eukaryotic translation initiation factor 5B [Homo sapiens]	897512.608	842232.5013	1154465.473	988440.4521	1188257.495	1001333.819	972640.8377	819727.0384	919618.17
EIF6	eukaryotic translation initiation factor 6 isoform a [Homo sapiens]	14860947.69	18484439.45	18505175.71	16427936.59	18342018.41	16875333.57	15987411.99	14608941.7	19086833.91
ELAC2	zinc phosphodiesterase ELAC protein 2 isoform 1 [Homo sapiens]	757146.3132	957735.3448	1142866.826	956502.5048	1128083.828	1008320.553	910130.3632	695072.2081	879941.5487
ELAVL1	ELAV-like protein 1 [Homo sapiens]	5005828.614	4080248.629	7088902.302	6000803.819	6966207.171	4860807.303	5938203.865	4298322.369	3941291.99
ELL	RNA polymerase II elongation factor ELL [Homo sapiens]	33286.55657	38271.1819	0	26775.30361	0	31254.28604	38797.52876	0	3466.503992
ELMO1	engulfment and cell motility protein 1 isoform 1 [Homo sapiens]	100181.2332	16605.36801	23771.54567	113324.2037	121631.4773	66655.27098	113286.1613	145878.1486	113293.5389
ELMO2	engulfment and cell motility protein 2 [Homo sapiens]	0	16043.66955	34393.16727	26753.06	0	27030.12818	0	0	0
ELOF1	transcription elongation factor 1 homolog [Homo sapiens]	8144413.381	9755604.333	3151102.381	2235755.655	0	0	2208821.024	1551854.47	9498449.095
ELOVL1	elongation of very long chain fatty acids protein 1 isoform 1 [Homo sapiens	4890328.05	1659497.119	5290738.614	3805470.743	4928364.107	2692644.243	184238.9286	1961730.856	1495244.25
ELOVL5	elongation of very long chain fatty acids protein 5 isoform 2 [Homo sapiens	4192990.472	1578760.896	8576939.921	3880594.718	9143561.851	4106235.688	4190358.128	3238900.216	4035125.875
ELP2	elongator complex protein 2 isoform 1 [Homo sapiens]	193476.5812	271414.9411	249477.9447	216268.6268	444921.388	260108.4389	222487.413	176590.6104	228706.7808
ELP3	elongator complex protein 3 [Homo sapiens]	348198.0397	339995.4777	261935.129	343907.781	390776.9244	433163.664	342655.2068	281515.4344	320757.6642
ELP4	elongator complex protein 4 [Homo sapiens]	170442.6367	233315.1845	252112.3499	225786.3821	251022.2213	187164.6067	142693.6698	104629.7853	120642.6748
ELP5	elongator complex protein 5 isoform 1 [Homo sapiens]	250205.321	702414.2131	336338.6313	459284.7585	0	369831.6658	408188.267	114469.8825	0
ELP6	elongator complex protein 6 [Homo sapiens]	222221.7245	110169.9733	336360.1314	250387.2594	384374.9431	213475.243	377185.2347	110356.3061	236912.1684
EMC1	ER membrane protein complex subunit 1 isoform 1 precursor [Homo sapiens]	354379.8105	425989.9884	568024.334	541571.8663	475041.5642	527668.4442	499021.9381	411248.7131	496143.4355
EMC10	ER membrane protein complex subunit 10 isoform 2 precursor [Homo sapiens]	960375.5921	2249000.434	1366922.842	1480413.333	1389106.901	1284507.556	0	1736396.849	2416134.267
EMC2	ER membrane protein complex subunit 2 [Homo sapiens]	860069.287	968748.7789	1090953.245	994016.8911	1073669.671	1157016.443	1205836.587	618036.0722	1050573.073
EMC3	ER membrane protein complex subunit 3 [Homo sapiens]	938731.9958	1105113.929	886445.4925	922536.5728	890921.264	1179352.113	1209083.502	918642.8708	1161733.899
EMC4	ER membrane protein complex subunit 4 [Homo sapiens]	1454459.593	1246320.387	2222875.657	2005818.755	1458718.897	2797724.207	1441726.617	1675330.138	0
EMC7	chromosome 15 open reading frame 24 precursor [Homo sapiens]	944143.5208	2025464.777	1646136.832	1330606.078	1110864.611	1387985.187	1030464.174	953415.0833	2475161.908
EMC8	ER membrane protein complex subunit 8 isoform 1 [Homo sapiens]	945383.6094	1086721.714	1328297.434	1189884.116	235491.0846	597558.0524	815893.2868	887888.9771	1299635.306
EMC9	ER membrane protein complex subunit 9 [Homo sapiens]	0	0	0	0	0	0	590659.2182	171938.8011	0
EMD	emerin [Homo sapiens]	4306370.528	4555188.084	4478845.294	4885511.722	4656344.317	4851631.063	4092592.4	3446087.075	4332918.975
EMG1	ribosomal RNA small subunit methyltransferase NEP1 [Homo sapiens]	2573834.754	2365195.618	3458143.295	2977256.102	3498701.022	3175889.614	2647442.102	2413342.805	3309501.584
EML2	echinoderm microtubule-associated protein-like 2 isoform 1 [Homo sapiens]	185007.4966	174968.921	262044.2609	243513.1732	320510.2648	135951.2503	216040.4111	150264.1975	224296.1105
EML3	echinoderm microtubule-associated protein-like 3 [Homo sapiens]	168000.87	62410.87576	223781.534	155999.972	251637.7512	160038.1576	162878.9252	155553.5975	94312.40281
EML4	echinoderm microtubule-associated protein-like 4 isoform a [Homo sapiens]	537297.0459	176555.0116	766164.4854	551278.0818	253934.5565	596053.6451	156938.0818	473593.3123	495091.4812
EMP3	epithelial membrane protein 3 [Homo sapiens]	2171652.987	3774634.368	0	1214209.033	4774135.421	946423.2368	2562757.822	1616678.888	2815350.429
EMR2	EGF-like module-containing mucin-like hormone receptor-like 2 isoform a pre	37130.06461	0	153878.3539	54106.07188	0	115360.6208	0	0	0
ENAH	protein enabled homolog isoform a [Homo sapiens]	446597.79	468950.1909	531170.5316	611904.1905	627067.7524	454951.8048	478912.4238	419414.6138	529828.8095
ENG	endoglin isoform 1 precursor [Homo sapiens]	209084.7132	378919.0997	294006.4172	311729.7546	277801.459	370332.6871	334481.2945	296670.566	126365.6411
ENO1	alpha-enolase isoform 1 [Homo sapiens]	138719015.1	138259534.6	147359132.7	146465156.1	145686641.6	156378985.3	160898331.5	133919245.9	164237006.6
ENO2	gamma-enolase [Homo sapiens]	753680.102	755507.2151	998451.4868	868620.1289	1020396.831	838857.3664	1022712.541	734253.6303	793896.7645
ENO3	beta-enolase isoform 1 [Homo sapiens]	45540394.08	50519245.28	50014567.86	47267779.72	49497899.17	50604150.12	54533503.73	38514837.23	58929290.93
ENOPH1	enolase-phosphatase E1 [Homo sapiens]	1986659.523	2157381.308	2689811.959	1960776.963	2795546.654	2291511.878	2251832.389	2043931.733	2408406.742
ENOSF1	mitochondrial enolase superfamily member 1 isoform rTSbeta [Homo sapiens]	0	0	0	0	0	0	184174.5859	152102.8553	113622.4795
ENOSF1	mitochondrial enolase superfamily member 1 isoform rTSgamma [Homo sapiens]	0	0	0	0	0	0	184174.5859	152102.8553	113622.4795
ENSA	alpha-endosulfine isoform 1 [Homo sapiens]	3909414.251	5562235.037	3311423.381	9778086.912	5552411.572	5005204.532	4883174.984	7561830.713	10309658.05
ENTHD2	AP-4 complex accessory subunit tepsin [Homo sapiens]	131832.9521	135870.2809	82932.30698	74348.75314	168892.7642	143033.8143	0	0	0
ENY2	enhancer of yellow 2 transcription factor homolog isoform 1 [Homo sapiens]	2883942.918	2961376.942	1972358.848	3057982.51	1524148	3094769.567	2624985.572	2437914.55	3271218.038
EP400	E1A-binding protein p400 [Homo sapiens]	10505.17814	9011.08008	9213.985229	14725.23197	23755.65684	12534.40578	8560.715261	14601.58177	9271.99798
EPB41	protein 4.1 isoform 1 [Homo sapiens]	154544.0208	170895.8954	135682.4911	104714.5033	190811.0818	141796.9348	94293.27383	85809.85473	126366.4679
EPB41L1	band 4.1-like protein 1 isoform a [Homo sapiens]	3431.276787	0	6699.751257	5117.806657	8557.523377	5372.740706	0	0	0
EPB41L2	band 4.1-like protein 2 isoform a [Homo sapiens]	157762.947	210082.8424	213604.7026	177702.6159	195621.0656	190642.4402	196028.9679	166925.9921	213309.2655
EPCAM	epithelial cell adhesion molecule precursor [Homo sapiens]	28327.59734	85683.9068	56315.83402	98797.76254	54409.96926	70706.02766	75821.59606	57888.96516	75114.67418
EPDR1	mammalian ependymin-related protein 1 isoform 1 precursor [Homo sapiens]	604653.9408	1159878.086	644070.4474	1193437.492	931638.3191	1219742.683	1217155.23	977896.0909	1067546.326
EPHA2	ephrin type-A receptor 2 precursor [Homo sapiens]	99204.04932	122048.1995	163786.7204	122119.025	156369.4906	145183.1016	217054.5563	172849.016	220518.4098
EPHX1	epoxide hydrolase 1 precursor [Homo sapiens]	1083639.27	1750557.002	1997122.184	1498939.593	2044930.527	1647555.356	1735401.698	1461614.099	1704089.781
EPHX4	epoxide hydrolase 4 [Homo sapiens]	0	0	0	0	0	0	82165.28977	72014.32955	83598.25189
EPM2AIP1	EPM2A-interacting protein 1 [Homo sapiens]	57667.49205	383935.625	0	0	53263.08607	44934.46721	0	44824.81275	81517.0041
EPN1	epsin-1 isoform a [Homo sapiens]	251761.9344	245787.1192	0	263889.9316	279391.0392	277426.8151	270907.9865	156660.8609	255841.6105
EPN2	epsin-2 isoform b [Homo sapiens]	158469.798	82925.5849	121723.6132	122489.811	155588.7321	101629.8119	78960.84915	71883.26115	109984.7963
EPPK1	epiplakin [Homo sapiens]	1552127.531	893401.5693	1120288.778	968039.2912	1186388.975	1099816.119	1047855.58	849455.7085	957327.9168
EPRS	bifunctional glutamate/proline--tRNA ligase [Homo sapiens]	1324247.611	1456925.736	1566947.1	1524573.156	1899094.556	1602472.963	1264379.466	1294049.29	1464255.696
EPS15	epidermal growth factor receptor substrate 15 isoform A [Homo sapiens]	119194.8431	107460.3803	193222.995	140595.0755	203133.1995	125906.0646	133634.4895	125880.4524	182921.9437
EPS15L1	epidermal growth factor receptor substrate 15-like 1 isoform 1 [Homo sapien	168458.6087	176483.4681	229745.8527	155005.9339	200575.4156	182825.5698	168068.7767	136085.1973	200695.6807
EPS8L2	epidermal growth factor receptor kinase substrate 8-like protein 2 [Homo sa	372856.1039	328364.576	469436.8519	411833.2891	469661.0291	324742.6721	407939.3025	338734.6476	418045.0067
EPT1	ethanolaminephosphotransferase 1 [Homo sapiens]	2493147.596	2641636.8	3044790.679	2676882.158	3268285.734	2994669.675	1967419.189	2023899.166	2964066.232
ERAL1	GTPase Era, mitochondrial [Homo sapiens]	432556.5745	186180.3438	451780.6268	180361.9525	302084.6179	125552.1136	205814.3301	352766.5069	418072.8813
ERAP1	endoplasmic reticulum aminopeptidase 1 isoform a precursor [Homo sapiens]	125160.5331	54793.57445	148954.5545	76420.04246	158312.3956	32443.62773	62925.69913	29534.57399	71502.61735
ERBB2IP	protein LAP2 isoform 8 [Homo sapiens]	585569.8045	86510.31428	87976.38693	99670.31364	95459.04134	105983.7176	105777.1117	86202.78673	222255.4099
ERBB4	receptor tyrosine-protein kinase erbB-4 isoform JM-a/CVT-1 precursor [Homo	0	0	0	0	0	0	0	0	0
ERC1	ELKS/Rab6-interacting/CAST family member 1 isoform epsilon [Homo sapiens]	100319.6554	103297.8547	170804.4306	138941.0888	150972.9386	85427.11614	118767.1735	60010.711	145634.7399
ERCC2	TFIIH basal transcription factor complex helicase XPD subunit isoform 1 [Ho	75031.50926	67104.01475	170212.3687	103567.7225	69283.50505	60010.35438	37539.59148	78140.83704	137953.299
ERCC3	TFIIH basal transcription factor complex helicase XPB subunit [Homo sapiens	96557.44954	0	121221.7753	66060.03846	92230.60932	50724.82437	94957.50526	55898.17486	100113.8512
ERCC4	DNA repair endonuclease XPF [Homo sapiens]	0	0	0	0	0	0	22784.379	18191.07001	0
ERCC6	DNA excision repair protein ERCC-6 [Homo sapiens]	6027.229402	43011.56086	51430.32139	0	40128.16609	39695.82553	24555.9996	20905.99556	49277.21095
ERCC6L	DNA excision repair protein ERCC-6-like [Homo sapiens]	74465.96753	150488.6858	70691.96429	69014.17276	55447.54558	64518.89264	72974.6982	59469.42307	189664.1485
ERF	ETS domain-containing transcription factor ERF [Homo sapiens]	0	16309.93941	84491.48803	28661.34441	105705.234	29103.1752	18916.33045	0	47568.29821
ERGIC1	endoplasmic reticulum-Golgi intermediate compartment protein 1 [Homo sapien	1963033.593	2205094.091	2484958.073	2187191.573	1572186.378	2085660.273	2181003.959	2017674.2	2144286.777
ERGIC2	endoplasmic reticulum-Golgi intermediate compartment protein 2 [Homo sapien	423842.7422	233360.7101	374956.8259	448729.3097	381639.9404	292169.8735	356451.4219	281334.4721	423865.269
ERGIC3	endoplasmic reticulum-Golgi intermediate compartment protein 3 isoform a [H	286831.9703	238077.6809	496403.9285	428025.1098	376047.4282	474808.5522	436082.2068	149659.3941	494936.101
ERH	enhancer of rudimentary homolog [Homo sapiens]	47092860.34	58772140.85	52164332.27	56241576.93	58345717.59	54049075.78	55702966.29	46929569.98	59937801.63
ERI1	3'-5' exoribonuclease 1 [Homo sapiens]	197173.5099	401623.4888	214140.6989	336951.3225	274815.934	526833.0927	376151.9156	0	423379.3891
ERI3	ERI1 exoribonuclease 3 [Homo sapiens]	414265.6695	0	496070.762	0	329465.4375	472704.8955	648513.0878	337754.8798	492474.7596
ERLEC1	endoplasmic reticulum lectin 1 isoform 1 precursor [Homo sapiens]	0	102233.0628	110459.5321	144075.6571	173774.6898	124720.3035	100310.0979	192429.3403	170058.9826
ERLIN1	erlin-1 [Homo sapiens]	436229.1511	254244.3297	824108.5687	458512.4814	830845.9633	514460.7889	464185.6953	359662.8355	552894.5361
ERLIN2	erlin-2 isoform 1 [Homo sapiens]	528284.451	581760.0682	709728.0741	574293.1111	822138.44	551471.3954	639990.458	375778.3577	595777.596
ERMP1	endoplasmic reticulum metallopeptidase 1 [Homo sapiens]	84521.69715	0	142719.398	0	89409.52964	138331.9487	99850.88241	104828.3475	0
ERO1L	ERO1-like protein alpha precursor [Homo sapiens]	1776601.204	1891966.266	2533748.906	1853434.143	2104578.42	2034829.198	2123030.497	1627491.923	2122677.743
ERP29	endoplasmic reticulum resident protein 29 isoform 1 precursor [Homo sapiens	9081141.625	8412877.479	9747030.241	9355431.196	9721601.75	9529650.464	9959374.741	8276916.263	9877360.098
ERP44	endoplasmic reticulum resident protein 44 precursor [Homo sapiens]	1242439.327	1309798.371	1594778.801	1483936.836	1931772.218	1473642.612	1587925.37	1387615.934	1712396.803
ESD	S-formylglutathione hydrolase [Homo sapiens]	6338790.745	6439311.828	8126798.872	6892777.751	8129029.083	7362634.926	5624074.915	6002177.513	7036050.909
ESF1	ESF1 homolog [Homo sapiens]	60543.75946	69522.03828	70915.51597	78083.03983	77068.53371	86287.68499	79966.7801	51763.2029	71574.28521
ESPL1	separin [Homo sapiens]	18733.49162	18097.45275	18065.96733	0	168394.8894	26422.2361	0	0	0
ESRP2	epithelial splicing regulatory protein 2 [Homo sapiens]	977738.9642	28069.90393	1086147.497	1010938.264	1199636.716	132528.9486	1066256.379	93191.46057	0
ESRRA	steroid hormone receptor ERR1 [Homo sapiens]	93583.98344	89350.3204	93640.66848	83177.88411	101152.2757	119996.7697	91524.62954	78900.06788	31805.9582

ESYT1	extended synaptotagmin-1 isoform 1 [Homo sapiens]	1072860.095	755022.3051	1127056.683	857403.2848	1177918.968	883269.6053	893642.7467	632272.6475	760451.815
ESYT2	extended synaptotagmin-2 [Homo sapiens]	268178.2195	277065.9931	334986.743	293673.1752	374429.0869	250517.231	240145.0577	213377.0974	287666.633
ETF1	eukaryotic peptide chain release factor subunit 1 [Homo sapiens]	3569953.372	4410713.76	4568710.174	4065135.916	4999776.777	4294578.808	3931476.078	3003117.316	4399867.19
ETFA	electron transfer flavoprotein subunit alpha, mitochondrial isoform a [Homo	5235611.813	5293829.186	6320371.511	6088163.023	6804483.938	6037636.098	6875495.503	5309113.692	6378824.95
ETFB	electron transfer flavoprotein subunit beta isoform 2 [Homo sapiens]	3160973.067	3439045.887	3834002.11	3685752.488	4028032.921	3744984.656	3560642.597	2950281.91	3759548.968
ETFDH	electron transfer flavoprotein-ubiquinone oxidoreductase, mitochondrial pre	176606.6217	72228.04646	119932.3143	242594.3035	202611.3731	171677.1858	118047.4019	79770.36699	104117.5066
ETHE1	protein ETHE1, mitochondrial [Homo sapiens]	485990.0901	88847.59012	1129740.379	1148901.953	754178	1022959.155	1371884.11	483734.3242	554748.1149
ETNK1	ethanolamine kinase 1 isoform A [Homo sapiens]	204355.6421	45779.49542	305460.5667	213203.1117	553729.3	97290.7504	161354.7468	163115.2867	231138.2689
EVPL	envoplakin [Homo sapiens]	17941.79413	14335.15366	19605.4781	17998.45277	20928.40222	22365.40687	10616.17743	10007.65421	11184.34264
EWSR1	RNA-binding protein EWS isoform 1 [Homo sapiens]	3523469.168	4087974.388	4563361.592	4412460.689	4821602.308	4410855.264	4263061.294	3656080.449	4301444.528
EXD2	exonuclease 3'-5' domain-containing protein 2 isoform 1 [Homo sapiens]	78001.51307	44005.27322	96382.10663	46977.5841	94421.19609	97940.51989	74839.03904	71757.77218	42875.4636
EXOC1	exocyst complex component 1 isoform 1 [Homo sapiens]	96659.13562	40813.05411	58727.34762	38751.84932	67317.97934	0	22361.5089	35790.95641	77990.55447
EXOC2	exocyst complex component 2 [Homo sapiens]	108046.489	95492.28803	133338.8087	102408.6359	92847.62536	87061.38503	92280.59052	0	79991.24454
EXOC3	exocyst complex component 3 [Homo sapiens]	159202.1471	67096.90333	210004.4517	155783.1319	203292.0908	66032.73673	65580.5597	81580.40127	63517.44037
EXOC4	exocyst complex component 4 isoform a [Homo sapiens]	84389.60724	56961.26849	111665.3756	90903.37957	108243.6746	98062.44622	93530.97597	47329.29436	97818.71684
EXOC5	exocyst complex component 5 [Homo sapiens]	112460.5126	157842.0879	149525.3258	106382.1035	188900.548	162824.7267	112516.8984	133756.7432	126921.3328
EXOC6	exocyst complex component 6 isoform a [Homo sapiens]	53700.26266	43789.7937	59896.32873	55498.80104	131506.8671	62460.5443	58644.91851	15823.59177	56024.01335
EXOC6B	exocyst complex component 6B [Homo sapiens]	29958.75947	22690.31605	38906.54848	31972.86827	40066.84015	37157.44488	24826.33958	0	27225.02912
EXOC7	exocyst complex component 7 isoform 4 [Homo sapiens]	103129.6113	125232.7452	123899.5025	130511.755	143119.0895	174771.2606	165235.2075	129419.9894	220635.7872
EXOC8	exocyst complex component 8 [Homo sapiens]	105633.8925	92004.86037	178427.8998	91751.86966	181446.0864	89294.94088	71065.95608	84323.45264	78704.28041
EXOG	nuclease EXOG, mitochondrial isoform 1 precursor [Homo sapiens]	0	0	0	0	0	0	18969.72646	0	127740.3784
EXOSC1	exosome complex component CSL4 [Homo sapiens]	856968.3724	983911.1909	1656237.447	1117969.811	1574813.718	1122873.952	1141950.045	925427.522	1682288.318
EXOSC10	exosome component 10 isoform 1 [Homo sapiens]	339558.6418	408347.1062	399093.1077	361927.8466	464827.192	379181.3299	334290.1157	361746.0572	403244.9887
EXOSC2	exosome complex component RRP4 [Homo sapiens]	1320225.913	1345041.171	1938611.929	1236330.596	1821976.07	1357060.282	1233545.88	1253349.592	1565854.548
EXOSC3	exosome complex component RRP40 isoform 1 [Homo sapiens]	1277443.235	1341450.021	1676646.706	1452826.06	1578976.291	1649214.75	1442559.497	1034349.926	1927162.321
EXOSC4	exosome complex component RRP41 [Homo sapiens]	1564466.805	2081594.149	2128059	2233005.885	1409688.214	2045118.828	2158661.544	1683283.459	2213025.531
EXOSC5	exosome complex component RRP46 [Homo sapiens]	882360.9185	728640	746814.0876	1057348.018	702013.5994	1145882.026	1094938.826	654991.9738	716301.4943
EXOSC6	exosome complex component MTR3 [Homo sapiens]	2103615.125	2080463.28	2922206.649	2273631.435	2616852.521	2300078.788	2208953.943	1818355.672	2230882.558
EXOSC7	exosome complex component RRP42 [Homo sapiens]	800940.0091	824532.9529	1314641.354	972336.6123	1506664.775	1385025.168	1322399.901	1117277.367	1109206.428
EXOSC8	exosome complex component RRP43 [Homo sapiens]	2104611.01	2544314.132	2818557.565	2356253.457	2654879.323	2421864.218	2391971.647	2165466.14	2655905.271
EXOSC9	exosome complex component RRP45 isoform 1 [Homo sapiens]	812643.0509	1021942.092	1164649.842	993332.2583	887059.2503	1080615.23	1029220.672	861022.6564	1204391.437
EYA3	eyes absent homolog 3 [Homo sapiens]	163227.2511	284526.5158	240786.9798	246110.9423	275394.9313	317312.5779	294140.8314	166743.8266	145931.0524
EZH2	histone-lysine N-methyltransferase EZH2 isoform a [Homo sapiens]	107184.0545	107859.272	166103.7073	130779.3202	146210.8981	90756.38676	128335.8805	119521.0165	114119.2707
EZR	ezrin [Homo sapiens]	4097100.039	4402734.683	5048386.126	4430955.091	5050863.097	4936471.049	4585473.337	3920674.9	4770131.845
F11R	junctional adhesion molecule A precursor [Homo sapiens]	68216.99272	0	117548.8665	154723.9515	150547.8131	0	0	0	0
F2	prothrombin preproprotein [Homo sapiens]	88532.08143	92034.21429	103993.0429	118495.7532	79432.33193	85751.55277	194041.5959	83870.46849	207301.8816
F3	tissue factor isoform 1 precursor [Homo sapiens]	0	0	0	0	0	0	0	0	0
F5	coagulation factor V precursor [Homo sapiens]	0	0	0	0	0	0	240071.2723	135753.2071	272599.4139
F8A1	factor VIII intron 22 protein [Homo sapiens]	162106.9119	572209.0515	235968.4738	101710.228	293224.4835	137952.3354	108025.5343	126886.0786	316704.1851
FABP5	fatty acid-binding protein, epidermal [Homo sapiens]	27067263.48	38599057.6	36032889.38	32852236.32	32779886.55	35520191.22	36418115.77	33439308.12	38047046.58
FADD	protein FADD [Homo sapiens]	902226.0462	916778.9104	1132900.164	566031.7806	528531.981	1075635.788	592824.1353	550627.971	1064894.166
FADS1	fatty acid desaturase 1 [Homo sapiens]	973780.6942	1094744.469	328658.7082	412878.1597	256483.8837	483569.5574	480829.1243	294762.5477	1019397.84
FADS3	fatty acid desaturase 3 [Homo sapiens]	197712.1539	308796.4972	406626.6114	263665.6437	296308.8802	206152.8254	188077.3533	0	156378.9773
FAF1	FAS-associated factor 1 [Homo sapiens]	733544.6634	829154.4504	582002.992	830355.4795	658412.6348	920765.4826	758452.3786	510272.7978	864565.9866
FAF2	FAS-associated factor 2 [Homo sapiens]	917039.6313	962706.3994	858193.3563	994335.8614	1090186.311	1020216.185	1031098.729	734468.2142	988462.0847
FAH	fumarylacetoacetase [Homo sapiens]	402758.7289	415520.944	559027.0125	466317.1312	504717.9422	636275.3104	581013.6629	363769.954	624219.7612
FAHD1	acylpyruvase FAHD1, mitochondrial isoform 2 [Homo sapiens]	441288.6199	0	192730.8685	580670.594	589228.868	925750.276	0	324054.0571	716034.8945
FAHD1	acylpyruvase FAHD1, mitochondrial isoform 1 [Homo sapiens]	441288.6199	0	192730.8685	580670.594	589228.868	925750.276	0	324054.0571	716034.8945
FAHD2A	fumarylacetoacetate hydrolase domain-containing protein 2A [Homo sapiens]	476374.5647	803491.1516	312365.1339	819253.4978	413205.926	994940.5866	883049.4498	754675.7673	687499.1429
FAIM	fas apoptotic inhibitory molecule 1 isoform c [Homo sapiens]	1521887.086	1540375.268	1649240.974	1681964.477	3536754.468	1889395.264	2378436.79	1368749.191	1579030.401
FAM103A1	RNMT-activating mini protein [Homo sapiens]	306842.1316	557438.7774	790944.6919	725950.5198	178963.2982	589417.2807	594397.0321	920913.4912	1062853.643
FAM105A	protein FAM105A [Homo sapiens]	83116.06761	92769.29403	56792.6022	174542.3208	94870.53145	197753.5975	144471.7044	116873.8805	126484.6226
FAM105B	protein FAM105B [Homo sapiens]	837815.9628	769116.9295	1127657.544	614934.3304	979723.7724	854280.7949	889340.9468	536700.9426	471669.5315
FAM107B	protein FAM107B [Homo sapiens]	604497.775	0	715911.93	537529.1867	844023.0233	607719.8442	536883.685	512918.2759	877871.722
FAM109B	sesquipedalian-2 [Homo sapiens]	0	0	0	0	0	0	161897.2287	0	76328.82846
FAM111A	protein FAM111A [Homo sapiens]	34312.31789	30621.04135	46957.1585	24213.23102	34830.89327	47938.33269	18658.2506	33269.10751	3998.193512
FAM111B	protein FAM111B isoform a [Homo sapiens]	0	25674.5856	65916.52134	86756.50954	0	85115.62195	0	0	0
FAM114A1	protein NOXP20 [Homo sapiens]	326772.8022	390442.683	545965.5178	354261.8398	463876.2268	451685.4216	299964.7073	376943.7051	418994.5088
FAM114A2	protein FAM114A2 [Homo sapiens]	167633.7021	74196.09441	165153.8391	108021.4132	143040.6822	143605.8644	0	94077.30319	64346.48473
FAM115A	protein FAM115A isoform 1 [Homo sapiens]	173943.7991	75540.32885	160383.4644	56732.88226	177717.5362	102742.9652	91500.09843	62945.79355	68427.53679
FAM118B	protein FAM118B [Homo sapiens]	255487.5393	287896.2071	337858.7375	300806.4786	367519.5	431497.8732	0	0	0
FAM120A	constitutive coactivator of PPAR-gamma-like protein 1 [Homo sapiens]	471123.0057	434210.8618	456972.7813	430910.7534	604998.2576	417847.8833	404874.4309	393875.7564	441157.5935
FAM120C	constitutive coactivator of PPAR-gamma-like protein 2 isoform 1 [Homo sapie	18154.61815	13798.58274	0	0	38292.453	30017.11914	11129.37533	0	40962.96313
FAM127B	protein FAM127B isoform 1 [Homo sapiens]	486181.9271	0	548914.7292	628455.2604	452551.5846	546024.0417	446135.5625	742020.1342	335366.0156
FAM129A	protein Niban [Homo sapiens]	148144.801	151104.1405	172668.6103	126795.3778	169004.0806	147738.6842	148873.4296	128529.488	210007.2537
FAM129B	niban-like protein 1 isoform 1 [Homo sapiens]	1956837.497	1725834.799	2376760.314	1892352.766	2502485.663	2165619.863	1932489.316	1584662.332	2074650.203
FAM134C	protein FAM134C [Homo sapiens]	611291.1842	187100.8492	514076.1189	286441.3214	537354.345	196708.7723	146624.0987	546297.0883	201522.0292
FAM136A	protein FAM136A [Homo sapiens]	2007179.34	2022255.899	3274253.791	2018878.243	2744167.642	2230411.491	2154608.352	1745830.31	1960579.318
FAM162A	protein FAM162A [Homo sapiens]	3104001.794	6344599.507	5423055.028	5508251.31	5978245.021	5249789.6	3342307.184	4738898.39	2929775.147
FAM168B	myelin-associated neurite-outgrowth inhibitor [Homo sapiens]	0	0	0	0	0	0	0	0	0
FAM175B	BRISC complex subunit Abro1 [Homo sapiens]	169056.2798	99511.65136	249734.6246	245454.0136	275168.0255	105063.4788	0	0	0
FAM177A1	protein FAM177A1 isoform 1 precursor [Homo sapiens]	486437.7919	933973.587	891574.9328	778018.9142	531850.4179	678711.0199	559802.4104	417510.2803	727186.3022
FAM192A	protein FAM192A [Homo sapiens]	357173.0477	264773.4237	517043.5324	210019.598	693764.7443	367978.313	387710.6851	252479.8282	433193.6102
FAM195A	protein FAM195A [Homo sapiens]	186595.8104	59741.77679	256545.2813	0	286114.6809	0	0	0	0
FAM203B	PREDICTED: protein FAM203B [Homo sapiens]	751992.9588	799865.2756	975569.4252	855850.4283	995505.2504	920371.8717	643610.4994	742568.1235	867335.6228
FAM207A	protein FAM207A [Homo sapiens]	355847.1203	685351.07	564871.4701	444427.0007	485350.4815	548510.9028	558650.4555	608705.5518	600054.99
FAM208A	protein FAM208A isoform a [Homo sapiens]	49720.21877	56831.7291	52875.07559	139855.1633	66531.01171	62359.43347	43657.40837	55573.96335	50952.20481
FAM20B	glycosaminoglycan xylosylkinase [Homo sapiens]	36786.06126	39480.17881	51990.35927	29423.16225	0	36662.19785	25411.4495	0	49540.88576
FAM210A	protein FAM210A [Homo sapiens]	0	421097.48	602244.26	488164.708	0	472246.168	566175.572	0	491406.66
FAM210B	protein FAM210B [Homo sapiens]	48623.00131	73378.62847	30925.46007	23787.08464	0	56448.66521	87400.92122	62838.65332	0
FAM213A	redox-regulatory protein FAM213A isoform 1 precursor [Homo sapiens]	2413660.234	2796251.338	2046096.775	1444370.512	2077495.662	3070379.014	3140442.544	1210543.959	2523274.493
FAM21A	WASH complex subunit FAM21A [Homo sapiens]	98224.81293	84754.57798	117197.1999	104213.4893	108171.33	98218.57349	84323.7573	75196.2144	31653.14263
FAM3A	protein FAM3A isoform 1 precursor [Homo sapiens]	0	0	0	0	0	0	305615.6739	0	244894.4511
FAM3C	protein FAM3C precursor [Homo sapiens]	918743.7793	978413.1374	1251792.038	813535.7226	1254768.094	1305428.255	1250529.484	946118.2359	1220497.096
FAM45A	protein FAM45A [Homo sapiens]	130840.5241	272812.4667	162004.3704	0	0	145194.1222	0	79939.98333	223569.5676
FAM49B	protein FAM49B [Homo sapiens]	2456648.934	2629432.394	3263646.683	2927844.98	3386549.517	2877076.909	2881439.946	2274250.254	2935018.761
FAM50A	protein FAM50A [Homo sapiens]	829005.887	1043951.044	1159190.196	500466.0565	1141564.211	843119.5147	788031.1593	789930.6492	1089285.494
FAM65A	protein FAM65A isoform 3 [Homo sapiens]	59457.63664	59681.31041	52384.10168	68784.77729	82523.63868	37288.94519	32250.76641	33589.4191	34080.44284
FAM73A	protein FAM73A isoform 2 [Homo sapiens]	17006.75614	0	42960.2995	9892.638874	20844.94801	16075.01782	0	0	0
FAM83D	protein FAM83D [Homo sapiens]	30978.70027	0	45880.88496	50751.25137	83643.53265	66991.7323	0	0	0
FAM83H	protein FAM83H [Homo sapiens]	33534.43072	70552.61199	477310.9419	128782.7643	35530.50177	42566.66266	0	0	0
FAM91A1	protein FAM91A1 [Homo sapiens]	74226.93563	130919.0521	234753.3116	114014.3834	0	134369.5341	24082.18843	76947.01041	92934.83892
FAM96A	MIP18 family protein FAM96A isoform a precursor [Homo sapiens]	808881.8689	945527.377	776926.1374	924707.9344	767116.0718	977034.9016	854543.2459	0	932611.8287
FAM96B	mitotic spindle-associated MMXD complex subunit MIP18 [Homo sapiens]	2472586.429	1852849.647	2533313.498	2322599.422	2961424.594	2021210.793	2245948.884	1608053.592	1817928.152
FAM98A	protein FAM98A [Homo sapiens]	701692.3665	956178.3569	818822.4919	971643.4939	947821.4269	992991.6919	995931.2503	816157.5396	944913.935
FAM98B	protein FAM98B isoform 1 [Homo sapiens]	1028859.332	972890.9511	1277599.377	1359140.288	1586678.49	1309953.369	1267456.352	1061131.572	1125597.845
FANCD2	Fanconi anemia group D2 protein isoform a [Homo sapiens]	57788.58325	50391.5846	97238.82258	92173.81704	131268.4393	1286750.636	69020.35837	82264.45494	77074.09161
FANCI	Fanconi anemia group I protein isoform 1 [Homo sapiens]	76016.10064	118714.1467	115787.9338	88494.50042	102563.5	101580.4058	88407.88517	81752.06234	110196.6113
FAR1	fatty acyl-CoA reductase 1 [Homo sapiens]	247824.7033	409940.8968	527063.5368	359975.8806	428627.1564	497154.9397	325948.4117	317996.2784	513413.0388
FARP1	FERM, RhoGEF and pleckstrin domain-containing protein 1 isoform 1 [Homo sap	41026.7127	33273.93998	59894.25466	22638.96941	40867.59091	36145.5035	0	0	0
FARS2	phenylalanine--tRNA ligase, mitochondrial precursor [Homo sapiens]	180174.0243	113680.4993	344310.33	137836.3113	289623.3813	109523.7381	179304.68	92417.26394	141309.1779
FARSA	phenylalanine--tRNA ligase alpha subunit [Homo sapiens]	1636770.242	1620849.468	1891059.642	1833679.479	2341875.545	1774551.564	1452308.159	1349366.278	1788826.565
FARSB	phenylalanine--tRNA ligase beta subunit [Homo sapiens]	1064694.73	1292098.64	1525486.049	1411635.584	1635384.778	1618874.276	1367991.279	1130253.328	1478142.39
FAS	tumor necrosis factor receptor superfamily member 6 isoform 1 precursor [Ho	93667.59691	138438.6389	196870.1191	89283.06174	92962.86134	107527.7805	123068.9545	125103.5948	110009.0615
FASN	fatty acid synthase [Homo sapiens]	4445260.461	4343882.964	6428119.458	4593374.978	6394655.91	4961756.37	4381556.997	3778157.637	4843969.499
FASTKD2	FAST kinase domain-containing protein 2 [Homo sapiens]	235815.1578	202543.5113	282104.1405	240033.6505	336623.2298	207094.3569	219797.3533	206489.851	212099.5798
FASTKD3	FAST kinase domain-containing protein 3 [Homo sapiens]	95550.28719	0	116463.0659	85118.7905	101931.2976	94679.47358	89469.49231	87470.70288	89992.24704
FASTKD5	FAST kinase domain-containing protein 5 [Homo sapiens]	52235.36281	90872.19526	97358.66628	85344.58259	75629.17931	105681.2026	82764.83814	60712.37745	87725.83624
FAU	ubiquitin-like protein fubi and ribosomal protein S30 precursor [Homo sapie	30272950.84	18795549.95	61197410.58	36130483.48	59520424.24	52968672.58	24479722.64	40418175.84	40558802.78
FBL	rRNA 2'-O-methyltransferase fibrillarin [Homo sapiens]	6556211.727	7300391.753	10029531.08	7118632.825	9605870.519	8193608.26	7081379.117	5771195.971	9143815.221
FBLN1	fibulin-1 isoform D precursor [Homo sapiens]	70632.19061	75079.04315	56238.66451	82124.69178	60154.07221	78752.88108	38598.35563	15581.32746	45356.66542
FBN1	fibrillin-1 precursor [Homo sapiens]	6161.502098	32408.54115	38198.3824	13338.17442	33580.16744	42256.51126	36949.4398	31366.6371	39110.47896
FBXL17	F-box/LRR-repeat protein 17 [Homo sapiens]	0	0	0	0	0	0	26469.99659	20642.8041	0
FBXL18	F-box/LRR-repeat protein 18 [Homo sapiens]	72653.61035	54788.67991	113635.2446	71550.23261	98199.45109	0	0	71946.15817	35893.175
FBXO11	F-box only protein 11 isoform 4 [Homo sapiens]	30593.26867	28268.68643	0	34860.05471	0	29225.90486	0	0	0
FBXO2	F-box only protein 2 [Homo sapiens]	1656409.654	1904837.067	2594669.844	1536578.862	2911414.198	2241608.317	1949390.363	1290670.823	2415629.088
FBXO21	F-box only protein 21 isoform 1 [Homo sapiens]	0	0	0	0	0	0	77976.41769	72758.13341	77020.83716
FBXO22	F-box only protein 22 isoform a [Homo sapiens]	656506.8717	789900.892	1145159.685	936997.1073	1186879.428	627202.1429	708544.8122	448461.9109	724068.5088
FBXO28	F-box only protein 28 isoform a [Homo sapiens]	77491.93568	68573.39284	75393.124	70703.89516	68594.4629	77003.94439	64077.9743	0	83723.92542
FBXO3	F-box only protein 3 isoform 1 [Homo sapiens]	0	0	0	0	0	0	195577.5148	0	54685.43713

FBXO30	F-box only protein 30 [Homo sapiens]	55235.02174	43967.48226	0	32965.38696	65692.57991	101762.325	59461.14404	0	96194.71413
FBXO38	F-box only protein 38 isoform a [Homo sapiens]	0	0	0	0	0	0	0	0	0
FBXO4	F-box only protein 4 isoform 1 [Homo sapiens]	220646.414	349188.4609	228521.9262	387744.311	249059.6457	351831.1063	296516.94	234692.8298	152869.8743
FBXO6	F-box only protein 6 [Homo sapiens]	232567.9825	105066.6461	188812.6565	195069.6321	173805.3949	192081.3008	100119.1624	97368.15336	78543.32944
FBXO7	F-box only protein 7 isoform 1 [Homo sapiens]	237259.1418	325859.1151	372417.3487	240857.9378	395079.0954	344674.4704	210542.0956	267414.8583	316506.5559
FBXO9	F-box only protein 9 isoform 2 [Homo sapiens]	0	65405.40895	59716.10565	89609.80065	112945.4884	0	49721.86199	31647.95063	0
FBXO9	F-box only protein 9 isoform 1 [Homo sapiens]	0	65405.40895	59716.10565	89609.80065	112945.4884	0	49721.86199	31647.95063	0
FBXW11	F-box/WD repeat-containing protein 11 isoform C [Homo sapiens]	82317.95908	0	91965.78724	52898.1439	61703.14145	66597.57513	84156.96711	72013.75912	69325.71026
FCF1	rRNA-processing protein FCF1 homolog [Homo sapiens]	215882.8273	0	376935.5	126422.0524	394838.2921	219096.2612	127577.2275	133241.3751	0
FCHO2	FCH domain only protein 2 isoform a [Homo sapiens]	115829.9604	165486.0781	125900.715	114201.0686	62887.34833	120836.8096	153479.1711	112045.6117	133933.1983
FDFT1	squalene synthase [Homo sapiens]	625887.8637	753795.3859	831240.4027	822998.4523	824503.7812	773679.2772	839415.7255	711472.0503	731543.7517
FDPS	farnesyl pyrophosphate synthase isoform b [Homo sapiens]	12094498.77	14643979.53	15264400.49	10231919.91	16205188.99	14149123.03	15151519.09	11984236.57	17184955.84
FDX1	adrenodoxin, mitochondrial precursor [Homo sapiens]	610795.84	336732.0417	875035.6133	753320.8267	812101.18	472677.55	453502.8308	808760.1967	400661.9251
FDXR	NADPH:adrenodoxin oxidoreductase, mitochondrial isoform 3 precursor [Homo s	222028.4112	226247.941	210737.9772	210027.615	327366.2287	286229.9461	256192.0809	210803.7307	286834.4443
FECH	ferrochelatase, mitochondrial isoform a precursor [Homo sapiens]	314520.152	144281.8987	258673.6172	188941.1558	172810.0534	278768	243134.1527	367454.5142	475797.3224
FEN1	flap endonuclease 1 [Homo sapiens]	5152718.616	5754097.737	6903253.127	5764129.467	6861236.676	6100514.89	4715219.486	5347655.021	6138957.925
FER	tyrosine-protein kinase Fer [Homo sapiens]	0	26887.19518	64614.31456	0	49415.58621	16930.67926	10212.65204	0	10057.0158
FERMT1	fermitin family homolog 1 [Homo sapiens]	153573.4173	35913.93624	150665.042	155783.195	165660.2616	175696.9561	183554.0181	143569.858	70884.36169
FERMT2	fermitin family homolog 2 isoform 2 [Homo sapiens]	1123492.174	1228706.195	1425002.336	1173601.122	1452401.081	1312457.201	1227645.963	1080080.768	1316260.245
FEZ2	fasciculation and elongation protein zeta-2 isoform 2 [Homo sapiens]	0	0	0	0	0	0	309599.7077	0	288549.9865
FGB	fibrinogen beta chain isoform 1 preproprotein [Homo sapiens]	0	0	0	0	0	0	0	0	0
FGFR1OP	FGFR1 oncogene partner isoform a [Homo sapiens]	56909.20302	145888.5683	84378.0004	78870.62624	89125.00141	196440.3264	201051.8324	61145.0453	112796.4564
FGG	fibrinogen gamma chain isoform gamma-B precursor [Homo sapiens]	0	0	0	0	0	0	0	0	0
FH	fumarate hydratase, mitochondrial [Homo sapiens]	8340334.292	8632027.7	9291437.895	9209991.626	10983094.11	8723167.292	8060550.357	7816789.444	8299715.31
FHAD1	forkhead-associated domain-containing protein 1 [Homo sapiens]	658428.2524	673081.849	0	0	845919.7683	731628.6993	0	0	0
FHL1	four and a half LIM domains protein 1 isoform 3 precursor [Homo sapiens]	350488.3587	183716.0953	0	247356.4648	929195.481	113618.6316	428632.2435	185879.0395	527057.438
FHL1	four and a half LIM domains protein 1 isoform 1 [Homo sapiens]	350488.3587	183716.0953	0	247356.4648	929195.481	113618.6316	428632.2435	185879.0395	527057.438
FHL2	four and a half LIM domains protein 2 [Homo sapiens]	694014.396	773216.8669	840624.9186	722715.1689	773649.9703	772462.3411	871014.9411	631532.7209	1033958.647
FHL3	four and a half LIM domains protein 3 isoform 1 [Homo sapiens]	313457.4325	365995.064	588286.6606	389986.6399	430299.6322	473588.2131	528033.2938	416034.5719	351093.4261
FHOD1	FH1/FH2 domain-containing protein 1 [Homo sapiens]	168876.7637	446447.7694	250045.5995	197011.1777	247320.5537	526227.1537	190205.3361	195352.6807	243135.8446
FIBP	acidic fibroblast growth factor intracellular-binding protein isoform a [Ho	0	0	0	0	0	0	170291.9274	144234.4281	0
FIP1L1	pre-mRNA 3'-end-processing factor FIP1 isoform 1 [Homo sapiens]	574999.6324	534030.3543	622265.811	576030.3703	784231.2	725905.3881	541488.5365	584568.8721	560947.1571
FIS1	mitochondrial fission 1 protein [Homo sapiens]	2253997.209	2278501.589	2065132.176	1521106.682	1861825.43	4030632.349	2255041.208	2273899.457	2639384.869
FKBP10	peptidyl-prolyl cis-trans isomerase FKBP10 precursor [Homo sapiens]	2225043.795	2171921.406	2596521.902	2088298.953	2601193.641	2051653.77	2116486.196	1863700.177	2397647.786
FKBP14	peptidyl-prolyl cis-trans isomerase FKBP14 precursor [Homo sapiens]	270117.5722	398073.9889	349291.3681	175145.4889	221844.9816	353904.0074	326564.5583	290323.707	415735.2784
FKBP15	FK506-binding protein 15 [Homo sapiens]	171547.2162	158052.5126	202603.3022	191073.4397	215940.8531	122544.6524	105006.0316	127588.2002	89859.10772
FKBP1A	peptidyl-prolyl cis-trans isomerase FKBP1A isoform a [Homo sapiens]	18379556.6	22992343.21	33739138.12	19989165.12	35312590.33	24655576.19	22002297.52	18422095.86	21443678.91
FKBP2	peptidyl-prolyl cis-trans isomerase FKBP2 precursor [Homo sapiens]	7482488.759	7939858.105	8976226.691	5139627.781	9054128.364	8082988.264	8426864.683	5936065.277	4478838.138
FKBP3	peptidyl-prolyl cis-trans isomerase FKBP3 [Homo sapiens]	4408931.692	5229561.009	5449725.634	4975803.027	5794184.614	4997927.485	4690443.403	3811328.725	5321275.787
FKBP4	peptidyl-prolyl cis-trans isomerase FKBP4 [Homo sapiens]	6106598.477	7087237.621	8673983.808	7786435.79	8429468.379	8134717.86	7309530.341	6504465.023	7610092.827
FKBP5	peptidyl-prolyl cis-trans isomerase FKBP5 isoform 1 [Homo sapiens]	780700.3273	513294.6198	817279.3991	684753.2022	766237.307	704315.6053	674413.9802	643030.7278	706330.4899
FKBP8	peptidyl-prolyl cis-trans isomerase FKBP8 [Homo sapiens]	1475180.167	1655668.181	2126785.179	1851000.618	2206897.778	1808275.094	1515823.777	1419089.717	2049310.499
FKBP9	peptidyl-prolyl cis-trans isomerase FKBP9 precursor [Homo sapiens]	539114.3509	664236.3341	585017.6182	614312.8324	602636.6273	607757.0977	614030.4364	358611.28	590315.6903
FLAD1	FAD synthase isoform 1 [Homo sapiens]	582397.0976	697360.0937	848370.1288	667308.6102	863331.1722	718120.9649	704306.3366	556214.2707	674575.6824
FLCN	folliculin isoform 1 [Homo sapiens]	35831.55003	0	45353.89286	36000.95936	39866.54957	0	0	0	0
FLG2	filaggrin-2 [Homo sapiens]	55736.64044	44279.00605	35140.79718	5467.897463	109220.191	28401.05978	11297.95667	43453.90692	17494.82029
FLII	protein flightless-1 homolog isoform 1 [Homo sapiens]	0	11955.25943	248901.8349	165211.1317	256674.3339	196931.6018	195373.1523	168473.8031	147703.588
FLII	protein flightless-1 homolog isoform 3 [Homo sapiens]	0	11955.25943	248901.8349	165211.1317	256674.3339	196931.6018	195373.1523	168473.8031	147703.588
FLNA	filamin-A isoform 2 [Homo sapiens]	2434831.867	2962067.613	3354912.673	2748198.493	3520109.111	3012825.203	2820523.33	2537131.084	3109406.182
FLNB	filamin-B isoform 1 [Homo sapiens]	1494191.6	1766358.15	1933346.195	1786806.71	1999950.591	1856462.757	1777513.748	1555116.727	1779309.41
FLNC	filamin-C isoform a [Homo sapiens]	590018.0744	700097.5369	728486.2256	635647.7725	829919.5217	750584.4178	671697.0228	663818.974	673306.0319
FLOT1	flotillin-1 [Homo sapiens]	936781.1591	947319.5088	1253309.41	1025533.619	1271606.396	1126446.163	979871.2347	821104.9005	1042445.959
FLOT2	flotillin-2 [Homo sapiens]	983725.5654	844097.58	1342015.439	1052824.496	1354204.084	1115275.303	954181.3027	711942.82	836457.7978
FLVCR1	feline leukemia virus subgroup C receptor-related protein 1 [Homo sapiens]	274717.486	476298.9551	391025.8291	312624.8381	390131.711	518301.422	444726.0652	73426.21731	446105.7691
FLYWCH2	FLYWCH family member 2 [Homo sapiens]	2008075.378	1970714.461	2967326.432	1992335.044	3378486.585	2263324.768	1809177.285	1603916.042	1849015.158
FMNL1	formin-like protein 1 [Homo sapiens]	74869.01947	93097.35851	117734.3055	91228.64865	99982.0286	97437.09629	84020.78986	98280.66744	74838.16009
FMNL2	formin-like protein 2 [Homo sapiens]	27165.98377	35950.8232	43544.55411	27255.24892	0	0	31680.44048	28939.41991	29254.43028
FMR1	fragile X mental retardation protein 1 isoform ISO1 [Homo sapiens]	64210.95062	121533.9724	179374.2406	158761.3364	262578.1035	218020.5398	207923.9707	101657.6574	117608.0922
FN1	fibronectin isoform 1 preproprotein [Homo sapiens]	0	0	0	0	0	0	28512.29621	60236.72937	14114.46736
FN3K	fructosamine-3-kinase [Homo sapiens]	0	161209.1354	93264.05066	69727.48459	116410.8058	51626.40496	36864.37139	0	124969.1033
FN3KRP	ketosamine-3-kinase [Homo sapiens]	542287.4035	534464.111	654444.4196	413243.8043	533740.6096	419761.1415	389805.9774	236048.0351	478644.1908
FNBP1	formin-binding protein 1 [Homo sapiens]	48369.88758	85817.66584	0	38890.91036	57067.99246	67568.52779	64737.87623	75858.05694	64365.81096
FNBP1L	formin-binding protein 1-like isoform 3 [Homo sapiens]	53104.97887	60164.57	51100.24736	36005.3794	0	0	34037.50121	72029.20049	24161.43442
FNBP4	formin-binding protein 4 [Homo sapiens]	53848.60531	144081.8715	120666.7656	152771.103	165061.0802	155184.0373	146871.5755	134285.322	125222.7274
FNDC3A	fibronectin type-III domain-containing protein 3A isoform 1 [Homo sapiens]	515835.5336	141546.5216	168412.965	136965.835	114656.2727	145648.6919	176450.8685	135665.161	124397.7317
FNDC3B	fibronectin type III domain-containing protein 3B [Homo sapiens]	28372.5517	34017.64235	37292.23337	30915.47331	13149.9375	27183.2681	30064.55541	25250.77125	22665.53541
FNTA	protein farnesyltransferase/geranylgeranyltransferase type-1 subunit alpha	503227.5193	496798.8385	616785.5937	541648.0759	627210.885	565364.4183	426354.1435	495338.5088	540500.5307
FOCAD	focadhesin [Homo sapiens]	59953.88564	71945.90469	77681.73268	68916.07693	89718.20577	56286.3864	53636.36366	55512.94279	72532.37645
FOLR1	folate receptor alpha precursor [Homo sapiens]	9244929.324	8829478.689	8680256.355	9758105.32	10724265.07	8590103.526	9409257.96	8301791.94	10637813.88
FOSL2	fos-related antigen 2 [Homo sapiens]	101307.1376	593916.9715	384539.3372	247108.0963	149781.055	235764.7798	194128.0688	201515.3991	226636.2729
FOXC1	forkhead box protein C1 [Homo sapiens]	202333.2602	0	262031.8023	186853.4877	308470.9221	163273.4959	0	0	0
FOXK1	forkhead box protein K1 [Homo sapiens]	193860.487	232815.7686	274639.3945	243569.1967	225659.7954	284640.3255	149612.9335	246060.3013	142443.7323
FOXRED1	FAD-dependent oxidoreductase domain-containing protein 1 [Homo sapiens]	404118.1367	467167.0818	468250.4691	445774.6307	464989.8549	433527.8926	396948.1615	321987.2952	367242.0329
FPGS	folylpolyglutamate synthase, mitochondrial isoform a precursor [Homo sapien	270457.2697	290732.3512	291240.5258	332326.6828	293924.7937	334259.4364	267527.1147	301922.7845	303569.4382
FPGT	fucose-1-phosphate guanylyltransferase isoform 1 [Homo sapiens]	108466.4787	113518.3282	157614.1765	0	187668.5095	142120.7559	112649.6416	93206.59005	119680.4982
FRG1	protein FRG1 [Homo sapiens]	0	0	0	0	0	0	0	269212.4898	202450.2293
FRMD3	FERM domain-containing protein 3 isoform 1 [Homo sapiens]	59511.2561	0	17590.6862	70970.44295	0	27309.00259	35113.00311	0	71196.29382
FRS2	fibroblast growth factor receptor substrate 2 [Homo sapiens]	0	0	0	0	0	0	7074.475356	6481.162773	0
FRYL	protein furry homolog-like [Homo sapiens]	17810.2255	19825.12542	21493.00541	12335.40206	22476.3571	14416.73279	18410.65086	14381.38225	15154.18767
FSCN1	fascin [Homo sapiens]	12631871.03	13459630.19	17483366.24	13999896.93	17474225.15	14347037.27	13493012.23	11052340.78	14629704.57
FTH1	ferritin heavy chain [Homo sapiens]	2019973.558	2276908.685	2619429.4	2321862.518	2630147.164	2648087.449	2137431.065	1938798.738	1491594.642
FTL	ferritin light chain [Homo sapiens]	742113.9001	434578.7414	1042335.51	744921.3843	245948.4757	787361.6893	582627.8774	633757.1036	226049.0556
FTO	alpha-ketoglutarate-dependent dioxygenase FTO [Homo sapiens]	260618.1534	233234.6568	348456.4052	275553.8172	311389.4818	238506.3144	203980.1135	172684.9935	183088.4081
FTSJ1	putative tRNA (cytidine(32)/guanosine(34)-2'-O)-methyltransferase isoform a	1657555.296	134876.5833	51982.30339	0	212099.386	101925.1268	1213902.991	0	665662.4271
FTSJ3	pre-rRNA processing protein FTSJ3 [Homo sapiens]	491549.3198	619943.1505	744215.912	615844.1984	794039.1693	617363.0211	575011.7765	492245.7096	682569.6278
FTSJD2	cap-specific mRNA (nucleoside-2'-O-)-methyltransferase 1 [Homo sapiens]	86211.4563	74237.358	555685.3005	135282.1472	274797.5786	127707.7713	104789.2766	66036.8963	1669554.508
FUBP1	far upstream element-binding protein 1 [Homo sapiens]	6410018.123	6970425.397	8130890.309	6971504.167	8404967.627	7180398.49	7216590.569	5846617.672	7989841.319
FUBP3	far upstream element-binding protein 3 [Homo sapiens]	1799197.535	2239847.649	2564454.251	2251320.416	2735125.997	2368021.769	2210599.336	1974087.246	2522949.345
FUK	L-fucose kinase [Homo sapiens]	0	0	0	0	0	0	16306.80308	15827.83254	0
FUNDC1	FUN14 domain-containing protein 1 [Homo sapiens]	1147238.792	1526355.51	1568047.969	1325436.063	1660898.219	1432860.771	0	1182256.367	1406751.616
FUNDC2	FUN14 domain-containing protein 2 [Homo sapiens]	632308.9231	679230.7012	747412.0121	470752.0513	615872.5128	562226.819	0	711706.7232	574112.4615
FUOM	fucose mutarotase isoform 1 [Homo sapiens]	171115.3467	183650.342	192854.6509	178161.1262	251310.1062	245850.0342	202549.6509	153693.5885	188590.2594
FUS	RNA-binding protein FUS isoform 1 [Homo sapiens]	10727871.58	10548161.35	14132400.92	12144200.63	15854393.53	10365458.3	11307030.37	9780131.787	12859409.97
FXN	frataxin, mitochondrial isoform 1 preproprotein [Homo sapiens]	197160.0284	183323.0682	339961.767	160500.4091	372941.3864	225469.8072	308649.5043	112376.0256	259570.4773
FXR1	fragile X mental retardation syndrome-related protein 1 isoform a [Homo sap	646494.2092	592122.1867	924815.6568	614308.7786	880061.9413	786343.1059	635879.6985	431859.2004	607639.1661
FXR2	fragile X mental retardation syndrome-related protein 2 [Homo sapiens]	454178.3783	567868.5264	624836.1975	575754.7957	561164.6297	620383.7036	523665.0736	454645.6848	590774.8678
FYCO1	FYVE and coiled-coil domain-containing protein 1 [Homo sapiens]	85432.03831	46550.60857	124054.0301	87086.00668	59379.79146	97787.80879	99004.46235	119043.9181	71674.69711
FYTTD1	UAP56-interacting factor isoform 1 [Homo sapiens]	177513.1041	151930.1807	151757.2568	270297.6889	222924.33	310940.0811	90607.54813	174340.9733	163942.8583
FZR1	fizzy-related protein homolog isoform 1 [Homo sapiens]	0	0	0	0	0	0	32904.64086	0	38144.00381
G3BP1	ras GTPase-activating protein-binding protein 1 [Homo sapiens]	7531667.78	9068545.757	9501119.91	8992892.458	9833444.791	9297326.107	8622179.305	7340949.367	8941122.062
G3BP2	ras GTPase-activating protein-binding protein 2 isoform b [Homo sapiens]	1942436.913	2003377.182	2539527.411	2053765.327	2657966.325	2237723.663	2038661.187	1564535.455	2051120.724
G6PC3	glucose-6-phosphatase 3 [Homo sapiens]	267449.7363	445369.0068	1182793.342	490767.6662	1576136.685	609260.7895	499612.0771	338851.6525	1216329.807
G6PD	glucose-6-phosphate 1-dehydrogenase isoform b [Homo sapiens]	1990805.755	0	2263059.956	2168757.717	2472696.544	2250904.271	2041131.388	1818653.978	2136414.793
GAA	lysosomal alpha-glucosidase preproprotein [Homo sapiens]	276022.7559	269468.8982	305385.1702	274848.3077	381946.2728	310541.6277	291056.9274	156603.728	268364.3428
GABARAP	gamma-aminobutyric acid receptor-associated protein [Homo sapiens]	0	914598.5444	1113461.06	949577.6876	0	1090083.89	0	0	0
GABARAPL1	gamma-aminobutyric acid receptor-associated protein-like 1 [Homo sapiens]	0	0	0	0	0	0	0	259370.0417	819597.0977
GABARAPL2	gamma-aminobutyric acid receptor-associated protein-like 2 [Homo sapiens]	998047.3818	418491.9464	1444969.581	1302980	1627705.711	1062315.91	823883.8036	1312914.594	764864.17
GABPA	GA-binding protein alpha chain [Homo sapiens]	373737.7986	354080.9766	404418.3011	347219.6114	396128.1738	334841.1301	200034.4666	346517.0541	323561.6269
GABPB1	GA-binding protein subunit beta-1 isoform beta 1 [Homo sapiens]	0	84643.0825	485295.1125	91105.31375	439105.7525	0	0	102177.175	118194.675
GADD45GIP1	growth arrest and DNA damage-inducible proteins-interacting protein 1 [Homo	889697.0927	1094995.391	1392311.394	973115.0784	1246329.561	1297334.579	978767.6461	803132.567	781555.0882
GAGE12H	G antigen 12H [Homo sapiens]	6567488.121	0	7060774.909	0	8128627.933	6927272.858	0	0	0
GAGE2B	G antigen 2B/2C [Homo sapiens]	0	0	0	0	0	0	0	14098418.71	16291752.84
GAGE7	G antigen 12G [Homo sapiens]	12399626.05	14670597.52	20899211.64	0	21540092.15	15113414.76	11865427.83	10847280.34	2255054.182
GAK	cyclin-G-associated kinase [Homo sapiens]	127337.9436	133354.4026	155166.5075	121917.9824	124494.5146	103708.9911	91288.09835	74368.06769	177727.1154
GALE	UDP-glucose 4-epimerase [Homo sapiens]	1063167.55	996685.0193	1204622.412	1145729.339	1261435.374	1140908.494	1063679.204	853959.605	1068642.84
GALK1	galactokinase [Homo sapiens]	1295147.853	2067129.677	1592089.874	1572553.64	1641316.207	1674443.112	1644713.36	1492909.312	1807371.16
GALK2	N-acetylgalactosamine kinase isoform 1 [Homo sapiens]	275998.9575	300362.1284	504318.0309	417891.4083	519820.0448	390604.934	390850.0084	338239.8428	433595.8244
GALM	aldose 1-epimerase [Homo sapiens]	679089.313	464041.8304	396498.5863	259264.179	586460.9529	517681.2577	467714.9217	260597.5609	418090.1783
GALNT1	polypeptide N-acetylgalactosaminyltransferase 1 [Homo sapiens]	86731.81813	45216.49333	92432.97058	0	53000.39778	40786.34333	54625.96667	51873.18333	85362.84551
GALNT10	polypeptide N-acetylgalactosaminyltransferase 10 [Homo sapiens]	0	0	0	0	0	0	9390.332976	0	8184.164065

GALNT2	polypeptide N-acetylgalactosaminyltransferase 2 precursor [Homo sapiens]	1344295.673	1617650.874	1938816.521	1601538.51	1924748.466	1751975.643	1652581.396	1293622.527	1628241.111
GALNT7	N-acetylgalactosaminyltransferase 7 [Homo sapiens]	69200.31084	98170.36502	0	93691.20532	101073.2146	93307.20152	71121.5827	79467.45817	86883.7443
GAMT	guanidinoacetate N-methyltransferase isoform a [Homo sapiens]	1881425.758	2095906.868	2307094.629	1514970.103	2338447.525	2193580.036	2026261.397	809548.7738	2033611.772
GAN	gigaxonin [Homo sapiens]	0	29486.58188	61854.02183	0	56210.04655	20035.78521	0	0	0
GANAB	neutral alpha-glucosidase AB isoform 2 precursor [Homo sapiens]	695210.9868	4795514.623	5504108.994	0	0	776102.2723	5159385.813	4262017.039	570907.1274
GAPDH	glyceraldehyde-3-phosphate dehydrogenase isoform 2 [Homo sapiens]	1589143.656	973799.944	1415277.301	495798.385	1924021.731	831628.9593	1055204.164	911831.7218	999096.045
GAPDH	glyceraldehyde-3-phosphate dehydrogenase isoform 1 [Homo sapiens]	1589143.656	973799.944	1415277.301	495798.385	1924021.731	831628.9593	1055204.164	911831.7218	999096.045
GAPVD1	GTPase-activating protein and VPS9 domain-containing protein 1 [Homo sapier	350483.0557	393783.8708	450648.487	393247.5257	415100.9685	423088.405	392657.5838	328471.9992	401806.6172
GAR1	H/ACA ribonucleoprotein complex subunit 1 [Homo sapiens]	1902222.882	551170.0231	1482405.881	2109991.116	1951010.882	2075423.114	2094313.339	1133910.586	916495.1496
GARS	glycine--tRNA ligase precursor [Homo sapiens]	2831334.316	2809844.979	3616893.422	2271989.808	3815783.14	2946067.665	2734483.805	2294140.049	3003782.145
GART	trifunctional purine biosynthetic protein adenosine-3 isoform 1 [Homo sapi	2190645.754	2440202.034	2371297.809	2659469.885	2554772.043	2541587.318	2478583.38	2245176.631	2867181.236
GATA2	endothelial transcription factor GATA-2 isoform 1 [Homo sapiens]	0	0	0	0	0	0	56725.71469	0	20107.59208
GATAD2A	transcriptional repressor p66-alpha [Homo sapiens]	239247.5749	228069.3356	326363.2062	180403.9917	247511.0977	213923.4336	263158.5169	224952.8595	333778.1641
GATAD2B	transcriptional repressor p66-beta [Homo sapiens]	481445.5232	405855.9792	789343.6844	568847.302	880400.8948	582549.6228	468112.5316	468909.3612	587627.872
GATSL1	GATS-like protein 1 [Homo sapiens]	0	0	0	0	0	0	0	0	0
GATSL3	GATS-like protein 3 [Homo sapiens]	0	0	0	0	0	0	173319.4938	0	284379.5625
GBA	glucosylceramidase isoform 1 precursor [Homo sapiens]	596634.616	741430.1225	718287.9491	201836.2031	963527.8207	557864.3654	741273.3124	460373.1218	600655.0834
GBAS	protein NipSnap homolog 2 isoform 1 [Homo sapiens]	869100.1588	601646.4427	1189527.142	949898.3038	1496706.773	345681.8727	1563698.766	249787.0344	1421216.82
GBE1	1,4-alpha-glucan-branching enzyme [Homo sapiens]	1448732.887	1141322.955	1727351.331	1042161.554	1847315.407	1103321.403	1126141.041	810144.0866	1168056.787
GBF1	Golgi-specific brefeldin A-resistance guanine nucleotide exchange factor 1	182981.9375	191275.4024	244888.9144	193286.3157	261688.7666	201946.3333	200336.3188	175626.582	208150.4714
GBP2	interferon-induced guanylate-binding protein 2 [Homo sapiens]	0	0	0	0	0	0	0	110277.1155	85223.96554
GCAT	2-amino-3-ketobutyrate coenzyme A ligase, mitochondrial isoform 1 precursor	346997.463	227554.2513	419711.2494	325192.7066	508871.5278	273249.0139	314325.2472	251682.0908	419158.0069
GCC1	GRIP and coiled-coil domain-containing protein 1 [Homo sapiens]	0	0	0	0	0	0	27621.8775	0	10852.52224
GCC2	GRIP and coiled-coil domain-containing protein 2 [Homo sapiens]	11635.97678	10280.88807	0	7349.261775	19686.79167	9360.690422	10143.45018	9847.569995	11178.88838
GCDH	glutaryl-CoA dehydrogenase, mitochondrial isoform a precursor [Homo sapiens	733640.7656	950996.1388	1041346.178	1021672.596	855776.735	1160815.128	982955.345	1018395.041	1152801.963
GCFC2	GC-rich sequence DNA-binding factor 2 isoform 1 [Homo sapiens]	22533.16652	133763.8365	17061.06178	110416.045	32387.01657	0	0	0	0
GCHFR	GTP cyclohydrolase 1 feedback regulatory protein [Homo sapiens]	983060.9531	2194392.375	1821318.438	1744341.469	2114069.846	2358256.047	1797572.813	0	2250432.253
GCLC	glutamate--cysteine ligase catalytic subunit isoform a [Homo sapiens]	1078386.798	431919.8537	647031.5934	613182.1914	691366.8926	654239.1377	693128.9152	468519.8947	544972.5381
GCLM	glutamate--cysteine ligase regulatory subunit [Homo sapiens]	3747775.453	3222452.616	5147696.754	3740242.227	4646254.224	3951295.756	4140472.085	3775734.33	4257178.568
GCN1L1	translational activator GCN1 [Homo sapiens]	451421.8991	441652.5083	524944.0585	456210.928	545007.1276	549543.6219	503758.0528	372255.9571	485429.1197
GCSH	glycine cleavage system H protein, mitochondrial precursor [Homo sapiens]	6194606.84	0	4951738.419	7987765.934	7547200.522	0	8561588.626	0	6058941.603
GDA	guanine deaminase isoform a [Homo sapiens]	2531920.463	2992113.032	3389702	2677174.816	3477185.079	2869606.739	2803513.137	2569727.175	3083284.617
GDAP1	ganglioside-induced differentiation-associated protein 1 isoform a [Homo sa	245495.4667	334075.55	119719.5917	257678.56	304648.6533	264460.215	280541.2709	236290.54	194068.4927
GDAP2	ganglioside-induced differentiation-associated protein 2 isoform a [Homo sa	59338.21622	93347.98346	95710.71184	48463.74932	43391.02872	81039.22973	38394.04865	97383.93514	101763.827
GDE1	glycerophosphodiester phosphodiesterase 1 [Homo sapiens]	94137.64565	0	0	123411.4783	111260.5424	178586.113	248937.1758	60739.87717	236595.4924
GDI1	rab GDP dissociation inhibitor alpha [Homo sapiens]	778831.6916	881711.6416	1137901.943	807338.3325	1183336.328	923152.0952	893416.3675	776297.4554	933019.2789
GDI2	rab GDP dissociation inhibitor beta isoform 1 [Homo sapiens]	9739849.162	8673111.607	11933410.46	9311848.539	11552627.02	10117660.61	9750784.743	8846968.455	9248798.403
GDPD3	glycerophosphodiester phosphodiesterase domain-containing protein 3 [Homo s	261636.6206	395400.8587	203401.569	305103.9772	262045.3214	380302.5963	323389.6793	465783.8155	379373.7694
GDPGP1	GDP-D-glucose phosphorylase 1 [Homo sapiens]	193287.3287	270825.5712	213362.7579	329968.6985	392382.0146	287993.0538	222293.4349	208558.2896	235400.9256
GEMIN2	gem-associated protein 2 isoform alpha [Homo sapiens]	686533.7417	682035.9799	448337.9436	695052.0244	832558.1728	588035.555	648085.2884	458967.3522	660314.2113
GEMIN4	gem-associated protein 4 [Homo sapiens]	713308.3989	930077.0657	493349.9449	699011.7997	524696.0503	748346.9006	866035.4323	891185.4635	466091.266
GEMIN5	gem-associated protein 5 isoform 1 [Homo sapiens]	333875.3265	427222.3408	427040.7544	429403.977	458308.4679	429112.8942	383627.3022	364942.8516	442830.7315
GEMIN6	gem-associated protein 6 [Homo sapiens]	673476.9458	656326.3255	234828.6981	579286.8553	160371.6604	713712.4145	648487.4034	0	829122.1574
GET4	Golgi to ER traffic protein 4 homolog [Homo sapiens]	971790.3562	1555896.229	1087268.158	1239603.06	1570467.631	1340949.876	1264815.531	1082765.579	1252361.066
GFER	FAD-linked sulfhydryl oxidase ALR [Homo sapiens]	141742.5333	163577.132	203783.9446	95871.16489	288766.5638	100087.8225	115382.2514	187281.7913	119060.9548
GFM1	elongation factor G, mitochondrial [Homo sapiens]	1462274.792	1682084.845	2013764.785	1675931.115	2028043.102	1788717.502	1710949.497	1164666.905	1778618.438
GFM2	ribosome-releasing factor 2, mitochondrial isoform 1 [Homo sapiens]	88918.00158	46816.78674	137734.853	69674.36588	176373.7198	85289.7934	87232.31959	72848.01677	86218.90907
GFPT1	glutamine--fructose-6-phosphate aminotransferase [isomerizing] 1 isoform 1	928536.8296	1191963.172	1153470.433	977360.1502	1103769.782	1066901.124	1095627	974960.8213	1322660.399
GGA1	ADP-ribosylation factor-binding protein GGA1 isoform 1 [Homo sapiens]	90836.19173	114558.9475	102693.7542	109692.8541	144989.1759	114700.8738	74578.99971	63820.79659	75239.01519
GGA2	ADP-ribosylation factor-binding protein GGA2 [Homo sapiens]	66357.10417	341991.3529	507867.1353	82429.88206	106168.3002	412410.027	82693.62868	0	169627.8475
GGCT	gamma-glutamylcyclotransferase isoform 1 [Homo sapiens]	3338860.314	4143979.5	3586758.331	4410471.839	5806638.061	4708805.58	3728943.795	2759547.908	4692085.67
GGCX	vitamin K-dependent gamma-carboxylase isoform 1 [Homo sapiens]	116011.5071	236066.1968	321932.7952	286830.238	183224.333	316184.2033	291318.6736	127267.8168	0
GGH	gamma-glutamyl hydrolase precursor [Homo sapiens]	612950.1061	4138825.048	578313.6449	2387919.262	3026584.317	2333149.507	705931.1257	204925.8292	2633184.702
GGPS1	geranylgeranyl pyrophosphate synthase [Homo sapiens]	206141.0015	192592.9839	275382.8831	72747.78226	317311.5575	200654.7935	320389.7802	176306.1265	259149.5414
GGT1	gamma-glutamyltranspeptidase 1 precursor [Homo sapiens]	0	0	0	0	0	0	848345.1722	702254.0864	0
GGT2	PREDICTED: gamma-glutamyltranspeptidase 2 isoform 1 [Homo sapiens]	0	0	0	0	0	0	0	0	0
GHITM	growth hormone-inducible transmembrane protein [Homo sapiens]	1056723.827	1667412.571	1861346.873	1440748.553	1903621.013	1082329.598	854174.9104	948124.5415	1681242.545
GID8	glucose-induced degradation protein 8 homolog [Homo sapiens]	1431061.826	1438057.992	1838386.914	1314333.376	1800675.698	1553727.117	1500061.099	1279803.006	1733783.874
GIGYF2	PERQ amino acid-rich with GYF domain-containing protein 2 isoform a [Homo s	0	0	0	0	0	0	96488.08614	67561.64948	156740.1137
GINS1	DNA replication complex GINS protein PSF1 [Homo sapiens]	338713.3364	296289.0361	170366.1105	256646.9536	146344.3224	294445.7823	282672.4381	0	37412.87081
GINS2	DNA replication complex GINS protein PSF2 [Homo sapiens]	581021.6143	638875.925	134282.325	535600.1714	379031.4071	0	0	400117.8321	889825.75
GINS3	DNA replication complex GINS protein PSF3 isoform a [Homo sapiens]	333481.7612	331885.248	235075.6919	359364.5747	341461.3924	390812.2887	343022.1599	355990.6323	359670.0197
GINS4	DNA replication complex GINS protein SLD5 [Homo sapiens]	198344.2857	242813.4592	0	367000.8648	0	102751.2398	230644.8554	225469.6757	147149.4968
GIPC1	PDZ domain-containing protein GIPC1 isoform 1 [Homo sapiens]	1044090.346	851582.363	1854107.397	1354337.914	1703711.386	1276105.042	1145430.561	1120260.472	1175957.258
GIT1	ARF GTPase-activating protein GIT1 isoform 1 [Homo sapiens]	88880.66472	121898.1559	127575.8455	116109.5023	121686.597	82870.66398	135746.7033	92697.48327	122635.8328
GIT2	ARF GTPase-activating protein GIT2 isoform 1 [Homo sapiens]	90558.26882	93675.0609	83586.53017	90314.17215	81488.50304	106477.8052	59023.8564	26483.85748	62509.06211
GK	glycerol kinase isoform d [Homo sapiens]	174158.0824	156117.4101	150024.2411	206408.4846	221382.7879	190190.006	123456.9073	24298.32093	68039.32146
GLA	alpha-galactosidase A precursor [Homo sapiens]	309731.4913	469858.3336	477528.8721	422587.5602	513944.8773	497992.27	415968.502	194384.0333	496274.7954
GLB1	beta-galactosidase isoform a preproprotein [Homo sapiens]	400734.0145	268754.2673	433173.5642	382463.4275	502563.1305	471355.142	409920.6069	421156.2835	445695.9971
GLCCI1	glucocorticoid-induced transcript 1 protein [Homo sapiens]	221099.9406	153666.0015	246490.527	209368.2033	263286.298	238110.4074	105245.9308	155001.1809	124949.6071
GLE1	nucleoporin GLE1 isoform 1 [Homo sapiens]	73005.2455	80726.77507	125017.0518	225839.3361	120297.3554	94874.34077	493964.6777	286318.5594	129756.8447
GLG1	Golgi apparatus protein 1 isoform 1 precursor [Homo sapiens]	175572.7208	177672.3085	215707.1523	176314.4047	225660.9126	183190.776	146233.8302	111486.4543	134593.1684
GLMN	glomulin [Homo sapiens]	304231.5132	220942.9101	388866.3511	346954.6568	474694.6507	337978.2168	392440.0632	312855.1694	286789.1731
GLO1	lactoylglutathione lyase [Homo sapiens]	14420467.84	16712423.65	18650386.28	17368941.07	16582825.6	18509984.92	17554628.97	11955461.91	18942927.55
GLOD4	glyoxalase domain-containing protein 4 [Homo sapiens]	2440007.453	3053491.145	3533547.301	3109648.012	3574130.202	3322594.194	3324941.022	2586087.881	3174945.685
GLRX	glutaredoxin-1 [Homo sapiens]	1173153.939	2555658.897	1549328.403	1252103.447	2097662.503	2364997.88	2191345.681	1387601.508	2473246.419
GLRX3	glutaredoxin-3 [Homo sapiens]	3724231.079	3870856.843	5142291.368	3931313.63	5006338.339	4105078.777	3954669.61	3607408.342	4287127.653
GLRX5	glutaredoxin-related protein 5, mitochondrial precursor [Homo sapiens]	2277644.36	2406559.342	3651632.182	2290493.369	3091660.149	3606186.471	3560702.587	3004793.624	3022918.742
GLS	glutaminase kidney isoform, mitochondrial isoform 2 [Homo sapiens]	1405961.66	1598132.121	1300443.262	988514.4587	1281712.902	1616665.56	1228726.192	975624.7788	1465680.984
GLS	glutaminase kidney isoform, mitochondrial isoform 1 precursor [Homo sapiens	1405961.66	1598132.121	1300443.262	988514.4587	1281712.902	1616665.56	1228726.192	975624.7788	1465680.984
GLT25D1	procollagen galactosyltransferase 1 precursor [Homo sapiens]	663663.2128	990300.5528	867397.77	932544.2204	867717.226	936644.96	926726.4168	850812.354	921656.4752
GLT8D1	glycosyltransferase 8 domain-containing protein 1 [Homo sapiens]	0	233444.4783	247056.6912	175033.9919	0	266249.0393	144730.6504	156448.3053	310362.9281
GLTP	glycolipid transfer protein [Homo sapiens]	0	0	0	0	0	0	0	801098.6715	939010.9477
GLTPD1	glycolipid transfer protein domain-containing protein 1 [Homo sapiens]	180930.4125	10495.54779	147159.1765	107863.8426	0	193372.1206	59746.49412	201600.7971	67312.43971
GLTSCR2	glioma tumor suppressor candidate region gene 2 protein [Homo sapiens]	274469.4431	270152.7375	294053.1742	284267.6756	342590.9777	353771.1997	306198.2985	232607.2909	292070.4855
GLUD1	glutamate dehydrogenase 1, mitochondrial precursor [Homo sapiens]	2000106.116	2472555.24	2691757.013	2097222.003	2758138.023	2496599.956	2488825.632	2028645.187	2807202.291
GLUL	glutamine synthetase [Homo sapiens]	41268.96171	98928.875	68737.44982	0	183107.9894	166835.375	33718.73988	0	101132.6426
GLYR1	putative oxidoreductase GLYR1 [Homo sapiens]	158604.1153	183297.943	242027.7985	191026.2322	261801.7472	207640.38	144498.0327	106805.4608	195936.6949
GM2A	ganglioside GM2 activator isoform 1 precursor [Homo sapiens]	0	372854.9091	601202.2727	506603.7636	635105.6727	723056.0115	584300.2	221198.1455	0
GMDS	GDP-mannose 4,6 dehydratase isoform 1 [Homo sapiens]	537981.8948	539271.7469	678478.9	363762.6833	676251.225	446640.1094	433748.4917	278556.2591	721834.0781
GMEB2	glucocorticoid modulatory element-binding protein 2 [Homo sapiens]	150560.9419	104037.2046	168073.0524	216004.2227	189350.3728	99636.56317	134091.0161	150492.8618	102941.952
GMFB	glia maturation factor beta [Homo sapiens]	2340608.923	1721053.358	4247004.538	3071663.169	4442744.346	1500426.335	2443031.4	3296867.431	1439967.812
GMIP	GEM-interacting protein [Homo sapiens]	7367.334758	11765.15487	0	7198.1949	0	10661.0499	9721.376248	9707.114806	2555.728165
GMNN	geminin [Homo sapiens]	443090.4925	802223.8899	397580.9648	561470.5119	437498.375	595519.4221	453481.6131	299542.6786	192538.496
GMPPA	mannose-1-phosphate guanyltransferase alpha [Homo sapiens]	525923.4001	660627.9973	466007.6074	541462.4257	496158.8105	472104.5775	459446.0625	361883.2584	581236.8849
GMPPB	mannose-1-phosphate guanyltransferase beta isoform 1 [Homo sapiens]	614672.4508	607040.8508	783301.2752	496013.3596	694327.4874	658741.7989	637833.5153	341761.1485	742080.0802
GMPR2	GMP reductase 2 isoform 1 [Homo sapiens]	834021.0211	543952.9314	1172552.692	928076.0015	914596.6135	737005.063	791310.9489	761061.6767	587876.7873
GMPS	GMP synthase [glutamine-hydrolyzing] [Homo sapiens]	2727383.104	3242672.706	3891400.659	2945643.674	3730574.789	3368087.047	3219997.531	2870376.563	3595841.14
GNA11	guanine nucleotide-binding protein subunit alpha-11 [Homo sapiens]	687869.7401	476488.4591	747390.6592	729320.4842	735714.9039	763822.1099	718047.5901	653343.7255	775423.7789
GNA13	guanine nucleotide-binding protein subunit alpha-13 [Homo sapiens]	510203.2672	323707.5819	310474.5345	2736801.278	638667.0178	354344.221	495023.5272	3327931.158	435208.4799
GNAI1	guanine nucleotide-binding protein G(i) subunit alpha-1 isoform 1 [Homo sap	240780.92	442557.6117	391024.7217	346018.3983	0	408384.24	227186.2517	322288.4	436729.5775
GNAI2	guanine nucleotide-binding protein G(i) subunit alpha-2 isoform 1 [Homo sap	2030570.077	1606385.374	2826294.295	2429496.581	2953063.708	2443341.191	2337039.523	1825637.841	2379044.366
GNAI3	guanine nucleotide-binding protein G(k) subunit alpha [Homo sapiens]	1806977.912	2323572.785	2215041.665	2246303.118	2589793.085	1852792.925	2077745.938	1740845.443	2467483.029
GNAQ	guanine nucleotide-binding protein G(q) subunit alpha [Homo sapiens]	219772.6184	209551.9194	244406.8109	0	249232.3886	182330.5159	244961.0798	217819.8446	106897.2807
GNAS	protein GNAS isoform XLas [Homo sapiens]	472865.2408	576613.128	660981.8678	547868.6764	696179.1325	521849.678	494379.2879	396230.5	558786.5841
GNB1	guanine nucleotide-binding protein G(I)/G(S)/G(T) subunit beta-1 [Homo sap	4858685.134	5447481.457	5540380.026	5634820.077	5398030.659	5791871.552	5550649.947	4487316.233	5415923.954
GNB2	guanine nucleotide-binding protein G(I)/G(S)/G(T) subunit beta-2 [Homo sap	2187656.132	2033131.869	3044158.506	2709956.56	2867354.072	2930457.961	2912325.088	2243416.715	2911679.932
GNB2L1	guanine nucleotide-binding protein subunit beta-2-like 1 [Homo sapiens]	30451358.2	32424575.42	38985109.57	35759243.69	42591803.85	35552692.82	33361080.8	28552449.21	32948713.74
GNB4	guanine nucleotide-binding protein subunit beta-4 [Homo sapiens]	0	603518.8178	501917.4096	524904.933	394636.9159	376676.1472	609393.7103	378813.4268	650103.2796
GNE	bifunctional UDP-N-acetylglucosamine 2-epimerase/N-acetylmannosamine kinase	435258.7359	856190.4956	1113188.334	459906.4407	1114344.002	974531.7244	579892.7852	492180.0715	588577.9196
GNG12	guanine nucleotide-binding protein G(I)/G(S)/G(O) subunit gamma-12 precurso	9537110.062	2992220.859	13149287.77	10383136.27	13130550	14129434.66	9431909.015	9820543.009	10151121.69
GNG5	guanine nucleotide-binding protein G(I)/G(S)/G(O) subunit gamma-5 precursor	6557991.545	13036540.6	1504719.803	7774094.252	3949308.752	3882412.714	4868143.552	9611814.379	13126960.91
GNL1	guanine nucleotide-binding protein-like 1 [Homo sapiens]	580511.7305	675528.2571	713067.8265	603226.0783	645353.3553	660079.1288	633331.077	508978.5	551722.823
GNL2	nucleolar GTP-binding protein 2 [Homo sapiens]	143901.0342	137565.7676	143030.9756	80757.04179	175458.2763	177990.7808	164612.6574	137726.2436	237137.2764
GNL3	guanine nucleotide-binding protein-like 3 isoform 1 [Homo sapiens]	997265.9598	1054406.861	1216070.723	1172642.46	1128172.452	1226681.181	1203963.57	948422.9432	1369180.507
GNL3L	guanine nucleotide-binding protein-like 3-like protein [Homo sapiens]	253310.4258	242949.0279	276390.9636	309412.6006	292855.6668	259113.3195	463877.2523	410124.423	424602.5453
GNPAT	dihydroxyacetone phosphate acyltransferase [Homo sapiens]	130989.5963	123268.4203	240227.2611	180803.4998	230439.6085	202243.5516	197868.7569	145485.176	151451.9604
GNPDA1	glucosamine-6-phosphate isomerase 1 [Homo sapiens]	1759593.389	2297088.957	2079183.422	2360197.323	2826725.444	4856114.22	4965458.276	1977886.323	2524035.581
GNPDA2	glucosamine-6-phosphate isomerase 2 isoform 1 [Homo sapiens]	0	0	0	0	0	0	0	0	0
GNPNAT1	glucosamine 6-phosphate N-acetyltransferase [Homo sapiens]	3090537.917	3556554.335	3985923.603	3344939.128	4280415.867	3643625.657	3357832.788	3028749.719	3551389.788

GNPTAB	N-acetylglucosamine-1-phosphotransferase subunits alpha/beta precursor [Hor	0	0	0	0	0	0	12085.95868	7975.022751	0
GNS	N-acetylglucosamine-6-sulfatase precursor [Homo sapiens]	819790.2049	451943.0979	495948.3988	574802.4098	515447.1395	643911.7478	583133.2321	472795.7711	527562.0281
GOLGA1	Golgin subfamily A member 1 [Homo sapiens]	134725.1223	38466.86	0	69673.06857	72979.51429	41786.95609	42264.2184	0	44862.4
GOLGA2	Golgin subfamily A member 2 [Homo sapiens]	299835.1705	350917.6728	381449.1756	334314.528	391564.8003	274980.7433	294744.6026	159135.6786	281895.2474
GOLGA3	Golgin subfamily A member 3 isoform 1 [Homo sapiens]	41062.25711	62917.76171	54948.5145	322482.0371	80874.72637	54876.65166	49021.13944	40091.55385	75187.92399
GOLGA4	Golgin subfamily A member 4 isoform 1 [Homo sapiens]	40108.37438	37782.96185	64103.15716	44542.85384	62140.10642	63204.78361	28610.5713	78551.14431	33058.43026
GOLGA5	Golgin subfamily A member 5 [Homo sapiens]	26114.27374	24168.61404	30184.91701	0	26027.4897	23704.23214	18909.25436	10524.88089	17621.16274
GOLGA7	Golgin subfamily A member 7 isoform a [Homo sapiens]	524905.0443	390670.41	673394.2328	589561.9598	1051030.961	816417.8212	850347.6466	742175.6422	740855.2155
GOLGB1	Golgin subfamily B member 1 isoform 1 [Homo sapiens]	73475.38351	63334.34729	49215.14292	42404.71693	146432.0064	44358.84227	58340.62444	0	41595.08684
GOLIM4	Golgi integral membrane protein 4 [Homo sapiens]	520555.6922	630674.2155	1658814.209	1411975.891	1909719.314	576709.7378	678725.3608	450468.9095	1564007.819
GOLM1	Golgi membrane protein 1 [Homo sapiens]	383721.3892	515741.8997	431004.9828	538964.5957	506030.6606	543914.9175	392651.7598	255329.1136	425897.2968
GOLPH3	Golgi phosphoprotein 3 [Homo sapiens]	1747512.439	2086477.357	2271815.281	2464572.127	2194758.929	1949348.864	1682612.957	1392340.53	1686867.897
GOLPH3L	Golgi phosphoprotein 3-like [Homo sapiens]	145193.4726	554959.1577	41883.99904	0	404433.7385	0	391356.1192	476166.2308	506307.4077
GOLT1B	vesicle transport protein GOT1B [Homo sapiens]	4124910.442	4594934.756	2440038.077	2265580.371	2770568.453	2368282.379	4659187.919	2092198.273	5318449.14
GON4L	GON-4-like protein isoform a [Homo sapiens]	0	270790.1115	0	14554.83156	17487.15953	14784.3476	0	0	0
GOPC	Golgi-associated PDZ and coiled-coil motif-containing protein isoform a [Ho	181298.7289	175938.6684	235810.1758	2169251.19	0	135599.0078	172566.4339	114492.8476	209294.607
GORASP1	Golgi reassembly-stacking protein 1 [Homo sapiens]	278643.5	0	437214.075	405322.0313	444541.9875	475194.0719	378495.9625	287186.575	0
GORASP2	Golgi reassembly-stacking protein 2 isoform 1 [Homo sapiens]	2870830.41	3355618.158	3865027.416	3445094.355	4160942.583	3532138.26	3342364.292	2850144.848	3578373.335
GOSR1	Golgi SNAP receptor complex member 1 isoform 1 [Homo sapiens]	486207.7967	594987.9922	520167.2714	486084.1979	525348.8934	542848.681	550312.0597	359141.4262	647542.19
GOSR2	Golgi SNAP receptor complex member 2 isoform B [Homo sapiens]	241272.1826	335427.6026	400164.288	481936.3245	476550.9399	465440.9782	509580.5112	573358.8399	280366.4138
GOT1	aspartate aminotransferase, cytoplasmic [Homo sapiens]	2460764.036	2455279.411	2817514.889	2420211.004	3233750.927	2593746.479	2329725.595	2179593.435	2392908.036
GOT2	aspartate aminotransferase, mitochondrial precursor [Homo sapiens]	6233544.869	8532625.801	8555831.932	7983713.375	8542905.915	8023328.46	8913339.693	7190252.335	9992014.08
GPAA1	glycosylphosphatidylinositol anchor attachment 1 protein [Homo sapiens]	187988.7827	2279152.019	279699.0528	280899.4021	200201.4547	487387.6248	244865.0913	376009.2725	2663475.217
GPATCH11	G patch domain-containing protein 11 isoform 1 [Homo sapiens]	15844.77521	34110.20733	0	0	208766.4948	25292.42414	117756.0602	0	20936.80577
GPATCH4	G patch domain-containing protein 4 isoform 1 [Homo sapiens]	639023.8387	638332.6743	903182.0157	736231.2874	933473.2147	776952.711	687613.3524	606524.8707	701788.7257
GPATCH8	G patch domain-containing protein 8 [Homo sapiens]	109814.0323	47426.79706	108924.3512	246077.3627	39568.59738	118349.8412	7137.558929	24246.68936	25384.97945
GPD1L	glycerol-3-phosphate dehydrogenase 1-like protein [Homo sapiens]	773984.917	770713.7872	1074301.526	877545.4894	1227576.462	844966.9121	823316.5915	702499.1857	996329.5773
GPD2	glycerol-3-phosphate dehydrogenase, mitochondrial precursor [Homo sapiens]	913666.4745	1044733.139	1433775.05	917204.5616	1489657.039	917219.3222	956071.2106	870857.4589	1283276.116
GPHN	gephyrin isoform 1 [Homo sapiens]	253829.932	313116.4997	355868.5392	308186.6111	337382.1808	350075.7055	300789.6637	234419.0729	335000.0256
GPI	glucose-6-phosphate isomerase isoform 2 [Homo sapiens]	32392446.03	34790176.78	41583095.38	35436069.93	44895152.72	38077925.44	34275025.93	29038091.68	35398376.44
GPKOW	G patch domain and KOW motifs-containing protein [Homo sapiens]	781507.3599	950934.3633	881559.857	862000.93	1018787.28	1036564.692	918882.2217	906270.0676	1169182.048
GPN1	GPN-loop GTPase 1 isoform a [Homo sapiens]	477064.8211	498205.6696	649415.6681	425209.5212	611216.9533	558883.972	421793.0553	248375.8093	230886.8254
GPN2	GPN-loop GTPase 2 [Homo sapiens]	0	0	0	0	0	0	98535.473	0	194027.2068
GPN3	GPN-loop GTPase 3 isoform 3 [Homo sapiens]	0	0	0	0	0	0	420934.2033	424036.0278	0
GPR107	protein GPR107 isoform 1 precursor [Homo sapiens]	85259.92172	98449.31313	0	66457.57071	123400.4154	93334.17929	0	0	0
GPR89A	Golgi pH regulator A isoform 1 [Homo sapiens]	262764.0461	417068.9415	50438.16623	369123.1312	464563.0745	265540.6223	407436.3741	0	354721.4078
GPR98	G-protein coupled receptor 98 precursor [Homo sapiens]	0	0	0	0	0	0	0	0	0
GPRC5A	retinoic acid-induced protein 3 [Homo sapiens]	3649789.8	4931943.776	3808965.155	3893438.237	5342204.171	3837388.143	6201565.182	3613613.52	6040122.083
GPRIN1	G protein-regulated inducer of neurite outgrowth 1 [Homo sapiens]	23325.15802	5908.463689	0	21740.6413	21303.60326	0	11186.90109	0	11069.62065
GPS1	COP9 signalosome complex subunit 1 isoform 1 [Homo sapiens]	1248045.977	1396723.616	1511284.156	727219.2948	829573.5964	707196.8453	451431.3906	490695.9709	1371995.858
GPX1	glutathione peroxidase 1 isoform 1 [Homo sapiens]	2079071.536	2801641.007	2398197.455	2013609.548	2984966.293	3069619.181	2267719.948	1935488.214	3043120.787
GPX4	phospholipid hydroperoxide glutathione peroxidase, mitochondrial isoform C	505645.9915	0	170242.4873	452270.4915	448672.8242	324542.6208	401055.5254	340066.6467	0
GPX8	probable glutathione peroxidase 8 [Homo sapiens]	1170466.597	1393287.968	1592702.061	888885.5731	1850822.747	1061000.587	1013985.89	1158195.333	2252887.921
GRAMD1A	GRAM domain-containing protein 1A isoform 1 [Homo sapiens]	0	87465.58238	176321.403	105627.7739	135555.5223	107494.5785	51462.55989	0	86420.44828
GRAMD1B	GRAM domain-containing protein 1B [Homo sapiens]	182517.12	93615.08668	154339.254	231964.7067	225038.7014	56200.00706	97926.65671	141232.3162	132974.325
GRAMD3	GRAM domain-containing protein 3 isoform 1 [Homo sapiens]	121870.7387	66278.42623	108263.8026	75969.59907	79789.37639	50332.37534	74708.65918	98030.5929	133633.403
GRB2	growth factor receptor-bound protein 2 isoform 1 [Homo sapiens]	1908439.18	1958244.627	2442393.656	1908653.641	2096325.385	1985633.464	1981924.391	1823697.435	2087434.075
GRHPR	glyoxylate reductase/hydroxypyruvate reductase [Homo sapiens]	2137043.424	1099399.279	2600906.898	1278086.527	2607499.528	1643498.283	2339938.653	2075962.208	1176398.493
GRIPAP1	GRIP1-associated protein 1 isoform 1 [Homo sapiens]	133324.7308	102554.9153	213523.6889	94140.54423	110435.4805	157813.5158	78988.59072	81547.90854	159428.7438
GRN	granulins precursor [Homo sapiens]	44928.71945	238117.3575	165305.9452	335404.5401	114903.837	323711.4901	265974.6285	90498.67065	288005.1428
GRPEL1	grpE protein homolog 1, mitochondrial precursor [Homo sapiens]	3338026.606	3784956.928	4448380.742	3685627.559	4685429.428	3989345.059	4440463.297	3509360.809	4026324.372
GRPEL2	grpE protein homolog 2, mitochondrial precursor [Homo sapiens]	144142.4721	37711.24958	116884.4152	32406.35491	63429.04018	0	0	0	0
GRSF1	G-rich sequence factor 1 isoform 1 [Homo sapiens]	1198659.797	1377615.787	1791086.807	1547625.323	1665417.47	1543550.762	1571402.837	1337464.141	1619863.72
GRWD1	glutamate-rich WD repeat-containing protein 1 [Homo sapiens]	2440671.03	2844754.593	3504273.327	2822182.412	3485582.165	2976237.098	3120533.11	2788516.382	3094522.019
GSDMD	gasdermin-D [Homo sapiens]	63538.73103	69748.95196	121879.681	102851.9459	76825.22993	80807.86127	0	0	0
GSK3A	glycogen synthase kinase-3 alpha [Homo sapiens]	227227.1695	347586.4309	233648.2955	320051.7495	254283.1337	312539.2368	323198.0928	273726.1311	341951.0455
GSK3B	glycogen synthase kinase-3 beta isoform 1 [Homo sapiens]	586082.7343	584282.8935	829356.8389	666107.1725	792366.1659	688495.9677	608939.8275	640297.6653	898376.3311
GSKIP	GSK3-beta interaction protein [Homo sapiens]	936889.3336	0	1275351.201	94054.05036	961942.7022	317625.0278	0	0	0
GSN	gelsolin isoform a precursor [Homo sapiens]	106144.4195	52173.40669	113196.493	46280.63644	0	54151.48123	43237.26542	43947.78542	49725.80458
GSPT1	eukaryotic peptide chain release factor GTP-binding subunit ERF3A isoform 1	2990545.307	3415290.35	4029438.865	3669733.354	3766997.976	3580705.046	3402936.522	3076314.329	3942942.052
GSR	glutathione reductase, mitochondrial isoform 1 precursor [Homo sapiens]	892608.2505	898720.7467	1197774.002	1009766.553	1233145.676	1044135.024	945025.0761	965551.2511	922845.1723
GSS	glutathione synthetase [Homo sapiens]	947131.7939	1269834.082	1255883.291	1132515.343	1295634.87	1385154.938	1266668.416	1079887.363	1265751.667
GSTCD	glutathione S-transferase C-terminal domain-containing protein isoform 1 [I	0	0	0	0	0	0	0	0	0
GSTK1	glutathione S-transferase kappa 1 isoform a [Homo sapiens]	3377410.645	0	3016324.846	3419928.269	3288314.892	2609825.71	0	3031900.467	3191462.608
GSTM1	glutathione S-transferase Mu 1 isoform 1 [Homo sapiens]	3152349.408	3431149.106	3683300.936	3424455.088	4054771.656	3809158.907	3439470.518	2675320.042	3388632.281
GSTM3	glutathione S-transferase Mu 3 [Homo sapiens]	16890554.69	20436250.88	23540632.72	19777915.35	23046553.14	20728158.74	21066035.3	16769543.1	20304554.17
GSTM4	glutathione S-transferase Mu 4 isoform 1 [Homo sapiens]	575141.8883	506518.7402	550735.2482	590375.6997	588718.641	591418.5554	608783.6729	328335.8519	757369.5523
GSTO1	glutathione S-transferase omega-1 isoform 1 [Homo sapiens]	6125876.363	6659311.381	7869080.453	6737519.293	8304901.021	6914466.467	6289170.67	5457572.693	7466631.633
GSTT1	glutathione S-transferase theta-1 [Homo sapiens]	327041.5697	746399.0371	33185.10336	385044.9533	747197.2836	0	327755.4533	159964.489	607316.3681
GSTT2B	glutathione S-transferase theta-2B [Homo sapiens]	418809.9943	575483.3672	982820.7273	672413.9886	905414.2017	616065.7045	581500.7935	550143.3247	358882.2458
GSTZ1	maleylacetoacetate isomerase isoform 1 [Homo sapiens]	281944.0862	295608.3231	286777.8986	354860.9764	558248.0203	484884.4257	479107.3041	506387.6605	500119.2027
GTF2A1	transcription initiation factor IIA subunit 1 isoform 1 [Homo sapiens]	0	0	0	0	0	0	0	774115.2857	951138.9683
GTF2A2	transcription initiation factor IIA subunit 2 [Homo sapiens]	0	0	0	0	0	0	239288.1281	0	410653.619
GTF2B	transcription initiation factor IIB [Homo sapiens]	642670.8618	681505.8475	771047.4662	690192.0537	736366.1542	726947.1239	565278.1689	444047.7723	586772.1537
GTF2E1	general transcription factor IIE subunit 1 [Homo sapiens]	176082.9564	183272.0388	194376.6123	150280.7925	251721.7907	264380.8257	167896.8707	35011.24569	463502.1139
GTF2E2	transcription initiation factor IIE subunit beta [Homo sapiens]	216996.346	201273.1164	220408.8493	136872.0066	222216.2434	162626.4156	173672.9247	214557.2701	210347.9691
GTF2F1	general transcription factor IIF subunit 1 [Homo sapiens]	648497.6963	767545.4581	902672.2593	738061.3108	937395.1402	784056.4178	733008.8266	641072.7021	807134.8776
GTF2F2	general transcription factor IIF subunit 2 [Homo sapiens]	3111375.21	1294289.356	1551867.416	1373528.915	892133.0008	861266.2587	813709.9611	1129198.575	1237453.148
GTF2H1	general transcription factor IIH subunit 1 [Homo sapiens]	94860.30381	165973.4126	161590.4423	114705.5465	104435.5221	131570.8987	119154.678	169778.9957	262807.8094
GTF2H2C	general transcription factor IIH subunit 2-like protein [Homo sapiens]	162065.2165	206756.0225	113794.809	194759.8489	204607.7073	260927.3741	218677.7554	199160.4964	193841.8912
GTF2H3	general transcription factor IIH subunit 3 isoform a [Homo sapiens]	104872.9863	333137.7275	194662.6579	94064.5175	243697.8988	252692.12	141756.3088	58431.26625	93557.58063
GTF2H4	general transcription factor IIH subunit 4 [Homo sapiens]	177090.6061	89896.17878	417066.7079	140050.3087	372089.8846	106210.1809	300750.2126	96157.2432	188493.9504
GTF2I	general transcription factor II-I isoform 1 [Homo sapiens]	1089306.224	1054838.479	1336812.231	1052928.112	0	1197474.18	929658.8898	789736.0829	1076197.816
GTF3C1	general transcription factor 3C polypeptide 1 [Homo sapiens]	58582.2009	68415.34936	74109.53459	73273.3261	90931.54749	66323.70852	83293.54342	65727.15189	108116.1354
GTF3C2	general transcription factor 3C polypeptide 2 [Homo sapiens]	141302.1048	119188.8325	122343.1868	153238.1658	164435.3679	200255.2065	172490.8041	141424.7945	155006.6192
GTF3C3	general transcription factor 3C polypeptide 3 isoform 1 [Homo sapiens]	156123.6636	154865.771	185608.6004	209110.5671	264870.8809	229802.9855	151101.8662	141982.21	168845.6439
GTF3C4	general transcription factor 3C polypeptide 4 [Homo sapiens]	161697.7664	238468.6334	259039.0105	192870.5001	275400.4279	237875.6105	217356.8446	193895.6132	234166.638
GTF3C5	general transcription factor 3C polypeptide 5 isoform 1 [Homo sapiens]	265239.5603	291656.8077	354386.4631	297219.6174	401740.9958	318254.1736	253182.1378	243324.4923	314390.1902
GTPBP1	GTP-binding protein 1 [Homo sapiens]	403880.3141	309207.3234	498136.4325	389983.8834	525610.6145	448678.7048	465319.9972	323533.5241	479170.1104
GTPBP10	GTP-binding protein 10 isoform 2 [Homo sapiens]	19476.23419	172717.7488	193533.3743	167536.312	257305.1134	160280.8939	161979.8808	139755.3438	122899.8774
GTPBP3	tRNA modification GTPase GTPBP3, mitochondrial isoform IV [Homo sapiens]	109897.6062	59919.96029	85549.27353	81644.24853	73745.85	83551.96335	91889.64247	101204.8964	78436.42641
GTPBP4	nucleolar GTP-binding protein 1 [Homo sapiens]	975201.0363	894627.5298	1256104.59	960569.3789	1322890.065	1253481.927	989892.8039	941624.5815	1035537.476
GTPBP6	putative GTP-binding protein 6 [Homo sapiens]	97449.34859	2490440.318	219030.3178	123728.2441	180788.8263	122392.1643	3663896.83	102687.3427	2875874.332
GTSE1	G2 and S phase-expressed protein 1 [Homo sapiens]	60676.45447	0	87993.59425	0	77435.33466	70191.59665	39286.05872	31162.39457	0
GUK1	guanylate kinase isoform c [Homo sapiens]	0	0	643904.0149	541092.7129	685106.5099	575172.1052	0	0	0
GUSB	beta-glucuronidase precursor [Homo sapiens]	174566.356	342858.4068	261180.4532	252841.2236	241485.7466	282472.7829	230487.621	202155.458	246385.7782
GXYLT1	glucoside xylosyltransferase 1 isoform 1 [Homo sapiens]	115083.9021	55810.10241	123241.4744	91491.71687	106958.7093	122857.4096	118765.2274	71760.46837	0
GYG1	glycogenin-1 isoform 2 [Homo sapiens]	1570355.332	0	0	1810887.349	1867767.02	1853353.492	2113785.889	0	2273121.638
GYG1	glycogenin-1 isoform 1 [Homo sapiens]	1570355.332	0	0	1810887.349	1867767.02	1853353.492	2113785.889	0	2273121.638
GYS1	glycogen [starch] synthase, muscle isoform 1 [Homo sapiens]	881072.0802	917814.9779	1209440.161	966804.8458	1219512.814	946249.1985	644359.5042	798748.3969	961283.7092
H1F0	histone H1.0 [Homo sapiens]	0	0	0	0	0	0	407009.3403	420415.1627	0
H1FX	histone H1x [Homo sapiens]	1480988.386	1529151.662	1288927.382	1536099.414	1369426.951	1635418.035	1433670.575	125496.9641	2512218.308
H2AFX	histone H2A.x [Homo sapiens]	0	0	0	0	0	0	24746.0322	11968428.87	12239.19271
H2AFY	core histone macro-H2A.1 isoform 3 [Homo sapiens]	5091921.05	6023126.718	6485885.682	6361001.457	7288615.427	6707207.159	6366895.124	5247279.378	6530132.981
H2AFZ	histone H2A.Z [Homo sapiens]	15200550.17	28033239.79	27795540.65	26369944.42	31754622.16	25685795.79	26132446.21	20839490.94	27005731.74
H3F3B	histone H3.3 [Homo sapiens]	11840308.62	9351793.038	15751434.87	11579285.69	15782955.33	7513770.896	12031558.24	7762709.405	13465580.67
HACL1	2-hydroxyacyl-CoA lyase 1 [Homo sapiens]	269230.4585	242339.726	319821.0886	262436.9088	421070.147	336950.115	363820.6885	270338.6902	333335.4433
HADH	hydroxyacyl-coenzyme A dehydrogenase, mitochondrial isoform 1 precursor [Ho	4351537.314	4144990.35	5872031.539	4595696.758	6015930.314	5152991.74	4479479.822	3856267.18	4557209.401
HADHA	trifunctional enzyme subunit alpha, mitochondrial precursor [Homo sapiens]	1898027.046	1997151.181	2442763.55	2063654.262	2450928.676	2016482.722	1903051.211	1794235.205	2343555.598
HADHB	trifunctional enzyme subunit beta, mitochondrial precursor [Homo sapiens]	2327471.072	2560136.02	3154829.084	2578767.959	3293032.996	2719376.29	2672966.805	2190313.142	2892628.402
HAGH	hydroxyacylglutathione hydrolase, mitochondrial isoform 1 precursor [Homo s	447857.5879	452691.2477	683167.1287	503434.1164	419868.7001	461856.1402	478389.4163	334840.7222	513541.6369
HAL	histidine ammonia-lyase isoform 1 [Homo sapiens]	0	0	0	0	0	0	0	0	0
HARS	histidine--tRNA ligase, cytoplasmic isoform 1 [Homo sapiens]	1322123.531	1403677.408	1696102.165	1432583.924	1792385.533	1536722.909	1436694.898	1219105.169	1508711.807
HARS2	probable histidine--tRNA ligase, mitochondrial precursor [Homo sapiens]	192086.9176	175779.162	231148.4072	143565.9933	266525.5178	167730.5977	126603.9649	131240.6046	216813.3631
HAS1	hyaluronan synthase 1 [Homo sapiens]	0	0	0	0	0	0	2816012.77	92657.41866	0
HAT1	histone acetyltransferase type B catalytic subunit [Homo sapiens]	3183379.368	4118614.882	4363191.792	4138907.393	4714089.796	4110567.244	3751662.929	3213292.561	3942902.396
HAUS1	HAUS augmin-like complex subunit 1 [Homo sapiens]	0	205923.0506	125698.4426	305006.9293	47083.5239	312692.9857	256749.3732	251854.2739	36591.40246
HAUS2	HAUS augmin-like complex subunit 2 isoform 1 [Homo sapiens]	92008.9	0	137289.428	252434.9346	96296.34118	324030.4529	0	0	0
HAUS4	HAUS augmin-like complex subunit 4 isoform 1 [Homo sapiens]	56902.96235	68512.99133	143997.9417	87355.94355	23261.33057	56843.84639	60075.59262	104539.2831	93941.66867
HAUS5	HAUS augmin-like complex subunit 5 [Homo sapiens]	65686.62687	39554.81896	58515.00287	61779.71198	59328.92347	113689.1571	43850.9044	27540.66068	35011.40503

HAUS6	HAUS augmin-like complex subunit 6 isoform 1 [Homo sapiens]	31453.16687	21252.28918	28248.4199	35694.9443	54876.58549	83092.52137	70809.05959	66321.96731	18210.68847
HAUS7	HAUS augmin-like complex subunit 7 [Homo sapiens]	0	0	0	0	0	0	20476.81371	29494.99447	0
HAX1	HCLS1-associated protein X-1 isoform a [Homo sapiens]	721689.0632	642007.4973	741740.2222	721345.6335	651858.9541	813658.5399	592099.2489	510323.7026	214567.9335
HBA1	hemoglobin subunit alpha [Homo sapiens]	4349155.334	2502108.796	3799424.847	2235982.506	3591823.234	4631012.46	17169511.81	4430822.383	4242224.96
HBB	hemoglobin subunit beta [Homo sapiens]	1293180.143	2296817.444	4721146.096	2064496.637	7342038.152	1492253.587	13933043.24	2729522.192	1996020.904
HBG1	hemoglobin subunit gamma-1 [Homo sapiens]	585934.2478	774499.6636	0	182260.2909	0	901465.9773	1074510.145	302519.3909	945773.3545
HBS1L	HBS1-like protein isoform 1 [Homo sapiens]	234322.5925	276800.926	364423.3873	269500.6853	295760.1699	270060.0159	265755.6356	216752.0118	310348.6045
HCCS	cytochrome c-type heme lyase [Homo sapiens]	2761849.459	3284488.523	3719694.602	3446497.523	3572187.412	3325094.738	3396345.665	2667909.996	3645777.858
HCFC1	host cell factor 1 [Homo sapiens]	518484.5562	466862.8241	658188.852	543831.4478	740974.405	537304.5746	451013.1783	429105.2193	525927.2583
HDAC1	histone deacetylase 1 [Homo sapiens]	947663.6837	1461590.961	1718765.837	1297576.272	1684843.62	1417744.418	1288252.111	1059572.901	1055325.642
HDAC2	histone deacetylase 2 [Homo sapiens]	1454832.794	733993.5913	1710958.937	1113790.237	1764791.012	1179274.014	1210304.343	1098231.302	1374685.235
HDAC3	histone deacetylase 3 [Homo sapiens]	201179.3841	275559.6281	223028.7551	238105.9274	438895.8306	161585.7188	163626.8654	127700.6073	197538.8434
HDAC6	histone deacetylase 6 [Homo sapiens]	36008.62759	38774.01195	82600.67703	53790.17105	88019.14778	62156.53244	46089.04568	28168.32385	34951.74655
HDAC7	histone deacetylase 7 isoform a [Homo sapiens]	56668.09687	86030.42201	81731.9582	80549.94969	131212.918	140166.2616	95129.57019	34601.43492	99375.85604
HDDC2	HD domain-containing protein 2 [Homo sapiens]	617789.223	570483.4372	366484.5678	341317.4275	852304.171	466121.1735	301635.9186	268671.6853	746088.678
HDDC3	guanosine-3',5'-bis(diphosphate) 3'-pyrophosphohydrolase MESH1 [Homo sapien	571665.0234	262537.4354	500874.5357	617010.0089	1153159.504	217985.6463	0	0	0
HDGF	hepatoma-derived growth factor isoform a [Homo sapiens]	19267258.28	19526275.72	25157707.8	22839432.51	25604483.63	22814330.04	19338886.82	16481176.88	19488626.49
HDGFRP2	hepatoma-derived growth factor-related protein 2 isoform 1 [Homo sapiens]	496498.6876	270167.0596	659659.4945	467487.1879	678518.8695	460897.2553	445076.1317	357443.0856	264393.1811
HDHD1	pseudouridine-5'-monophosphatase isoform b [Homo sapiens]	1138863.724	1280926.61	2956064.025	1141810.193	2866249.563	1303929.81	1217546.318	959382.6592	1053978.438
HDHD2	haloacid dehalogenase-like hydrolase domain-containing protein 2 [Homo sap:	186221.6709	163264.75	234731.8865	105543.7692	0	258156.0223	190840.8393	167657.8903	276195.1493
HDLBP	vigilin isoform a [Homo sapiens]	831303.6591	765022.4506	937255.4491	945748.7862	990048.0429	800114.8942	782634.0966	1066532.771	827260.0559
HEATR1	HEAT repeat-containing protein 1 [Homo sapiens]	304989.987	320759.9649	362466.039	327967.3682	324226.251	339933.085	247696.4305	254961.189	299785.7645
HEATR2	HEAT repeat-containing protein 2 [Homo sapiens]	425961.3454	425049.4289	548258.9072	367024.7251	533435.8151	401574.9502	330995.0431	372821.7588	468064.1983
HEATR3	HEAT repeat-containing protein 3 [Homo sapiens]	134128.7593	147257.8847	135640.5628	178402.9174	78566.57209	159738.198	161846.0387	110846.5647	47339.8806
HEATR5A	HEAT repeat-containing protein 5A [Homo sapiens]	66597.14394	59448.35786	93669.663	77943.01849	68768.13541	73260.13713	66969.16641	44841.04904	53055.31222
HEATR5B	HEAT repeat-containing protein 5B [Homo sapiens]	31307.7597	30412.73355	32878.28581	36857.68775	44222.24627	30837.22201	29158.75709	24935.76036	25460.23416
HEATR6	HEAT repeat-containing protein 6 [Homo sapiens]	55454.77954	79060.35224	77188.30418	45272.36454	77517.44342	48587.3971	52092.15117	49110.43757	32569.71415
HEBP1	heme-binding protein 1 [Homo sapiens]	2797313.215	2626361.886	2347399.515	1689747.443	3031670.905	4044636.64	3731723.093	2133261.492	2241301.999
HEBP2	heme-binding protein 2 [Homo sapiens]	318261.3585	519070.2388	378045.9851	151769.5336	20109.97575	90340.49627	0	0	0
HECTD1	E3 ubiquitin-protein ligase HECTD1 [Homo sapiens]	87740.93424	103338.4636	131468.2276	90998.72948	112691.6393	96606.91132	95778.68781	77557.73438	120653.0083
HECTD3	E3 ubiquitin-protein ligase HECTD3 [Homo sapiens]	256507.2845	140120.5957	179291.0872	132614.0641	404125.7197	85018.09009	119264.2952	103426.5065	144130.4975
HECTD4	probable E3 ubiquitin-protein ligase HECTD4 [Homo sapiens]	6830.788441	0	7935.578263	6605.797214	8748.38034	89220.27945	5371.372829	5136.866905	0
HELB	DNA helicase B [Homo sapiens]	0	0	0	0	0	0	0	0	0
HELLS	lymphoid-specific helicase [Homo sapiens]	238578.8761	241895.8054	347692.003	333924.9286	317390.5135	289189.6862	286961.0832	189339.8576	270201.763
HERC1	probable E3 ubiquitin-protein ligase HERC1 [Homo sapiens]	3615.72866	6148.349385	6216.408848	7720.985882	4368.362572	10269.68728	6923.499981	2248.702566	0
HERC2	E3 ubiquitin-protein ligase HERC2 [Homo sapiens]	13309.66788	18696.33662	13962.56514	12626.08743	18534.85332	14375.5423	10444.62295	10847.25258	16307.88843
HERC4	probable E3 ubiquitin-protein ligase HERC4 isoform a [Homo sapiens]	375543.5132	285055.2004	519701.2966	377068.9471	501292.1753	394419.0986	370479.6839	307849.2253	321925.727
HERC5	E3 ISG15--protein ligase HERC5 [Homo sapiens]	0	0	0	0	0	0	0	34533.84528	20622.59051
HEXA	beta-hexosaminidase subunit alpha preproprotein [Homo sapiens]	685020.7465	588549.6017	735818.5365	870553.9428	753421.7755	853789.6384	881142.4245	672338.0969	602186.7518
HEXB	beta-hexosaminidase subunit beta preproprotein [Homo sapiens]	1356770.178	5308261.746	1675372.121	3465392.677	1794205.553	6760385.56	5864230.689	4659753.156	1508893.769
HEXIM1	protein HEXIM1 [Homo sapiens]	1361066.755	2005649.508	2034573.927	2090155.7	2348959.519	2111163.222	1830686.855	1524189.236	2099390.12
HEXIM2	protein HEXIM2 [Homo sapiens]	36787.74219	33486.83734	0	25024.16086	0	19185.83402	0	0	0
HFE	hereditary hemochromatosis protein isoform 1 precursor [Homo sapiens]	361158.5733	406279.8776	656005.323	491872.8472	607313.5208	607253.2546	548934.2546	256043.4741	0
HGS	hepatocyte growth factor-regulated tyrosine kinase substrate [Homo sapiens]	405022.1639	615796.1681	701826.8542	583709.4113	782082.9235	614154.1874	517908.3752	364197.1154	626363.345
HIBADH	3-hydroxyisobutyrate dehydrogenase, mitochondrial precursor [Homo sapiens]	2207076.727	2965415.269	2655098.167	2914007.261	2848673.689	2799492.211	2814565.416	2530566.693	3103313.345
HIBCH	3-hydroxyisobutyryl-CoA hydrolase, mitochondrial isoform 1 precursor [Homo	298393.2363	511677.2065	408703.324	289568.9653	470431.8386	530740.8921	479676.854	380006.8025	567715.0034
HIF1AN	hypoxia-inducible factor 1-alpha inhibitor [Homo sapiens]	254094.493	259365.6952	285391.3209	244309.5861	263461.6655	0	292184.0702	221520.1141	319883.5877
HIGD1A	HIG1 domain family member 1A, mitochondrial isoform b [Homo sapiens]	5243969.454	8214902.203	7288164.02	7994867.894	7583112.309	5271873.063	8250344.609	4712788.386	8150468.203
HIGD2A	HIG1 domain family member 2A, mitochondrial [Homo sapiens]	4049497.955	2584118.257	5437292.153	2800510.408	5412577.779	1112795.053	1268944.336	3780148.368	2652465.655
HINT1	histidine triad nucleotide-binding protein 1 [Homo sapiens]	19228379.66	23769128.47	30885613.04	23001355.55	27853930.8	26332229.14	25224741.39	21488030.33	28624666.73
HINT2	histidine triad nucleotide-binding protein 2, mitochondrial precursor [Homo	731713.6816	1073525.66	1554976.022	1737241.861	1594634.648	1735119.911	1791658.026	640511.6702	1483925.836
HINT3	histidine triad nucleotide-binding protein 3 [Homo sapiens]	0	0	0	0	0	0	387164.6308	0	472439.3269
HIP1	huntingtin-interacting protein 1 isoform 1 [Homo sapiens]	42369.42219	0	356218.7139	34460.5649	0	42018.10118	0	110606.8907	44327.94623
HIP1R	huntingtin-interacting protein 1-related protein [Homo sapiens]	20714.57041	49620.62	58992.28358	63933.00805	76935.75751	63421.98429	48365.27124	45207.40163	62920.53536
HIST1H1A	histone H1.1 [Homo sapiens]	1791953.101	2256978.565	1955589.443	2139849.43	2711081.743	1874151.583	2335303.415	1872230.197	1661013.324
HIST1H1B	histone H1.5 [Homo sapiens]	15656127.36	15525541.66	27814145.5	14651949.83	29053699.1	14749973.13	16164096.81	16844991.46	29931601.26
HIST1H1C	histone H1.2 [Homo sapiens]	6428751.564	10543668.48	16039728.35	9665811.979	11461780.34	7657722.729	18100028.44	15411898.44	9627483.879
HIST1H1D	histone H1.3 [Homo sapiens]	939337.2627	0	2450936.939	2250049.173	0	1162287.305	1026958.626	992716.104	2006564.633
HIST1H1E	histone H1.4 [Homo sapiens]	7380873.854	1325747.074	10429353.72	10434259.56	11146524.06	11126917.98	5897592.316	7599288.931	7743623.042
HIST1H2AA	histone H2A type 1-A [Homo sapiens]	0	0	0	0	0	0	0	0	0
HIST1H2AD	histone H2A type 1-D [Homo sapiens]	0	0	0	0	0	0	658508648.1	560502512.8	675393229.5
HIST1H2BB	histone H2B type 1-B [Homo sapiens]	44139100.74	48633409.95	81199028.75	75850993.83	77296429.15	58921954.69	53861650.92	56978747.22	56711588.22
HIST1H2BL	histone H2B type 1-L [Homo sapiens]	0	0	0	0	0	0	137696745.2	0	130626376.4
HIST1H3A	histone H3.1 [Homo sapiens]	11056905.99	6619425.43	2803205.208	14467903.7	50403575.39	44384762.63	39304813.47	23544610.23	10213474.16
HIST2H2BF	histone H2B type 2-F isoform b [Homo sapiens]	0	0	141713457	187846158.4	149664416.1	178020975.7	0	0	0
HIST2H3A	histone H3.2 [Homo sapiens]	24584261.29	13160112.22	31746609.25	19486490.3	29231101.28	27483517.14	21165723.94	11769022.97	20415624.71
HIST2H4A	histone H4 [Homo sapiens]	559072247.7	553179352.5	616408997.4	535634282.8	511842121.9	636566179.8	589804554.1	634097251.7	801128138.4
HK1	hexokinase-1 isoform HKI-ta/tb [Homo sapiens]	764990.4331	726640.7317	998569.7933	807878.4075	1042645.802	792754.4373	800438.4419	694664.9427	848108.7056
HK2	hexokinase-2 [Homo sapiens]	985098.8134	725652.7476	483620.2382	905040.0916	989971.7691	1044455.932	905185.5276	744782.8217	427093.717
HKDC1	putative hexokinase HKDC1 [Homo sapiens]	15088.72716	34379.92815	69619.64467	145948.9285	105701.7411	49179.9997	78158.04188	51994.61589	45778.98581
HLA-A	HLA class I histocompatibility antigen, A-1 alpha chain precursor [Homo sap	5676837.005	7226742.324	7068038.33	6981071.217	6829750.815	7357271.732	6623604.255	5588820.976	4698084.321
HLA-B	major histocompatibility complex, class I, B precursor [Homo sapiens]	3951518.261	4748933.828	5280778.819	7904820.519	4993424.26	4835860.075	8457164.637	3635271.042	4629618.819
HLA-C	HLA class I histocompatibility antigen, Cw-1 alpha chain precursor [Homo sa	2246318.905	3072579.014	1378078.564	3088546.045	2319144.775	3467629.545	3173951.711	2332927.738	2713870.394
HLCS	biotin--protein ligase [Homo sapiens]	0	66340.81405	70734.33058	47184.6157	66279.07231	59957.2624	34578.50491	0	62492.85331
HLTF	helicase-like transcription factor [Homo sapiens]	263673.8037	269813.7681	368129.8616	300844.453	455141.3111	382668.465	307922.0222	225441.6107	278087.7458
HM13	minor histocompatibility antigen H13 isoform 1 [Homo sapiens]	2760696.574	2360026.889	3691647.078	3364626.554	4899361.008	2432583.751	3022781.401	2453946.84	3910654.114
HMBS	porphobilinogen deaminase isoform 1 [Homo sapiens]	437093.4664	251952.0543	298044.8201	370210.8178	303501.9738	231417.2821	316583.1124	201187.9377	417039.467
HMG20A	high mobility group protein 20A [Homo sapiens]	414152.0364	325718.5601	528518.1954	137794.284	501940.178	522157.9083	476466.8791	463317.0498	429120.0629
HMGA1	high mobility group protein HMG-I/HMG-Y isoform a [Homo sapiens]	15177853.59	10713606.05	21763026.34	19488138.85	22020214.83	18132432	14269767.26	14543518.36	14646355.67
HMGA1	high mobility group protein HMG-I/HMG-Y isoform b [Homo sapiens]	15177853.59	10713606.05	21763026.34	19488138.85	22020214.83	18132432	14269767.26	14543518.36	14646355.67
HMGB1	high mobility group protein B1 [Homo sapiens]	13094396.46	12860274.23	17138407.61	13512970.35	19511139.6	14012948.62	11350380.13	9948010.26	13555119.37
HMGB2	high mobility group protein B2 [Homo sapiens]	3365199.767	3428127.8	4034354.993	3524116.106	5857118.188	3287993.506	3472542.819	2985700.822	1939315.275
HMGB3	high mobility group protein B3 [Homo sapiens]	3186854.24	3767018.465	4282874.004	2867724.371	3820726.298	3252199.837	3526424.928	2826644.424	3836423.724
HMGCL	hydroxymethylglutaryl-CoA lyase, mitochondrial isoform 1 precursor [Homo sa	272258.9269	201218.8597	447643.1651	388043.6132	168739.0071	370745.7819	348099.7028	69604.4375	220591.8479
HMGCS1	hydroxymethylglutaryl-CoA synthase, cytoplasmic [Homo sapiens]	1496474.468	1994773.665	1943079.691	1671412.973	1818287.284	1817905.08	1977517.363	1581856.792	2496943.237
HMGN1	non-histone chromosomal protein HMG-14 [Homo sapiens]	148375.0269	376933.5322	135279.4547	1307341.431	598102.5909	488508.9531	443212.5626	113183.7391	460023.3059
HMGN2	non-histone chromosomal protein HMG-17 [Homo sapiens]	98757.6172	82783.00864	161620.8744	73058.22377	129312.5446	115682.4442	106006.5463	103666.0904	0
HMGN5	high mobility group nucleosome-binding domain-containing protein 5 [Homo sa	610352.0963	713461.8415	848616.8107	816287.5642	838731.7	833624.8805	705896.595	733439.3289	276426.3003
HMHA1	minor histocompatibility protein HA-1 isoform 1 [Homo sapiens]	54167.25303	60827.34414	82292.64407	0	108283.5245	58203.24799	46526.69542	58171.46709	55254.37005
HMOX1	heme oxygenase 1 [Homo sapiens]	598270.2344	749774.9306	683671.9815	949851.5068	630553.631	695072.951	850935.4216	491186.0623	493070.4962
HMOX2	heme oxygenase 2 [Homo sapiens]	1481458.371	1912168.817	2126755.2	1909308.644	2040217.395	1921928.253	1851574.64	1529955.542	1519023.56
HN1	hematological and neurological expressed 1 protein isoform 1 [Homo sapiens]	19904967.86	23906858.68	21304880.61	23883121.71	28572230.95	26154156.03	15715670.06	22218370.68	24301964.95
HN1L	hematological and neurological expressed 1-like protein [Homo sapiens]	3051787.484	4176786.293	4222521.486	3521490.371	3276774.027	3690173.393	3429413.64	3289608.584	4428697.428
HNMT	histamine N-methyltransferase isoform 1 [Homo sapiens]	424680.4618	412018.4434	538597.2342	419673.9187	522335.9835	451028.5165	383801.2941	347166.7977	196515.5047
HNRNPA0	heterogeneous nuclear ribonucleoprotein A0 [Homo sapiens]	11454703.94	13973137.64	9791735.877	11244000.16	9304133.489	12397079.75	12336594.35	13514842.06	8150935.39
HNRNPA1	heterogeneous nuclear ribonucleoprotein A1 isoform a [Homo sapiens]	49563456.75	54361363.72	62043922.49	0	2911801.489	2558606.257	56412001.7	46868805.47	59344683.25
HNRNPA2B1	heterogeneous nuclear ribonucleoproteins A2/B1 isoform B1 [Homo sapiens]	86051157.11	79672243.79	108599665.2	90367302.54	107290727.7	95560735.78	84867448.04	71915766.81	83791137.33
HNRNPA3	heterogeneous nuclear ribonucleoprotein A3 [Homo sapiens]	20958695.3	20736885.67	27843646.38	22429693.43	23771451.71	23290737.11	21760006.81	16339235.12	21935341.32
HNRNPAB	heterogeneous nuclear ribonucleoprotein A/B isoform b [Homo sapiens]	14000372.56	0	0	17578538.15	20287609.54	18634932.68	0	15637379.9	18696154.87
HNRNPC	heterogeneous nuclear ribonucleoproteins C1/C2 isoform b [Homo sapiens]	12063677.55	15275027.43	16180967.55	14525250.69	11687820.5	17761861.67	14738573.25	11646362.22	15018530.39
HNRNPC	heterogeneous nuclear ribonucleoproteins C1/C2 isoform a [Homo sapiens]	12063677.55	15275027.43	16180967.55	14525250.69	11687820.5	17761861.67	14738573.25	11646362.22	15018530.39
HNRNPD	heterogeneous nuclear ribonucleoprotein D0 isoform a [Homo sapiens]	19967350.96	22810521.79	24807159.29	21804025.48	27042582.64	0	22283736.8	18685918.12	25056233.62
HNRNPF	heterogeneous nuclear ribonucleoprotein F [Homo sapiens]	9937192.917	11783179.73	13728485.55	11677627.47	13891988.78	11434506.27	11741488.75	10577543.36	8849680.447
HNRNPH1	heterogeneous nuclear ribonucleoprotein H [Homo sapiens]	14761634.21	16420783.3	17085213.72	17807174.39	16606264.86	18189058.11	17546409.86	14682863.28	17749586.74
HNRNPH2	heterogeneous nuclear ribonucleoprotein H2 [Homo sapiens]	4713624.788	3312574.597	3305250.224	3154835.504	3126210.197	3238487.427	3053476.754	3933281.235	5015619.638
HNRNPH3	heterogeneous nuclear ribonucleoprotein H3 isoform a [Homo sapiens]	115344.4426	4770952.73	5430176.115	139236.6644	5636216.845	179576.1689	388477.7345	3801449.891	432146.4961
HNRNPH3	heterogeneous nuclear ribonucleoprotein H3 isoform b [Homo sapiens]	115344.4426	4770952.73	5430176.115	139236.6644	5636216.845	179576.1689	388477.7345	3801449.891	432146.4961
HNRNPK	heterogeneous nuclear ribonucleoprotein K isoform a [Homo sapiens]	40696630.78	45573218.89	54724896.44	47424953.04	54377122.91	49983888.07	43773450.85	38575437.82	41745110.92
HNRNPL	heterogeneous nuclear ribonucleoprotein L isoform a [Homo sapiens]	8102283.311	9842707.189	10321259.02	8928688.469	10588639.82	8893409.107	9346976.944	7137641.98	9531624.847
HNRNPM	heterogeneous nuclear ribonucleoprotein M isoform a [Homo sapiens]	10233479.06	10598903.99	11209531.36	10313757.07	12623329.53	10581455.82	10365033.38	8860477.731	9946437.532
HNRNPR	heterogeneous nuclear ribonucleoprotein R isoform 2 [Homo sapiens]	1869905.242	2018229.341	2656492.991	2320837.42	2743717.1	2447002.751	1983644.094	1864580.737	2110658
HNRNPR	heterogeneous nuclear ribonucleoprotein R isoform 1 [Homo sapiens]	1869905.242	2018229.341	2656492.991	2320837.42	2743717.1	2447002.751	1983644.094	1864580.737	2110658
HNRNPU	heterogeneous nuclear ribonucleoprotein U isoform a [Homo sapiens]	14482338.35	15866134.22	18781512.18	16907265.16	8292.434484	17274742.48	17141622.12	14427216.9	17461773.84
HNRNPUL1	heterogeneous nuclear ribonucleoprotein U-like protein 1 isoform a [Homo sa	1010327.107	903765.9134	1074058.1	968511.1194	1199155.777	1204505.491	1122546.223	1012582.566	1230936.451
HNRNPUL2	heterogeneous nuclear ribonucleoprotein U-like protein 2 [Homo sapiens]	1672427.761	1929333.908	2381529.187	1967553.594	2290891.847	2044715.277	1899808.189	1616366.291	1863983.854
HNRPDL	heterogeneous nuclear ribonucleoprotein D-like isoform a [Homo sapiens]	10463379.04	12618547.28	13335091.54	11414763.04	13208967.98	11452677.93	10564708.41	9799808.088	12998457.07
HNRPLL	heterogeneous nuclear ribonucleoprotein L-like isoform 1 [Homo sapiens]	268976.3161	263864.292	384431.7314	310492.1888	420640.3635	376127.5681	242388.4975	214429.333	255866.6445
HOOK3	protein Hook homolog 3 [Homo sapiens]	34075.33908	40079.70795	49829.04281	55668.05199	45956.54716	40667.02006	30580.31575	28726.62252	153881.8465
HP1BP3	heterochromatin protein 1-binding protein 3 [Homo sapiens]	951874.0094	820664.8502	1159844.832	815911.6882	1204974.581	1016220.55	724766.8798	662567.9071	825716.1599
HPCAL1	hippocalcin-like protein 1 [Homo sapiens]	2736997.413	3887048.558	4158549.743	4180727.995	5150837.139	4492407.738	4231432.361	3642677.019	4018293.934

HPDL	4-hydroxyphenylpyruvate dioxygenase-like protein [Homo sapiens]	1705845.011	1924707.615	2139343.76	1908835.68	1998825.218	1931343.97	2123225.942	1692776.636	2038576.719
HPRT1	hypoxanthine-guanine phosphoribosyltransferase [Homo sapiens]	9253930.108	8766484.557	10709726.12	10294728.04	9732926.979	10986065.01	10418763.99	9171579.883	9078016.082
HPS3	Hermansky-Pudlak syndrome 3 protein [Homo sapiens]	59996.55249	54559.58644	36037.53588	69349.94442	36566.27218	90307.02039	66191.6718	45685.28315	61543.60981
HPS5	Hermansky-Pudlak syndrome 5 protein isoform a [Homo sapiens]	23783.51863	30483.95341	27602.82508	3695.972143	24683.15931	26683.69434	14450.14854	29499.78313	24762.21243
HPS6	Hermansky-Pudlak syndrome 6 protein [Homo sapiens]	87631.64198	167599.3681	125392.5187	103948.8247	121020.8663	104147.3529	105081.501	57530.35556	200814.5075
HRAS	GTPase HRas isoform 1 [Homo sapiens]	0	0	0	0	0	0	251599.6285	191019.5385	0
HRNR	hornerin [Homo sapiens]	118470.141	76265.47715	71062.29317	72263.92904	141978.0853	96202.20685	108371.5788	44997.91645	92586.4902
HRSP12	ribonuclease UK114 [Homo sapiens]	2801226.061	4034565.109	3913052.022	2848533.63	3143240.87	3500573.717	2878537.735	2457950.587	3259328.578
HS2ST1	heparan sulfate 2-O-sulfotransferase 1 isoform 1 [Homo sapiens]	150365.6291	2336775.898	144010.6009	398760.7979	184669.4383	183026.5471	278981.0931	247491.0662	155364.9073
HSBP1	heat shock factor-binding protein 1 [Homo sapiens]	6191380.641	6229339.178	8742647.348	5610280.019	11885661.82	7899549.274	6945101.252	6537307.144	7829500.444
HSD17B10	3-hydroxyacyl-CoA dehydrogenase type-2 isoform 1 [Homo sapiens]	14398856.78	18841388.08	19097811.32	15312295.06	18805670.29	16672293.14	17054186.06	14253585.36	19006564.91
HSD17B11	estradiol 17-beta-dehydrogenase 11 precursor [Homo sapiens]	80219.93415	0	146720.7024	157935.4137	167496.4295	215086.4018	159950.3341	128760.3884	136464.1674
HSD17B12	estradiol 17-beta-dehydrogenase 12 [Homo sapiens]	1019631.933	1220361.654	1333652.155	1195607.489	1516334.332	1326868.444	1318293.864	979866.1342	1404374.916
HSD17B4	peroxisomal multifunctional enzyme type 2 isoform 2 [Homo sapiens]	1508543.396	1777407.485	2110673.152	1791493.792	2307700.443	1892721.376	1761275.057	1499871.022	1868278.846
HSD17B7	3-keto-steroid reductase [Homo sapiens]	330852.2035	372511.6936	0	388773.4867	543657.9558	0	447528.6549	340374.354	0
HSD17B8	estradiol 17-beta-dehydrogenase 8 [Homo sapiens]	318949.4085	0	404666.4512	471210.9329	324348.5701	0	507189.7866	0	413742.1585
HSDL1	inactive hydroxysteroid dehydrogenase-like protein 1 isoform a [Homo sapier	212358.8368	148893.2521	93844.07192	113903.8938	105882.8761	294607.9667	0	0	0
HSDL2	hydroxysteroid dehydrogenase-like protein 2 isoform 1 [Homo sapiens]	1794395.464	1966065.279	2291029.821	1819358.191	2276124.19	2119109.109	2079977.693	1357677.374	1855326.561
HSF1	heat shock factor protein 1 [Homo sapiens]	176880.9513	218557.0647	184219.9276	153063.2309	257614.2189	261619.4814	235431.9663	187170.9499	146104.6247
HSP90AA1	heat shock protein HSP 90-alpha isoform 1 [Homo sapiens]	28349345.69	34595393.11	32907256.87	35803852.82	34869579.18	36913741.59	35542457.02	31207771.04	35024432.15
HSP90AB1	heat shock protein HSP 90-beta isoform a [Homo sapiens]	48960230.97	56329953.49	54560225.71	60985009.02	66486477.65	63168019.52	62687121.77	55322668.52	59447703.26
HSP90B1	endoplasmin precursor [Homo sapiens]	6414050.912	6919626.063	8412976.974	7814280.412	8254548.321	7734770.335	7463673.457	7058846.196	7944154.838
HSPA13	heat shock 70 kDa protein 13 precursor [Homo sapiens]	206311.4273	313716.2298	276926.4638	265489.0137	362713.2024	297998.5461	245351.1563	569830.507	242656.8509
HSPA14	heat shock 70 kDa protein 14 isoform 1 [Homo sapiens]	717909.1803	837868.3441	925114.4032	815726.6926	981671.8101	513912.9397	818021.5569	733778.3771	477360.6795
HSPA1B	heat shock 70 kDa protein 1A/1B [Homo sapiens]	21368003.52	27372858.4	30937291.28	28025645.27	34635673.18	27275513.5	28604396.56	24493576.04	30555500.89
HSPA4	heat shock 70 kDa protein 4 [Homo sapiens]	3403386.312	3641915.365	4524278.787	4175853.898	4309563.84	4044990.064	4139782.506	3506064.378	3940900.017
HSPA4L	heat shock 70 kDa protein 4L [Homo sapiens]	647050.7043	743963.3657	786154.2292	795170.7239	824616.5193	799935.618	870641.3968	583790.4595	830337.2718
HSPA5	78 kDa glucose-regulated protein precursor [Homo sapiens]	17421289.64	20600803.84	22633079.39	18994028.53	23258582.73	19475318.77	21888407.66	16673017.8	22495234.71
HSPA8	heat shock cognate 71 kDa protein isoform 1 [Homo sapiens]	55957752.22	65740163.62	68147984.4	63841808.61	71938558.16	66577148.3	69728129.86	60109458.3	71756531.52
HSPA9	stress-70 protein, mitochondrial precursor [Homo sapiens]	10418329.76	11649938.3	12833478.07	12056360.6	13419818.07	12126079.17	12868986	10932514.22	12387816.16
HSPB1	heat shock protein beta-1 [Homo sapiens]	26072305.18	32974684.23	35569378.97	25212393.3	34248377.18	34303451.62	30057607.66	26235750.94	30209720.35
HSPB11	heat shock protein beta-11 [Homo sapiens]	3233172.089	4820995.328	4866232.186	4799453.486	5056251.167	4943570.958	4737220.497	3679086.725	4703143.761
HSPB8	heat shock protein beta-8 [Homo sapiens]	1529208.142	1262127.486	1523830.885	1718884.685	1358809.256	1222875.771	1423670.627	1470458.894	1049581.117
HSPBP1	hsp70-binding protein 1 [Homo sapiens]	1233154.415	1294407.342	1563876.881	1022468.233	1541298.732	1375407.527	1412397.741	1148772.007	1435389.867
HSPD1	60 kDa heat shock protein, mitochondrial [Homo sapiens]	45990445.5	49565087.76	56501088.7	50634861.63	57309559.68	51504926.11	50204109.41	44140034.85	50853685.62
HSPE1	10 kDa heat shock protein, mitochondrial [Homo sapiens]	78528320	85525991.35	107508052.1	55326209.91	95921009.2	59109688.98	91641943.54	79411539.39	105581314.5
HSPE1-MOB4	HSPE1-MOB4 protein [Homo sapiens]	0	0	0	0	0	0	0	0	0
HSPH1	heat shock protein 105 kDa [Homo sapiens]	2360165.906	2531307.496	3041982.238	2160968.537	2722934.698	2655198.463	2120789.662	2199268.245	2894742.856
HTATIP2	oxidoreductase HTATIP2 isoform a precursor [Homo sapiens]	1580486.972	1892182.544	2012667.647	1872848.798	1991024.891	1896084.502	1500566.817	1222727.828	2011989.535
HTATSF1	HIV Tat-specific factor 1 [Homo sapiens]	616075.2259	490883.3667	439365.4417	453692.9546	587374.163	468178.4836	342783.188	276068.2303	525350.4852
HTR3D	5-hydroxytryptamine receptor 3D isoform 2 [Homo sapiens]	0	0	0	0	0	0	0	0	0
HTRA2	serine protease HTRA2, mitochondrial isoform 1 preproprotein [Homo sapiens]	441563.3012	294902.7479	501558.7035	420138.394	532597.6332	484759.0437	509747.8672	355098.2733	419735.8436
HTT	huntingtin [Homo sapiens]	26249.03011	41121.74577	52715.27358	40408.05395	39710.7424	47134.77876	35181.16899	38049.85078	32872.16045
HUS1	checkpoint protein HUS1 [Homo sapiens]	359138.8418	444460.4588	948255.0421	403057.3563	472294.8155	405807.7258	396393.3062	186390.0838	435167.2631
HUWE1	E3 ubiquitin-protein ligase HUWE1 [Homo sapiens]	277689.0136	249574.4268	322568.9568	264384.7723	342193.8915	334466.7125	284816.1874	199594.4874	261658.9517
HYI	putative hydroxypyruvate isomerase isoform 3 [Homo sapiens]	0	0	0	0	0	0	0	0	0
HYOU1	hypoxia up-regulated protein 1 precursor [Homo sapiens]	1216948.343	1506461.196	1539461.154	1418464.841	1594608.307	1469042.922	1547831.799	1312703.096	1544762.338
HYPK	huntingtin-interacting protein K isoform 1 [Homo sapiens]	5266412.54	6107780.984	7213520.377	5982316.061	7072208.858	6622932.382	5702781.102	5118883.03	5334291.351
IAH1	isoamyl acetate-hydrolyzing esterase 1 homolog precursor [Homo sapiens]	771644.3908	755990.5704	1090625.695	690517.0801	1137091.256	680371.4716	914295.1023	685410.816	1251903.354
IARS	isoleucine--tRNA ligase, cytoplasmic [Homo sapiens]	980008.818	1100452.144	1339019.783	1076671.169	1411696.558	1201159.459	1128675.356	886096.1423	1086647.826
IARS2	isoleucine--tRNA ligase, mitochondrial precursor [Homo sapiens]	488149.0616	507015.1608	641582.9493	520486.0368	630303.6405	558563.3965	545844.0427	436170.0141	515089.1568
IBA57	putative transferase CAF17, mitochondrial precursor [Homo sapiens]	148144.8586	148695.5451	255293.7049	245266.1967	169057.4986	214716.1168	234556.4119	141576.4098	212330.6988
ICAM1	intercellular adhesion molecule 1 precursor [Homo sapiens]	518908.6751	685745.8136	611901.6537	643111.6904	606480.0186	875999.0893	604484.3435	501937.7841	795173.3237
ICAM2	intercellular adhesion molecule 2 precursor [Homo sapiens]	464521.3417	437924.266	528834.3038	549648.7	491978.2876	560384.1042	503396.9264	369584.1311	463025.7498
ICMT	protein-S-isoprenylcysteine O-methyltransferase [Homo sapiens]	259396.7803	323078.9615	108750.2979	393712.0052	322890.6164	676629.9949	522926.7492	308862.3767	523985.6907
ICT1	peptidyl-tRNA hydrolase ICT1, mitochondrial precursor [Homo sapiens]	658974.5609	646769.1985	0	455989.477	686258.6348	242046.3962	551186.5605	261257.2022	342438.8415
ID1	DNA-binding protein inhibitor ID-1 isoform a [Homo sapiens]	221804.6179	356389.5857	0	208158.6628	250051.4287	343201.1191	293708.8232	237943.9257	281911.1049
IDE	insulin-degrading enzyme isoform 1 [Homo sapiens]	670459.8901	746841.5899	704434.5281	736658.8573	719143.5148	737936.3978	572169.2538	629619.5933	754843.9662
IDH1	isocitrate dehydrogenase [NADP] cytoplasmic [Homo sapiens]	5577891.955	5554523.991	7454256.322	5986925.253	7709568.569	6111072.345	5399422.258	4456167.238	5980641.5
IDH2	isocitrate dehydrogenase [NADP], mitochondrial precursor [Homo sapiens]	931201.5399	906620.7495	1260559.591	926270.0682	1268375.53	988082.4631	1038915.542	860331.2354	1023609.112
IDH3A	isocitrate dehydrogenase [NAD] subunit alpha, mitochondrial precursor [Homo	3178499.604	3444546.197	4440883.965	4105313.178	4220046.509	3623599.782	4348320.414	3628652.505	4581713.005
IDH3B	isocitrate dehydrogenase [NAD] subunit beta, mitochondrial isoform a precu	1186149.015	921188.1197	1812556.981	1301595.981	1775576.18	1281473.896	1332206.665	947812.6155	1520621.768
IDH3G	isocitrate dehydrogenase [NAD] subunit gamma, mitochondrial isoform a precu	1166052.835	1220509.316	1640436.213	1489358.624	1634443.146	1561052.821	1157095.296	1393031.18	1656641.757
IDI1	isopentenyl-diphosphate Delta-isomerase 1 [Homo sapiens]	2349379.998	2189120.961	2877989.839	2475397.598	2934216.775	2692259.747	2655191.812	2064229.111	2681444.169
IER3IP1	immediate early response 3-interacting protein 1 precursor [Homo sapiens]	11864607.3	14321045.24	13655049.85	12806279.85	15117735.9	12894071.57	13069174.78	11560253.92	15422489.2
IFI16	gamma-interferon-inducible protein 16 isoform 2 [Homo sapiens]	1157973.982	1261931.334	1473889.609	1246650.804	1567203.443	1207573.098	1108734.068	869522.4043	1160956.67
IFI27L2	interferon alpha-inducible protein 27-like protein 2 precursor [Homo sapier	730770.0158	675285.5104	1313905.693	745367.4167	0	935194.5575	267227.3594	0	518963.1354
IFI35	interferon-induced 35 kDa protein [Homo sapiens]	854204.3938	1290713.17	552096.1739	631903.6355	1145508.491	869864.1475	421482.7038	649499.9419	786615.9958
IFIH1	interferon-induced helicase C domain-containing protein 1 [Homo sapiens]	0	0	0	0	0	0	0	12643.35756	17964.74381
IFIT1	interferon-induced protein with tetratricopeptide repeats 1 isoform 1 [Homo	513193.8027	534388.0149	587178.1765	493274.4882	625760.6611	557544.8059	594526.6914	426231.0506	581415.2362
IFIT2	interferon-induced protein with tetratricopeptide repeats 2 [Homo sapiens]	145647.5069	132535.8591	353232.0398	241777.5983	243318.914	356149.1966	284277.2792	249242.0257	383249.8896
IFIT3	interferon-induced protein with tetratricopeptide repeats 3 [Homo sapiens]	472122.0037	384572.2563	646955.5314	581735.2919	699805.953	598434.7897	429134.9423	551405.3	640929.8519
IFITM1	interferon-induced transmembrane protein 1 [Homo sapiens]	2498987.579	0	3355437.996	3301200.704	3355263.463	3646813.667	3360334.194	2862961.389	0
IFITM2	interferon-induced transmembrane protein 2 [Homo sapiens]	0	0	0	0	0	0	1103664.979	0	5831692.604
IFITM3	interferon-induced transmembrane protein 3 [Homo sapiens]	27135112.5	26877329.01	31619858.39	21914309.66	30104571.16	30069039.04	17614601.49	20368711.35	22755015.14
IFRD1	interferon-related developmental regulator 1 isoform 1 [Homo sapiens]	0	85684.84468	21991.70087	93661.89526	0	89639.66185	93852.60838	74710.29988	121532.7457
IFRD2	interferon-related developmental regulator 2 [Homo sapiens]	354244.8433	341569.8887	405491.6392	312596.7914	465444.2502	400332.873	321116.5973	213733.9331	254817.4932
IFT27	intraflagellar transport protein 27 homolog isoform 1 [Homo sapiens]	0	0	0	0	0	0	389507.8575	0	660137.0211
IGBP1	immunoglobulin-binding protein 1 [Homo sapiens]	661299.7488	1010784.308	694551.1267	845553.6986	1015727.014	879838.2863	779857.5616	699034.3808	1020775.081
IGF1R	insulin-like growth factor 1 receptor precursor [Homo sapiens]	0	0	0	0	0	0	53898.81786	33199.42415	29083.5988
IGF2BP1	insulin-like growth factor 2 mRNA-binding protein 1 isoform 1 [Homo sapiens	1329199.979	1505945.722	1984479.821	1603617.927	1963963.71	1757008.832	1652933.273	1398750.391	1600652.641
IGF2BP2	insulin-like growth factor 2 mRNA-binding protein 2 isoform a [Homo sapiens	0	938336.3705	771517.9907	1035637.693	772351.6732	991288.022	904294.0084	1702088.304	1108330.991
IGF2BP3	insulin-like growth factor 2 mRNA-binding protein 3 [Homo sapiens]	1482684.161	1528263.073	1962587.259	1497008.352	1962555.475	1524180.408	1488066.984	1417581.692	1579712.68
IGF2R	cation-independent mannose-6-phosphate receptor precursor [Homo sapiens]	63966.09314	96268.4874	113528.0797	74902.50463	94645.53694	102503.43	139413.8693	148049.6908	79863.85367
IGHMBP2	DNA-binding protein SMUBP-2 [Homo sapiens]	0	0	0	0	0	0	16650.78435	17298.64437	38032.77489
IK	protein Red [Homo sapiens]	405849.3489	356032.6913	314614.1988	208685.334	567793.9996	413028.8032	216945.3276	200468.2623	362502.6878
IKBIP	inhibitor of nuclear factor kappa-B kinase-interacting protein isoform 1 [	687814.632	863749.5802	803012.101	825246.3795	855574.8037	819226.9924	807014.2661	660062.6016	949758.2384
IKBIP	inhibitor of nuclear factor kappa-B kinase-interacting protein isoform 2 [	687814.632	863749.5802	803012.101	825246.3795	855574.8037	819226.9924	807014.2661	660062.6016	949758.2384
IKBKAP	elongator complex protein 1 [Homo sapiens]	281735.4758	321248.5364	353482.5032	298727.5578	365920.7484	366231.5908	340251.2627	282339.9343	334549.0207
IKBKB	inhibitor of nuclear factor kappa-B kinase subunit beta isoform 1 [Homo sa	0	0	0	0	0	0	67615.40878	0	41419.91639
IKBKG	NF-kappa-B essential modulator isoform b [Homo sapiens]	146884.7978	410711.7798	160603.0246	167187.8092	120734.44	197900.4427	220342.3941	216619.5884	133425.8447
IL18	interleukin-18 isoform 1 proprotein [Homo sapiens]	2410699.411	2894477.725	3487562.657	2648256.898	3261813.428	3319117.495	2911186.477	2212677.536	3264676.883
ILF2	interleukin enhancer-binding factor 2 isoform 1 [Homo sapiens]	11156724.3	12454992.43	15135691.66	11867017.41	16045764.74	12889560.04	11985096.21	10285767.56	13004982.41
ILF3	interleukin enhancer-binding factor 3 isoform d [Homo sapiens]	0	0	0	0	0	0	0	3502348.29	4074863.923
ILK	integrin-linked protein kinase isoform 1 [Homo sapiens]	699745.2081	788717.3575	975737.4398	846979.6968	1083559.533	931547.6898	781766.6435	667636.8785	1097683.787
ILKAP	integrin-linked kinase-associated serine/threonine phosphatase 2C [Homo sa	316836.029	287457.7288	189615.8996	458235.7117	400710.7615	336961.789	44991.00829	105058.4807	383483.656
ILVBL	acetolactate synthase-like protein [Homo sapiens]	595859.5184	647715.4413	698076.6447	635260.8131	730310.8704	685228.0214	650687.6063	506866.2961	665614.1442
IMMT	mitochondrial inner membrane protein isoform 1 [Homo sapiens]	106238.3169	98951.85925	32802.37039	58868.27075	356361.1546	151260.1627	115113.5	671345.228	0
IMMT	mitochondrial inner membrane protein isoform 3 [Homo sapiens]	106238.3169	98951.85925	32802.37039	58868.27075	356361.1546	151260.1627	115113.5	671345.228	0
IMP3	U3 small nucleolar ribonucleoprotein protein IMP3 [Homo sapiens]	1258536.945	1576826.711	1523200.597	6470125.478	1826908.68	1130109.705	1018086.348	1286153.811	1259571.465
IMP4	U3 small nucleolar ribonucleoprotein protein IMP4 [Homo sapiens]	476708.1251	154098.078	543222.9416	283574.1022	685524.2926	241106.759	445145.5837	422416.3903	569025.5747
IMPA1	inositol monophosphatase 1 isoform 2 [Homo sapiens]	805367.965	0	1067321.615	0	990992.4785	936552.9175	684154.4435	0	714368.57
IMPA1	inositol monophosphatase 1 isoform 1 [Homo sapiens]	805367.965	0	1067321.615	0	990992.4785	936552.9175	684154.4435	0	714368.57
IMPA2	inositol monophosphatase 2 [Homo sapiens]	590322.7039	1324258.45	1535817.759	1320044.669	1509491.984	1430635.64	1147659.691	1042442.319	1363411.155
IMPAD1	inositol monophosphatase 3 [Homo sapiens]	1104049.275	1222482.128	1475689.517	1268415.525	1446698.29	1285355.082	1214853.612	1029982.812	1404761.578
IMPDH1	inosine-5'-monophosphate dehydrogenase 1 isoform a [Homo sapiens]	1104262.679	1330548.945	1261995.508	1331501.671	1198672.097	1513267.502	1414571.078	1236352.416	1139825.727
IMPDH2	inosine-5'-monophosphate dehydrogenase 2 [Homo sapiens]	9651661.382	12042568.66	12034564.39	11840223.28	13204537.73	12373976.63	12132483.18	10477770.04	11915542.23
INA	alpha-internexin [Homo sapiens]	215460.6427	286460.7678	258925.2288	255415.0847	312987.5883	231701.6328	314190.9684	210276.637	294551.6944
INCENP	inner centromere protein isoform 1 [Homo sapiens]	99500.86854	60780.60043	90801.87865	48576.50843	137381.7896	78483.58539	14917.325	72868.11811	0
INF2	inverted formin-2 isoform 1 [Homo sapiens]	458638.6195	438209.0756	579062.9216	447395.9225	597594.2864	554040.8995	513932.4063	417067.389	409447.4373
INO80B	INO80 complex subunit B [Homo sapiens]	68990.97139	62319.92599	221609.3442	92617.74683	167212.6655	93541.15472	69566.01782	46448.28389	35589.45246
INPP1	inositol polyphosphate 1-phosphatase [Homo sapiens]	67957.24046	302154.5344	162573.5305	156547.1162	214906.4943	164704.25	154444.8821	109823.4708	0
INPP5K	inositol polyphosphate 5-phosphatase K isoform 1 [Homo sapiens]	0	0	0	0	0	0	0	60119.6831	54088.69587
INPPL1	phosphatidylinositol 3,4,5-trisphosphate 5-phosphatase 2 [Homo sapiens]	248556.1466	4562234.246	323864.7706	251853.3513	295476.6957	282798.6445	251642.828	213261.3101	256349.4913
INTS1	integrator complex subunit 1 [Homo sapiens]	15246.62651	16150.77205	18799.70777	26767.47731	28111.30141	35049.23733	21674.74089	10978.77833	16068.26967
INTS12	integrator complex subunit 12 [Homo sapiens]	109382.0777	0	0	134782.311	115382.3763	123103.7442	0	0	0
INTS2	integrator complex subunit 2 [Homo sapiens]	27426.58164	21179.03481	37213.72303	21277.72094	61603.75961	31305.04388	45553.93447	0	25325.83992
INTS3	integrator complex subunit 3 [Homo sapiens]	332952.7595	270474.7292	381117.067	322225.2068	399809.9832	336605.4873	267008.662	150413.7375	370331.5289
INTS4	integrator complex subunit 4 [Homo sapiens]	51603.97423	71204.60693	73445.60025	67130.77561	76666.61086	73558.14599	43723.39008	37951.62707	61693.2197
INTS5	integrator complex subunit 5 [Homo sapiens]	36505.03149	31244.9635	61057.63382	38242.30821	65805.48187	32899.68321	35111.60115	0	76766.66229

INTS6	integrator complex subunit 6 isoform a [Homo sapiens]	52388.94371	11382.01448	80411.55829	21154.51773	81242.57677	72442.13553	208223.2739	37828.15256	36567.78365
INTS7	integrator complex subunit 7 isoform 1 [Homo sapiens]	111373.5907	453891.1706	120119.7113	98378.49625	111369.4119	111389.9065	103766.2677	165343.7599	112045.451
INTS9	integrator complex subunit 9 isoform 1 [Homo sapiens]	187166.0026	126789.68077	110238.9115	92258.33913	147961.9679	135982.1321	0	0	0
IPO11	importin-11 isoform 2 [Homo sapiens]	304826.9464	350438.869	406283.8361	364367.5384	521024.2626	372772.8361	364350.6439	286128.6664	306205.0453
IPO13	importin-13 [Homo sapiens]	21187.09835	20196.25533	19731.32655	50385.14779	22165.08963	35069.21064	57280.56612	0	175186.579
IPO4	importin-4 [Homo sapiens]	805403.6683	901094.6094	1473235.305	859103.9583	1352416.824	2323834.442	871293.0596	762298.6185	1020407.537
IPO5	importin-5 [Homo sapiens]	3228678.535	3609350.193	4132934.523	3563189.947	4058638.637	4152440.661	3712216.015	3230075.24	3530517.833
IPO7	importin-7 [Homo sapiens]	3705187.955	3470887.777	4819012.508	3823253.22	4736307.699	3911028.654	3760328.482	3362643.676	4084965.32
IPO8	importin-8 isoform 1 [Homo sapiens]	152464.1361	155465.8019	213577.3552	125350.4705	246131.101	202288.7767	160069.7773	137310.9341	274128.502
IPO9	importin-9 [Homo sapiens]	1171590.67	1312545.339	1613086.733	1302151.94	1704350.379	1520935.92	1302368.442	1137283.63	1126529.331
IQGAP1	ras GTPase-activating-like protein IQGAP1 [Homo sapiens]	2159763.962	2470485.079	2981283.715	2491180.893	3160015.849	2637284.788	2401682.169	2033588.356	2529655.489
IQGAP3	ras GTPase-activating-like protein IQGAP3 [Homo sapiens]	30219.50249	26471.51629	41556.0067	22512.85983	32737.33961	17255.40423	29972.64274	12854.70189	0
IRAK1	interleukin-1 receptor-associated kinase 1 isoform 1 [Homo sapiens]	111901.0224	55928.53901	95016.81557	77803.62981	85813.92472	57316.16821	63304.29693	23550.0534	69411.46788
IREB2	iron-responsive element-binding protein 2 [Homo sapiens]	65192.32428	84103.37305	128914.9321	121984.5096	107089.2063	137627.0165	113762.916	98714.7147	172927.7513
IRF2BP1	interferon regulatory factor 2-binding protein 1 [Homo sapiens]	128448.1137	150024.9457	184543.9215	144373.9705	381053.2744	275136.1214	251942.9378	171405.6111	254760.5762
IRF2BP2	interferon regulatory factor 2-binding protein 2 isoform A [Homo sapiens]	527708.088	650448.4195	714835.191	498508.7284	743446.4585	609347.052	234148.8027	411440.6743	447144.7976
IRF2BPL	interferon regulatory factor 2-binding protein-like [Homo sapiens]	88717.00405	0	123470.5789	86508.13158	159170.6351	116904.2227	125656.0294	111305.4575	122141.2024
IRF3	interferon regulatory factor 3 isoform 1 [Homo sapiens]	327228.7217	0	467492.8895	407692.8715	725315.5936	494290.5052	465530.7296	829509.7068	458984.4436
IRGQ	immunity-related GTPase family Q protein [Homo sapiens]	316478.7896	300925.666	410555.2278	371354.219	388949.6753	345950.4223	349852.9981	312285.5098	350080.7271
IRS2	insulin receptor substrate 2 [Homo sapiens]	19058.33716	12724.01292	19789.72187	20901.83385	51978.83424	27764.86623	9629.070045	0	55179.07299
ISCA1	iron-sulfur cluster assembly 1 homolog, mitochondrial precursor [Homo sapie	533401.6861	534401.3484	799939.3703	474661.25	462674.2951	648551.918	570854.3207	591840.3985	321033.4207
ISCA2	iron-sulfur cluster assembly 2 homolog, mitochondrial isoform 1 precursor [	111373.1409	229947.3307	219973.8398	0	1092152.875	248810.2478	184090.7273	0	197106.5227
ISCU	iron-sulfur cluster assembly enzyme ISCU, mitochondrial isoform ISCU2 precu	676541.3315	794596.2152	805318.8498	748545.4455	565005.978	639889.7179	713129.9038	719752.2115	700366.0249
ISG15	ubiquitin-like protein ISG15 precursor [Homo sapiens]	32676590.15	35705121.4	43619984.18	36978780.67	45830575.18	38603069.42	39835353.38	34241205.35	39986903.93
ISG20L2	interferon-stimulated 20 kDa exonuclease-like 2 [Homo sapiens]	155890.798	137835.0538	371482.4746	133503.9134	243783.8694	291227.2825	125677.0639	133548.8558	183382.6045
ISOC1	isochorismatase domain-containing protein 1 [Homo sapiens]	1278165.958	847365.9857	1278286.784	1301873.763	1575302.988	1322275.34	603789.6108	1197653.465	1096243.843
ISOC2	isochorismatase domain-containing protein 2, mitochondrial isoform 2 [Homo	1128756.724	965086.1581	1530479.919	1243688.904	1722501.178	1602865.975	999379.2304	1168610.514	1654335.688
IST1	IST1 homolog isoform c [Homo sapiens]	905346.6669	866010.703	1084870.935	997719.4985	1351741.912	1016572.506	951562.0564	828571.8143	779323.6158
ISY1-RAB43	ISY1-RAB43 protein [Homo sapiens]	279578.5817	386210.6612	336820.0089	399387.1647	1107866.268	422782.9928	354552.9218	318993.8905	358504.8925
ITCH	E3 ubiquitin-protein ligase Itchy homolog isoform 1 [Homo sapiens]	79010.58155	0	0	68975.39041	152471.179	90302.6317	77751.19979	34280.80657	103529.8719
ITFG3	protein ITFG3 [Homo sapiens]	0	0	0	0	0	0	67868.36025	0	99515.90006
ITGA1	integrin alpha-1 precursor [Homo sapiens]	56045.85139	49694.63658	56665.41517	63372.93586	64583.51231	67742.33409	103670.9923	64991.43188	46670.16095
ITGA2	integrin alpha-2 precursor [Homo sapiens]	103340.9462	394201.3636	161499.0016	173391.4529	345068.2438	148398.5361	166202.9113	109662.0413	508692.1204
ITGA3	integrin alpha-3 isoform b precursor [Homo sapiens]	316632.1801	222539.5062	421770.3976	351734.0381	410489.1259	387859.8943	397048.3482	225570.7423	257646.9157
ITGA5	integrin alpha-5 precursor [Homo sapiens]	354277.4605	478676.8195	461722.1867	388120.8769	503282.6697	428499.3333	463850.2667	360982.4524	431187.3367
ITGA6	integrin alpha-6 isoform a precursor [Homo sapiens]	58463.32348	70042.60847	85872.48406	310157.4195	86936.8751	77154.85886	77578.81594	67157.20079	94636.93949
ITGAV	integrin alpha-V isoform 1 precursor [Homo sapiens]	269525.6664	332108.1738	389368.9214	303142.4092	362556.4092	353149.3997	378988.422	279290.207	333945.878
ITGB1	integrin beta-1 isoform 1A precursor [Homo sapiens]	2279040.341	0	3504664.095	2955715.284	3242415.618	2688138.403	3155759.527	2584748.552	2351237.069
ITGB4	integrin beta-4 isoform 1 precursor [Homo sapiens]	113584.6156	45879.22236	49964.23384	110511.3507	73782.54135	55693.08912	55032.45513	73937.42829	28032.15093
ITGB5	integrin beta-5 precursor [Homo sapiens]	98891.74007	172709.7726	67638.61949	86883.66563	95315.20758	93130.10484	186686.0719	85781.59567	148155.5096
ITM2B	integral membrane protein 2B [Homo sapiens]	0	160387.8038	145228.9755	248301.4839	236085.747	177356.1109	0	0	0
ITPA	inosine triphosphate pyrophosphatase isoform a [Homo sapiens]	2090051.032	3352493.472	983392.9514	2728047.604	1362526.228	2942926.913	2056292.238	1790126.635	2091307.519
ITPK1	inositol-tetrakisphosphate 1-kinase isoform a [Homo sapiens]	441459.7261	622851.5	929024.2279	0	774677.2941	697999.1544	605146.4007	0	513850.7868
ITPR1	inositol 1,4,5-trisphosphate receptor type 1 isoform 3 [Homo sapiens]	26069.56421	55728.26489	35638.61509	54181.0291	47558.23354	53923.89689	35171.14025	25770.28293	45103.37493
ITPR3	inositol 1,4,5-trisphosphate receptor type 3 [Homo sapiens]	89208.53075	432399.5298	111433.7234	77016.25318	110485.1292	93751.60007	90189.08639	67072.00159	101992.4952
ITPRIP	inositol 1,4,5-trisphosphate receptor-interacting protein precursor [Homo s	89665.19355	1247702.808	0	110189.3861	106013.5838	123814.4461	159698.641	124906.5366	0
ITSN1	intersectin-1 isoform ITSN-1 [Homo sapiens]	0	0	13703.15664	29061.91339	23508.94819	46446.57694	32715.7583	18396.01729	12487.77248
ITSN2	intersectin-2 isoform 1 [Homo sapiens]	15453.7346	13315.94022	0	4878.942183	11145.90508	12693.199	14469.85934	0	8868.52301
IVD	isovaleryl-CoA dehydrogenase, mitochondrial isoform 1 precursor [Homo sapie	276488.8344	432928.8963	346103.504	245291.8558	446901.1271	435649.7035	514842.476	3486279.019	3606970.471
IVNS1ABP	influenza virus NS1A-binding protein [Homo sapiens]	185855.9661	470935.3087	343906.7662	126909.0402	360526.807	372461.1925	127711.3733	292689.4665	429781.055
IWS1	protein IWS1 homolog [Homo sapiens]	254960.6448	274213.2568	251458.3725	302509.3721	226414.6737	292045.5596	301693.5604	245070.2057	347209.1499
JAGN1	protein jagunal homolog 1 [Homo sapiens]	2188675.088	2825417.891	0	2925111.016	6108941.259	2981308.743	2933049.609	0	3101604.132
JAK1	tyrosine-protein kinase JAK1 [Homo sapiens]	17222.60832	10024.65351	18577.76917	19937.04616	21628.55275	19852.94111	17257.36965	11945.95257	9949.121016
JMJD1C	probable JmjC domain-containing histone demethylation protein 2C isoform a	3743.506764	11470.24795	7137.024279	0	4889.45611	7514.762014	0	0	0
JMJD6	bifunctional arginine demethylase and lysyl-hydroxylase JMJD6 isoform 1 [Ho	741480.4012	615076.5626	958303.3491	852488.1621	977025.9565	889823.4863	878746.4354	742703.4615	906877.7752
JTB	protein JTB precursor [Homo sapiens]	1174264.25	1152860.214	1450699.187	1014688.851	1546650.409	1108427.282	1020576.155	90875.40402	1215267.221
JUN	transcription factor AP-1 [Homo sapiens]	0	0	0	0	0	0	0	0	0
JUNB	transcription factor jun-B [Homo sapiens]	447728.1663	191178.5838	438187.2767	464174.2496	565652.5857	524334.4328	255687.3649	286798.3803	491213.3495
JUND	transcription factor jun-D [Homo sapiens]	216061.9838	329662.9369	294975.8133	374291.559	234010.5	377435.4874	0	273447.7809	473973.6592
JUP	junction plakoglobin [Homo sapiens]	1039221.194	1034278.947	1261168.898	1078898.728	1390751.399	1374686.47	934426.49	1040448.792	1075106.776
KANK1	KN motif and ankyrin repeat domain-containing protein 1 isoform a [Homo sap	0	14532.09525	24489.35086	29657.34457	25836.68461	26797.01099	17248.68115	23560.09079	17339.14632
KANK2	KN motif and ankyrin repeat domain-containing protein 2 isoform 2 [Homo sap	19376.78325	57467.00953	92329.63869	169147.0901	0	169484.7253	155623.7222	0	41661.49593
KARS	lysine--tRNA ligase isoform 2 [Homo sapiens]	2622020.833	3514235.898	3214190.232	3276634.037	3254354.046	3322830.042	3364719.935	2830062.615	3442671.264
KAT7	histone acetyltransferase KAT7 isoform 1 [Homo sapiens]	199416.974	212689.9394	155592.0476	204149.0024	267708.6696	192548.9814	173415.6339	144694.0346	205298.3861
KAT8	histone acetyltransferase KAT8 isoform 2 [Homo sapiens]	137906.1928	128965.5811	249625.901	125740.6504	249932.9143	0	0	0	0
KATNA1	katanin p60 ATPase-containing subunit A1 isoform 1 [Homo sapiens]	0	41933.72962	77886.02935	68642.24165	88211.02391	101543.4568	129890.798	43262.94891	48651.58043
KATNB1	katanin p80 WD40 repeat-containing subunit B1 [Homo sapiens]	71955.26635	32888.08269	70739.10385	28399.575	102648.1154	51392.52935	58709.96154	46436.84231	19684.55433
KCMF1	E3 ubiquitin-protein ligase KCMF1 [Homo sapiens]	752877.053	808642.8329	535218.1196	719818.1069	516605.1884	607720.5177	378061.0737	489086.537	597911.3214
KCNAB2	voltage-gated potassium channel subunit beta-2 isoform 1 [Homo sapiens]	413763.1072	767368.6174	564755.1419	0	581670.1703	0	0	0	0
KCNK1	potassium channel subfamily K member 1 [Homo sapiens]	0	0	0	0	0	0	276806.9352	324580.9352	0
KCNN4	intermediate conductance calcium-activated potassium channel protein 4 [Hor	445476.3952	0	103525.8253	112488.6862	144297.0274	114676.7003	0	0	0
KCTD10	BTB/POZ domain-containing adapter for CUL3-mediated RhoA degradation protei	0	0	103397.0055	73027.29927	139661.6861	65575.27099	0	0	0
KCTD15	BTB/POZ domain-containing protein KCTD15 isoform 2 [Homo sapiens]	0	0	0	0	0	0	3335946.627	0	7705606.146
KCTD3	BTB/POZ domain-containing protein KCTD3 [Homo sapiens]	105172.6281	62150.23212	0	57711.67184	57246.76081	48244.71555	35002.34261	53078.28269	0
KCTD5	BTB/POZ domain-containing protein KCTD5 [Homo sapiens]	2218821.109	2782938.193	121577.4831	98818.15759	491244.6915	289567.3271	338434.8963	2986272.314	188195.9774
KCTD9	BTB/POZ domain-containing protein KCTD9 [Homo sapiens]	118299.0435	136959.4539	136496.3502	87566.34043	148406.9374	152214.7039	113024.1206	0	93841.49043
KDELC1	KDEL motif-containing protein 1 precursor [Homo sapiens]	76850.75986	52958.92661	106766.4128	0	108809.1164	212042.5069	139071.9635	0	85249.52124
KDELC2	KDEL motif-containing protein 2 precursor [Homo sapiens]	396616.6707	352311.7566	429823.8583	383883.3014	435562.5693	402275.8543	380475.6051	306968.2755	318771.4272
KDELR1	ER lumen protein retaining receptor 1 [Homo sapiens]	2623727.357	1396401.205	1225699.018	1869507.313	1246857.179	2227095.518	1647708.25	742307.6964	3966245.75
KDELR2	ER lumen protein retaining receptor 2 isoform 1 [Homo sapiens]	1180380.464	608741.3275	600897.9354	1336184.141	1500516.701	0	1040772.831	0	1185638.44
KDM1A	lysine-specific histone demethylase 1A isoform b [Homo sapiens]	0	0	0	0	0	0	300799.2276	0	284194.9061
KDM1A	lysine-specific histone demethylase 1A isoform a [Homo sapiens]	0	0	0	0	0	0	300799.2276	0	284194.9061
KDM2A	lysine-specific demethylase 2A isoform a [Homo sapiens]	97890.69606	95579.5254	355166.3709	111045.8745	163744.0125	107468.381	109943.5786	112541.0367	228317.3751
KDM3B	lysine-specific demethylase 3B [Homo sapiens]	55891.0904	103353.9719	74020.30525	89532.15845	79366.65727	184715.6459	38573.17133	55161.69791	118808.2464
KDM5B	lysine-specific demethylase 5B [Homo sapiens]	12800.89539	6522.014002	0	0	12116.26999	14114.03759	0	0	0
KDM5C	lysine-specific demethylase 5C isoform 1 [Homo sapiens]	32409.32208	16904.61105	53672.98736	28309.75481	29298.83952	35027.25865	20620.23422	17169.90033	15732.35347
KDSR	3-ketodihydrosphingosine reductase precursor [Homo sapiens]	606218.7095	730327.8333	1443053.926	305996.2037	1653852.6	327757.7037	447534.6736	616909.2384	798195.6933
KEAP1	kelch-like ECH-associated protein 1 [Homo sapiens]	155405.2997	95654.7151	124767.114	204805.0809	125832.7682	70623.89461	125206.951	164394.4798	54999.76225
KHDRBS1	KH domain-containing, RNA-binding, signal transduction-associated protein 1	4550574.799	4670906.881	6085739.207	5396120.33	6329472.978	5730061.38	5832874.084	3493901.225	4696224.682
KHSRP	far upstream element-binding protein 2 [Homo sapiens]	4604078.151	4648118.105	6099076.192	4985636.1	6699614.95	4983289.575	4801219.388	4113008.369	4911561.909
KIAA0020	pumilio domain-containing protein KIAA0020 [Homo sapiens]	404198.3299	393640.3672	445708.1299	229062.2715	592906.8789	525129.5455	398589.2771	318202.0912	435340.605
KIAA0101	PCNA-associated factor isoform 1 [Homo sapiens]	1351861.734	1064873.896	1784596.815	1454771.123	1765305.734	684548.9767	680793.4331	0	535021.5116
KIAA0196	WASH complex subunit strumpellin [Homo sapiens]	116979.8756	113889.393	111091.3434	82511.52679	118587.1402	92906.82388	111860.028	86254.74204	107655.7519
KIAA0368	proteasome-associated protein ECM29 homolog [Homo sapiens]	327811.4928	347400.5099	421109.7594	366687.6915	409951.3327	363093.9174	331222.2441	247781.4688	318945.0182
KIAA0391	mitochondrial ribonuclease P protein 3 isoform 1 precursor [Homo sapiens]	146445.1466	152626.9103	222325.7871	164334.1705	171610.401	168884.7297	197522.5198	117588.5533	184542.3808
KIAA0528	C2 domain-containing protein 5 [Homo sapiens]	0	0	0	0	0	0	30968.10443	0	31058.36587
KIAA0664	clustered mitochondria protein homolog [Homo sapiens]	540637.2584	569198.6186	618209.4842	552056.497	729801.9571	580451.0407	651995.1213	463502.1582	634147.4755
KIAA0947	uncharacterized protein KIAA0947 [Homo sapiens]	9043.965938	9724.20875	4315.923438	0	60803.54649	0	0	0	0
KIAA1033	WASH complex subunit 7 [Homo sapiens]	104224.0188	88952.55214	142414.1356	105538.7119	137151.7682	120612.4412	100561.4274	89912.43658	94448.25472
KIAA1279	KIF1-binding protein [Homo sapiens]	168499.7403	340360.0003	331908.979	182486.3288	236439.4669	338747.9885	269852.2247	211462.1181	200903.1956
KIAA1429	protein virilizer homolog isoform 1 [Homo sapiens]	52868.43874	51903.97861	937270.7417	41042.18318	66162.24858	57263.6975	59687.48172	30424.40651	81342.29175
KIAA1468	lisH domain and HEAT repeat-containing protein KIAA1468 [Homo sapiens]	21502.87259	31265.78253	43915.2232	34111.8854	19703.50482	44269.04648	46642.73072	25459.23665	30964.64116
KIAA1522	uncharacterized protein KIAA1522 isoform 1 [Homo sapiens]	26613.0346	0	46276.1929	38889.51339	52543.87794	25503.19321	14134.80875	18516.61064	26270.35478
KIAA1524	protein CIP2A [Homo sapiens]	192020.4122	159575.9022	120441.6748	173546.8513	247821.4468	202392.4468	149333.0158	142124.9893	130787.9
KIAA1598	shootin-1 isoform a [Homo sapiens]	138882.2831	128942.4978	194285.4756	137748.6829	150074.7728	147742.1615	88782.77119	95560.00667	155726.1603
KIAA1671	uncharacterized protein KIAA1671 [Homo sapiens]	15596.65789	22081.57932	17629.83353	22492.74336	27148.57822	15094.56369	10628.38816	4858.713752	21705.60596
KIAA1704	uncharacterized protein KIAA1704 [Homo sapiens]	0	0	0	0	0	0	40833.32732	0	126386.7874
KIAA1715	protein lunapark [Homo sapiens]	0	0	0	0	0	0	0	0	0
KIAA1967	DBIRD complex subunit KIAA1967 [Homo sapiens]	632625.8035	605198.2135	795569.7096	703950.1637	818017.2515	723437.8482	663226.8427	537154.7477	610569.2096
KIAA2013	uncharacterized protein KIAA2013 precursor [Homo sapiens]	109857.1509	173872.2867	184110.4635	203013.2865	211412.7431	182266.8876	177204.5103	166603.2398	235062.9457
KIDINS220	kinase D-interacting substrate of 220 kDa [Homo sapiens]	30369.38239	7064.849871	40150.50447	8835.907059	37212.38463	29026.75355	30070.64038	8075.581938	24720.23731
KIF11	kinesin-like protein KIF11 [Homo sapiens]	173159.3603	205216.5859	181692.4076	212138.3431	233633.667	249139.8962	165537.1076	109970.8245	125195.5296
KIF13A	kinesin-like protein KIF13A isoform a [Homo sapiens]	85893.73767	101465.1104	30990.72457	22110.88318	37068.32461	51116.53108	97395.90764	28776.1013	28333.06974
KIF14	kinesin-like protein KIF14 [Homo sapiens]	40147.65598	41366.56671	31469.84765	46448.37323	45497.98431	26342.31241	31165.95248	18133.52012	51420.19577
KIF15	kinesin-like protein KIF15 [Homo sapiens]	30412.50187	50927.98426	73926.07559	43345.55885	68833.75663	57671.04033	125333.2099	28475.20265	32596.72621
KIF18B	kinesin-like protein KIF18B isoform 1 [Homo sapiens]	26638.98362	10474.57789	2049.222139	0	28984.76393	0	0	0	0
KIF1B	kinesin-like protein KIF1B isoform b [Homo sapiens]	45798.91996	0	54132.83603	49006.43139	61392.59782	41008.70773	47723.19092	55352.7694	59904.35016
KIF1C	kinesin-like protein KIF1C [Homo sapiens]	20341.62025	20428.33798	37062.64312	20031.17352	0	36071.54747	0	0	0
KIF20A	kinesin-like protein KIF20A [Homo sapiens]	58386.06332	61122.20514	81845.72626	71757.59637	123037.5622	38683.695	9663.816168	23398.36562	69094.52131
KIF21A	kinesin-like protein KIF21A isoform 1 [Homo sapiens]	304813.3256	418940.1818	251329.8061	218963.4964	273485.4647	230360.4587	151331.3426	942267.9075	165329.0785

KIF22	kinesin-like protein KIF22 isoform 1 [Homo sapiens]	0	0	0	0	0	0	0	47552.75808	95965.91556
KIF23	kinesin-like protein KIF23 isoform 1 [Homo sapiens]	0	501012.2833	101963.3443	426124.3367	101409.0229	337304.6624	410144.1085	0	496565.3837
KIF2A	kinesin-like protein KIF2A isoform 2 [Homo sapiens]	96946.4731	150229.7019	197630.9792	146891.3027	200721.5758	114393.1322	149081.6577	90717.02169	160634.8135
KIF2C	kinesin-like protein KIF2C [Homo sapiens]	138787.9711	199687.929	224860.3583	187523.704	213498.089	234575.3227	177984.8905	167771.4822	140917.8599
KIF4A	chromosome-associated kinesin KIF4A [Homo sapiens]	182701.7689	118813.1711	125894.4286	98576.90254	75349.56398	90732.89121	89469.87545	115878.2808	107000.9346
KIF5B	kinesin-1 heavy chain [Homo sapiens]	738046.5363	802182.4637	908194.1185	772021.6461	1068163.041	883777.7091	791533.3207	677834.7185	823326.7234
KIFC1	kinesin-like protein KIFC1 [Homo sapiens]	271592.8181	64351.85091	125478.4877	103426.8602	112018.9499	107488.6233	99461.42112	64405.28407	70548.72119
KIFC3	kinesin-like protein KIFC3 isoform 1 [Homo sapiens]	10892.85582	15099.7589	0	1374.149444	0	10096.63172	4484.748567	12092.27479	0
KIN	DNA/RNA-binding protein KIN17 [Homo sapiens]	123953.2331	88167.05797	68558.45353	43309.61353	85339.29802	40855.46558	74582.92179	299394.1347	67925.73169
KIRREL	kin of IRRE-like protein 1 precursor [Homo sapiens]	0	0	0	0	0	0	0	0	0
KLC1	kinesin light chain 1 isoform 3 [Homo sapiens]	486337.4278	504743.818	625094.0813	521993.3394	683574.2239	536247.8549	479549.7725	486830.0046	477993.0468
KLC2	kinesin light chain 2 isoform 1 [Homo sapiens]	336976.9191	345428.0549	450141.8429	392144.9014	500884.3115	425165.7934	337432.8523	165239.9855	304831.5116
KLC4	kinesin light chain 4 isoform b [Homo sapiens]	74927.15455	120156.4496	90904.15672	122947.8252	77205.78313	121191.181	90547.97325	182133.343	70806.55116
KLF16	Krueppel-like factor 16 [Homo sapiens]	202912.6904	181877.1276	303737.7782	187443.2949	229783.5665	187437.3776	175143.7075	0	252184.0136
KLF5	Krueppel-like factor 5 [Homo sapiens]	486966.4411	613165.875	0	502929.5206	672088.7446	606512.8536	382844.3607	246419.4616	200805.2179
KLHDC4	kelch domain-containing protein 4 isoform 1 [Homo sapiens]	139874.9488	291509.2391	194657.9801	242173.9337	936080.5022	195359.8801	203814.0949	99762.34167	897299.0694
KLHL8	kelch-like protein 8 [Homo sapiens]	26759.125	27828.64272	31549.75112	19421.87807	27715.23577	24668.84961	0	0	0
KNOP1	lysine-rich nucleolar protein 1 [Homo sapiens]	35324.93697	0	113727.5784	76162.72619	59266.49153	131974.0069	86820.25458	95743.16835	114045.7182
KNSTRN	small kinetochore-associated protein isoform a [Homo sapiens]	424304.1769	530633.8971	565360.6072	580602.0437	826521.4196	575390.4965	516643.7771	388040.3974	541726.5341
KNTC1	kinetochore-associated protein 1 [Homo sapiens]	41573.96947	70931.53629	72435.12842	72277.07612	69757.89607	75527.48369	44654.22245	32428.9105	43964.66374
KPNA1	importin subunit alpha-1 [Homo sapiens]	798311.2456	420760.6971	1248358.362	581698.2334	1279807.218	1020503.713	977790.1036	426840.7515	1246397.589
KPNA2	importin subunit alpha-2 [Homo sapiens]	10175015.69	10222069.14	13610217.69	11690140.87	13921975.25	11274127.73	11421882.67	9547057.101	11151565.99
KPNA3	importin subunit alpha-3 [Homo sapiens]	1602582.419	1495658.024	2059137.184	1800024.883	2154741.155	1845397.925	1569430.772	1413900.997	1669618.226
KPNA4	importin subunit alpha-4 [Homo sapiens]	2321168.529	2487545.347	3255366.684	1775846.663	3282463.2	1904633.13	1909684.962	1830497.65	2161580.136
KPNA6	importin subunit alpha-7 [Homo sapiens]	1017682.261	904221.7068	1016396.443	1101266.976	1565257.533	1140903.92	1133203.314	1043050.369	1312948.51
KPNB1	importin subunit beta-1 isoform 1 [Homo sapiens]	9651760.718	10438210.08	12994051.44	10951688.74	12817421.79	11447591.76	11022306.79	9391517.901	11575964
KPRP	keratinocyte proline-rich protein [Homo sapiens]	355568.2772	0	204126.0644	0	633730.453	445503.4827	0	320712	482011.146
KRI1	protein KRI1 homolog [Homo sapiens]	219914.8255	246178.115	326271.8757	261230.2952	272884.4747	225568.8064	205152.6822	154352.1541	232108.1474
KRR1	KRR1 small subunit processome component homolog [Homo sapiens]	442491.4299	814185.03	704150.8215	547994.169	528705.0045	676642.3515	591347.8335	426218.0546	301258.3125
KRT1	keratin, type II cytoskeletal 1 [Homo sapiens]	50924904.89	46758960.65	44865840.91	34179201.34	81528392.55	65018708.84	55492030.6	47403890.53	46572805.84
KRT10	keratin, type I cytoskeletal 10 [Homo sapiens]	54918811.19	47222344.82	45447567.72	26512699.21	89549640.04	58173384.83	56000553.67	51730038.8	48265925.22
KRT13	keratin, type I cytoskeletal 13 isoform a [Homo sapiens]	2406198.104	1781404.024	3230337.957	2094176.984	2285780.796	0	0	0	0
KRT14	keratin, type I cytoskeletal 14 [Homo sapiens]	2265983.937	1784996.468	1802746.991	1032948.576	3414738.27	2572739.952	1916711.698	1594500.929	2165830.335
KRT16	keratin, type I cytoskeletal 16 [Homo sapiens]	1432160.826	579509.7978	410536.0032	387919.299	1006680.56	1180372.834	707374.1966	880413.4966	816177.9436
KRT17	keratin, type I cytoskeletal 17 [Homo sapiens]	13474957.65	9969561.033	18090598.54	10501281.51	16842038.86	12968011.86	10827176.94	10063205.69	14090379.06
KRT18	keratin, type I cytoskeletal 18 [Homo sapiens]	47311621.55	54359441.47	56819997.17	49072458.11	61632957.78	54324799.89	51317595.81	43435329.55	55735528.48
KRT19	keratin, type I cytoskeletal 19 [Homo sapiens]	5189136.305	5315418.32	6475304.533	5419229.098	6672286.88	5487412.572	4503077.31	4133729.02	5137389.76
KRT2	keratin, type II cytoskeletal 2 epidermal [Homo sapiens]	20450117.64	17343883.12	17317071.2	9759022.771	33694279.4	21265698.83	21513714.69	18811979.02	17020645.9
KRT23	keratin, type I cytoskeletal 23 [Homo sapiens]	0	0	0	0	0	0	22760.04385	0	57770.78534
KRT5	keratin, type II cytoskeletal 5 [Homo sapiens]	2346367.331	2180511.795	1965529.776	983728.1947	4440745.444	2677856.319	2367388.13	1927607.882	2853082.307
KRT6A	keratin, type II cytoskeletal 6A [Homo sapiens]	0	0	0	0	0	0	0	0	0
KRT6B	keratin, type II cytoskeletal 6B [Homo sapiens]	211556.5867	103128.7244	190207.1004	0	0	219423.3731	0	116653.9489	592994.8316
KRT6C	keratin, type II cytoskeletal 6C [Homo sapiens]	1070888.995	601688.7351	0	405156.8875	1092843.314	0	0	751370.5991	2137436.818
KRT7	keratin, type II cytoskeletal 7 [Homo sapiens]	9354022.778	9985761.826	11651263.13	10605475.29	10998345.8	11112884.67	10716644.5	8214653.348	10352733.11
KRT72	keratin, type II cytoskeletal 72 isoform 1 [Homo sapiens]	794675.3662	0	852061.3004	75964.84868	808895.8289	226374.4089	607761.1636	377701.4868	0
KRT77	keratin, type II cytoskeletal 1b [Homo sapiens]	0	30396.39381	12677.18113	0	253325.6956	42053.52488	113017.4067	55245.95631	186431.0449
KRT78	keratin, type II cytoskeletal 78 [Homo sapiens]	476949.7647	246828.2486	223306.6214	103101.9281	527133.6622	278270.3035	245018.39	247445.0058	260226.447
KRT8	keratin, type II cytoskeletal 8 isoform 1 [Homo sapiens]	69092571.63	73389910.77	82240301.39	72558837.25	90681070.59	75110719.37	70524669.67	56371289.32	75919523.41
KRT80	keratin, type II cytoskeletal 80 isoform K80 [Homo sapiens]	176440.157	181128.5472	190951.2542	206956.9973	306438.8041	258956.9712	155762.6057	161287.2501	96130.95536
KRT9	keratin, type I cytoskeletal 9 [Homo sapiens]	29468143.27	29485212.39	32919568.87	16166777.22	50761230.16	37687806.3	29641096.99	30189687.04	27912672.95
KRTCAP2	keratinocyte-associated protein 2 [Homo sapiens]	3178166.988	4958737.388	5293627.512	4340304.157	5289446.81	0	0	3595039.226	5085182.407
KTI12	protein KTI12 homolog [Homo sapiens]	387091.7173	368240.2767	355065.5324	274592.765	457511.9185	324907.7811	68667.67366	254774.2205	238178.4056
KTN1	kinectin isoform a [Homo sapiens]	391511.219	408214.9422	448221.0172	407429.1406	493448.5639	382748.8403	401346.0791	285010.4531	447896.4049
KXD1	kxDL motif-containing protein 1 [Homo sapiens]	0	0	0	0	0	0	368524.5278	276526.1065	0
KYNU	kynureninase isoform a [Homo sapiens]	6430415.039	8729492.128	9488625.179	7089979.564	10616390.04	9140614.045	8827200.218	7532029.615	8839289.279
L1CAM	neural cell adhesion molecule L1 isoform 1 precursor [Homo sapiens]	938992.0961	0	1369085.968	1132047.418	1358640.449	1181333.953	991948.339	889416.4617	0
L2HGDH	L-2-hydroxyglutarate dehydrogenase, mitochondrial precursor [Homo sapiens]	250595.625	238784.9522	355236.657	393149.2747	373848.8504	281244.4051	336022.9902	265432.3376	257551.6658
LACTB	serine beta-lactamase-like protein LACTB, mitochondrial isoform a [Homo sap	489125.3665	595978.4804	745138.3438	705551.1964	835319.6098	711159.5049	707675.9567	557052.9643	646638.1384
LACTB2	beta-lactamase-like protein 2 [Homo sapiens]	1866020.55	2425510.555	2576184.186	2201619.035	2697305.971	2287447.917	1804312.578	1854085.269	2327755.999
LAGE3	L antigen family member 3 [Homo sapiens]	1629808.709	1431427.575	748702.9491	484398.4554	2086074.433	1442958.337	1239905.214	0	1263329.809
LAMA5	laminin subunit alpha-5 precursor [Homo sapiens]	20086.83358	8738.643626	10046.53297	7644.853779	20237.30698	14256.87051	10161.26939	2581.240702	11072.94472
LAMB1	laminin subunit beta-1 precursor [Homo sapiens]	81215.14471	65404.79578	87088.74507	109518.0892	98677.65328	88084.8453	111168.0888	76144.40485	64763.31363
LAMB3	laminin subunit beta-3 precursor [Homo sapiens]	4314.874178	7371.598822	0	2441.476357	0	5462.992496	2451.614103	0	4785.308164
LAMC1	laminin subunit gamma-1 precursor [Homo sapiens]	45985.29986	60858.51088	58079.11808	55299.4472	63765.75117	64102.57175	59287.64844	33580.90261	84452.47683
LAMP1	lysosome-associated membrane glycoprotein 1 precursor [Homo sapiens]	1297232.369	1158234.476	4228873.393	1652152.753	1328878.494	1279669.145	1434867.726	1588941.697	3543252.553
LAMP2	lysosome-associated membrane glycoprotein 2 isoform C precursor [Homo sapie	3063453.97	3823777.883	4162996.022	4019522.005	4140083.218	4066058.757	3742487.467	3047148.833	4175883.876
LAMTOR1	ragulator complex protein LAMTOR1 [Homo sapiens]	2762078.51	1354464.196	3290589.241	2860068.906	3700471.222	1803916.278	1393298.287	1216072.039	1944775.408
LAMTOR2	ragulator complex protein LAMTOR2 isoform 1 [Homo sapiens]	1252629.757	0	1444574.45	792216.55	1626019.657	0	0	0	0
LAMTOR3	ragulator complex protein LAMTOR3 isoform 1 [Homo sapiens]	1389109.907	1851154.295	1950471.535	1810980.651	1756185.126	2605873.023	1596935.948	1423502.366	1862844.57
LAMTOR4	ragulator complex protein LAMTOR4 [Homo sapiens]	2307623.695	3047523.935	3045119.278	2686016.075	2914328.323	1849905.029	2228139.46	1930762.982	2828674.105
LAMTOR5	ragulator complex protein LAMTOR5 [Homo sapiens]	2070386.314	1187675.365	1437668.322	1347159.767	834155.3627	1252383.384	917507.1888	803504.4853	1331379.833
LANCL1	lanC-like protein 1 [Homo sapiens]	2475202.326	2232355.816	2692851.909	2348225.495	2624682.568	1973838.764	1929104.887	1995503.858	2712301.095
LANCL2	lanC-like protein 2 [Homo sapiens]	489690.953	415503.9636	625246.3431	338653.3259	637604.8069	521622.1221	350293.4871	300989.1935	463083.2165
LAP3	cytosol aminopeptidase [Homo sapiens]	1984232.627	1817655.292	2111768.185	2126969.79	2372707.343	2180191.345	1701098.283	1806337.21	2354379.299
LARP1	la-related protein 1 [Homo sapiens]	567293.0357	664194.1573	776488.4469	707477.2229	832651.2386	685517.3563	642599.4611	548859.0573	646306.9949
LARP4	la-related protein 4 isoform a [Homo sapiens]	156427.6218	458130.4639	179667.2772	450509.206	655589.689	134186.079	409834.3905	362526.6971	419072.8988
LARP4	la-related protein 4 isoform b [Homo sapiens]	156427.6218	458130.4639	179667.2772	450509.206	655589.689	134186.079	409834.3905	362526.6971	419072.8988
LARP4B	la-related protein 4B [Homo sapiens]	235911.6214	347381.1267	392547.1451	358070.8959	460375.0212	483855.4773	398815.1729	302599.6784	406047.6278
LARP7	la-related protein 7 isoform 2 [Homo sapiens]	156132.4424	126297.6301	155277.6075	126580.8175	160564.279	160260.1206	128094.0305	77738.5829	259848.9728
LARS	leucine--tRNA ligase, cytoplasmic [Homo sapiens]	693091.2083	852216.3959	901054.6558	849035.7653	889390.6158	870575.9379	799201.5542	669989.3819	774378.8809
LARS2	probable leucine--tRNA ligase, mitochondrial [Homo sapiens]	166051.1435	174978.2806	220351.0627	183036.517	202229.5168	185801.3619	191382.3762	163326.0993	220815.069
LAS1L	ribosomal biogenesis protein LAS1L isoform 1 [Homo sapiens]	388455.5037	437918.7871	446345.7199	411323.1668	389304.1432	424703.4542	433579.4487	333812.2703	343129.06
LASP1	LIM and SH3 domain protein 1 isoform a [Homo sapiens]	7200671.73	8493974.177	8274897.623	8174792.626	9175325.757	8331658.277	7891089.518	6899930.5	8612481.248
LBR	lamin-B receptor [Homo sapiens]	589222.4952	962890.6595	1189696.735	836956.8361	779002.793	1100001.069	801214.9088	772526.2987	1051845.057
LCLAT1	lysocardiolipin acyltransferase 1 isoform 1 [Homo sapiens]	294459.0398	420314.4633	497856.0788	356191.2803	600334.5172	377081.6347	335969.381	250345.7753	512588.9516
LCMT1	leucine carboxyl methyltransferase 1 isoform a [Homo sapiens]	181816.587	241728.4522	335544.9467	299768.3873	391738.3505	202285.0668	228079.9877	299798.1241	195432.4311
LDHA	L-lactate dehydrogenase A chain isoform 3 [Homo sapiens]	89626347.89	95102133.49	111096780.4	102611838.9	120158991	113131739.9	102753486.2	79807351.87	97235408.4
LDHB	L-lactate dehydrogenase B chain [Homo sapiens]	68245645.18	80771443.51	89712547.06	76554247.26	93891786.79	79030910.17	80147436.55	65025683.28	82282217.38
LDLR	low-density lipoprotein receptor isoform 1 precursor [Homo sapiens]	70135.59155	147951.2506	102822.0872	83094.07197	135925.1397	168887.4833	204324.8342	111247.9908	191636.3307
LEMD2	LEM domain-containing protein 2 isoform 1 [Homo sapiens]	151845.9408	113852.5534	144404.6046	137180.5667	142949.7729	270235.6652	164380.7352	99304.39758	298709.9526
LEMD3	inner nuclear membrane protein Man1 isoform 1 [Homo sapiens]	57029.03631	135100.8128	152434.6625	46668.56612	145242.0065	570372.5196	63821.01483	538231.0983	219358.0533
LENG1	leukocyte receptor cluster member 1 [Homo sapiens]	0	147199.6657	49049.60639	72538.37257	41111.9741	172643.634	108817.6086	39521.07586	189189.9243
LENG8	leukocyte receptor cluster member 8 [Homo sapiens]	0	0	0	0	0	0	9938.276815	55694.09809	62988.7364
LENG9	leukocyte receptor cluster member 9 [Homo sapiens]	142639.375	0	180591.4008	0	222580.0706	78578.56324	58773.00588	55857.36103	0
LEO1	RNA polymerase-associated protein LEO1 [Homo sapiens]	98935.99076	41839.58032	201894.0687	237243.5012	291054.7354	243078.7527	142078.6783	271198.2109	112529.9242
LEPRE1	prolyl 3-hydroxylase 1 isoform 1 precursor [Homo sapiens]	166370.1946	147133.5677	0	150519.0841	195667.5515	181716.9363	0	151284.73	218882.187
LEPRE1	prolyl 3-hydroxylase 1 isoform 3 precursor [Homo sapiens]	166370.1946	147133.5677	0	150519.0841	195667.5515	181716.9363	0	151284.73	218882.187
LEPROTL1	leptin receptor overlapping transcript-like 1 isoform 2 [Homo sapiens]	219166.4412	258623.1373	350186.8578	209867.5392	383913.5931	0	202954.3824	0	203900.3137
LETM1	LETM1 and EF-hand domain-containing protein 1, mitochondrial precursor [Hor	870705.503	1061409.755	1228968.336	1060377.642	1250667.374	1047876.261	1126995.067	1029562.747	1325454.55
LETMD1	LETM1 domain-containing protein 1 isoform 3 [Homo sapiens]	0	0	0	0	0	0	0	0	0
LGALS1	galectin-1 [Homo sapiens]	66164475.41	68246464.43	68585938.02	72286628.14	77413354.86	75279366.43	72359064.53	62305464.24	73982027.16
LGALS3	galectin-3 isoform 1 [Homo sapiens]	11370308.88	9788638.798	13184038.81	11950960.8	15522423.72	12194511.43	11725637.15	9791530.552	13021450.85
LGALS3BP	galectin-3-binding protein precursor [Homo sapiens]	1201609.319	1456252.147	1714096.817	1408415.117	1670963.43	1152238.169	1265232.055	1031554.938	1385996.267
LGALS7B	galectin-7 [Homo sapiens]	3833825.419	2837109.166	4638664.183	2560743.327	4265903.249	4909133.344	3498334.531	3105410.2	4533635.48
LGALS8	galectin-8 isoform a [Homo sapiens]	0	145850.2649	157408.4112	76329.01237	331086.4605	141101.5888	92021.99138	72827.38816	190883.9844
LGMN	legumain preproprotein [Homo sapiens]	300127.0801	256725.1994	327052.9114	286831.013	357900.4776	120900.4223	109279.8904	223123.136	123537.005
LHPP	phospholysine phosphohistidine inorganic pyrophosphate phosphatase isoform	0	0	0	0	0	0	0	0	0
LIG1	DNA ligase 1 [Homo sapiens]	194904.1675	183898.3965	249523.5874	176328.5858	260394.9052	180233.3088	191921.5153	142454.5082	185745.6394
LIG3	DNA ligase 3 isoform alpha precursor [Homo sapiens]	67343.14857	103773.8825	137062.3437	119604.1101	138861.4889	92676.72401	118683.3135	96398.2901	117198.3768
LIMA1	LIM domain and actin-binding protein 1 isoform 1 [Homo sapiens]	1789737.43	2035637.823	2267126.511	2070101.46	2141248.698	2143246.58	2061654.688	1807703.031	2248604.025
LIMCH1	LIM and calponin homology domains-containing protein 1 isoform a [Homo sap	171652.2032	156755.5357	203616.2033	166355.4993	226368.2657	191046.6542	153699.7382	2096401.661	188531.7508
LIMD1	LIM domain-containing protein 1 [Homo sapiens]	117251.3773	101865.297	32640.55882	0	0	99313.44118	0	0	0
LIMS1	LIM and senescent cell antigen-like-containing domain protein 1 isoform a [	0	0	0	0	0	0	243449.9849	0	415868.1437
LIN7C	protein lin-7 homolog C [Homo sapiens]	760185.2889	809489.8596	1052859.03	601133.4401	1055480.412	888849.3258	721639.397	505193.1962	848915.2739
LIN9	protein lin-9 homolog isoform 1 [Homo sapiens]	55222.92886	72113.10976	78781.32927	0	102211.1575	86645.32317	183305.5366	72700.12667	0
LIPA	lysosomal acid lipase/cholesteryl ester hydrolase precursor [Homo sapiens]	449890.2056	184965.4953	528343.5117	381443.9463	0	728609.7196	0	0	0
LLGL1	lethal(2) giant larvae protein homolog 1 [Homo sapiens]	63256.80121	104302.3483	61663.8535	44085.66978	122834.2281	58200.86401	86953.48885	105487.3646	70288.79134
LLGL2	lethal(2) giant larvae protein homolog 2 isoform c [Homo sapiens]	0	87485.59887	61040.05774	94512.59147	60386.30508	83969.31427	62947.70621	62814.83192	40932.24506
LMAN1	protein ERGIC-53 precursor [Homo sapiens]	2772813.723	2809249.826	2473364.001	2211351.594	2456470.517	2346939.576	2923896.227	2356751.38	2066395.751
LMAN2	vesicular integral-membrane protein VIP36 precursor [Homo sapiens]	3389432.654	3656389.118	4663093.135	2911257.408	4403281.302	4004131.261	3970434.288	2915371.014	3117479.671

LMAN2L	VIP36-like protein isoform 1 precursor [Homo sapiens]	286843.0247	352669.3193	290043.8412	240731.0379	291560.3601	258028.4799	0	0	0
LMF2	lipase maturation factor 2 [Homo sapiens]	153551.1566	233299.0118	177310.4412	233060.1318	264125.1628	229210.7632	265465.9019	143094.5371	547793.7091
LMNA	lamin isoform A [Homo sapiens]	2041263.706	2183852.607	2958762.13	2541145.508	3075207.542	2415480.281	1845942.488	1833806.391	2463008.682
LMNA	lamin isoform C [Homo sapiens]	2041263.706	2183852.607	2958762.13	2541145.508	3075207.542	2415480.281	1845942.488	1833806.391	2463008.682
LMNB1	lamin-B1 isoform 1 [Homo sapiens]	2907768.789	3769470.646	3458735.183	2990545.597	3692015.825	2988504.045	3134727.279	2843855.532	3595120.557
LMNB2	lamin-B2 [Homo sapiens]	741987.2518	835845.5393	950482.0748	761646.4918	1009194.408	832306.1951	993199.9295	860751.6761	752479.4767
LMO7	LIM domain only protein 7 isoform 1 [Homo sapiens]	0	0	0	0	0	0	47705.97737	45955.46454	46756.45993
LMO7	LIM domain only protein 7 isoform 2 [Homo sapiens]	0	0	0	0	0	0	47705.97737	45955.46454	46756.45993
LNPEP	leucyl-cystinyl aminopeptidase isoform 1 [Homo sapiens]	183149.2349	187580.1412	205320.5875	213119.1222	232625.8769	200604.4139	219385.6674	186976.4461	251207.6669
LOC100507855	PREDICTED: adenylate kinase isoenzyme 4, mitochondrial-like [Homo sapiens]	1550472.045	1740548.421	1703050.148	1706709.115	1468881.014	1752742.752	1888178.227	1765343.811	1941723.592
LOC100996516	PREDICTED: uncharacterized protein LOC100996516 [Homo sapiens]	395143.5092	376343.7615	0	353239.9725	0	322135.5138	354225.0436	0	150270.422
LOC100996752	PREDICTED: uncharacterized protein LOC100996752 [Homo sapiens]	246723.9371	403777.5192	0	314392.8776	0	296600.8374	315359.9292	218015.8331	0
LOC100996848	PREDICTED: FERM and PDZ domain-containing protein 3-like [Homo sapiens]	0	0	0	0	0	0	0	232087.4624	98786.68936
LOC101059922	PREDICTED: uncharacterized LOC101059922 [Homo sapiens]	692894.8643	1506635.125	1857473.386	1448476.171	0	1703728.871	1447240.654	1201199.164	1832409.714
LOC101060291	PREDICTED: myomegalin-like isoform 12 [Homo sapiens]	8988.918585	9335.020156	11706.42317	10398.70727	71831.48977	8985.738428	0	0	0
LOC101060316	PREDICTED: rho guanine nucleotide exchange factor 5-like [Homo sapiens]	17348.84888	17699.61433	11388.40785	16249.32703	12404.5	18741.73834	15240.94228	12970.8806	15048.01941
LOC101060318	PREDICTED: eukaryotic translation initiation factor 1A, X-chromosomal-like	0	0	17963693.03	15956416.02	18692693.37	16746013.6	15339038.27	12268770.13	19410643.75
LOC101060460	PREDICTED: DNA-directed RNA polymerase III subunit RPC3-like isoform 2 [Hor	97268.60132	190702.9007	115019.2747	109957.762	108484.0257	126013.8336	152378.5813	144607.8456	193194.0468
LOC101060521	PREDICTED: DNA-directed RNA polymerase III subunit RPC5-like isoform 4 [Hor	14349.07309	61816.92319	50613.56702	99886.97346	52600.21468	90053.88363	57472.64258	83475.55651	97457.58444
LOC101060541	PREDICTED: RNA-binding protein 8A-like isoform 2 [Homo sapiens]	9700818.831	11379258.01	13322643.02	11406000.41	14290176.58	11643304.67	11801168.58	11069863.07	13435161.75
LOC101060547	PREDICTED: LIX1-like protein-like [Homo sapiens]	0	0	0	0	0	0	0	176452.9726	201243.3373
LOC101060567	PREDICTED: peroxisomal membrane protein 11B-like isoform 1 [Homo sapiens]	0	0	0	0	0	0	0	80418.94101	171310.2839
LOC101060601	PREDICTED: chromodomain-helicase-DNA-binding protein 1-like isoform 2 [Homo	0	57331.84016	72196.42931	77606.44809	106799.1188	85753.06848	80538.6137	58126.85194	81107.79965
LOC101060672	PREDICTED: neuroblastoma breakpoint family member 14-like [Homo sapiens]	0	0	0	0	0	0	875154.9085	1374142.646	1021321.917
LOC101060714	PREDICTED: sulfotransferase 1A3/1A4-like isoform 2 [Homo sapiens]	511664.4669	370191.6107	544643.2422	644677.1223	548519.7192	573199.951	511433.9099	483317.3514	574743.3716
LOC101060751	PREDICTED: nucleoside diphosphate kinase B-like [Homo sapiens]	5361085.966	12001656.33	10055809.07	9205314.167	10426131.78	9801810.167	10483320.82	7132306.049	11326866.7
LOC391322	D-dopachrome decarboxylase related protein [Homo sapiens]	6975509.593	9006382.337	8942281.198	3232180.158	7778032.716	7694739.186	6910947.907	3288156.837	7991812.779
LOC650157	PREDICTED: peptidyl-prolyl cis-trans isomerase A-like [Homo sapiens]	20973437.25	26106348.05	15907260.88	16701626.97	24569387.53	14540797.56	23417229.12	26544553.56	23115129.21
LONP1	lon protease homolog, mitochondrial isoform 1 precursor [Homo sapiens]	916848.8166	1773801.25	1248977.796	965997.7595	1232118.309	1025507.25	1052328.546	1033188.859	1076575.095
LONP2	lon protease homolog 2, peroxisomal [Homo sapiens]	108635.9046	104187.2343	124450.6929	70630.55565	143429.6721	118826.0184	72808.02015	62799.28274	0
LPCAT1	lysophosphatidylcholine acyltransferase 1 [Homo sapiens]	983199.1271	575206.6809	868685.1351	1069286.438	1249372.238	1085776.398	1080418.476	659576.7824	1116961.202
LPCAT3	lysophospholipid acyltransferase 5 [Homo sapiens]	541085.7388	581766.9851	700859.1264	548953.832	652872.2253	587540.6502	505892.5778	288315.1497	370335.6612
LPGAT1	acyl-CoA:lysophosphatidylglycerol acyltransferase 1 [Homo sapiens]	180055.5666	152431.5294	197515.8006	205419.8043	163577.0368	211643.9118	261573.7426	169990.5821	0
LPP	lipoma-preferred partner isoform a [Homo sapiens]	352716.1351	351364.8004	488280.9078	415449.2619	509510.6358	448095.9749	417164.1729	346121.1922	491976.7504
LRBA	lipopolysaccharide-responsive and beige-like anchor protein isoform 2 [Homo	12049.05144	7670.352655	21172.07596	20332.87061	24926.48776	11573.2022	2879.887084	14783.53149	8169.961755
LRBA	lipopolysaccharide-responsive and beige-like anchor protein isoform 1 [Homo	12049.05144	7670.352655	21172.07596	20332.87061	24926.48776	11573.2022	2879.887084	14783.53149	8169.961755
LRCH1	leucine-rich repeat and calponin homology domain-containing protein 1 isof	44380.71504	77004.92428	56843.19068	0	96093.32627	68560.93962	93539.22881	63109.27966	33562.26483
LRP10	low-density lipoprotein receptor-related protein 10 precursor [Homo sapiens	0	0	0	0	0	0	9055909.679	0	5407797.194
LRP8	low-density lipoprotein receptor-related protein 8 isoform 1 precursor [Hor	35385.41794	96202.91334	72760.20629	85477.51975	45758.75408	68200.01267	60213.27083	55589.19402	50716.53107
LRPAP1	alpha-2-macroglobulin receptor-associated protein precursor [Homo sapiens]	491583.1804	572288.7401	731386.8244	558941.2777	507339.235	540189.0147	373116.371	337062.0122	531471.9254
LRPPRC	leucine-rich PPR motif-containing protein, mitochondrial precursor [Homo sa	1206365.585	1339757.964	1659434.605	1409740.145	1725395.698	1360922.545	1360495.381	1161818.068	1497870.722
LRRC1	leucine-rich repeat-containing protein 1 [Homo sapiens]	76891.63649	0	103115.7057	63285.09189	108622.1345	61895.97297	39926.42838	48998.19054	67572.37568
LRRC16A	leucine-rich repeat-containing protein 16A isoform 1 [Homo sapiens]	478034.3128	127085.7144	138522.9605	127600.1696	152149.5381	124953.8663	129725.4839	104541.1099	120815.0914
LRRC40	leucine-rich repeat-containing protein 40 [Homo sapiens]	406684.0626	431571.0786	518152.6704	429737.4152	532263.8309	492241.8622	464864.7991	412768.6855	413989.9
LRRC41	leucine-rich repeat-containing protein 41 [Homo sapiens]	91522.75059	84071.85787	115769.1052	92689.18091	105755.1956	100983.2275	77953.27237	63242.87007	91809.11052
LRRC47	leucine-rich repeat-containing protein 47 [Homo sapiens]	720753.6257	2063757.05	938714.5035	649476.5126	1003729.764	1953388.371	646619.413	1509589.974	860328.9704
LRRC57	leucine-rich repeat-containing protein 57 [Homo sapiens]	0	0	0	0	0	0	55752.40979	0	1607955.871
LRRC59	leucine-rich repeat-containing protein 59 [Homo sapiens]	10172782.95	11002067.65	13467881.15	11675046.68	12357651.19	11506350.44	11716229.77	10238184.28	11974157.63
LRRC8A	leucine-rich repeat-containing protein 8A [Homo sapiens]	55693	117958.6379	0	52615.36196	63285.5715	0	0	0	0
LRRC8D	leucine-rich repeat-containing protein 8D [Homo sapiens]	52184.70744	69606.94522	82370.4	76438.07825	80180.55991	79251.90103	72133.80259	66053.11319	72999.17034
LRRC8E	leucine-rich repeat-containing protein 8E isoform 1 [Homo sapiens]	0	0	0	0	0	0	0	0	0
LRRFIP1	leucine-rich repeat flightless-interacting protein 1 isoform 3 [Homo sapien	269753.135	340820.3607	395246.5893	0	373568.9013	311513.4429	0	292813.2952	339346.5367
LRRFIP2	leucine-rich repeat flightless-interacting protein 2 isoform 1 [Homo sapien	101102.6559	114306.4332	164373.0332	145392.1124	94616.61118	159191.9164	123786.5442	85499.47694	118175.802
LRSAM1	E3 ubiquitin-protein ligase LRSAM1 isoform 1 [Homo sapiens]	49845.08775	70660.42126	136114.4448	59113.57036	187492.8837	86394.85043	116251.1167	57455.91556	57006.87831
LRWD1	leucine-rich repeat and WD repeat-containing protein 1 [Homo sapiens]	139841.8868	242955.11	221721.5844	210647.4519	225469.8846	209650.4185	215624.7734	158335.2702	174024.3892
LSG1	large subunit GTPase 1 homolog [Homo sapiens]	294806.6511	256611.207	400863.2355	320921.6736	401098.9553	346547.73	342165.6242	230096.2015	352221.9737
LSM1	U6 snRNA-associated Sm-like protein LSm1 [Homo sapiens]	1656440.215	1592208.965	1922426.338	1115934.839	1364565.227	2077042.107	795690.6305	824154.4417	1095391.902
LSM12	protein LSM12 homolog [Homo sapiens]	3485061.732	3576424.991	4261737.716	4255415.857	4022865.229	3981180.087	4409605.46	3893196.099	3124607.406
LSM14A	protein LSM14 homolog A isoform b [Homo sapiens]	308412.2165	514639.6	247480.6553	372872.3657	319143.3463	582048.0781	505221.6591	447193.493	429754.4146
LSM14B	protein LSM14 homolog B [Homo sapiens]	197215.0454	120394.1525	248770.0417	256745.7158	301775.7896	195068.7235	129741.9871	0	118022.6289
LSM2	U6 snRNA-associated Sm-like protein LSm2 [Homo sapiens]	5324883.758	7056604.633	8384047.058	7007767.445	8737057.685	7708271.328	7095143.695	5937118.293	7078040.64
LSM3	U6 snRNA-associated Sm-like protein LSm3 [Homo sapiens]	7446167.6	9872032.175	10482386.71	9284483.192	9568175.547	10113221.74	9425302.989	8418589.772	9796561.189
LSM4	U6 snRNA-associated Sm-like protein LSm4 isoform 1 [Homo sapiens]	1284298.225	1794722.517	2135252.799	1865554.417	2293751.569	2068375.2	1529921.868	1766190.348	1847856.648
LSM5	U6 snRNA-associated Sm-like protein LSm5 isoform a [Homo sapiens]	10907176.7	12134650.75	25157914.04	12693230.14	13563628.21	13455806.23	12425035.78	12409346.7	20644626.95
LSM6	U6 snRNA-associated Sm-like protein LSm6 [Homo sapiens]	5026323.903	4877509.661	0	3266272.533	886610.9236	2516285.889	1570522.654	2890995.183	3326121.433
LSM7	U6 snRNA-associated Sm-like protein LSm7 [Homo sapiens]	4336283.412	4195125.529	5055287.31	7685280.921	4208161.517	5766177.157	4974938.881	1957184.231	4745290.8
LSMD1	LSM domain-containing protein 1 precursor [Homo sapiens]	0	0	0	0	0	0	357948.12	143737.61	247400.9233
LSS	lanosterol synthase isoform 1 [Homo sapiens]	182138.7229	139489.6263	113668.9663	151657.6164	207056.1252	165336.2421	226174.124	139974.1325	114324.2373
LTA4H	leukotriene A-4 hydrolase isoform 1 [Homo sapiens]	2130800.159	2517302.132	2818776.122	2484576.641	2811464.262	2452555.908	2398381.187	1987678.632	2748474.363
LTBR	tumor necrosis factor receptor superfamily member 3 isoform 1 precursor [Hc	172001.0221	29825.78157	267021.1364	182773.8428	43754.72475	38673.98611	18136.29735	0	153152.1313
LTF	lactotransferrin isoform 1 precursor [Homo sapiens]	310151.7097	0	0	217814.8704	352285.6654	209253.1574	431233.4648	395954.402	0
LTN1	E3 ubiquitin-protein ligase listerin [Homo sapiens]	48129.92282	37724.74403	73515.73127	70644.00876	72400.93137	123572.7164	71014.46326	56927.92709	26677.8992
LTV1	protein LTV1 homolog [Homo sapiens]	183756.4251	202762.9334	262149.5098	202653.6616	242633.1673	251486.4851	223359.7925	91410.40404	327008.3273
LUC7L	putative RNA-binding protein Luc7-like 1 isoform b [Homo sapiens]	275802.038	224785.2646	254865.2658	408070.0567	493303.0874	261449.64	313303.1626	328209.7549	366298.2549
LUC7L2	putative RNA-binding protein Luc7-like 2 isoform 1 [Homo sapiens]	2048844.83	2548377.945	3428191.97	2675952.006	3324239.792	2332867.923	2451336.306	2023711.239	2669596.527
LUC7L3	luc7-like protein 3 [Homo sapiens]	1417373.546	1583848.652	1988819.998	1526791.535	2012211.167	1758326.635	1383687.703	998999.996	1647227.251
LUM	lumican precursor [Homo sapiens]	86335.59845	89109.4823	283833.6532	76857.64823	277888.4425	362257.7865	0	0	0
LUZP1	leucine zipper protein 1 [Homo sapiens]	40841.03759	40342.99269	34450.79132	31999.01085	44113.13148	33452.15183	43615.59275	45703.64933	350884.6928
LY6K	lymphocyte antigen 6K isoform 1 precursor [Homo sapiens]	730067.0588	807288.6029	1082885.038	816655.5588	659524.8031	948654.3235	0	0	0
LYAR	cell growth-regulating nucleolar protein [Homo sapiens]	1397506.221	1363793.944	1999722.484	1607273.281	2181606.517	1735318.508	1643943.681	1188649.148	1610282.525
LYN	tyrosine-protein kinase Lyn isoform B [Homo sapiens]	0	0	0	0	0	0	135729.1294	255313.9651	0
LYPLA1	acyl-protein thioesterase 1 [Homo sapiens]	4832291.918	5277658.595	7785250.772	5834269.217	7334584.932	5601318.8	5479576.912	5310546.383	6370942.492
LYPLA2	acyl-protein thioesterase 2 [Homo sapiens]	2271559.441	2866501.537	4279709.799	3550920.561	4232738.68	3690835.899	3631539.99	2460450.885	3562941.592
LYPLAL1	lysophospholipase-like protein 1 [Homo sapiens]	0	0	0	0	0	0	149569.1	0	261479.0938
LYRM4	LYR motif-containing protein 4 isoform 1 [Homo sapiens]	0	0	0	0	0	0	0	406344.5439	163560.3991
LYRM7	complex III assembly factor LYRM7 [Homo sapiens]	1501881.354	1250709.043	1787243.909	1563006.866	1809697.909	1075105.458	1020163.662	1253548.011	1121999.942
LYSMD2	lysM and putative peptidoglycan-binding domain-containing protein 2 isofor	0	348019.0661	146825.2174	540628.0603	441711.0284	548099.1236	496028.0072	0	575898.6437
LYZ	lysozyme C precursor [Homo sapiens]	0	0	0	0	0	0	0	797429.0152	1243235.428
LZIC	protein LZIC [Homo sapiens]	720051.3629	825050.1783	1271191.316	790871.4331	1138505.453	781896.612	840538.5219	905644.3169	1125398.035
LZTFL1	leucine zipper transcription factor-like protein 1 isoform 1 [Homo sapiens]	165571.5827	0	274645.3845	108316.4752	186097.7762	81684.31574	0	0	0
LZTR1	leucine-zipper-like transcriptional regulator 1 [Homo sapiens]	24171.64857	36368.1283	12419.4127	22356.8065	0	28430.55666	20752.16906	15260.04877	0
M6PR	cation-dependent mannose-6-phosphate receptor isoform 1 precursor [Homo sap	995041.5521	2559144.811	1732389.779	2317705.034	2846756.28	2535666.806	2747870.404	2730015.717	2727015.012
MACF1	microtubule-actin cross-linking factor 1 [Homo sapiens]	16687.92065	32008.53627	25892.64537	33815.52823	25123.54739	35576.0483	30359.03856	24792.52374	19160.94345
MACROD1	O-acetyl-ADP-ribose deacetylase MACROD1 [Homo sapiens]	1009850.892	1372942.647	1608208.175	970314.1229	1647578.178	1233944.258	1335471.201	1209307.248	1502281.322
MAD1L1	mitotic spindle assembly checkpoint protein MAD1 [Homo sapiens]	94062.90325	134564.7908	131687.5856	95638.64919	110081.3137	130693.7937	127521.4602	95986.80961	150434.6921
MAD2L1	mitotic spindle assembly checkpoint protein MAD2A [Homo sapiens]	115478.6928	1365384.431	0	1291107.531	183296.5	1193548.982	1459005.416	1009616.033	977665.1905
MAD2L1BP	MAD2L1-binding protein isoform 1 [Homo sapiens]	165700.8909	138480.2912	152130.925	167547.5318	133067.3227	116819.4182	0	0	0
MADD	MAP kinase-activating death domain protein isoform d [Homo sapiens]	26521.03766	0	0	14348.05636	20128.01229	6381.600715	6382.01822	3652.092585	0
MAEA	macrophage erythroblast attacher isoform 1 [Homo sapiens]	121430.8442	161509.4433	189970.4006	140118.408	178263.4415	156934.9781	152280.3751	170415.9823	201344.407
MAFK	transcription factor MafK [Homo sapiens]	0	0	0	0	0	0	0	0	0
MAGEA1	melanoma-associated antigen 1 [Homo sapiens]	683955.1456	655758.6307	786143.5631	751219.7965	754111.1279	646883.907	597118.055	505714.3437	657420.5478
MAGEA6	melanoma-associated antigen 6 [Homo sapiens]	468083.109	370971.8343	435483.9064	302070.2768	447165.5405	678432.5802	0	180500.1683	505595.874
MAGED2	melanoma-associated antigen D2 [Homo sapiens]	691501.4187	596651.0227	924497.7976	820793.6367	774721.7163	845658.7869	674117.8299	635067.2622	721920.4801
MAGOHB	protein mago nashi homolog 2 [Homo sapiens]	7770815.956	3716417.132	10011656.56	5613776.608	10364280.38	2793616.257	5383277.983	5079791.59	2681627.931
MAGT1	magnesium transporter protein 1 [Homo sapiens]	1399302.978	1060387.928	1904776.589	735464.7374	2200006.46	878441.9914	1073139.251	1090538.781	1799484.042
MAK16	protein MAK16 homolog [Homo sapiens]	443001.0762	537680.9938	212073.4631	632714.7548	652384.0479	481318.825	466747.5551	509399.0389	594083.9732
MALSU1	mitochondrial assembly of ribosomal large subunit protein 1 [Homo sapiens]	0	0	0	0	0	0	104191.421	75606.69038	151921.0274
MALT1	mucosa-associated lymphoid tissue lymphoma translocation protein 1 isoform	278013.0187	307526.6843	348011.5429	295911.1274	389594.5897	330009.0343	352339.1246	272789.4979	325991.796
MAN1A2	mannosyl-oligosaccharide 1,2-alpha-mannosidase IB [Homo sapiens]	0	0	0	0	0	0	54582.78274	76031.27234	35496.52827
MAN1B1	endoplasmic reticulum mannosyl-oligosaccharide 1,2-alpha-mannosidase [Homo	157063.4996	109841.4814	146149.9414	87760.9997	0	171076.2496	103091.2221	100686.8529	144915.5827
MAN2A1	alpha-mannosidase 2 [Homo sapiens]	103723.664	182446.1424	97878.81074	92778.40903	130514.217	112302.7339	118640.4713	89339.71535	114301.6537
MAN2B1	lysosomal alpha-mannosidase isoform 1 precursor [Homo sapiens]	138902.857	188456.2508	294476.1179	218050.1444	182751.3031	102324.5632	182892.4701	143516.8192	227229.9282
MANF	mesencephalic astrocyte-derived neurotrophic factor precursor [Homo sapiens	3503892.62	3851407.281	4341624.352	3786351.298	4745714.834	3779060.86	3918375.524	3043076.608	4254015.982
MAOB	amine oxidase [flavin-containing] B [Homo sapiens]	206718.4322	609094.3045	265288.2682	234260.9004	392160.7136	601136.2945	584975.8106	187934.5276	170337.3386
MAP1LC3B	microtubule-associated proteins 1A/1B light chain 3B [Homo sapiens]	1692230.854	1194021.039	2110949.878	1772579.611	2227857.254	1881716.315	1692271.644	1393164.734	1657673.861
MAP1S	microtubule-associated protein 1S [Homo sapiens]	98071.31707	124020.9897	165514.2026	156569.3658	100171.8206	147479.6822	141927.1527	85132.1863	135612.5221
MAP2K1	dual specificity mitogen-activated protein kinase kinase 1 [Homo sapiens]	1182622.939	1623113.991	2954247.051	1620111.166	2754256.483	1719777.13	919416.175	527944.779	1541156.493
MAP2K2	dual specificity mitogen-activated protein kinase kinase 2 [Homo sapiens]	901520.0168	1182827.434	963673.9217	1096209.192	1142355.739	1270650.394	1095896.56	764864.5938	746050.7969
MAP2K3	dual specificity mitogen-activated protein kinase kinase 3 isoform B [Homo	550802.863	823549.9767	922759.7137	590518.6283	910795.3466	634425.9224	882201.863	709888.0068	995396.6644
MAP2K4	dual specificity mitogen-activated protein kinase kinase 4 [Homo sapiens]	279754.3034	240698.352	337113.6196	236207.1114	318410.9366	290979.316	307760.2888	315079.2975	230538.8942

MAP2K6	dual specificity mitogen-activated protein kinase kinase 6 [Homo sapiens]	523588.6442	468538.9994	648567.8456	602379.7413	639147.0594	588845.3872	512196.7489	419921.9811	445253.9023
MAP2K7	dual specificity mitogen-activated protein kinase kinase 7 [Homo sapiens]	25467.90666	91380.52178	191704.4856	14956.70092	141380.6577	189859.4837	184318.9365	21992.94634	192754.8914
MAP3K2	mitogen-activated protein kinase kinase kinase 2 [Homo sapiens]	0	48933.20953	27385.32628	54074.56398	25445.62992	0	0	0	0
MAP3K7	mitogen-activated protein kinase kinase kinase 7 isoform B [Homo sapiens]	41362.72788	39672.85951	69442.91982	88786.22124	78635.86199	51784.51385	0	0	0
MAP4	microtubule-associated protein 4 isoform 1 [Homo sapiens]	1741207.635	1873721.331	2044832.644	1910572.272	2266287.397	1864125.978	1828861.792	1514526.821	2005142.67
MAP4K4	mitogen-activated protein kinase kinase kinase kinase 4 isoform 2 [Homo sap	0	81851.26275	71573.02189	50012.45101	26224.8006	25788.8924	54252.35943	29920.77804	122567.8498
MAP7	ensconsin isoform 1 [Homo sapiens]	0	0	0	0	0	0	135871.5547	108097.1455	179682.5004
MAP7	ensconsin isoform 2 [Homo sapiens]	0	0	0	0	0	0	135871.5547	108097.1455	179682.5004
MAP7D1	MAP7 domain-containing protein 1 [Homo sapiens]	102843.6086	34002.34315	53104.52807	55281.65834	37937.96552	110804.1719	97997.62175	88836.55641	73665.48975
MAPK1	mitogen-activated protein kinase 1 [Homo sapiens]	3519412.376	4287018.177	4554945.567	3949860.554	4561226.866	4046461.693	4114738.646	3175157.699	4644983.403
MAPK14	mitogen-activated protein kinase 14 isoform 2 [Homo sapiens]	1044710.729	1078806.142	1334066.903	1127524.626	1526876.61	1060801.848	1216024.691	755352.2467	1311530.64
MAPK1IP1L	MAPK-interacting and spindle-stabilizing protein-like [Homo sapiens]	33637322.07	39682514.43	38644610.82	43078244.38	49867246.68	49309046.86	39213433.5	42833269.36	45041258.81
MAPK3	mitogen-activated protein kinase 3 isoform 1 [Homo sapiens]	778915.4092	772424.4638	838002.8204	786856.0382	821407.3401	867615.1059	801869.5336	456207.6941	453690.2741
MAPKAP1	target of rapamycin complex 2 subunit MAPKAP1 isoform 1 [Homo sapiens]	61209.69464	35524.46618	114367.9831	79157.40039	95976.84483	80496.24894	90159.43319	73167.78444	25871.3285
MAPKAPK2	MAP kinase-activated protein kinase 2 isoform 2 [Homo sapiens]	4925771.389	80344.17284	107901.3704	342527.9475	0	90052.60765	0	0	0
MAPKAPK3	MAP kinase-activated protein kinase 3 [Homo sapiens]	189278.5771	172211.5377	256226.0788	0	312768.6242	161515.4521	256961.854	2535496.215	273880.2466
MAPRE1	microtubule-associated protein RP/EB family member 1 [Homo sapiens]	5568099.701	7589978.666	6793170.103	6064224.111	6849183.188	7980553.017	7628994.113	5896685.819	8451464.166
MAPRE2	microtubule-associated protein RP/EB family member 2 isoform 1 [Homo sapien	325971.2526	0	482784.1458	0	645747.2167	652238.775	547283.1167	274365.1167	476109.5417
MARCKS	myristoylated alanine-rich C-kinase substrate [Homo sapiens]	2946257.7	3187068.954	2921980.177	2516436.724	3240585.83	3057424.446	2964839.919	3410262.256	3240157.589
MARCKSL1	MARCKS-related protein [Homo sapiens]	1160577.907	3137334.777	1183063.378	1109088.102	1155147.279	1651280.769	123811.4892	0	1173901.077
MARK2	serine/threonine-protein kinase MARK2 isoform d [Homo sapiens]	148044.8673	117664.8086	143717.0165	0	148323.3161	0	167143.7283	89908.59714	102342.3612
MARK3	MAP/microtubule affinity-regulating kinase 3 isoform a [Homo sapiens]	53689.81686	62362.92806	64880.13205	29662.26913	35219.66473	60763.44126	68213.22057	84475.39481	52564.23309
MARS	methionine--tRNA ligase, cytoplasmic [Homo sapiens]	1959474.448	2425672.52	2691677.39	2416553.48	3068094.15	2632502.825	2206615.045	2223377.561	2360659.585
MARS2	methionine--tRNA ligase, mitochondrial precursor [Homo sapiens]	114915.8548	86189.81923	97934.59029	116600.962	166077.3284	57020.66367	106546.7657	90573.59362	34176.05374
MASTL	serine/threonine-protein kinase greatwall isoform 1 [Homo sapiens]	26969.13718	46592.47484	0	55242.80276	0	100621.2662	85770.30318	92423.33279	64597.49188
MAT2A	S-adenosylmethionine synthase isoform type-2 [Homo sapiens]	8201635.928	10138553.45	10716404.08	9841282.908	10818244.66	10179459.49	10101899.91	8559028.388	10715112.45
MAT2B	methionine adenosyltransferase 2 subunit beta isoform 1 [Homo sapiens]	394242.4154	2282730.1	2639678.122	2315144.267	2514944.81	2315570.214	2235367.027	1762124.044	2560850.779
MATR3	matrin-3 isoform a [Homo sapiens]	5747915.623	6027165.167	5723114.289	5662954.433	7980602.547	6796895.705	4340660.348	5008102.839	4547658.126
MAU2	MAU2 chromatid cohesion factor homolog [Homo sapiens]	117102.8658	0	0	136506.8633	42357.15678	104613.8531	0	0	0
MAVS	mitochondrial antiviral-signaling protein isoform 1 [Homo sapiens]	0	344653.9523	241705.6729	245253.7997	485976.4658	234535.1872	0	0	0
MAX	protein max isoform b [Homo sapiens]	301575.0696	277075.6585	239115.9472	325774.9349	302580.3451	358289.1294	232664.6183	0	337344.2289
MAZ	myc-associated zinc finger protein isoform 2 [Homo sapiens]	117542.1343	141771.1176	0	116521.1786	0	130248.4375	127006.9449	98541.74484	81515.94621
MB21D1	cyclic GMP-AMP synthase [Homo sapiens]	444259.4617	95856.6062	563137.715	406331.9263	604740.2883	160774.7096	183711.9461	374656.4815	561991.4549
MBD2	methyl-CpG-binding domain protein 2 isoform 1 [Homo sapiens]	156550.6314	218404.4261	0	106592.9276	387039.0194	156868.1343	347191.0559	426629.0956	235802.8807
MBD3	methyl-CpG-binding domain protein 3 [Homo sapiens]	712883.1334	901775.7168	847326.156	961940.8464	887547.9336	984790.5064	871389.172	836164.1064	913702.8744
MBD4	methyl-CpG-binding domain protein 4 isoform 1 [Homo sapiens]	26169.62108	0	96026.07927	21286.02439	72133.83101	43080.3485	32779.93946	0	81565.93902
MBLAC2	metallo-beta-lactamase domain-containing protein 2 [Homo sapiens]	103630.8243	217520.6464	185342.8649	184198.1014	212448.6379	240466.3359	150704.2831	162610.1261	213240.4887
MBNL1	muscleblind-like protein 1 isoform c [Homo sapiens]	1007727.734	281245.0236	795185.2614	900124.9974	1362916.771	968805.8168	811116.5746	809441.7913	687025.8685
MBNL1	muscleblind-like protein 1 isoform a [Homo sapiens]	1007727.734	281245.0236	795185.2614	900124.9974	1362916.771	968805.8168	811116.5746	809441.7913	687025.8685
MBNL2	muscleblind-like protein 2 isoform 1 [Homo sapiens]	327331.682	0	413765.46	362510.1238	362672.1869	440411.9879	398309.2257	0	313982.6845
MBNL3	muscleblind-like protein 3 isoform 1 [Homo sapiens]	467304.1168	511241.9884	88661.73585	419860.3739	0	507572.6669	259330.1772	49684.77977	307651.5315
MBOAT7	lysophospholipid acyltransferase 7 isoform 1 [Homo sapiens]	1166533.489	1098111.181	1316747.25	909612.1184	1599648.959	1114388.274	901684.2149	628151.3382	1110536.558
MCAM	cell surface glycoprotein MUC18 precursor [Homo sapiens]	234387.7639	331359.731	281055.9694	364201.8622	297512.8478	378583.3252	248890.6067	202219.5086	344342.2187
MCAT	malonyl-CoA-acyl carrier protein transacylase, mitochondrial isoform a prec	149462.5245	458094.4073	193216.3322	309068.0012	0	117106.3011	86279.24301	72651.59007	490632.2063
MCCC1	methylcrotonoyl-CoA carboxylase subunit alpha, mitochondrial precursor [Hom	0	108099.3402	0	109943.2773	63292.7875	111479.5518	117429.2857	55919.99532	90039.28811
MCCC2	methylcrotonoyl-CoA carboxylase beta chain, mitochondrial [Homo sapiens]	427732.2633	527124.2048	264883.2065	555151.5483	482555.0785	501396.6836	537212.9739	374063.8313	591709.5488
MCFD2	multiple coagulation factor deficiency protein 2 isoform A precursor [Homo	4178647.33	4749293.462	4958739.605	3268983.897	3134435.897	5042620.57	4393852.27	5820448.759	6167201.419
MCM2	DNA replication licensing factor MCM2 [Homo sapiens]	2118382.756	2464383.346	2849399.456	2512246.363	2991414.539	2700647.329	2538991.649	2103566.306	2667971.36
MCM3	DNA replication licensing factor MCM3 isoform 1 [Homo sapiens]	2599714.016	3239970.879	3549988.997	3146345.455	3618511.288	3292198.686	3231215.119	2558196.262	3583416.559
MCM3AP	80 kDa MCM3-associated protein [Homo sapiens]	13938.75965	16424.56085	19627.83563	20850.85966	24510.36547	15823.58912	0	0	0
MCM4	DNA replication licensing factor MCM4 [Homo sapiens]	2686427.874	2865985.773	3141114.813	2605566.972	3094278.845	2734646.743	2892716.752	2568543.533	2951895.504
MCM5	DNA replication licensing factor MCM5 [Homo sapiens]	3109558.468	3362404.912	4069759.665	3460792.609	4170664.168	3611272.809	3441766.974	2860343.978	3398294.741
MCM6	DNA replication licensing factor MCM6 [Homo sapiens]	2303538.039	2486064.276	2792950.151	2569813.605	3037183.165	2650912.848	2523518.48	2126522.734	2588186.652
MCM7	DNA replication licensing factor MCM7 isoform 1 [Homo sapiens]	3645889.438	3718818.5	4503638.656	3994933.247	4436348.13	4083030.838	3832685.221	2792313.853	3863896.299
MCMBP	mini-chromosome maintenance complex-binding protein isoform 1 [Homo sapiens	441168.0549	107241.45	550870.1776	530848.4706	602974.7505	0	535912.5575	471711.5584	495767.4336
MCTP2	multiple C2 and transmembrane domain-containing protein 2 isoform 1 [Homo s	39265.2137	56955.1884	59869.26361	30418.92716	65517.75645	50156.63372	42170.26971	42638.05814	43525.25622
MCTS1	malignant T-cell-amplified sequence 1 isoform 2 [Homo sapiens]	3855680.469	3433042.268	3273955.706	4238424.315	4656749.674	4556509.001	4062323.363	3596835.357	5964369.564
MCU	calcium uniporter protein, mitochondrial isoform 1 precursor [Homo sapiens]	645186.8656	530653.3891	812949.7697	668528.4369	829595.9493	576566.2171	637835.0594	462856.0499	641663.1517
MCUR1	mitochondrial calcium uniporter regulator 1 [Homo sapiens]	79963.43337	94150.98217	0	51343.66643	0	56460.91286	89552.77429	85499.80823	0
MDC1	mediator of DNA damage checkpoint protein 1 [Homo sapiens]	134824.1617	115093.9021	169913.932	120964.3692	162870.882	137107.9028	118132.9515	91444.11742	90838.75995
MDH1	malate dehydrogenase, cytoplasmic isoform 1 [Homo sapiens]	9654811.795	11264088.77	11978931.15	10968614.17	13911265.96	11024579.77	11618282.08	9502872.692	12171559.73
MDH2	malate dehydrogenase, mitochondrial precursor [Homo sapiens]	19590633.97	20437140.7	26514750.24	22007985.62	25458518.41	23187383.22	21872845.96	18973133.46	21997471.54
MDN1	midasin [Homo sapiens]	24504.58444	24287.90799	33618.96145	28529.95386	35007.30547	29165.21367	26853.32152	28770.73459	25854.50521
ME1	NADP-dependent malic enzyme [Homo sapiens]	595626.7082	660969.5154	743543.5043	753677.7346	841748.1986	808512.874	753306.812	611572.9288	714372.1106
ME2	NAD-dependent malic enzyme, mitochondrial isoform 1 precursor [Homo sapiens	483495.0197	525291.3798	681002.2821	532538.3225	807340.8317	542005.2284	495977.5459	488662.8729	539872.7821
MEA1	male-enhanced antigen 1 [Homo sapiens]	621021.5956	3619276.706	3309501.835	3318593.706	4007394.647	3493438.332	3273288.647	2671781.007	3571540.353
MEAF6	chromatin modification-related protein MEAF6 isoform 1 [Homo sapiens]	566378.1729	776076.2048	429630.4	619282.1789	1390776.038	579249.5248	694115.4695	526851.9323	316818.3656
MECP2	methyl-CpG-binding protein 2 isoform 2 [Homo sapiens]	77341.32089	86581.67339	73817.25101	152236.2833	34470.29637	101196.4382	102539.9993	47455.67597	117304.8367
MED1	mediator of RNA polymerase II transcription subunit 1 [Homo sapiens]	51564.70539	67869.67012	67339.68867	45731.39588	58657.39443	60701.80665	60398.40852	37243.96744	44479.43397
MED10	mediator of RNA polymerase II transcription subunit 10 [Homo sapiens]	0	0	0	0	0	0	329129.8347	0	124814.7944
MED11	mediator of RNA polymerase II transcription subunit 11 [Homo sapiens]	961892.2996	838590.4129	1268166.946	926691.5429	885065.6694	970251.5196	882825.1416	652193.5735	205717.1545
MED12	mediator of RNA polymerase II transcription subunit 12 [Homo sapiens]	30117.23745	7587.805962	48120.46801	15413.3184	48718.32148	10077.45077	3443.148884	0	10826.95031
MED14	mediator of RNA polymerase II transcription subunit 14 [Homo sapiens]	0	51540.51643	49344.17402	51517.61867	80569.42402	52475.4171	54156.46881	30220.93571	72313.30959
MED15	mediator of RNA polymerase II transcription subunit 15 isoform a [Homo sap:	205327.2641	340054.5508	441940.7207	366384.7678	393637.9768	376801.7418	356890.9023	390334.2545	212733.2368
MED17	mediator of RNA polymerase II transcription subunit 17 [Homo sapiens]	69247.2102	74609.79796	48671.11633	51479.09984	42362.92808	82671.28351	54815.34453	62067.88061	65010.746
MED20	mediator of RNA polymerase II transcription subunit 20 [Homo sapiens]	728358.3952	591694.3306	1275575.46	611883.7944	1105461.032	619286.2823	689200.121	673141.8024	0
MED21	mediator of RNA polymerase II transcription subunit 21 isoform 1 [Homo sap:	411056.07	478130.5774	573178.9191	368959.3126	514231.5588	469570.4229	447798.4609	400800.4762	511954.7482
MED23	mediator of RNA polymerase II transcription subunit 23 isoform a [Homo sap:	20015.54546	43229.7382	55705.71724	38743.42463	37968.39589	46395.06663	29793.83265	34130.25449	24555.39719
MED24	mediator of RNA polymerase II transcription subunit 24 isoform 1 [Homo sap:	58584.92183	72808.71941	74980.07487	72638.16422	52400.93106	90817.58572	0	0	0
MED25	mediator of RNA polymerase II transcription subunit 25 [Homo sapiens]	102558.3394	21536.76894	0	211991.2356	240604.0421	328069.2333	0	0	0
MED27	mediator of RNA polymerase II transcription subunit 27 isoform 1 [Homo sap:	265141.4681	213054.6705	158501.71	135616.5694	191270.4939	185091.5271	81685.19721	154753.7413	622244.4341
MED28	mediator of RNA polymerase II transcription subunit 28 [Homo sapiens]	1006823.435	547905.1153	0	853983.5302	1149484.414	525934.6186	163683.1181	917832.0334	306889.8083
MED30	mediator of RNA polymerase II transcription subunit 30 [Homo sapiens]	442639.1217	647572.7155	357194.2895	309878.6192	356596.4836	305503.7632	301943.4145	350918.1678	700087.5263
MED31	mediator of RNA polymerase II transcription subunit 31 [Homo sapiens]	200820.6067	573092.2115	0	345478.8558	545479.5913	0	211580.8292	0	409214.4615
MED4	mediator of RNA polymerase II transcription subunit 4 isoform 1 [Homo sapie	0	0	0	0	0	0	0	512890.5561	428594.979
MED8	mediator of RNA polymerase II transcription subunit 8 isoform 3 [Homo sapie	168730.9806	233462.5984	203938.9197	174638.4783	217551.27	210540.4216	174373.8901	128261.8981	0
MEF2BNB	protein MEF2BNB isoform 1 [Homo sapiens]	339178.2973	0	460909.4124	0	503807.3958	327272.266	300695.7722	0	289052.7569
MEF2D	myocyte-specific enhancer factor 2D isoform 1 [Homo sapiens]	225280.4876	283552.5996	323452.5426	308857.2676	208314.7496	265908.394	262708.4221	193586.8179	323528.3126
MEMO1	protein MEMO1 isoform 1 [Homo sapiens]	919490.8767	1002663.227	1093095.138	1068607.497	1238051.104	1177501.862	980109.6121	893697.2392	1145207.357
MEN1	menin isoform 1 [Homo sapiens]	127124.9978	136734.2399	207976.076	171475.3376	180343.0599	43441.79109	193606.5248	0	122725.8583
MEPCE	7SK snRNA methylphosphate capping enzyme isoform A [Homo sapiens]	156809.2624	1523562.07	200724.5443	201889.3601	222849.4018	180936.9778	172343.1338	107653.6404	1344416.751
MESDC2	LDLR chaperone MESD precursor [Homo sapiens]	1436381.941	1438982.843	1493982.992	1610792.829	1517273.188	1639641.504	1468668.585	1094810.433	1609824.25
METAP1	methionine aminopeptidase 1 [Homo sapiens]	1329973.05	1299284.101	1947049.103	1270945.155	1905298.399	1342113.865	1306087.545	1085720.698	1728006.422
METAP2	methionine aminopeptidase 2 [Homo sapiens]	1483781.523	2092957.645	2100082.951	1545380.003	2194295.505	1632701.97	1522210.433	1585129.503	2013770.452
METTL1	tRNA (guanine-N(7)-)-methyltransferase isoform a [Homo sapiens]	363357.734	331972.6641	608114.2525	392474.4009	429601.8687	391206.5699	271425.2896	628673.5332	766675.507
METTL13	methyltransferase-like protein 13 isoform 1 [Homo sapiens]	169182.356	212006.8194	252081.634	395811.2615	919143.0815	210631.4649	179518.0772	155050.7025	126522.488
METTL14	methyltransferase-like protein 14 [Homo sapiens]	0	0	0	0	0	0	120660.8058	0	127640.8835
METTL15	probable methyltransferase-like protein 15 isoform 1 [Homo sapiens]	0	140715.9779	145644.4033	77312.791	76322.17106	93002.54535	85711.123	126260.8498	97234.28041
METTL16	methyltransferase-like protein 16 [Homo sapiens]	67032.2428	142415.6416	138846.8415	253882.8707	209715.9551	158539.1236	114709.0197	160819.157	200172.8863
METTL2A	methyltransferase-like protein 2A [Homo sapiens]	0	0	0	0	0	0	160463.3456	115030.2779	0
METTL2B	methyltransferase-like protein 2B [Homo sapiens]	229530.2254	224993.9178	341439.5618	276031.2727	352497.7209	262312.5727	0	0	0
METTL3	N6-adenosine-methyltransferase 70 kDa subunit [Homo sapiens]	26433.835	151856.581	65569.90046	37759.25463	103788.406	72808.73764	72215.625	121051.588	138673.7869
METTL5	methyltransferase-like protein 5 [Homo sapiens]	216105.5979	570301.2906	283526.4464	200599.7798	0	224111.7496	422365.6567	0	707922.6012
METTL7A	methyltransferase-like protein 7A precursor [Homo sapiens]	2370714.131	2989613.015	3324097.155	2617464.24	3716469.131	2179814.138	2468995.238	2963807.132	2197567.957
METTL7B	methyltransferase-like protein 7B precursor [Homo sapiens]	358545.4861	0	353462.3065	291482.0595	377492.8349	381581.6831	364634.3724	330500.4874	365671.4792
METTL9	methyltransferase-like protein 9 isoform 1 precursor [Homo sapiens]	0	90458.30804	149347.1987	165496.2277	71738.47879	112603.6188	100213.7534	116378.0067	126014.5804
MFAP1	microfibrillar-associated protein 1 [Homo sapiens]	331910.5741	221145.9077	402043.1935	373270.521	440821.3339	535006.5517	365090.2225	214962.9207	198433.2445
MFF	mitochondrial fission factor isoform b [Homo sapiens]	495298.8562	484808.259	634630.5192	531306.871	721916.153	498627.6081	442882.8164	371498.9849	0
MFN1	mitofusin-1 [Homo sapiens]	62501.04556	54290.50493	112322.9729	141847.4984	107343.6682	0	0	78000.02056	58270.05921
MFN2	mitofusin-2 [Homo sapiens]	169226.0351	182925.2744	191435.448	162083.561	241005.0039	184579.8043	212352.4002	143358.4769	139166.1706
MFSD1	major facilitator superfamily domain-containing protein 1 isoform 1 [Homo s	746129.2609	741478.687	974058.2522	715569.7435	1121937.452	796034.087	646245.8826	517616.8043	0
MFSD10	major facilitator superfamily domain-containing protein 10 [Homo sapiens]	381590.5725	452470.9291	580828.7139	413530.9874	599320.0238	481959.2139	514549.4554	458193.6828	557495.5683
MFSD12	major facilitator superfamily domain-containing protein 12 isoform a [Homo	188821.1927	241825.7524	503040.4695	318988.3239	571596.2926	420051.2033	254074.4135	151219.3025	184160.4504
MGAT1	alpha-1,3-mannosyl-glycoprotein 2-beta-N-acetylglucosaminyltransferase [Hom	0	0	0	0	0	0	36371.8582	112657.3333	0
MGAT2	alpha-1,6-mannosyl-glycoprotein 2-beta-N-acetylglucosaminyltransferase [Hom	218293.7148	177578.1121	0	72766.50718	57665.91201	251480.3822	106698.1376	83569.31224	120168.7955
MGEA5	bifunctional protein NCOAT isoform a [Homo sapiens]	520448.8585	497529.6104	657586.1119	573897.0286	606402.5459	622031.5654	621853.7868	528334.3748	309110.3664
MGLL	monoglyceride lipase isoform 1 [Homo sapiens]	1206583.451	1177135.001	1742697.545	1154090.627	1390990.799	1115862.606	1136998.599	1175078.73	1407162.993
MGME1	mitochondrial genome maintenance exonuclease 1 [Homo sapiens]	0	0	0	0	0	0	80439.34107	0	175931.0304
MGMT	methylated-DNA--protein-cysteine methyltransferase [Homo sapiens]	500205.8072	524371.3193	370518.7078	650115.7741	551127.7389	583979.9518	352965.3498	325371.1649	268608.9398
MGST1	microsomal glutathione S-transferase 1 isoform a [Homo sapiens]	2839744.881	6927575.534	7251115.855	6590471.274	4141176.989	6732263.594	6311565.579	1994371.234	7694550.221

MGST2	microsomal glutathione S-transferase 2 isoform 1 precursor [Homo sapiens]	4890681.165	6029383.125	4061464.043	4780974.975	4596110.3	6311717.45	5651464.858	4284368.075	4161841.22
MGST3	microsomal glutathione S-transferase 3 [Homo sapiens]	9955325.217	8455269.138	10600922.57	7886305.976	11873157.17	9538243.345	10153025.41	7598681.048	9446267.81
MIA3	melanoma inhibitory activity protein 3 precursor [Homo sapiens]	34465.02537	33308.46568	141603.3482	41991.16087	157021.9323	43688.11385	39051.68911	39460.37287	36594.0713
MICA	MHC class I polypeptide-related sequence A isoform 2 (MICA*00801) precursor	174635.9634	227233.3356	0	125298.7823	0	175993.8671	226158.5833	199091.5278	223232.1341
MICAL3	protein-methionine sulfoxide oxidase MICAL3 isoform 1 [Homo sapiens]	29915.77494	194495.7195	16076.78727	33115.26937	61334.94102	23649.67131	31516.77406	37717.25	0
MICALL1	MICAL-like protein 1 [Homo sapiens]	85199.28474	29225.86838	22965.43707	36160.00662	30471.51029	45112.11556	29737.55744	36894.22291	50495.28979
MICU1	calcium uptake protein 1, mitochondrial isoform 1 [Homo sapiens]	210710.4605	211467.7397	160167.6651	283247.9805	303170.1598	262750.9719	157280.5275	96007.52179	210358.7306
MICU2	calcium uptake protein 2, mitochondrial [Homo sapiens]	272864.071	300525.2072	359328.9224	353489.3861	385217.9878	403773.0917	199318.2363	169691.0104	334435.0247
MID1	midline-1 isoform 1 [Homo sapiens]	0	17398.37185	72163.66231	0	47028.15718	21433.15494	0	0	0
MID1IP1	mid1-interacting protein 1 [Homo sapiens]	614050.9523	2871644.655	2162551.911	706175.6614	1400387.367	3920102.854	741397.4136	684569.0804	4528685.425
MIEN1	migration and invasion enhancer 1 [Homo sapiens]	1641801.598	1234041.429	1950515.857	1441366.697	779483.8929	2333097.086	1936124.729	1034358.956	974949.8857
MIER1	mesoderm induction early response protein 1 isoform b [Homo sapiens]	62317.82682	29897.96875	44880.70247	0	53433.11719	0	82873.03646	23858.67708	162099.2214
MIF	macrophage migration inhibitory factor [Homo sapiens]	248871197.5	280736618.9	310147641	315872991.9	287582999.8	329455046.3	333080836.5	280460211	315603009.9
MINA	bifunctional lysine-specific demethylase and histidyl-hydroxylase MINA isoi	258659.8909	218966.2249	387052.467	214655.5984	341734.769	489611.964	255522.9688	209353.023	252243.4885
MINPP1	multiple inositol polyphosphate phosphatase 1 isoform 1 precursor [Homo sa	0	0	0	0	0	0	538928.907	101359.6702	80167.7722
MIPEP	mitochondrial intermediate peptidase [Homo sapiens]	36215.10776	136904.7121	132684.9523	210407.1724	106530.2511	222955.6345	113848.6519	156437.2371	56070.08103
MISP	uncharacterized protein C19orf21 [Homo sapiens]	1124388.879	1422279.397	1135024.798	1368935.525	1691586.252	1451121.727	1169833.787	963390.6835	1211630.699
MITF	microphthalmia-associated transcription factor isoform 1 [Homo sapiens]	0	0	0	0	0	0	0	0	0
MKI67	antigen KI-67 isoform 1 [Homo sapiens]	120513.6539	264906.2358	147492.5357	286190.2404	145908.8359	153610.9888	144483.4842	125640.668	155077.2174
MKI67IP	MKI67 FHA domain-interacting nucleolar phosphoprotein [Homo sapiens]	1367217.718	2268283.796	2438632.343	1814667.884	2106282.726	1940230.104	1585830.984	1322149.055	2087843.388
MKL1	MKL/myocardin-like protein 1 [Homo sapiens]	0	81856.7351	34198.94273	126089.1518	70725.67	26189.6537	156462.7546	80271.85677	5387.173397
MKL2	MKL/myocardin-like protein 2 [Homo sapiens]	60083.24835	52117.14653	0	32784.80827	0	46085.83419	32299.12356	40838.61168	20559.65104
MKLN1	muskelin isoform 2 [Homo sapiens]	14494.77216	48864.50147	70332.41137	77339.89967	79534.28054	22458.88671	53181.40662	74373.41806	90066.3208
MLEC	malectin precursor [Homo sapiens]	1000098.576	1283359.693	672019.6504	2292901.83	1417022.94	2589611.774	2555842.258	1887552.005	880909.6281
MLF2	myeloid leukemia factor 2 [Homo sapiens]	571699.758	549963.7739	0	543458.3085	744634.0559	410429.8351	380226.8404	360695.1011	260071.8711
MLH1	DNA mismatch repair protein Mlh1 isoform 1 [Homo sapiens]	199235.3807	129506.386	285885.0888	166302.132	253512.8266	254401.6175	184788.4832	37495.5637	79420.16497
MLKL	mixed lineage kinase domain-like protein isoform 1 [Homo sapiens]	257732.91	353319.3942	438817.371	351225.0584	493441.9908	369246.976	354325.8046	235565.3304	906647.1618
MLL	histone-lysine N-methyltransferase MLL isoform 1 precursor [Homo sapiens]	0	0	0	0	0	0	1352.978363	10552.49791	8320.135962
MLL2	histone-lysine N-methyltransferase MLL2 [Homo sapiens]	5070.974903	118254.6885	6388.055538	4883.482236	8958.614637	3152.214044	1332.984254	0	6911.879139
MLL4	histone-lysine N-methyltransferase MLL4 [Homo sapiens]	4513.847301	0	7909.85465	5105.666401	5858.681611	438908.7596	3475.898132	0	5897.358606
MLLT1	protein ENL [Homo sapiens]	56146.93703	145973.8164	98063.12406	106811.1687	203078.9408	99950.88252	174944.9236	210871.5912	52903.35985
MLLT10	protein AF-10 isoform c [Homo sapiens]	17678.43947	27159.0315	0	16169.16683	42729.19291	0	0	41349.65701	15521.60925
MLLT4	afadin isoform 1 [Homo sapiens]	0	0	0	0	0	0	0	0	0
MLST8	target of rapamycin complex subunit LST8 isoform a [Homo sapiens]	224923.905	112862.454	373557.1243	208142.3381	385637.343	254041.9329	204836.2524	274045.9148	248748.4844
MLYCD	malonyl-CoA decarboxylase, mitochondrial [Homo sapiens]	0	0	0	0	0	0	19271.8566	0	565023.3939
MMAB	cob(I)yrinic acid a,c-diamide adenosyltransferase, mitochondrial precursor	264026.5012	0	607951.6075	483151.972	639228.25	163148.3937	0	0	0
MMADHC	methylmalonic aciduria and homocystinuria type D protein, mitochondrial [Ho	69510.43085	120647.8628	0	151066.6822	0	122336.8711	0	0	0
MMGT1	membrane magnesium transporter 1 precursor [Homo sapiens]	1616821.406	1486974	2044811.021	1569705.906	2150535.229	1586170.75	1872845.362	1448239.677	0
MMS19	MMS19 nucleotide excision repair protein homolog [Homo sapiens]	380911.7591	419641.2347	489720.5091	418221.4721	501179.3256	402541.9279	411726.8955	370155.7422	449972.1815
MNAT1	CDK-activating kinase assembly factor MAT1 isoform 1 [Homo sapiens]	318998.5502	415604.406	906816.5	210563.6917	290257.8243	417013.7882	434916.8741	159691.6614	370246.5902
MNF1	mitochondrial nucleoid factor 1 [Homo sapiens]	694164.2693	0	573654.6691	566185.4881	0	718889.7794	485795.0037	583783.6516	0
MOB1A	MOB kinase activator 1A [Homo sapiens]	2042189.696	0	3107674.712	2275186.137	3354439.562	2442736.026	2229016.327	0	1682612.875
MOB1B	MOB kinase activator 1B isoform 1 [Homo sapiens]	0	0	0	0	0	0	0	0	0
MOB4	MOB-like protein phocein isoform 1 [Homo sapiens]	1086275.603	773720.8694	1293133.01	199327.179	1079777.47	0	1246257.531	583135.2554	981171.8806
MOCOS	molybdenum cofactor sulfurase [Homo sapiens]	473023.1278	179133.5631	318454.1451	548539.4	363342.3789	270787.8873	222204.4787	178971.1102	238549.9346
MOCS2	molybdopterin synthase catalytic subunit small subunit MOCS2A [Homo sapiens	941819.9379	230646.0609	1217412.683	944497.153	909907.2603	165587.7373	1056096.637	162374.3315	946116.9111
MOCS2	molybdopterin synthase catalytic subunit large subunit MOCS2B [Homo sapiens	941819.9379	230646.0609	1217412.683	944497.153	909907.2603	165587.7373	1056096.637	162374.3315	946116.9111
MOGS	mannosyl-oligosaccharide glucosidase isoform 1 [Homo sapiens]	512650.3823	500043.1279	835969.7823	514727.7608	806892.1615	589271.9088	618583.9633	451299.8272	597868.0848
MON2	protein MON2 homolog isoform 1 [Homo sapiens]	123395.6258	76023.90933	0	209568.8211	99241.07814	0	0	165260.4927	95022.57056
MORC2	MORC family CW-type zinc finger protein 2 [Homo sapiens]	32167.71308	41259.36979	66901.45208	31095.46758	66653.99115	41100.354	43914.10365	37676.62708	43601.39896
MORC3	MORC family CW-type zinc finger protein 3 [Homo sapiens]	64497.36297	64818.63251	90567.1618	69229.32031	128887.2174	60869.16792	73788.51383	50759.51432	65770.96398
MORF4L1	mortality factor 4-like protein 1 isoform 1 [Homo sapiens]	0	1258248.49	1356900.106	1234771.766	1303245.177	1304892.85	835225.9213	683179.3942	0
MORF4L2	mortality factor 4-like protein 2 [Homo sapiens]	882008.1214	718783.2557	781555.02	1198431.14	730543.0664	618024.1244	603867.4536	839381.9221	729299.2486
MOSPD2	motile sperm domain-containing protein 2 isoform 1 [Homo sapiens]	201073.4651	182201.481	237552.989	152756.7164	279458.1256	234682.2047	221033.0066	161069.6612	239752.7364
MOV10	putative helicase MOV-10 [Homo sapiens]	224267.8147	270267.2541	272555.9482	248981.0619	278018.4682	278726.5814	244481.1122	185827.2808	202163.4996
MPC1	mitochondrial pyruvate carrier 1 isoform 1 [Homo sapiens]	966927.7647	0	1197405.824	0	1230071.34	1139325.906	1014025.457	644558.2155	0
MPC2	mitochondrial pyruvate carrier 2 [Homo sapiens]	383193.3238	581254.6309	1205862.726	728859.067	1147415.848	730659.0893	588607.6496	0	122295.0818
MPDU1	mannose-P-dolichol utilization defect 1 protein [Homo sapiens]	3900654.764	3700976.904	7831362.149	3980537.708	8166577.326	4156082.987	4477240.417	4131777.413	5949730.289
MPG	DNA-3-methyladenine glycosylase isoform a [Homo sapiens]	331722.0071	306749.8499	501500.813	345951.9302	521481.5975	375320.2535	277865.3853	332801.634	274429.5474
MPHOSPH10	U3 small nucleolar ribonucleoprotein protein MPP10 [Homo sapiens]	375237.0751	369126.7276	431477.7081	357510.0966	467967.3862	414985.8195	406715.4842	298299.1818	353857.3461
MPHOSPH6	M-phase phosphoprotein 6 [Homo sapiens]	0	0	0	0	0	0	686272.716	599168.2716	283443.4444
MPHOSPH8	M-phase phosphoprotein 8 [Homo sapiens]	18117.37357	110505.846	49905.11509	38466.76394	46593.79433	13791.09073	50740.49543	62574.3242	57342.24381
MPI	mannose-6-phosphate isomerase [Homo sapiens]	588137.0553	869189.4477	1361615.613	1162339.555	1322490.369	1137274.68	954552.4867	599362.819	711941.7241
MPLKIP	M-phase-specific PLK1-interacting protein [Homo sapiens]	437648.7997	272112.3839	493332.0969	386567.1156	977921.7664	395460.5779	245532.8041	0	271924.1875
MPP1	55 kDa erythrocyte membrane protein isoform 1 [Homo sapiens]	254342.2522	236012.5159	405795.245	241309.6073	686504.1376	318084.3276	70883.57803	0	176715.0578
MPP6	MAGUK p55 subfamily member 6 [Homo sapiens]	103061.5748	146441.433	135449.3651	175676.0915	154046.8724	128230.5864	213617.983	151781.1381	237910.4986
MPRIP	myosin phosphatase Rho-interacting protein isoform 1 [Homo sapiens]	141482.0508	317826.4805	266578.9456	242995.2409	319117.1822	243660.6983	305999.478	339156.9371	172921.3284
MPST	3-mercaptopyruvate sulfurtransferase isoform 1 [Homo sapiens]	2628400.796	2024541.539	2443325.41	1568658.222	2331771.902	2668689.929	2123041.539	2410686.239	2303390.836
MPV17	protein Mpv17 [Homo sapiens]	1653806.386	1564707.39	2073053.9	455460.1798	2185967.816	1557378.318	1545558.068	1352366.418	1399787.259
MPV17L2	mpv17-like protein 2 precursor [Homo sapiens]	386679.3229	0	744494.6806	478590.9861	739186.8333	618905.5972	0	0	0
MPZL1	myelin protein zero-like protein 1 isoform a precursor [Homo sapiens]	2485170.922	2134710.294	3100580.528	2011575.87	3151042.632	2584317.871	2107015.7	1647864.87	2811823.441
MPZL2	myelin protein zero-like protein 2 precursor [Homo sapiens]	526864.0513	0	0	637228.4568	673722.5385	703010.5594	485632.1955	277632.8237	0
MRC2	C-type mannose receptor 2 precursor [Homo sapiens]	31750.55114	62670.21598	49684.58239	67481.54432	123065.2636	74613.66761	45696.7875	34171.76591	41562.89461
MRE11A	double-strand break repair protein MRE11A isoform 1 [Homo sapiens]	695563.6654	395297.6315	430811.2197	373726.679	445198.9763	374826.4831	220161.6304	309117.5949	307264.0322
MRFAP1	MORF4 family-associated protein 1 isoform a [Homo sapiens]	1574209.666	3441007.99	3010161.586	3751203.031	4411447.98	3859441.404	2319425.645	2057931.6	3061755.237
MRGBP	MRG/MORF4L-binding protein [Homo sapiens]	581144.4114	797134.4058	0	0	532374.8608	535027.7405	0	89028.89241	589457.3924
MRI1	methylthioribose-1-phosphate isomerase isoform 1 [Homo sapiens]	1339173.44	1750085.597	1805304.168	1832918.793	1770650.008	1829908.389	1823111.028	1422998.663	1455044.912
MRM1	rRNA methyltransferase 1, mitochondrial precursor [Homo sapiens]	0	0	0	0	0	0	262497.187	22696.69705	0
MRP63	ribosomal protein 63, mitochondrial [Homo sapiens]	0	0	0	0	0	0	0	0	0
MRPL1	39S ribosomal protein L1, mitochondrial precursor [Homo sapiens]	204952.0626	297358.1801	213636.0369	211679.2646	212214.4558	927868.0389	776788.7718	161549.118	1156138.402
MRPL10	39S ribosomal protein L10, mitochondrial isoform b [Homo sapiens]	901315.3825	775874.6725	1388602.29	886654.1992	1260690.814	1042157.273	889965.3358	740394.925	960456.5963
MRPL11	39S ribosomal protein L11, mitochondrial isoform a [Homo sapiens]	756614.505	794036.75	1230229.936	1708995.372	2561772.02	1555141.927	972367.6179	690873.3933	1530055.846
MRPL12	39S ribosomal protein L12, mitochondrial [Homo sapiens]	7171301.537	7819810.058	11560986.97	8446792.277	10785847.5	7805728.713	8667307.358	8350499.492	8994292.265
MRPL13	39S ribosomal protein L13, mitochondrial [Homo sapiens]	734954.3739	870279.0861	939390.6147	859175.264	948941.3874	1069963.694	936366.3691	452774.9083	888340.6753
MRPL14	39S ribosomal protein L14, mitochondrial [Homo sapiens]	1418114.28	1393816.885	1686936.811	1532533.592	1861633.32	1537651.135	1418652.189	960196.6486	1414575.648
MRPL15	39S ribosomal protein L15, mitochondrial [Homo sapiens]	444826.3473	438917.544	496036.4755	379244.6684	510212.3502	413478.6183	574882.1334	354252.4214	409948.6105
MRPL16	39S ribosomal protein L16, mitochondrial [Homo sapiens]	239645.6748	324473.2596	327440.772	214477.6059	718315.3224	629575.5728	250380.0375	198524.3961	218453.75
MRPL17	39S ribosomal protein L17, mitochondrial [Homo sapiens]	542930.2343	1106034.43	1020862.178	633957.3972	1010471.38	608815.1748	976903.5836	628021.0878	882638.9828
MRPL18	39S ribosomal protein L18, mitochondrial [Homo sapiens]	695517.4173	1072501.625	403332.0454	872882.1594	254932.2048	935872.3516	863901.7961	761578.6235	941562.65
MRPL19	39S ribosomal protein L19, mitochondrial [Homo sapiens]	465300.3328	619753.5238	380234.7388	514044.7103	507614.9063	378239.0741	351192.0339	325223.4682	475128.2679
MRPL2	39S ribosomal protein L2, mitochondrial [Homo sapiens]	848372.7401	398097.5782	450899.0595	526972.1071	200201.781	1168084.814	978377.4524	800996.483	553794.271
MRPL20	39S ribosomal protein L20, mitochondrial [Homo sapiens]	397091.0632	0	396154.8722	687099.3908	547772.1178	0	0	0	0
MRPL21	39S ribosomal protein L21, mitochondrial isoform d [Homo sapiens]	533113.696	1018333.552	457283.9444	1278823.025	917393.892	1654460.726	1483364.148	487076.5779	1472920.689
MRPL22	39S ribosomal protein L22, mitochondrial isoform a [Homo sapiens]	693454.6643	585021.8463	1062072.44	953342.2816	1008878.698	930279.7573	766571.0874	443790.0704	870734.6262
MRPL23	39S ribosomal protein L23, mitochondrial [Homo sapiens]	1732650.338	0	149191.6866	2233172.258	0	2409663.035	1873883.887	1758009.986	1586805.079
MRPL24	39S ribosomal protein L24, mitochondrial [Homo sapiens]	437240.5982	79207.47266	598061.6596	214900.5714	776022.2824	340026.8432	287516.4212	403011.3013	130313.3745
MRPL27	39S ribosomal protein L27, mitochondrial [Homo sapiens]	585566104.8	1392226.34	687389881.4	548389828.6	676460330.9	487281268.9	226978307.8	655896.0205	556656050.4
MRPL28	39S ribosomal protein L28, mitochondrial [Homo sapiens]	259774.1901	579178.8941	585156.5332	621134.0539	570452.7168	643965.9824	465468.9198	468152.0085	595250.6016
MRPL3	39S ribosomal protein L3, mitochondrial [Homo sapiens]	379212.7681	591345.1459	660178.8673	688914.6765	566865.793	508369.2422	447969.3219	351890.362	524385.9085
MRPL32	39S ribosomal protein L32, mitochondrial precursor [Homo sapiens]	0	0	0	0	0	0	108166.1236	146767.7759	132038.4728
MRPL33	39S ribosomal protein L33, mitochondrial isoform a [Homo sapiens]	0	1525323.392	1493324.095	1524205.308	1259839.486	0	1365750.27	909131.5508	1380247.068
MRPL37	39S ribosomal protein L37, mitochondrial [Homo sapiens]	460851.4384	1848696.262	597718.0017	321176.6061	550477.6844	489094.7821	465557.6339	432738.713	646455.0691
MRPL38	39S ribosomal protein L38, mitochondrial [Homo sapiens]	514014.3879	518369.9364	659395.2618	527064.9078	658504.7073	609626.5139	1607424.267	458594.0633	392057.3414
MRPL39	39S ribosomal protein L39, mitochondrial isoform b [Homo sapiens]	739005.2132	861351.3921	3638002.028	3528665.79	1104103.981	970951.3901	867998.9993	656840.8394	735510.6887
MRPL4	39S ribosomal protein L4, mitochondrial isoform a [Homo sapiens]	882094.272	1018146.176	1150081.43	1269820.626	1419541.398	1124200.526	1201461.37	752839.3194	896721.7496
MRPL40	39S ribosomal protein L40, mitochondrial [Homo sapiens]	800877.8737	992426.9571	279360.7033	1046727.245	830289.7854	873453.1849	1005077.249	838035.8005	1047319.493
MRPL41	39S ribosomal protein L41, mitochondrial [Homo sapiens]	711827.2625	689506.5205	987002.8197	944836.8143	1049383.836	940830.1434	933622.8061	934020.5472	1029818.335
MRPL42	39S ribosomal protein L42, mitochondrial precursor [Homo sapiens]	1251546.624	1025598.843	1136687.293	888835.25	1670643.386	1575029.207	302851.2931	238971.2026	495568.0238
MRPL43	39S ribosomal protein L43, mitochondrial isoform a [Homo sapiens]	442274.4138	481665.5055	30470.62772	0	394259.394	482364.2005	447978.3306	0	361645.6
MRPL43	39S ribosomal protein L43, mitochondrial isoform d [Homo sapiens]	442274.4138	481665.5055	30470.62772	0	394259.394	482364.2005	447978.3306	0	361645.6
MRPL44	39S ribosomal protein L44, mitochondrial [Homo sapiens]	545087.3229	483725.1166	664664.3843	735881.428	738814.8497	687792.2095	735530.7937	587932.1649	446664.5484
MRPL45	39S ribosomal protein L45, mitochondrial isoform 1 [Homo sapiens]	707460.1658	714132.9525	917083.8596	1142808.401	1015852.484	599591.4994	758579.234	604582.6489	676161.3165
MRPL46	39S ribosomal protein L46, mitochondrial [Homo sapiens]	932461.2194	975408.825	1180669.684	1056880.782	1334546.141	1111643.619	1146543.895	962979.2113	968937.6427
MRPL47	39S ribosomal protein L47, mitochondrial isoform a [Homo sapiens]	559407.1506	349311.4565	819296.3472	686484.782	715172.1106	415379.2997	419018.4017	537051.453	491295.3292
MRPL48	39S ribosomal protein L48, mitochondrial [Homo sapiens]	769918.4463	911518.9302	1046695.281	892238.9288	925713.3151	904608.2122	881492.3638	558111.3927	559837.3655
MRPL49	39S ribosomal protein L49, mitochondrial [Homo sapiens]	1879362.889	1899962.755	2136238.298	1787830.038	2063825.375	1783984.22	1546492.141	1462820.985	1876812.285
MRPL50	39S ribosomal protein L50, mitochondrial [Homo sapiens]	1335608.194	1584254.151	1970991.567	1537798.943	1991746.119	1643047.288	1541083.678	1293139.319	1207779.442
MRPL51	39S ribosomal protein L51, mitochondrial [Homo sapiens]	0	456584.5	716353.7297	539825.3446	703977.4966	586572.7432	411267.3328	0	710268.8961
MRPL52	39S ribosomal protein L52, mitochondrial isoform a [Homo sapiens]	973614.7075	1273940.396	2189977.147	910309.033	1276132.396	0	1147863.217	0	1218536.198
MRPL53	39S ribosomal protein L53, mitochondrial [Homo sapiens]	2263292.466	1226327.338	2660502.094	2503919.852	2379473.868	2305310.426	2466183.844	2017518.864	2772209.618

MRPL54	39S ribosomal protein L54, mitochondrial [Homo sapiens]	1469804	1538406.258	1827087.516	0	1841786.781	0	0	996157	2425050.323
MRPL55	39S ribosomal protein L55, mitochondrial isoform b [Homo sapiens]	737600.7945	519239.561	593771.5383	0	774363.5387	564233.2476	812641.4264	677542.8794	410447.2093
MRPL9	39S ribosomal protein L9, mitochondrial [Homo sapiens]	485180.0048	475518.6119	581573.3603	532083.7217	537803.5214	583435.1631	509131.0994	414117.6786	279641.1607
MRPS10	28S ribosomal protein S10, mitochondrial [Homo sapiens]	785814.8653	681723.5917	1120053.833	743169.9106	846746.5884	841249.1722	698719.4092	697650.1361	771689.6778
MRPS11	28S ribosomal protein S11, mitochondrial isoform a [Homo sapiens]	553401.3205	0	909280.5769	366090.3173	406015.8726	653692.3158	348723.7853	544748.5337	0
MRPS14	28S ribosomal protein S14, mitochondrial [Homo sapiens]	453215.4786	0	227496.7107	731324.0233	585695.7786	757882.2857	0	0	0
MRPS15	28S ribosomal protein S15, mitochondrial [Homo sapiens]	328950.7996	335989.3108	0	258234.1486	556834.8514	317161.7922	297336.194	237155.5938	361438.3378
MRPS16	28S ribosomal protein S16, mitochondrial [Homo sapiens]	1929668.893	1609120.889	1338305.366	1060286.978	1591700.729	1553402.572	819233.918	1072579.714	0
MRPS17	28S ribosomal protein S17, mitochondrial [Homo sapiens]	2194443.586	2471418.556	2502121.831	1901015.266	3253517.237	2805743.48	2200995.12	1783755.973	2787340.915
MRPS18A	28S ribosomal protein S18a, mitochondrial isoform 1 precursor [Homo sapiens	0	366514.8182	467360.2045	245214.5818	521798.9591	386178.2682	299872.7557	305626.7466	0
MRPS18B	28S ribosomal protein S18b, mitochondrial [Homo sapiens]	702685.4309	929525.8586	718759.9752	934008.1108	508117.315	1111026.177	707696.5428	0	672240.4981
MRPS2	28S ribosomal protein S2, mitochondrial [Homo sapiens]	765618.9267	761185.4155	826809.2279	669209.6711	793961.5922	666601.7818	671623.1466	502922.2295	575103.8945
MRPS21	28S ribosomal protein S21, mitochondrial [Homo sapiens]	0	0	0	0	0	0	453599.7366	0	1038142.502
MRPS22	28S ribosomal protein S22, mitochondrial [Homo sapiens]	720022.2653	2388911.525	1033385.743	1423983.048	1051865.922	2473396.02	1367269.52	1497371.743	1576991.962
MRPS23	28S ribosomal protein S23, mitochondrial [Homo sapiens]	1386459.281	1583627.278	1780000.635	1466153.144	1953819.058	1742533.909	1605841.279	1114745.257	1547095.608
MRPS24	28S ribosomal protein S24, mitochondrial precursor [Homo sapiens]	705938.2966	513799.0592	958813.4242	663283.3159	1109799.851	528051.9692	535794.0946	0	584224.0195
MRPS25	28S ribosomal protein S25, mitochondrial [Homo sapiens]	1129021.756	1798765.704	1068638.902	1421584.001	1330258.04	898166.572	1288484.244	730042.0854	1474826.596
MRPS26	28S ribosomal protein S26, mitochondrial [Homo sapiens]	706363.5039	1039637.928	931103.0827	779833.1476	1314410.26	717710.4029	1203674.866	689028.9459	1043703.965
MRPS27	28S ribosomal protein S27, mitochondrial [Homo sapiens]	1034059.558	997513.1147	1724389.161	1190464.951	1408888.791	1033745.271	963618.6465	917077.7718	961982.8347
MRPS28	28S ribosomal protein S28, mitochondrial [Homo sapiens]	1256786.989	1150657.438	1402871.94	1356277.693	1397429.907	1126912.853	1316994.93	1171633.633	1303064.563
MRPS30	28S ribosomal protein S30, mitochondrial [Homo sapiens]	490833.4808	538764.5224	203523.2463	421108.8809	416264.6429	485617.279	339447.6401	577044.0361	363392.965
MRPS31	28S ribosomal protein S31, mitochondrial [Homo sapiens]	552419.8856	533766.6836	724356.4708	498801.9645	619920.3128	585994.0456	552073.0359	441371.6581	569023.6051
MRPS33	28S ribosomal protein S33, mitochondrial [Homo sapiens]	0	0	0	0	0	0	508167.8784	569469.538	403653.577
MRPS34	28S ribosomal protein S34, mitochondrial [Homo sapiens]	1251322.673	932305.5297	963235.4757	1085736.188	1394835.564	1323962.103	1353798.695	1224162.515	1486175.895
MRPS35	28S ribosomal protein S35, mitochondrial isoform 1 precursor [Homo sapiens]	887901.2934	733237.6762	1130354.919	843731.704	653639.085	884832.7993	830311.0623	672979.0046	580661.979
MRPS36	28S ribosomal protein S36, mitochondrial [Homo sapiens]	2105918.492	1795831.999	2927462.381	2625454.163	2725900.6	2311463.131	2416775.017	2398041.14	6280771.846
MRPS5	28S ribosomal protein S5, mitochondrial [Homo sapiens]	276578.6041	300515.081	377306.3814	241914.5401	420763.3018	332147.1166	156194.8243	163497.6864	273475.3557
MRPS6	28S ribosomal protein S6, mitochondrial [Homo sapiens]	1678384.876	1677150.352	2348874.038	1949151.314	1534045.857	2141476.184	1513564.94	1303050.288	1488435.842
MRPS7	28S ribosomal protein S7, mitochondrial [Homo sapiens]	1087304.288	1098147.039	1198085.281	1435975.815	1363383.477	1067645.188	1014587.058	1077394.922	1315186.66
MRPS9	28S ribosomal protein S9, mitochondrial [Homo sapiens]	500993.4771	494064.8795	748285.9718	563013.3062	540220.1656	452418.9555	446285.6092	455374.2244	585889.1338
MRRF	ribosome-recycling factor, mitochondrial isoform 1 precursor [Homo sapiens]	782464.165	1125062.834	714849.5971	1030180.08	829575.1787	990669.6441	979727.1283	824123.2598	960521.3291
MRS2	magnesium transporter MRS2 homolog, mitochondrial precursor [Homo sapiens]	117465.9386	135600.4997	139693.9694	230852.0259	79641.22691	121451.4507	168143.4529	158511.1129	107169.292
MRTO4	mRNA turnover protein 4 homolog [Homo sapiens]	2122047.524	2473403.918	3417587.908	3290622.641	2839365.735	2813872.046	2859265.404	2408109.816	3736510.275
MSH2	DNA mismatch repair protein Msh2 isoform 1 [Homo sapiens]	592425.9941	710886.0511	725030.4219	644341.064	722754.4944	677991.1478	914652.136	510991.1301	1030603.738
MSH3	DNA mismatch repair protein Msh3 [Homo sapiens]	79351.10885	78497.0334	38472.56378	45178.24955	103760.004	77262.25806	67516.24034	55108.00559	69693.31054
MSH6	DNA mismatch repair protein Msh6 [Homo sapiens]	330318.2393	356274.4772	458932.7199	372521.8952	422996.5392	395614.3668	362343.8549	303943.2624	350132.331
MSI2	RNA-binding protein Musashi homolog 2 isoform a [Homo sapiens]	194121.5329	188574.5426	101663.3366	1330055.216	170668.1748	240125.1231	170140.0719	146721.4174	123212.9837
MSI2	RNA-binding protein Musashi homolog 2 isoform b [Homo sapiens]	194121.5329	188574.5426	101663.3366	1330055.216	170668.1748	240125.1231	170140.0719	146721.4174	123212.9837
MSMO1	methylsterol monooxygenase 1 isoform 1 [Homo sapiens]	365653.9623	759226.387	431453.7432	718329.3322	717363.2115	693360.6062	314460.17	485042.5234	1159368.781
MSN	moesin [Homo sapiens]	4036952.381	4649159.875	4211925.833	4833103.279	3907493.679	4706447.529	4621889.147	3838873.625	3801464.936
MSRA	mitochondrial peptide methionine sulfoxide reductase isoform a [Homo sapier	0	367269.2	186939.5375	326631.8594	218147.9844	234913.0625	344222.1563	92925.33438	0
MSRB3	methionine-R-sulfoxide reductase B3 isoform 1 precursor [Homo sapiens]	0	0	0	0	0	0	0	53322.03958	590564.8208
MST1R	macrophage-stimulating protein receptor isoform 1 preproprotein [Homo sapie	9920.107969	27671.72766	42904.09817	94942.13722	13578.4473	36587.05488	7974.035427	4644.999599	3568.905607
MST4	serine/threonine-protein kinase MST4 isoform 1 [Homo sapiens]	1561817.048	1674964.682	1542318.733	1209895.275	1532555.931	1509763.365	1431162.403	1106373.696	1572225.356
MSTO1	protein misato homolog 1 isoform a [Homo sapiens]	150467.6984	878897.46	198928.8301	177679.6618	230608.1887	245162.6105	174843.0839	120992.4211	961161.5768
MT1E	metallothionein-1E [Homo sapiens]	3441776.486	2972399.106	4514325.904	3901046.115	3613462.808	3214006.346	2963049.736	3427529.947	3985075.158
MT2A	metallothionein-2 [Homo sapiens]	3962854.719	3539403.476	9749125.769	6771392.192	6823781.019	4871696.458	2715572.963	3426418.736	9511268.246
MTA1	metastasis-associated protein MTA1 isoform MTA1 [Homo sapiens]	83132.6975	63442.77878	102634.3303	131065.7303	127428.0668	105657.3035	51523.13079	80628.7453	90515.82774
MTA2	metastasis-associated protein MTA2 [Homo sapiens]	859781.9819	1137595.252	1270744.704	1075917.75	1210780.842	1058605.144	1120946.556	957346.1073	1208764.484
MTA3	metastasis-associated protein MTA3 [Homo sapiens]	59593.40275	118953.8868	40265.61261	51586.30054	134008.1247	75122.54306	82102.69221	64415.76802	125801.9384
MTAP	S-methyl-5'-thioadenosine phosphorylase [Homo sapiens]	5332594.598	5297760.018	6713849.897	5659740.746	6763919.886	5850567.024	4737676.994	3353970.085	4342833.282
MTCH1	mitochondrial carrier homolog 1 PSAP-LL [Homo sapiens]	705010.8069	836498.3992	1305079.754	768132.5612	1148872.256	1017659.106	968788.9786	578982.279	932852.6611
MTCH2	mitochondrial carrier homolog 2 [Homo sapiens]	4296993.632	4777244.609	5350551.977	5306963.599	6700843.764	5960769.375	5492930.884	3657072.708	5255411.501
MTDH	protein LYRIC [Homo sapiens]	629304.8594	814570.1672	936819.9319	789275.9585	937105.6624	844556.6402	815180.2873	705233.5764	791712.3336
MTERFD1	mTERF domain-containing protein 1, mitochondrial precursor [Homo sapiens]	98019.63429	85181.34286	101391.5943	110629.1229	115107.3114	0	0	0	0
MTF2	metal-response element-binding transcription factor 2 isoform a [Homo sapie	7576.029965	89222.02427	115121.6118	0	21369.1228	0	0	0	0
MTFP1	mitochondrial fission process protein 1 isoform a [Homo sapiens]	461417.5397	725549.0635	658786.5457	820759.2817	593838.377	357949.754	522551.3671	596369.7262	610022.8889
MTFR1	mitochondrial fission regulator 1 isoform 3 [Homo sapiens]	359276.413	222708.4203	312896.8533	166080.0308	584237.6522	102746.8635	213774	173281.4565	266094.0435
MTG2	GTP-binding protein 5 [Homo sapiens]	16771.60638	38563.46354	0	22729.91609	0	16147.40278	30052.03588	58474.87886	29771.15895
MTHFD1	C-1-tetrahydrofolate synthase, cytoplasmic [Homo sapiens]	2582186.376	3143844.088	3344970.669	3077421.347	3389845.105	3270862.819	3020969.825	2649370.084	3395147.87
MTHFD1L	monofunctional C1-tetrahydrofolate synthase, mitochondrial isoform 1 precu	391916.6222	473422.6299	491402.7286	434552.0356	506875.1032	342577.8684	453943.839	2030121.251	523098.6719
MTHFD2	bifunctional methylenetetrahydrofolate dehydrogenase/cyclohydrolase, mitoch	1960288.558	2158143.408	2687013.033	2143940.613	2575326.814	2394714.463	2321412.901	1847925.194	1678489.766
MTHFS	5-formyltetrahydrofolate cyclo-ligase isoform a [Homo sapiens]	820214.0863	1081216.891	1022815.217	882403.8461	1504381.285	942954.2415	974955.8648	754812.8091	751213.6615
MTIF2	translation initiation factor IF-2, mitochondrial precursor [Homo sapiens]	86548.5596	0	132440.1805	48154.51096	175491.9156	68690.79222	53633.12086	79103.51262	42484.73344
MTM1	myotubularin [Homo sapiens]	0	65372.04785	100826.6465	24735.70313	0	16973.12769	28026.82715	38873.10107	0
MTMR1	myotubularin-related protein 1 [Homo sapiens]	56608.02931	433633.7317	75037.45073	100394.6822	102155.1796	148056.7066	138195.5522	87751.64228	145236.731
MTMR12	myotubularin-related protein 12 [Homo sapiens]	87174.21747	40975.57065	39683.9589	0	78356.34332	22378.89298	44366.43719	42217.6989	46221.7576
MTMR14	myotubularin-related protein 14 isoform 2 [Homo sapiens]	85840.37241	0	83587.57366	88018.09879	80460.39585	78337.75768	71588.12192	58173.98884	70924.77094
MTMR2	myotubularin-related protein 2 isoform 1 [Homo sapiens]	5279864.204	459638.1792	433473.7922	342440.0598	515005.2196	442704.8031	391832.0691	293580.4307	494851.9353
MTMR6	myotubularin-related protein 6 [Homo sapiens]	60286.02894	53237.4385	127565.8051	80983.40799	94785.49146	77847.76	70994.85555	0	67057.2761
MTO1	protein MTO1 homolog, mitochondrial isoform c [Homo sapiens]	51249.50853	59477.30676	52187.31307	48747.93345	107931.9113	63770.08532	63874.91085	71497.31143	83386.65679
MTOR	serine/threonine-protein kinase mTOR [Homo sapiens]	52669.06059	63418.14446	85963.53963	72721.92428	74751.78804	68211.80269	69761.5237	59088.98832	63107.7991
MTPAP	poly(A) RNA polymerase, mitochondrial precursor [Homo sapiens]	209534.3272	124696.49	276514.1474	144484.4549	267989.1553	164965.97	159203.6703	84857.6384	237276.4157
MTPN	myotrophin [Homo sapiens]	11146148.1	14921202.61	14896169.97	14027736.97	13120313	14354329.98	15223971.79	11638823.45	16276017.55
MTR	methionine synthase [Homo sapiens]	121457.9658	125472.2379	128707.4484	98878.50952	129546.2198	119381.8733	89510.56987	133961.0795	68616.5574
MTRR	methionine synthase reductase isoform 2 [Homo sapiens]	822782.9226	747112.214	780011.27	895135.8708	1136240.896	767814.063	871301.1037	698374.3568	849901.4852
MTX1	metaxin-1 isoform 1 [Homo sapiens]	344406.2324	793293.1751	643361.701	875986.729	459913.8144	959104.9047	868814.3497	0	981771.7078
MTX2	metaxin-2 [Homo sapiens]	2023996.557	1658582.458	1593006.383	1800367.362	2779232.61	610308.68	1620930.716	565959.1211	1896820.843
MTX3	metaxin-3 isoform 2 [Homo sapiens]	0	0	0	0	0	0	0	0	0
MUC1	mucin-1 isoform 10 precursor [Homo sapiens]	76790.30306	209278.5032	0	321272.0602	71381.98177	0	491532.0156	394269.5026	0
MUC13	mucin-13 precursor [Homo sapiens]	40953.11218	484452.8611	423716.6315	319631.6962	564717.2191	208310.4466	299906.1435	0	343329.6496
MUS81	crossover junction endonuclease MUS81 [Homo sapiens]	0	0	0	0	0	0	11117.9495	0	3134.614069
MUT	methylmalonyl-CoA mutase, mitochondrial precursor [Homo sapiens]	187644.0027	213016.7809	309034.8792	209160.6892	235539.5897	221231.7721	214172.4461	176761.9593	248786.1252
MUTED	biogenesis of lysosome-related organelles complex 1 subunit 5 isoform 1 [Ho	76701.86019	236261.9253	236476.1051	162663.833	207608.1778	276653.1975	77827.98325	0	265144.2577
MVB12A	multivesicular body subunit 12A [Homo sapiens]	383501.3132	542922.9587	856921.5132	741552.6059	533605.8053	541179.8514	393124.6901	0	467905.2346
MVD	diphosphomevalonate decarboxylase [Homo sapiens]	0	0	0	0	0	0	116693.0373	76625.40858	0
MVK	mevalonate kinase [Homo sapiens]	222138.1816	157298.2683	430816.0198	185059.2883	241344.7294	312350.7487	108981.3585	270887.5893	196983.8776
MVP	major vault protein [Homo sapiens]	243968.9775	290574.0957	318744.13	293711.7609	313082.0012	312290.119	234033.7906	194051.6955	247094.0824
MYADM	myeloid-associated differentiation marker [Homo sapiens]	1338406.664	650426.6563	1201843.311	1190677.551	1436635.488	993678.7344	1025464.501	840287.9466	1340137.945
MYBBP1A	myb-binding protein 1A isoform 1 [Homo sapiens]	887939.2716	1266746.623	1191951.887	954481.77	1278653.95	1000362.47	1039278.842	834437.842	1365427.672
MYCBP	C-Myc-binding protein [Homo sapiens]	3882108.367	5302460.569	7520365.498	4542927.65	4128942.025	4738578.89	4997676.64	4421550.267	4779664.196
MYCBP2	probable E3 ubiquitin-protein ligase MYCBP2 [Homo sapiens]	0	0	0	0	0	0	6130.655653	3446.034527	5338.543638
MYEOV2	myeloma-overexpressed gene 2 protein isoform 2 [Homo sapiens]	8029285.071	902232.75	16321942.4	0	2222862.464	0	0	0	0
MYH10	myosin-10 isoform 1 [Homo sapiens]	120409.3023	87895.81782	142059.0996	150105.1288	129275.2599	253811.8031	150913.1047	0	146797.4596
MYH14	myosin-14 isoform 3 [Homo sapiens]	0	0	0	0	0	0	0	0	0
MYH15	myosin-15 precursor [Homo sapiens]	0	0	0	0	0	0	0	0	0
MYH9	myosin-9 [Homo sapiens]	600171.3673	690764.6051	820784.329	762028.444	813948.8821	763054.0679	730970.7301	669430.9981	843823.1639
MYL12B	myosin regulatory light chain 12B [Homo sapiens]	7618721.764	7865612.489	9157180.88	5986624.086	9261088.83	6543661.927	8147467.727	7835951.25	10483961.59
MYL6	myosin light polypeptide 6 isoform 2 [Homo sapiens]	2016942.716	820610.3603	1642436.373	2011397.3	2522420.396	2517373.845	1562722.661	17022645.93	12331786.69
MYL6	myosin light polypeptide 6 isoform 1 [Homo sapiens]	2016942.716	820610.3603	1642436.373	2011397.3	2522420.396	2517373.845	1562722.661	17022645.93	12331786.69
MYL6B	myosin light chain 6B [Homo sapiens]	210760.9946	251501.1559	150562.4839	196527.6841	0	152271.0874	139547.5666	0	155343.7057
MYLK2	myosin light chain kinase 2, skeletal/cardiac muscle [Homo sapiens]	0	0	0	0	0	0	432278.9914	368929.0911	0
MYO10	unconventional myosin-X [Homo sapiens]	10204.5565	0	33045.33764	12241.98565	13590.9832	12234.6408	12269.02225	7827.316221	13414.14543
MYO18A	unconventional myosin-XVIIIa isoform a [Homo sapiens]	49309.64655	44667.78758	48082.06399	50437.95789	57752.35097	57366.40991	42046.64064	37667.63335	604819.6696
MYO19	unconventional myosin-XIX isoform 2 [Homo sapiens]	45466.79033	18342.47832	44590.25855	22012.91001	48413.27318	38934.0716	16917.98257	16791.02773	12249.26169
MYO1B	unconventional myosin-Ib isoform 1 [Homo sapiens]	177509.7117	184677.1218	230343.1273	197361.7852	234662.1506	201971.0629	219856.7054	206037.96	220462.5371
MYO1C	unconventional myosin-Ic isoform a [Homo sapiens]	627435.3845	644959.6664	822671.0212	705579.0068	881675.4942	771422.1963	752627.4037	674586.2433	3387566.054
MYO1E	unconventional myosin-Ie [Homo sapiens]	624263.2135	670203.0658	827205.6671	695483.0247	927594.2265	797606.6585	704564.5071	618365.2017	619572.3234
MYO1G	unconventional myosin-Ig [Homo sapiens]	735247.7943	226070.1756	261255.3108	186255.5343	278904.0092	230697.7346	200585.5331	150017.8675	279405.6734
MYO6	unconventional myosin-VI [Homo sapiens]	66799.73529	44178.00108	59236.54658	51039.86647	62151.93427	58447.45537	68761.60915	34285.11594	28969.92869
MYO9B	unconventional myosin-IXb isoform 1 [Homo sapiens]	0	0	0	0	0	0	12314.4387	14631.76169	11137.19169
MYOF	myoferlin isoform a [Homo sapiens]	425900.9723	478045.9818	566422.0659	487339.6794	579880.8678	521020.3458	526125.2414	409150.941	524633.2666
MYPN	myopalladin isoform a [Homo sapiens]	109025.3009	133824.4847	98748.18941	133039.6256	144696.1379	115870.9453	105053.4973	106509.6117	108622.2771
MZT1	mitotic-spindle organizing protein 1 [Homo sapiens]	1619609.192	1512757.769	1607357.765	1725318.88	1912952.692	1352990.692	0	1737522.163	667014.9038
MZT2B	mitotic-spindle organizing protein 2B [Homo sapiens]	502416.4141	705250.9309	395867.8789	680642.7747	554825.6692	798122.9141	564105.5442	460141.557	599099.582
N6AMT1	hemK methyltransferase family member 2 isoform 1 [Homo sapiens]	0	387284.6059	541340.678	73908.67903	543983.9364	0	441844.892	476903.8051	538133.1441
NAA10	N-alpha-acetyltransferase 10 isoform 1 [Homo sapiens]	2469605.943	2300876.374	3078922.361	2469048.154	3578600.418	2356333.008	2146781.955	2003284.234	2443326.714
NAA15	N-alpha-acetyltransferase 15, NatA auxiliary subunit [Homo sapiens]	484296.2428	575357.2285	599755.485	579752.3554	667486.3696	597461.0339	581118.1264	495236.153	641965.4584
NAA16	N-alpha-acetyltransferase 16, NatA auxiliary subunit isoform 1 [Homo sapier	66053.66068	64292.15046	48451.64329	29840.67683	0	0	30378.8128	0	61814.32885
NAA20	N-alpha-acetyltransferase 20 isoform a [Homo sapiens]	0	0	0	0	0	0	581810.2661	855521.1855	5586801.61

NAA25	N-alpha-acetyltransferase 25, NatB auxiliary subunit [Homo sapiens]	206878.8472	225861.7351	291036.1113	241695.7885	286095.8733	263871.3945	240358.2666	257663.9305	242920.6366
NAA30	N-alpha-acetyltransferase 30 [Homo sapiens]	0	0	0	0	0	0	190346.9731	190935.0558	0
NAA35	N-alpha-acetyltransferase 35, NatC auxiliary subunit [Homo sapiens]	84577.8369	79084.17518	146111.8845	67605.85296	149374.6718	144458.3061	162496.4641	139032.4403	85556.84628
NAA38	N-alpha-acetyltransferase 38, NatC auxiliary subunit [Homo sapiens]	4868364.712	8967479.308	8161887.204	11444583.31	6739428.338	11782033.98	10972594.42	6990567.954	11259328.69
NAA40	N-alpha-acetyltransferase 40 [Homo sapiens]	749095.0824	577032.5984	972876.3365	780638.6241	966286.4929	798596.804	755033.7504	633081.0244	753116.7199
NAA50	N-alpha-acetyltransferase 50 [Homo sapiens]	2931167.3	1354579.356	2559367.404	2227548.173	2581767.362	2428630.394	2374375.835	3908708.801	3282055.621
NAB1	NGFI-A-binding protein 1 [Homo sapiens]	0	33085.20918	0	41791.12787	70956.73724	52622.65372	33338.99745	0	38364.69515
NABP2	SOSS complex subunit B1 [Homo sapiens]	617883.5597	528156.5522	497008.6713	610575.1381	505032.5765	470497.2845	455043.6493	267495.9627	343083.4058
NACA	nascent polypeptide-associated complex subunit alpha isoform a [Homo sapien	16834996.1	19600079.35	23105327.74	20765209.73	22611822.89	21293314.34	20943427.92	16723271.08	21466944.89
NACC1	nucleus accumbens-associated protein 1 [Homo sapiens]	291001.2171	337660.5977	281022.2049	364405.1548	432606.0802	352701.107	317296.196	234399.7464	343990.1505
NADKD1	NAD kinase domain-containing protein 1, mitochondrial isoform 1 [Homo sapie	1402650.369	735573.4924	818016.2813	646147.8237	893442.6061	697650.5207	995588.5394	1203352.744	964521.2581
NAE1	NEDD8-activating enzyme E1 regulatory subunit isoform a [Homo sapiens]	1267583.634	1149119.084	1660844.766	843614.2962	1661468.623	1388388.225	1449781.211	1035648.631	1028718.642
NAF1	H/ACA ribonucleoprotein complex non-core subunit NAF1 isoform a [Homo sapie	696793.772	0	921520.1787	536358.29	839136.7385	567426.9683	538330.7043	423497.957	208071.4631
NAGA	alpha-N-acetylgalactosaminidase precursor [Homo sapiens]	283642.5577	203933.182	548357.1739	341147.1833	617135.6959	110395.2822	108279.4564	206408.7668	310764.0461
NAGK	N-acetyl-D-glucosamine kinase [Homo sapiens]	532723.2782	224754.2875	898071.8354	470255.541	941322.4677	512614.4737	314361.4212	394470.2333	571848.2882
NAGLU	alpha-N-acetylglucosaminidase precursor [Homo sapiens]	88012.41733	72701.48848	60518.80244	65870.75922	126096.8183	87180.5653	0	78772.80935	64111.8753
NAGS	N-acetylglutamate synthase, mitochondrial [Homo sapiens]	0	0	0	0	0	0	0	0	0
NAMPT	nicotinamide phosphoribosyltransferase precursor [Homo sapiens]	2728245.607	2556265.251	3215550.299	2391556.272	3757639.981	3162691.646	2397060.54	2920780.32	2962734.572
NANP	N-acylneuraminate-9-phosphatase [Homo sapiens]	0	0	0	0	0	0	0	46610.79167	148640.6175
NANS	sialic acid synthase [Homo sapiens]	3167513.957	3071715.085	3862659.939	3198766.636	3927501.232	3283640.648	3400574.243	3184904.07	3352045.498
NAP1L1	nucleosome assembly protein 1-like 1 [Homo sapiens]	17302568.27	20987803.43	19400890.83	20828890.52	23403103.91	21957490.66	20308182.81	17938070.89	21206426.18
NAP1L4	nucleosome assembly protein 1-like 4 [Homo sapiens]	6051741.2	7696321.791	7188821.33	7581074.818	6716988.664	7773282.372	7450785.438	5778133.694	7144183.035
NAPA	alpha-soluble NSF attachment protein [Homo sapiens]	2804792.058	2754408.651	3619966.589	3322103.562	3901136.51	3289918.631	3148007.669	2411851.108	3190318.519
NAPG	gamma-soluble NSF attachment protein [Homo sapiens]	675216.2662	848738.9701	591327.3246	814092.9336	1038657.937	623211.7295	724280.9928	757609.3276	756450.2634
NAPRT1	nicotinate phosphoribosyltransferase [Homo sapiens]	1150364.064	1114074.508	1510855.509	1250199.586	1595103.2	1317619.847	1136934.99	1069366.122	1270010.559
NARG2	NMDA receptor-regulated protein 2 isoform a [Homo sapiens]	16332.80244	8276.12979	0	0	41431.88052	33560.71948	15554.09026	0	8996.230925
NARR	ras-related protein Rab-34, isoform NARR [Homo sapiens]	98843.92105	84439.22214	127042.6503	0	125887.8728	126862.8816	52847.47944	50085.9364	102468.7654
NARS	asparagine--tRNA ligase, cytoplasmic [Homo sapiens]	2280062.35	2203434.798	2661323.222	2187806.46	3277102.771	2375114.161	2116798.058	2198605.117	2557499.864
NARS2	probable asparagine--tRNA ligase, mitochondrial isoform 1 precursor [Homo s	401829.7991	1694210.976	1514843.946	1777298.245	1359383.748	473413.3334	448034.8788	1577938.658	554339.4405
NASP	nuclear autoantigenic sperm protein isoform 2 [Homo sapiens]	2985727.484	2509263.872	3429812.766	3220656.622	3458505.81	3216750.268	3249969.582	2900887.24	2896441.738
NAT10	N-acetyltransferase 10 isoform a [Homo sapiens]	688964.734	666402.4242	957743.2134	737765.0758	929020.351	830185.1224	754100.7023	654040.4289	817876.0688
NAV1	neuron navigator 1 isoform 1 [Homo sapiens]	12856.35906	14031.07103	13368.88986	4685.662838	17900.82264	7970.446115	9163.975085	0	9581.991132
NBAS	neuroblastoma-amplified sequence [Homo sapiens]	23173.34698	22305.67662	24849.29309	26831.30328	31364.92248	27808.66744	19807.70082	23179.97319	22540.06731
NBEAL2	neurobeachin-like protein 2 [Homo sapiens]	17272.66396	94331.46258	22798.42747	12901.71654	17596.75285	88111.35788	112512.59	7241.215586	29380.00154
NBN	nibrin [Homo sapiens]	90409.94382	97431.26542	72743.88649	123869.2252	125216.8892	132371.1096	85089.93417	70077.89395	161643.6962
NBR1	next to BRCA1 gene 1 protein [Homo sapiens]	29437.29303	24905.37868	0	24604.26799	0	23118.26073	19695.13115	16350.40136	0
NCAPD2	condensin complex subunit 1 [Homo sapiens]	388765.0358	376045.2394	437793.7422	381782.9295	423200.3173	440662.175	451172.6066	391755.1503	415074.7871
NCAPD3	condensin-2 complex subunit D3 [Homo sapiens]	93648.53228	102373.0792	128258.2658	104354.7316	136746.747	105545.0047	77959.16774	57479.09197	179703.495
NCAPG	condensin complex subunit 3 [Homo sapiens]	461656.9465	535588.8394	614102.3495	478934.0795	636242.7841	540196.9071	463917.1828	410725.8674	540696.199
NCAPG2	condensin-2 complex subunit G2 [Homo sapiens]	102761.8522	364909.0724	137244.6113	97295.40635	104528.8663	493845.0861	100325.0597	315915.8543	63640.04509
NCAPH	condensin complex subunit 2 [Homo sapiens]	501008.2298	506527.344	506762.9472	569292.2673	702590.1774	670947.475	628726.8984	492799.1516	558562.4702
NCAPH2	condensin-2 complex subunit H2 isoform 3 [Homo sapiens]	75223.23316	100622.4093	141155.6438	410632.9443	119108.294	47272.28109	71118.57513	0	88258.49352
NCBP1	nuclear cap-binding protein subunit 1 [Homo sapiens]	901587.5651	981628.4525	1129902.571	1116248.036	1162300.277	1016343.511	949181.7838	744082.7599	902797.8246
NCBP2	nuclear cap-binding protein subunit 2 isoform 1 [Homo sapiens]	0	0	0	0	0	0	1048774.958	2297493.798	0
NCDN	neurochondrin isoform 1 [Homo sapiens]	245811.4497	464542.3394	570966.6394	411764.7624	531231.6122	507707.3068	469271.7163	285598.4357	422557.3946
NCEH1	neutral cholesterol ester hydrolase 1 isoform a [Homo sapiens]	1501495.2	1847228.242	1681684.173	1707461.735	1089512.178	1031551.978	1142658.914	1576861.587	2005549.417
NCK1	cytoplasmic protein NCK1 isoform 1 [Homo sapiens]	381413.5786	470784.5991	288794.9049	363386.4256	396528.7454	556074.2757	369622.9662	269244.2432	314069.2545
NCKAP1	nck-associated protein 1 isoform 2 [Homo sapiens]	378244.7005	357848.3079	347047.6798	435879.1074	332052.7372	3413580.904	448368.9382	385229.1421	385806.9094
NCKIPSD	NCK-interacting protein with SH3 domain isoform 1 [Homo sapiens]	208780.7844	232235.0502	254312.3287	277097.9193	285430.5483	283808.1752	241443.8089	214403.0449	198554.9706
NCL	nucleolin [Homo sapiens]	10936294.41	11114640.4	14282241.92	11880053.25	14712843.6	11884419.85	11378740.2	10329664.34	11570388.29
NCLN	nicalin precursor [Homo sapiens]	1034883.159	1013751.292	1048084.705	1006656.272	1227918.948	1004631.947	1095059.955	919978.5199	1209571.86
NCOA3	nuclear receptor coactivator 3 isoform a [Homo sapiens]	21566.81243	0	39928.5927	0	78641.33097	20784.58527	19919.67199	0	37076.94681
NCOA5	nuclear receptor coactivator 5 [Homo sapiens]	311845.4866	483542.8496	366244.0675	284882.6627	348029.8888	359651.0385	330786.4945	277804.0364	611827.2101
NCOA6	nuclear receptor coactivator 6 isoform 1 [Homo sapiens]	191838.2037	32249.69235	3787.424435	0	0	689833.8821	0	9976.567349	2230.485116
NCOA7	nuclear receptor coactivator 7 isoform 1 [Homo sapiens]	37892.15796	19471.93328	39562.58225	53393.07311	50762.52742	33345.25953	41326.54258	43215.18668	0
NCOR1	nuclear receptor corepressor 1 isoform 1 [Homo sapiens]	20027.94692	22269.8283	63009.84919	29764.73179	48483.57905	33495.43547	34540.82471	8577.698683	27931.34197
NCOR2	nuclear receptor corepressor 2 isoform 1 [Homo sapiens]	5477.16835	9842.646322	7034.900235	18415.30849	10171.10998	20224.81505	7947.01118	5755.969617	22563.08747
NCS1	neuronal calcium sensor 1 isoform 1 [Homo sapiens]	0	542273.2829	288375.3	323351.8875	368765.5563	352256.2258	441571.6156	431991.4735	59477.76406
NCSTN	nicastrin precursor [Homo sapiens]	613982.9399	631612.1174	725482.338	748943.4178	754601.1362	984918.6455	747652.2915	598014.9451	709251.8164
ND1	NADH dehydrogenase subunit 1 [Homo sapiens]	1009078.769	477704.7212	954637.3077	0	0	937493.0529	0	0	0
ND2	NADH dehydrogenase subunit 2 [Homo sapiens]	807640.2417	0	1448926.758	27438.21042	1681861.358	783608.1855	0	0	0
ND3	NADH dehydrogenase subunit 3 [Homo sapiens]	5776125.141	8073924.094	6869329	6721999.688	8583510.688	6452868.563	7467966.625	5563268.25	8389092.906
ND4	NADH dehydrogenase subunit 4 [Homo sapiens]	165822.9949	323763.1096	285234.2089	371250.6815	268860.6678	272778.9084	340508.9178	307766.8219	362622.7997
ND5	NADH dehydrogenase subunit 5 [Homo sapiens]	50616.61343	208510.6057	835518.6481	447567.9005	886871.7685	530495.682	558167.5087	204868.9884	527632.8148
ND6	NADH dehydrogenase subunit 6 [Homo sapiens]	0	0	0	0	0	0	0	2044455.238	3698457.81
NDC80	kinetochore protein NDC80 homolog [Homo sapiens]	164705.5194	116715.9715	125196.915	114212.1968	188043.9908	161226.1231	90117.71377	114982.0171	90065.56083
NDE1	nuclear distribution protein nudE homolog 1 [Homo sapiens]	93634.02844	143867.9754	99917.49701	179135.8038	138585.3501	93557.37928	112566.295	150564.2849	0
NDFIP1	NEDD4 family-interacting protein 1 [Homo sapiens]	816288.754	401466.739	915387.7798	442220.0492	867038.0635	819359.8611	475075.4981	453424.5903	304573.3175
NDFIP2	NEDD4 family-interacting protein 2 isoform 1 [Homo sapiens]	170273.4367	264994.0814	0	163856.391	63617.93314	211197.2835	0	0	0
NDRG1	protein NDRG1 isoform 1 [Homo sapiens]	790226.3044	692475.9698	1009714.526	642674.0721	1145166.123	536314.8018	615519.0245	588248.5192	534041.1688
NDRG3	protein NDRG3 isoform a [Homo sapiens]	1221431.255	1399280.845	1557743.611	1267298.632	1744021.928	1636139.632	1647107.861	1371840.811	1550556.226
NDUFA10	NADH dehydrogenase [ubiquinone] 1 alpha subcomplex subunit 10, mitochondria	790035.6724	957153.116	1197209.041	959885.3686	1320363.024	965745.0481	907334.2808	567684.0628	845954.9885
NDUFA12	NADH dehydrogenase [ubiquinone] 1 alpha subcomplex subunit 12 isoform a [Ho	625218.949	528548.3651	708832.8088	618185.796	778198.4069	679011.8419	581089.5699	485505.2129	616182.3829
NDUFA13	NADH dehydrogenase [ubiquinone] 1 alpha subcomplex subunit 13 [Homo sapiens	2474149.707	1996835.772	3003564.202	2488113.963	2922918.623	2664451.933	2378874.73	1395026.735	2113973.925
NDUFA2	NADH dehydrogenase [ubiquinone] 1 alpha subcomplex subunit 2 isoform 1 [Hom	1701667.378	2739233.039	2748056.888	2051846.9	3313722.869	2148723.149	1711946.238	1704722.179	2404463.939
NDUFA3	NADH dehydrogenase [ubiquinone] 1 alpha subcomplex subunit 3 [Homo sapiens]	5970305	5857416.404	5812550.881	5752936.056	8137104.852	3263670.935	5127180.222	4455717.474	6826155.722
NDUFA4	NADH dehydrogenase [ubiquinone] 1 alpha subcomplex subunit 4 [Homo sapiens]	16066455.29	13291080.33	20369012.83	16710747.37	15558683.03	17282284.3	15336058.68	13098096.08	15687153.9
NDUFA5	NADH dehydrogenase [ubiquinone] 1 alpha subcomplex subunit 5 [Homo sapiens]	3357442.431	5306418.51	7099324.788	4792177.323	7191294.9	6125384.512	4628556.962	4709363.838	5499550.083
NDUFA6	NADH dehydrogenase [ubiquinone] 1 alpha subcomplex subunit 6 [Homo sapiens]	0	0	0	0	0	0	0	247927.8182	1545287.585
NDUFA7	NADH dehydrogenase [ubiquinone] 1 alpha subcomplex subunit 7 [Homo sapiens]	307946.6493	0	557076.4703	326962.161	639159.339	366301.4544	0	0	0
NDUFA8	NADH dehydrogenase [ubiquinone] 1 alpha subcomplex subunit 8 [Homo sapiens]	1881515.989	1197618.138	2876807.807	2361347.713	1577210.54	1310887.575	1155324.346	1050629.928	1788885.298
NDUFA9	NADH dehydrogenase [ubiquinone] 1 alpha subcomplex subunit 9, mitochondria	1184229.209	1129475.791	1625644.092	1250786.001	1639391.146	1295553.902	1122612.179	892804.0713	972680.2661
NDUFAB1	acyl carrier protein, mitochondrial precursor [Homo sapiens]	8981329.467	9292330.796	16175890.21	8456882.927	15428864.64	13921255.46	9773344.207	9673936.564	9386946.155
NDUFAF1	complex I intermediate-associated protein 30, mitochondrial precursor [Homo	0	19600.22555	91692.67857	78387.2551	106782.4728	130204.8218	0	57494.7466	158876.7555
NDUFAF2	mimitin, mitochondrial [Homo sapiens]	1202569.797	1356915.04	1146250.124	1569750.678	1526665.376	1625715.02	1538325.13	1249605.602	1578031.996
NDUFAF3	NADH dehydrogenase [ubiquinone] 1 alpha subcomplex assembly factor 3 isofor	975915.2621	4695778.056	1499932.242	810152.2581	5170733.248	914167.6361	4679747.121	2855060.887	1065835.994
NDUFAF4	NADH dehydrogenase [ubiquinone] 1 alpha subcomplex assembly factor 4 [Homo	825291.8806	542900.6504	1491596.072	888765.8088	1994043.172	863875.7703	1026843.55	605489.0213	905794.1062
NDUFAF6	NADH dehydrogenase (ubiquinone) complex I, assembly factor 6 precursor [Hom	101232.3758	121734.7483	137670.0327	86386.7057	0	94650.39404	120309.7947	73558.24709	0
NDUFAF7	NADH dehydrogenase [ubiquinone] complex I, assembly factor 7 isoform 1 [Hom	226301.9067	139561.0849	324246.5198	203024.6458	274849.579	231964.6554	179576.061	159965.4448	329762.2573
NDUFB1	NADH dehydrogenase [ubiquinone] 1 beta subcomplex subunit 1 [Homo sapiens]	0	0	0	0	0	0	0	3237288.85	4610803.25
NDUFB10	NADH dehydrogenase [ubiquinone] 1 beta subcomplex subunit 10 [Homo sapiens]	2392421.739	2579602.623	3051532.877	2066388.727	3160139.262	2233335.343	2484142.395	1536669.043	2577011.273
NDUFB11	NADH dehydrogenase [ubiquinone] 1 beta subcomplex subunit 11, mitochondria	2358344.037	4378762.963	4401508.603	3538343.324	3885139.038	3509410.81	2976810.665	5283665.897	6774488.622
NDUFB3	NADH dehydrogenase [ubiquinone] 1 beta subcomplex subunit 3 [Homo sapiens]	2463308.771	1749617.408	3449112.517	2390920.36	1772830.824	2293217.792	1886310.697	1526540.613	2349462.563
NDUFB4	NADH dehydrogenase [ubiquinone] 1 beta subcomplex subunit 4 isoform 1 [Hom	1554292.612	1752584.583	2796548.319	1675992.902	2572794.005	1164917.183	1992656.302	1761909.084	1743417.856
NDUFB5	NADH dehydrogenase [ubiquinone] 1 beta subcomplex subunit 5, mitochondrial	906014.4856	215436.7256	945752.4964	582953.9368	731706.0115	692316.569	78163.46875	0	1389643.389
NDUFB6	NADH dehydrogenase [ubiquinone] 1 beta subcomplex subunit 6 isoform 1 [Hom	1661371.284	4035471.875	3769168.443	2121075.825	3975372.362	2358526.032	1872178.8	2327090.971	2635221.343
NDUFB7	NADH dehydrogenase [ubiquinone] 1 beta subcomplex subunit 7 [Homo sapiens]	920207.048	751041.27	1112961.355	997462.7717	1359713.762	1340412.587	1252448.065	753081.5091	1316933.609
NDUFB8	NADH dehydrogenase [ubiquinone] 1 beta subcomplex subunit 8, mitochondrial	1199272.966	1439062.408	1245613.016	1267606.881	1688920.404	1297728.313	927538.3952	1171457.384	1657353.616
NDUFB9	NADH dehydrogenase [ubiquinone] 1 beta subcomplex subunit 9 [Homo sapiens]	1743361.163	1230421.458	1347185.551	1485073.512	1301563.862	1292076.012	1174410.093	1342785.961	1534953.478
NDUFC1	NADH dehydrogenase [ubiquinone] 1 subunit C1, mitochondrial precursor [Hom	828049.6757	0	945763.1757	753239.8784	929020.0203	726150.1116	0	0	0
NDUFC2	NADH dehydrogenase [ubiquinone] 1 subunit C2 isoform 1 [Homo sapiens]	0	0	0	0	0	0	1483566.864	1290732.148	773355.2542
NDUFS1	NADH-ubiquinone oxidoreductase 75 kDa subunit, mitochondrial isoform 5 [Hor	486996.6375	799818.1954	776116.917	762547.3869	885260.5749	857940.0841	789377.8117	604946.0749	769787.583
NDUFS2	NADH dehydrogenase [ubiquinone] iron-sulfur protein 2, mitochondrial isofor	850202.3929	1075872.501	1344836.279	935435.1599	1413702.043	1200004.824	1062629.764	889715.7995	1170639.901
NDUFS3	NADH dehydrogenase [ubiquinone] iron-sulfur protein 3, mitochondrial precu	2326117.151	2762710.337	3067726.39	2748932.573	2998944.26	2930967.154	2656100.16	2237598.423	2542065.967
NDUFS4	NADH dehydrogenase [ubiquinone] iron-sulfur protein 4, mitochondrial precu	805706.2912	829887.0447	1775306.768	1573217.882	1085158.412	1639653.08	1484336.635	0	1483545.116
NDUFS5	NADH dehydrogenase [ubiquinone] iron-sulfur protein 5 [Homo sapiens]	1357323.937	1578309.804	877859.6531	1000034.629	859270.8966	830220.7997	1318983.034	1131460.648	1046808.264
NDUFS6	NADH dehydrogenase [ubiquinone] iron-sulfur protein 6, mitochondrial precu	1369141.04	986645.4864	1521476.736	1012033.372	1236236.305	1039018.917	1146457.927	683398.2562	1493452.891
NDUFS7	NADH dehydrogenase [ubiquinone] iron-sulfur protein 7, mitochondrial [Homo	799626.319	1224766.206	1312394.622	941930.0916	706190.5457	1241222.198	1791765.298	1171709.041	2089722.844
NDUFS8	NADH dehydrogenase [ubiquinone] iron-sulfur protein 8, mitochondrial precu	1967384.893	1978829.451	2455441.653	2242214.183	2342321.843	2188785.571	1920247.305	1956223.155	2141751.07
NDUFV1	NADH dehydrogenase [ubiquinone] flavoprotein 1, mitochondrial isoform 1 pr	575360.8985	624698.6753	724118.4768	664219.7335	746579.1433	722640.3572	576657.6026	456651.3905	587323.4588
NDUFV2	NADH dehydrogenase [ubiquinone] flavoprotein 2, mitochondrial precursor [Ho	996777.2926	881472.8691	1727897.774	446514.3432	1693380.412	481594.1947	503423.007	642645.6199	1155227.351
NDUFV3	NADH dehydrogenase [ubiquinone] flavoprotein 3, mitochondrial isoform a pre	436978.7372	217236.104	616569.7917	511354.6688	606762.9638	524712.5206	401760.3567	478457.4858	589721.4055
NECAP1	adaptin ear-binding coat-associated protein 1 [Homo sapiens]	603986.9033	439852.2938	714758.4346	464000.4081	823567.1	583520.3242	271484.1958	265750.1808	394167.16
NECAP2	adaptin ear-binding coat-associated protein 2 isoform 2 [Homo sapiens]	420216.8624	553276.8207	515002.0413	413481.4817	580967.4947	542504.7627	475269.2234	339181.3798	531211.7798
NEDD1	protein NEDD1 isoform a [Homo sapiens]	55297.68384	35004.97414	59342.63685	49655.74841	82292.16022	43523.3619	0	0	0
NEDD4	E3 ubiquitin-protein ligase NEDD4 isoform 1 [Homo sapiens]	81333.2887	127827.1117	605685.679	115536.5881	215651.9421	191841.7107	113232.0682	139768.8086	157532.7525
NEDD4L	E3 ubiquitin-protein ligase NEDD4-like isoform 1 [Homo sapiens]	0	0	0	0	0	0	22281.55759	5576.807179	19239.48586
NEDD8	NEDD8 precursor [Homo sapiens]	570582.5703	358713.8375	658995.4438	0	427772.3625	391735.5188	0	0	0
NEDD8-MDP1	NEDD8-MDP1 protein [Homo sapiens]	90631.758	87663.96221	206130.0116	3403603.935	217319.4335	159678.766	3276225.338	2888398.614	0
NEFH	neurofilament heavy polypeptide [Homo sapiens]	82966.39261	73700.80837	61788.40479	80757.86089	69511.64333	130986.46	6461.914903	30034.34132	44315.36138

NEK7	serine/threonine-protein kinase Nek7 [Homo sapiens]	211078.8512	550232.2067	593659.8117	561125.2645	236019.5496	651990.0919	667249.4797	371160.152	696555.1094
NEK9	serine/threonine-protein kinase Nek9 [Homo sapiens]	76662.91918	99097.7947	158450.9279	143332.278	160855.9775	138696.1793	88374.72161	88688.55012	100377.5015
NELFB	negative elongation factor B [Homo sapiens]	448962.2716	467454.6077	402289.2136	349630.405	467542.0757	542406.0036	311504.0437	357342.14	404259.8483
NELFCD	negative elongation factor C/D [Homo sapiens]	509794.3798	428413.3735	601901.461	564312.9061	605643.7866	521198.8735	455064.6919	385101.8547	417065.7064
NEMF	nuclear export mediator factor NEMF [Homo sapiens]	47303.02796	122903.6632	150658.534	92007.56295	62254.84386	91298.08271	107429.5426	90237.17733	145391.0248
NENF	neudesin precursor [Homo sapiens]	1858541.818	2512896.742	2816334.058	1727329.153	2230845.152	1702971.558	2296091.777	1579087.11	2798224.483
NES	nestin [Homo sapiens]	37479.32405	52545.74712	56979.31804	70526.58371	88657.4357	103076.0214	50921.27712	46410.85567	59260.31423
NEU1	sialidase-1 precursor [Homo sapiens]	37865.95177	196062.2638	385478.8054	134665.9124	0	50264.19046	0	0	0
NF1	neurofibromin isoform 1 [Homo sapiens]	205134.2521	16554.90217	47566.9692	14782.32728	24696.39148	11508.16206	17670.79354	35933.6862	241934.3852
NF2	merlin isoform 1 [Homo sapiens]	0	0	0	0	0	0	2989685.739	75557.78909	104118.184
NFAT5	nuclear factor of activated T-cells 5 isoform b [Homo sapiens]	63969.39722	68952.76019	102378.1	68244.81481	108289.9407	57635.56159	70821.82119	53516.80393	83989.08333
NFATC2IP	NFATC2-interacting protein [Homo sapiens]	210443.1074	249211.5581	204750.2645	222111.7137	318964.0676	155394.4037	136927.4819	119393.9784	132172.9691
NFIC	nuclear factor 1 C-type isoform 1 [Homo sapiens]	295174.1679	497247.0847	837685.7373	368287.9393	869124.1599	635353.8333	530747.2382	355415.1668	588973.6994
NFKB1	nuclear factor NF-kappa-B p105 subunit isoform 1 [Homo sapiens]	224834.192	228491.6454	252528.4739	197919.4522	250653.6305	286950.4067	230348.0786	84759.0566	255076.9174
NFKB2	nuclear factor NF-kappa-B p100 subunit isoform a [Homo sapiens]	234152.2662	267908.5216	400025.3047	269848.8263	256604.0605	329224.7123	329559.1011	466867.3976	360423.6868
NFKBIB	NF-kappa-B inhibitor beta isoform 1 [Homo sapiens]	208122.4388	206531.0675	135346.0327	262404.4916	252715.2897	187318.6007	177730.697	0	209807.2993
NFRKB	nuclear factor related to kappa-B-binding protein isoform 2 [Homo sapiens]	22794.31644	6166.466709	27084.18892	20761.11304	26492.72917	23052.75239	5490.320806	0	27018.74099
NFS1	cysteine desulfurase, mitochondrial isoform a precursor [Homo sapiens]	488427.4598	499779.8665	570533.0786	454553.9667	782610.3862	471148.7378	531744.8773	425338.0647	473200.203
NFU1	NFU1 iron-sulfur cluster scaffold homolog, mitochondrial isoform 2 [Homo sa	807876.5503	2421880.976	1728802.944	1420119.384	1522294.802	847614.1667	1374900.618	1117235.627	1525476.542
NFX1	transcriptional repressor NF-X1 isoform 1 [Homo sapiens]	0	24006.97916	41383.38599	21697.80548	47263.63957	22274.09714	0	0	0
NFXL1	NF-X1-type zinc finger protein NFXL1 [Homo sapiens]	33060.82701	36674.599	40381.62739	23968.55175	33374.51596	0	25473.47562	0	24498.3756
NFYA	nuclear transcription factor Y subunit alpha isoform 1 [Homo sapiens]	894601.5909	1025072.255	1981897.127	1683663.4	2237521.042	1962368.162	1262589.709	1175198.3	1550043.155
NFYB	nuclear transcription factor Y subunit beta [Homo sapiens]	840878.3555	379662.3682	918631.5516	866298.098	1100435.393	1044759.859	1230844.253	487863.4945	639523.7386
NFYC	nuclear transcription factor Y subunit gamma isoform 1 [Homo sapiens]	854627.7991	827838.882	765558.6379	84616.26235	1057319.198	557688.014	796343.3442	871324.1481	931422.6531
NGDN	neuroguidin isoform 1 [Homo sapiens]	951743.4765	889438.1324	1105550.44	803642.7463	1027734.621	877854.4309	763500.1382	657743.9765	607830.487
NGRN	neugrin precursor [Homo sapiens]	0	387560.4688	0	240226.3927	447255.8542	205767.7995	0	160944.973	285171.6572
NHLRC2	NHL repeat-containing protein 2 [Homo sapiens]	214792.8212	284713.9462	210383.4396	306423.1435	357693.5957	297900.4499	204417.1707	258537.501	249931.5179
NHP2	H/ACA ribonucleoprotein complex subunit 2 isoform a [Homo sapiens]	4076773.195	3001208.289	5163258.585	3582307.057	4917897.652	4854742.18	4449155.164	5328484.074	3617586.475
NHP2L1	NHP2-like protein 1 [Homo sapiens]	17254729.36	21563512.84	22458442.48	22541949.5	25891984.66	21329232.05	21883022.59	17843249.25	21984433.09
NIF3L1	NIF3-like protein 1 isoform 1 [Homo sapiens]	1123877.399	1034302.214	1479977.623	1316074.28	1596132.557	1239460.735	1172566.136	998159.4842	1168611.19
NIP7	60S ribosome subunit biogenesis protein NIP7 homolog isoform 1 [Homo sapier	1576252.803	1348352.116	2615843.971	2511803.037	2763468.795	1821230.203	2480603.524	1475190.07	1983661.596
NIPBL	nipped-B-like protein isoform A [Homo sapiens]	28788.65497	30267.76226	34238.77002	39424.59514	36607.11723	37599.21857	34586.31838	27581.42907	58523.83194
NIPSNAP1	protein NipSnap homolog 1 isoform 1 [Homo sapiens]	1647812.545	2103887.933	1909080.719	2027303.005	2300398.081	2168000.728	1986330.927	1380333.178	2108182.152
NIPSNAP3A	protein NipSnap homolog 3A [Homo sapiens]	248080.9363	507087.6263	346521.149	444287.3479	371937.4545	271219.9495	355083.6795	278806.5051	384223.48
NISCH	nischarin isoform 1 [Homo sapiens]	0	0	0	0	0	0	0	0	0
NIT1	nitrilase homolog 1 isoform 4 [Homo sapiens]	0	351016.9269	234129.0347	0	291059.2083	258372.8616	251045.5112	173853.4492	265184.6883
NIT1	nitrilase homolog 1 isoform 1 [Homo sapiens]	0	351016.9269	234129.0347	0	291059.2083	258372.8616	251045.5112	173853.4492	265184.6883
NIT2	omega-amidase NIT2 [Homo sapiens]	2926021.066	3265344.12	3961156.267	3259747.137	4106464.695	3510253.089	3378138.156	2993223.391	3543261.96
NKRF	NF-kappa-B-repressing factor isoform 1 [Homo sapiens]	711547.3441	295071.3392	322631.6575	354225.8962	372271.3971	836372.0984	824681.0224	262442.5521	279556.0848
NKTR	NK-tumor recognition protein [Homo sapiens]	48717.53046	31001.2647	69517.6962	27027.99139	13618.66258	0	21777.77583	28317.98907	32708.16155
NLE1	notchless protein homolog 1 isoform a [Homo sapiens]	574918.092	627284.828	795461.7611	659527.8874	751241.7966	641097.2874	500161.9812	409798.6219	365800.9445
NLN	neurolysin, mitochondrial [Homo sapiens]	273255.7173	254007.9927	352499.2236	121972.1501	377162.5081	264088.576	236094.1714	152131.7555	286674.7852
NMD3	60S ribosomal export protein NMD3 [Homo sapiens]	1079059.483	1044015.713	2767158.259	2475251.382	1373856.213	1123814.637	1197926.884	1942284.319	1242513.332
NME1	nucleoside diphosphate kinase A isoform a [Homo sapiens]	5580322.617	3199394.742	5936378.723	4934173.944	5519392.808	953664.7227	1001931.464	2945874.405	3169567.897
NME1-NME2	NME1-NME2 protein [Homo sapiens]	20234326.23	48886255.39	35950998.39	32298647.46	34618726.38	32915412.52	59378062.55	41093252.69	50524170.45
NME3	nucleoside diphosphate kinase 3 precursor [Homo sapiens]	703133.619	663696.8996	614031.0385	1430252.445	667587.7808	997521.6484	1438821.097	995281.9729	829029.1678
NME7	nucleoside diphosphate kinase 7 isoform a [Homo sapiens]	150285.7329	82166.97007	174227.9658	239258.4867	0	213103.8057	0	143264.0834	178183.2312
NMI	N-myc-interactor [Homo sapiens]	554509.9635	733078.1986	747308.1036	689017.0555	597990.4958	732038.7173	660369.4856	525741.3699	573306.5159
NMRAL1	nmrA-like family domain-containing protein 1 [Homo sapiens]	326054.4417	226635.3958	434343.3833	423461.6587	534388.134	382429.1353	480244.9848	250455.9651	476941.8167
NMT1	glycylpeptide N-tetradecanoyltransferase 1 [Homo sapiens]	2553087.821	2680938.053	3923750.217	2982680.243	3893502.565	2843514.446	2852493.406	2366095.051	3223346.494
NNMT	nicotinamide N-methyltransferase [Homo sapiens]	8227434.704	7480895.489	9461548.069	9607474.99	9564734.458	7704184.264	9068114.8	7513408.504	8492626.339
NNT	NAD(P) transhydrogenase, mitochondrial [Homo sapiens]	1537528.578	1610278.106	2063700.636	1916750.688	2337426.048	1942450.37	1913031.468	1552180.461	2051898.418
NOA1	nitric oxide-associated protein 1 [Homo sapiens]	75491.00117	68776.95436	83277.66844	27487.36621	186430.6015	74569.98986	90694.26241	53074.37167	72975.45553
NOB1	RNA-binding protein NOB1 [Homo sapiens]	841216.5331	837772.2385	1149895.351	901600.7609	1146732.066	942149.7172	763507.4166	764113.8751	883295.4278
NOC2L	nucleolar complex protein 2 homolog [Homo sapiens]	330100.9586	679758.6636	829169.0815	630236.0566	751439.2657	642623.6287	648606.6238	316383.0892	606111.7622
NOC3L	nucleolar complex protein 3 homolog [Homo sapiens]	347611.6073	366652.9474	435122.8013	343249.4	489210.6846	405710.5143	399390.0801	322622.2501	402283.6606
NOC4L	nucleolar complex protein 4 homolog [Homo sapiens]	682555.8586	487153.8604	848359.7743	535697.5173	746213.211	497951.5216	564107.9466	638336.0518	427611.2186
NOL10	nucleolar protein 10 isoform 1 [Homo sapiens]	213327.3874	185793.7447	220134.4578	181007.7884	349152.3538	217749.9241	175244.5307	456328.1285	188659.2139
NOL11	nucleolar protein 11 [Homo sapiens]	587107.1748	450604.1419	799555.7	715394.0715	786296.1313	669158.1346	726182.6041	678844.7256	931303.8089
NOL12	nucleolar protein 12 [Homo sapiens]	176354.615	0	498149.2146	117465.4469	353179.6858	0	0	0	0
NOL3	nucleolar protein 3 isoform D [Homo sapiens]	2313927.928	2605603.369	2878359.638	2729034.585	2910098.725	2476206.389	2652188.366	2411883.2	2387967.919
NOL6	nucleolar protein 6 alpha isoform [Homo sapiens]	220188.1668	199747.219	265473.8345	232373.8923	275077.73	287080.3428	239825.1574	179988.8271	284932.366
NOL7	nucleolar protein 7 [Homo sapiens]	236593.1193	1059364.934	900975.1221	1184237.033	453273.1908	960817.5631	806923.1994	177079.4098	1000558.896
NOL8	nucleolar protein 8 isoform a [Homo sapiens]	16867.38046	41015.87325	19164.16103	33459.29879	31266.73397	31997.66427	30087.80696	40537.91276	40448.15393
NOL9	polynucleotide 5'-hydroxyl-kinase NOL9 [Homo sapiens]	303112.722	405184.8818	373383.6046	312922.3113	304728.5951	472701.3887	400120.8398	359349.0251	422145.2182
NOLC1	nucleolar and coiled-body phosphoprotein 1 [Homo sapiens]	1553351.045	1423009.536	2246727.404	1783921.32	2210113.758	1918597.257	1657273.622	962758.2873	1929864.492
NOM1	nucleolar MIF4G domain-containing protein 1 [Homo sapiens]	0	0	0	0	0	0	0	26607.52799	14519.90315
NOMO2	nodal modulator 2 isoform 1 precursor [Homo sapiens]	752863.9694	686910.0554	1023061.576	746327.4256	149297.9044	954013.5433	885040.8281	712329.6043	0
NONO	non-POU domain-containing octamer-binding protein isoform 1 [Homo sapiens]	9410498.563	10931529.74	12672437.93	10652592.44	11919195.14	11015488.88	10549657.73	8428546.274	11741713.26
NOP10	H/ACA ribonucleoprotein complex subunit 3 [Homo sapiens]	1435200.468	3373264.928	0	2738681.564	0	3201878.256	4244888.338	4426300.125	3135974.647
NOP14	nucleolar protein 14 [Homo sapiens]	189100.1607	289619.6319	262900.4188	193172.7341	377922.4897	326795.9884	319593.5625	274966.3442	224876.012
NOP16	nucleolar protein 16 isoform 3 [Homo sapiens]	1264987.174	1479378.37	1839122.58	1335079.397	1734652.464	1500861.142	1409447.374	971512.749	1801476.451
NOP2	putative ribosomal RNA methyltransferase NOP2 isoform 3 [Homo sapiens]	1214553.33	1230580.414	1616041.502	1251229.721	1822041.029	1266310.799	1104084.17	1252147.279	1389018.056
NOP56	nucleolar protein 56 [Homo sapiens]	2514174.017	2428783.33	3192688.434	2591332.27	3225862.18	2454988.302	2459890.028	2062169.278	2787965.81
NOP58	nucleolar protein 58 [Homo sapiens]	2026041.162	2436226.188	2735979.567	2435912.133	2996024.318	2694242.348	2610550.496	2211377.988	2666669.037
NOP9	nucleolar protein 9 [Homo sapiens]	231759.6758	312961.6166	332616.1429	307923.2152	281630.1776	252283.328	364489.0122	294082.1705	354408.6482
NOS1AP	carboxyl-terminal PDZ ligand of neuronal nitric oxide synthase protein isoi	34027.84745	38552.07325	99605.79032	228117.4276	112006.3659	141497.2612	39241.81099	0	102613.7979
NOSIP	nitric oxide synthase-interacting protein [Homo sapiens]	1759036.212	1851140.641	2381734.359	2011726.558	2436071.846	2080276.207	1344456.984	1258934.084	1333543.382
NOTCH2	neurogenic locus notch homolog protein 2 isoform 1 preproprotein [Homo sapi	0	0	0	0	0	0	0	16152.82406	39526.86379
NPC1	Niemann-Pick C1 protein precursor [Homo sapiens]	211437.276	178540.8695	347073.2095	113499.0855	387964.9824	256292.5481	282914.7388	94164.85752	138238.0632
NPC2	epididymal secretory protein E1 precursor [Homo sapiens]	6099192.112	2444564.482	4920131.608	3863679.341	4342262.606	2340434.9	3892580.149	3488970.839	5947920.908
NPEPL1	probable aminopeptidase NPEPL1 isoform 1 [Homo sapiens]	136770.8899	230224.6557	297142.2437	122263.0346	235976.9694	331688.4811	163038.298	205459.2136	0
NPEPPS	puromycin-sensitive aminopeptidase [Homo sapiens]	2887791.954	3013416.579	3912393.915	3120240.497	3846562.368	3514254.271	2726812.299	2853449.445	3069008.962
NPLOC4	nuclear protein localization protein 4 homolog [Homo sapiens]	905045.5467	944335.7181	1035971.141	979776.8024	962100.611	929484.8681	943537.9105	801823.8505	976677.331
NPM1	nucleophosmin isoform 1 [Homo sapiens]	105215440.4	121947716.5	147468723.8	117283129.6	137959966.7	130370973.3	122478001.2	99985830.51	129489503.2
NPM3	nucleoplasmin-3 [Homo sapiens]	6691622.346	5990357.375	9383872.398	6849161.613	7871690.69	4821857.563	4633007.275	5897950.021	7528480.227
NPRL2	nitrogen permease regulator 2-like protein [Homo sapiens]	0	15581.70291	37013.852	16001.7781	29043.79948	26739.4234	17488.24913	11805.78819	10084.22678
NPTN	neuroplastin isoform b precursor [Homo sapiens]	536435.9558	393183.3494	605071.3164	488148.6791	502903.1337	1190356.647	1090510.566	1012294.306	525217.083
NQO1	NAD(P)H dehydrogenase [quinone] 1 isoform a [Homo sapiens]	4370379.359	6587429.968	5574882.967	5932609.807	6250924.294	4405971.784	4954819.56	5002404.404	6437435.389
NQO2	ribosyldihydronicotinamide dehydrogenase [quinone] [Homo sapiens]	2333031.921	2567500.9	3205953.088	2328314.508	2516922.582	2587332.395	2521911.923	2293534.843	3023070.083
NR2C2AP	nuclear receptor 2C2-associated protein [Homo sapiens]	366510.1735	1559263.888	1215724.981	349066.2602	1372869.838	987851.8802	0	757857.1518	774494.7433
NR2F2	COUP transcription factor 2 isoform a [Homo sapiens]	143903.6441	115619.6291	160647.1608	207643.5082	166884.4809	122281.6973	177533.2159	138280.9619	127159.9666
NR3C1	glucocorticoid receptor isoform gamma [Homo sapiens]	103063.8867	112190.9147	129555.4145	136733.464	158133.103	116133.5172	86499.38046	96991.52185	102510.1651
NRAS	GTPase NRas [Homo sapiens]	1935041.181	3108234.521	4043929.841	2931804.159	3133237.661	3349229.873	3299223.557	2822895.177	4244251.15
NRBP1	nuclear receptor-binding protein [Homo sapiens]	740813.6887	860468.4101	1037707.705	776400.2629	1042461.707	788510.3987	640841.0465	628742.2747	621441.1336
NRD1	nardilysin isoform a precursor [Homo sapiens]	1635265.727	1630881.726	1768558.936	1374728.595	1991941.087	1827840.709	1458192.746	1288288.246	1965640.996
NRDE2	protein NRDE2 homolog [Homo sapiens]	0	42862.03788	18897.76894	73120.94833	30073.14976	44466.09307	63023.53801	11388.32224	47120.93554
NRF1	nuclear respiratory factor 1 [Homo sapiens]	161694.598	367725.0825	355346.2592	0	626899.1373	0	0	0	0
NRM	nurim isoform 2 [Homo sapiens]	0	527203.3547	564743.5946	595316.7703	706532.2741	426476.4065	705926.5405	486430.7132	741612.968
NSA2	ribosome biogenesis protein NSA2 homolog isoform 1 [Homo sapiens]	1033600.597	1270320.296	1205446.032	751972.9545	1014901.524	1282573.666	1018009.739	922142.0867	1079147.959
NSDHL	sterol-4-alpha-carboxylate 3-dehydrogenase, decarboxylating [Homo sapiens]	1082905.306	1535802.93	1844315.229	1875560.024	1735755.258	1874248.274	1812432.793	1474405.45	1901312.182
NSF	vesicle-fusing ATPase [Homo sapiens]	533462.5041	731784.3759	813336.174	636445.831	771568.7423	653573.7708	959626.7351	552648.727	861409.3991
NSFL1C	NSFL1 cofactor p47 isoform d [Homo sapiens]	1373318.007	1589326.992	1947186.99	1795738.343	1903465.293	1803195.458	1671617.795	1413043.59	1797565.426
NSL1	kinetochore-associated protein NSL1 homolog isoform 1 [Homo sapiens]	0	0	438819.8453	180822.4749	203837.1066	184646.6926	0	0	0
NSMAF	protein FAN isoform 2 [Homo sapiens]	18522.575	96211.25097	49943.62422	25736.70938	0	24353.68164	0	0	0
NSMCE2	E3 SUMO-protein ligase NSE2 [Homo sapiens]	0	0	308858.5036	36422.82311	348107.717	963905.4774	864495.0625	0	196928.5042
NSRP1	nuclear speckle splicing regulatory protein 1 isoform 1 [Homo sapiens]	0	0	0	0	0	0	0	17454.17857	66364.6
NSUN2	tRNA (cytosine(34)-C(5))-methyltransferase isoform 1 [Homo sapiens]	3258701.56	3796844.597	4191278.019	3461161.836	4238013.066	3595736.745	3913035.959	3383690.465	4053334.327
NSUN4	5-methylcytosine rRNA methyltransferase NSUN4 isoform a [Homo sapiens]	304214.2738	253131.8353	0	317712.3889	0	320170.8648	428753.4663	53073.66717	33879.10714
NSUN5	putative methyltransferase NSUN5 isoform 3 [Homo sapiens]	152193.0455	166228.7291	213665.2004	188318.0466	241003.6135	167251.4014	221186.45	118968.9285	163278.7501
NT5C	5'(3')-deoxyribonucleotidase, cytosolic type isoform 1 [Homo sapiens]	1172849.125	1165021.686	1223972.59	1153905.831	1365009.007	1144999.858	1008840.234	975449.4811	791185.495
NT5C1B-RDH14	NT5C1B-RDH14 protein isoform 1 [Homo sapiens]	50811.84151	67684.83208	48956.82098	0	96616.11415	0	0	0	0
NT5C2	cytosolic purine 5'-nucleotidase [Homo sapiens]	730752.4606	580114.1967	996185.369	776503.8512	1068238.183	948530.4169	814959.338	649518.2568	799950.931
NT5C3A	cytosolic 5'-nucleotidase 3 isoform 1 [Homo sapiens]	1026067.097	1446566.973	1466369.28	1275942.947	1620676.907	1533766.141	1298448.12	1051679.704	1084982.579
NT5DC1	5'-nucleotidase domain-containing protein 1 [Homo sapiens]	562700.6202	874057.8463	897532.236	867964.5903	1016492.333	620191.3829	896861.2114	594254.6817	1004824.262
NT5DC2	5'-nucleotidase domain-containing protein 2 isoform 1 [Homo sapiens]	90663.2122	73560.31668	132110.4301	124177.4596	167750.089	146947.7856	126342.5358	75678.66098	97877.12561
NT5E	5'-nucleotidase isoform 1 preproprotein [Homo sapiens]	114861.5565	74471.70328	99998.06439	88890.34551	153644.6061	106979.8662	91489.30177	94068.20106	152582.2498
NTHL1	endonuclease III-like protein 1 [Homo sapiens]	0	219966.3469	326905.5426	0	141127.4457	75642.23208	0	0	0
NTMT1	N-terminal Xaa-Pro-Lys N-methyltransferase 1 [Homo sapiens]	2198531.207	2649806.59	1628834.571	2388910.624	3140596.326	2567169.367	2349794.024	1985873.306	2514174.292
NTPCR	cancer-related nucleoside-triphosphatase [Homo sapiens]	1321804.014	1661981.599	1423740.986	1116212.246	1570711.059	1333574.096	1273942.731	930181.5733	1630716.704

NUB1	NEDD8 ultimate buster 1 isoform 1 [Homo sapiens]	0	24075.28571	81171.30604	46371.00804	84720.12279	56156.36161	43282.03707	44093.25625	46501.56964
NUBP1	cytosolic Fe-S cluster assembly factor NUBP1 isoform 1 [Homo sapiens]	883879.8342	636374.6692	1121493.887	1124231.029	1149964.952	1229008.145	1035235.757	1036802.179	1069998.767
NUBP2	cytosolic Fe-S cluster assembly factor NUBP2 isoform 1 [Homo sapiens]	3984556.629	1560200.435	2498823.467	2171317.235	2386315.898	1801851.962	1908826.73	2983017.983	1518968.704
NUCB1	nucleobindin-1 precursor [Homo sapiens]	1135211.97	1179512.699	1185672.261	1383584.644	1243677.109	1454290.607	1287863.787	953567.99	1301206.359
NUCB2	nucleobindin-2 precursor [Homo sapiens]	426703.7651	502520.0221	680550.504	576509.2166	367163.5377	407802.4808	563089.1633	488646.0572	351994.8972
NUCKS1	nuclear ubiquitous casein and cyclin-dependent kinase substrate 1 [Homo sa	957731.0387	2766629.189	1483879.822	1162818.877	1548429.779	1031906.143	1208485.075	1243293.922	1674293.903
NUDC	nuclear migration protein nudC [Homo sapiens]	4990701.629	5796962.624	6417709.635	5400324.26	6447753.503	5657694.644	5521195.599	4791895.107	6286761.172
NUDCD1	nudC domain-containing protein 1 isoform 1 [Homo sapiens]	661744.3945	997708.024	1270076.326	669157.882	1238149.029	884695.2385	836287.2575	665760.352	599842.3805
NUDCD2	nudC domain-containing protein 2 [Homo sapiens]	2346908.92	2495297.266	4333866.132	3394981.766	3654115.157	3940145.086	3431112.8	2982723.464	3523207.232
NUDCD3	nudC domain-containing protein 3 [Homo sapiens]	279932.9939	372418.4441	522661.1871	392423.7993	537471.4195	398641.1922	394823.5295	337471.3472	465257.3475
NUDT1	7,8-dihydro-8-oxoguanine triphosphatase isoform p22 [Homo sapiens]	1428254.37	1937840.911	1549884.807	1801927.549	2448050.908	2118894.33	1414137.22	1823382.97	3166968.016
NUDT15	probable 8-oxo-dGTP diphosphatase NUDT15 [Homo sapiens]	821067.2006	1080597.177	1312077.021	1161689.962	1163458.869	976729.3029	970055.4271	1046538.762	698173.6925
NUDT16	U8 snoRNA-decapping enzyme isoform 2 [Homo sapiens]	996938.6661	1145384.826	797985.0049	1349488.417	1032334.922	898432.6039	1251873.438	1089428.572	1452893.075
NUDT19	nucleoside diphosphate-linked moiety X motif 19, mitochondrial precursor [	245819.4847	256264.6187	351244.8853	242534.8114	191940.5417	338305.1625	256060.1467	282520.385	286612.3466
NUDT2	bis(5'-nucleosyl)-tetraphosphatase [asymmetrical] [Homo sapiens]	0	1151524.451	912382.4255	1172885.554	852808.3143	825360.8661	868442.8438	589853.7366	728887.6229
NUDT21	cleavage and polyadenylation specificity factor subunit 5 [Homo sapiens]	4283660.768	4573000.3	6604592.588	5302204.563	6710816.209	5464215.346	3828864.26	4336657.859	5328132.953
NUDT5	ADP-sugar pyrophosphatase [Homo sapiens]	7065845.424	10294073.08	8995091.931	9400944.029	9442486.669	10627490.54	9542618.995	8221288.311	10557329.78
NUDT9	ADP-ribose pyrophosphatase, mitochondrial isoform a precursor [Homo sapiens	185040.8145	266509.5983	311862.2107	281965.5255	122538.3239	336000.2044	226499.0849	261382.2075	211934.1871
NUF2	kinetochore protein Nuf2 [Homo sapiens]	136481.2468	0	201159.3453	151901.6311	188821.612	99101.62924	167686.0549	98758.90506	131121.365
NUFIP2	nuclear fragile X mental retardation-interacting protein 2 [Homo sapiens]	725770.6629	880734.6121	1265248.211	939154.0664	1471098.326	883406.3069	858910.5478	687219.1948	722213.1302
NUMA1	nuclear mitotic apparatus protein 1 [Homo sapiens]	304869.0366	309153.2736	420511.158	323538.5226	416681.6073	322506.683	332865.4085	285951.7291	389432.4695
NUMB	protein numb homolog isoform 1 [Homo sapiens]	154636.0248	163556.6071	233981.9395	163183.7288	252765.4117	86444.09505	110395.4713	133300.1784	85286.01514
NUMBL	numb-like protein [Homo sapiens]	24148.41403	39799.77054	0	40371.21244	292137.4308	26368.9199	42653.43016	27169.19073	129775.6221
NUP107	nuclear pore complex protein Nup107 [Homo sapiens]	334066.4949	342484.3037	397206.9927	370637.3847	386943.8879	401825.2972	363577.237	298183.4418	352807.8116
NUP133	nuclear pore complex protein Nup133 [Homo sapiens]	432858.4834	398186.8033	490365.8779	455513.5022	594509.6308	486537.5659	459946.9381	438238.3049	407778.2948
NUP153	nuclear pore complex protein Nup153 isoform 2 [Homo sapiens]	331639.846	412374.5339	479411.5934	409328.2119	0	418183.0683	423527.4665	344558.0077	413363.2207
NUP155	nuclear pore complex protein Nup155 isoform 1 [Homo sapiens]	785287.338	874491.0063	1044699.544	916181.1174	1163866.397	915695.4359	897116.6118	707955.9493	877105.8444
NUP160	nuclear pore complex protein Nup160 [Homo sapiens]	475939.4471	470211.1499	633014.1842	522546.2219	592450.2367	441218.8962	504922.2254	440481.2478	518739.1875
NUP188	nucleoporin NUP188 homolog [Homo sapiens]	174050.0615	135160.7204	223750.2119	166581.3694	212654.4631	176669.314	104868.6451	111600.85	185118.6337
NUP205	nuclear pore complex protein Nup205 [Homo sapiens]	241186.816	277734.0749	315533.389	276776.928	306578.8768	265918.3632	277800.7082	238195.4067	268911.256
NUP210	nuclear pore membrane glycoprotein 210 precursor [Homo sapiens]	513909.2448	541861.707	717166.3623	535947.2509	791209.8221	558111.1461	519991.0301	430131.5126	550487.8008
NUP214	nuclear pore complex protein Nup214 [Homo sapiens]	282691.8087	284467.5549	354635.4186	290642.6615	387498.803	348245.9613	300886.2492	289571.4719	277561.3036
NUP35	nucleoporin NUP53 [Homo sapiens]	1879006.786	1879496.582	1795795.406	1945989.602	1813727.517	2092430.759	1687463.567	1324160.151	1738615.758
NUP37	nucleoporin Nup37 [Homo sapiens]	1219035.538	1288349.433	1384285.946	1807895.995	1964909.151	1128750.485	1050564.512	1541217.659	1709397.317
NUP43	nucleoporin Nup43 [Homo sapiens]	1152986.611	1589341.928	1418365.999	1538367.56	1309122.3	1528454.444	1710956.914	1220623.73	1784861.962
NUP50	nuclear pore complex protein Nup50 isoform b [Homo sapiens]	700354.0495	992023.2661	1128933.115	1018838.389	1152224.837	972291.7737	860308.6656	840169.8516	1203366.388
NUP54	nucleoporin p54 isoform 1 [Homo sapiens]	587301.5667	654340.5891	783257.9836	600591.8615	810649.5309	712387.2315	717993.3709	570565.1853	1010765.813
NUP62	nuclear pore glycoprotein p62 [Homo sapiens]	2688253.33	2658740.304	3505920.23	2556725.26	3572539.464	3017701.445	2332413.171	2358751.729	2953237.623
NUP85	nuclear pore complex protein Nup85 [Homo sapiens]	541393.3961	678402.1636	747986.9472	637954.6143	765578.3022	654849.9948	462274.5727	451941.9022	663666.8446
NUP88	nuclear pore complex protein Nup88 [Homo sapiens]	566805.2838	635107.0106	661780.9525	612168.0902	736875.3887	567096.0751	491171.8332	512871.8933	621367.6762
NUP93	nuclear pore complex protein Nup93 isoform 1 [Homo sapiens]	831691.1854	903533.1726	1070036.477	821061.9128	1199289.879	990180.6799	935839.3243	795280.0365	1105639.15
NUP98	nuclear pore complex protein Nup98-Nup96 isoform 1 [Homo sapiens]	358478.9076	392932.5164	1409066.824	1280493.208	1478931.016	1301491.805	1177917.908	1577693.247	302152.2872
NUPL1	nucleoporin p58/p45 isoform a [Homo sapiens]	723823.5657	1049242.366	963222.4685	1202878.63	1052381.489	1242286.362	1168594.323	802441.5977	911144.0831
NUPL2	nucleoporin-like protein 2 [Homo sapiens]	643703.4023	389475.0989	318268.0953	666043.5038	339189.8878	695665.1335	499352.3985	424113.8383	427131.3271
NUSAP1	nucleolar and spindle-associated protein 1 isoform 1 [Homo sapiens]	262739.7003	263987.0737	374420.6789	269064.3305	413062.9613	279393.1781	191285.5469	173623.4265	184559.4179
NUTF2	nuclear transport factor 2 [Homo sapiens]	23748144.02	26605526.67	33123744.52	28799238.06	31357829.84	29005485.4	29203217.14	24497120.45	27959672.44
NVL	nuclear valosin-containing protein-like isoform 1 [Homo sapiens]	136603.9758	171695.7484	199010.0059	169922.7693	196815.3299	173081.9757	151077.8634	83424.02338	157369.7634
NXF1	nuclear RNA export factor 1 isoform 1 [Homo sapiens]	499397.2344	520503.7141	792291.7118	563623.8882	667316.7214	536028.2244	495785.7443	423688.729	574948.5622
NXT1	NTF2-related export protein 1 [Homo sapiens]	1299648.25	0	1536158.224	1219418.658	1263623.118	0	0	0	0
OARD1	O-acetyl-ADP-ribose deacetylase 1 [Homo sapiens]	262746.6575	453167.4212	358277.9521	206357.823	371109.1233	197938.4538	181068.3074	150443.2858	288500.589
OAS3	2'-5'-oligoadenylate synthase 3 [Homo sapiens]	331395.0346	375826.5894	484838.9472	362087.6326	494565.5616	460048.5452	410250.846	335533.996	459778.2525
OASL	2'-5'-oligoadenylate synthase-like protein isoform a [Homo sapiens]	448635.7149	572477.5558	466701.5546	581000.0228	668091.5091	629701.1102	613240.8345	466859.753	611845.4609
OAT	ornithine aminotransferase, mitochondrial isoform 1 precursor [Homo sapiens	1658791.714	1540724.727	2377052.099	1344122.719	1482322.135	1864488.977	1462937.117	1282960.304	1749924.167
OCIAD1	OCIA domain-containing protein 1 isoform 4 [Homo sapiens]	1953768.955	1783465.22	2150653.072	1951971.51	2222786.968	2226390.513	1963343.133	1753900.479	2026148.388
OCIAD2	OCIA domain-containing protein 2 isoform 1 [Homo sapiens]	0	389368.5684	562741.4312	370564.799	537038.5761	298868.4936	323460.9529	204326.587	254479.3787
OCRL	inositol polyphosphate 5-phosphatase OCRL-1 isoform a [Homo sapiens]	120199.2596	110001.3967	19288.3647	183332.9498	854598.2659	170744.1743	86167.5189	140600.8622	101266.5342
OGDH	2-oxoglutarate dehydrogenase, mitochondrial isoform 1 precursor [Homo sapie	1163319.81	1179160.613	1546492.61	1263788.815	1624400.814	1259438.229	1294946.074	1014957.198	1145897.226
OGFOD1	2-oxoglutarate and iron-dependent oxygenase domain-containing protein 1 [Ho	362995.0104	301493.7519	502227.7737	491273.8002	529100.8959	435463.0123	427445.7326	347777.2153	249281.0226
OGFR	opioid growth factor receptor [Homo sapiens]	355782.2725	369854.4678	383233.7178	438797.7278	431597.968	366503.2953	333521.6494	356911.3735	404267.3942
OGT	UDP-N-acetylglucosamine--peptide N-acetylglucosaminyltransferase 110 kDa su	717262.013	696789.848	698425.3945	596681.511	480115.3173	469225.6561	338779.7335	329858.4194	589042.5803
OLA1	obg-like ATPase 1 isoform 1 [Homo sapiens]	5102889.978	5991425.045	6582361.124	6219837.249	7010122.175	6160421.757	5942494.136	4834911.594	5554787.897
OPA1	dynamin-like 120 kDa protein, mitochondrial isoform 7 [Homo sapiens]	412937.2347	0	578582.0653	421996.6852	551296.5102	350847.7809	381950.9097	0	316839.0958
OPA3	optic atrophy 3 protein isoform a [Homo sapiens]	0	0	0	0	0	0	265837.6061	0	369557.5606
OPTN	optineurin [Homo sapiens]	145200.8321	30197.36372	0	46760.55235	0	27156.60108	0	0	0
OR1M1	olfactory receptor 1M1 [Homo sapiens]	712279.8889	926980.716	0	772738.5417	662573.6158	901504.1728	1168270.999	1384766.981	810016.5432
ORC2	origin recognition complex subunit 2 [Homo sapiens]	332090.7838	221672.7063	291272.7009	353464.8944	476725.8537	309551.6674	250437.4438	179786.2536	251310.3668
ORC3	origin recognition complex subunit 3 isoform 1 [Homo sapiens]	198299.1252	190241.1981	265053.1616	197638.7781	266453.387	220835.949	183358.3974	143215.9539	146506.8399
ORC4	origin recognition complex subunit 4 isoform 1 [Homo sapiens]	214529.736	119349.1134	234103.8996	260217.5166	213965.2838	236383.3289	230743.4063	186062.7593	194013.5549
ORC5	origin recognition complex subunit 5 isoform 1 [Homo sapiens]	279602.4502	170000.7452	304662.2627	260520.2173	242287.9169	178362.288	102336.9665	177212.7292	204708.9628
ORMDL1	ORM1-like protein 1 [Homo sapiens]	0	0	0	0	0	0	626591.4878	403911.9878	0
ORMDL2	ORM1-like protein 2 [Homo sapiens]	2650780.639	2022374.171	1724174.743	0	2211745.51	278111.1341	2177588.22	63968.73476	2655435.637
ORMDL3	ORM1-like protein 3 [Homo sapiens]	0	0	0	0	0	0	300971.8537	161778.9085	0
OS9	protein OS-9 isoform 1 precursor [Homo sapiens]	44644.77251	0	62628.64113	66494.51364	46499.52644	0	0	0	0
OSBP	oxysterol-binding protein 1 [Homo sapiens]	407724.9731	1886957.642	1240957.971	490035.3836	1338829.616	647289.522	615464.4065	439748.2372	618311.9245
OSBPL10	oxysterol-binding protein-related protein 10 isoform 1 [Homo sapiens]	183005.5421	183195.1583	159993.1164	122137.1941	233818.2775	142628.9905	176715.9238	148759.7943	186966.3978
OSBPL11	oxysterol-binding protein-related protein 11 [Homo sapiens]	97748.99684	119432.6315	169235.7117	128707.1455	131841.2389	116557.4218	37352.70091	91976.51029	137770.1868
OSBPL1A	oxysterol-binding protein-related protein 1 isoform 2 [Homo sapiens]	0	0	0	0	0	0	11330.0243	12654.1883	0
OSBPL2	oxysterol-binding protein-related protein 2 isoform 2 [Homo sapiens]	19671551.32	121951.3973	58528.35811	23303039.99	0	68579.58681	114389.0189	115202.8733	60446.56557
OSBPL3	oxysterol-binding protein-related protein 3 isoform a [Homo sapiens]	20350.75362	12697.83039	0	3809.150183	79336.33962	7532.951812	14772.45489	48595.99846	0
OSBPL8	oxysterol-binding protein-related protein 8 isoform a [Homo sapiens]	205000.6109	185137.6983	258337.8296	220498.2397	245246.8344	236429.5743	223126.5711	172138.3549	177354.1488
OSBPL9	oxysterol-binding protein-related protein 9 isoform e [Homo sapiens]	379256.313	389123.5244	457876.2868	445650.2252	468345.4508	402540.6919	375635.2912	332774.3369	440323.9942
OSGEP	probable tRNA threonylcarbamoyladenosine biosynthesis protein OSGEP [Homo s	367301.9888	570203.8713	698298.5374	462117.362	782319.8451	467720.3791	873645.6053	358842.9579	776197.09
OSGEPL1	probable tRNA threonylcarbamoyladenosine biosynthesis protein OSGEPL1 [Homo	0	0	0	0	0	0	72306.08864	17829.4966	65795.32313
OSTC	oligosaccharyltransferase complex subunit OSTC isoform 3 [Homo sapiens]	23585173.33	17929562.06	10634948.66	191442.4314	4747159.267	273988.1269	297059.3849	22525765.82	9141784.108
OSTF1	osteoclast-stimulating factor 1 [Homo sapiens]	1304424.607	1969056.774	1578795.917	1725447.208	1664338.39	1967174.641	1772613.536	1303984.293	2522857.684
OTUB1	ubiquitin thioesterase OTUB1 [Homo sapiens]	6102571.222	5985326.47	8414485.011	6779637.412	8736315.984	6839796.533	6790622.111	5065343.325	6931985.511
OTUD4	OTU domain-containing protein 4 isoform 3 [Homo sapiens]	42594.26101	81162.50409	49094.65837	66493.2428	75477.33225	74668.57275	48875.7229	49965.94604	78660.575
OTUD5	OTU domain-containing protein 5 isoform a [Homo sapiens]	51519.13158	93959.21121	65552.73421	101028.0757	80870.02305	92806.92132	116816.8516	93136.43342	0
OTUD6B	OTU domain-containing protein 6B [Homo sapiens]	565407.2255	353000.8839	740020.3846	566910.7653	785751.9493	511083.0417	519466.4481	482859.03	443285.2426
OTUD7B	OTU domain-containing protein 7B [Homo sapiens]	38435.3919	78085.5991	46481.84872	75026.3972	97978.61579	53922.24729	42719.52012	74796.09969	67668.60838
OVCA2	ovarian cancer-associated gene 2 protein [Homo sapiens]	927009.3915	2045637.586	1750394.223	1193796.411	1731888.662	1593177.797	1118321.728	850406.7196	1689521.731
OXA1L	mitochondrial inner membrane protein OXA1L [Homo sapiens]	162338.8917	410693.2609	190045.7707	259027.944	242610.0505	312753.6201	211733.4674	120457.1966	303205.1671
OXCT1	succinyl-CoA:3-ketoacid coenzyme A transferase 1, mitochondrial precursor [	3887430.478	4791446.422	5299242.097	4946254.901	5399205.662	4977967.475	5104880.451	4439900.054	4999104.791
OXNAD1	oxidoreductase NAD-binding domain-containing protein 1 precursor [Homo sapi	158811.0066	0	178025.4395	156282.264	234023.1194	0	0	0	0
OXR1	oxidation resistance protein 1 isoform 3 [Homo sapiens]	28290.88798	3030.821484	35105.58234	24180.69232	51335.82309	26504.70289	24028.81658	0	26735.09885
OXSM	3-oxoacyl-[acyl-carrier-protein] synthase, mitochondrial isoform 1 precurso	179440.6224	173985.8997	163182.9694	175759.9803	185415.9057	245495.1973	265505.2145	217687.1469	245479.5936
OXSR1	serine/threonine-protein kinase OSR1 [Homo sapiens]	1334797.575	1726472.104	1840612.41	1647829.994	1999288.753	1701998.081	1668982.142	1389517.265	1575324.448
P4HA1	prolyl 4-hydroxylase subunit alpha-1 isoform 2 precursor [Homo sapiens]	849852.9405	861464.2761	768664.5365	931111.6653	1063111.462	1115745.35	1040274.232	815520.2261	1119325.133
P4HA2	prolyl 4-hydroxylase subunit alpha-2 isoform 1 precursor [Homo sapiens]	77007.2127	0	0	90808.74515	90214.02965	55471.8058	82259.6686	38286.59333	0
P4HA2	prolyl 4-hydroxylase subunit alpha-2 isoform 2 precursor [Homo sapiens]	77007.2127	0	0	90808.74515	90214.02965	55471.8058	82259.6686	38286.59333	0
P4HB	protein disulfide-isomerase precursor [Homo sapiens]	6849110.187	7564438.196	8524905.863	7337748.89	9233419.808	7822395.918	8046462.808	7001578.073	8329867.027
P4HTM	transmembrane prolyl 4-hydroxylase isoform c [Homo sapiens]	0	102717.9006	106374.1572	203937.345	225530.3713	84716.30117	168750.0365	208700.0309	75759.65351
PA2G4	proliferation-associated protein 2G4 [Homo sapiens]	15798621.01	14465529.81	21427053.92	16990395.3	22882402.02	17759904.37	17468840.04	14847052.73	18127323.21
PAAF1	proteasomal ATPase-associated factor 1 isoform 2 [Homo sapiens]	242480.0451	284003.6574	241789.7632	0	245576.4079	281872.5714	0	236581.1621	282741.3271
PABPC1	polyadenylate-binding protein 1 [Homo sapiens]	6019130.425	7217655.806	7825311.172	7483707.201	7652998.597	7107537.989	7140860.835	5864584.59	5875937.934
PABPC4	polyadenylate-binding protein 4 isoform 1 [Homo sapiens]	1669750.914	2129779.576	2148026.327	1987440.788	2148005.049	2082295.496	3310400.072	2907484.447	2361933.371
PABPN1	polyadenylate-binding protein 2 [Homo sapiens]	3915603.891	5793770.906	5369261.214	5370968.282	5444066.978	5783685.697	5297360.912	4771528.876	5252564.98
PACS1	phosphofurin acidic cluster sorting protein 1 [Homo sapiens]	81209.18544	102499.8711	78550.00988	76183.76883	95025.57532	94907.23823	88292.15541	67484.58625	108586.1682
PACSIN2	protein kinase C and casein kinase substrate in neurons protein 2 isoform	916198.4341	1033766.672	963932.275	835797.0394	1063462.461	861372.9423	851990.9909	814225.4889	1122312.819
PACSIN3	protein kinase C and casein kinase substrate in neurons protein 3 [Homo sa	666244.8357	1241005.617	769240.7791	758201.5148	833205.8867	805545.549	1002048.383	609701.0413	1096406.98
PADI1	protein-arginine deiminase type-1 [Homo sapiens]	91243.16563	165279.1365	0	159146.3625	141676.8854	0	0	0	0
PAF1	RNA polymerase II-associated factor 1 homolog isoform 1 [Homo sapiens]	428331.7126	500085.5802	535144.2226	432278.6828	580035.9717	606949.7892	636125.9613	284402.3762	624394.9259
PAFAH1B1	platelet-activating factor acetylhydrolase IB subunit alpha [Homo sapiens]	2470956.342	2720735.595	3034423.054	2391031.751	3250975.429	2492515.332	2678123.553	2163609.483	2678444.594
PAFAH1B2	platelet-activating factor acetylhydrolase IB subunit beta isoform a [Homo	7954528.279	6768776.031	9572657.788	7671224.561	9757190.744	7589138.897	7046602.747	6128560.863	7125416.216
PAFAH1B3	platelet-activating factor acetylhydrolase IB subunit gamma [Homo sapiens]	2507616.295	2385371.757	2585826.333	2743657.684	2683808.939	2529896.768	2600012.741	1878425.581	3359825.519
PAGE1	P antigen family member 1 [Homo sapiens]	32409230.88	32446228.03	46172491.38	31581438.45	47131158.58	33912133	27395954.98	20812502.43	31923246.03
PAICS	multifunctional protein ADE2 isoform 2 [Homo sapiens]	8211476.072	8968259.012	9556410.114	0	10712280.04	9044486.976	8750056.807	7157785.096	9085694.807
PAIP1	polyadenylate-binding protein-interacting protein 1 isoform 1 [Homo sapiens	2292750.915	2786850.466	3346854.191	2773620.785	3276569.991	2620809.447	2732007.409	2366282.528	2954494.117
PAIP2	polyadenylate-binding protein-interacting protein 2 [Homo sapiens]	10276245.5	6077419.807	7764748.321	4695163.144	8604262.368	7479916.114	5623065.339	5522126.789	6184157.936
PAK1IP1	p21-activated protein kinase-interacting protein 1 [Homo sapiens]	721708.7589	764342.0702	901011.6488	683951.7393	814719.4833	583359.4768	746477.3375	466872.6922	770366.4661

PAK2	serine/threonine-protein kinase PAK 2 [Homo sapiens]	2041694.213	2525366.105	2891822.515	1706459.836	2844322.28	2003404.186	1865105.962	1883199.496	2399990.951
PAK4	serine/threonine-protein kinase PAK 4 isoform 1 [Homo sapiens]	233566.6788	105198.7524	679772.9905	228022.4861	0	158315.5124	46024.84565	182854.7054	89264.28099
PALLD	palladin isoform 4 [Homo sapiens]	171372.0802	146278.9585	488458.9289	220785.9861	218302.5465	218436.0478	225495.3029	164016.8327	26170.89496
PALLD	palladin isoform 3 [Homo sapiens]	171372.0802	146278.9585	488458.9289	220785.9861	218302.5465	218436.0478	225495.3029	164016.8327	26170.89496
PALM2-AKAP2	PALM2-AKAP2 protein isoform 1 [Homo sapiens]	15523.21187	39225.38249	15023.61962	0	39750.69199	0	0	0	0
PANK2	pantothenate kinase 2, mitochondrial isoform 1 preproprotein [Homo sapiens]	19931.30109	20346.33039	27798.80738	25757.80446	22475.77801	22769.34647	14240.49533	0	24149.01751
PANK4	pantothenate kinase 4 [Homo sapiens]	76653.16403	156442.0056	99729.37933	85327.0302	100779.7664	142526.4216	115997.0649	92601.62765	117106.0503
PANX1	pannexin-1 [Homo sapiens]	131194.2584	197052.7917	282382.1148	236829.8208	161097.2667	186750.8111	215740.1222	238813.4481	182870.8815
PAPOLA	poly(A) polymerase alpha isoform 1 [Homo sapiens]	249030.3557	319775.0504	388557.7676	307249.7473	410789.1644	705849.0626	263640.5122	266016.5163	325696.3221
PAPSS1	bifunctional 3'-phosphoadenosine 5'-phosphosulfate synthase 1 [Homo sapiens	366557.2439	401867.6868	400815.9759	483378.2543	449616.5089	452379.0984	341544.9696	364189.0098	416475.4502
PAPSS2	bifunctional 3'-phosphoadenosine 5'-phosphosulfate synthase 2 isoform b [Ho	263107.9147	437814.14	447440.1519	261862.6507	477091.8181	271268.4078	304116.3838	253527.3481	492738.5627
PARD3	partitioning defective 3 homolog isoform 1 [Homo sapiens]	9895.266892	19165.7784	10387.85357	13719.00107	16596.35473	16069.68159	12613.55464	0	16362.29276
PARG	poly(ADP-ribose) glycohydrolase [Homo sapiens]	86748.74282	69547.27053	114034.5134	43365.16976	108606.861	67233.96232	42414.44682	52393.07816	68792.78589
PARK7	protein DJ-1 [Homo sapiens]	26435560.05	28002068.3	35828941.77	28859985.38	32595763.7	32321694.17	28419702.16	27193718.83	31025713.66
PARN	poly(A)-specific ribonuclease PARN isoform 1 [Homo sapiens]	237285.5769	126394.8742	278354.4233	112856.5691	281895.4055	3042940.93	193127.9146	275206.6951	204979.3374
PARP1	poly [ADP-ribose] polymerase 1 [Homo sapiens]	1756281.002	1881454.21	2269826.298	1907072.342	2349975.565	2032503.176	1880127.307	1572753.666	1952611.971
PARP12	poly [ADP-ribose] polymerase 12 [Homo sapiens]	40125.91747	51528.48891	62380.91343	70741.33596	105954.5606	44747.23679	80679.31509	53015.6566	188840.9958
PARP14	poly [ADP-ribose] polymerase 14 [Homo sapiens]	41659.28152	49002.20414	53212.34127	43135.91501	68220.4968	50674.03226	44782.11816	32188.1164	57040.25155
PARP2	poly [ADP-ribose] polymerase 2 isoform 1 [Homo sapiens]	82171.60035	77019.72266	120105.9001	94327.30191	156137.5418	93488.98426	37521.19124	0	65565.52367
PARP4	poly [ADP-ribose] polymerase 4 [Homo sapiens]	64780.04895	39341.70233	36081.2379	47148.13734	55929.42365	52169.86545	33165.40844	25226.84266	25966.17701
PARP9	poly [ADP-ribose] polymerase 9 isoform a [Homo sapiens]	0	0	0	0	0	0	0	3607.667041	38559.44706
PARVA	alpha-parvin [Homo sapiens]	555427.2868	679124.2866	755006.6358	626217.714	781809.7799	791545.9101	738399.9458	556931.628	681230.3363
PARVB	beta-parvin isoform a [Homo sapiens]	76034.56833	0	130823.1317	120476.7231	0	68366.20287	35057.17	25694.9675	0
PATL1	protein PAT1 homolog 1 [Homo sapiens]	174287.9624	100529.2703	234633.3477	209494.9881	258931.2236	193066.3574	160807.779	54697.10616	139464.2307
PAWR	PRKC apoptosis WT1 regulator protein [Homo sapiens]	800902.8079	739399.6424	927206.7899	310036.2893	1154943.171	958811.8712	848509.8698	311583.274	536983.9188
PAXBP1	PAX3- and PAX7-binding protein 1 isoform 1 [Homo sapiens]	84515.48249	81717.71454	118005.0278	60954.03382	116520.0137	70098.9389	77767.89962	68816.57777	83037.52264
PAXIP1	PAX-interacting protein 1 [Homo sapiens]	0	0	0	0	0	0	120767.9594	137282.9992	54423.59288
PBK	lymphokine-activated killer T-cell-originated protein kinase [Homo sapiens]	1235609.02	1808208.599	2156357.301	1785231.947	2103078.245	1969373.016	1852705.626	1594314.147	1960726.24
PBRM1	protein polybromo-1 [Homo sapiens]	37386.97148	48326.27349	49828.94774	47883.6424	42110.5699	50263.35898	32668.26757	36143.34228	39194.54645
PBXIP1	pre-B-cell leukemia transcription factor-interacting protein 1 [Homo sapien	106599.5076	136107.1461	110348.3817	107992.5179	128475.6635	107643.5936	106390.569	81861.27218	49170.64339
PC	pyruvate carboxylase, mitochondrial precursor [Homo sapiens]	343244.9998	346166.1455	394025.322	364145.8708	373389.271	512462.0297	314135.6958	281193.209	340267.8109
PCBD1	pterin-4-alpha-carbinolamine dehydratase precursor [Homo sapiens]	6050349.186	4178063.709	9498019.17	7477264.256	8665550.67	6832995.547	4577316.514	6134392.695	5045921.644
PCBP1	poly(rC)-binding protein 1 [Homo sapiens]	27637390.63	31248333.27	33938293.94	31845004.95	35746142.93	33012970.81	32448698.28	29063373.08	31088434.13
PCBP2	poly(rC)-binding protein 2 isoform b [Homo sapiens]	4407547.43	0	5485103.735	4184778.868	5426717.789	0	4353988.474	0	4551481.98
PCBP2	poly(rC)-binding protein 2 isoform e [Homo sapiens]	4407547.43	0	5485103.735	4184778.868	5426717.789	0	4353988.474	0	4551481.98
PCBP2	poly(rC)-binding protein 2 isoform a [Homo sapiens]	4407547.43	0	5485103.735	4184778.868	5426717.789	0	4353988.474	0	4551481.98
PCBP2	poly(rC)-binding protein 2 isoform d [Homo sapiens]	4407547.43	0	5485103.735	4184778.868	5426717.789	0	4353988.474	0	4551481.98
PCCA	propionyl-CoA carboxylase alpha chain, mitochondrial isoform a precursor [H	0	0	0	0	0	0	0	0	0
PCCB	propionyl-CoA carboxylase beta chain, mitochondrial isoform 2 precursor [Ho	18686.97115	0	109599.6458	59190.25385	91859.76795	46743.26603	0	50701.40641	10715.1891
PCF11	pre-mRNA cleavage complex 2 protein Pcf11 [Homo sapiens]	37962.01009	128345.8886	43751.97054	45250.05899	41154.12228	45652.37144	39996.07312	50562.47994	51102.86927
PCGF6	polycomb group RING finger protein 6 isoform a [Homo sapiens]	0	0	0	0	0	0	109738.6329	96827.24016	0
PCID2	PCI domain-containing protein 2 isoform 2 [Homo sapiens]	329006.2458	358582.9969	492060.776	395879.6205	513497.8959	431146.2627	340336.3979	392047.5465	462382.0308
PCK2	phosphoenolpyruvate carboxykinase [GTP], mitochondrial isoform 1 precursor	690970.8227	948736.5286	890407.2134	402808.6467	657373.7118	505006.0441	468142.8227	877506.913	521229.5143
PCM1	pericentriolar material 1 protein [Homo sapiens]	13171.96358	22649.64875	15903.66821	27732.04503	0	16207.01511	14742.42947	27206.90878	11735.92748
PCMT1	protein-L-isoaspartate(D-aspartate) O-methyltransferase isoform 2 [Homo sap	4616045.503	5298280.202	5900253.444	4633492.255	6400057.214	5165829.104	4654821.377	4231244.591	5244531.805
PCNA	proliferating cell nuclear antigen [Homo sapiens]	14968591.7	14967261.36	18688557	14927341.51	20097805.96	16465818.66	14784111.29	12571311.06	16926013.02
PCNP	PEST proteolytic signal-containing nuclear protein [Homo sapiens]	3232812.351	3132458.748	3770806.065	3427087.578	4064753.85	3260826.148	3055743.719	2255038.339	2915335.364
PCNT	pericentrin [Homo sapiens]	11109.04221	2817.540765	6222.02805	8384.401779	7599.122632	5523.140956	3974.45808	6138.125296	0
PCSK9	proprotein convertase subtilisin/kexin type 9 preproprotein [Homo sapiens]	68829.16395	0	62317.46047	0	43619.37064	47262.78256	0	0	0
PCYOX1	prenylcysteine oxidase 1 precursor [Homo sapiens]	815990.2713	781195.4146	1100770.632	734756.7024	979031.3604	819394.5457	851968.3841	759323.3396	896360.4451
PCYT1A	choline-phosphate cytidylyltransferase A [Homo sapiens]	841591.7006	888469.5721	937555.4837	888655.8099	915261.6151	987246.3983	902881.911	774436.1483	736854.2198
PCYT2	ethanolamine-phosphate cytidylyltransferase isoform 1 [Homo sapiens]	237148.2483	222033.8542	224837.6515	213499.18	218256.4239	213854.0553	233212.187	177247.4434	247669.7508
PDAP1	28 kDa heat- and acid-stable phosphoprotein [Homo sapiens]	1801519.457	3059064.565	3308495.659	2829081.835	3476968.561	3077334.837	2806162.27	2875278.39	2798111.115
PDCD10	programmed cell death protein 10 [Homo sapiens]	3126445.375	3077012.469	4046736.5	3502283.095	4136553.022	2934926.651	3088042.189	3130791.179	2537902.903
PDCD11	protein RRP5 homolog [Homo sapiens]	204900.1893	186301.5363	267939.7143	231299.9887	292824.9207	207070.4192	170565.1434	187589.1693	187837.9323
PDCD2	programmed cell death protein 2 isoform 1 [Homo sapiens]	0	0	83161.26872	83061.31472	45950.4105	97648.23776	17598.1985	0	180633.239
PDCD2L	programmed cell death protein 2-like [Homo sapiens]	1323099.18	1058720.161	1680638.186	1180528.633	1461597.441	1322124.906	1154715.708	779357.5418	1166473.459
PDCD4	programmed cell death protein 4 isoform 1 [Homo sapiens]	1038792.755	1182682.533	1287352.689	1310575.726	1269681.767	1337916.426	0	752956.3531	860247.0543
PDCD5	programmed cell death protein 5 [Homo sapiens]	14193500.23	18050680.46	20393802.1	17506056.41	19151996.43	18627405.46	17695747.96	13888341.76	18388089.58
PDCD6	programmed cell death protein 6 isoform 1 [Homo sapiens]	18208399.16	22520774.78	10511866.39	13527054.46	6948870.396	16834066.07	14454174.27	11456016.41	18086094.17
PDCD6IP	programmed cell death 6-interacting protein isoform 1 [Homo sapiens]	478667.838	560444.2544	699972.7004	594439.3284	629083.1667	627626.0371	597679.9232	557405.6375	718363.7646
PDCD6IP	programmed cell death 6-interacting protein isoform 2 [Homo sapiens]	478667.838	560444.2544	699972.7004	594439.3284	629083.1667	627626.0371	597679.9232	557405.6375	718363.7646
PDCL	phosducin-like protein [Homo sapiens]	114033.0441	189858.8272	228461.7343	226586.5714	224898.4349	173709.9869	0	0	0
PDCL3	phosducin-like protein 3 [Homo sapiens]	1129700.017	1342398.884	1465508.327	1239722.575	1419276.871	1170220.122	1194364.793	1004375.625	1325312.325
PDE12	2',5'-phosphodiesterase 12 [Homo sapiens]	752334.0594	775381.4614	868895.0267	844401.8228	932711.7876	775010.501	786908.355	659350.85	804345.4713
PDE3A	cGMP-inhibited 3',5'-cyclic phosphodiesterase A isoform 1 [Homo sapiens]	191341.7551	266299.4131	320992.6807	286088.4036	268240.1004	298411.4103	234819.1352	210295.2108	236771.9339
PDE3B	cGMP-inhibited 3',5'-cyclic phosphodiesterase B [Homo sapiens]	35626.40678	0	92254.49364	128695.1644	38783.79661	53633.55791	0	0	0
PDE4D	cAMP-specific 3',5'-cyclic phosphodiesterase 4D isoform PDE4D5 [Homo sapien	0	0	0	0	0	0	89191.6278	47113.83919	613540.0154
PDE6D	retinal rod rhodopsin-sensitive cGMP 3',5'-cyclic phosphodiesterase subunit	0	0	0	0	0	0	456451.2779	0	817554.8364
PDGFD	platelet-derived growth factor D isoform 1 precursor [Homo sapiens]	0	0	0	0	0	0	0	0	0
PDHA1	pyruvate dehydrogenase E1 component subunit alpha, somatic form, mitochondi	2618692.858	3298251.179	3723321.678	3248632.275	3587636.382	3236746.982	3491455.323	2665660.333	3704522.524
PDHB	pyruvate dehydrogenase E1 component subunit beta, mitochondrial isoform 1 p	5399799.698	6182988.417	7168531.874	6273190.938	7191558.95	6935562.708	6538331.266	5464298.812	6742185.814
PDHX	pyruvate dehydrogenase protein X component, mitochondrial isoform 1 precurs	382766.6043	264561.4694	178692.5032	266362.5886	524515.2005	516103.1773	480528.1039	332588.2532	298892.6236
PDIA3	protein disulfide-isomerase A3 precursor [Homo sapiens]	13001778.97	13396728.17	16091443.45	14314620.93	17094405.87	14393236.69	13692692.35	12326187.19	13652603.97
PDIA4	protein disulfide-isomerase A4 precursor [Homo sapiens]	2636313.161	2760570.629	2939566.564	2803155.278	3304107.678	3010453.309	2952906.184	2382865.519	3464200.644
PDIA5	protein disulfide-isomerase A5 precursor [Homo sapiens]	258770.6662	216695.8722	345196.0848	113211.4082	387461.7958	271182.1659	310760.0092	364470.1336	233343.1483
PDIA6	protein disulfide-isomerase A6 precursor [Homo sapiens]	11709383.22	13049429.22	14862468.45	13173622.97	14648856.18	13850693.59	14199899.27	11831980.23	14771079
PDK1	[Pyruvate dehydrogenase [lipoamide]] kinase isozyme 1, mitochondrial isofor	0	96610.97202	43983.78825	85665.63798	74127.43579	0	68009.43377	0	73812.13661
PDLIM1	PDZ and LIM domain protein 1 [Homo sapiens]	269598.5808	148422.4689	382516.4703	252027.7094	263212.3035	253021.7659	222183.7708	250176.3551	96760.49788
PDLIM2	PDZ and LIM domain protein 2 isoform 2 [Homo sapiens]	81992.48464	548566.2778	188859.3611	397700.8556	178544.0179	193027.6543	135227.2139	100242.6349	178133.7232
PDLIM5	PDZ and LIM domain protein 5 isoform a [Homo sapiens]	1141994.42	1824860.814	1973811.454	1678807.746	1924560.735	1680803.785	1606844.839	1436442.786	1217142.831
PDLIM7	PDZ and LIM domain protein 7 isoform 1 [Homo sapiens]	1551945.564	676106.2379	1051011.515	548004.4968	924351.8665	684440.2951	466348.4954	511171.065	1007859.493
PDPK1	3-phosphoinositide-dependent protein kinase 1 isoform 1 [Homo sapiens]	88395.9912	0	55539.91244	56850.1976	38381.76152	0	75894.10659	49115.38682	0
PDPR	pyruvate dehydrogenase phosphatase regulatory subunit, mitochondrial precu	129097.3887	30891.69601	99283.71834	58575.02595	96428.77076	62428.94748	66093.4999	58423.69146	123605.7467
PDRG1	p53 and DNA damage-regulated protein 1 [Homo sapiens]	439770.4653	386873.8872	409916.8542	381591.0243	456379.566	500430.2975	377950.0147	371079.8976	391350.3924
PDS5A	sister chromatid cohesion protein PDS5 homolog A isoform 1 [Homo sapiens]	169864.1884	211967.8378	268104.4411	188878.3698	280512.3492	236930.5461	263653.5144	177448.3007	267736.8328
PDS5B	sister chromatid cohesion protein PDS5 homolog B [Homo sapiens]	135614.6872	168772.0306	242505.0702	171844.0521	248047.9341	189067.5376	159259.7619	141324.2544	172860.0604
PDSS1	decaprenyl-diphosphate synthase subunit 1 [Homo sapiens]	36029.765	0	23255.47714	16532.45965	26699.22786	23431.66143	28985.42	0	22690.58857
PDXDC1	pyridoxal-dependent decarboxylase domain-containing protein 1 [Homo sapiens	380601.3982	415164.4591	510326.3127	434023.0467	553415.0192	468560.2417	369699.1895	352600.2313	438944.3076
PDXK	pyridoxal kinase [Homo sapiens]	3873129.17	4392180.645	5258257.956	3314345.31	5214707.468	4349603.352	4381160.858	3727262.376	4046347.693
PDXP	pyridoxal phosphate phosphatase [Homo sapiens]	240428.8677	281714.2577	214573.8947	279485.1082	293798.2975	221926.556	277270.7012	226523.3016	104580.1886
PDZD11	PDZ domain-containing protein 11 [Homo sapiens]	543727.3545	1302082.891	810619.6727	776695.5278	879488.6938	939985.6182	922635.4273	942388.1864	1066221.227
PEA15	astrocytic phosphoprotein PEA-15 [Homo sapiens]	2179075.773	3306692.543	2973329.467	2418782.61	3497742.587	2600618.663	3786806.16	3922136.72	2336713.172
PEAK1	pseudopodium-enriched atypical kinase 1 [Homo sapiens]	4568.02849	0	4949.526977	4450.392272	7398.347044	4539.399662	0	0	0
PEBP1	phosphatidylethanolamine-binding protein 1 preproprotein [Homo sapiens]	36546016.16	44995149.86	48335753.95	44434954.21	48311671.84	45990764.26	45123873.21	40908716.41	47605794.18
PEF1	peflin [Homo sapiens]	0	0	0	0	0	0	2088960.31	1372936.016	835507.9744
PEG10	retrotransposon-derived protein PEG10 isoform 3 [Homo sapiens]	0	0	0	0	0	0	52628.44828	0	50463.74475
PELO	protein pelota homolog [Homo sapiens]	450605.9649	329855.7759	651931.6349	471385.558	544568.3942	345761.3492	350350.8866	358674.8892	686507.4829
PELP1	proline-, glutamic acid- and leucine-rich protein 1 isoform 1 [Homo sapiens	666764.3455	631988.6602	787532.9235	730730.2322	756193.0061	653918.1098	720069.4784	600659.1966	717568.5299
PEPD	xaa-Pro dipeptidase isoform 1 [Homo sapiens]	1598761.993	1748834.253	2081159.54	1737772.361	1971386.15	1178097.023	1647616.499	1418034.94	1544566.793
PERP	p53 apoptosis effector related to PMP-22 [Homo sapiens]	929003.0592	1882004.912	1163884.054	1295806.439	0	0	2144510.419	1082635.811	2216136.074
PES1	pescadillo homolog isoform 1 [Homo sapiens]	1081922.267	841420.6395	1457183.708	891447.3199	1574735.719	1218982.045	1353540.702	1033288.685	1179005.511
PEX1	peroxisome biogenesis factor 1 [Homo sapiens]	4694.080963	0	18287.89788	10251.37045	0	9752.009438	8072.875317	10746.37144	0
PEX13	peroxisome biogenesis factor 13 [Homo sapiens]	0	199303.1621	185021.7056	213908.963	0	159676.501	153565.1829	120886.8004	68798.02407
PEX14	peroxisomal membrane protein PEX14 [Homo sapiens]	604187.0315	761001.5244	705909.0217	627440.6172	552215.5894	384352.169	501634.7793	654635.4648	545906.4218
PEX16	peroxisomal biogenesis factor 16 isoform 2 [Homo sapiens]	474877.8115	647230.624	741360.775	499467.7805	732975.2472	632499.6491	607302.14	358759.0024	589381.1684
PEX19	peroxisomal biogenesis factor 19 isoform a [Homo sapiens]	1376463.335	1603627.066	1757674.876	1472158.046	2027181.003	1453841.885	1089787.923	959859.5655	1594049.642
PEX26	peroxisome assembly protein 26 isoform a [Homo sapiens]	199786.0649	48091.45913	232993.5433	114845.5013	170795.4063	232845.3341	171197.5529	98817.7674	0
PEX3	peroxisomal biogenesis factor 3 [Homo sapiens]	315689.8164	209233.5591	423406.836	341818.2644	390216.2372	397844.9331	424272.187	462962.7224	239163.689
PEX5	peroxisomal biogenesis factor 5 isoform a [Homo sapiens]	77649.02174	152949.1294	115510.5342	292996.4184	252902.1781	292613.3877	308252.55	136814.4634	131235.7825
PEX6	peroxisome biogenesis factor 6 [Homo sapiens]	27082.56291	60576.35232	44256.57007	61056.05147	78152.09722	62875.40686	0	0	0
PFAS	phosphoribosylformylglycinamidine synthase [Homo sapiens]	1263373.23	1302916.773	1755337.824	1342983.234	1725251.997	1409963.974	1303706.337	1145035.641	1327218.105
PFDN1	prefoldin subunit 1 [Homo sapiens]	2473849.481	3267158.681	2998115.788	2725801.804	3163877.359	2663054.543	2813985.057	2911223.688	2886360.537
PFDN2	prefoldin subunit 2 [Homo sapiens]	9738763.745	12499852.19	12070470.46	11096918.08	12708396.59	11915197.57	12426317.88	9890027.63	13899906.32
PFDN4	prefoldin subunit 4 [Homo sapiens]	5980384.726	6380849.312	8235223.918	6503673.052	8939776.212	7280060.486	6644840.932	6314399.87	6878330.94
PFDN5	prefoldin subunit 5 isoform alpha [Homo sapiens]	8888026.44	10624188.36	11375679.14	8817967.523	11292552.67	10483678.73	9765009.89	8186077.68	10116990.62
PFDN6	prefoldin subunit 6 [Homo sapiens]	6372467.063	8182827.206	9078413.799	7475473.468	8789832.32	8911610.269	7419910.406	6413579.607	7703067.904
PFKFB2	6-phosphofructo-2-kinase/fructose-2,6-bisphosphatase 2 isoform a [Homo sap	94705.63333	112227.4501	28160.4	88286.85471	94070.77517	87161.68125	104634.5625	0	108490.3544
PFKL	6-phosphofructokinase, liver type [Homo sapiens]	1056493.735	1097740.459	837120.1875	762312.1751	935319.1345	1381970.773	735975.1495	583855.4128	583714.5594
PFKM	6-phosphofructokinase, muscle type isoform 1 [Homo sapiens]	1366725.346	1365718.909	2061869.552	1325022.86	2157838.183	1837318.467	1772894.982	1098651.678	1634061.74

PFKP	6-phosphofructokinase type C isoform 1 [Homo sapiens]	3582895.171	4367705.575	5328830.317	4602560.338	5666076.003	4912440.094	4811417.199	3851189.446	4885299.732
PFN1	profilin-1 [Homo sapiens]	442467405.8	485986347.1	492251441.1	490133811.4	510196098.2	496761514.4	472879860.4	418859081.9	527862802.1
PFN2	profilin-2 isoform a [Homo sapiens]	3583949.505	3802205.14	4669513.678	4768022.062	4660288.198	2913821.012	4382780.635	2543228.956	4451969.162
PFN2	profilin-2 isoform b [Homo sapiens]	3583949.505	3802205.14	4669513.678	4768022.062	4660288.198	2913821.012	4382780.635	2543228.956	4451969.162
PGAM1	phosphoglycerate mutase 1 [Homo sapiens]	47952206.04	54520327.7	57616112.79	49116551.38	59009948.55	55102046.85	52438553.91	44676147.58	47978690.9
PGAM5	serine/threonine-protein phosphatase PGAM5, mitochondrial isoform 2 [Homo s	420101.7824	840164.414	211393.7901	691134.7853	795216.395	805214.416	849978.0626	551600.6613	497543.2977
PGAM5	serine/threonine-protein phosphatase PGAM5, mitochondrial isoform 1 [Homo s	420101.7824	840164.414	211393.7901	691134.7853	795216.395	805214.416	849978.0626	551600.6613	497543.2977
PGD	6-phosphogluconate dehydrogenase, decarboxylating [Homo sapiens]	6458474.893	7510103.158	8256715.684	7176580.989	8365797.655	7514653.153	6956032.915	6145943.153	7674221.983
PGGT1B	geranylgeranyl transferase type-1 subunit beta [Homo sapiens]	0	251708.773	129938.0567	85076.53638	0	75426.04348	248651.7305	0	110914.523
PGK1	phosphoglycerate kinase 1 [Homo sapiens]	38253060.84	43587884.84	52873960.99	44781798.77	56786872.73	47414288.84	44461904.86	38162684.74	45389633.05
PGLS	6-phosphogluconolactonase [Homo sapiens]	5226364.299	5522260.333	6061135.129	6113540.571	6659649.491	6031902.242	5808679.142	3998650.08	5975770.801
PGM1	phosphoglucomutase-1 isoform 1 [Homo sapiens]	2392522.923	2531170.964	3354292.618	2736784.589	3403410.589	3136362.687	2792171.688	2159911.779	2612694.031
PGM2	phosphoglucomutase-2 [Homo sapiens]	681290.6358	767710.7111	826957.4609	596511.0728	661428.1626	753727.421	657259.7111	598978.1877	664133.0749
PGM3	phosphoacetylglucosamine mutase isoform 1 [Homo sapiens]	736706.4959	929068.8622	1040981.602	0	1080295.13	973957.0117	955714.1939	747403.9403	928825.1781
PGP	phosphoglycolate phosphatase [Homo sapiens]	3311606.367	3452609.104	4211217.463	3580073.395	4070355.685	3830759.734	3492315.066	2852267.163	4308478.755
PGPEP1	pyroglutamyl-peptidase 1 [Homo sapiens]	0	69616.94658	1224991.641	92240.16532	0	353316.6219	0	0	0
PGRMC1	membrane-associated progesterone receptor component 1 [Homo sapiens]	8071651.785	6353400.363	9574250.181	5297757.882	9388937.714	9258161	9863766.575	8987357.46	10524859.15
PGRMC2	membrane-associated progesterone receptor component 2 [Homo sapiens]	391652.8535	824838.1494	2463266.398	2372623.155	2417834.886	3705147.897	2442569.838	2394219.222	3665945.214
PHACTR4	phosphatase and actin regulator 4 isoform 2 [Homo sapiens]	100425.2992	94432.56058	156365.3618	131781.1217	125077.6152	159331.997	141934.6909	81100.80184	132014.1192
PHAX	phosphorylated adapter RNA export protein [Homo sapiens]	637184.1323	533593.5537	654256.6915	633381.7585	834052.9494	521424.9363	451869.0726	382952.7717	592894.9469
PHB	prohibitin [Homo sapiens]	13660049.02	15377958.04	17509792.5	15722789.86	17903805.11	16071177.54	16181097.24	12744712.18	16190631.52
PHB2	prohibitin-2 isoform 1 [Homo sapiens]	11606847.7	11902575.13	16331312.15	13038958.61	17046928.45	13582058.14	13778878.67	10518535.06	13349950.05
PHC2	polyhomeotic-like protein 2 isoform a [Homo sapiens]	0	0	0	0	0	0	50182.09903	48598.52987	0
PHF10	PHD finger protein 10 isoform a [Homo sapiens]	0	0	0	0	0	0	256671.9101	213822.4329	167454.186
PHF14	PHD finger protein 14 [Homo sapiens]	138069.8008	91446.66761	102369.4684	94308.23944	159428.4796	88027.83932	79246.87524	64954.83358	116161.8954
PHF21A	PHD finger protein 21A isoform a [Homo sapiens]	0	0	0	0	0	0	46052.58949	46939.8784	0
PHF23	PHD finger protein 23 [Homo sapiens]	0	0	0	0	0	0	0	0	0
PHF3	PHD finger protein 3 [Homo sapiens]	26142.83017	12284.72295	24954.24311	18168.05282	26434.73321	20301.65612	13072.92098	19370.90342	8626.475912
PHF5A	PHD finger-like domain-containing protein 5A [Homo sapiens]	2843597.312	3842801.49	2964903.769	3986690.117	3126951.976	3767393.559	3853130.852	3028536.671	3256909.966
PHF6	PHD finger protein 6 isoform 1 [Homo sapiens]	208973.4684	209538.3925	339692.0112	223962.5969	518746.5458	264523.2354	308054.0278	333173.6554	436890.1633
PHF8	histone lysine demethylase PHF8 isoform 1 [Homo sapiens]	26549.56663	40528.92658	55832.99402	40125.97249	24880.85825	36696.41919	43224.4689	0	53733.09959
PHGDH	D-3-phosphoglycerate dehydrogenase [Homo sapiens]	8890149.138	9518529.689	11397402.09	9514111.958	12301677.43	8972278.844	9258894.617	8065321.389	10075919.96
PHIP	PH-interacting protein [Homo sapiens]	53172.82999	80493.91242	77175.52906	76036.48795	104241.7924	82962.01021	69727.92264	46997.89469	72513.87622
PHKA1	phosphorylase b kinase regulatory subunit alpha, skeletal muscle isoform is	0	34092.6035	59924.85314	0	27678.73109	39806.61303	18559.07033	26855.86452	25271.04891
PHKA2	phosphorylase b kinase regulatory subunit alpha, liver isoform [Homo sapier	21774.87651	25102.17651	0	331795.9624	0	529588.6651	388572.9157	0	570092.5573
PHKB	phosphorylase b kinase regulatory subunit beta isoform a [Homo sapiens]	29572.40044	19256.71519	31881.25213	27665.48048	34396.73063	26234.02574	23099.653	17271.08581	0
PHKG2	phosphorylase b kinase gamma catalytic chain, liver/testis isoform isoform	0	0	0	0	0	0	0	170755.8713	55646.31138
PHLDA2	pleckstrin homology-like domain family A member 2 [Homo sapiens]	784598.1346	963037.7244	1018855.269	0	991375.2821	0	964351.6891	0	1108699.267
PHLDB1	pleckstrin homology-like domain family B member 1 isoform a [Homo sapiens]	0	0	0	0	0	0	4520.959768	4428.266029	8572.862522
PHLDB2	pleckstrin homology-like domain family B member 2 isoform a [Homo sapiens]	54654.47817	76968.70874	41757.13842	42108.65149	42562.42415	46640.78871	53331.03666	88040.09381	31191.13264
PHLPP1	PH domain leucine-rich repeat-containing protein phosphatase 1 [Homo sapier	0	0	0	0	0	0	15533.30779	13380.79775	4159.916403
PHPT1	14 kDa phosphohistidine phosphatase isoform 3 [Homo sapiens]	5153336.488	4991194.978	9442456.776	5169240.368	9024717.28	5795186.38	6437604.67	4419936.076	4649688.538
PI4K2A	phosphatidylinositol 4-kinase type 2-alpha [Homo sapiens]	0	88860.12787	286405.3163	174992.351	326513.1195	102342.0049	63750.21768	79326.95394	123742.5216
PI4KA	phosphatidylinositol 4-kinase alpha isoform 1 [Homo sapiens]	24264.60066	35185.51026	35945.42074	78513.17633	79970.45367	86528.01443	79739.97947	60360.95888	21786.28905
PI4KB	phosphatidylinositol 4-kinase beta isoform 1 [Homo sapiens]	132991.8095	43593.92652	65143.45131	168217.5276	121362.2203	11382.13534	13894.5597	463596.9362	139845.1322
PIAS1	E3 SUMO-protein ligase PIAS1 [Homo sapiens]	88196.64353	210929.921	169064.5478	136429.4069	155942.4265	164432.2073	37202.76225	116649.549	94809.83765
PIAS4	E3 SUMO-protein ligase PIAS4 [Homo sapiens]	97718.38782	118457.8401	100351.4352	83392.9867	142804.0508	0	37712.43147	47699.63515	0
PICALM	phosphatidylinositol-binding clathrin assembly protein isoform 1 [Homo sap	1594550.131	1162383.756	2286024.535	1565258.794	2145106.734	1616655.496	1116232.144	1687989.322	1655068.371
PIEZO1	piezo-type mechanosensitive ion channel component 1 [Homo sapiens]	15469.17178	31602.41409	0	14729.81665	10849.85888	14948.86845	9083.735951	8093.150702	16802.20817
PIGK	GPI-anchor transamidase precursor [Homo sapiens]	398790.3692	267992.1306	332156.3681	440279.4328	257557.584	324907.5999	491937.7687	341719.4002	237020.1449
PIGO	GPI ethanolamine phosphate transferase 3 isoform 1 [Homo sapiens]	15668.42126	10979.61358	28568.56182	59923.36643	19626.55505	0	11516.74041	8341.933664	4033.731386
PIGS	GPI transamidase component PIG-S [Homo sapiens]	308702.0412	286322.9713	390200.5288	220417.6834	265517.9864	295290.699	302661.5157	224889.4863	325342.8204
PIGT	GPI transamidase component PIG-T isoform 1 precursor [Homo sapiens]	191340.515	200905.6832	202323.4503	375050.6585	224655.6428	394683.5862	790008.7624	105989.3306	188901.0376
PIGU	phosphatidylinositol glycan anchor biosynthesis class U protein precursor	805406.3444	1011166.911	1243064.398	0	1278222.537	0	0	0	0
PIH1D1	PIH1 domain-containing protein 1 [Homo sapiens]	1027041.998	1361799.31	1684027.007	5509090.54	1448141.841	5086798.771	1337693.402	1221690.871	1740232.024
PIK3C2A	phosphatidylinositol 4-phosphate 3-kinase C2 domain-containing subunit alpl	17271.81602	16774.65704	35277.40163	20498.67427	28254.23261	24673.15647	17894.53283	9716.919739	19583.29078
PIK3C3	phosphatidylinositol 3-kinase catalytic subunit type 3 [Homo sapiens]	62264.08104	75259.20055	76281.5261	57392.71093	58875.61522	72648.50962	55624.03049	54415.06799	71087.71635
PIK3R4	phosphoinositide 3-kinase regulatory subunit 4 [Homo sapiens]	53412.32734	101859.4523	88035.55	98969.193	88971.9065	88840.86216	64989.78634	58770.06492	100123.497
PIN1	peptidyl-prolyl cis-trans isomerase NIMA-interacting 1 [Homo sapiens]	1736454.609	3054053.751	1789109.696	2198788.661	1732463.216	2252708.808	2222066.44	1768915.001	3660944.169
PIN4	peptidyl-prolyl cis-trans isomerase NIMA-interacting 4 isoform 1 [Homo sap	1244008.282	1305880.649	1615763.618	780273.9669	1647605.477	761386.3976	722333.171	877226.009	1400701.041
PINX1	PIN2/TERF1-interacting telomerase inhibitor 1 [Homo sapiens]	48016.73893	78345.9259	0	31960.55093	0	81655.23	70351.48859	98669.20596	0
PIP	prolactin-inducible protein precursor [Homo sapiens]	0	0	0	0	0	0	0	0	0
PIP4K2A	phosphatidylinositol 5-phosphate 4-kinase type-2 alpha [Homo sapiens]	85697.99719	112211.1675	99796.08383	115749.4261	57423.60086	82597.63102	108190.6572	62369.45994	79495.14222
PIP4K2B	phosphatidylinositol 5-phosphate 4-kinase type-2 beta [Homo sapiens]	97036.32386	103482.2639	141172.8466	126592.9517	133252.8764	125641.5043	93285.32386	54911.14063	135762.9377
PIP4K2C	phosphatidylinositol 5-phosphate 4-kinase type-2 gamma isoform a [Homo sap	483374.2795	417825.504	663358.3796	485512.9752	663743.8862	463787.9309	427512.8486	166408.8391	387665.9255
PIP5K1A	phosphatidylinositol 4-phosphate 5-kinase type-1 alpha isoform 1 [Homo sap	68568.481	162437.3037	100025.8032	174137.1475	43511.1269	164506.5079	178511.2302	170498.3705	203090.0292
PIR	pirin [Homo sapiens]	2595822.305	2858082.334	3336143.658	3518127.377	3452009.634	3747267.926	2788890.732	1909745.602	3508727.82
PITHD1	PITH domain-containing protein 1 [Homo sapiens]	568428.4517	730588.3042	383476.6487	780412.4812	423292.0769	823566.0769	872693.8616	717344.2859	794079.2594
PITPNA	phosphatidylinositol transfer protein alpha isoform [Homo sapiens]	1050133.97	1126947.961	1503009.096	1199609.56	1422955.536	864695.7597	847650.2306	620773.6637	1312800.434
PITPNB	phosphatidylinositol transfer protein beta isoform [Homo sapiens]	1969452.956	1843972.331	2764305.043	2182325.123	2780106.118	1761162.332	1872396.228	2038966.286	2030209.108
PITPNM1	membrane-associated phosphatidylinositol transfer protein 1 isoform a [Homo	0	0	0	0	0	0	2726.144392	4960.9375	27417.55577
PITRM1	presequence protease, mitochondrial isoform 1 precursor [Homo sapiens]	344229.9712	383718.6055	422011.8712	390490.5406	464758.5199	393092.6809	410450.5641	366587.2644	375448.6898
PKM	pyruvate kinase isozymes M1/M2 isoform a [Homo sapiens]	52737453.67	58804573.62	55825673.42	63266409.46	58970781.39	74339672.25	65117620.25	57385861.5	60562414.74
PKM	pyruvate kinase isozymes M1/M2 isoform c [Homo sapiens]	52737453.67	58804573.62	55825673.42	63266409.46	58970781.39	74339672.25	65117620.25	57385861.5	60562414.74
PKMYT1	membrane-associated tyrosine- and threonine-specific cdc2-inhibitory kinase	0	0	0	0	0	0	52231.60784	0	45779.44751
PKN1	serine/threonine-protein kinase N1 isoform 1 [Homo sapiens]	131390.4762	115666.5411	144123.7093	129445.7569	133727.1439	125537.4279	115306.9141	73436.49266	81565.31569
PKN2	serine/threonine-protein kinase N2 [Homo sapiens]	319808.0301	457287.0955	448849.9019	409729.3554	518112.1675	399697.6224	390420.0768	337549.2224	444569.3282
PKN3	serine/threonine-protein kinase N3 [Homo sapiens]	189092.308	14682.07652	776652.485	23757.26454	132938.4907	19876.50087	0	0	0
PKNOX1	homeobox protein PKNOX1 [Homo sapiens]	240316.3515	0	261326.3515	256631.7525	294485.083	272408.3762	0	0	0
PKP1	plakophilin-1 isoform 1b [Homo sapiens]	0	0	0	0	0	0	61497.647	41695.99186	103275.5228
PKP2	plakophilin-2 isoform 2b [Homo sapiens]	54898.20477	97155.82406	95868.38011	55836.47549	116152.9509	39518.38643	81905.20297	83984.73063	162614.6129
PKP3	plakophilin-3 [Homo sapiens]	239389.4761	1154485.158	1302468.761	269500.854	306524.0202	1257804.088	251723.2845	177602.4045	232213.5356
PKP4	plakophilin-4 isoform a [Homo sapiens]	29645.13287	26999.50278	24947.27882	27063.31483	0	26530.94628	18763.30387	0	20806.46659
PLA2G16	HRAS-like suppressor 3 [Homo sapiens]	0	0	0	0	0	0	0	686415.0615	667504.041
PLA2G4A	cytosolic phospholipase A2 [Homo sapiens]	523416.3486	437761.1039	599783.2008	494506.1367	634483.5263	568744.1915	441778.3525	323628.6625	433726.1533
PLAA	phospholipase A-2-activating protein [Homo sapiens]	536150.3871	603320.5967	601105.9583	394334.5812	682311.4306	403769.5524	636543.3096	353114.1082	672866.0653
PLAUR	urokinase plasminogen activator surface receptor isoform 1 precursor [Homo	0	0	0	0	0	0	50614.95991	34004.08373	0
PLBD2	putative phospholipase B-like 2 isoform 1 precursor [Homo sapiens]	143350.1559	341755.2235	145891.3767	226749.1835	172112.4301	314419.5159	291191.5737	276483.4017	219913.9263
PLCB3	1-phosphatidylinositol 4,5-bisphosphate phosphodiesterase beta-3 isoform 1	173312.9913	149104.621	194064.551	186061.6785	213787.5292	203173.9012	187986.743	137576.6993	169755.4531
PLCD3	1-phosphatidylinositol 4,5-bisphosphate phosphodiesterase delta-3 [Homo sap	54309.0831	103837.5543	0	86347.67586	69427.54224	92042.58793	82628.12645	72526.80372	88674.7041
PLCG1	1-phosphatidylinositol 4,5-bisphosphate phosphodiesterase gamma-1 isoform a	30819.42696	52393.7282	121924.6887	57125.08839	45045.4701	53829.95959	50317.07092	23350.73367	51285.06006
PLCH1	1-phosphatidylinositol 4,5-bisphosphate phosphodiesterase eta-1 isoform a	565708.5338	127354.0255	726248.467	0	678177.6967	0	11851.18938	0	188179.9384
PLCL2	inactive phospholipase C-like protein 2 isoform 1 [Homo sapiens]	0	0	0	0	0	0	21588.73065	22229.63036	0
PLD3	phospholipase D3 [Homo sapiens]	365119.0417	367042.9249	459437.3954	520193.87	446327.6773	392062.4636	318373.5553	242660.5996	159177.9746
PLDN	biogenesis of lysosome-related organelles complex 1 subunit 6 [Homo sapiens	0	0	0	0	0	0	477513.8043	473632.4675	0
PLEC	plectin isoform 1 [Homo sapiens]	184313.0802	29731.10141	54559.8204	212119.3752	42285.85061	230304.2284	40059.42447	40045.26234	255733.3373
PLEC	plectin isoform 1a [Homo sapiens]	184313.0802	29731.10141	54559.8204	212119.3752	42285.85061	230304.2284	40059.42447	40045.26234	255733.3373
PLEK2	pleckstrin-2 [Homo sapiens]	0	0	0	0	0	0	125855.1726	130983.1569	43219.77673
PLEKHA1	pleckstrin homology domain-containing family A member 1 isoform 1 [Homo sap	7886.568989	14766.30738	0	10947.72225	0	16929.5117	0	0	0
PLEKHA2	pleckstrin homology domain-containing family A member 2 [Homo sapiens]	165083.7795	0	133855.9284	133119.2766	127467.1383	172048.2967	104052.5415	152397.6997	170021.3075
PLEKHA6	pleckstrin homology domain-containing family A member 6 [Homo sapiens]	26441.85391	34662.69006	33361.43056	21606.76389	38117.42068	49925.69504	21644.02378	0	33741.30395
PLEKHF1	pleckstrin homology domain-containing family F member 1 [Homo sapiens]	271545.2573	243558.4677	301865.7525	272823.3398	339685.7213	299873.8491	324817.153	188501.6142	245521.1474
PLIN2	perilipin-2 [Homo sapiens]	105485.2115	140557.7115	110752.9481	68240.45428	106123.9654	105809.9458	80351.39233	0	233811.1761
PLIN3	perilipin-3 isoform 1 [Homo sapiens]	5884262.312	10310354.51	8016925.953	6645316.905	8586553.58	7017147.887	6402749.578	5407750.056	6700872.333
PLK1	serine/threonine-protein kinase PLK1 [Homo sapiens]	159201.9856	161788.1724	108752.3747	193891.1343	362142.4194	236941.5313	134266.4387	7964.875059	192055.8033
PLOD1	procollagen-lysine,2-oxoglutarate 5-dioxygenase 1 precursor [Homo sapiens]	371309.5431	317798.2477	420336.8235	534057.2477	388781.3071	342322.7323	300711.9164	393847.4007	
PLOD2	procollagen-lysine,2-oxoglutarate 5-dioxygenase 2 isoform 1 precursor [Homo	1184151.8	1203183.398	1471502.353	1211246.925	1529488.635	1038057.059	1216357.398	963449.734	1248773.3
PLOD3	procollagen-lysine,2-oxoglutarate 5-dioxygenase 3 precursor [Homo sapiens]	454020.5251	609709.262	546634.0819	588300.2066	629333.1624	567428.897	617002.3613	435915.4373	664226.3727
PLRG1	pleiotropic regulator 1 isoform 1 [Homo sapiens]	608205.5485	871145.25	909528.3461	646178.4025	858279.4338	892189.7574	749342.3765	391106.1455	853825.9211
PLS1	plastin-1 [Homo sapiens]	1153517.526	1149700.801	591592.2515	1198091.943	1809786.26	1253053.191	1143789.121	1010885.366	1244962.679
PLS3	plastin-3 isoform 1 [Homo sapiens]	4857585.183	5669359.127	6443888.175	5467296.179	6494578.206	5821108.79	5354009.798	4986183.377	5879360.175
PLXNB2	plexin-B2 precursor [Homo sapiens]	112740.001	165462.9936	154448.3692	570091.6251	116451.2469	152209.8119	155287.3509	97191.81819	151181.6215
PLXND1	plexin-D1 precursor [Homo sapiens]	7815.212891	8185.329492	1944.453077	8374.214819	16905.65703	13534.99932	8545.470642	4415.200391	6812.450294
PM20D2	peptidase M20 domain-containing protein 2 [Homo sapiens]	525839.7578	552286.9396	765323.5804	592349.3846	823806.3638	618385.1031	568368.7791	288812.1187	599006.7101
PML	protein PML isoform 1 [Homo sapiens]	223889.8421	160285.9738	255639.9679	225840.2211	288574.2302	235324.4277	238851.9178	0	186384.5496
PMM2	phosphomannomutase 2 [Homo sapiens]	1570368.521	1757456.279	2794420.655	1624202.912	2637254.631	1723727.918	1489462.054	1475984.237	1633395.085
PMPCA	mitochondrial-processing peptidase subunit alpha precursor [Homo sapiens]	783450.8208	727130.6086	1038019.134	666265.7569	1091776.352	657655.7208	832804.1137	755987.9041	818353.4355
PMPCB	mitochondrial-processing peptidase subunit beta precursor [Homo sapiens]	973589.0061	1055642.419	1055951.611	1172740.58	1296218.312	1226829.007	1239261.34	994082.3508	1303383.397
PMS2	mismatch repair endonuclease PMS2 isoform a [Homo sapiens]	58299.56979	71697.75307	113212.5988	115695.1821	102935.5992	38133.60328	75318.58601	51999.54653	82630.20859
PMVK	phosphomevalonate kinase [Homo sapiens]	1717840.069	1523134.091	1677850.447	1435792.758	2185899.076	1439614.871	1449354.153	1235336.929	1811234.888

PNISR	arginine/serine-rich protein PNISR [Homo sapiens]	112529.8982	108913.8216	177795.4846	112743.4129	0	0	90514.97472	80953.30899	112120.139
PNKD	probable hydrolase PNKD isoform 1 precursor [Homo sapiens]	262472.8938	382977.2149	267148.1644	313699.4658	288881.3767	364811.3288	330155.7671	242124.8219	322874.5479
PNKP	bifunctional polynucleotide phosphatase/kinase [Homo sapiens]	153698.805	185185.2402	119436.252	154379.8422	114915.2818	134170.1179	193828.6769	119163.4208	187795.6169
PNN	pinin [Homo sapiens]	621284.0739	828286.7922	750606.9076	598214.6221	845128.9852	662441.8899	725877.2529	531030.6448	833509.1725
PNO1	RNA-binding protein PNO1 [Homo sapiens]	1217247.935	862823.2359	1463358.262	1363101.081	1371088.637	1168769.905	1299032.26	1077798.164	1381475.836
PNP	purine nucleoside phosphorylase [Homo sapiens]	9689495.257	10829403.41	14549507.54	10427828.65	15091604.53	11872951.98	12092111.71	10434325.58	10765545.21
PNPLA2	patatin-like phospholipase domain-containing protein 2 [Homo sapiens]	84400.44535	0	131512.1101	19276.94261	129444.1069	65569.88799	0	0	0
PNPLA6	neuropathy target esterase isoform a [Homo sapiens]	89852.24834	108099.6102	99047.14379	93251.82866	134972.2125	120657.4512	98092.57265	88870.81798	114985.6755
PNPLA7	patatin-like phospholipase domain-containing protein 7 isoform a [Homo sap	0	0	0	0	0	0	47650.52782	37934.04309	0
PNPO	pyridoxine-5'-phosphate oxidase [Homo sapiens]	2012790.417	2624968.321	2758018.044	2553705.658	2822550.375	2443943.907	2393448.026	2079333.824	2822830.033
PNPT1	polyribonucleotide nucleotidyltransferase 1, mitochondrial precursor [Homo	710971.4465	849411.5215	883040.3869	794162.4538	953629.3279	849356.0788	808319.2612	621762.4901	962795.0157
PODXL	podocalyxin isoform 1 precursor [Homo sapiens]	713548.8903	2331510.781	600011.5665	2084856.652	2353830.784	795655.1679	2398738.679	1969723.538	2392460.945
PODXL2	podocalyxin-like protein 2 precursor [Homo sapiens]	98389.19692	180005.363	212792.7542	168113.6396	195388.2854	149273.1198	199463.9813	168040.525	171232.8354
POFUT1	GDP-fucose protein O-fucosyltransferase 1 isoform 1 precursor [Homo sapiens	0	402087.8785	286842.5141	372024.8051	282350.2465	421263.4542	454493.7606	464266.438	378858.1104
POGK	pogo transposable element with KRAB domain [Homo sapiens]	40440.51804	35569.38312	67997.54896	0	79785.57359	0	0	0	0
POGLUT1	protein O-glucosyltransferase 1 precursor [Homo sapiens]	0	0	0	0	0	0	0	0	0
POGZ	pogo transposable element with ZNF domain isoform 1 [Homo sapiens]	97855.19599	142508.4715	119933.5538	132983.0061	145986.4116	141826.21	96287.25437	73962.94543	108278.3734
POLA1	DNA polymerase alpha catalytic subunit [Homo sapiens]	147107.1127	210455.7439	201577.3366	198620.5375	214818.5321	216839.6953	193151.03	174195.0605	208140.7364
POLA2	DNA polymerase alpha subunit B [Homo sapiens]	846496.9348	792448.2497	989666.1751	837715.4878	1129011.582	811916.7613	780528.0459	742898.2348	934217.226
POLD1	DNA polymerase delta catalytic subunit [Homo sapiens]	193582.0352	220486.0523	238667.5382	218971.8741	241229.6532	234756.4373	221431.8679	174784.7819	288538.8262
POLD2	DNA polymerase delta subunit 2 isoform 2 [Homo sapiens]	0	0	477532.7879	414557.4258	502158.695	423261.339	298040.6609	287125.459	368638.1417
POLD2	DNA polymerase delta subunit 2 isoform 1 [Homo sapiens]	0	0	477532.7879	414557.4258	502158.695	423261.339	298040.6609	287125.459	368638.1417
POLD3	DNA polymerase delta subunit 3 [Homo sapiens]	283373.085	324446.8811	258612.2451	260861.9393	388521.6019	1171144.684	344084.9951	321504.3944	360066.9502
POLDIP2	polymerase delta-interacting protein 2 [Homo sapiens]	1030770.483	1121213.486	988633.6949	1222710.363	1393761.322	1189581.909	1031650.414	812078.4224	1119079.663
POLDIP3	polymerase delta-interacting protein 3 isoform 1 [Homo sapiens]	489223.6604	776015.4362	790124.885	741152.3787	871639.7836	559600.1874	695955.5184	559889.4382	571579.2324
POLE	DNA polymerase epsilon catalytic subunit A [Homo sapiens]	11917.61108	15821.47589	16624.4227	20264.86338	14165.70639	12055.83783	9147.98798	5963.401909	17970.21412
POLE2	DNA polymerase epsilon subunit 2 isoform 1 [Homo sapiens]	62358.7541	0	69718.5082	0	74971.03552	86604.80126	0	82686.38525	84655.31557
POLE3	DNA polymerase epsilon subunit 3 [Homo sapiens]	4379674.021	5579650.056	6199122.379	4868614.524	5841742.323	5348913.085	4960233.345	4533653.186	2995576.818
POLE4	DNA polymerase epsilon subunit 4 [Homo sapiens]	3487788.05	5030439.843	4431996.358	5395083.958	4018935.93	4890258.673	5016528.445	2574375.483	3983846.753
POLG	DNA polymerase subunit gamma-1 [Homo sapiens]	15537.22347	30233.66222	0	0	14076.65764	37001.14196	31540.10069	12242.3981	46943.57029
POLG2	DNA polymerase subunit gamma-2, mitochondrial [Homo sapiens]	133470.8007	121459.2462	122430.8577	142526.4256	69833.69744	0	0	0	0
POLR1A	DNA-directed RNA polymerase I subunit RPA1 [Homo sapiens]	127711.0649	129188.9315	162362.6071	121805.3655	162084.7219	131441.5902	152358.8323	98089.58683	139796.5794
POLR1B	DNA-directed RNA polymerase I subunit RPA2 isoform 1 [Homo sapiens]	161118.7054	148408.278	208430.488	177288.2976	170565.7919	189006.5258	162728.027	125488.7494	178501.073
POLR1C	DNA-directed RNA polymerases I and III subunit RPAC1 [Homo sapiens]	2138026.989	2855239.26	2620417.579	2676857.323	2794916.718	2684486.408	2537319.74	2211883.13	2971744.692
POLR1D	DNA-directed RNA polymerases I and III subunit RPAC2 isoform 1 [Homo sapier	1889512.613	2058249.196	2722096.618	2089222.641	2544046.525	1892401.313	2449096.684	1250572.714	1699594.677
POLR1E	DNA-directed RNA polymerase I subunit RPA49 [Homo sapiens]	319652.2002	247198.9228	225991.2212	355015.349	279842.1929	317097.9733	358984.4856	352281.3357	244015.1372
POLR2A	DNA-directed RNA polymerase II subunit RPB1 [Homo sapiens]	145130.9983	185752.6818	207619.4404	232851.1444	222432.0641	199435.7674	158004.328	183677.2769	171277.1321
POLR2B	DNA-directed RNA polymerase II subunit RPB2 [Homo sapiens]	298179.9334	319168.7504	368988.1359	301552.5918	398279.784	355396.8176	267559.3966	239840.512	352139.7143
POLR2C	DNA-directed RNA polymerase II subunit RPB3 [Homo sapiens]	1219201.897	1533878.772	2002145.939	1249865.079	2078414.521	1672619.254	1267493.279	1408002.529	1372452.651
POLR2D	DNA-directed RNA polymerase II subunit RPB4 [Homo sapiens]	1087529.816	1358371.169	1673896.467	1304915.197	1438199.499	1167877.791	1218489.665	1107640.946	1371796
POLR2E	DNA-directed RNA polymerases I, II, and III subunit RPABC1 [Homo sapiens]	1544095.096	1601757.866	3067330.614	1523720.02	2960170.014	1570329.225	761740.5082	1551126.288	1748577.846
POLR2F	DNA-directed RNA polymerases I, II, and III subunit RPABC2 [Homo sapiens]	0	0	0	0	0	0	2052686.913	1137059.075	0
POLR2G	DNA-directed RNA polymerase II subunit RPB7 [Homo sapiens]	1182870.201	1242811.081	1275524.318	1269756.678	1751442.929	1357558.187	903794.3573	986536.9095	1173932.23
POLR2H	DNA-directed RNA polymerases I, II, and III subunit RPABC3 [Homo sapiens]	2275411.757	2870985.031	5236198.764	2411726.905	5180171.797	2535057.897	2238518.322	1987862.145	1877085.036
POLR2I	DNA-directed RNA polymerase II subunit RPB9 [Homo sapiens]	2049571.114	2312786.643	2782462.418	2392606.657	2659825.073	2745216.755	2363038.723	2028085.017	2205303.139
POLR2J	DNA-directed RNA polymerase II subunit RPB11-a [Homo sapiens]	1967032.091	2098191.957	2656944.859	2601624.22	2504618.041	2218292.165	1977911.995	1884652.859	2176770.583
POLR2K	DNA-directed RNA polymerases I, II, and III subunit RPABC4 [Homo sapiens]	3019842.612	0	2764029.545	3127619.214	2237144.719	3428030.679	2553739.643	2232579.813	2864703.875
POLR2L	DNA-directed RNA polymerases I, II, and III subunit RPABC5 [Homo sapiens]	5409381.413	2947285.815	6288336.225	5932062.913	6527339.204	3052651.831	5127365.079	7871043	5050152.042
POLR2M	protein GRINL1A isoform 1 [Homo sapiens]	0	0	0	0	0	0	0	0	0
POLR3A	DNA-directed RNA polymerase III subunit RPC1 [Homo sapiens]	50527.49571	54916.77739	83852.98483	59728.43111	61811.703	62901.0094	51169.72559	58924.24727	53828.02108
POLR3B	DNA-directed RNA polymerase III subunit RPC2 isoform 1 [Homo sapiens]	100355.3072	106465.1276	142782.7412	33223.35752	301010.072	46357.77953	113280.9493	0	36315.14699
POLR3D	DNA-directed RNA polymerase III subunit RPC4 [Homo sapiens]	77366.14969	103640.3321	84355.83815	121960.0679	92852.04646	135450.9513	158051.8859	91751.2981	63373.83492
POLRMT	DNA-directed RNA polymerase, mitochondrial precursor [Homo sapiens]	108746.9877	103061.1013	142476.2088	114244.6379	144428.9494	122708.998	120267.2836	75033.96964	106920.1301
POM121	nuclear envelope pore membrane protein POM 121 isoform 1 [Homo sapiens]	83424.50202	0	34162.41173	27023.62966	103852.9263	24507.32072	0	0	0
POM121C	nuclear envelope pore membrane protein POM 121C [Homo sapiens]	0	67744.28575	63518.25354	54777.89566	0	30041.95187	74824.51778	69245.4725	8808.183962
POMP	proteasome maturation protein [Homo sapiens]	1984161.998	2249885.008	2731953.042	2235245.49	2564206.954	2209617.869	2010082.299	1857905.257	1881043.008
PON2	serum paraoxonase/arylesterase 2 isoform 1 [Homo sapiens]	2420197.551	2192743.965	2766690.632	2561543.977	2557797.531	2632628.673	2671632.667	2278550.101	2341110.93
POP1	ribonucleases P/MRP protein subunit POP1 [Homo sapiens]	332578.809	411447.4274	440471.774	338260.1921	420310.1046	412931.5914	389323.5448	256258.6392	295835.7981
POP4	ribonuclease P protein subunit p29 [Homo sapiens]	185228.431	216690.0088	391708.0833	291356.9649	406332.0541	219749.1798	202491.7193	189110.0461	257791.9232
POP7	ribonuclease P protein subunit p20 [Homo sapiens]	1346346.78	698967.5932	1117799.316	0	0	2192514.059	1874708.085	1619855.556	1953073.763
POR	NADPH--cytochrome P450 reductase [Homo sapiens]	1842974.649	1744726.94	2446167.418	1991798.265	2399237.851	2102627.614	1578017.636	1618256.613	1891551.644
POU2F1	POU domain, class 2, transcription factor 1 isoform 1 [Homo sapiens]	315226.2152	346829.0865	443265.7434	337080.2883	459139.8301	662486.2921	306812.988	231652.5587	365385.1287
PPA1	inorganic pyrophosphatase [Homo sapiens]	13742437.28	16193577.17	19125513.43	17221401.9	19407900.57	18405382.19	18386966.94	15330909.59	17700273.16
PPA2	inorganic pyrophosphatase 2, mitochondrial isoform 1 precursor [Homo sapier	4437818.412	5951942.332	6114780.511	5731988.534	5699103.135	5859685.74	5785263.745	5059739.262	6013506.772
PPAN-P2RY11	PPAN-P2RY11 protein isoform 1 [Homo sapiens]	286283.3128	249115.1784	340539.731	310964.4326	425783.2663	244512.5357	172356.0999	345399.6546	1679679.599
PPAP2A	lipid phosphate phosphohydrolase 1 isoform 2 [Homo sapiens]	244194.3978	151896.2467	212082.0723	162627.8735	221761	235227.8072	236004.6054	161124.5663	0
PPAP2C	lipid phosphate phosphohydrolase 2 isoform 3 [Homo sapiens]	534524.9279	925651.196	638824.2115	537625.2681	668014.7933	688585.7452	0	0	0
PPAT	amidophosphoribosyltransferase proprotein [Homo sapiens]	583048.3187	788212.5535	656835.1545	566080.6016	673126.608	663286.4513	676323.3679	612466.6781	756059.1551
PPCDC	phosphopantothenoylcysteine decarboxylase [Homo sapiens]	338790.7817	551812.5141	334021.4577	210848.787	298338.1197	436198.3082	0	320584.5282	345589.1175
PPCS	phosphopantothenate--cysteine ligase isoform a [Homo sapiens]	271771.7478	533435.4665	405237.3605	386537.6313	431791.7355	24837.35882	97263.69643	126438.7193	0
PPDPF	pancreatic progenitor cell differentiation and proliferation factor [Homo s	499275.9396	0	846056.5179	0	641235.1854	889968.1964	0	0	0
PPFIA1	liprin-alpha-1 isoform b [Homo sapiens]	116163.668	91216.79734	115669.065	118871.0925	150754.9133	125283.4351	118140.4471	97912.45495	119355.5797
PPFIBP1	liprin-beta-1 isoform 2 [Homo sapiens]	106845.9054	82674.87023	120057.5159	86660.63879	118111.1142	121287.7627	105551.3967	101355.0264	101831.5944
PPHLN1	periphilin-1 isoform 2 [Homo sapiens]	185997.5035	241965.1613	321813.772	270878.9165	323245.125	1022896.829	893630.236	185801.2419	235367.2796
PPHLN1	periphilin-1 isoform 1 [Homo sapiens]	185997.5035	241965.1613	321813.772	270878.9165	323245.125	1022896.829	893630.236	185801.2419	235367.2796
PPIA	peptidyl-prolyl cis-trans isomerase A [Homo sapiens]	317092415.2	348362778.8	401398391.5	339893090.2	403031437.2	371000508.1	367599757.4	343370346.9	434342859.1
PPIB	peptidyl-prolyl cis-trans isomerase B precursor [Homo sapiens]	23901762.11	28842426.5	31612596.6	28669322.25	32446174.25	28564310.75	29585078.23	23892223.64	33227528.3
PPIC	peptidyl-prolyl cis-trans isomerase C precursor [Homo sapiens]	1822082.38	2247406.691	2103098.229	1555416.343	1998579.143	1675424.618	1616966.109	1451787.782	691704.4622
PPID	peptidyl-prolyl cis-trans isomerase D [Homo sapiens]	657054.8294	288896.3002	894042.4382	709672.6876	992529.3547	756246.6212	633753.1441	432280.5701	210855.3245
PPIE	peptidyl-prolyl cis-trans isomerase E isoform 4 [Homo sapiens]	2809015.108	1028834.145	3309754.601	911349.0894	1337523.383	801119.3333	395926.4943	1055150.081	1548140.664
PPIF	peptidyl-prolyl cis-trans isomerase F, mitochondrial precursor [Homo sapier	2503096.361	3686463.064	2792578.695	3065545.84	3074034.238	3872052.821	3309378.241	3199761.815	3785533.926
PPIG	peptidyl-prolyl cis-trans isomerase G [Homo sapiens]	283250.7083	371714.245	357312.7952	374444.3217	317889.8188	216614.7796	376389.1657	254166.5802	215595.7736
PPIH	peptidyl-prolyl cis-trans isomerase H [Homo sapiens]	2794642.955	2737623.397	3160581.633	2574080.217	3389087.378	2726008.972	2626539.048	2484115.248	2908513.074
PPIL1	peptidyl-prolyl cis-trans isomerase-like 1 [Homo sapiens]	6487467.258	5202024.03	8168715.432	5221736.338	8635098.96	5184921.063	5301709.847	3881742.903	5179849.805
PPIL2	peptidyl-prolyl cis-trans isomerase-like 2 isoform a [Homo sapiens]	129451.8315	130468.7893	191662.4147	138384.7689	183018.9959	147796.0693	108454.2846	59286.85417	101925.5946
PPIL3	peptidyl-prolyl cis-trans isomerase-like 3 isoform PPIL3a [Homo sapiens]	968191.1487	0	1895075.262	0	973373.8592	2125133.582	0	1893084.961	1798363.818
PPIL4	peptidyl-prolyl cis-trans isomerase-like 4 [Homo sapiens]	170971.4543	251265.9189	310479.6807	233651.3151	213317.6689	258603.6855	251400.0171	237293.1225	75507.43781
PPIP5K1	inositol hexakisphosphate and diphosphoinositol-pentakisphosphate kinase 1	0	0	0	0	0	0	52430.51343	30385.04053	35846.42857
PPIP5K2	inositol hexakisphosphate and diphosphoinositol-pentakisphosphate kinase 2	29266.40338	36175.38075	37491.23632	16582.47485	40144.32223	25117.17131	0	0	0
PPL	periplakin [Homo sapiens]	19670.05437	8139.131891	28017.06202	31225.93868	30465.8971	33677.83366	17479.66513	0	34593.10143
PPM1A	protein phosphatase 1A isoform 3 [Homo sapiens]	126436.3638	0	150591.956	244012.7032	323298.2978	178336.041	238577.9083	166372.7097	401092.5506
PPM1B	protein phosphatase 1B isoform 1 [Homo sapiens]	195348.5262	42838.7703	307144.2408	178653.6025	295727.6352	181271.5631	118578.844	145917.3973	197587.3866
PPM1F	protein phosphatase 1F [Homo sapiens]	374256.259	289558.6332	375432.0306	304076.4465	413685.6508	492111.5553	390308.0853	195167.8461	330068.7178
PPM1G	protein phosphatase 1G [Homo sapiens]	2398918.918	3128682.789	3392879.735	2816033.933	3321955.446	3208062.822	2483844.021	2710330.802	3455409.531
PPM1H	protein phosphatase 1H [Homo sapiens]	58526.41851	72315.25657	98262.78729	63316.2721	98206.58011	97829.90193	74684.31354	0	92094.66575
PPME1	protein phosphatase methylesterase 1 isoform b [Homo sapiens]	1667068.231	2077675.03	1964948.162	2106465.957	2090522.296	1851064.353	1673023.907	1675041.804	2399613.472
PPOX	protoporphyrinogen oxidase [Homo sapiens]	268743.0178	340481.4317	348583.0614	115651.5949	390172.1905	306154.1813	251218.8953	301723.8436	351953.7622
PPP1CA	serine/threonine-protein phosphatase PP1-alpha catalytic subunit isoform 3	1786985.634	3107212.243	2617864.225	2300769.277	2751815.706	2376364.531	2139463.912	1888181.924	2277951.965
PPP1CB	serine/threonine-protein phosphatase PP1-beta catalytic subunit isoform 1	2769763.111	2448534.07	3106937.113	2339171.273	3257679.497	3001111.399	2749035.386	2389718.703	2850648.355
PPP1CC	serine/threonine-protein phosphatase PP1-gamma catalytic subunit isoform 2	2218582.295	2471754.851	2436711.95	2307715.042	1700617.83	2575779.676	1874041.414	2020619.543	1938816.108
PPP1R10	serine/threonine-protein phosphatase 1 regulatory subunit 10 [Homo sapiens]	205471.2166	195542.2747	210867.6478	262970.0863	195348.4967	170907.3265	206921.3391	145242.4031	273493.7878
PPP1R11	protein phosphatase 1 regulatory subunit 11 [Homo sapiens]	1771658.291	1520879.978	2339984.161	709630.256	3045913.426	617552.8164	475066.1662	533529.0924	1662494.159
PPP1R12A	protein phosphatase 1 regulatory subunit 12A isoform a [Homo sapiens]	204631.7457	225110.2793	218975.6348	230931.6454	219851.4842	253284.6896	256558.7592	184298.4172	275835.9145
PPP1R13L	relA-associated inhibitor [Homo sapiens]	129962.9014	187328.3158	256287.7527	158767.4381	272943.8733	175000.5325	152189.5087	117663.0844	175184.7584
PPP1R14B	protein phosphatase 1 regulatory subunit 14B [Homo sapiens]	5389064.696	5409703.644	6478149.2	5234319.265	6365215.504	5804082.974	5036384.528	5139265.389	5768386.802
PPP1R18	phostensin [Homo sapiens]	81589.74068	155391.3114	191600.5027	108384.8457	237767.83	103873.855	95775.78126	0	107170.0276
PPP1R2	protein phosphatase inhibitor 2 [Homo sapiens]	2236302.663	2492956.151	2956995.139	3294273.376	3623218.701	2622316.73	2241116.477	1747974.478	1954999.605
PPP1R3D	protein phosphatase 1 regulatory subunit 3D [Homo sapiens]	51101.64496	26464.00315	0	93553.14126	82976.14286	7119191.143	0	0	0
PPP1R7	protein phosphatase 1 regulatory subunit 7 [Homo sapiens]	642578.0331	620274.2123	675098.8013	661378.1915	722634.9861	842289.5483	721110.7954	506385.0493	579231.7781
PPP1R8	nuclear inhibitor of protein phosphatase 1 isoform alpha [Homo sapiens]	873770.2488	1174735.764	1207924.21	509905.2724	1251745.99	998783.526	869220.2537	527865.685	1225039.97
PPP1R9B	neurabin-2 [Homo sapiens]	231603.4223	33384.634	31357.46563	18329.84531	36577.03281	0	0	0	0
PPP2CA	serine/threonine-protein phosphatase 2A catalytic subunit alpha isoform [Ho	3777193.682	7282466.044	8533975.443	7699018.386	8166598.785	7844973.803	7350509.85	6424390.988	8825202.203
PPP2R1A	serine/threonine-protein phosphatase 2A 65 kDa regulatory subunit A alpha	3528511.712	4340254.002	4865823.272	4375713.574	5313575.816	4190550.044	4471792.373	3230039.07	6440715.617
PPP2R1B	serine/threonine-protein phosphatase 2A 65 kDa regulatory subunit A beta is	600170.4056	653745.0867	715801.4948	605003.2124	849948.191	0	695804.2695	505140.2858	626368.9584
PPP2R2A	serine/threonine-protein phosphatase 2A 55 kDa regulatory subunit B alpha	1340035.186	1355757.415	1630000.956	1414454.25	1979025.644	1405447.052	1546737.675	1344005.351	1509052.247
PPP2R2D	serine/threonine-protein phosphatase 2A 55 kDa regulatory subunit B delta	61389.67016	0	89017.09762	109266.4042	0	97498.65135	0	0	0
PPP2R4	serine/threonine-protein phosphatase 2A activator isoform a [Homo sapiens]	2281164.981	1901296.932	1523210.723	1938071.698	2041050.436	1632731.332	2150108.566	1933097.797	2036009.94
PPP2R5A	serine/threonine-protein phosphatase 2A 56 kDa regulatory subunit alpha isc	115044.0845	103903.6316	123549.1715	135531.7587	118488.7983	129073.0988	126851.7544	96844.23117	101044.5492
PPP2R5C	serine/threonine-protein phosphatase 2A 56 kDa regulatory subunit gamma isc	189518.5212	246579.1991	263313.2869	231322.9898	170182.544	294598.1775	269606.6802	156190.4022	240411.8191
PPP2R5D	serine/threonine-protein phosphatase 2A 56 kDa regulatory subunit delta isc	384087.7299	549114.0647	757395.2823	566792.2084	749539.3233	534877.2108	383716.1247	470990.504	573458.6181

PPP2R5E	serine/threonine-protein phosphatase 2A 56 kDa regulatory subunit epsilon i	230655.3557	283985.8949	279808.8907	263922.6023	198734.6454	230868.0079	370846.0102	130149.6001	248329.9947
PPP3CA	serine/threonine-protein phosphatase 2B catalytic subunit alpha isoform iso	881664.7989	1226130.901	1234728.952	1280813.864	1007455.582	1160819.164	1095840.828	802795.993	1317211.764
PPP3R1	calcineurin subunit B type 1 [Homo sapiens]	935077.6573	952243.4926	1412044.382	429919.5868	1218192.312	1325152.605	1376475.345	1214114.165	468265.9819
PPP4C	serine/threonine-protein phosphatase 4 catalytic subunit [Homo sapiens]	1583472.044	7577659.854	2403440.777	1958768.762	2346752.842	1910816.088	1802023.018	1393823.442	7781941.286
PPP4R1	serine/threonine-protein phosphatase 4 regulatory subunit 1 isoform a [Homo	269501.3279	0	339572.5221	294872.0147	169569.5112	318683.5988	267540.2807	192815.1696	270193.4287
PPP4R2	serine/threonine-protein phosphatase 4 regulatory subunit 2 [Homo sapiens]	668213.8687	744312.722	765392.5753	745505.182	883533.4167	762524.9273	885050.9607	695540.3587	775378.9747
PPP5C	serine/threonine-protein phosphatase 5 isoform 1 [Homo sapiens]	1399121.066	1618567.824	1646844.534	1458813.764	1818557.894	1515384.526	1431209.695	1082111.979	1554236.258
PPP6C	serine/threonine-protein phosphatase 6 catalytic subunit isoform a [Homo sa	1571863.13	1693487.448	1691111.344	1552191.34	1738616.683	1574006.671	1006125.761	1352496.171	1597411.98
PPP6R1	serine/threonine-protein phosphatase 6 regulatory subunit 1 [Homo sapiens]	250910.2235	231904.471	334519.9872	454492.0482	435721.7821	347371.8073	293775.0578	445906.5829	1344610.096
PPP6R2	serine/threonine-protein phosphatase 6 regulatory subunit 2 isoform 1 [Homo	63241.00585	0	92451.4791	108660.9191	76052.4793	214465.4749	87545.13391	0	133674.7734
PPP6R3	serine/threonine-protein phosphatase 6 regulatory subunit 3 isoform 4 [Homo	693393.0376	756945.8505	834717.7728	764903.5799	882589.2215	838445.7032	774052.0505	599835.4254	544917.2434
PPT1	palmitoyl-protein thioesterase 1 isoform 1 precursor [Homo sapiens]	1680653.382	1981157.1	2880964.566	2368799.934	2916273.738	2485110.245	1937609.959	1747717.389	2080209.933
PPT2	lysosomal thioesterase PPT2 isoform b precursor [Homo sapiens]	780659.1962	611055.22	931559.4289	0	874652.6334	607470.7215	718835.3418	470445.3639	0
PPWD1	peptidylprolyl isomerase domain and WD repeat-containing protein 1 [Homo sa	201403.5336	234419.3437	240269.3471	228045.0964	241716.0836	234171.1087	262070.1541	210745.0969	286741.0082
PQBP1	polyglutamine-binding protein 1 isoform 1 [Homo sapiens]	1477558.883	1723190.889	1954942.821	1773211.616	2100081.45	1939521.053	1598011.845	1639449.515	1821074.453
PRAF2	PRA1 family protein 2 [Homo sapiens]	1144766.337	766241.5163	1230249.778	1121834.884	1205306.519	1247000.882	820577.1343	888376.3635	852701.3606
PRC1	protein regulator of cytokinesis 1 isoform 1 [Homo sapiens]	762520.4216	774388.5198	1081319.61	838649.1597	90161.22302	239017.1947	0	668921.7198	678421.7806
PRCC	proline-rich protein PRCC [Homo sapiens]	481523.7472	624761.3975	689034.8138	626116.3607	998339.648	711408.0678	594029.2111	539782.2566	629999.0541
PRCP	lysosomal Pro-X carboxypeptidase isoform 2 preproprotein [Homo sapiens]	451914.0591	399259.5801	648300.429	337286.8481	463988.4728	202900.3278	204717.9834	345815.6202	382262.3617
PRDX1	peroxiredoxin-1 [Homo sapiens]	151931769.3	148944389.6	192599438.7	157748091.7	204030991.7	158528091.1	150752068.3	131036916.8	168922503.6
PRDX2	peroxiredoxin-2 [Homo sapiens]	25645182.62	26832915.74	32426809.61	27591894.61	32782890.64	27340503	25743487.28	23454241.91	27219103.64
PRDX3	thioredoxin-dependent peroxide reductase, mitochondrial isoform a precursor	15105664.8	18212142.07	20500633.72	18740667.56	19589484.65	19947143.29	19922410.95	13015937.51	17962823.8
PRDX4	peroxiredoxin-4 precursor [Homo sapiens]	8845839.492	11528875.77	11932244.6	11758358.21	12379010.64	11656907.91	12634106.68	10109878.75	13311157.72
PRDX5	peroxiredoxin-5, mitochondrial isoform a precursor [Homo sapiens]	22850172.74	28774086.68	29330147.07	27131713.51	27417214.69	29072814.85	27851479.72	24469593.57	32087158.38
PRDX6	peroxiredoxin-6 [Homo sapiens]	42507093.04	50234305.55	56328648.85	49998942.12	60134649.4	53547141.75	48669280.5	43606981.32	49647870.85
PREB	prolactin regulatory element-binding protein [Homo sapiens]	52589.18936	471987.1309	251825.2901	67064.91296	424980.793	341886.3517	249485.7299	566655.363	456732.3235
PREP	prolyl endopeptidase [Homo sapiens]	505172.8835	638189.231	717640.8372	573618.8272	558512.5843	680417.0824	684959.5579	476222.1172	507401.8521
PREPL	prolyl endopeptidase-like isoform 1 [Homo sapiens]	337931.4306	538214.3102	441328.7969	386500.5783	329644.592	386092.7681	314240.3359	282811.6695	343222.1332
PRIM1	DNA primase small subunit [Homo sapiens]	243005.0369	317406.957	314327.4648	218480.342	366961.0715	235260.861	287622.4601	199723.2373	238242.7946
PRIM2	DNA primase large subunit [Homo sapiens]	574615.7938	658426.2262	770628.4836	639798.1738	755095.9524	688581.8258	657630.9391	539613.4867	575745.0142
PRKAA1	5'-AMP-activated protein kinase catalytic subunit alpha-1 isoform 2 [Homo s	308257.9558	410593.3719	347035.4639	385842.3855	356544.8465	382253.2664	312029.9565	277534.8708	389454.8933
PRKAB1	5'-AMP-activated protein kinase subunit beta-1 [Homo sapiens]	382958.3508	386428.6556	499121.1212	490156.4937	483137.199	443479.906	477605.7328	154089.0261	473664.9872
PRKACA	cAMP-dependent protein kinase catalytic subunit alpha isoform 1 [Homo sapie	750736.846	545458.9104	1657965.064	1296941.603	1056293.777	666388.4149	604330.97	503702.3332	1049327.531
PRKACB	cAMP-dependent protein kinase catalytic subunit beta isoform 1 [Homo sapien	106439.9854	134716.0782	326448.2039	302381.6313	286601.9409	374079.4844	339233.7542	93122.40084	199700.6497
PRKAG1	5'-AMP-activated protein kinase subunit gamma-1 isoform 3 [Homo sapiens]	564284.93	0	654794.2118	494448.9533	877981.3328	616038.5153	491083.5063	480683.7468	408000.9182
PRKAR1A	cAMP-dependent protein kinase type I-alpha regulatory subunit isoform a [Ho	942957.8994	988732.9441	1206878.205	1313366.913	1409398.738	1391538.488	1179884.859	652364.0323	1243940.208
PRKAR1B	cAMP-dependent protein kinase type I-beta regulatory subunit [Homo sapiens]	387266.0855	192812.3708	279237.758	106856.3089	395882.3323	2167246.568	1887182.75	118815.2868	237108.302
PRKAR2A	cAMP-dependent protein kinase type II-alpha regulatory subunit [Homo sapien	2032268.054	2381080.108	2442690.602	2094498.474	2339581.814	2154747.108	1815115.161	1708323.371	2461973.36
PRKCB	protein kinase C beta type isoform 2 [Homo sapiens]	0	0	0	0	0	0	0	72912.86745	70297.83534
PRKCD	protein kinase C delta type [Homo sapiens]	67799.63498	63732.08907	96141.57352	69357.01412	110058.8488	47115.63133	41261.08252	0	129109.5871
PRKCDBP	protein kinase C delta-binding protein [Homo sapiens]	232920.3481	343226.0559	137680.9898	279631.2628	146638.5638	232115.3608	240689.6448	198284.0559	177314.9479
PRKCI	protein kinase C iota type [Homo sapiens]	306732.8148	268983.265	251105.4528	265522.2289	322768.5598	275743.5478	228224.1298	280008.1874	218428.3419
PRKCSH	glucosidase 2 subunit beta isoform 1 precursor [Homo sapiens]	9139593.548	10606307.33	11909042.43	8865559.602	11873996.7	10423359.16	11352280.86	9147976.783	12189297.73
PRKDC	DNA-dependent protein kinase catalytic subunit isoform 1 [Homo sapiens]	363144.9932	419924.9337	492160.9678	406069.8736	523607.3585	438580.7095	413622.447	350738.5107	448967.8489
PRKRA	interferon-inducible double stranded RNA-dependent protein kinase activator	1165638.853	1106870.598	1486052.289	1212369.936	1531207.991	1179472.351	1033155.675	1218671.345	1017851.641
PRMT1	protein arginine N-methyltransferase 1 isoform 3 [Homo sapiens]	6744710.384	7433327.174	8737360.304	8018433.203	9132937.5	8141895.993	8199699.268	6519661.91	8316768.043
PRMT10	putative protein arginine N-methyltransferase 10 [Homo sapiens]	0	0	0	0	0	0	0	0	0
PRMT3	protein arginine N-methyltransferase 3 isoform 1 [Homo sapiens]	694777.3059	755686.5176	840742.5069	677683.2553	809077.0096	678851.9723	600253.766	585571.1713	763546.3814
PRMT5	protein arginine N-methyltransferase 5 isoform a [Homo sapiens]	2132569.295	2233395.701	2798010.605	2272975.402	2630112.771	2337266.639	2280479.705	1899322.6	2352880.113
PRMT7	protein arginine N-methyltransferase 7 isoform 1 [Homo sapiens]	302442.5226	228898.4159	419619.323	491300.1681	403720.561	314208.2171	222484.0397	161064.121	225651.9688
PRNP	major prion protein preproprotein [Homo sapiens]	0	0	0	0	0	0	0	0	0
PROCR	endothelial protein C receptor precursor [Homo sapiens]	770288.7357	1166720.426	968796.1292	1085191.419	674338.8241	1191428.94	1328541.253	1470273.678	692385.2213
PROS1	vitamin K-dependent protein S preproprotein [Homo sapiens]	33437.87654	0	0	28730.88284	49019.53704	27345.39866	0	0	0
PROSC	proline synthase co-transcribed bacterial homolog protein [Homo sapiens]	293304.4157	326151.3939	701225.0849	464504.5861	417312.4292	283102.1557	308367.8391	176396.4057	0
PRPF19	pre-mRNA-processing factor 19 [Homo sapiens]	6656002.948	7361075.477	8807427.974	7943277.516	8048492.34	8468142.516	8534205.725	6721773.052	8672490.745
PRPF3	U4/U6 small nuclear ribonucleoprotein Prp3 [Homo sapiens]	3672082.409	475224.5418	868888.431	535080.3222	882878.1734	3806437.003	3806075.561	429233.5415	716624.2509
PRPF31	U4/U6 small nuclear ribonucleoprotein Prp31 [Homo sapiens]	740519.4646	1010982.748	1046278.669	882938.0933	926200.4574	926508.3877	776458.7323	716257.2359	1067555.855
PRPF38A	pre-mRNA-splicing factor 38A [Homo sapiens]	272762.6535	461002.0793	385511.6727	351954.421	458359.429	504725.1242	450297.5735	350325.3967	679532.5952
PRPF38B	pre-mRNA-splicing factor 38B [Homo sapiens]	147561.2387	184924.2139	190822.657	175309.3679	199557.3439	189362.6036	172197.7731	87985.29256	0
PRPF39	pre-mRNA-processing factor 39 [Homo sapiens]	33198.18065	0	26746.03884	14957.02639	43542.19633	40994.62426	40897.8506	19783.30615	46375.72578
PRPF4	U4/U6 small nuclear ribonucleoprotein Prp4 isoform 1 [Homo sapiens]	893238.3729	1046287.583	1267317.968	1118573.677	1321607.546	1214659.186	1039494.508	895903.9043	1047399.461
PRPF40A	pre-mRNA-processing factor 40 homolog A [Homo sapiens]	654153.4622	714477.2882	943906.6408	732802.9944	960539.6051	737129.8807	659311.2764	628454.7212	733564.0617
PRPF4B	serine/threonine-protein kinase PRP4 homolog [Homo sapiens]	171224.949	200172.4453	226318.7382	181109.3699	206063.5525	183790.1254	180517.5917	151012.9139	194678.8212
PRPF6	pre-mRNA-processing factor 6 [Homo sapiens]	377688.7748	549843.98	560039.5939	489935.837	468874.1516	531327.1345	476774.3907	496953.2393	584898.7664
PRPF8	pre-mRNA-processing-splicing factor 8 [Homo sapiens]	647361.3904	738609.4065	920722.4425	1054435.909	1351230.996	1181942.897	758239.1218	616064.8435	667908.3432
PRPS1	ribose-phosphate pyrophosphokinase 1 isoform 1 [Homo sapiens]	1427118.145	2631620.224	2568053.101	2296528.403	1803777.568	1845491.582	1864825.829	1342392.509	2023882.437
PRPS2	ribose-phosphate pyrophosphokinase 2 isoform 1 [Homo sapiens]	3729497.414	2894166.976	4986936.396	4040706.328	4229425.938	4138396.655	3999456.05	3354415.306	4080936.63
PRPSAP1	phosphoribosyl pyrophosphate synthase-associated protein 1 [Homo sapiens]	617700.0652	847146.9474	1013796.57	697700.7266	754575.0864	967137.9981	906255.4747	806385.9669	926284.6805
PRPSAP2	phosphoribosyl pyrophosphate synthase-associated protein 2 isoform 1 [Homo	1044219.02	979041.3604	1404214.462	755997.7128	1371215.198	1319709.348	799450.0745	931339.4262	993472.6054
PRR12	proline-rich protein 12 [Homo sapiens]	11721.8812	0	21243.14876	12538.22441	0	11140.72562	7818.192046	5683.895868	0
PRR3	proline-rich protein 3 isoform a [Homo sapiens]	222490.7258	488240.2808	224828.2427	249614.6929	260896.8204	180901.3173	227734.8634	19259.59075	235914.473
PRRC1	protein PRRC1 [Homo sapiens]	1580805.387	1305151.119	1991475.748	2086598.259	2045884.247	2180013.621	2144144.903	1562630.331	2699268.681
PRRC2A	protein PRRC2A [Homo sapiens]	63258.98936	75865.09184	90247.80639	95181.80295	98925.77327	98349.57083	90616.97919	70853.28858	73601.26736
PRRC2B	protein PRRC2B [Homo sapiens]	14577.74558	15333.96796	17501.66292	12895.53744	56313.03499	10676.5191	0	8236.441298	9243.633287
PRRC2C	protein PRRC2C [Homo sapiens]	136627.391	140889.2863	147166.0435	143322.0854	164632.3922	143563.0724	114954.6822	85729.26112	113334.2271
PRSS2	PREDICTED: putative trypsin-6 isoform 2 [Homo sapiens]	0	2596671.308	0	2314550.538	1735188.89	1746628.712	2607083.699	3181171.342	2063826.171
PRSS21	testisin isoform 1 preproprotein [Homo sapiens]	948068.9292	547873.1274	965292.532	324257.1472	1253916.503	762708.6808	948575.1016	972142.594	1756267.943
PRSS3	trypsin-3 isoform 1 preproprotein [Homo sapiens]	20074859.73	0	22821204.48	14819776.54	22956418.42	15321911.85	16590718.11	14499316.34	20402732.4
PRSS56	serine protease 56 precursor [Homo sapiens]	550599.0311	823618.0417	799644.449	764904.5087	819495.7398	640960.0665	258040.9809	298768.2516	156762.3532
PRTFDC1	phosphoribosyltransferase domain-containing protein 1 [Homo sapiens]	170593.7249	2521482.058	251705.2479	188146.0618	252367.5829	148336.8783	72168.62921	175508.9494	162708.5433
PRUNE	protein prune homolog [Homo sapiens]	157120.4418	223340.296	226580.623	225009.6964	328416.9336	245289.0129	205103.6636	103441.4469	201707.8721
PSAP	proactivator polypeptide isoform b preproprotein [Homo sapiens]	6114547.141	0	6947347.75	6896374.168	6851339.734	7310987.511	6458671.25	5295014.898	5765855.989
PSAT1	phosphoserine aminotransferase isoform 1 [Homo sapiens]	6852335.995	7754015.925	8695781.687	8421494.843	7483383.963	8262906.276	7690142.03	6428003.144	7820952.09
PSCA	prostate stem cell antigen preproprotein [Homo sapiens]	0	0	0	0	0	0	832446.9167	3985740.354	1292876.625
PSEN1	presenilin-1 isoform I-467 [Homo sapiens]	0	0	0	0	0	0	0	110633.877	254752.6012
PSEN2	presenilin-2 isoform 1 [Homo sapiens]	176918.2257	0	196124.6466	225229.6905	74530.96094	219792.7313	0	0	0
PSIP1	PC4 and SFRS1-interacting protein isoform 2 [Homo sapiens]	788418.554	1247002.086	1417014.134	783511.3036	1167929.69	884880.2529	702962.1374	554169.8165	1593189.547
PSMA1	proteasome subunit alpha type-1 isoform 1 [Homo sapiens]	10854884.1	11777871.08	12761767.31	14930874.09	14453015.96	11542889.43	11527661.11	8357029.196	12353047.31
PSMA2	proteasome subunit alpha type-2 [Homo sapiens]	12808769.09	14717461.08	22486113.02	16905053.74	23071241.62	15953469.01	19510854.66	13909549.35	16102029.87
PSMA3	proteasome subunit alpha type-3 isoform 1 [Homo sapiens]	7608486.624	11284842.96	10274438.4	10689672.03	10300177.64	10690109.92	11615348.32	10211959.84	9897490.589
PSMA4	proteasome subunit alpha type-4 isoform 1 [Homo sapiens]	10342168.77	10492228.87	12476887.23	10580685.66	11754314.68	11273217.77	10342770.46	8745418.1	11364222.09
PSMA5	proteasome subunit alpha type-5 isoform 1 [Homo sapiens]	36510257.51	35877615.13	46348338.93	38936571.24	51554976	39830254.36	37295422.58	31693279.14	38694366.18
PSMA6	proteasome subunit alpha type-6 [Homo sapiens]	12398570.77	13428374.21	15403601.88	14386040.34	16128777.22	13589828.29	13220296.37	11039027.99	16191801.52
PSMA7	proteasome subunit alpha type-7 [Homo sapiens]	15552049.39	18093616.76	19683030.83	17993350.83	21843115.18	18817680.04	18015490.72	15321155.18	19550137.68
PSMB1	proteasome subunit beta type-1 [Homo sapiens]	11196521.47	13215950.88	14206680.26	15115564.6	17692137.47	16319360.57	15613117.79	11230681.6	17390475.94
PSMB2	proteasome subunit beta type-2 isoform 1 [Homo sapiens]	16619290.94	15768146.96	18909068.74	17082859.4	19112766.19	16906660.31	16299217.3	9867394.28	15973780.37
PSMB3	proteasome subunit beta type-3 [Homo sapiens]	16530923.01	19253248.33	21800649.63	20956829.01	21909087.15	21380061.94	20782882.42	16299326.53	20401478.13
PSMB4	proteasome subunit beta type-4 [Homo sapiens]	12399596.93	15463788.6	17525029.13	15986991.56	16729121.73	16740632.21	15692031.67	12830228.7	16454789.73
PSMB5	proteasome subunit beta type-5 isoform 1 [Homo sapiens]	2665608.612	2726851.753	6420622.09	4722968.111	3729779.565	2852875.421	4667983.332	3538368.198	2591760.624
PSMB5	proteasome subunit beta type-5 isoform 2 [Homo sapiens]	2665608.612	2726851.753	6420622.09	4722968.111	3729779.565	2852875.421	4667983.332	3538368.198	2591760.624
PSMB6	proteasome subunit beta type-6 isoform 1 proprotein [Homo sapiens]	21112123.63	23149794.18	28292365.78	22043722.63	28245385.34	24254649.97	17351520.03	19501014.71	25037238.74
PSMB7	proteasome subunit beta type-7 proprotein [Homo sapiens]	6824345.779	6948953.214	10421580.69	8366052.788	9484943.767	8157315.314	8392922.016	5222431.544	9508065.186
PSMB8	proteasome subunit beta type-8 isoform E2 proprotein [Homo sapiens]	442020.1294	449148.2242	570612.9774	378673.6954	290677.0607	669725.9997	691904.3908	496411.6631	494312.2035
PSMC1	26S protease regulatory subunit 4 [Homo sapiens]	3254003.24	3273294.532	4151740.447	3581000.065	4038333.982	3181989.309	3524409.316	3471441.474	3044900.696
PSMC2	26S protease regulatory subunit 7 isoform 1 [Homo sapiens]	2583879.662	3209124.016	3080845.546	3126564.071	2784514.14	3279435.494	2936471.949	2573717.863	3192754.32
PSMC3	26S protease regulatory subunit 6A [Homo sapiens]	3879140.946	4758919.363	5197520.513	4817411.431	5408029.618	4935019.228	4626424.102	3994357.889	4814096.305
PSMC4	26S protease regulatory subunit 6B isoform 1 [Homo sapiens]	3212624.556	3131907.954	3966719.018	3280670.059	4206995.683	3759101.144	3175024.645	2714553.193	3052788.51
PSMC5	26S protease regulatory subunit 8 isoform 1 [Homo sapiens]	2725044.311	3215270.102	3623627.117	2960375.764	3689337.338	3464217.417	3203533.224	2765942.982	3224172.034
PSMC6	26S protease regulatory subunit 10B [Homo sapiens]	4332829.649	4300596.102	5751714.724	4835024.23	5560286.148	5003950.196	4659042.114	4198513.702	4451580.894
PSMD1	26S proteasome non-ATPase regulatory subunit 1 isoform 1 [Homo sapiens]	1378677.65	1471288.14	1806537.739	2611362.386	1785438.979	1473326.736	1508315.623	1223170.586	2705123.185
PSMD10	26S proteasome non-ATPase regulatory subunit 10 isoform 1 [Homo sapiens]	2902236.895	1501574.869	2301220.436	1641955.626	2230131.201	2360301.232	1697639.947	1834918.348	1888557.425
PSMD11	26S proteasome non-ATPase regulatory subunit 11 [Homo sapiens]	4034564.189	4388135.219	5305236.86	4825017.777	5503811.495	5141919.357	4615068.247	4154639.385	4402400.836
PSMD12	26S proteasome non-ATPase regulatory subunit 12 isoform 1 [Homo sapiens]	2412143.824	2602497.697	3211288.717	2589010.928	3018252.247	2616703.199	2485819.946	2045442.095	2551572.834
PSMD13	26S proteasome non-ATPase regulatory subunit 13 isoform 1 [Homo sapiens]	3134771.876	3831631.693	5062550.632	4041098.785	5493133.591	3779888.104	3790605.905	2988554.917	3992112.027
PSMD14	26S proteasome non-ATPase regulatory subunit 14 [Homo sapiens]	4567942.77	5170665.036	3858066.569	3336827.933	5774601.311	3494316.369	4978294.621	4031604.983	5050397.848
PSMD2	26S proteasome non-ATPase regulatory subunit 2 [Homo sapiens]	2389037.677	2428351.91	2868125.909	2365510.157	2905144.843	2585377.484	2427064.251	1910229.91	2566405.809
PSMD3	26S proteasome non-ATPase regulatory subunit 3 [Homo sapiens]	2239116.399	2644372.628	2878756.966	2384971.731	3094262.398	2654153.098	2309095.943	2204259.315	2802687.352
PSMD4	26S proteasome non-ATPase regulatory subunit 4 [Homo sapiens]	3040056.473	3878248.27	6342519.555	4733673.112	5520360.412	4262633.423	3324766.569	3546395.442	4235631.499
PSMD5	26S proteasome non-ATPase regulatory subunit 5 isoform 1 [Homo sapiens]	1879763.898	1777705.665	2249210.764	2014239.783	2127880.746	1631477	1868364.164	1950229.006	1768785.896
PSMD6	26S proteasome non-ATPase regulatory subunit 6 isoform 2 [Homo sapiens]	0	2627642.781	3210119.395	2645991.324	3337154.427	2803936.009	2667601.249	0	2724554.601

PSMD7	26S proteasome non-ATPase regulatory subunit 7 [Homo sapiens]	2766811.449	3833899.318	4079514.473	3305984.287	4469226.584	4009761.443	3582637.785	3001881.388	4236208.12
PSMD8	26S proteasome non-ATPase regulatory subunit 8 [Homo sapiens]	2405128.569	2573120.708	2985810.641	2463952.296	2790488.561	2785031.985	2507676.224	1909889.155	2533906.306
PSMD9	26S proteasome non-ATPase regulatory subunit 9 isoform 1 [Homo sapiens]	1980290.102	2750471.107	1956572.626	2772546.176	1899264.851	2610725.844	2531845.706	2406808.143	2854819.854
PSME1	proteasome activator complex subunit 1 isoform 1 [Homo sapiens]	6177919.029	8604409.2	8459427.142	8421455.817	8243283.663	8869771.067	8337908.758	7201373.295	9293717.142
PSME2	proteasome activator complex subunit 2 [Homo sapiens]	7810024.723	7711849.893	8362150.256	6658266.764	8911787.428	9259776.468	8645730.543	7037702.412	7982767.496
PSME3	proteasome activator complex subunit 3 isoform 2 [Homo sapiens]	5890191.432	6922479.086	6806257.939	6019771.223	7512620.247	5630573.008	5777959.545	5767776.958	6180692.375
PSME4	proteasome activator complex subunit 4 [Homo sapiens]	93572.2769	95498.4528	92837.62754	100103.5459	126962.5883	151269.7862	88695.03082	60597.41543	90431.19708
PSMF1	proteasome inhibitor PI31 subunit [Homo sapiens]	1312461.667	1425533.992	1522186.806	1929173.258	1826827.121	1480079.508	1388763.348	1589250.844	1337383.207
PSMG1	proteasome assembly chaperone 1 isoform a [Homo sapiens]	2577335.199	2842629.291	3510841.683	2897245.538	3425607.542	2705331.341	2591590.73	2248847.347	4227520.667
PSMG2	proteasome assembly chaperone 2 isoform 1 [Homo sapiens]	2648755.631	1454385.087	2106447.069	1623632.298	1902327.19	1427947.944	1966297.703	2165797.471	1946106.112
PSMG3	proteasome assembly chaperone 3 [Homo sapiens]	3948815.102	4185005.228	5658851.963	4596458.374	5278342.135	2078561.688	2012947.035	3777824.035	4568396.228
PSMG4	proteasome assembly chaperone 4 isoform a [Homo sapiens]	2805386.648	1963470.885	3401535.152	3232964.631	3480620.35	3225082.942	2330060.527	2101875.017	2050542.241
PSPC1	paraspeckle component 1 [Homo sapiens]	1537706.528	1182743.811	1260567.404	969147.1907	1312305.666	1426310.37	951349.2648	759169.4709	1227761.678
PSPH	phosphoserine phosphatase [Homo sapiens]	2463689.546	3211256.209	3012650.65	3113322.996	3442091.221	3273821.023	3074211.687	2828721.804	2079507.826
PTBP1	polypyrimidine tract-binding protein 1 isoform a [Homo sapiens]	14118803.92	13144287.22	22082744.44	18017864.32	20854508.02	14101519.98	13277585.73	15640589.82	17531022.27
PTBP2	polypyrimidine tract-binding protein 2 [Homo sapiens]	0	0	0	0	0	0	0	0	0
PTBP3	polypyrimidine tract-binding protein 3 isoform 6 [Homo sapiens]	0	0	944335.1847	862964.2982	699926.8618	721251.8588	641537.2994	569779.9225	754971.9276
PTCD3	pentatricopeptide repeat domain-containing protein 3, mitochondrial precurs	349635.8962	373917.4743	346581.8541	379392.5899	488453.7536	303979.5493	279779.5446	280116.3962	403697.2562
PTDSS1	phosphatidylserine synthase 1 [Homo sapiens]	429485.87	410010.4436	413089.8104	530320.9933	601093.9567	485966.5633	455544.9075	376664.6039	296327.23
PTER	phosphotriesterase-related protein isoform 1 [Homo sapiens]	229495.9391	238459.6292	367942.167	283894.5279	449037.5393	338154.3688	316440.686	274270.3554	252960.561
PTGES	prostaglandin E synthase [Homo sapiens]	5589699.604	13780554.94	3634369.133	13945160.61	4587420.908	14359326.2	15508838.37	12882392.2	16639563.94
PTGES2	prostaglandin E synthase 2 isoform 1 [Homo sapiens]	1801526.001	2032598.845	2655351.666	2061064.081	2583169.302	2110331.181	2060782.696	1933778.013	2127922.966
PTGES3	prostaglandin E synthase 3 [Homo sapiens]	37359358.11	32393083.78	45967678.26	39062930.31	39007475.58	37846223.11	37517780.68	36500317.65	44446801.65
PTGES3L-AARSD1	PTGES3L-AARSD1 protein isoform 1 [Homo sapiens]	479684.308	626019.587	641469.9109	432122.445	544714.9828	576710.8004	604681.645	518816.3185	595075.5681
PTGFRN	prostaglandin F2 receptor negative regulator precursor [Homo sapiens]	69122.8894	171390.1547	139737.0539	134986.5644	156283.1653	159857.8344	139907.7234	82836.9176	147681.5521
PTGR1	prostaglandin reductase 1 isoform 1 [Homo sapiens]	3209191.408	3233127.295	4265012.707	3548908.173	4135516.084	3493915.761	3323609.572	2414847.575	3415499.661
PTGR2	prostaglandin reductase 2 [Homo sapiens]	71835.32332	108217.1611	256841.4412	113078.4688	398616.1587	140173.9789	115205.619	105685.2548	123192.3654
PTGS1	prostaglandin G/H synthase 1 isoform 1 precursor [Homo sapiens]	198145.4555	202952.1924	260796.1518	148961.9852	298026.8438	211410.9808	212951.7627	184329.8048	188976.1442
PTK2	focal adhesion kinase 1 isoform b [Homo sapiens]	56551.77548	81595.27176	91218.47176	72125.56501	0	82438.78548	73517.09795	49838.51412	77021.22313
PTMA	prothymosin alpha isoform 1 [Homo sapiens]	87099559.71	87576844.52	102568396.2	84880683.81	101363278.8	90753248.94	78865316.42	72938820.55	78972899.42
PTMS	parathymosin [Homo sapiens]	28746243.43	17845031.06	16481253.44	16460280.31	17345230.24	18004309.13	18019896.74	21614015.46	11816603.18
PTP4A1	protein tyrosine phosphatase type IVA 1 [Homo sapiens]	607262.7542	482589.2631	857859.6655	690818.5676	930471.5293	761155.317	662369.1042	517252.8334	766762.0822
PTP4A2	protein tyrosine phosphatase type IVA 2 isoform 1 [Homo sapiens]	861560.8099	1170787.641	1302520.752	990606.1197	1285639.007	1144074	0	885996.7297	861505.0441
PTPLAD1	very-long-chain (3R)-3-hydroxyacyl-[acyl-carrier protein] dehydratase 3 [Ho	1681873.573	3586696.1	2978434.629	2604931.113	3208298.657	2868345.726	2559334.896	2078557.412	3007598.46
PTPLB	very-long-chain (3R)-3-hydroxyacyl-[acyl-carrier protein] dehydratase 2 [Ho	981692.7315	288311.1712	0	3939574.8	742683.6579	17339.09344	22904.86896	124593.5194	4620421.493
PTPMT1	phosphatidylglycerophosphatase and protein-tyrosine phosphatase 1 isoform	954137.8438	874823.6554	949720.1658	849557.0321	921161.9679	915526.6602	920699.3238	740884	868252.4258
PTPN1	tyrosine-protein phosphatase non-receptor type 1 [Homo sapiens]	922079.0386	987594.0054	1217178.716	1031975.996	1149520.768	2724198.151	2904174.532	925364.7272	1053665.143
PTPN11	tyrosine-protein phosphatase non-receptor type 11 isoform 1 [Homo sapiens]	381113.2213	524421.2772	856438.3471	492808.4791	611754.6894	493769.4471	483389.5236	410885.3829	536742.8871
PTPN12	tyrosine-protein phosphatase non-receptor type 12 isoform 1 [Homo sapiens]	109138.2204	62341.95714	156222.6015	107853.1335	147014.4453	103340.532	144862.4427	0	108597.1431
PTPN2	tyrosine-protein phosphatase non-receptor type 2 isoform 1 [Homo sapiens]	306867.6676	320481.8364	372041.468	402714.2141	339063.6381	432738.8887	412540.3356	332483.5247	212109.0581
PTPN23	tyrosine-protein phosphatase non-receptor type 23 [Homo sapiens]	150581.4466	159227.8089	192741.6808	180435.5654	171344.5828	176352.7687	162455.6309	142053.2284	131540.2996
PTPN9	tyrosine-protein phosphatase non-receptor type 9 [Homo sapiens]	57801.28728	107666.9814	101901.2582	66632.05618	98860.76482	89129.5057	74607.83316	59675.40259	121500.2925
PTPRF	receptor-type tyrosine-protein phosphatase F isoform 1 precursor [Homo sapi	50191.74579	46181.88144	63741.23338	30618.3125	76538.00089	38095.97911	46002.69655	53394.23289	86908.80109
PTPRJ	receptor-type tyrosine-protein phosphatase eta isoform 1 precursor [Homo sa	0	0	0	0	0	0	44300.65263	40623.38026	64493.52895
PTPRS	receptor-type tyrosine-protein phosphatase S isoform 1 precursor [Homo sapi	35563.93427	0	36394.75344	41358.75997	38188.30468	0	120197.9987	14901.81814	80781.57164
PTRF	polymerase I and transcript release factor [Homo sapiens]	834168.7877	774868.8733	1041427.624	808809.5257	1176219.06	777623.8198	736537.992	700995.4225	640675.7561
PTRH1	probable peptidyl-tRNA hydrolase [Homo sapiens]	152161.121	155491.6452	227245.5881	161803.8374	288768.5233	246353.5917	155305.1445	149011.8387	415719.3394
PTRH2	peptidyl-tRNA hydrolase 2, mitochondrial precursor [Homo sapiens]	3281584.975	3169562.046	4137314.315	3361011.863	4091353.205	3680224.118	3590276.412	2486577.831	3415874.428
PTRHD1	putative peptidyl-tRNA hydrolase PTRHD1 [Homo sapiens]	3937434.157	3669165.931	4386487.378	2928670.256	5287597.598	3844344.198	3590426.263	2914420.835	3166461.13
PTS	6-pyruvoyl tetrahydrobiopterin synthase [Homo sapiens]	1476536.682	2456462.768	2737775.1	1507727.29	2562712.812	1952464.847	1879859.6	1527585.993	2545543.775
PTTG1IP	pituitary tumor-transforming gene 1 protein-interacting protein precursor	0	0	0	0	0	0	0	0	0
PUF60	poly(U)-binding-splicing factor PUF60 isoform e [Homo sapiens]	1343359.787	658894.942	0	349036.2104	0	644578.8665	1175985.057	566047.1518	661107.524
PUF60	poly(U)-binding-splicing factor PUF60 isoform b [Homo sapiens]	1343359.787	658894.942	0	349036.2104	0	644578.8665	1175985.057	566047.1518	661107.524
PUM1	pumilio homolog 1 isoform 1 [Homo sapiens]	93632.14435	119405.7668	116366.1491	0	122267.7857	0	62531.45015	94804.74195	108366.7434
PUM1	pumilio homolog 1 isoform 2 [Homo sapiens]	93632.14435	119405.7668	116366.1491	0	122267.7857	0	62531.45015	94804.74195	108366.7434
PUM2	pumilio homolog 2 [Homo sapiens]	111563.3168	82019.35873	116677.6104	104160.7842	146548.3545	347130.024	88547.31849	79451.33904	0
PURA	transcriptional activator protein Pur-alpha [Homo sapiens]	376018.5747	630528.1812	526172.0073	444449.3829	864528.3017	465796.5893	426368.5571	574090.3884	573423.2183
PURB	transcriptional activator protein Pur-beta [Homo sapiens]	879660.762	483569.7576	170997.3496	0	566556.9881	955696.4209	473272.6367	362558.7054	484694.3949
PUS1	tRNA pseudouridine synthase A, mitochondrial isoform 2 [Homo sapiens]	205043.9193	263107.3875	335182.6774	273653.3583	351388.7444	302380.1288	260414.2962	0	323952.3715
PUS1	tRNA pseudouridine synthase A, mitochondrial isoform 1 [Homo sapiens]	205043.9193	263107.3875	335182.6774	273653.3583	351388.7444	302380.1288	260414.2962	0	323952.3715
PUS3	tRNA pseudouridine(38/39) synthase isoform 1 [Homo sapiens]	0	0	0	0	0	0	0	34950.6966	153678.5584
PUS7	pseudouridylate synthase 7 homolog [Homo sapiens]	524764.9349	638027.3346	727735.1801	658181.9904	809033.5212	625333.4178	658429.0839	561597.988	713026.5932
PUS7L	pseudouridylate synthase 7 homolog-like protein isoform a [Homo sapiens]	0	0	0	0	0	0	8041.133122	12467.47554	0
PVR	poliovirus receptor isoform alpha precursor [Homo sapiens]	904629.4896	812494.9173	703939.276	593873.3438	738797.4063	799293.138	632732.3808	434184.9463	718948.3347
PVRL2	poliovirus receptor-related protein 2 isoform delta precursor [Homo sapiens	0	0	0	0	0	0	135076.9229	100787.7394	0
PWP1	periodic tryptophan protein 1 homolog [Homo sapiens]	1378952.003	1460867.799	1588755.359	1552106.375	1744350.329	1707864.965	1582406.585	1435277.337	1565027.309
PWP2	periodic tryptophan protein 2 homolog [Homo sapiens]	448851.9789	539740.7657	654704.6079	531828.0368	638171.3186	441641.9695	550790.5428	473332.8138	503082.7151
PXN	paxillin isoform 3 [Homo sapiens]	279171.7829	320007.3184	462687.4934	382434.1454	562463.5142	377633.5502	348590.4566	291886.3847	339092.0971
PYCR1	pyrroline-5-carboxylate reductase 1, mitochondrial isoform 1 [Homo sapiens]	2142365.445	1960178.047	2985050.284	1933601.33	2606960.273	2179467.519	2334096.668	1746103.67	2044550.698
PYCR2	pyrroline-5-carboxylate reductase 2 isoform 1 [Homo sapiens]	1665373.99	1830009.241	2031055.178	1817547.397	1992908.607	1477254.691	1932494.873	1366253.617	1752664.774
PYCRL	pyrroline-5-carboxylate reductase 3 [Homo sapiens]	463367.1638	735367.9312	1105541.395	1046173.648	1148084.239	638686.2589	723266.529	368144.7757	460810.728
PYGB	glycogen phosphorylase, brain form [Homo sapiens]	491133.2564	288472.9651	311531.1131	267607.6344	428532.2304	293051.3958	328778.6589	268299.04	329604.0115
PYGL	glycogen phosphorylase, liver form isoform 1 [Homo sapiens]	672317.974	933498.9922	932606.019	727456.2807	1350986.987	784157.3385	679587.4721	503435.364	997930.3648
PYGO2	pygopus homolog 2 [Homo sapiens]	0	0	0	0	0	0	58530.94477	364844.3765	494042.4477
PYROXD1	pyridine nucleotide-disulfide oxidoreductase domain-containing protein 1 [H	94491.7454	44956.49432	129993.6222	48214.91193	80517.6804	0	42624.21094	110294.4517	47135.90625
QARS	glutamine--tRNA ligase isoform a [Homo sapiens]	1878007.74	2015366.271	2201817.338	1884623.529	2329236.177	2403448.699	2055322.805	1928597.102	1777150.78
QDPR	dihydropteridine reductase [Homo sapiens]	1870478.145	2599651.943	2873843.43	2370574.351	2767462.295	2644143.422	2581939.07	1714086.558	2331646.62
QKI	protein quaking isoform HQK-5 [Homo sapiens]	349318.3653	461077.2787	514620.0574	432411.3716	421031.1193	400263.5747	483487.7796	370360.2407	111237.7926
QPCTL	glutaminyl-peptide cyclotransferase-like protein isoform 1 [Homo sapiens]	115367.3957	129793.453	446330.1053	54573.11842	455582.7218	0	0	58898.09586	315096.8995
QPRT	nicotinate-nucleotide pyrophosphorylase [carboxylating] precursor [Homo sap	267004.4331	407435.7827	514597.4613	343152.4011	0	519333.9792	459985.0238	624779.5863	492832.445
QRICH1	glutamine-rich protein 1 [Homo sapiens]	434044.0421	539882.8829	461747.6014	441486.8544	445823.1666	513606.4391	463590.3903	316229.9241	402396.7308
QSER1	glutamine and serine-rich protein 1 [Homo sapiens]	36768.06706	25298.07319	40899.93774	33206.92172	56527.73577	47480.03113	19555.63192	25594.18617	26447.13045
QSOX2	sulfhydryl oxidase 2 precursor [Homo sapiens]	173139.0032	199878.9154	226164.0576	173525.6894	184439.2396	190909.3402	211351.7004	171284.7138	226844.5884
QTRT1	queuine tRNA-ribosyltransferase [Homo sapiens]	807106.0235	863283.222	1066734.711	710024.2273	1056254.173	977051.2765	885833.2826	709171.2345	933750.2326
QTRTD1	queuine tRNA-ribosyltransferase subunit QTRTD1 isoform 2 [Homo sapiens]	300549.9307	373843.9135	359532.1875	505156.4704	576548.0403	523288.8381	542746.1619	276241.2262	382028.4242
R3HDM4	R3H domain-containing protein 4 [Homo sapiens]	0	0	0	0	0	0	0	0	0
RAB10	ras-related protein Rab-10 [Homo sapiens]	4694404.199	4849406.507	6293188.086	4939132.378	6051084.98	5299341.432	5222505.11	3968782.282	6299044.451
RAB11A	ras-related protein Rab-11A isoform 1 [Homo sapiens]	0	413152.6613	90506.70699	119403.707	230326.5148	46310.53428	196532.7111	0	92513.67742
RAB11B	ras-related protein Rab-11B [Homo sapiens]	11274917.98	3109559.743	3411183.223	3047125.021	3298587.002	3311999.06	2755098.967	2589591.384	3046228.438
RAB11FIP1	rab11 family-interacting protein 1 isoform 3 [Homo sapiens]	41790.52561	71396.2667	0	69159.41566	63582.31292	68360.94878	63355.81599	23569.03007	52385.01183
RAB11FIP5	rab11 family-interacting protein 5 [Homo sapiens]	31728.24714	38963.17366	68361.7958	42695.93225	0	56044.10832	34570.60115	22915.62977	95949.15267
RAB12	ras-related protein Rab-12 [Homo sapiens]	167450.9797	180795.3674	242881.4228	203572.58	216541.1311	215204.7739	189409.06	136442.7576	169742.3882
RAB13	ras-related protein Rab-13 isoform 1 [Homo sapiens]	2296584.6	3508293.415	2960312.707	3173510.927	3096813.726	1818480.386	1693339.835	2025314.24	1807322.086
RAB14	ras-related protein Rab-14 [Homo sapiens]	4953580.698	5924794.069	5249749.025	4918178.133	5999336.312	5176095.859	4472895.188	3549566.793	5712946.086
RAB18	ras-related protein Rab-18 isoform 1 [Homo sapiens]	2168371.324	2608309.638	2584279.019	3398467.085	2915897.82	2850756.32	3042926.617	2488862.866	0
RAB1A	ras-related protein Rab-1A isoform 1 [Homo sapiens]	3965553.437	4631238.92	5580066.393	3929390.443	5450901.685	5066325.955	4840153.769	3821597.297	5402077.762
RAB1B	ras-related protein Rab-1B [Homo sapiens]	6617647.657	6978967.101	7875757.532	7507181.925	7673123.695	8031513.543	7817068.279	5755029.198	7429127.701
RAB21	ras-related protein Rab-21 [Homo sapiens]	1322736.879	1723222.818	1333496.902	1657955.617	1711283.306	1893899.084	1510137.767	1538377.695	1097845.883
RAB22A	ras-related protein Rab-22A [Homo sapiens]	0	0	0	0	0	0	202628.2602	96112.56395	0
RAB23	ras-related protein Rab-23 [Homo sapiens]	52689.92647	490925.2886	590626.0135	230387.7718	456597.4623	285014.1644	325298.3183	313460.3039	497095.6814
RAB27A	ras-related protein Rab-27A [Homo sapiens]	0	0	0	0	0	0	97074.26724	592801.7701	600952.6207
RAB27B	ras-related protein Rab-27B [Homo sapiens]	184802.9947	219103.6461	345570.3006	216792.5225	528167.0464	0	223076.4888	0	221664.9691
RAB2A	ras-related protein Rab-2A isoform a [Homo sapiens]	8357548.598	7507459.623	10896760.09	5706456.488	6996826.374	10103171.41	10140310.16	5798688.28	7221084.59
RAB31	ras-related protein Rab-31 [Homo sapiens]	664161.4715	582756.8882	549155.4367	1674283.141	819695.1219	1659726.271	956822.4099	607238.9661	1172873.408
RAB32	ras-related protein Rab-32 [Homo sapiens]	850744.384	651090.0292	1253511.918	955862.5543	816714.1612	847287.6728	930929.3737	418224.3102	1248446.192
RAB34	ras-related protein Rab-34 isoform 1 [Homo sapiens]	979925.4336	1240605.52	1284361.129	1236366.676	1306237.67	1442508.947	1320083.24	967102.7741	1416358.813
RAB35	ras-related protein Rab-35 isoform 1 [Homo sapiens]	14122582.59	14616921.4	17492628.92	15486731.59	15717628.34	14654305.78	14991269.17	14870774.41	16632798.69
RAB3A	ras-related protein Rab-3A [Homo sapiens]	256475.8384	358522.0823	0	319871.1993	290550.811	256297.9695	0	0	0
RAB3GAP1	rab3 GTPase-activating protein catalytic subunit isoform 2 [Homo sapiens]	340958.1644	389588.09	170428.7837	404491.6922	443181.9995	221034.6181	218769.96	0	714829.459
RAB3GAP2	rab3 GTPase-activating protein non-catalytic subunit [Homo sapiens]	118662.1436	177135.7091	159170.9915	131892.968	163867.9882	158143.2457	167714.0028	139333.9207	169982.0272
RAB4A	ras-related protein Rab-4A isoform 1 [Homo sapiens]	839241.8068	514510.2935	347528.331	591317.0299	587934.8949	492989.1181	1541519.194	546546.7124	1005861.932
RAB5A	ras-related protein Rab-5A [Homo sapiens]	2754302.921	2804184.244	3593130.63	2916476.744	3987593.262	3070632.383	2976566.277	2234533.579	3137125.091
RAB5B	ras-related protein Rab-5B isoform 1 [Homo sapiens]	2165201.909	1477726.469	1815419.378	2338597.152	2329290.641	1695581.979	2197092.677	1691478.331	2104990.091
RAB5C	ras-related protein Rab-5C isoform b [Homo sapiens]	7388380.963	7673539.984	7726893.792	6956202.352	7166929.451	7327267.666	7485984.924	5534210.373	6484412.302
RAB6A	ras-related protein Rab-6A isoform a [Homo sapiens]	1658108.452	680021.1827	1046132.737	885719.4578	1004598.861	1212106.013	1067899.781	996993.0368	900367.9579
RAB6A	ras-related protein Rab-6A isoform b [Homo sapiens]	1658108.452	680021.1827	1046132.737	885719.4578	1004598.861	1212106.013	1067899.781	996993.0368	900367.9579
RAB7A	ras-related protein Rab-7a [Homo sapiens]	16329265.98	16889425.34	20520304.09	16990030.95	20689591.96	19373752.62	18722478.55	15908693.61	20207400.81
RAB8A	ras-related protein Rab-8A [Homo sapiens]	1661441.404	2260584.45	1399634.476	2595320.704	2008170.766	2667624.738	2461203.847	2071558.728	1653730.375
RAB8B	ras-related protein Rab-8B [Homo sapiens]	1121917.147	0	557837.6767	1503615.805	408912.9567	1575496.098	1500604.859	1362949.522	434453.8053
RAB9A	ras-related protein Rab-9A [Homo sapiens]	1328692.362	1950177.094	2354115.5	826771.4642	1567961.076	1341509.108	1388242.033	1419671.305	1869822.1

RABEP1	rab GTPase-binding effector protein 1 isoform 1 [Homo sapiens]	148220.6034	119299.1649	183306.9038	132473.9354	181438.947	156436.9071	94319.41689	128468.7875	76223.21223
RABEP2	rab GTPase-binding effector protein 2 [Homo sapiens]	124630.5001	75369.72401	90389.25057	89167.29846	138817.4916	87915.71291	54087.4363	105331.6993	91430.80837
RABEPK	rab9 effector protein with kelch motifs isoform a [Homo sapiens]	120594.3803	243912.4431	151683.0841	80307.25	298204.0415	142024.2591	148171.6145	0	120915.0466
RABGAP1	rab GTPase-activating protein 1 [Homo sapiens]	80347.92182	54634.80804	90330.19804	58957.0691	131972.4229	76141.73409	92590.65243	68968.50297	50520.05539
RABGAP1L	rab GTPase-activating protein 1-like isoform A [Homo sapiens]	15918.73217	38253.92869	32565.5151	57979.69044	0	79914.84691	65679.43289	12949.50881	68823.17114
RABGEF1	rab5 GDP/GTP exchange factor [Homo sapiens]	100547.8328	92547.2883	66788.85957	38094.59437	0	86060.49894	81200.16532	98544.72872	0
RABGGTA	geranylgeranyl transferase type-2 subunit alpha [Homo sapiens]	382721.4281	232314.8521	446752.1608	1571682.218	299261.9789	185880.8932	294789.1682	1521001.07	344465.9304
RABGGTB	geranylgeranyl transferase type-2 subunit beta [Homo sapiens]	1150864.197	1119357.148	1365710.304	1206120.125	1480059.263	1245479.668	1093172.316	1022091.257	1199218.797
RABIF	guanine nucleotide exchange factor MSS4 [Homo sapiens]	1671147.668	2127023.886	2051822.664	2565752.894	2150576.274	1480886.968	861431.8857	1861469.039	2023358.723
RABL3	rab-like protein 3 [Homo sapiens]	550677.9534	295617.9281	554988.1116	430445.7599	699489.9427	448169.75	457832.059	330539.5349	510255.6551
RAC1	ras-related C3 botulinum toxin substrate 1 isoform Rac1b [Homo sapiens]	6690532.495	6526353.499	7534314.259	6753966.821	4327414.046	7061199.52	6609859.113	5654282.36	7333827.927
RAC2	ras-related C3 botulinum toxin substrate 2 [Homo sapiens]	548094.5171	988182.1781	1274382.37	752311.0092	1223349.651	1047984	1226762.932	844212.3579	1172130.781
RACGAP1	rac GTPase-activating protein 1 [Homo sapiens]	580439.556	581457.8619	686738.4881	585052.1052	603823.0631	547261.5608	594479.7884	499301.9254	487475.2082
RAD1	cell cycle checkpoint protein RAD1 [Homo sapiens]	414474.4695	228060.2674	904415.2384	0	0	988067.5959	351863.2093	0	618648.1807
RAD18	E3 ubiquitin-protein ligase RAD18 [Homo sapiens]	230369.7739	249651.056	284888.0277	260606.4687	304082.5486	263741.916	278484.4315	200308.7212	183546.7014
RAD21	double-strand-break repair protein rad21 homolog [Homo sapiens]	551422.759	569779.1151	708548.6245	595405.3844	808344.6816	542326.275	705769.9698	478599.7302	528649.7123
RAD23A	UV excision repair protein RAD23 homolog A isoform 1 [Homo sapiens]	2770608.983	3196428.526	3134828.577	2984822.296	2356994.183	3030100.844	3108313.157	2666633.624	1976648.056
RAD23B	UV excision repair protein RAD23 homolog B isoform 1 [Homo sapiens]	4814807.692	5561041.587	6337730.657	5999938.533	5659819.804	6155564.388	5857859.694	5027338.75	5793986.472
RAD50	DNA repair protein RAD50 [Homo sapiens]	109616.3828	103233.9474	127587.2393	106510.9646	180296.8253	104688.5231	92377.79457	79635.09881	127531.6319
RAD51	DNA repair protein RAD51 homolog 1 isoform 2 [Homo sapiens]	61571.32593	0	280752.3471	154400.3306	167751.5331	151526.0103	0	111283.8678	208604.4494
RAD54B	DNA repair and recombination protein RAD54B isoform 1 [Homo sapiens]	51839.29545	0	63631.37032	50647.33957	63387.05348	78430.83824	0	48837.38503	73599.31217
RAD54L2	helicase ARIP4 [Homo sapiens]	5457.426236	2590.116037	15385.19864	0	16151.49419	0	0	0	0
RAD9A	cell cycle checkpoint control protein RAD9A isoform 1 [Homo sapiens]	110849.8848	142432.5282	171070.6391	0	126386.5511	0	0	0	0
RAE1	mRNA export factor [Homo sapiens]	2923601.824	2244644.847	4022928.364	2361876.229	3787566.658	3491706.986	3320802.445	2965285.681	3420161.761
RAF1	RAF proto-oncogene serine/threonine-protein kinase [Homo sapiens]	0	0	0	0	0	0	50854.73552	0	90954.88158
RAI1	retinoic acid-induced protein 1 [Homo sapiens]	15042.48728	16182.14886	21236.90763	20718.86608	17731.44669	19303.87873	11905.19064	18731.71932	15715.15427
RAI14	ankycorbin isoform d [Homo sapiens]	0	31965.15885	7189.430618	31440.60245	0	42362.93202	64701.16883	23404.61517	158319.3393
RALA	ras-related protein Ral-A precursor [Homo sapiens]	3529699.117	4589750.471	4356160.055	4002792.315	2588690.482	4094832.255	4488622.879	3442064.849	3438194.982
RALB	ras-related protein Ral-B [Homo sapiens]	459169.3211	583920.3184	672729.5632	663530.798	797334.5026	598981.9069	584072.3316	604598.9211	0
RALGAPA1	ral GTPase-activating protein subunit alpha-1 isoform 2 [Homo sapiens]	25212.52246	16700.44645	0	27332.08172	0	34044.0696	74221.34932	26277.9305	17421.52766
RALGAPB	ral GTPase-activating protein subunit beta [Homo sapiens]	4969.035326	25187.5199	18010.15613	33725.63208	40048.39575	22858.42458	0	0	0
RALY	RNA-binding protein Raly isoform 1 [Homo sapiens]	1309011.414	1682112.135	1548442.741	1415588.636	1714230.491	1556628.739	1384805.003	1380156.712	1810524.967
RAN	GTP-binding nuclear protein Ran [Homo sapiens]	67251674.45	73991466.51	84494864.91	73722690.77	83073281.91	74530077.19	75690761.44	69437886.53	81447711.86
RANBP1	ran-specific GTPase-activating protein [Homo sapiens]	22195264.31	22776391.05	26403196.16	25375301.75	19064600.42	26861507.28	26628127.55	21930398.43	25322193.68
RANBP2	E3 SUMO-protein ligase RanBP2 [Homo sapiens]	137958.013	179697.3009	193869.206	458851.7379	202604.5255	170473.1528	138427.3372	129151.3929	189392.3273
RANBP3	ran-binding protein 3 isoform RANBP3-a [Homo sapiens]	323186.8038	315303.1352	63814.04409	340868.0944	71678.58385	63262.84913	309724.6981	42097.74935	336671.2936
RANBP3	ran-binding protein 3 isoform RANBP3-d [Homo sapiens]	323186.8038	315303.1352	63814.04409	340868.0944	71678.58385	63262.84913	309724.6981	42097.74935	336671.2936
RANBP6	ran-binding protein 6 isoform 1 [Homo sapiens]	920929.4827	889633.9747	312235.1464	962220.5791	190327.5295	1011445.944	903934.7052	746447.2338	900353.1099
RANBP9	ran-binding protein 9 [Homo sapiens]	299915.6637	326996.2239	513489.7619	455929.9116	494962.6252	382453.1752	375984.8903	321195.0705	525095.5141
RANGAP1	ran GTPase-activating protein 1 [Homo sapiens]	1803405.708	2216641.402	2337660.04	1951040.61	2448776.809	2086660.999	1981983.655	3162421.613	2394952.48
RANGRF	ran guanine nucleotide release factor isoform A [Homo sapiens]	595778.8889	1264312.808	448646.0153	1299453.457	1084731.886	1417682.9	1321237.206	2319119.273	1102780.359
RAP1A	ras-related protein Rap-1A [Homo sapiens]	2468435.848	2145439.016	1795379.919	1680774.062	1439667.238	2206075.171	2149022.321	1772673.54	2286745.832
RAP1B	ras-related protein Rap-1b isoform 1 precursor [Homo sapiens]	3135059.167	5046385.137	0	4252566.52	4075881.584	4365068.366	4770325.526	3656210.913	0
RAP1GDS1	rap1 GTPase-GDP dissociation stimulator 1 isoform 1 [Homo sapiens]	428274.8469	464169.8521	0	486644.6438	0	493500.7745	420886.3538	372125.54	422549.2433
RAP2B	ras-related protein Rap-2b [Homo sapiens]	743339.5303	773734.2045	1214221.008	863742.0821	1020361.715	846171.6768	809617.6742	795384.5524	1012261.43
RAP2C	ras-related protein Rap-2c isoform 1 precursor [Homo sapiens]	0	0	0	0	0	0	1524214.956	613020.4559	1443790.406
RAPGEF6	rap guanine nucleotide exchange factor 6 isoform 1 [Homo sapiens]	0	23844.72134	26811.38171	23811.28726	18299.36153	14937.29252	13842.47181	7277.595564	4336.664179
RAPH1	ras-associated and pleckstrin homology domains-containing protein 1 isofor	123302.3433	102549.4581	167916.9989	142780.7861	166477.5652	490893.9799	152927.8646	98167.39647	102106.4367
RARS	arginine--tRNA ligase, cytoplasmic [Homo sapiens]	1839835.014	2100853.405	2200535.057	2203713.058	2467155.073	2265768.372	2134755.338	1731889.545	2165057.156
RASA1	ras GTPase-activating protein 1 isoform 1 [Homo sapiens]	50386.58209	70373.6669	72799.44505	0	55262.6075	83981.73266	97165.74901	37751.04555	53696.68786
RASAL2	ras GTPase-activating protein nGAP isoform 2 [Homo sapiens]	12721.47108	25774.27328	44180.71846	19878.1946	21196.58945	17034.09079	24108.62218	32546.13631	27034.75435
RAVER1	ribonucleoprotein PTB-binding 1 [Homo sapiens]	766206.24	786885.0067	975403.8904	852818.7767	880480.8046	864598.5513	607361.6146	451647.1763	635839.8608
RB1	retinoblastoma-associated protein [Homo sapiens]	23237.71068	19728.91928	36851.16944	21717.92312	34267.33568	23861.89066	0	0	0
RBBP4	histone-binding protein RBBP4 isoform a [Homo sapiens]	3755121.048	4138540.881	5159321.913	3821338.834	2915370.908	4449500.942	3845708.325	3001145.67	4591042.742
RBBP5	retinoblastoma-binding protein 5 isoform 1 [Homo sapiens]	217927.9131	285989.4558	287330.1692	362104.6983	358064.7726	298759.0974	318114.1382	273530.5992	175573.7967
RBBP6	E3 ubiquitin-protein ligase RBBP6 isoform 1 [Homo sapiens]	21628.6942	16093.73812	56562.19494	29521.60304	38742.76786	31469.73097	25277.84309	26753.40635	37531.81498
RBBP7	histone-binding protein RBBP7 isoform 2 [Homo sapiens]	6527489.899	7141168.338	8898858.765	6945276.899	8230700.895	7118130.441	6438723.02	5644045.434	7401332.149
RBFOX2	RNA binding protein fox-1 homolog 2 isoform 5 [Homo sapiens]	672690.9402	545621.937	551947.6774	714813.8884	984092.0239	763860.1368	623910.7308	538980.6859	357551.7911
RBKS	ribokinase [Homo sapiens]	44026.46165	251883.8295	165464.5653	174341.7301	130068.3835	185343.6477	0	0	0
RBM10	RNA-binding protein 10 isoform 5 [Homo sapiens]	78760.23721	66586.82231	92360.40708	22860.96379	226433.2589	20856.38969	188973.8793	148027.2885	132506.9193
RBM10	RNA-binding protein 10 isoform 3 [Homo sapiens]	78760.23721	66586.82231	92360.40708	22860.96379	226433.2589	20856.38969	188973.8793	148027.2885	132506.9193
RBM12	RNA-binding protein 12 [Homo sapiens]	679002.2983	712355.6668	899036.0218	688266.6269	929409.3937	775230.255	714177.0597	651576.3357	787169.4412
RBM12B	RNA-binding protein 12B [Homo sapiens]	85163.34915	89280.89954	104375.5052	51785.00707	129821.6012	95672.10186	97001.04612	64837.26188	57403.08481
RBM14	RNA-binding protein 14 isoform 1 [Homo sapiens]	2651108.666	2944120.201	3628011.701	3242516.88	4035913.969	3029046.658	2326720.041	1931587.28	2653823.607
RBM15	putative RNA-binding protein 15 isoform 1 [Homo sapiens]	105693.9847	55157.23461	162815.946	83718.11228	117010.9091	115186.1869	72999.48758	363651.576	136607.686
RBM17	splicing factor 45 [Homo sapiens]	596179.4899	398850.3157	564991.4722	726556.1207	545977.0076	873584.8929	770525.0162	384946.0321	767033.8247
RBM19	probable RNA-binding protein 19 [Homo sapiens]	91242.84818	69886.23793	73755.524	71558.31379	122682.4701	64758.72855	84177.74851	64811.13037	117569.8542
RBM22	pre-mRNA-splicing factor RBM22 [Homo sapiens]	615195.5201	502807.5421	601626.648	511018.0084	736407.6056	652408.3464	565162.7084	493441.5112	747621.7207
RBM25	RNA-binding protein 25 [Homo sapiens]	782306.3911	631357.1127	1088111.228	760215.9674	1002223.254	735104.7613	737652.0038	545383.7634	783200.1626
RBM26	RNA-binding protein 26 [Homo sapiens]	219811.0235	156243.0503	240384.7673	184776.5118	289391.2523	193948.099	191671.1211	181537.3582	152575.992
RBM27	RNA-binding protein 27 [Homo sapiens]	123565.6963	117822.2375	168174.525	132895.1267	161315.3052	142103.1204	206459.1803	90684.25658	110008.0378
RBM28	RNA-binding protein 28 isoform 1 [Homo sapiens]	309386.0367	356374.9717	392147.4613	371633.937	331744.5479	373525.6566	190554.5099	302791.6618	283247.0665
RBM3	putative RNA-binding protein 3 [Homo sapiens]	15423293.61	18563638.83	20259575.12	27480442.62	17558833.64	20870030.83	13050377.34	16099294.13	17143083.36
RBM33	RNA-binding protein 33 [Homo sapiens]	76014.72566	16695.28092	103294.373	51238.30758	59159.77292	18393.46053	0	0	0
RBM34	RNA-binding protein 34 isoform 1 [Homo sapiens]	260719.7587	317276.3134	338596.7398	300799.2607	354414.1453	343257.8831	263984.8295	233299.2304	297323.1211
RBM39	RNA-binding protein 39 isoform b [Homo sapiens]	2612065.661	2927272.052	3464851.933	2816842.204	3297758.359	3152641.655	2714627.312	2330567.664	2877921.68
RBM4	RNA-binding protein 4 isoform 1 [Homo sapiens]	2137262.507	2063917.246	0	2132984.899	0	2105838.423	2004236.655	968311.746	2044572.215
RBM42	RNA-binding protein 42 [Homo sapiens]	730144.4364	724642.7265	796793.8426	727446.6296	936261.2914	788114.0914	682427.8389	590261.8194	635236.5185
RBM45	RNA-binding protein 45 [Homo sapiens]	73388.14371	49834.96732	65703.14279	51431.82934	71696.63558	73218.02157	47425.93306	24862.35017	73538.70655
RBM47	RNA-binding protein 47 isoform a [Homo sapiens]	85066.47108	186355.0907	185272.4492	37597.45305	187180.8925	75473.03759	161712.0877	68189.42897	215212.2566
RBM4B	RNA-binding protein 4B [Homo sapiens]	244028.7704	263843.9937	2160878.393	198449.6185	2067584.836	212822.1654	210619.8444	157917.7111	71531.87407
RBM5	RNA-binding protein 5 [Homo sapiens]	138032.5126	94068.89691	64861.18349	110201.738	20934.30887	169582.9455	82120.93755	38648.36774	71685.2781
RBM6	RNA-binding protein 6 isoform 1 [Homo sapiens]	0	17426.82839	50836.74815	106715.9686	22483.5107	18542.63797	15995.62077	32085.97861	29492.96212
RBM7	RNA-binding protein 7 [Homo sapiens]	278114.3867	205477.0855	614465.2632	315490.5297	168904.6842	638516.2456	0	268527.1469	436100.8761
RBMS1	RNA-binding motif, single-stranded-interacting protein 1 isoform a [Homo sa	649263.4363	711232.5049	433446.882	962151.7415	867380.6054	1066995.256	882801.6838	1056141.245	1498563.054
RBMS2	RNA-binding motif, single-stranded-interacting protein 2 [Homo sapiens]	0	0	0	0	0	0	645700.7918	0	524751.0372
RBMX	RNA-binding motif protein, X chromosome isoform 1 [Homo sapiens]	1903585.142	6399555.54	2172706.05	2216905.598	1896003.035	2234046.17	2168218.93	1997905.509	22597.78069
RBMXL1	RNA binding motif protein, X-linked-like-1 [Homo sapiens]	195858.4292	0	252583.2178	229504.6958	237518.7169	237538.9778	224794.8305	122866.4575	238119.8927
RBPJ	recombining binding protein suppressor of hairless isoform 1 [Homo sapiens]	168970.5089	208337.2179	196635.48	154084.8438	225852.0803	264400.79	0	0	0
RBPJL	recombining binding protein suppressor of hairless-like protein [Homo sapie	0	0	0	0	0	0	199269.5914	154631.6343	0
RBX1	E3 ubiquitin-protein ligase RBX1 [Homo sapiens]	12458871.38	413254.4494	15855177.41	13715714.78	16821213.39	14400848.14	6435966.284	9989476.797	14599335.9
RCC1	regulator of chromosome condensation isoform a [Homo sapiens]	4922218.501	5024736.152	5687721.278	5802764.389	7844682.272	6301387.049	5051523.485	4856873.177	6048622.489
RCC2	protein RCC2 [Homo sapiens]	2419051.821	2695947.477	3165904.743	2883581.528	3277436.212	2903727.624	2867522.25	2394350.127	2712291.97
RCCD1	RCC1 domain-containing protein 1 [Homo sapiens]	216554.0879	312152.974	346870.9325	208335.5412	331052.6674	316673.3648	288037.7415	138498.3492	203972.6447
RCE1	CAAX prenyl protease 2 isoform 1 [Homo sapiens]	0	0	0	0	0	0	0	0	0
RCL1	RNA 3'-terminal phosphate cyclase-like protein [Homo sapiens]	454752.1103	305206.4437	269437.257	34419.56379	315618.5552	118376.183	59422.14634	0	483425.1759
RCN1	reticulocalbin-1 precursor [Homo sapiens]	4745076.703	5550382.993	5758613.686	5062292.782	6449464.449	5146313.809	5588349.558	4645781.242	6028032.902
RCN2	reticulocalbin-2 isoform b precursor [Homo sapiens]	1902300.073	2008716.238	2757785.043	1512174.324	2786884.483	1629916.971	2900508.59	2525965.859	2813424.945
RCOR1	REST corepressor 1 [Homo sapiens]	422895.0753	947294.0387	536079.5062	520778.1794	451670.8452	818061.251	490773.5245	406410.162	412710.3942
RCOR3	REST corepressor 3 isoform a [Homo sapiens]	18334.81076	17114.7623	24191.33536	0	25424.32697	19304.71036	14436.54282	0	19231.81756
RDBP	negative elongation factor E [Homo sapiens]	368119.3496	461556.3186	411454.8252	436683.9647	449973.9254	463890.3199	378889.7657	356807.2234	504354.6956
RDH11	retinol dehydrogenase 11 isoform 1 precursor [Homo sapiens]	1943372.384	2127859.2	2747850.732	1483691.677	3177115.966	2019520.97	2380542.342	1988388.371	2280536.462
RDH13	retinol dehydrogenase 13 isoform 1 precursor [Homo sapiens]	280639.0991	353478.5972	333536.6549	282504.9427	375876.1409	349343.6501	349185.8763	231865.7767	183270.7572
RDH14	retinol dehydrogenase 14 [Homo sapiens]	0	0	0	0	0	0	0	0	0
RDX	radixin isoform 1 [Homo sapiens]	0	1508279.504	1659725.11	1439457.286	1700473.374	1682350.171	1355824.089	1325670.266	1576105.617
RECQL	ATP-dependent DNA helicase Q1 [Homo sapiens]	840211.2244	1015686.346	1055304.019	1009057.268	1268469.244	967666.6052	1123856.891	908613.6981	1208166.356
REEP3	receptor expression-enhancing protein 3 [Homo sapiens]	0	52283.06027	109061.1295	146982.0838	104240.4196	193321.2857	131957.3066	141806.3248	186310.5079
REEP4	receptor expression-enhancing protein 4 precursor [Homo sapiens]	0	350018.3391	269046.2674	287517.087	602706.6957	0	300482.0435	308553.3946	63249.69565
REEP5	receptor expression-enhancing protein 5 [Homo sapiens]	3017734.997	3507430.637	43452.74208	6671680.81	150749.1132	2397693.135	7256498.352	2752277.354	3627076.876
REEP6	receptor expression-enhancing protein 6 [Homo sapiens]	1639808.789	1533946.684	1807981.305	1433678.465	1981138.85	1571872.758	1596421.836	1248299.785	1483045.148
RELA	transcription factor p65 isoform 1 [Homo sapiens]	676830.689	1054436.121	1287677.463	768296.1901	1001931.035	828178.436	1287062.1	1007645.531	1135367.649
REPIN1	replication initiator 1 isoform 3 [Homo sapiens]	140430.5549	130090.555	211996.884	149768.3401	244263.8903	137373.2555	103835.0125	93028.57132	134991.7022
REPS1	ralBP1-associated Eps domain-containing protein 1 isoform a [Homo sapiens]	0	0	0	0	0	0	121398.4622	43720.66379	70111.83172
RER1	protein RER1 [Homo sapiens]	1875878.833	3071355.089	2481835.588	2175004.706	2642755.401	1428704.056	3323161.836	1951296.56	3184441.014
RETSAT	all-trans-retinol 13,14-reductase precursor [Homo sapiens]	479130.1667	458356.0542	673540.3266	509943.6059	456847.8133	544818.8823	560239.802	382943.0134	486509.5574
REXO2	oligoribonuclease, mitochondrial precursor [Homo sapiens]	2589495.277	2732386.51	3156425.372	3164163.043	3308212.214	3074564.839	3136986.952	2587285.823	2557209.074
REXO4	RNA exonuclease 4 [Homo sapiens]	181435.1452	198453.4934	163984.1553	160936.9408	165888.9169	123851.0791	169177.7593	121713.5162	226757.0979
RFC1	replication factor C subunit 1 isoform 2 [Homo sapiens]	85739.3094	49245.60207	114666.6121	72783.93374	134837.0376	88155.06564	91018.41624	65957.58533	48834.13277
RFC2	replication factor C subunit 2 isoform 1 [Homo sapiens]	1162271.709	1302935.856	1479642.723	1288547.101	1570961.625	1377471.804	1207732.583	926165.9839	1203908.528
RFC3	replication factor C subunit 3 isoform 1 [Homo sapiens]	694935.0032	773381.6095	1014468.691	785923.8956	1076550.742	809180.3696	778286.3329	635207.5652	832210.1994

RFC4	replication factor C subunit 4 [Homo sapiens]	737781.5671	674909.7555	851769.8713	1030726.384	963839.9701	1094238.991	944561.1512	809513.9256	1090371.206
RFC5	replication factor C subunit 5 isoform 1 [Homo sapiens]	1035714.073	1018291.443	1117735.89	1065257.201	1426215.48	1091891.09	769492.1755	849394.4706	730068.9119
RFK	riboflavin kinase [Homo sapiens]	289841.5522	312994.1841	539743.4643	367075.7332	471186.493	659371.4554	383426.4295	277178.58	205766.1111
RFT1	protein RFT1 homolog [Homo sapiens]	0	0	0	0	0	0	185028.0745	287028.8596	482529.4195
RFTN1	raftlin [Homo sapiens]	11420.37429	13110.94849	0	0	23098.46168	21946.77575	0	0	0
RFX1	MHC class II regulatory factor RFX1 [Homo sapiens]	95828.51129	52825.92249	162159.5533	77898.11494	153077.2811	104637.3764	95676.57708	146888.926	47607.23605
RFX5	DNA-binding protein RFX5 [Homo sapiens]	28337.39796	0	70426.33909	27158.97545	69893.04411	44356.20425	37288.05091	42161.81636	17647.37659
RFXAP	regulatory factor X-associated protein [Homo sapiens]	74349.21173	85717.27833	0	41704.42969	70801.45625	60653.2211	58027.42813	0	22073.9125
RGPD8	RANBP2-like and GRIP domain-containing protein 8 [Homo sapiens]	4726.167497	39449.69948	7001.570717	4584.875789	7176.776394	25388.73082	53706.2251	0	4492.849602
RGS10	regulator of G-protein signaling 10 isoform a [Homo sapiens]	0	373277.573	435876.0689	356635.1545	492603.4831	245210.6383	307178.3357	452322.8291	332772.0899
RHBDF2	inactive rhomboid protein 2 isoform 1 [Homo sapiens]	0	0	0	0	0	0	10744.71774	11411.26457	2599.111242
RHEB	GTP-binding protein Rheb [Homo sapiens]	4342681.194	5401313.517	5500420.641	5153590.879	4882113.714	4412340.336	5382325.645	4215235.824	5552694.048
RHOA	transforming protein RhoA precursor [Homo sapiens]	8738466.707	9301522.26	11521946.64	17996174.12	7311837.006	10099401.57	18862098.93	7042628.274	12309438.02
RHOB	rho-related GTP-binding protein RhoB precursor [Homo sapiens]	0	0	0	0	0	0	0	0	0
RHOC	rho-related GTP-binding protein RhoC precursor [Homo sapiens]	3538028.388	3586005.4	4595135.038	3551626.946	5066758.059	3884157.66	3817094.062	3228681.736	4493225.102
RHOF	rho-related GTP-binding protein RhoF precursor [Homo sapiens]	203462.9036	135516.8614	0	231434.0836	0	278415.1506	179252.6928	123804.0282	314515.6627
RHOG	rho-related GTP-binding protein RhoG precursor [Homo sapiens]	2839641.811	3287229.521	3533354.187	2896008.244	3751333.221	3328825	3469265.814	2922896.258	3630014.607
RHOT1	mitochondrial Rho GTPase 1 isoform 1 [Homo sapiens]	95748.04924	128112.6212	98392.60568	131942.922	170791.086	160995.3138	144813.5515	99335.43924	112266.5395
RHOT2	mitochondrial Rho GTPase 2 [Homo sapiens]	297295.0267	444630.7441	440123.2886	653721.7911	350321.6469	613779.1289	594716.5726	320659.2222	570472.7811
RHPN2	rhophilin-2 [Homo sapiens]	125695.5337	84750.53539	237849.7813	112554.6504	0	0	0	0	0
RIC8A	synembryn-A [Homo sapiens]	379371.3103	416951.5271	541555.3562	451006.396	532720.431	452886.4975	431426.0207	392546.633	437859.321
RICTOR	rapamycin-insensitive companion of mTOR [Homo sapiens]	27913.51416	23346.80669	46750.45525	22458.51683	37714.77672	34405.57846	29630.42488	21337.02325	27120.04006
RIF1	telomere-associated protein RIF1 isoform 1 [Homo sapiens]	85823.52336	108205.2652	140707.2917	107067.1292	262810.0611	120440.3954	108594.8825	95308.6298	99937.64702
RILP	rab-interacting lysosomal protein [Homo sapiens]	0	72369.69913	120457.3381	80132.37112	121275.6758	113084.486	63300.07143	42959.52096	27111.56522
RILPL1	RILP-like protein 1 [Homo sapiens]	52459.1828	232899.7119	62877.6428	95110.16398	332685.0529	84302.10419	59072.87737	0	201996.2963
RIN1	ras and Rab interactor 1 [Homo sapiens]	187279.5832	134481.125	122307.9792	130550.5798	245221.8231	145185.7405	198422.727	136965.1596	205045.8909
RING1	E3 ubiquitin-protein ligase RING1 [Homo sapiens]	405584.0006	353417.345	380394.8218	459635.5911	377048.4961	503094.6047	404651.782	376177.1562	405760.2867
RINT1	RAD50-interacting protein 1 [Homo sapiens]	0	0	0	0	0	0	0	0	0
RIOK1	serine/threonine-protein kinase RIO1 isoform 1 [Homo sapiens]	422431.3895	409455.7919	558342.4225	466263.1539	621385.093	554425.5484	499252.6124	384083.2793	231027.2587
RIOK2	serine/threonine-protein kinase RIO2 isoform 1 [Homo sapiens]	98804.1614	131684.1227	132485.2294	90943.56081	149934.4186	102125.5924	97515.13405	105653.9799	79288.32995
RIOK3	serine/threonine-protein kinase RIO3 [Homo sapiens]	0	0	0	0	0	0	0	0	0
RIPK1	receptor-interacting serine/threonine-protein kinase 1 [Homo sapiens]	451389.5943	571790.9565	207815.9348	220976.6119	224615.7283	981022.9707	211637.2276	387586.2762	546724.6504
RLIM	E3 ubiquitin-protein ligase RLIM [Homo sapiens]	7942.052333	13652.62004	0	8062.365575	0	11665.61136	0	0	0
RMDN1	regulator of microtubule dynamics protein 1 [Homo sapiens]	304681.4555	605651.2436	530021.1743	674296.6008	869387.3944	442546.8321	788552.0762	479604.8548	552784.9901
RMDN3	regulator of microtubule dynamics protein 3 [Homo sapiens]	337118.7304	375802.6671	433836.9098	431962.1121	445001.7582	375110.9264	189811.0187	301503.0223	380634.1274
RMND5A	protein RMD5 homolog A [Homo sapiens]	118065.2658	89816.21127	183131.8508	624549.9789	348134.1197	693183.115	605774.092	132186.4361	193298.6778
RNASEH1	ribonuclease H1 precursor [Homo sapiens]	171878.2398	0	243747.6356	320457.2815	361761.0824	349881.0516	284082.8452	0	316906.8373
RNASEH2A	ribonuclease H2 subunit A [Homo sapiens]	757868.6163	721994.0685	945932.1826	933787.2157	892537.027	926466.7548	829426.273	890032.4496	992917.4817
RNASEH2B	ribonuclease H2 subunit B isoform 1 [Homo sapiens]	122851.6573	443005.0347	831109.4825	499454.66	441602.9021	320372.292	425579.4025	234119.2308	538205.8997
RNASEH2C	ribonuclease H2 subunit C [Homo sapiens]	1719798.043	457704.9184	2206026.346	1119912.579	2156623.307	1264493.2	557440.3149	1334413.472	1162467.623
RND3	rho-related GTP-binding protein RhoE precursor [Homo sapiens]	0	0	0	0	0	0	0	0	0
RNF103-CHMP3	RNF103-CHMP3 protein [Homo sapiens]	1275157.258	1270123.57	1753973.614	1365206.646	1742607.698	1453211.355	1264080.757	1004544.93	0
RNF113A	RING finger protein 113A [Homo sapiens]	207137.4591	146517.4594	0	292334.771	0	317042.2254	294898.2085	220177.5616	504773.4025
RNF114	RING finger protein 114 [Homo sapiens]	683035.6662	635112.3746	645302.3823	694153.3852	790922.2602	624726.3618	633438.3601	653770.3451	954281.8062
RNF121	RING finger protein 121 [Homo sapiens]	114238.7791	160779.1456	1020778.34	102570.0631	0	0	148803.659	142904.6189	145624.1262
RNF123	E3 ubiquitin-protein ligase RNF123 [Homo sapiens]	39423.68626	34454.26289	32570.06513	0	48291.44255	50658.76266	38151.13045	0	30349.30106
RNF126	RING finger protein 126 [Homo sapiens]	0	62169.83604	117230.5795	0	1968060.701	1672924.753	0	0	0
RNF13	E3 ubiquitin-protein ligase RNF13 [Homo sapiens]	387147.8966	0	602995.5603	452385.9914	686903.6466	0	0	0	0
RNF14	E3 ubiquitin-protein ligase RNF14 isoform 1 [Homo sapiens]	25660.29565	200893.2116	0	73821.82353	78020.9281	0	89375.25327	34216.15952	49816.1252
RNF167	E3 ubiquitin-protein ligase RNF167 precursor [Homo sapiens]	64683.31388	55471.14938	91184.14973	0	106563.4162	69630.15634	65032.06663	67092.82933	98170.48592
RNF168	E3 ubiquitin-protein ligase RNF168 [Homo sapiens]	23577.53648	0	26236.11409	0	25891.14284	6334.827047	0	0	0
RNF169	E3 ubiquitin-protein ligase RNF169 [Homo sapiens]	0	0	0	0	0	0	0	0	0
RNF181	E3 ubiquitin-protein ligase RNF181 [Homo sapiens]	1935271.559	1921331.243	2620247.406	2183172.676	2666440.733	1482968.577	1529697.746	1537480.724	0
RNF2	E3 ubiquitin-protein ligase RING2 [Homo sapiens]	50622.74016	193461.5857	121561.1336	125314.03	121469.4055	236133.5731	143665.3996	143212.2491	155106.8731
RNF20	E3 ubiquitin-protein ligase BRE1A [Homo sapiens]	183743.0718	315338.1947	110618.2866	143955.1298	129616.0762	141066.5505	117935.917	104709.7844	127487.905
RNF213	E3 ubiquitin-protein ligase RNF213 isoform 3 [Homo sapiens]	16678.47731	18093.22282	28832.10572	20494.55295	40873.19506	20872.65201	23918.59551	16548.62662	19048.38023
RNF214	RING finger protein 214 isoform 1 [Homo sapiens]	68814.17696	63758.37308	73698.82784	58520.74359	84633.68773	84896.24985	59588.01432	50518.04304	66439.3315
RNF25	E3 ubiquitin-protein ligase RNF25 [Homo sapiens]	0	127780.5684	0	284781.7221	36159.75662	138261.9905	0	0	0
RNF40	E3 ubiquitin-protein ligase BRE1B isoform 1 [Homo sapiens]	170173.0706	164285.7799	216519.8193	182156.1773	274457.8643	194048.9697	188949.5294	157878.8323	152192.0637
RNF5	E3 ubiquitin-protein ligase RNF5 [Homo sapiens]	450521.0119	554191.7209	734164.2571	504695.2947	778655.8578	596450.2069	456649.3372	616844.4138	447873.5867
RNF7	RING-box protein 2 isoform 1 [Homo sapiens]	656579.4894	1065973.933	1756945.214	893001.5286	1874987.4	1144993.475	907067.4894	696398.4823	1570161.169
RNGTT	mRNA-capping enzyme [Homo sapiens]	33009.41225	80673.24339	118178.9668	74179.39328	87145.44018	93506.30258	62494.03598	44323.70742	101538.3542
RNH1	ribonuclease inhibitor [Homo sapiens]	3216259.594	4190853.677	3835420.617	3588568.676	4085855.913	3438704.567	4046689.06	3366287.744	4553786.368
RNMT	mRNA cap guanine-N7 methyltransferase [Homo sapiens]	325128.7233	407174.5082	391396.5892	366290.2615	296668.7761	374619.2248	373583.6403	349314.2674	333372.1126
RNMTL1	RNA methyltransferase-like protein 1 [Homo sapiens]	220405.5089	215037.2398	206521.6841	234227.9901	307530.5072	259362.3434	335932.8777	183204.9063	346221.7734
RNPEP	aminopeptidase B [Homo sapiens]	1479234.101	1910438.61	2364591.527	2293371.623	2697850.288	2017091.298	2249829.84	1894272.584	2289053.033
RNPS1	RNA-binding protein with serine-rich domain 1 [Homo sapiens]	2317518.087	2289060.512	3088784.961	2871811.449	3246843.313	2986926.8	2657564.012	2138338.213	2739709.045
ROBO1	roundabout homolog 1 isoform a precursor [Homo sapiens]	0	0	0	0	0	0	15407.80045	0	24102.98482
ROBO2	roundabout homolog 2 isoform ROBO2b precursor [Homo sapiens]	0	0	0	0	0	0	0	0	0
ROCK1	rho-associated protein kinase 1 [Homo sapiens]	36194.33994	51969.60983	52348.94848	20646.41655	20297.81422	26953.7426	36726.64456	168765.4536	14660.373
ROCK2	rho-associated protein kinase 2 [Homo sapiens]	206891.5341	427375.8294	270523.9659	219198.9942	301667.6726	234349.8977	203935.0199	159780.0289	200367.8134
ROMO1	reactive oxygen species modulator 1 [Homo sapiens]	7516776.46	10134891.84	10207842.16	8914544.88	0	9158112.22	8303896.58	5894847.772	8466322.972
RP2	protein XRP2 [Homo sapiens]	406189.017	381097.4796	377995.1125	304038.29	409680.3595	374812.8665	510789.284	311996.8825	309891.73
RPA1	replication protein A 70 kDa DNA-binding subunit [Homo sapiens]	924320.8682	1176719.244	1363688.331	1094634.967	1269667.967	1289720.767	1051397.417	869939.9736	1098307.906
RPA2	replication protein A 32 kDa subunit [Homo sapiens]	2443820.56	3426173.167	2342984.312	2372609.647	2352015.071	2516446.409	2472435.059	1702702.609	2517149.553
RPA3	replication protein A 14 kDa subunit [Homo sapiens]	6172851.755	5080661.138	7621552.21	6806638.95	7305342.283	6881528.976	4084556.345	6004312.876	4797954.124
RPAP1	RNA polymerase II-associated protein 1 [Homo sapiens]	44062.55161	18880.474	52273.76969	10081.64516	67086.3746	25364.73592	39683.80655	18869.26188	13331.54739
RPAP2	putative RNA polymerase II subunit B1 CTD phosphatase RPAP2 [Homo sapiens]	37068.69164	30080.92527	64705.70644	49676.03915	48619.85516	38289.47541	34598.0089	0	25981.98132
RPAP3	RNA polymerase II-associated protein 3 isoform 1 [Homo sapiens]	432717.7311	473694.0292	582452.1622	570259.4702	607266.4724	632458.9933	590657.2883	481769.2835	499058.5819
RPE	ribulose-phosphate 3-epimerase isoform 1 [Homo sapiens]	2126229.148	1832392.982	2885961.777	2075161.347	3014183.626	1570141.411	1930156.3	2409120.239	1954892.426
RPF1	ribosome production factor 1 [Homo sapiens]	386229.5464	109693.1548	469173.8613	346665.3706	562355.8429	299107.9142	405381.4868	133949.7195	169645.2304
RPF2	ribosome production factor 2 homolog [Homo sapiens]	1078976.517	709753.1542	1283693.773	639272.7524	517364.2101	852971.6114	746821.1018	541411.0124	926921.8789
RPIA	ribose-5-phosphate isomerase [Homo sapiens]	343902.2697	270737.8922	339388.9942	372804.777	317835.4681	385543.0083	423101.4605	353615.7995	425581.6997
RPL10	60S ribosomal protein L10 isoform a [Homo sapiens]	8145344.784	10166131.16	12379132.68	8483755.73	12999067.63	10641676.42	9718852.516	8680130.098	10905708.26
RPL10A	60S ribosomal protein L10a [Homo sapiens]	18890366.35	19307932.34	28465769.38	17365420.26	26148839.58	21546854.72	20444532.06	17779712.31	18796910.46
RPL11	60S ribosomal protein L11 isoform 1 [Homo sapiens]	18781611.78	35757925.27	41176311.2	22722984.31	34573516.78	23760456.3	33620265.3	17989927.32	39158497.81
RPL12	60S ribosomal protein L12 [Homo sapiens]	42463005.71	58855003.03	58087087.15	57214692.05	61310462	56231808.15	57463855.37	44696003.79	59985155.81
RPL13	60S ribosomal protein L13 isoform 1 [Homo sapiens]	21953271.51	27828739.18	29868068.66	28227845.32	28567576.5	28506519.45	27718385.47	22240152.92	30870712.1
RPL13A	60S ribosomal protein L13a isoform 1 [Homo sapiens]	20743129.85	19512812.9	27520209.45	20609948.93	27970580.14	21254961.11	20211430.59	17768552.94	21712046.08
RPL14	60S ribosomal protein L14 [Homo sapiens]	23396523.51	25109806.74	20006565.41	26935781.09	31667528.76	21643602.08	19316536.11	21650701.4	16392329.54
RPL15	60S ribosomal protein L15 isoform 1 [Homo sapiens]	15903291.06	19253782.84	18545278.3	20637992.39	21124071.71	20026743.43	19823038.43	14864097.18	21405469.29
RPL17-C18orf32	RPL17-C18orf32 protein isoform 1 [Homo sapiens]	24064963.34	24106547.28	31157319.5	26261976.96	35816228.6	29201021.9	24775079.97	20182248.05	24852981.28
RPL18	60S ribosomal protein L18 isoform 1 [Homo sapiens]	36928193.39	38495349.41	51106184.94	40336606.63	52269494.43	44745539.97	40739530.3	35907110.43	44021359.83
RPL18A	60S ribosomal protein L18a [Homo sapiens]	13543969.19	14249490.63	18470200.42	15639316.45	18925647.86	16323839.92	15479030.57	12799394.11	18470497.15
RPL19	60S ribosomal protein L19 [Homo sapiens]	6430808.499	8874844.569	8589207.292	8197233.314	9148036.088	10068310.58	8862976.124	21346602.57	8895972.796
RPL21	60S ribosomal protein L21 [Homo sapiens]	15132290.05	13901468.49	19702002.44	12720586.82	19809825.83	16528309.06	13025326.32	4716452.813	12042203.1
RPL22	60S ribosomal protein L22 proprotein [Homo sapiens]	61586040.63	66209970.72	77860668.65	67291851.27	81790270.55	66379576.2	66517839	57931910	72654369.55
RPL23	60S ribosomal protein L23 [Homo sapiens]	53542212.55	67362785.73	74322054.67	64661641.39	76812212.51	73004333.99	63611737.1	53365732.04	68769212.51
RPL23A	60S ribosomal protein L23a [Homo sapiens]	31495116.59	29439812.3	37495351.97	34054687.79	39446515.23	36406182.16	33002810.36	27718467.01	32465780.74
RPL24	60S ribosomal protein L24 [Homo sapiens]	18273799.35	25048326.02	25944110.57	23595951.4	26506481.63	27047051.63	23337816.65	20037621.8	16392632.87
RPL26	60S ribosomal protein L26 [Homo sapiens]	11283237.81	14150736.22	16073549.98	15188971.09	18323463.67	15335677.97	14810300.05	12062095.81	14602703.28
RPL27	60S ribosomal protein L27 [Homo sapiens]	30276156.12	31083348.84	36479596.68	31230533.8	40811183.09	33753900.57	32333427.54	27819094.05	31657690.64
RPL27A	60S ribosomal protein L27a [Homo sapiens]	28574931.01	35516182.7	39736647.43	38382249.09	41436544.74	44286167.32	38455138.91	35571878.96	46125093.96
RPL28	60S ribosomal protein L28 isoform 2 [Homo sapiens]	6935741.973	10494193.1	11200631.4	14857281	11900049.25	10881392.45	6463096.807	0	9582628.583
RPL29	60S ribosomal protein L29 [Homo sapiens]	40371678.36	39319821.17	30844179.98	46382769.42	20941840.68	47382624.23	40163952.77	1327765.283	21565060.99
RPL3	60S ribosomal protein L3 isoform a [Homo sapiens]	12447338.61	14830322.87	15055355.15	13997146.99	15574432.59	14990281.53	14123713.29	12815839.01	14028677.72
RPL30	60S ribosomal protein L30 [Homo sapiens]	24687825.34	33070878.02	31779715.87	33905877.96	31735490.94	33573246.02	34201652.3	25221207.26	31803389.98
RPL31	60S ribosomal protein L31 isoform 2 [Homo sapiens]	23664417.67	24013037.68	30747036.63	26720643.09	31608157.15	26782841.15	34996066.62	34070926.79	21998266.72
RPL32	60S ribosomal protein L32 [Homo sapiens]	9734033.773	9855215.185	11863919.49	10228653.46	10589121.64	10627126.86	10472495.76	7838449.549	11026568.18
RPL34	60S ribosomal protein L34 [Homo sapiens]	9497741.931	17619740.96	222565.261	9002408.749	441215.953	9871364.091	12065646.75	13486699.75	0
RPL35	60S ribosomal protein L35 [Homo sapiens]	3811565.269	5832511.332	889705.5894	2910101.347	906902.9335	3658282.775	5245576.275	11289660.25	1675168.505
RPL35A	60S ribosomal protein L35a [Homo sapiens]	8659015.189	13207033.04	9429045.251	75883.46979	11068253.83	9892838.315	12610933.55	11172094.76	8054185.723
RPL36	60S ribosomal protein L36 [Homo sapiens]	25845944.84	32341925.36	32916499.45	32116118.2	33825090.07	34809975.87	16875137.83	14171679.71	34434868.54
RPL36A-HNRNPH2	RPL36A-HNRNPH2 protein isoform a [Homo sapiens]	119606.1864	160294.5669	203294.2998	71429.86737	0	148888.8423	0	16361.78264	27660.45487
RPL36AL	60S ribosomal protein L36a-like [Homo sapiens]	0	0	0	0	0	0	166997.8738	0	30070.57223
RPL37	60S ribosomal protein L37 [Homo sapiens]	0	0	0	0	0	0	27771.95671	35166.11415	0
RPL37A	60S ribosomal protein L37a [Homo sapiens]	17304391.98	20421955.57	20907550.76	30361281.92	25048412.69	19017080.77	18899753.07	15684524.31	19976867.59
RPL38	60S ribosomal protein L38 [Homo sapiens]	29582073.4	39790869.18	40362931.07	41932334.87	40649198.73	43826353.87	38038613.22	29751837.6	37734826.89
RPL39	60S ribosomal protein L39 [Homo sapiens]	17251469.48	21705680.63	22476755.35	26956149.73	21941147.46	24885927.9	0	18399653.28	21521473.68
RPL4	60S ribosomal protein L4 [Homo sapiens]	12888082.63	17392447.04	16951555.96	17060500.03	18276763.1	17952088.94	17157584.59	14926926.79	16940313.06

RPL5	60S ribosomal protein L5 [Homo sapiens]	17327671.81	17363772.65	21493829.55	18177582.38	24100588.54	19103806.59	17084368.11	14178052.79	20676542.97
RPL6	60S ribosomal protein L6 [Homo sapiens]	15146593.99	16272489.69	16622715.19	18068686.48	17630729.14	18611484.54	16306426.17	13525160.84	17814328.54
RPL7	60S ribosomal protein L7 [Homo sapiens]	20669731.36	23742010.52	27063981.79	23523812.87	24460674.15	23993169.4	23329954.86	20381992.06	25857293.74
RPL7A	60S ribosomal protein L7a [Homo sapiens]	22300142.28	25668362.06	29132768.15	23659987.6	30879639.96	25843824.75	25333568.41	20451324.09	27393706.39
RPL7L1	60S ribosomal protein L7-like 1 [Homo sapiens]	1392903.333	1375024.864	2320138.537	1813266.834	2077180.082	1869698.017	1617132.968	1066100.767	1497589.469
RPL8	60S ribosomal protein L8 [Homo sapiens]	15176374.13	15438107.94	21432306.68	17900641.21	23869497.37	19112651.26	17838315.9	7614863.524	16002654.18
RPL9	60S ribosomal protein L9 [Homo sapiens]	30601591.84	34871748.44	39603098.35	34994146.77	29650865.67	39512571.81	34857671.65	28466374.77	36084117.83
RPLP0	60S acidic ribosomal protein P0 [Homo sapiens]	41472727.78	49631333.97	55535406.07	50534522.18	56263890.02	52495607.39	47031956.93	42238752.31	47964693.54
RPLP1	60S acidic ribosomal protein P1 isoform 1 [Homo sapiens]	296946746.3	247247286.4	396226663.5	298820050.1	359831883.7	84266510.43	293119707.4	279087995.5	268253737.2
RPLP2	60S acidic ribosomal protein P2 [Homo sapiens]	139127004	173456038.1	188827715.7	162569910.3	172678823.3	176173099.2	160987094.9	154803275.9	180809589.6
RPN1	dolichyl-diphosphooligosaccharide--protein glycosyltransferase subunit 1 pr	5116326.992	6067872.265	6691456.752	5836219.218	6369576.071	6184377.731	5883149.328	4641161.458	6194994.521
RPN2	dolichyl-diphosphooligosaccharide--protein glycosyltransferase subunit 2 is	4389022.652	4577172.227	5218701.919	4927880.351	5654065.114	4717153.94	4202636.811	3957465.522	4507822.078
RPP14	ribonuclease P protein subunit p14 [Homo sapiens]	1141084.846	1063817.495	1679550.718	753043.2181	0	677308.524	858361.0332	577801.8883	1144359.011
RPP25	ribonuclease P protein subunit p25 [Homo sapiens]	953652.1926	881871.54	1039700.932	0	1089783.182	0	750872.5034	802200.5699	951689.1892
RPP30	ribonuclease P protein subunit p30 isoform a [Homo sapiens]	735797.2364	996602.3874	1046537.319	1074179.733	1068496.228	1064749.334	940279.2881	706882.6734	1089946.89
RPP38	ribonuclease P protein subunit p38 [Homo sapiens]	259093.2301	189249.2512	344743.3537	304107.424	592436.9422	166819.5876	320229.3894	288440.0451	99910.77306
RPP40	ribonuclease P protein subunit p40 [Homo sapiens]	489369.0505	752719.8253	1048049.237	820076.2996	724725.3372	722691.5424	717458.4364	632257.368	796209.6674
RPRD1A	regulation of nuclear pre-mRNA domain-containing protein 1A [Homo sapiens]	558086.5306	318089.4367	548386.3048	464535.7676	559238.1888	556139.7739	593542.1818	416086.1827	312949.9388
RPRD1B	regulation of nuclear pre-mRNA domain-containing protein 1B [Homo sapiens]	896407.3036	1278069.489	1267928.625	1140365.219	1330228.945	1076142.743	842683.6209	922394.1223	1199048.591
RPRD2	regulation of nuclear pre-mRNA domain-containing protein 2 [Homo sapiens]	134691.1819	155877.058	455239.4786	172268.6089	460106.3192	127388.8516	111493.0384	130651.5864	138186.7675
RPS10	40S ribosomal protein S10 [Homo sapiens]	16403419.86	14604469.42	36196461.42	18291065.56	33444951.59	17739179.29	14921450.37	22680063.74	23359974.37
RPS10-NUDT3	RPS10-NUDT3 protein [Homo sapiens]	696590.5489	991984.0908	0	854650.4069	0	931306.687	0	0	0
RPS11	40S ribosomal protein S11 [Homo sapiens]	13026617.71	16546282.92	15753839.86	15891963.02	16193904.89	16956688.66	15528765.86	13028153.14	17487383.63
RPS12	40S ribosomal protein S12 [Homo sapiens]	46851010.21	52026469.36	60450449.7	51589341.05	57467412.9	53172140.48	53797477.46	46218867.15	54376867.56
RPS13	40S ribosomal protein S13 [Homo sapiens]	44872791.1	45060141.84	58890114.05	46542917.09	59071559.85	50028280.13	44555432.28	39326600.84	47598484.26
RPS14	40S ribosomal protein S14 [Homo sapiens]	35692976.55	45232419.38	50042666.52	45833372.42	51583723.09	47266072.86	46070341.61	38873091.11	48018633.44
RPS15	40S ribosomal protein S15 [Homo sapiens]	25381645.88	28811201.93	33685170.65	28849908.01	36145219.51	30943026.48	27268336.03	14048320.02	22793035.71
RPS15A	40S ribosomal protein S15a [Homo sapiens]	41939404.9	51201276.03	53274860.53	49282978.4	53157218.72	50211981.93	50823725.88	40472431.19	54967209.47
RPS16	40S ribosomal protein S16 [Homo sapiens]	31584782.96	35570790.84	43843937.52	35674046.01	43357519.54	39100036.57	35756291.24	32156049.47	37784373.81
RPS17	40S ribosomal protein S17 [Homo sapiens]	37254427.59	38568159.54	48923708.76	40859348.74	49888552.43	42307824.17	26951289.16	35403619.96	39969230.7
RPS18	40S ribosomal protein S18 [Homo sapiens]	30269513.02	38249962.21	36899387.89	36492524.25	39994071.8	37614958.61	36687311.79	31258970.76	38101326.76
RPS19	40S ribosomal protein S19 [Homo sapiens]	39418105.8	48300845.85	47559034.86	45205604.85	49500901.3	49231138.1	46473290.53	37680272.63	43142113.47
RPS19BP1	active regulator of SIRT1 [Homo sapiens]	324348.6	439963.7882	0	454959.7088	397421.6706	499879.6206	383275.5412	328813.7692	290703.1882
RPS2	40S ribosomal protein S2 [Homo sapiens]	27849465.88	30248008.71	38027258.22	32571994.01	38665338.15	34471908.54	29752623.1	26640829.5	33716561.09
RPS20	40S ribosomal protein S20 isoform 1 [Homo sapiens]	33003572.71	0	40004770.15	53778800.35	43333265.08	37336548.04	34286625.03	47389411.72	36043200.65
RPS21	40S ribosomal protein S21 [Homo sapiens]	51308230.52	63773634.95	73393226.95	65342220.6	70765362.71	62372043.21	61428432.52	51334910.05	59132227.1
RPS23	40S ribosomal protein S23 [Homo sapiens]	13663849.05	14187515.87	18154561.04	17137495.16	19918700.72	18238693.53	17709752.45	17023324.04	17681113.06
RPS24	40S ribosomal protein S24 isoform d [Homo sapiens]	13995258.28	17888030.38	20037130.69	18729337.43	21041979.07	20046101.97	18926993.59	13869230.95	19003515.98
RPS25	40S ribosomal protein S25 [Homo sapiens]	43296746.67	51047010.42	54781444.11	53442977.26	57960030.29	49807438.87	53250838.36	44305521.5	56212448.55
RPS26	40S ribosomal protein S26 [Homo sapiens]	7022394.28	19397220.45	26910376.89	9539828.017	34925541.03	19789165.52	18240824.3	11403782.97	2225601.859
RPS27	40S ribosomal protein S27 [Homo sapiens]	22069008.45	25898310.51	28138584.85	22153248.37	49670863.26	38823945.53	21287134.4	18089228.83	19503621.81
RPS27A	ubiquitin-40S ribosomal protein S27a precursor [Homo sapiens]	107770878.2	137624747.3	133838942.9	137575631.5	118354349.5	148629493.1	147365225.6	140338495.9	166459994.2
RPS27L	40S ribosomal protein S27-like [Homo sapiens]	13284253.53	16130310.35	17170168.24	6877661.559	17994518.35	0	7002303.747	9998931.765	15256705.88
RPS28	40S ribosomal protein S28 [Homo sapiens]	59313349.55	80557158.89	42713610.55	44643208.87	42476248.68	50085036.89	47559299.11	42952395.21	60973126.63
RPS29	40S ribosomal protein S29 isoform 2 [Homo sapiens]	0	0	0	0	0	0	0	0	0
RPS3	40S ribosomal protein S3 isoform 1 [Homo sapiens]	37138829.6	41628989.76	50390925.67	41680680.94	49512560.92	38905121.12	42204765.98	35091396.99	40032537.17
RPS3A	40S ribosomal protein S3a isoform 1 [Homo sapiens]	20045993.34	22629954.38	25812849.97	23257674.62	25643193.53	23505712.97	23092636.97	20002634.61	25475914.62
RPS4X	40S ribosomal protein S4, X isoform X isoform [Homo sapiens]	21457523.58	24601375.34	31945631.77	26482756.47	32809016.27	29521669.12	27245595.19	23788715.67	22556451.05
RPS5	40S ribosomal protein S5 [Homo sapiens]	22331812.96	28707878.65	28485631.28	29360469.95	27626697.44	31958572.8	28545421.05	25528210.54	27835981.18
RPS6	40S ribosomal protein S6 [Homo sapiens]	18783959.41	24173504.09	26607076.54	22348826.61	25036123.08	24222755.9	23323365.35	19481877.44	26879906.38
RPS6KA1	ribosomal protein S6 kinase alpha-1 isoform b [Homo sapiens]	268769.9665	292632.5504	339954.7234	297471.5369	313817.7123	322191.6339	313668.4601	189680.9177	412254.1867
RPS6KA3	ribosomal protein S6 kinase alpha-3 [Homo sapiens]	411644.1135	603824.2625	560615.1524	556966.1895	603334.3514	498048.3459	509416.7595	424226.8574	614858.323
RPS6KA4	ribosomal protein S6 kinase alpha-4 isoform a [Homo sapiens]	100203.0364	0	116453.758	17959.54202	138796.5204	177687.8627	27023.20098	59037.62444	66887.04536
RPS6KA5	ribosomal protein S6 kinase alpha-5 isoform a [Homo sapiens]	0	0	0	0	0	0	0	0	0
RPS6KB1	ribosomal protein S6 kinase beta-1 isoform a [Homo sapiens]	0	0	0	0	0	0	104964.6212	130334.1192	0
RPS6KB2	ribosomal protein S6 kinase beta-2 [Homo sapiens]	0	0	0	0	0	0	0	0	0
RPS7	40S ribosomal protein S7 [Homo sapiens]	35245657.66	34715252.86	42170628.36	38480960.47	38847616.85	37055611.58	32616163.12	31382534.19	34494629.75
RPS8	40S ribosomal protein S8 [Homo sapiens]	29976598.24	34350111.04	39650687.08	29455645.71	42123768.96	37747561.15	30757205.68	27841802.6	32648679.28
RPS9	40S ribosomal protein S9 [Homo sapiens]	20641518.28	24293101.56	26868569.67	24259770.42	25833209.5	25746365.97	24574906.74	20709032.43	27806967.78
RPSA	40S ribosomal protein SA [Homo sapiens]	61005447.69	62193643.64	84097016.14	66258735.08	83756756.84	67481757.4	63307195.6	53190695.28	68542137.67
RPTOR	regulatory-associated protein of mTOR isoform 1 [Homo sapiens]	54990.98529	59914.52527	80625.08748	34069.11173	63636.91273	35845.4157	25620.02309	0	55025.90547
RPUSD2	RNA pseudouridylate synthase domain-containing protein 2 [Homo sapiens]	75053.54242	224814.4195	220145.6139	74220.00533	243488.3244	161815.7228	113730.2575	51935.25357	143786.6519
RPUSD3	RNA pseudouridylate synthase domain-containing protein 3 isoform 1 [Homo sa	0	0	0	0	0	0	124237.2113	149258.4371	221760.0811
RPUSD4	RNA pseudouridylate synthase domain-containing protein 4 isoform a [Homo sa	0	66024.1009	0	60888.76434	149517.9398	81461.92247	56528.12199	101972.5798	50591.73343
RQCD1	cell differentiation protein RCD1 homolog isoform 2 [Homo sapiens]	572569.2606	670767.8873	0	606838.2911	865213.7121	0	565411.3871	0	724244.1409
RRAGA	ras-related GTP-binding protein A [Homo sapiens]	262153.6806	279027.2686	313565.1929	243324.9269	264101.308	227833.1788	206837.7223	204717.7981	0
RRAGC	ras-related GTP-binding protein C isoform 1 [Homo sapiens]	274821.8627	0	343007.8991	266987.2795	362441.9802	0	0	305245.566	256825.5779
RRAS2	ras-related protein R-Ras2 isoform a [Homo sapiens]	620576.2017	1123439.811	2723343.612	881687.1281	2732190.939	2504840.472	2173900.805	809679.375	2642223.59
RRBP1	ribosome-binding protein 1 [Homo sapiens]	324739.345	458689.2995	577219.3129	465693.5975	531586.0834	518882.1898	446954.8741	385444.2388	489450.0841
RREB1	ras-responsive element-binding protein 1 isoform 1 [Homo sapiens]	0	0	0	0	0	0	0	18124.58343	25709.00873
RRM1	ribonucleoside-diphosphate reductase large subunit [Homo sapiens]	3115968.123	3799445.42	4203245.974	3580928.42	4285787.57	3780540.548	3674172.043	2965691.316	3797092.056
RRM2	ribonucleoside-diphosphate reductase subunit M2 isoform 1 [Homo sapiens]	1113986.655	1479568.176	1644525.23	1349725.076	1910160.793	1478745.405	869793.3434	641377.406	844491.6918
RRM2B	ribonucleoside-diphosphate reductase subunit M2 B isoform 1 [Homo sapiens]	147884.8907	0	123493.1246	87772.33595	137700.3251	98387.0703	83273.7149	84217.1764	0
RRM2B	ribonucleoside-diphosphate reductase subunit M2 B isoform 2 [Homo sapiens]	147884.8907	0	123493.1246	87772.33595	137700.3251	98387.0703	83273.7149	84217.1764	0
RRN3	RNA polymerase I-specific transcription initiation factor RRN3 [Homo sapier	65726.1881	140656.6965	111202.4913	88413.25737	143434.1401	98963.73642	55520.90483	17910.30913	22618.73491
RRP1	ribosomal RNA processing protein 1 homolog A [Homo sapiens]	452096.3541	721479.8575	631179.3614	761470.357	555838.4995	774281.3478	717612.8014	442787.3156	785538.258
RRP12	RRP12-like protein isoform 1 [Homo sapiens]	317742.6461	302257.0197	382208.0308	338820.9961	387537.8108	343264.2906	525709.3339	282418.1484	381633.7925
RRP15	RRP15-like protein [Homo sapiens]	1118628.855	925853.2121	717944.3454	832624.8369	642842.9296	626470.1362	557982.7545	1028594.481	1049110.265
RRP1B	ribosomal RNA processing protein 1 homolog B [Homo sapiens]	370261.2724	330380.7585	543223.3806	403445.1894	554480.6673	394455.6658	332988.367	312995.65	327791.6303
RRP36	ribosomal RNA processing protein 36 homolog [Homo sapiens]	225657.6957	368112.8942	342480.9355	222790.9412	394057.2794	200846.2614	281696.4493	134265.616	176661.8121
RRP7A	ribosomal RNA-processing protein 7 homolog A [Homo sapiens]	533392.7608	452682.3211	581451.4547	500565.5302	589402.8695	551401.787	504425.735	499555.7384	417847.6154
RRP8	ribosomal RNA-processing protein 8 [Homo sapiens]	470194.8894	411790.9265	559795.6759	435196.0011	640500.8	480876.8496	425452.5117	212363.2481	477149.1334
RRP9	U3 small nucleolar RNA-interacting protein 2 [Homo sapiens]	930390.0875	973660.5195	1215626.398	952551.2775	1269622.685	1000252.516	969190.3925	757926.509	972556.114
RRS1	ribosome biogenesis regulatory protein homolog [Homo sapiens]	593570.549	822355.8205	904075.9495	931915.6081	859205.2048	884745.3081	877156.4405	607462.2914	878763.5286
RSBN1	round spermatid basic protein 1 [Homo sapiens]	34268.05413	30133.95282	14666.69738	50669.16126	0	44406.5801	23047.62429	42705.76855	27831.54128
RSF1	remodeling and spacing factor 1 [Homo sapiens]	100394.0941	127211.5365	163168.2052	91267.80114	136808.55	117137.9894	106689.5532	76330.03118	147811.0908
RSL1D1	ribosomal L1 domain-containing protein 1 [Homo sapiens]	3005319.81	3078299.205	3951177.631	3550998.023	4017796.624	3628328.177	3279924.117	2917087.067	3484814.983
RSL24D1	probable ribosome biogenesis protein RLP24 [Homo sapiens]	0	3140576.024	2789589.389	2732869.137	2957387	0	3428002.253	2541626.066	3449754.116
RSRC1	serine/Arginine-related protein 53 isoform 1 [Homo sapiens]	45822.69966	193018.8156	66508.77083	84142.266	128946.6488	159523.9319	78486.88089	151926.8453	129334.7246
RSRC2	arginine/serine-rich coiled-coil protein 2 [Homo sapiens]	293079.5121	426805.8389	433788.0106	320950.2274	461307.1788	327755.5911	229217.0514	303339.9952	383108.1437
RSU1	ras suppressor protein 1 isoform 1 [Homo sapiens]	1745306.743	2161637.415	2350516.289	2742081.082	2638628.311	2683162.665	2402979.676	1971799.978	2216302.629
RTCA	RNA 3'-terminal phosphate cyclase isoform a [Homo sapiens]	213513.9448	450672.8135	529790.5869	460032.856	453591.3006	473872.1053	460083.6067	315186.4533	405834.9001
RTCB	tRNA-splicing ligase RtcB homolog [Homo sapiens]	2790178.722	2880877.399	3744302.885	2864856.269	4084206.979	2969171.926	2826364.856	2523118.177	2842448.289
RTF1	RNA polymerase-associated protein RTF1 homolog [Homo sapiens]	171960.0249	147040.7248	240872.8142	199289.6499	293923.7872	190181.5697	194653.8125	156547.1065	175148.264
RTFDC1	protein RTF2 homolog [Homo sapiens]	231882.8814	401059.4713	0	85870.97472	482103.6426	165754.0378	97270.93368	162759.5828	0
RTKN	rhotekin isoform a [Homo sapiens]	209608.9474	230508.7012	85325.53862	178146.5976	223091.3978	262411.6529	163428.9015	155174.4838	311926.0789
RTN3	reticulon-3 isoform a [Homo sapiens]	8703537.036	8172974.858	9781539.203	9674918.432	12343097.95	10524679.17	9889100.667	7725774.246	9421595.845
RTN4	reticulon-4 isoform B [Homo sapiens]	13112704.71	12281239.92	16907027.44	13486109.59	19044849.8	13228646.22	13915149.22	10727616.88	15620451.54
RTN4IP1	reticulon-4-interacting protein 1, mitochondrial precursor [Homo sapiens]	0	0	0	0	0	0	338521.922	183860.6087	0
RUFY1	RUN and FYVE domain-containing protein 1 isoform a [Homo sapiens]	132197.3018	87731.2525	107665.251	171420.5078	195896.6535	186203.1838	140904.8457	126735.692	167299.906
RUSC1	RUN and SH3 domain-containing protein 1 isoform d [Homo sapiens]	30308.48036	23626.69866	0	23543.01563	54574.13438	31795.92991	0	0	0
RUVBL1	ruvB-like 1 [Homo sapiens]	6471651.559	6451237.57	8391433.559	6835568.543	8196965.704	7040512.027	6906667.747	5925847.36	6532673.462
RUVBL2	ruvB-like 2 [Homo sapiens]	4976943.028	5333821.625	6819135.57	5765868.2	6737574.535	5289043.58	5145473.205	4965429.514	5419092.3
RWDD1	RWD domain-containing protein 1 isoform a [Homo sapiens]	1149643.725	1225138.288	1333210.793	1161161.621	1108601.68	1271990.619	1287200.503	1042508.612	1145625.793
RWDD2B	RWD domain-containing protein 2B [Homo sapiens]	0	0	183015.7836	145287.8469	147276.2778	71118.35417	0	0	0
RWDD4	RWD domain-containing protein 4 [Homo sapiens]	475320.9205	0	497740.3182	635600.7083	423210.3712	654748.2689	592846.3788	565387.3788	0
RXRB	retinoic acid receptor RXR-beta isoform 1 [Homo sapiens]	86190.0728	153464.2353	382587.9288	194802.1485	373047.2	133456.4061	199764.8217	148400.265	195908.6484
RYBP	RING1 and YY1-binding protein [Homo sapiens]	11301.07032	31978.32647	0	9879.05642	0	26344.48513	30487.04004	35044.01592	0
S100A10	protein S100-A10 [Homo sapiens]	52921630.85	61357525.49	46315822.66	59598427.98	60922424.63	64732903.27	75550717.93	59101153.29	83357345.32
S100A11	protein S100-A11 [Homo sapiens]	99907235.44	111833812	109445641.1	121906740.9	104464538	127498132.4	130970940.5	115755402.9	97987196.7
S100A13	protein S100-A13 [Homo sapiens]	6859128.29	7716582.94	6317252.762	7737701.493	3790698.688	7549340.226	8178003.588	6124772.576	9826814.405
S100A14	protein S100-A14 [Homo sapiens]	0	0	0	0	0	0	0	0	0
S100A16	protein S100-A16 [Homo sapiens]	14064914.27	10282253.55	15528538.27	11485963.38	10463869.74	12145945.04	10634220.31	10446127.41	10817920.05
S100A2	protein S100-A2 [Homo sapiens]	1837812.192	1418763.871	2792568.457	1679713.843	2487655.971	2259041.318	1780968.636	1421165.614	1501821.3
S100A4	protein S100-A4 [Homo sapiens]	86903465.4	104135766.7	103114094	98227150.36	106145386	112139024	106718046.3	92053723.26	117667830.4
S100A6	protein S100-A6 [Homo sapiens]	4839198.96	4245804.1	18481461.87	9344488.853	17052743.99	15007727.42	7229875.575	15560304.86	4636347.72
S100A9	protein S100-A9 [Homo sapiens]	213370.8138	0	78490.89628	0	433178.8864	250478.9043	0	0	0
S100P	protein S100-P [Homo sapiens]	21332355.81	24281658.47	23133174.75	25243576.8	27995964.02	24779244.38	24403543.05	19895011.49	20918542.87
SAAL1	protein SAAL1 [Homo sapiens]	321484.4079	429635.491	555813.1701	429288.588	469798.0032	458960.8853	373634.5772	399087.5586	384897.7803
SAC3D1	SAC3 domain-containing protein 1 [Homo sapiens]	88187.31861	143102.0692	160362.4374	148278.589	157676.1819	209965.0399	118569.6076	187932.4028	180057.0599

SACM1L	phosphatidylinositide phosphatase SAC1 [Homo sapiens]	659637.6808	791408.4667	910978.0399	832293.0479	914052.4188	833174.9254	891269.4685	632760.6991	874997.4197
SAE1	SUMO-activating enzyme subunit 1 isoform a [Homo sapiens]	2853700.734	3294909.974	3379262.724	3130128.745	3619068.167	3518051.104	3236073.715	2974231.549	3354651.918
SAFB	scaffold attachment factor B1 isoform 1 [Homo sapiens]	408739.3391	499981.373	643568.6752	463667.4424	680638.84	499889.7741	329184.6611	372727.7569	472347.3468
SAFB2	scaffold attachment factor B2 [Homo sapiens]	268323.6791	245194.4511	365806.7325	254855.5989	404221.2338	245259.2707	209577.8678	166508.5282	250963.7717
SAMD1	atherin [Homo sapiens]	9443.241183	5833.763614	54274.26733	9899.083693	49377.56931	62473.18936	0	13577.57024	41698.0755
SAMD4B	protein Smaug homolog 2 [Homo sapiens]	0	0	146643.5518	55190.93256	235688.5677	63483.36456	32742.42253	157645.4235	240631.4291
SAMD9	sterile alpha motif domain-containing protein 9 [Homo sapiens]	63679.68704	51485.05328	84357.75863	342016.807	99209.53948	361964.1796	46196.65219	41985.95327	67241.92083
SAMHD1	SAM domain and HD domain-containing protein 1 [Homo sapiens]	92765.16942	119118.3383	112432.6703	155847.1837	165242.7646	153305.9792	132230.2501	101891.7111	108643.1339
SAMM50	sorting and assembly machinery component 50 homolog [Homo sapiens]	560492.9144	845527.2056	990411.0328	858108.8122	1118848.966	844990.2394	884939.5639	487432.1653	919627.1239
SAP130	histone deacetylase complex subunit SAP130 isoform a [Homo sapiens]	68751.58219	121133.4709	113624.3737	63353.95935	0	72859.79281	37343.49565	58469.20421	322673.6438
SAP18	histone deacetylase complex subunit SAP18 [Homo sapiens]	4727667.889	5134167.501	3714192.663	5095963.933	5121447.641	5772346.596	3452313.357	3904306.564	4632539.005
SAP30BP	SAP30-binding protein [Homo sapiens]	395142.5569	557331.8385	842643.2177	507453.9581	415149.8826	622021.6734	442887.0181	327680.3306	878371.5202
SAPCD2	suppressor APC domain-containing protein 2 [Homo sapiens]	0	23986.06645	30861.5871	22249.03951	28286.96229	20418.2579	18481.16097	13326.34734	21683.63174
SAR1A	GTP-binding protein SAR1a [Homo sapiens]	4885187.034	7250679.818	9227631.573	8122113.216	8435554.504	7977845.895	7800035.544	6629876.103	8516060.922
SAR1B	GTP-binding protein SAR1b [Homo sapiens]	3039351.936	3334257.099	3517409.684	3363867.292	3201015.975	3266395.992	3364285.551	2960175.821	2773439.992
SARNP	SAP domain-containing ribonucleoprotein [Homo sapiens]	4784280.868	6522810.485	5864584.777	5700830.074	6116524.441	5666109.371	5291436.165	5307378.691	5651925.952
SARS	serine--tRNA ligase, cytoplasmic [Homo sapiens]	2085438.104	1986349.654	2896397.85	2422783.636	2711574.662	2421843.31	2524249.131	2109199.813	2623509.038
SARS2	serine--tRNA ligase, mitochondrial isoform b precursor [Homo sapiens]	374156.0094	485932.5862	524592.4078	449080.5282	589541.5341	499492.2141	468693.433	354243.9216	416780.6099
SART1	U4/U6.U5 tri-snRNP-associated protein 1 [Homo sapiens]	414982.6989	468148.8979	673285.3929	499884.8908	820858.3216	420807.4147	460102.2234	389254.0968	358691.1721
SART3	squamous cell carcinoma antigen recognized by T-cells 3 [Homo sapiens]	497895.9244	293362.6806	269151.1055	300647.944	280116.8529	293426.0321	248591.3847	207556.2428	312201.1392
SASS6	spindle assembly abnormal protein 6 homolog [Homo sapiens]	0	0	32644.13946	68551.19303	35864.4035	51125.16541	0	0	0
SBDS	ribosome maturation protein SBDS [Homo sapiens]	3131405.974	3671764.847	3389140.907	3700796.557	3644289.991	3044873.243	3651125.254	3115468.844	3864076.74
SBF1	myotubularin-related protein 5 [Homo sapiens]	11696.05973	9871.210166	14469.32944	10435.23373	19487.03143	12683.9913	8894.424356	7461.615761	12130.97754
SBNO1	protein strawberry notch homolog 1 isoform 1 [Homo sapiens]	59618.28196	165274.2292	61976.0792	69432.76883	73007.3177	96447.98537	68996.97567	74147.23811	58369.7395
SBSN	suprabasin isoform 1 precursor [Homo sapiens]	122795.3394	91266.26017	103082.4819	51397.37827	220642.197	164192.802	110127.6384	0	110375.8543
SCAF1	splicing factor, arginine/serine-rich 19 [Homo sapiens]	23981.47073	28137.17679	26394.88489	24041.50318	49668.37217	37630.49582	34877.84775	28589.51138	30360.20164
SCAF11	protein SCAF11 [Homo sapiens]	61447.0556	48934.48252	147157.6386	152831.5211	138403.0251	80814.37237	94353.51907	111179.5465	106318.3844
SCAF4	splicing factor, arginine/serine-rich 15 isoform 1 [Homo sapiens]	0	0	0	0	0	0	0	27875.32471	16310.94514
SCAF8	protein SCAF8 [Homo sapiens]	0	11144.67732	70733.95904	48011.51852	21685.71127	35456.56405	7240.575348	0	47982.46358
SCAMP1	secretory carrier-associated membrane protein 1 [Homo sapiens]	999990.8539	936857.1134	1251576.5	935667.5378	1136049.527	1036552.921	869217.6512	897828.8209	978189.6778
SCAMP2	secretory carrier-associated membrane protein 2 [Homo sapiens]	1718798.037	2101374.633	1577908.434	2267107.194	1685095.424	2368928.203	1511885.321	631763.0634	2100320.012
SCAMP3	secretory carrier-associated membrane protein 3 isoform 1 [Homo sapiens]	3037207.754	3763480.597	3031334.096	3604659.935	3252918.355	3331201.142	3587929.244	3170080.318	4485051.577
SCAMP4	secretory carrier-associated membrane protein 4 [Homo sapiens]	1695970.74	1599268.56	1440026.08	1341930.8	1826620.02	1498987.945	1684269.215	1341188.57	2057536.54
SCARB1	scavenger receptor class B member 1 isoform 1 [Homo sapiens]	1179569.466	1388397.415	1620318.433	1475412.425	1661747.962	1460451.742	1468675.05	1222701.91	1533338.966
SCARB2	lysosome membrane protein 2 isoform 1 precursor [Homo sapiens]	678677.3026	449421.5558	1069740.24	498158.3952	968486.3728	552765.013	565021.8423	647293.9501	855427.945
SCCPDH	saccharopine dehydrogenase-like oxidoreductase [Homo sapiens]	563633.1428	777595.7774	564965.2293	848387.1239	603026.514	919196.6239	1165904.551	729432.2058	592629.5511
SCD	acyl-CoA desaturase [Homo sapiens]	666294.9011	1838893.511	1796803.719	2325926.277	2702026.466	2306473.305	2191097.813	1372320.57	1584543.095
SCFD1	sec1 family domain-containing protein 1 isoform a [Homo sapiens]	939021.4108	655026.15	1190942.282	897697.1617	1101030.647	879566.5667	1026616.023	760021.7758	594072.3058
SCFD2	sec1 family domain-containing protein 2 [Homo sapiens]	78510.27674	76946.16047	60021.87735	52469.37674	85032.73488	0	25702.26076	52214.8066	75573.45526
SCML2	sex comb on midleg-like protein 2 [Homo sapiens]	81973.9458	19406.44936	128273.3068	66306.84056	71549.01444	58792.1486	39002.39731	60834.15846	0
SCO1	protein SCO1 homolog, mitochondrial [Homo sapiens]	381504.5485	234030.3579	355630.0797	457591.6779	350077.7165	221406.6525	379120.1382	0	255209.0848
SCO2	protein SCO2 homolog, mitochondrial precursor [Homo sapiens]	319746.4005	522660.3495	370086.4115	560716.2767	585797.4284	514138.5231	428839.2929	452575.2427	674327.7343
SCOC	short coiled-coil protein isoform 1 [Homo sapiens]	1905417.574	2128194.765	2918492.035	2197169.311	2529332.818	1538575.568	1244077.111	1817718.015	1835762.058
SCP2	non-specific lipid-transfer protein isoform 1 proprotein [Homo sapiens]	1273242.482	2028577.672	2715289.685	2291802.003	3079471.779	2201095.147	2183855.576	1819896.747	2603183.245
SCPEP1	retinoid-inducible serine carboxypeptidase precursor [Homo sapiens]	280549.3167	416840.5434	379251.8881	290071.2754	299278.1701	249021.8946	266000.9901	128074.2963	216730.6138
SCRIB	protein scribble homolog isoform a [Homo sapiens]	64665.91546	96441.50014	113642.5664	81150.00386	156744.6089	107880.9512	77326.23129	82758.69923	118775.3635
SCRN2	secernin-2 isoform 1 [Homo sapiens]	125919.9823	368060.3125	315488.3523	50410.91331	246196.8535	73259.58669	391942.2117	404165.4335	231673.1099
SCYL1	N-terminal kinase-like protein isoform A [Homo sapiens]	367142.86	358018.2313	501640.9857	287252.2797	474686.8193	340284.935	354990.946	344614.981	434626.7557
SCYL2	SCY1-like protein 2 [Homo sapiens]	155995.4999	118384.2485	131765.0245	147570.7487	198723.8017	131326.0457	87016.63072	100630.8589	99296.24922
SDAD1	protein SDA1 homolog [Homo sapiens]	143222.8177	137282.4733	215618.9283	153835.7608	217621.749	162960.0001	144663.4196	266660.6815	88202.06369
SDC1	syndecan-1 precursor [Homo sapiens]	852086.0114	1146100.899	1040440.015	0	1024419.879	0	0	0	0
SDCBP	syntenin-1 isoform 1 [Homo sapiens]	2495607.699	2903569.066	3471009.594	3079014.356	3625886.788	3183286.581	3348711.128	2896306.343	2435426.432
SDCCAG3	serologically defined colon cancer antigen 3 isoform 1 [Homo sapiens]	0	0	0	0	0	0	47525.02961	44436.23602	48705.04112
SDE2	protein SDE2 homolog [Homo sapiens]	101907.9137	0	83925.78356	118551.3988	105118.0326	66957.95	551745.6966	260004.891	57425.09361
SDF2L1	stromal cell-derived factor 2-like protein 1 precursor [Homo sapiens]	3148484.716	3941753.609	4266879.082	3958081.134	4666092.427	2925596.176	4146092.616	2957971.158	4135442.325
SDF4	45 kDa calcium-binding protein isoform 2 precursor [Homo sapiens]	867466.2333	1100589.718	929909.4607	1101046.591	848741.343	1018304.961	965733.9407	878721.3593	1070049.23
SDHA	succinate dehydrogenase [ubiquinone] flavoprotein subunit, mitochondrial [I	3776545.789	3870858.684	4891740.215	4329656.541	5083209.496	4334648.886	3732419.448	3199221.301	4160379.915
SDHAF1	succinate dehydrogenase assembly factor 1, mitochondrial [Homo sapiens]	32891.3323	36681.62917	39857.23958	43645.14375	63344.42917	31862.68855	0	0	0
SDHAF2	succinate dehydrogenase assembly factor 2, mitochondrial [Homo sapiens]	2236928.545	2194531.828	2945147.865	1760575.797	2487958.312	1723531.328	1725090.241	1008851.869	1286870.429
SDHB	succinate dehydrogenase [ubiquinone] iron-sulfur subunit, mitochondrial pr	2005923.997	3650282.949	4441142.477	2616415.739	4490029.801	3991428.628	3763023.661	3563741.391	2789492.292
SDHC	succinate dehydrogenase cytochrome b560 subunit, mitochondrial isoform 1 p	758337.9279	744205.6058	3748591.762	3850330.073	7965235.929	469390.7237	4136236.413	3525263.969	8327824.169
SDHD	succinate dehydrogenase [ubiquinone] cytochrome b small subunit, mitochond	1736022.9	2336973.1	1643647.416	2421374.697	1961316.666	2300281.556	2282578.766	1865658.019	2350369.475
SEC11A	signal peptidase complex catalytic subunit SEC11A isoform 2 [Homo sapiens]	0	0	0	0	0	0	4199014.732	0	4194341.962
SEC13	protein SEC13 homolog isoform 1 [Homo sapiens]	9126101.588	10004690	11559112.66	9572965.68	21411310.39	10416906.4	9535035.053	7894553.349	9859035.531
SEC14L1	SEC14-like protein 1 isoform b [Homo sapiens]	22472.52369	8906.478146	0	11823.85365	9989.487324	16446.92416	11874.59786	12140.13568	0
SEC16A	protein transport protein Sec16A isoform 1 [Homo sapiens]	110454.3777	134886.7899	157668.8657	112410.8299	140687.933	116866.5016	107737.9258	150319.4206	147561.927
SEC22A	vesicle-trafficking protein SEC22a [Homo sapiens]	156599.4553	154811.3796	184574.4158	145362.3	0	0	0	0	0
SEC22B	vesicle-trafficking protein SEC22b precursor [Homo sapiens]	7483986.634	8869307.931	9318501.444	8330037.556	10344025.09	9162246.912	8678596.591	6993854.993	9529087.136
SEC23A	protein transport protein Sec23A [Homo sapiens]	1186096.341	1316866.796	1527374.699	1280216.517	1457947.546	1365963.74	1253375.644	1120756.983	910253.3619
SEC23B	protein transport protein Sec23B isoform 1 [Homo sapiens]	485043.8852	579624.8176	635388.1066	581994.3016	700940.3266	569541.9086	530634.966	421257.9555	555957.9094
SEC23IP	SEC23-interacting protein [Homo sapiens]	269443.9251	291407.5982	913818.6029	223349.6623	986633.2485	240183.7068	256244.2626	259925.5749	336564.3117
SEC24A	protein transport protein Sec24A isoform 1 [Homo sapiens]	296260.2499	395215.4974	352254.281	412554.703	387651.8222	416001.997	341302.0808	310200.5059	322518.0454
SEC24B	protein transport protein Sec24B isoform a [Homo sapiens]	54788.5927	91698.8691	134209.8427	126231.3039	143933.3756	96694.68713	67869.61446	79124.68273	99931.71671
SEC24C	protein transport protein Sec24C [Homo sapiens]	775881.7117	761274.0308	1034943.426	773271.3987	1062996.79	740324.4264	728358.0963	647970.7462	844757.3368
SEC24D	protein transport protein Sec24D [Homo sapiens]	138597.5523	80674.19652	259992.4124	139506.0924	246656.1478	227943.7358	108291.7012	112216.4037	257988.3224
SEC31A	protein transport protein Sec31A isoform 1 [Homo sapiens]	678064.6323	842153.2078	907281.8308	748058.3657	1018391.188	790924.3931	791679.6167	631385.4159	801483.8781
SEC61A1	protein transport protein Sec61 subunit alpha isoform 1 [Homo sapiens]	1695669.326	1617724.553	2166309.457	1303959.635	1515159.129	1444291.889	1612059.34	2855496.061	1432349.91
SEC61B	protein transport protein Sec61 subunit beta [Homo sapiens]	25378901.41	23694332.69	34386236.39	26799185.37	38402040.32	27231298.37	26583659.22	23893694.62	29935830.15
SEC61G	protein transport protein Sec61 subunit gamma [Homo sapiens]	23857535.66	24629107.75	39927899.1	26244955.24	28303795.36	24877617.64	25623036.6	21277868.07	26464673.46
SEC63	translocation protein SEC63 homolog [Homo sapiens]	234561.8264	381326.9065	372223.8577	317393.4161	428954.4397	362229.2456	423327.8632	264085.0309	431742.1479
SEH1L	nucleoporin SEH1 isoform 1 [Homo sapiens]	775111.6795	1204362.909	1211617.149	1071992.446	1221206.608	1193151.021	1146111.343	839433.3479	1187941.531
SEL1L	protein sel-1 homolog 1 isoform 1 precursor [Homo sapiens]	168298.3452	194123.0214	265545.6359	164344.1901	153885.4224	187762.6048	233624.1607	133104.8274	222479.1605
SELENBP1	selenium-binding protein 1 isoform 3 [Homo sapiens]	834971.0398	958208.0833	1160804.593	925572.8817	1137367.639	1044238.551	841998.8081	775694.6414	1142777.565
SELO	selenoprotein O [Homo sapiens]	143176.0722	165245.8084	195122.3967	157735.8304	189289.7858	158879.5518	159085.5837	81370.69185	132846.9416
SELRC1	sel1 repeat-containing protein 1 [Homo sapiens]	419635.101	540220.5878	411743.9349	429884.1439	352859.3209	360822.351	455479.7172	478683.8182	314138.668
SELT	selenoprotein T precursor [Homo sapiens]	537616.902	1126205.91	1109140.19	1391519.758	682180.6743	914759.6457	1144750.237	1842569.923	1200340.504
SENP1	sentrin-specific protease 1 [Homo sapiens]	0	0	0	0	0	0	0	0	0
SENP3	sentrin-specific protease 3 [Homo sapiens]	209116.2979	357565.3888	492382.3142	315023.0538	382127.823	383053.4723	342435.9318	222838.0085	0
SENP6	sentrin-specific protease 6 isoform 1 [Homo sapiens]	0	0	0	0	0	0	0	7036.221405	11147.04466
SEPHS1	selenide, water dikinase 1 isoform 1 [Homo sapiens]	1434578.871	1663875.789	1861394.536	1587506.561	1940710.586	1530644.728	1598809.7	1311075.394	1621331.113
SERBP1	plasminogen activator inhibitor 1 RNA-binding protein isoform 3 [Homo sapie	690684.6127	782419.5877	697681.7224	554338.3846	993245.0662	656731.8397	664169.3248	608500.7662	785241.7613
SERBP1	plasminogen activator inhibitor 1 RNA-binding protein isoform 1 [Homo sapie	690684.6127	782419.5877	697681.7224	554338.3846	993245.0662	656731.8397	664169.3248	608500.7662	785241.7613
SERPINB1	leukocyte elastase inhibitor [Homo sapiens]	4206649.763	4489731.057	5042214.854	4619950.76	5247373.181	4350528.098	3700057.696	3701504.102	3451434.783
SERPINB12	serpin B12 [Homo sapiens]	12856.96115	228068.4519	0	109946.8809	37067.30195	31361.80448	23582.322	589875.2568	34250.92335
SERPINB5	serpin B5 [Homo sapiens]	3052643.329	3725637.399	3719862.755	3218637.13	3687187.344	3381802.997	3093072.126	2816683.291	2767411.328
SERPINB6	serpin B6 isoform a [Homo sapiens]	2687676.884	4132950.583	3525544.25	4030425.438	5647003.52	1907439.892	1228062.433	1152884.704	2144116.129
SERPINB8	serpin B8 isoform a [Homo sapiens]	127772.1922	149769.9198	102196.3673	82882.2484	127136.8642	0	139640.7654	140630.892	215620
SERPINB9	serpin B9 [Homo sapiens]	273072.6769	381680.5971	279035.3173	321179.0149	282902.3676	332524.4356	309838.5007	235773.8887	389001.1521
SERPINH1	serpin H1 precursor [Homo sapiens]	10564729.69	10668595.93	13259333.67	10964251.34	13011404.59	12179033.2	11677414.86	10325066.87	11151446.71
SESTD1	SEC14 domain and spectrin repeat-containing protein 1 [Homo sapiens]	0	0	60949.87392	34840.37536	26346.55751	35222.67402	20059.83935	0	27044.66255
SET	protein SET isoform 1 [Homo sapiens]	2368357.551	2866705.205	4618459.116	2131503.319	4357736.364	3034181.425	3181325.006	2095905.143	2153213.37
SET	protein SET isoform 2 [Homo sapiens]	2368357.551	2866705.205	4618459.116	2131503.319	4357736.364	3034181.425	3181325.006	2095905.143	2153213.37
SETD2	histone-lysine N-methyltransferase SETD2 [Homo sapiens]	10574.45149	8206.682829	9978.986258	49727.81674	0	15317.16498	9652.975651	236090.824	0
SETD3	histone-lysine N-methyltransferase setd3 isoform a [Homo sapiens]	241080.5236	280691.7759	270999.2561	304869.4132	390521.2215	295920.5261	198695.3422	275277.4046	251898.9222
SETD7	histone-lysine N-methyltransferase SETD7 [Homo sapiens]	678473.3022	866381.2561	1333710.783	261795.9719	1282407.354	1123444.195	737094.3706	672995.4233	668582.6719
SETDB1	histone-lysine N-methyltransferase SETDB1 isoform 1 [Homo sapiens]	47242.4184	2906.226654	27177.86442	0	149716.9964	0	0	0	0
SETMAR	histone-lysine N-methyltransferase SETMAR isoform 1 [Homo sapiens]	0	45158.21643	34832.99117	32968.79284	59230.93597	0	33377.01458	0	43713.44876
SF1	splicing factor 1 isoform 1 [Homo sapiens]	66485.406	0	89805.41133	111619.3456	89394.69703	116992.8235	777246.2541	81392.5	1119325.221
SF1	splicing factor 1 isoform 4 [Homo sapiens]	66485.406	0	89805.41133	111619.3456	89394.69703	116992.8235	777246.2541	81392.5	1119325.221
SF1	splicing factor 1 isoform 6 [Homo sapiens]	66485.406	0	89805.41133	111619.3456	89394.69703	116992.8235	777246.2541	81392.5	1119325.221
SF3A1	splicing factor 3A subunit 1 isoform 1 [Homo sapiens]	2043731.62	2427691.018	2578223.08	2240973.182	2965856.378	2200398.088	2386193.761	2027800.931	2420776.769
SF3A2	splicing factor 3A subunit 2 [Homo sapiens]	2729327.98	2859644.12	2013714.26	2361462.53	3828919.393	3048729.45	2933787.029	1916289.2	3417169.569
SF3A3	splicing factor 3A subunit 3 [Homo sapiens]	2243351.075	2279606.991	2739363.484	2304134.877	2585305.721	2396813.637	2271415.533	2016753.619	2402288.3
SF3B1	splicing factor 3B subunit 1 isoform 1 [Homo sapiens]	1116111.223	1202601.116	1317015.44	1291499.499	1453063.847	1316555.707	1292066.459	1086853.811	1395878.665
SF3B14	pre-mRNA branch site protein p14 [Homo sapiens]	5517694.811	6420069.786	7510889.251	6219454.889	8083846.254	6844915.419	6295324.783	5123574.779	3932021.13
SF3B2	splicing factor 3B subunit 2 [Homo sapiens]	2215388.266	2123074.278	3074853.219	2168357.502	2931013.939	2121978.004	1800823.174	1426458.762	1542154.224
SF3B3	splicing factor 3B subunit 3 [Homo sapiens]	2036596.236	2088375.194	2681236.098	2254616.048	2400077.488	2364256.754	2140849.167	1856282.13	2069744.78
SF3B4	splicing factor 3B subunit 4 [Homo sapiens]	8165824.428	7474094.984	9798848.162	7614493.573	9552747.79	8303778.005	6982146.001	5295886.031	7979835.778
SF3B5	splicing factor 3B subunit 5 [Homo sapiens]	12707447.49	12516913.88	16289595.81	13841352.25	18187089.34	13243053.43	11399051.05	8036856.929	14037222.75
SFN	14-3-3 protein sigma [Homo sapiens]	14565256.84	14098911.92	19390843.94	14642997.1	15893273.14	16354392.81	14784648.65	12558895.46	15482486.85

SFPQ	splicing factor, proline- and glutamine-rich [Homo sapiens]	7429827.824	7716381.718	8312908.218	7940970.764	8475501.581	7930628.229	7170409.433	5377582.75	8006376.954
SFSWAP	splicing factor, suppressor of white-apricot homolog isoform 1 [Homo sapien	16317.80419	48841.71953	55687.66256	55025.41246	67238.98953	50244.80241	42603.5197	79115.98754	0
SFT2D2	vesicle transport protein SFT2B [Homo sapiens]	0	919897.4792	414171.2292	976940.0625	999647.6667	0	929127.3204	761055.8438	455374.3021
SFXN1	sideroflexin-1 [Homo sapiens]	3757473.073	5054995.205	4338491.874	4290655.056	4801503.175	4374119.178	4616343.563	3729108.042	4606700.46
SFXN2	sideroflexin-2 [Homo sapiens]	0	0	0	0	0	0	48282.15092	0	49613.57724
SFXN3	sideroflexin-3 [Homo sapiens]	2050285.309	3037467.466	2290937.858	2049244.931	2297026.046	2193331.981	2226570.56	1887817.63	1894640.262
SFXN4	sideroflexin-4 [Homo sapiens]	0	0	0	0	0	0	100899.4746	0	252279.2775
SGPL1	sphingosine-1-phosphate lyase 1 [Homo sapiens]	173533.1937	199376.2511	321347.9615	214943.933	359565.5688	154960.1328	160477.6211	94339.31308	236081.0804
SGPP1	sphingosine-1-phosphate phosphatase 1 [Homo sapiens]	118230.6922	281318.0238	159374.1078	157033.6238	8470.954291	279669.1982	241071.6975	71624.68622	346260.9959
SGSH	N-sulphoglucosamine sulphohydrolase precursor [Homo sapiens]	182938.7386	161008.9722	253687.8411	169924.9014	217249.6556	178375.6953	196401.1148	146714.079	116936.5956
SGTA	small glutamine-rich tetratricopeptide repeat-containing protein alpha [Hor	2850788.497	3113474.916	3923986.588	3550219.435	4189580.363	3288808.816	3278066.642	2824840.483	3380295.666
SH2D4A	SH2 domain-containing protein 4A isoform a [Homo sapiens]	212093.4041	194696.018	176405.9458	253089.7323	248176.399	270255.3214	219120.133	225405.0962	299374.7749
SH3BGRL	SH3 domain-binding glutamic acid-rich-like protein [Homo sapiens]	482686.3265	3053520.315	3449671.9	1873714.944	787889.1563	3056543.246	2591913.479	2211646.373	1997914.248
SH3BGRL3	SH3 domain-binding glutamic acid-rich-like protein 3 [Homo sapiens]	8805584.95	9480599.268	10842699.46	10316503.39	12200933.49	10392898.37	9477069.535	8219266.309	10192416.94
SH3GL1	endophilin-A2 isoform 1 [Homo sapiens]	1750984.799	1914647.093	2426083.117	2051414.203	2467933.107	2076853.198	1978271.281	1725330.283	2128621.203
SH3GLB1	endophilin-B1 isoform 2 [Homo sapiens]	553925.3183	640607.8686	747623.6736	642300.7575	811017.2201	664862.6331	619174.011	402846.6862	654127.2165
SH3GLB2	endophilin-B2 [Homo sapiens]	273929.6711	173570.4309	395420.2536	1147719.335	395374.8559	441088.7291	835378.4632	367865.9326	380816.9059
SH3KBP1	SH3 domain-containing kinase-binding protein 1 isoform a [Homo sapiens]	312405.7809	486297.3933	489174.1077	462014.3973	540973.1586	489447.3495	459256.7973	316170.5264	483600.299
SHC1	SHC-transforming protein 1 isoform 3 [Homo sapiens]	425098.4816	485631.7123	518209.0959	333332.8922	491830.1904	479789.6693	449372.2494	404029.2183	387188.2774
SHCBP1	SHC SH2 domain-binding protein 1 [Homo sapiens]	338427.95	205448.8273	195161.2008	255004.925	187989.575	398076.2727	151520.4023	277268.6549	173480.0901
SHKBP1	SH3KBP1-binding protein 1 [Homo sapiens]	33216.45023	63160.54638	178114.3337	35640.33371	93420.03977	51178.35068	41815.32325	25254.49661	0
SHMT1	serine hydroxymethyltransferase, cytosolic isoform 1 [Homo sapiens]	908135.2311	1087839.45	1152034.673	955489.6667	1205174.833	1044292.011	936183.6459	848099.7388	946898.5694
SHMT2	serine hydroxymethyltransferase, mitochondrial isoform 1 precursor [Homo sa	3646548.015	4696223.732	4545831.835	4232499.454	4937482.249	4685089.491	4975067.854	4147575.458	5218538.897
SHOC2	leucine-rich repeat protein SHOC-2 isoform 1 [Homo sapiens]	0	0	0	0	0	0	65360.45249	57314.40138	31714.57972
SHPK	sedoheptulokinase [Homo sapiens]	154695.2126	139473.1772	238192.9213	155394.7441	239076.8661	178205.6063	0	0	0
SIDT2	SID1 transmembrane family member 2 precursor [Homo sapiens]	0	0	0	0	0	0	0	0	0
SIGMAR1	sigma non-opioid intracellular receptor 1 isoform 1 [Homo sapiens]	2916113.851	2450125.647	3177771.796	2821547.265	2736929.969	3028861.749	3821609.48	2252564.842	2011196.684
SIL1	nucleotide exchange factor SIL1 precursor [Homo sapiens]	406678.8144	553930.4933	449360.5751	399121.6123	564577.8912	408518.7123	488894.2934	303608.3199	495341.8084
SIN3A	paired amphipathic helix protein Sin3a [Homo sapiens]	146734.0765	177081.0552	200540.92	144222.1294	230610.5717	162078.7404	131914.7824	129973.3511	336196.892
SIPA1	signal-induced proliferation-associated protein 1 [Homo sapiens]	57215.8212	35519.84511	77341.60964	49845.41825	76646.05683	28126.25803	24836.5	19948.85425	33587.28689
SIPA1L1	signal-induced proliferation-associated 1-like protein 1 [Homo sapiens]	5427.605312	5940.087014	6045.947323	7451.73783	10099.16533	5783.634302	0	0	0
SIPA1L3	signal-induced proliferation-associated 1-like protein 3 [Homo sapiens]	0	0	0	0	0	0	0	0	0
SIRT1	NAD-dependent protein deacetylase sirtuin-1 isoform a [Homo sapiens]	90034.97486	34382.9756	56811.6602	53617.95155	41117.66327	58873.75527	47372.78273	47866.60698	57400.85714
SIRT2	NAD-dependent protein deacetylase sirtuin-2 isoform 1 [Homo sapiens]	0	0	0	0	0	0	232087.7559	28534.21622	0
SKA1	spindle and kinetochore-associated protein 1 [Homo sapiens]	124219.9284	248470.6585	123592.1946	170490.6646	116225.0488	184979.2581	140131.9898	87314.72057	0
SKA3	spindle and kinetochore-associated protein 3 isoform 1 [Homo sapiens]	75422.58333	66695.40586	115784.7222	137624.6165	146129.0108	143089.3488	64841.71296	0	68577.40432
SKIV2L	helicase SKI2W [Homo sapiens]	187316.3276	136324.683	204062.4442	208342.4029	210599.0524	217038.3499	167826.6903	166914.6342	146175.9035
SKIV2L2	superkiller viralicidic activity 2-like 2 [Homo sapiens]	472643.5736	623027.6902	886066.8736	701868.4287	715115.6294	719362.5702	608972.5353	462035.9984	692692.1781
SKP1	S-phase kinase-associated protein 1 isoform b [Homo sapiens]	8541162.197	12015576.21	10740410.44	11247794.53	11115942.27	13059443.88	10948722.38	9636900.851	12441537.75
SKP2	S-phase kinase-associated protein 2 isoform 1 [Homo sapiens]	220085.0054	314946.1313	373286.7479	415581.795	440760.504	440276.6334	326926.007	262074.2392	154849.8687
SLAIN2	SLAIN motif-containing protein 2 [Homo sapiens]	0	0	0	0	0	0	27850.17174	81826.89783	0
SLBP	histone RNA hairpin-binding protein [Homo sapiens]	263820.3239	150312.0129	1503901.15	207308.5215	154892.0734	213006.4815	141457.2922	327039.2161	158350.8665
SLC12A2	solute carrier family 12 member 2 isoform 1 [Homo sapiens]	46364.1383	31513.27476	39145.37367	34766.86375	60739.32979	32339.3059	48604.0758	31657.09343	41590.25077
SLC12A4	solute carrier family 12 member 4 isoform c [Homo sapiens]	0	37696.88655	17137.21818	53056.23209	20350.30815	0	0	0	0
SLC12A6	solute carrier family 12 member 6 isoform a [Homo sapiens]	0	0	0	0	0	0	38588.61264	0	23606.28365
SLC16A1	monocarboxylate transporter 1 [Homo sapiens]	2480794.773	2483472.697	2492356.226	2385317.349	4088907.06	2580847.493	2315531.834	1798481.028	2732278.68
SLC16A3	monocarboxylate transporter 4 [Homo sapiens]	2148943.258	2747681.369	3127573.037	2601687.914	3558818.649	2409466.216	2606539.081	2175550.873	2644795.556
SLC16A6	monocarboxylate transporter 7 [Homo sapiens]	235475.2414	95255.15386	237257.2103	135702.0873	269313.5253	154432.1328	91340.15952	98196.36124	0
SLC16A7	monocarboxylate transporter 2 [Homo sapiens]	0	0	0	0	0	0	0	59783.437	222285.856
SLC19A1	folate transporter 1 isoform 1 [Homo sapiens]	0	0	0	0	0	0	194507.5229	0	84722.43598
SLC1A3	excitatory amino acid transporter 1 isoform 1 [Homo sapiens]	355567.1569	406996.4783	619800.8287	470170.3593	648980.782	175111.8249	358079.389	451849.7316	422588.6502
SLC1A5	neutral amino acid transporter B(0) isoform 1 [Homo sapiens]	6087627.434	6559575.813	7714516.791	6901293.336	7411185.377	7216145.405	7335226.038	6655198.287	7039087.096
SLC20A1	sodium-dependent phosphate transporter 1 [Homo sapiens]	230902.6728	162613.5291	277052.1073	238994.2565	277147.6322	219878.91	178195.3672	0	135224.4596
SLC25A1	tricarboxylate transport protein, mitochondrial isoform b [Homo sapiens]	1900637.904	2304820.299	2615836.926	2135423.967	2679722.39	2249513.573	2346787.873	2124227.118	2782410.579
SLC25A10	mitochondrial dicarboxylate carrier isoform 1 [Homo sapiens]	1528195.294	1779749.691	2042974.893	1746145.691	2039492.607	1738479.92	1801152.982	1409258.064	1836797.362
SLC25A11	mitochondrial 2-oxoglutarate/malate carrier protein isoform 1 [Homo sapiens	3265235.222	3316701.483	4247609.306	3820481.009	4790249.684	3842270.58	3811055.226	2315475.132	3688568.73
SLC25A12	calcium-binding mitochondrial carrier protein Aralar1 [Homo sapiens]	595132.537	611447.8613	657407.0667	601288.8276	676431.8609	602041.2173	612460.0144	528709.6535	661771.4403
SLC25A13	calcium-binding mitochondrial carrier protein Aralar2 isoform 1 [Homo sapie	943713.9514	1050367.547	1144785.383	963237.3672	1184036.951	991330.5285	1022120.89	938067.6024	1132330.424
SLC25A15	mitochondrial ornithine transporter 1 [Homo sapiens]	289856.0058	525153.2356	566102.031	272783.0906	457281.4954	315034.3583	531249.1074	462694.8402	482086.0966
SLC25A17	peroxisomal membrane protein PMP34 [Homo sapiens]	103210.3586	197429.2149	125509.386	140020.2869	139294.4605	291682.2456	245211.5073	231523.751	179537.9357
SLC25A19	mitochondrial thiamine pyrophosphate carrier [Homo sapiens]	396747.2461	411394.186	571938.1008	380270.8778	581896.9104	394549.1266	381582.8114	328088.7652	339940.1987
SLC25A20	mitochondrial carnitine/acylcarnitine carrier protein [Homo sapiens]	1090697.634	450115.4449	665429.8772	992695.2652	632552.5685	804944.7613	765312.6525	490188.8757	520774.5915
SLC25A22	mitochondrial glutamate carrier 1 [Homo sapiens]	615395.031	370699.9952	665071.2671	615267.154	877927.5547	646701.99	642922.7115	465462.6448	844391.3974
SLC25A24	calcium-binding mitochondrial carrier protein SCaMC-1 isoform 1 [Homo sapie	1426670.783	1576914.435	1768127.745	1837613.019	1973005.944	1623742.979	2105760.331	1509734.938	1487258.827
SLC25A26	S-adenosylmethionine mitochondrial carrier protein isoform a [Homo sapiens]	283782.5016	0	395055.7561	343260.5061	393122.7256	352814.439	368517.6555	285563.9437	0
SLC25A3	phosphate carrier protein, mitochondrial isoform a precursor [Homo sapiens]	7075194.845	7682694.34	9556258.485	8007794.56	10548083.47	8165070.5	0	6558839.185	6225042.885
SLC25A3	phosphate carrier protein, mitochondrial isoform b precursor [Homo sapiens]	7075194.845	7682694.34	9556258.485	8007794.56	10548083.47	8165070.5	0	6558839.185	6225042.885
SLC25A32	mitochondrial folate transporter/carrier [Homo sapiens]	434667.775	338681.0326	448973.1378	314672.7	407673.4877	518832.8185	586683.058	529928.0783	264060.1261
SLC25A35	solute carrier family 25 member 35 [Homo sapiens]	0	0	0	0	0	0	141233.1279	132795.9338	179812.7703
SLC25A4	ADP/ATP translocase 1 [Homo sapiens]	1460266.629	1881733.079	2300056.097	1979585.296	2299677.19	2175680.02	2171966.535	1603011.814	2156416.832
SLC25A40	solute carrier family 25 member 40 [Homo sapiens]	0	0	0	0	0	0	0	3196347.447	4355762.38
SLC25A46	solute carrier family 25 member 46 [Homo sapiens]	162805.7829	113826.7884	187093.3904	60733.01782	220263.1503	88964.03509	80184.12939	84500.94216	104162.9661
SLC25A5	ADP/ATP translocase 2 [Homo sapiens]	18727509.44	25135376.47	24634838.7	24536795.72	27000547.88	25003894.75	25886721.43	20930845.05	28040942.77
SLC25A6	ADP/ATP translocase 3 [Homo sapiens]	8819647.81	9522028.467	9963753.577	9823053.474	10391588.36	9867599.723	10562083.08	9241607.971	11326448.51
SLC26A6	solute carrier family 26 member 6 isoform 1 [Homo sapiens]	111350.0744	50293.56733	134346.2489	73448.69638	118676.5866	292799.3348	134393.4962	151252.8597	41617.64261
SLC27A2	very long-chain acyl-CoA synthetase isoform 1 [Homo sapiens]	1275803.986	1151939.024	1532143.7	1211620.953	1590779.721	1254763.352	1295617.571	1073312.406	1189558.228
SLC27A4	long-chain fatty acid transport protein 4 [Homo sapiens]	100552.5749	115003.9907	207613.2656	169467.6881	201057.5319	154981.4909	216907.8428	140308.3847	172096.5073
SLC29A1	equilibrative nucleoside transporter 1 [Homo sapiens]	612208.4191	403687.3705	709552.3231	476506.1242	1183255.99	893740.151	377045.447	423371.9053	472431.2781
SLC2A1	solute carrier family 2, facilitated glucose transporter member 1 [Homo sap	2178755.588	2121688.778	2786636.472	2328365.511	3038380.707	2609258.647	2461906.904	1642472.353	2576360.166
SLC2A14	solute carrier family 2, facilitated glucose transporter member 14 [Homo sa	0	0	0	0	0	0	0	179088.5615	490652.4167
SLC2A3	solute carrier family 2, facilitated glucose transporter member 3 [Homo sap	0	252753.9139	0	119250.7899	302622.9241	151650.4517	0	0	0
SLC30A1	zinc transporter 1 [Homo sapiens]	1332918.436	1473564.1	1983289.43	1957286.47	2147711.205	1743171.114	1899212.532	1330757.14	2009070.088
SLC30A7	zinc transporter 7 [Homo sapiens]	1254431.292	387834.2912	1070673.999	584815.0301	1237847.596	813172.4865	652410.6984	797816.6812	4069067.367
SLC33A1	acetyl-coenzyme A transporter 1 [Homo sapiens]	257147.4702	413671.596	486657.7152	0	484001.5972	407766.4719	0	323670.7848	563236.3113
SLC35A2	UDP-galactose translocator isoform a [Homo sapiens]	147252.0593	105373.8387	0	336971.4637	348792.2219	0	272018.7679	262735.9643	416082.551
SLC35B1	solute carrier family 35 member B1 [Homo sapiens]	0	111957.5449	501109.1765	554971.5941	579848.2588	551358.7952	448218.2824	0	508596.8618
SLC35B2	adenosine 3'-phospho 5'-phosphosulfate transporter 1 precursor [Homo sapien	1004569.264	894252.2277	486120.8641	901050.7681	598935.1013	1131202.695	1002828.589	830621.4879	754148.4202
SLC35C1	GDP-fucose transporter 1 isoform a [Homo sapiens]	0	0	0	0	0	0	0	108826.0165	280895.6374
SLC35E1	solute carrier family 35 member E1 [Homo sapiens]	615012.7121	972256.9911	1532213.547	504742.8767	882727.7606	542080.0187	524403.5313	47635.21303	618452.596
SLC35F2	solute carrier family 35 member F2 [Homo sapiens]	448098.0941	690514.9015	459395.7765	625467.6294	151906.7471	681936.2	669879.9941	431425.4882	619778.9559
SLC35F6	solute carrier family 35 member F6 precursor [Homo sapiens]	1148931.41	1685529.325	697162.9629	1542866.078	812529.5269	1315197.59	1774765.506	1195664.937	1809393
SLC38A1	sodium-coupled neutral amino acid transporter 1 isoform 2 [Homo sapiens]	431713.5039	569235.9477	678204.7694	829483.7543	678822.0276	529165.6468	854585.1667	166727.1134	0
SLC38A2	sodium-coupled neutral amino acid transporter 2 [Homo sapiens]	920207.5205	1421186.972	1422938.43	1261911.992	1116770.716	1321943.834	1381071.831	1095047.603	1017653.542
SLC38A5	sodium-coupled neutral amino acid transporter 5 [Homo sapiens]	2491510.219	2683693.942	2769651.181	1626682.543	3013501.172	2614517.138	1446281.797	2112705.246	2409666.367
SLC39A1	zinc transporter ZIP1 isoform a [Homo sapiens]	0	0	0	0	0	0	493048.775	872325.774	0
SLC39A10	zinc transporter ZIP10 precursor [Homo sapiens]	105096.0191	174185.1102	181812.9809	167180.0833	208160.3559	158337.3157	126772.1536	91698.45657	98646.02436
SLC39A14	zinc transporter ZIP14 isoform b precursor [Homo sapiens]	919826.3804	1157198.717	703968.3465	1573382.63	1003675.198	1107054.027	1248074.177	1624963.582	1409719.375
SLC39A6	zinc transporter ZIP6 isoform 1 precursor [Homo sapiens]	0	0	0	0	0	0	74207.97077	0	121396.4409
SLC39A7	zinc transporter SLC39A7 precursor [Homo sapiens]	1107810.323	1057298.288	1900528.316	0	1569427.505	1248344.496	1047076.217	0	1285219.839
SLC39A8	zinc transporter ZIP8 isoform a precursor [Homo sapiens]	447102.0441	0	128080.4353	0	582918.5588	148908.7368	521288.5765	350111.0045	0
SLC3A2	4F2 cell-surface antigen heavy chain isoform c [Homo sapiens]	879543.991	906558.9717	1067547.403	922506.3986	1116309.676	1011814.961	915346.1667	887291.8479	595203.5923
SLC3A2	4F2 cell-surface antigen heavy chain isoform f [Homo sapiens]	879543.991	906558.9717	1067547.403	922506.3986	1116309.676	1011814.961	915346.1667	887291.8479	595203.5923
SLC41A3	solute carrier family 41 member 3 isoform 1 [Homo sapiens]	191564.3685	159104.0593	220539.4703	68972.86638	215095.6078	0	0	199088.0539	167753.0776
SLC43A3	solute carrier family 43 member 3 isoform 2 [Homo sapiens]	1356324.168	1861681.088	1094983.841	1957987.21	747984.0173	954331.305	1003657.493	822075.2321	995681.6782
SLC44A1	choline transporter-like protein 1 [Homo sapiens]	322275.182	159414.3407	421826.1095	318213.9225	305049.9963	292658.1863	334799.065	185694.1786	182499.8722
SLC46A1	proton-coupled folate transporter isoform 1 [Homo sapiens]	502048.8852	451613.8462	599819.25	462721.5385	630275.4904	484602.2633	0	0	0
SLC48A1	heme transporter HRG1 [Homo sapiens]	0	0	0	0	0	0	318225.4342	223119.7769	0
SLC4A1AP	kanadaptin [Homo sapiens]	184918.6906	164749.0831	255788.3541	170977.0768	214089.8001	174574.4744	159588.4981	156281.4604	206008.2676
SLC4A2	anion exchange protein 2 isoform 1 [Homo sapiens]	199823.2249	94781.60942	258135.5403	255186.36	262682.8324	202434.8322	243663.283	117322.235	244790.1504
SLC4A7	sodium bicarbonate cotransporter 3 isoform a [Homo sapiens]	123980.1114	0	191586.9237	135721.6158	189117.3384	133566.5241	134402.638	105162.7367	132232.0097
SLC5A3	sodium/myo-inositol cotransporter [Homo sapiens]	130830.0609	17039.96447	0	108764.415	132492.2893	0	0	113388.6142	20454.95305
SLC5A6	sodium-dependent multivitamin transporter [Homo sapiens]	290637.3	554198.7333	697650.0167	586368.7083	234666.6354	647627.3928	597503.325	173392.4083	0
SLC6A6	sodium- and chloride-dependent taurine transporter isoform a [Homo sapiens]	807350.4542	559223.018	879806.6536	442332.1601	1031896.327	913416.902	532082.3636	732255.2941	1152806.713
SLC6A9	sodium- and chloride-dependent glycine transporter 1 isoform 2 [Homo sapien	258690.6689	0	351188.7965	356352.924	391935.125	408455.3277	0	0	0
SLC7A1	high affinity cationic amino acid transporter 1 [Homo sapiens]	1052568.517	1181636.267	1643973.289	1034030.012	1704151.938	1784459.875	1482974.092	816428.7589	1522010.12
SLC7A11	cystine/glutamate transporter [Homo sapiens]	494374.5766	417249.6515	710413.6058	441384.9015	631211.0803	452023.6378	431823.396	192804.1314	580540.6861
SLC7A5	large neutral amino acids transporter small subunit 1 [Homo sapiens]	4744747.692	5733152.44	6530291.646	5474223.197	7306799.376	5930724.894	5309828.298	4547878.439	6936531.109
SLC7A6	Y+L amino acid transporter 2 [Homo sapiens]	235299.6513	440626.4547	136063.86	292104.0575	334789.2025	415211.8631	428635.6925	222780.7066	0
SLC9A3R1	Na(+)/H(+) exchange regulatory cofactor NHE-RF1 [Homo sapiens]	1137695.531	1012772.798	1198657.611	1353899.021	1256831.205	1424342.811	1424014.314	983123.625	1559514.537
SLC9A3R2	Na(+)/H(+) exchange regulatory cofactor NHE-RF2 isoform a [Homo sapiens]	385852.4748	487520.1933	673757.9781	319333.9346	658288.0013	506099.5146	487876.5487	267032.9891	624088.2203

SLCO4A1	solute carrier organic anion transporter family member 4A1 [Homo sapiens]	350950.4749	205926.9768	406800.6557	456797.8999	463980.5525	232317.4648	357648.9161	314445.5384	139086.838
SLFN5	schlafen family member 5 [Homo sapiens]	0	0	36241.22681	18471.93243	53618.44458	33786.54783	32315.24675	17513.98696	0
SLIRP	SRA stem-loop-interacting RNA-binding protein, mitochondrial isoform 1 prec	8758806.166	6506943.452	8162971.333	7477104.153	7944119.207	9384352.224	6690935.384	7788858.217	9721529.202
SLK	STE20-like serine/threonine-protein kinase [Homo sapiens]	115632.2367	127781.618	177831.9148	126936.4583	195674.5831	111552.1064	935977.6945	123622.294	141436.7098
SLTM	SAFB-like transcription modulator isoform a [Homo sapiens]	194777.7534	147829.3139	280454.8723	187333.4344	273494.4564	191300.8963	169542.9218	85399.66961	254708.0215
SLU7	pre-mRNA-splicing factor SLU7 [Homo sapiens]	26873.73548	92066.68278	74843.14965	72658.49746	64880.56602	75373.1694	35291.08408	433265.707	71081.18444
SMAD2	mothers against decapentaplegic homolog 2 isoform 1 [Homo sapiens]	544582.6987	666007.243	789438.4623	747654.2268	541141.0543	529550.8551	476294.1415	558463.3889	365136.0199
SMAD4	mothers against decapentaplegic homolog 4 [Homo sapiens]	126125.2973	277487.2116	332440.3303	142916.707	201881.5739	149057.5426	129720.2438	0	185158.4792
SMAP1	stromal membrane-associated protein 1 isoform A [Homo sapiens]	528244.2759	645735.2517	640656.179	626080.4755	1177362.536	581764.1673	607501.3122	500362.8942	688359.2177
SMAP2	stromal membrane-associated protein 2 isoform 1 [Homo sapiens]	130785.3242	142741.1309	174345.8555	277926.0532	216341.044	250608.3203	181729.9766	185886.8467	225722.9004
SMARCA1	probable global transcription activator SNF2L1 isoform a [Homo sapiens]	85486.24915	102991.8249	110021.3645	90574.93775	82150.58835	81244.2307	92859.6886	67106.91515	101018.4448
SMARCA1	probable global transcription activator SNF2L1 isoform b [Homo sapiens]	85486.24915	102991.8249	110021.3645	90574.93775	82150.58835	81244.2307	92859.6886	67106.91515	101018.4448
SMARCA4	transcription activator BRG1 isoform A [Homo sapiens]	222774.0141	241645.3887	210862.4685	244624.3996	317072.5966	175641.9558	188179.5405	193865.7162	307164.1651
SMARCA5	SWI/SNF-related matrix-associated actin-dependent regulator of chromatin su	356667.4193	567611.5199	382393.2133	370108.8976	385429.0229	335810.1012	390122.5538	281219.7843	395724.7916
SMARCAD1	SWI/SNF-related matrix-associated actin-dependent regulator of chromatin su	79450.01338	43176.89211	58681.07281	68459.25871	82147.65791	227483.4282	81277.45669	76711.96714	85210.06659
SMARCB1	SWI/SNF-related matrix-associated actin-dependent regulator of chromatin su	535053.815	624595.4781	803829.528	1389366.487	842479.1347	755867.0973	652418.7667	541321.2505	979142.84
SMARCC1	SWI/SNF complex subunit SMARCC1 [Homo sapiens]	335584.1026	351303.4162	501771.9947	623237.4866	599807.0656	446759.6816	374479.1296	503922.7792	402087.0912
SMARCC2	SWI/SNF complex subunit SMARCC2 isoform a [Homo sapiens]	238986.8884	284504.0145	307280.4687	283097.372	324079.294	283301.1451	275675.6395	254056.7757	293849.4771
SMARCD1	SWI/SNF-related matrix-associated actin-dependent regulator of chromatin su	116459.5532	139375.7398	152869.7832	115724.138	169869.573	85412.24833	111024.0913	126290.4506	117822.811
SMARCD2	SWI/SNF-related matrix-associated actin-dependent regulator of chromatin su	226256.1102	236919.5116	287697.7877	163753.6261	281657.0555	314179.363	279238.2647	160285.2245	255745.4843
SMARCE1	SWI/SNF-related matrix-associated actin-dependent regulator of chromatin su	359937.052	652328.047	1008909.707	557476.2565	755144.191	669687.5964	516261.3986	476134.2613	485377.4321
SMC1A	structural maintenance of chromosomes protein 1A [Homo sapiens]	242385.9623	255368.6889	261927.6202	299986.9909	302820.3648	238040.0479	267494.9776	192382.4777	257181.3774
SMC2	structural maintenance of chromosomes protein 2 [Homo sapiens]	398846.1161	404947.7287	436628.6576	411054.6384	446537.6424	352764.0802	363192.9033	356924.1912	380976.8977
SMC3	structural maintenance of chromosomes protein 3 [Homo sapiens]	268852.5267	476307.4006	243641.1325	363597.7823	308602.6275	318925.7805	228321.9468	298463.3992	230787.2017
SMC4	structural maintenance of chromosomes protein 4 [Homo sapiens]	425826.3191	419067.1359	390567.2006	422878.6275	480280.5224	371570.5968	320933.5518	270845.9239	328947.0367
SMCHD1	structural maintenance of chromosomes flexible hinge domain-containing prot	79664.51665	87642.33294	103812.0447	88406.01353	113746.9375	146490.7785	85966.20975	64914.02054	103605.8227
SMCR7	mitochondrial dynamic protein MID49 isoform 2 [Homo sapiens]	0	0	45680.96015	18353.47714	55618.24392	14619.44966	0	0	0
SMDT1	single-pass membrane protein with aspartate-rich tail 1, mitochondrial prec	0	515532.0327	480902.5682	86587.40909	219937.7045	330626.7159	0	0	0
SMEK1	serine/threonine-protein phosphatase 4 regulatory subunit 3A [Homo sapiens]	154759.536	197367.5003	173853.3154	197347.321	214392.2905	221382.5648	197578.6616	170596.0191	205673.0805
SMEK2	serine/threonine-protein phosphatase 4 regulatory subunit 3B isoform 1 [Hor	75946.36534	94761.64836	112087.8958	94533.30228	114681.6553	102270.2635	69371.92191	34737.82225	82222.51077
SMG1	serine/threonine-protein kinase SMG1 [Homo sapiens]	0	9708.987086	11395.80902	9818.503959	11191.5891	10676.39866	6189.726401	7460.73442	6664.678908
SMG5	protein SMG5 [Homo sapiens]	0	0	0	0	0	0	14784.18605	0	17658.92025
SMG6	telomerase-binding protein EST1A isoform 1 [Homo sapiens]	0	15287.59558	20702.17259	4907.341578	20866.44589	18909.70537	0	0	0
SMG7	protein SMG7 isoform 4 [Homo sapiens]	23475.36776	26629.00781	48718.08693	0	35361.36979	37243.27344	23681.92188	0	17852.83496
SMG8	protein SMG8 [Homo sapiens]	40219.69682	20849.46579	82800.41819	112098.7761	108685.4681	82060.24461	91419.36501	88097.96733	82885.43666
SMG9	protein SMG9 [Homo sapiens]	269405.8583	130971.3708	388417.9903	141096.5161	452940.3117	137531.5715	146387.5984	146995.6525	303147.4237
SMIM11	small integral membrane protein 11 [Homo sapiens]	502573.6379	586781.0172	709796.3276	624133.8103	0	619301.72	514483.6207	0	796149.3879
SMIM13	small integral membrane protein 13 [Homo sapiens]	0	0	0	0	0	0	186262.488	0	367851.8774
SMIM20	small integral membrane protein 20 [Homo sapiens]	2351211.318	4163218.241	4443727.215	4071330.148	5015461.481	4111050.389	4172926.178	3392388.444	4003509.667
SMIM4	small integral membrane protein 4 [Homo sapiens]	1835947.179	2097185.244	1918243.769	1833877.295	2211591.538	2033516.974	1600522.684	1352578.115	1713897.872
SMN1	survival motor neuron protein isoform d [Homo sapiens]	1385835.494	1428675.264	930064.3964	1436433.058	1885398.888	1475633.564	1413026.339	1101369.407	1461593.276
SMNDC1	survival of motor neuron-related-splicing factor 30 [Homo sapiens]	1058556.565	1291119.963	1465419.376	666571.5477	1581706.511	1408682.801	1039026.194	1186327.783	1694899.908
SMPD4	sphingomyelin phosphodiesterase 4 isoform 1 [Homo sapiens]	69354.8532	106863.2256	112779.873	0	129354.5547	85139.2792	101018.215	0	133357.8034
SMPD4	sphingomyelin phosphodiesterase 4 isoform 2 [Homo sapiens]	69354.8532	106863.2256	112779.873	0	129354.5547	85139.2792	101018.215	0	133357.8034
SMPDL3B	acid sphingomyelinase-like phosphodiesterase 3b isoform 1 precursor [Homo s	154605.9539	347056.4918	202904.2664	235503.9693	197108.8392	173044.5697	151828.3315	0	153627.7152
SMS	spermine synthase isoform 1 [Homo sapiens]	6466988.331	6907847.421	8064012.939	5703425.697	5174199.741	5773092.348	7465723.246	6060183.423	7138529.285
SMU1	WD40 repeat-containing protein SMU1 [Homo sapiens]	1282682.112	1060819.946	1208232.305	1123904.035	1339307.262	1182500.621	1061331.032	889203.8726	1080138.318
SMYD2	N-lysine methyltransferase SMYD2 [Homo sapiens]	307836.0465	321775.2926	323998.3377	83426.91134	134351.4491	113647.3895	308013.1792	264023.1507	369604.0334
SMYD3	histone-lysine N-methyltransferase SMYD3 isoform 1 [Homo sapiens]	91921.93407	138767.6573	120665.6264	114817.0769	134730.8716	110196.3379	58254.53643	89087.84275	135381.9847
SMYD4	SET and MYND domain-containing protein 4 [Homo sapiens]	8434.899066	13893.51338	0	0	25495.8007	89386.3243	62978.09091	17851.53969	0
SMYD5	SET and MYND domain-containing protein 5 [Homo sapiens]	135663.1436	225409.0426	154887.521	0	205048.9371	35888.56993	0	110536.736	260808.0315
SNAP23	synaptosomal-associated protein 23 isoform SNAP23A [Homo sapiens]	735888.0311	555834.8004	1034713.768	888444.0376	1108402.925	914001.2665	726329.3265	501344.6063	760774.191
SNAP29	synaptosomal-associated protein 29 [Homo sapiens]	825712.4408	733714.1266	796445.1886	799639.518	812965.3744	956173.5296	702463.4132	855425.5344	490192.2688
SNAPIN	SNARE-associated protein Snapin [Homo sapiens]	1182079.904	1573333.719	4124342.779	1416288.187	1246032.524	1795569.247	1474943.789	1476972.559	1215738.71
SNCG	gamma-synuclein [Homo sapiens]	166932.1964	171132.0667	0	315490.4643	0	274564.6439	0	0	0
SND1	staphylococcal nuclease domain-containing protein 1 [Homo sapiens]	2288901.151	2662674.826	3178751.158	2747498.397	3093262.153	2823415.719	2639660.036	2282461.319	2717846.943
SNF8	vacuolar-sorting protein SNF8 [Homo sapiens]	371114.4322	549701.6632	547726.5447	264728.9368	717772.5943	329165.5619	348991.386	323804.2006	852742.9632
SNIP1	smad nuclear-interacting protein 1 [Homo sapiens]	0	0	0	0	0	0	227402.7295	75361.86423	0
SNRNP200	U5 small nuclear ribonucleoprotein 200 kDa helicase [Homo sapiens]	1196906.781	1366911	971927.7335	1348420.597	979650.3415	906602.1406	1342280.441	683197.9757	839749.7826
SNRNP40	U5 small nuclear ribonucleoprotein 40 kDa protein [Homo sapiens]	2824597.53	3218030.69	3534911.388	3043785.94	3690576.26	2656784.503	2446295.17	2378370.002	2985290.142
SNRNP70	U1 small nuclear ribonucleoprotein 70 kDa [Homo sapiens]	2710019.419	3076131.072	3849027.303	3337292.365	3749698.698	3422326.308	3444177.935	1990000.227	3509358.268
SNRPA	U1 small nuclear ribonucleoprotein A [Homo sapiens]	4913874.155	5217424.84	6056244.375	4847047.776	6063947.159	5532744.484	4811389.707	4113617.287	5552172.551
SNRPA1	U2 small nuclear ribonucleoprotein A' [Homo sapiens]	5109532.497	6341995.429	6229776.712	5241149.353	5965267.074	5819232.553	5445804.89	4668115.992	7015894.433
SNRPB2	U2 small nuclear ribonucleoprotein B'' [Homo sapiens]	1832430.264	2318782.969	2157679.325	2036680.402	2232514.435	1888712.116	1960947.974	1457830.298	1794009.83
SNRPC	U1 small nuclear ribonucleoprotein C [Homo sapiens]	12260536.18	15804363.56	14401410.23	3109797.255	13072377.39	15584477.55	15213886.45	2735867.845	15650811.8
SNRPD1	small nuclear ribonucleoprotein Sm D1 [Homo sapiens]	31872424.24	25556774.43	40337383.61	28839361.33	41301783.04	32443826.41	29117366.88	24524395.43	28566546.06
SNRPD2	small nuclear ribonucleoprotein Sm D2 isoform 1 [Homo sapiens]	14657908.52	10159171.21	12636555.01	8131591.667	11136399.98	16519323.72	15088085.88	12490381.74	17790497.16
SNRPD3	small nuclear ribonucleoprotein Sm D3 [Homo sapiens]	27669701.42	27612518.23	36635078.11	28603690.32	36400097.22	27246853.2	27114688.89	21856678.91	28431495.91
SNRPE	small nuclear ribonucleoprotein E [Homo sapiens]	32787352.92	26206247.85	39822403.26	33382965.46	38758568.54	31504252.26	30276514.18	20743604.55	28894310.49
SNRPF	small nuclear ribonucleoprotein F [Homo sapiens]	9271875.603	10891291.97	24999782.06	16034636.49	26559135.03	16645879.18	11515922.65	5659940.437	20111247.08
SNRPG	small nuclear ribonucleoprotein G [Homo sapiens]	46126747.73	18929720.99	32585518.63	54289867.33	63753252.83	62926016.93	28263685.99	45833650.27	27062611.14
SNRPN	small nuclear ribonucleoprotein-associated protein N [Homo sapiens]	7057755.658	9013512.505	9368661.745	9306781.8	10003328.84	9172251.502	9014130.607	7003983.432	9968565.787
SNTB2	beta-2-syntrophin [Homo sapiens]	264699.766	245400.6285	315831.0803	308231.4664	335889.2923	288221.1752	243708.1638	191338.2385	321786.2438
SNUPN	snurportin-1 [Homo sapiens]	263984.9708	799504.322	145403.8812	394703.2979	112635.9716	493528.0532	352754.5674	370151.274	704494.6206
SNW1	SNW domain-containing protein 1 [Homo sapiens]	580755.434	755198.5135	821752.2071	754762.966	830047.7919	830802.3296	668621.9636	647353.0259	643542.7714
SNX1	sorting nexin-1 isoform a [Homo sapiens]	852402.2967	1138350.184	1139138.227	1020251.717	1225043.257	0	1011131.543	864560.8715	0
SNX12	sorting nexin-12 isoform 1 [Homo sapiens]	1803240.004	2091726.973	2357296.202	1565317.901	2085148.11	2434236.084	1714545.861	1524944.612	1878509.754
SNX15	sorting nexin-15 isoform A [Homo sapiens]	0	0	0	0	0	0	0	0	0
SNX17	sorting nexin-17 isoform 1 [Homo sapiens]	220977.0599	251674.1024	251512.6209	562635.9573	840148.7161	307954.4488	212836.4592	140271.2038	67009.38479
SNX18	sorting nexin-18 isoform b [Homo sapiens]	0	129298.5266	192418.3805	156166.9003	0	142311.8657	123510.6148	0	137624.1569
SNX2	sorting nexin-2 isoform 1 [Homo sapiens]	718702.2359	604600.765	867736.6264	750961.8068	966744.8705	735791.455	2279372.506	648149.1655	749227.1664
SNX27	sorting nexin-27 [Homo sapiens]	346814.9826	471166.9171	667018.1147	446066.659	806770.1208	487496.3416	405690.4128	0	515901.1701
SNX3	sorting nexin-3 isoform a [Homo sapiens]	1555747.707	2144152.763	1551295.016	2140159.305	1979487.806	2095578.05	2028247.917	1791694.973	1972366.187
SNX4	sorting nexin-4 [Homo sapiens]	54886.20685	85943.87848	17917.89784	0	80529.67005	0	0	0	0
SNX5	sorting nexin-5 [Homo sapiens]	187935.906	491385.6138	333825.9066	517537.3325	334947.4551	335524.2855	510123.8261	190563.1443	535490.3046
SNX6	sorting nexin-6 isoform b [Homo sapiens]	524821.9234	559103.1	732327.2657	618109.1955	690296.7313	628918.3109	567045.4498	477461.1292	525978.8443
SNX8	sorting nexin-8 [Homo sapiens]	84920.00212	152189.0445	243617.4911	206723.9947	262860.7641	216954.5587	189368.9759	126528.6574	316940.842
SNX9	sorting nexin-9 [Homo sapiens]	161501.1567	195268.5341	139419.2957	219536.8293	118579.5952	265458.8069	199339.9786	168615.6838	266007.7105
SOAT1	sterol O-acyltransferase 1 isoform 1 [Homo sapiens]	327902.5851	820097.1683	526774.4858	549804.6566	666315.1106	775243.2233	709140.4619	413741.3058	826491.9048
SOD1	superoxide dismutase [Cu-Zn] [Homo sapiens]	41800791.04	51853253.94	39778496.02	56862934.26	38156070.79	52713049.6	53210128.49	47644941.83	54981312.36
SOD2	superoxide dismutase [Mn], mitochondrial isoform A precursor [Homo sapiens]	11990605.93	13971566.37	13687423.86	13059766.74	12052904.2	12503621.16	15409721.97	11199680.22	14653277.45
SOGA2	protein SOGA2 [Homo sapiens]	5981.839905	0	10063.56612	5520.346668	10366.83557	5815.616249	6130.122127	7368.683345	251012.1256
SOLH	calpain-15 [Homo sapiens]	0	0	0	0	0	0	0	0	0
SORBS3	vinexin isoform 1 [Homo sapiens]	15668.2718	0	0	36366.08562	120963.1904	48637.59075	40911.78648	50865.88879	0
SORD	sorbitol dehydrogenase [Homo sapiens]	2637830.732	3613647.675	3885766.543	3356700.29	3167421.712	3804940.326	3356564.995	2109400.773	3514473.449
SOWAHC	ankyrin repeat domain-containing protein SOWAHC [Homo sapiens]	105009.4623	104195.395	146880.2692	116111.4504	135885.5619	114147.3898	120060.8202	69216.42175	120518.9376
SP1	transcription factor Sp1 isoform a [Homo sapiens]	762901.9496	931312.5211	993779.0647	993118.4278	1155536.978	926468.4594	884978.5489	694028.4408	963869.4526
SP100	nuclear autoantigen Sp-100 isoform 2 [Homo sapiens]	50661.17015	0	60882.70087	85452.8502	0	36694.95226	84434.00903	0	88477.66313
SPAG1	sperm-associated antigen 1 [Homo sapiens]	35222.52084	39613.83333	46045.6305	43501.21263	66072.39655	49563.67808	36493.46418	29064.25728	45152.49772
SPAG5	sperm-associated antigen 5 [Homo sapiens]	121112.8853	132138.0984	167769.8674	133336.5344	201986.5568	150711.1827	115011.1181	94026.70993	97278.98131
SPAG7	sperm-associated antigen 7 [Homo sapiens]	1146041.422	1463327.494	1595091.927	1273589.368	1602621.058	1392506.128	1222567.386	822025.6373	1146161.346
SPAG9	C-Jun-amino-terminal kinase-interacting protein 4 isoform 2 [Homo sapiens]	0	0	0	0	0	0	115794.4813	117886.1663	5037.21742
SPAG9	C-Jun-amino-terminal kinase-interacting protein 4 isoform 1 [Homo sapiens]	0	0	0	0	0	0	115794.4813	117886.1663	5037.21742
SPAST	spastin isoform 1 [Homo sapiens]	102118.0458	167392.7801	80532.76071	308753.4726	82740.34286	256979.233	105051.1667	80195.87082	136667.8679
SPATA5	spermatogenesis-associated protein 5 [Homo sapiens]	137438.3763	172277.428	172298.2025	154530.322	201396.8461	181936.707	113142.9757	109769.8639	169997.5449
SPATA5L1	spermatogenesis-associated protein 5-like protein 1 [Homo sapiens]	55152.99602	68666.53679	54103.79475	86230.87251	51461.801	66238.59532	86085.8612	67928.04542	63096.84114
SPATS2	spermatogenesis-associated serine-rich protein 2 [Homo sapiens]	296540.5034	331062.8137	346908.2166	301659.2033	364544.0474	370302.9025	287392.676	229061.8977	299892.6572
SPATS2L	SPATS2-like protein isoform a [Homo sapiens]	35500.70036	25180.71164	60450.4357	56038.83722	62313.97473	63675.37502	91001.97971	56236.93321	37173.78386
SPC24	kinetochore protein Spc24 [Homo sapiens]	815871.3339	659593.1549	1444748.72	754668.3808	1494895.859	562982.3204	630293.2359	1067886.167	491690.8751
SPC25	kinetochore protein Spc25 [Homo sapiens]	405764.9428	299906.4482	496046.2283	439817.3145	0	394954.9572	242878.0594	229509.849	330673.421
SPCS1	signal peptidase complex subunit 1 [Homo sapiens]	4595733.478	6043961.444	5818088.19	5019639.62	6463073.832	5183814.844	6136984.768	4490854.049	6826927.92
SPCS2	signal peptidase complex subunit 2 [Homo sapiens]	645015.9786	2997269.834	3748922.149	3577645.294	3401296.326	2318795.554	2640821.569	2407538.18	3547395.074
SPCS3	signal peptidase complex subunit 3 [Homo sapiens]	4960837.169	5510705.712	0	7287979.262	6994757.983	4328476.223	7529365.877	4684816.255	5853047.485
SPDL1	protein Spindly [Homo sapiens]	28720.74071	82349.27027	61368.63338	41259.3277	0	36974.39865	0	0	0
SPECC1	cytospin-B isoform 1 [Homo sapiens]	65550.84759	74544.56613	41609.24693	84070.63276	45975.47879	51974.84151	33701.18558	72536.78687	84037.60481
SPECC1L	cytospin-A isoform 1 [Homo sapiens]	60586.71788	56956.89487	19980.01986	9306.270052	57283.77985	45876.23698	58753.80348	129206.1687	46468.54142
SPEN	msx2-interacting protein [Homo sapiens]	7320.780452	3854.137688	12703.35183	10450.41466	0	8928.893294	9833.090236	5948.259741	23860.49546
SPG20	spartin [Homo sapiens]	685147.6321	564976.0266	1265635.576	1077693.482	1293802.343	923458.2422	991910.0025	760629.7494	861250.054
SPG21	maspardin isoform a [Homo sapiens]	350208.8293	348720.1298	601239.4976	559893.8911	349509.8782	406909.1298	296519.232	166498.2396	1469370.288
SPHK1	sphingosine kinase 1 isoform 2 [Homo sapiens]	0	0	0	0	0	0	0	0	0

SPIN1	spindlin-1 [Homo sapiens]	0	0	144614.4483	163481.5582	173350.2421	240707.152	192484.2662	262765.2672	0
SPNS1	protein spinster homolog 1 isoform 1 [Homo sapiens]	207341.3702	223831.9062	291349.9617	261657.1851	301555.0237	240102.15	206927.8503	157752.0025	241188.651
SPPL2A	signal peptide peptidase-like 2A precursor [Homo sapiens]	0	0	0	0	0	0	624602.3515	489922.6504	0
SPR	sepiapterin reductase [Homo sapiens]	2368516.375	2790778.345	3104075.028	2364237.425	3086914.274	2196222.788	2110436.851	1873423.204	3069097.352
SPRYD4	SPRY domain-containing protein 4 [Homo sapiens]	1801604.339	1370631.865	2156178.418	1319800.074	1659412.747	1446515.103	1250031.553	876784.2535	1517352.926
SPRYD7	SPRY domain-containing protein 7 isoform 1 [Homo sapiens]	0	745792.0522	489105.1833	272013.173	423252.6458	500895.0897	273479.3958	381790.35	0
SPTAN1	spectrin alpha chain, non-erythrocytic 1 isoform 2 [Homo sapiens]	520457.2736	547144.472	695289.1128	542292.3108	743213.5056	536346.5091	497463.5154	0	552972.8063
SPTBN1	spectrin beta chain, non-erythrocytic 1 isoform 1 [Homo sapiens]	392566.5627	424380.995	507267.4027	518769.1044	552602.1956	428468.9051	424588.4574	290288.3367	618696.1204
SPTBN2	spectrin beta chain, non-erythrocytic 2 [Homo sapiens]	95970.71339	93790.02774	94301.59706	91059.47864	145012.8837	94555.84626	86017.50257	55598.88745	70515.6077
SPTLC1	serine palmitoyltransferase 1 isoform a [Homo sapiens]	575538.6043	729393.6099	984760.6673	677892.155	938734.1246	718293.617	745197.3854	485926.4951	897560.1275
SPTLC2	serine palmitoyltransferase 2 [Homo sapiens]	215858.3588	215438.1403	304314.1526	241041.5841	207231.1221	156623.6645	164085.9467	194880.5936	290903.7151
SQLE	squalene monooxygenase [Homo sapiens]	141687.0248	51923.20531	54455.01995	49109.33575	183081.0072	151239.5628	202943.0906	182799.6691	233196.7222
SQRDL	sulfide:quinone oxidoreductase, mitochondrial [Homo sapiens]	561551.0093	690964.7601	753179.394	715479.6464	866932.0765	780767.3284	726909.4847	664351.5672	908572.5104
SQSTM1	sequestosome-1 isoform 1 [Homo sapiens]	1699038.095	1835840.461	2033237.436	2558750.172	2110456.114	2783529.221	2641930.583	2266047.363	2681381.733
SRA1	steroid receptor RNA activator 1 isoform 1 [Homo sapiens]	561617.063	749118.5195	734219.1876	1066234.494	911872.0053	679381.0599	986460.859	525513.5302	807629.3245
SRBD1	S1 RNA-binding domain-containing protein 1 [Homo sapiens]	0	24669.20214	25989.10234	0	24408.35797	20848.94985	0	0	0
SRCAP	helicase SRCAP [Homo sapiens]	11835.45436	9673.976762	15617.59489	8082.704535	11349.43466	11565.87861	10985.74029	0	11859.98184
SREK1	splicing regulatory glutamine/lysine-rich protein 1 isoform a [Homo sapiens	308830.0143	268778.3286	320249.7024	332503.4106	312623.7969	398924.1361	367191.0464	310601.4783	360010.3982
SRFBP1	serum response factor-binding protein 1 [Homo sapiens]	44875.19831	44960.75738	44852.33439	49343.69464	58577.60232	34555.29984	29002.38502	0	50654.9362
SRGAP1	SLIT-ROBO Rho GTPase-activating protein 1 [Homo sapiens]	70208.87164	94074.92377	0	70390.06881	35596.74888	62084.21525	82919.3017	65856.11953	85290.53531
SRI	sorcin isoform A [Homo sapiens]	7140885.161	5815227.927	10557282	9342183.595	11898187.41	9123898.739	8496305.161	6904213.122	6540648.422
SRM	spermidine synthase [Homo sapiens]	7037962.344	5703831.934	9206757.634	9213894.312	10624998.54	9225508.096	8338314.717	7102987.712	8760673.369
SRP14	signal recognition particle 14 kDa protein [Homo sapiens]	14556306.14	19708823.08	24631055.76	19584831.68	23491199.02	20617580.76	17195164.92	12843370.75	18178128.14
SRP19	signal recognition particle 19 kDa protein isoform 1 [Homo sapiens]	1371507.67	2028208.71	1334736.525	1860028.913	2033522.983	1064803.493	1897904.201	2070915.173	1894612.199
SRP54	signal recognition particle 54 kDa protein isoform 1 [Homo sapiens]	1073975.306	1153352.945	1817131.738	1425248.418	1539526.809	1578992.442	1355303.088	1162539.368	1068706.446
SRP68	signal recognition particle subunit SRP68 isoform 1 [Homo sapiens]	801628.6547	945896.1993	1149155.475	884467.7401	1141145.616	962207.9084	880395.7345	761326.6453	876325.5456
SRP72	signal recognition particle subunit SRP72 isoform 1 [Homo sapiens]	817466.6182	849900.1487	1079944.136	946870.32	1101975.289	1135445.792	1055653.208	761531.8465	1026006.213
SRP9	signal recognition particle 9 kDa protein isoform 2 [Homo sapiens]	11664147.93	20559219.11	21377138.13	20985261.9	23086369.6	21823542.87	20470639.02	17610229.67	21521290.96
SRPK1	SRSF protein kinase 1 [Homo sapiens]	890969.4301	1024958.328	1240076.736	1017755.505	1163685.839	1071773.367	995797.2268	1417661.351	1090746.64
SRPK2	SRSF protein kinase 2 isoform a [Homo sapiens]	81650.81911	133971.19	146111.1223	205258.8074	143206.9329	241413.0698	146844.1358	65799.10907	99561.66463
SRPR	signal recognition particle receptor subunit alpha isoform 1 [Homo sapiens]	496748.1982	541248.2015	634344.1347	550153.3697	697385.4919	576327.7037	599694.0683	461030.6838	533084.6668
SRPRB	signal recognition particle receptor subunit beta [Homo sapiens]	4880714.942	5954922.564	6360185.951	5713634.503	6983412.488	5619813.423	5679718.628	4409450.816	6347575.995
SRR	serine racemase [Homo sapiens]	55761.44276	205028.743	77249.90654	57937.96495	79407.74722	48387.47576	37666.46379	0	45927.0035
SRRM1	serine/arginine repetitive matrix protein 1 [Homo sapiens]	308944.3352	432853.8224	535691.1595	434139.3934	399811.9051	421891.3234	412398.6827	420164.7044	469836.9843
SRRM2	serine/arginine repetitive matrix protein 2 [Homo sapiens]	236078.1121	440983.3573	739511.2258	520055.4079	485709.8801	598329.9594	277065.1724	360727.1725	560195.1371
SRRT	serrate RNA effector molecule homolog isoform a [Homo sapiens]	679277.8333	695023.5124	922965.5423	837120.0225	827756.9732	795193.7615	802335.0921	851798.967	782529.5947
SRSF1	serine/arginine-rich splicing factor 1 isoform 1 [Homo sapiens]	9911731.688	14555278.79	12961044.62	12069442.48	12613273.47	14161783.86	14141828.54	12385771.83	14690753.19
SRSF10	serine/arginine-rich splicing factor 10 isoform 2 [Homo sapiens]	3216728.741	2641211.108	4081899.913	3596934.046	4107239.279	2472145.704	2185030.821	2702239.611	2489403.694
SRSF11	serine/arginine-rich splicing factor 11 isoform 1 [Homo sapiens]	1141338.015	960874.8026	1797480.587	1535864.664	1711344.186	1735791.874	1591225.106	1468223.354	1007089.668
SRSF2	serine/arginine-rich splicing factor 2 [Homo sapiens]	10991667.25	12874473.35	14019555.81	13732692.64	15347177.37	13662150.93	13057022.31	17402584.44	20311483.23
SRSF3	serine/arginine-rich splicing factor 3 [Homo sapiens]	25235847.03	12248126.25	15472910.06	27784351.01	11399833.44	8349854.267	25485145.9	7626340.555	9395513.769
SRSF4	serine/arginine-rich splicing factor 4 [Homo sapiens]	560805.1319	643808.1524	755492.9636	649826.8486	819434.1144	703478.7645	668917.4316	578515.3837	872291.2144
SRSF5	serine/arginine-rich splicing factor 5 [Homo sapiens]	1630660.762	2629636.744	2686155.708	2390808.147	2734447.435	1643361.865	2640417.62	2089577.056	2271245.417
SRSF6	serine/arginine-rich splicing factor 6 [Homo sapiens]	2404184.319	2757461.91	2986276.966	2636389.216	1595088.816	2699294.238	2759962.762	2306555.038	3219391.428
SRSF7	serine/arginine-rich splicing factor 7 isoform 1 [Homo sapiens]	7362482.179	8288253.477	3174359.55	7785915.775	3238927.025	5013655.953	8887545.715	8032381.205	6998125.57
SRSF8	serine/arginine-rich splicing factor 8 [Homo sapiens]	39852.86853	86182.25862	171370.4713	108155.449	141092.0776	84097.97414	0	0	0
SRSF9	serine/arginine-rich splicing factor 9 [Homo sapiens]	2980069.197	3562404.118	3405609.702	1826050.61	3578017.3	3686993.791	3684629.762	2626000.44	3483497.985
SRXN1	sulfiredoxin-1 [Homo sapiens]	0	0	0	0	0	0	464028.7561	248212.7046	0
SS18	protein SSXT isoform 1 [Homo sapiens]	1226253.51	189696.8163	315765.2245	1098167.791	0	1542875.582	1178466.816	1219655.949	162122.7577
SS18L2	SS18-like protein 2 [Homo sapiens]	0	363300.3671	279769.2339	470197.12	0	261516.4088	354750.5768	256500.1008	159870.7339
SSB	lupus La protein [Homo sapiens]	6144035.749	6367563.04	7839677.427	6966948.802	7573791.731	7025895.568	6476892.612	5149415.026	6689240.405
SSBP1	single-stranded DNA-binding protein, mitochondrial precursor [Homo sapiens]	7373969.879	9763126.951	9739072.051	7958600.694	9018082.688	10816689.84	9305731.676	8631975.954	11038285.02
SSBP4	single-stranded DNA-binding protein 4 isoform a [Homo sapiens]	85059.91558	0	159371.9026	141061.8564	172283.461	185362.1956	0	0	0
SSFA2	sperm-specific antigen 2 isoform 1 [Homo sapiens]	97217.38328	89269.32294	98086.28468	98289.86086	120448.0569	118115.3888	103577.447	79061.06957	81584.18235
SSH3	protein phosphatase Slingshot homolog 3 [Homo sapiens]	89096.63095	56908.92648	126834.6106	75756.4111	161298.3228	84288.94076	106525.1365	139923.9709	0
SSNA1	Sjoegren syndrome nuclear autoantigen 1 [Homo sapiens]	757581.9258	1388986.518	1031813.394	0	1239380.009	0	729704.707	408010.0742	1264934.844
SSR1	translocon-associated protein subunit alpha precursor [Homo sapiens]	16349445.73	18777390.7	20564129.1	18118327.65	21626381.68	18353142.72	19755104.86	15168821.42	21206697.3
SSR2	translocon-associated protein subunit beta precursor [Homo sapiens]	70644.87972	31197.62368	76383.09994	0	131638.1686	45324.21772	0	0	0
SSR3	translocon-associated protein subunit gamma [Homo sapiens]	6830960.884	15735899.42	9376111.119	14885120.86	12497619.93	17743819.05	16516675.94	12859071.58	15368229.02
SSR4	translocon-associated protein subunit delta isoform 1 precursor [Homo sapie	30249175.61	28203237.88	37850808.1	31734861.98	38403098.43	32656744.25	34476818.31	28058730.02	31632373.88
SSRP1	FACT complex subunit SSRP1 [Homo sapiens]	1974179.876	2634348.323	2669391.306	2384643.752	2735730.343	2583903.908	2524485.38	2234011.172	2701390.3
SSSCA1	Sjoegren syndrome/scleroderma autoantigen 1 [Homo sapiens]	3531900.798	5230490.794	4912469.422	3012512.972	3603757.758	4522192.602	4364471.236	4343995.625	6270538.656
SSU72	RNA polymerase II subunit A C-terminal domain phosphatase SSU72 [Homo sapie	1320706.33	1914930.813	1723321.321	1622446.376	1319919.396	1720267.568	1659845.603	1348929.353	1659711.049
ST13	hsc70-interacting protein isoform 1 [Homo sapiens]	8748628.071	7789901.955	10958784.71	7357426.708	8707073.799	9885613.786	9777112.584	7908638.39	10260195.6
ST6GALNAC4	alpha-N-acetyl-neuraminyl-2,3-beta-galactosyl-1,3-N-acetyl-galactosaminide	0	0	0	0	0	0	0	0	0
STAG1	cohesin subunit SA-1 [Homo sapiens]	33043.35113	79729.48577	16174.13142	138532.3157	0	96658.93002	86133.26618	77709.74281	117480.6615
STAG2	cohesin subunit SA-2 isoform b [Homo sapiens]	150139.9382	0	187712.8271	157277.8679	190691.2076	160448.9317	147419.6678	118970.026	143248.2723
STAG2	cohesin subunit SA-2 isoform a [Homo sapiens]	150139.9382	0	187712.8271	157277.8679	190691.2076	160448.9317	147419.6678	118970.026	143248.2723
STAM	signal transducing adapter molecule 1 [Homo sapiens]	864884.7848	1064328.466	1212743.142	995650.4638	1049976.596	915502.6399	1119389.073	973648.4058	1127446.598
STAM2	signal transducing adapter molecule 2 [Homo sapiens]	0	0	0	0	0	0	86929.38411	290911.3634	85126.87086
STAMBP	STAM-binding protein [Homo sapiens]	94091.16566	168234.093	107441.901	86399.67018	0	193439.1751	147266.8253	95854.11542	171271.3603
STAMBPL1	AMSH-like protease [Homo sapiens]	232802.4565	126850.6756	201409.1568	158515.1124	232409.0929	169996.3181	165634.1745	101641.1595	219925.8187
STAT1	signal transducer and activator of transcription 1-alpha/beta isoform alpha	1007442.768	1096889.299	1340628.732	1135146.388	1524241.916	1211039.911	1159795.493	998081.8357	1276213.628
STAT2	signal transducer and activator of transcription 2 isoform 1 [Homo sapiens]	67965.91777	91877.78877	106418.6885	106540.0851	100012.8605	113426.5415	65244.94296	61569.37043	89614.09947
STAT3	signal transducer and activator of transcription 3 isoform 1 [Homo sapiens]	269618.5071	302991.5038	409445.3691	737871.995	419158.3079	317788.6081	774916.511	601742.863	309907.6032
STAT3	signal transducer and activator of transcription 3 isoform 2 [Homo sapiens]	269618.5071	302991.5038	409445.3691	737871.995	419158.3079	317788.6081	774916.511	601742.863	309907.6032
STAT5A	signal transducer and activator of transcription 5A [Homo sapiens]	93117.87191	80982.0729	28292.12633	95064.6191	59892.9947	100755.0976	112398.1387	80693.68117	51927.55675
STAT5B	signal transducer and activator of transcription 5B [Homo sapiens]	233419.1395	166237.4807	239668.5406	214645.498	403526.1356	207055.4202	167024.0501	135720.9778	357883.4234
STAT6	signal transducer and activator of transcription 6 isoform 1 [Homo sapiens]	184203.4191	150401.9972	160964.9351	171314.8027	159292.2698	180750.0304	152650.6074	143577.8113	173989.2713
STAU1	double-stranded RNA-binding protein Staufen homolog 1 isoform b [Homo sapie	36576.61354	101992.7241	130213.1189	0	148408.3275	92655.52266	0	0	0
STAU2	double-stranded RNA-binding protein Staufen homolog 2 isoform a [Homo sapie	165969.0382	140737.6935	218776.5809	162425.8963	226852.6012	242775.4544	232584.2138	121424.3421	0
STBD1	starch-binding domain-containing protein 1 [Homo sapiens]	0	0	0	0	0	0	0	62004.52818	120724.4749
STEAP3	metalloreductase STEAP3 isoform a [Homo sapiens]	0	0	0	0	0	0	0	91529.9516	65331
STEAP4	metalloreductase STEAP4 isoform 1 [Homo sapiens]	1180019.319	1511819.151	2210713.959	1625002.947	1599062.122	1676625.859	1084285.408	846790.0203	1217803.075
STIM1	stromal interaction molecule 1 isoform 2 precursor [Homo sapiens]	101004.8219	121024.0765	235759.9723	261253.0177	247408.9064	195404.6431	200607.0997	0	205512.3724
STIM2	stromal interaction molecule 2 isoform 1 precursor [Homo sapiens]	0	0	0	0	0	0	7043.15378	10589.65206	0
STIP1	stress-induced-phosphoprotein 1 [Homo sapiens]	7918664.163	8643188.142	9724297.903	9482712.796	10437764.85	9840182.886	9218447.249	8295342.581	10178066.09
STK10	serine/threonine-protein kinase 10 [Homo sapiens]	74108.72864	14019.7569	92566.1258	41225.2658	78758.85722	87135.42047	71715.8545	84143.50938	64533.2711
STK24	serine/threonine-protein kinase 24 isoform b [Homo sapiens]	0	0	0	0	0	0	321087.6273	263904.625	0
STK24	serine/threonine-protein kinase 24 isoform a [Homo sapiens]	0	0	0	0	0	0	321087.6273	263904.625	0
STK3	serine/threonine-protein kinase 3 isoform 2 [Homo sapiens]	68119.79953	139373.8237	27017.51563	97083.41745	106486.7358	140318.1132	12385.72524	52689.37292	117518.3396
STK38	serine/threonine-protein kinase 38 [Homo sapiens]	165429.3639	685820.3802	190713.8188	166784.3986	213210.5695	156560.8627	189923.985	624258.4802	615344.487
STK39	STE20/SPS1-related proline-alanine-rich protein kinase [Homo sapiens]	107019.4699	163971.9643	286425.4841	146348.4076	222657.6295	234763.4884	237843.9987	146480.6263	73764.18814
STK4	serine/threonine-protein kinase 4 [Homo sapiens]	145868.7846	0	188096.8564	115828.3167	155181.441	165242.0333	107608.3988	62928.86862	66466.22538
STMN1	stathmin isoform a [Homo sapiens]	6181552.935	0	7874555.505	0	7518802.535	8471746.76	8218660.72	7651767.635	0
STMN1	stathmin isoform b [Homo sapiens]	6181552.935	0	7874555.505	0	7518802.535	8471746.76	8218660.72	7651767.635	0
STOM	erythrocyte band 7 integral membrane protein isoform a [Homo sapiens]	1834427.911	2609806.673	2490295.928	7405272.725	2682707.663	6681865.548	2387712.525	1930395.835	2717779.707
STOML2	stomatin-like protein 2, mitochondrial [Homo sapiens]	1943232.765	1967860.186	2755390.358	2202972.562	2303094.625	2223729.528	2041772.815	1825247.664	2307670.311
STRA13	centromere protein X isoform 1 [Homo sapiens]	113897.5857	727947.2571	0	0	195101.5857	672536.4	422733.8143	348345.2857	525902.5214
STRAP	serine-threonine kinase receptor-associated protein [Homo sapiens]	6676967.421	7794829.187	8789983.423	7472671.695	8860824.935	7769215.277	7470927.185	6270721.798	8249067.707
STRBP	spermatid perinuclear RNA-binding protein isoform 1 [Homo sapiens]	539581.7253	607256.7892	724882.3687	440335.2828	619612.3572	853168.9259	700878.3818	751313.5249	696836.2545
STRIP1	striatin-interacting protein 1 isoform 1 [Homo sapiens]	59546.98442	110950.4738	41313.50455	87022.3476	55501.7996	43757.76636	100422.6338	87212.65084	95150.53259
STRN	striatin [Homo sapiens]	125922.4518	134840.1564	216078.46	81724.54689	218350.3327	156257.481	151757.7235	126506.9199	102151.1319
STRN3	striatin-3 isoform 2 [Homo sapiens]	93329.73765	0	117457.7457	89487.27965	0	95664.60955	0	79738.24455	156096.9498
STRN3	striatin-3 isoform 1 [Homo sapiens]	93329.73765	0	117457.7457	89487.27965	0	95664.60955	0	79738.24455	156096.9498
STRN4	striatin-4 isoform 2 [Homo sapiens]	92012.38539	65718.87759	184856.8347	47290.74689	171574.0851	89462.74585	131123.1452	59878.68975	205675.0381
STT3A	dolichyl-diphosphooligosaccharide--protein glycosyltransferase subunit STT3	1645869.165	1870202.873	1910483.509	2008506.344	2264328.318	2077889.219	1932316.535	1512837.594	2290357.834
STT3B	dolichyl-diphosphooligosaccharide--protein glycosyltransferase subunit STT3	686697.5611	846452.8596	703206.7829	852591.8306	873836.0794	817445.898	866267.9702	597358.7659	917313.3373
STUB1	E3 ubiquitin-protein ligase CHIP [Homo sapiens]	1373385.533	1581744.744	1911829.656	1571405.84	1984211.814	1667209.136	1627450.895	1318838.839	1674584.131
STX10	syntaxin-10 isoform 1 [Homo sapiens]	334034.7074	272772.8737	630868.4665	572790.8474	723508.2518	460423.2536	562459.3782	447154.0732	593387.4009
STX12	syntaxin-12 [Homo sapiens]	455547.1445	364281.8969	713758.8301	444013.7718	473633.6505	232305.6117	687288.5922	528973.5218	197248.3119
STX16	syntaxin-16 isoform a [Homo sapiens]	85888.75105	137465.6265	189945.3727	196302.3756	165563.9043	245505.6379	160186.4281	0	338957.7475
STX18	syntaxin-18 [Homo sapiens]	124814.5312	66203.63961	154806.0871	77295.70565	185305.4616	105950.556	83604.72929	64021.82914	38270.2289
STX4	syntaxin-4 isoform 3 [Homo sapiens]	266597.624	363989.8256	554507.9157	218895.4278	87121.60853	156387.9908	256114.1912	284092.3411	378564.8863
STX5	syntaxin-5 isoform 1 [Homo sapiens]	1121850.627	318219.1393	497550.6536	720756.2362	446765.829	598018.4143	489911.7942	1536087.991	365901.5346
STX6	syntaxin-6 [Homo sapiens]	317080.7566	785180.9559	611823.532	595197.7191	467242.9176	748383.3632	676326.5336	675723.1395	1020196.972
STX7	syntaxin-7 [Homo sapiens]	934033.564	977353.0595	1122155.812	1053985.245	1058131.436	1026738.442	1063136.204	690633.4472	1017650.672
STX8	syntaxin-8 [Homo sapiens]	217972.6569	366222.7133	399004.0944	323717.0816	476866.4209	248270.5836	145115.2239	299895.8291	324518.6701
STXBP1	syntaxin-binding protein 1 isoform a [Homo sapiens]	0	0	74767.0612	76573.3413	89850.5137	47898.40662	0	68211.4619	55900.79935
STXBP1	syntaxin-binding protein 1 isoform b [Homo sapiens]	0	0	74767.0612	76573.3413	89850.5137	47898.40662	0	68211.4619	55900.79935

STXBP2	syntaxin-binding protein 2 isoform c [Homo sapiens]	215252.1717	206666.7409	293566.2698	287469.4773	311825.2771	233455.8239	292439.1074	197527.3469	286388.9239
STXBP3	syntaxin-binding protein 3 [Homo sapiens]	239355.4161	215154.8923	295311.4105	276535.4849	299536.686	269983.8605	232732.0467	233217.1258	252254.3578
STXBP4	syntaxin-binding protein 4 [Homo sapiens]	0	301022.0116	0	19362.14583	102145.6678	48741.75176	44759.19502	36603.82986	213985.875
STXBP5	syntaxin-binding protein 5 isoform b [Homo sapiens]	26114.35422	26839.90699	0	23520.47757	41656.43372	0	40201.24274	17518.51352	26967.21256
SUB1	activated RNA polymerase II transcriptional coactivator p15 [Homo sapiens]	23088590.57	29655074.06	34076978.99	25829849	27123691.04	28108343.84	27910865.59	22348238.45	31860265.33
SUCLA2	succinyl-CoA ligase [ADP-forming] subunit beta, mitochondrial precursor [Ho	1146878.957	1118295.298	1197903.364	1004501.582	1225479.64	1202762.618	1280337.62	914164.2201	1210473.685
SUCLG1	succinyl-CoA ligase [ADP/GDP-forming] subunit alpha, mitochondrial precurso	2122378.153	6467013.896	3026881.578	2601970.68	2788618.893	2747836.974	2610820.772	2697912.457	3628788.7
SUCLG2	succinyl-CoA ligase [GDP-forming] subunit beta, mitochondrial isoform 1 pre	571108.2265	0	789689.1745	0	781658.407	672375.1635	608821.093	506397.2775	713011.945
SUCLG2	succinyl-CoA ligase [GDP-forming] subunit beta, mitochondrial isoform 2 pre	571108.2265	0	789689.1745	0	781658.407	672375.1635	608821.093	506397.2775	713011.945
SUDS3	sin3 histone deacetylase corepressor complex component SDS3 [Homo sapiens]	0	0	0	0	0	0	0	52537.16456	31938.3394
SUGP1	SURP and G-patch domain-containing protein 1 [Homo sapiens]	97184.37914	148121.5965	153289.5758	43486.8912	87013.41182	195671.2317	91958.89243	68082.36901	108486.6792
SUGP2	SURP and G-patch domain-containing protein 2 [Homo sapiens]	46403.69517	35927.81119	38992.8492	36172.20563	45058.55203	41695.12582	41839.00749	75271.72301	53913.01245
SUGT1	suppressor of G2 allele of SKP1 homolog isoform SGT1A [Homo sapiens]	2679699.098	111173.1747	3227148.596	127669.5197	3166253.089	2825899.848	833167.4609	1540250.361	883708.2707
SUGT1	suppressor of G2 allele of SKP1 homolog isoform SGT1B [Homo sapiens]	2679699.098	111173.1747	3227148.596	127669.5197	3166253.089	2825899.848	833167.4609	1540250.361	883708.2707
SUMF1	sulfatase-modifying factor 1 isoform 1 precursor [Homo sapiens]	0	0	0	0	0	0	243240.4152	193276.8841	239870.0972
SUMF2	sulfatase-modifying factor 2 isoform b precursor [Homo sapiens]	2026205.619	2014451.111	2642401.997	2289749.59	2761395.976	2191217.921	1868306.101	1551485.134	2343850.328
SUMO1	small ubiquitin-related modifier 1 isoform a precursor [Homo sapiens]	7766499.686	8354873.633	7878801.082	6995988.904	7888208.263	7950050.247	7726146.114	7281705.331	7342964.882
SUMO3	small ubiquitin-related modifier 3 precursor [Homo sapiens]	161099783.7	157991076.4	0	84831982.03	225023570.5	0	77434420.38	135605937.5	85323917.46
SUN1	SUN domain-containing protein 1 isoform a [Homo sapiens]	265012.0247	162827.2887	367961.0565	256030.539	374603.4667	289575.2727	258433.3423	184464.5279	120700.9963
SUN2	SUN domain-containing protein 2 isoform a [Homo sapiens]	208276.8873	188904.8209	246492.6762	204685.3524	227009.8578	370820.6451	249203.7813	197217.2405	238723.5219
SUPT16H	FACT complex subunit SPT16 [Homo sapiens]	1678523.866	1706977.097	1968006.575	1816756.736	2157809.634	1822101.683	1890296.274	1585233.711	1578855.18
SUPT3H	transcription initiation protein SPT3 homolog isoform 2 [Homo sapiens]	0	118655.1894	150981.2137	0	158204.3397	154087.8654	145704.3473	107488.7778	0
SUPT4H1	transcription elongation factor SPT4 [Homo sapiens]	185959.899	0	364600.0769	232336.9808	0	199804.1538	0	0	0
SUPT5H	transcription elongation factor SPT5 isoform a [Homo sapiens]	372707.9388	413769.1699	467418.3367	477274.3982	485554.7355	398241.0804	342951.6497	325959.4536	374909.0125
SUPT6H	transcription elongation factor SPT6 [Homo sapiens]	144662.2415	163117.2054	200439.5407	175296.2593	197369.321	177192.667	182682.6146	138062.2276	126430.4373
SUPT7L	STAGA complex 65 subunit gamma [Homo sapiens]	343538.961	250140.6719	184817.8795	161369.8463	374861.3497	247953.1254	229707.2171	395178.5893	82077.75893
SUPV3L1	ATP-dependent RNA helicase SUPV3L1, mitochondrial precursor [Homo sapiens]	154754.9461	272753.3738	211170.1733	217957.4182	274117.7031	242257.7529	204385.0337	200410.313	381356.2904
SURF1	surfeit locus protein 1 [Homo sapiens]	276667.6016	253727.3553	273736.3917	228695.9838	354402.0323	444666.525	451453.5042	228153.3657	272055.8678
SURF2	surfeit locus protein 2 [Homo sapiens]	390470.7672	246814.2755	326606.8768	0	312428.1341	0	375713.6694	258700.6483	219072.9999
SURF4	surfeit locus protein 4 [Homo sapiens]	4654704.61	5034835.403	5820689.646	5207615.838	6477453.735	5478915.993	4783718.756	4095531.199	5104044.713
SURF6	surfeit locus protein 6 [Homo sapiens]	0	0	0	0	0	0	0	99849.4833	109123.6384
SUZ12	polycomb protein SUZ12 [Homo sapiens]	139145.177	145712.4372	297684.4305	157695.8116	300649.2337	157624.1607	126869.9368	97830.50576	123239.6808
SVIL	supervillin isoform 2 [Homo sapiens]	12751.94855	0	13634.37231	17499.98779	13477.64823	20992.35463	22290.5631	12610.89656	10154.856
SWAP70	switch-associated protein 70 [Homo sapiens]	254018.7902	481521.0308	532117.628	418006.5605	566335.9098	454852.0353	538575.8469	355160.514	601019.1171
SYAP1	synapse-associated protein 1 [Homo sapiens]	1360541.614	1874340.529	2188934.091	2052360.935	2337968.39	1632439.123	1446723.971	1609009.827	1866818.275
SYBU	syntabulin isoform a [Homo sapiens]	0	0	0	0	0	0	37462.95095	44513.4462	0
SYF2	pre-mRNA-splicing factor SYF2 isoform 1 [Homo sapiens]	272348.8109	508459.5192	318966.9594	182963.1388	677821.4055	212739.7142	98435.97638	249445.5448	190439.629
SYMPK	symplekin [Homo sapiens]	193846.1353	204361.8935	456190.6721	173734.3598	398055.0192	212948.9555	157920.4581	154819.1191	172711.8999
SYNCRIP	heterogeneous nuclear ribonucleoprotein Q isoform 1 [Homo sapiens]	6779829.336	7624926.037	8930042.074	7859013.498	8609838.804	8538907.683	8109652.022	6914214.79	8688866.686
SYNE1	nesprin-1 isoform 1 [Homo sapiens]	0	0	0	0	0	0	9873.499593	37675.85746	0
SYNE2	nesprin-2 isoform 5 [Homo sapiens]	7072.488373	11048.88812	63883.99642	9238.919952	7974.110259	10102.23399	8812.459215	6315.187878	9334.292961
SYNGR2	synaptogyrin-2 [Homo sapiens]	2140926.406	2253580.786	1539544.21	2037142.066	3057675.02	2091697.406	2162393.858	1824453.827	2695536.594
SYNJ2BP-COX16	SYNJ2BP-COX16 protein isoform 1 [Homo sapiens]	1219169.676	1624574.559	1684050.428	1490200.074	1111988.467	1764993.209	1807685.658	1153908.246	1570826.23
SYNRG	synergin gamma isoform 1 [Homo sapiens]	39387.18552	25457.95383	41459.69718	52970.11678	83876.59507	32428.67754	11746.66124	29781.89554	34405.34507
SYPL1	synaptophysin-like protein 1 isoform b [Homo sapiens]	3165133.291	4353306.691	3827192.104	3892257.04	4470198.915	3519496.664	3340466.43	3506792.76	5160480.158
SYTL4	synaptotagmin-like protein 4 [Homo sapiens]	0	56233.6299	35220.87542	54868.9251	0	57850.52093	58386.08644	0	50525.70753
SYVN1	E3 ubiquitin-protein ligase synoviolin isoform b precursor [Homo sapiens]	323088.1933	180106.6336	0	324746.1086	375735.3865	182263.3923	277738.5568	191161.9408	165262.6051
SZRD1	SUZ domain-containing protein 1 isoform 1 [Homo sapiens]	0	796442.9054	504639.2112	993179.7973	0	991896.9291	797559.2218	732897.4865	625304.8814
TAB1	TGF-beta-activated kinase 1 and MAP3K7-binding protein 1 isoform alpha [Hon	127954.1858	164734.7708	192577.1934	0	167033.6588	177153.3601	138968.2813	86233.37588	0
TAB3	TGF-beta-activated kinase 1 and MAP3K7-binding protein 3 [Homo sapiens]	15073.58077	15263.16199	11063.26359	0	0	20790.27174	15935.21075	13108.57488	16680.3285
TACC1	transforming acidic coiled-coil-containing protein 1 isoform 1 [Homo sapien	113474.5417	115173.6648	101616.9573	75247.13744	90992.08	116783.9824	104325.5027	94330.51567	99531.20647
TACC2	transforming acidic coiled-coil-containing protein 2 isoform a [Homo sapien	0	0	0	0	0	0	4420.719842	34574.40434	285459.2704
TACC3	transforming acidic coiled-coil-containing protein 3 [Homo sapiens]	334268.6947	207422.8947	214999.0399	125329.2564	237366.5338	206768.0432	176067.6019	76908.38525	147996.3366
TACO1	translational activator of cytochrome c oxidase 1 [Homo sapiens]	232494.2083	365843.25	727319.6133	438906.7283	825540.1712	496337.5114	383886.982	291071.8343	585518.1414
TADA1	transcriptional adapter 1 [Homo sapiens]	0	0	0	0	0	0	0	0	0
TAF10	transcription initiation factor TFIID subunit 10 [Homo sapiens]	1407566	1316351.203	1878003.415	1000515.107	2212501.454	1613929.653	996419.409	0	3937064.066
TAF15	TATA-binding protein-associated factor 2N isoform 1 [Homo sapiens]	1327399.425	1429776.51	1590580.047	1660167.792	1640161.329	1579335.544	1529663.09	1361006.358	1460880.362
TAF4	transcription initiation factor TFIID subunit 4 [Homo sapiens]	0	0	0	0	0	0	320462.4467	0	52437.08865
TAF5L	TAF5-like RNA polymerase II p300/CBP-associated factor-associated factor 65	0	0	0	0	0	0	13252.66691	0	33894.04408
TAF6	transcription initiation factor TFIID subunit 6 isoform epsilon [Homo sapie	271392.2396	21699.12734	0	37212.03808	22082.56133	223207.9265	34693.36997	52571.5412	42322.95273
TAF6L	TAF6-like RNA polymerase II p300/CBP-associated factor-associated factor 65	105249.0096	81198.77522	100532.8535	118783.0675	206034.7955	116355.2566	57852.44303	0	110661.0614
TAF7	transcription initiation factor TFIID subunit 7 [Homo sapiens]	295633.3112	196245.2897	381344.2871	234842.6276	318327.6724	371465.3759	150884.9172	54602.50172	199368.7754
TAF8	transcription initiation factor TFIID subunit 8 [Homo sapiens]	302136.0573	0	247141.2852	268121.1183	280827.855	226583.6578	226888.0477	237512.05	137026.8533
TAF9	adenylate kinase isoenzyme 6 isoform a [Homo sapiens]	700451.9225	336232.0504	734488.471	412951.9597	860303.663	437230.3206	533316.2547	440712.5041	863113.2609
TAF9	adenylate kinase isoenzyme 6 isoform b [Homo sapiens]	700451.9225	336232.0504	734488.471	412951.9597	860303.663	437230.3206	533316.2547	440712.5041	863113.2609
TAGLN	transgelin [Homo sapiens]	1010540.579	1068923.551	1567835.193	1097128.707	1147618.395	1196693.637	1183753.26	998776.5211	1400936.013
TAGLN2	transgelin-2 isoform a [Homo sapiens]	72971206.7	82106719.94	93142962.36	86396375.96	93851336.4	95261877.38	84191231.31	74378400.1	81813369.13
TALDO1	transaldolase [Homo sapiens]	7780813.17	8048563.605	10145231.09	8177433.068	10048864.22	8132513.163	8310489.667	7118442.98	7850530.85
TAMM41	mitochondrial translocator assembly and maintenance protein 41 homolog prec	534797.0463	616177.2899	683880.0317	515230.7692	829517.2436	584527.4957	697105.7521	362266.6056	556923.7037
TANC1	protein TANC1 isoform 1 [Homo sapiens]	10471.69196	13114.67087	23455.86017	10842.9892	16270.029	24156.16338	11012.48647	0	4201.741722
TAOK1	serine/threonine-protein kinase TAO1 isoform 1 [Homo sapiens]	14753.04638	13905.21465	18045.4369	14238.78083	15458.35605	36737.16346	63834.1448	0	17086.39124
TAOK3	serine/threonine-protein kinase TAO3 [Homo sapiens]	22533.97564	13087.62511	27747.43615	19124.25469	30307.66404	46243.48477	24838.30893	0	19374.22995
TAP1	antigen peptide transporter 1 [Homo sapiens]	301484.2706	247996.5452	332717.3362	379284.485	336045.5552	298498.0093	376962.2639	357677.711	390425.719
TAP2	antigen peptide transporter 2 isoform 1 [Homo sapiens]	129497.7788	140956.2557	141700.3642	177389.4451	164405.2487	316855.7136	209898.356	138301.1913	247816.2981
TAPBP	tapasin isoform 2 precursor [Homo sapiens]	938714.8944	1075617.906	1740312.324	1144253.571	1795349.122	1214078.504	1096476.128	907449.4384	1330339.678
TAPT1	transmembrane anterior posterior transformation protein 1 homolog [Homo sap	0	35361.23537	0	30904.84634	49506.15488	38853.19939	39526.04024	214855.1024	49550.19634
TARBP1	probable methyltransferase TARBP1 [Homo sapiens]	32099.22145	45097.67089	44385.71955	30846.12448	29858.10731	32988.51072	17403.46952	26309.05769	46788.26392
TARBP2	RISC-loading complex subunit TARBP2 isoform a [Homo sapiens]	455454.7125	154910.4615	429671.8316	311889.1917	574238.5681	6281.450912	726888.7578	160727.0498	0
TARDBP	TAR DNA-binding protein 43 [Homo sapiens]	6759111.573	6231181.366	8116667.016	7342693.084	7875127.633	7276151.909	6711924.527	5362527.25	6652643.498
TARS	threonine--tRNA ligase, cytoplasmic isoform 1 [Homo sapiens]	6704297.057	8633458.921	8381636.747	8286629.722	8625261.968	9212052.665	8589124.509	7083532.832	8397922.658
TARS2	threonine--tRNA ligase, mitochondrial isoform a [Homo sapiens]	225861.4152	175737.6778	191757.4849	209213.361	200702.6748	182093.9052	189840.3421	166258.3558	196205.8348
TATDN1	putative deoxyribonuclease TATDN1 isoform a [Homo sapiens]	536433.7904	674798.9302	742322.3754	741088.0851	506025.0878	545046.1428	558030.372	287106.5348	401591.0218
TATDN3	putative deoxyribonuclease TATDN3 isoform 5 [Homo sapiens]	93949.24216	788475.6126	52332.34459	0	112312.6532	0	0	0	0
TAX1BP1	tax1-binding protein 1 isoform 1 [Homo sapiens]	39192.973	0	16377.72749	22367.25631	21982.60915	26289.78224	0	0	0
TAX1BP3	tax1-binding protein 3 isoform 1 [Homo sapiens]	9339973.211	11634749.84	12268376.82	10617304.35	12341116.17	11842214.21	10308492.84	10054590.21	11360437.78
TBC1D10B	TBC1 domain family member 10B [Homo sapiens]	27053.63406	59639.09149	97694.69384	1677211.283	44811.65942	49521.10373	68873.38428	724369.9739	54063.77083
TBC1D13	TBC1 domain family member 13 [Homo sapiens]	0	0	111505.7006	195135.2213	131101.4046	159187.7675	112077.9664	120152.3618	212712.4331
TBC1D15	TBC1 domain family member 15 isoform 2 [Homo sapiens]	0	0	0	0	0	0	0	0	0
TBC1D2	TBC1 domain family member 2A isoform 1 [Homo sapiens]	58575.43245	121798.14	150380.5224	167352.7083	152511.9752	124559.1395	185560.2939	89614.60063	97260.9393
TBC1D22A	TBC1 domain family member 22A [Homo sapiens]	68590.66356	0	0	97694.87069	123146.75	90501.94548	0	0	0
TBC1D23	TBC1 domain family member 23 isoform 1 [Homo sapiens]	107006.5922	168239.9968	71483.17234	168602.317	218050.3911	96467.38672	74934.12102	93508.62119	118426.916
TBC1D25	TBC1 domain family member 25 [Homo sapiens]	0	0	0	0	0	0	19175.99973	11677.53084	0
TBC1D2B	TBC1 domain family member 2B isoform a [Homo sapiens]	5841.799076	6122.59278	9604.790014	7095.943766	9956.094272	6252.948687	5466.124721	0	8777.556907
TBC1D4	TBC1 domain family member 4 [Homo sapiens]	95225.76908	131372.5382	115886.4207	103667.4418	118229.1293	147344.173	107254.3458	105703.7771	76712.91835
TBC1D5	TBC1 domain family member 5 isoform a [Homo sapiens]	45103.61316	47993.25439	43883.25263	0	51979.60526	60856.70526	74385.9193	62997.58179	66120.27368
TBC1D9B	TBC1 domain family member 9B isoform a [Homo sapiens]	26166.46191	24592.6667	40453.84891	0	0	38430.40526	0	9307.603893	34050.0205
TBCA	tubulin-specific chaperone A [Homo sapiens]	12928656.07	13642048.93	12477476.28	14707152.21	12749028.68	14532179.05	15076960.63	12252304.21	13381556.8
TBCB	tubulin-folding cofactor B [Homo sapiens]	2560106.496	2755703.507	3624674.543	2897761.27	3694962.479	3092467.497	2807779.613	2417244.981	3184598.406
TBCC	tubulin-specific chaperone C [Homo sapiens]	440421.1915	677391.0093	609899.9426	598501.7197	644157.7036	677699.0219	507574.4702	398663.1097	413489.6271
TBCD	tubulin-specific chaperone D [Homo sapiens]	124402.5337	121804.6479	186949.9447	142565.2257	178311.4799	138935.5377	148439.9147	108123.4336	124896.8701
TBCE	tubulin-specific chaperone E [Homo sapiens]	469424.3746	432162.6721	584280.032	452341.5434	550356.1456	480787.9671	427165.1192	349915.0563	374137.9686
TBK1	serine/threonine-protein kinase TBK1 [Homo sapiens]	128534.3904	171387.5415	176983.5116	155159.2798	819712.4058	894565.4898	124360.9722	109850.7986	105640.1747
TBL1X	F-box-like/WD repeat-containing protein TBL1X isoform b [Homo sapiens]	0	0	0	0	0	0	224410.0597	0	178591.2098
TBL1X	F-box-like/WD repeat-containing protein TBL1X isoform a [Homo sapiens]	0	0	0	0	0	0	224410.0597	0	178591.2098
TBL1XR1	F-box-like/WD repeat-containing protein TBL1XR1 [Homo sapiens]	405357.4173	910403.2618	1055200.807	762964.8596	1024010.025	942920.6434	809634.175	562062.4179	535874.4369
TBL2	transducin beta-like protein 2 precursor [Homo sapiens]	467413.477	387573.4487	672900.367	483340.8828	736840.3946	516829.6369	499515.2803	390830.362	534839.1059
TBL3	transducin beta-like protein 3 [Homo sapiens]	876887.9482	807010.2306	977506.0388	805896.3442	1086024.479	967371.2446	1263716.146	750494.9403	904325.3518
TBRG4	protein TBRG4 isoform 3 [Homo sapiens]	876230.8826	908879.4	1275356.388	1057922.317	1317108.397	1084725.62	976178.3483	833586.7661	1056725.587
TCEA1	transcription elongation factor A protein 1 isoform 1 [Homo sapiens]	1865302.923	1967869.148	2390849.41	2054335.623	2301374.634	2035636.142	1847759.129	1554782.329	2000238.272
TCEAL1	transcription elongation factor A protein-like 1 [Homo sapiens]	600114.9182	0	421437.1452	638880.7958	757063.6338	0	0	0	0
TCEAL4	transcription elongation factor A protein-like 4 [Homo sapiens]	144851.4206	147390.385	263549.8172	149911.281	201964.27	173525.7843	121751.5917	8553.562246	0
TCEB1	transcription elongation factor B polypeptide 1 isoform a [Homo sapiens]	19830137.72	17738342.55	22282486.07	22506565.51	24364424.89	22011067.73	21755896.56	17041253.24	23316723.32
TCEB2	transcription elongation factor B polypeptide 2 isoform b [Homo sapiens]	5852318.394	5829285.417	5877915.42	5342952.832	4057848.026	5502848.152	5008531.128	5047279.503	4933272.84
TCEB3	transcription elongation factor B polypeptide 3 [Homo sapiens]	95057.92525	63988.03218	78651.8	101647.4567	142348.1071	119205.9142	38524.04245	99126.28493	102705.7746
TCERG1	transcription elongation regulator 1 isoform 1 [Homo sapiens]	350993.34	458044.5328	405829.3846	376929.6078	469382.1204	403602.928	369179.4225	320834.2236	420599.6907
TCF20	transcription factor 20 isoform 1 [Homo sapiens]	20411.16429	16555.7384	4812.420886	22778.34669	29929.43162	20299.41394	31066.35461	18692.30827	11513.59072
TCF25	transcription factor 25 [Homo sapiens]	56971.24407	79396.56569	162598.2631	49336.9229	71473.8881	16771.3498	24267.60913	91689.51435	34438.35232
TCIRG1	V-type proton ATPase 116 kDa subunit a isoform 3 isoform a [Homo sapiens]	126986.7834	1158708.409	138724.0867	159687.5508	516549.1808	1731790.598	1084607.564	1026856.617	181440.3839
TCOF1	treacle protein isoform e [Homo sapiens]	38268.4743	43616.24914	61240.99876	295951.8744	18867.27424	30800.15528	293158.243	246410.6367	18162.09633
TCOF1	treacle protein isoform d [Homo sapiens]	38268.4743	43616.24914	61240.99876	295951.8744	18867.27424	30800.15528	293158.243	246410.6367	18162.09633

TCP1	T-complex protein 1 subunit alpha isoform a [Homo sapiens]	5507601.457	7012940.196	7303943.849	7192594.831	8095008.973	6893042.553	7059813.164	5603086.922	7522924.525
TCP11L1	T-complex protein 11-like protein 1 [Homo sapiens]	71909.2526	88107.29104	109854.1484	94123.10646	122659.8067	71934.74677	0	64395.89943	62634.15536
TDP1	tyrosyl-DNA phosphodiesterase 1 [Homo sapiens]	65391.50745	231453.0561	110204.2142	100395.4028	58955.9784	77085.77008	83810.18802	55553.66449	105031.1893
TDP2	tyrosyl-DNA phosphodiesterase 2 [Homo sapiens]	388206.5447	260426.5966	319984.4299	422176.8896	289314.1352	409297.4828	203959.1315	194356.8125	299432.748
TDRD3	tudor domain-containing protein 3 isoform 1 [Homo sapiens]	41946.83919	0	82755.97046	41200.28901	72871.81414	41134.60707	13484.27609	60813.36414	41088.76304
TDRD7	tudor domain-containing protein 7 [Homo sapiens]	16079.11138	44503.84861	16750.54593	41237.44243	36513.89748	42607.7618	15625.01067	30141.07528	22747.30085
TEAD2	transcriptional enhancer factor TEF-4 isoform 2 [Homo sapiens]	0	0	0	0	0	0	0	0	0
TECR	very-long-chain enoyl-CoA reductase [Homo sapiens]	3067750.629	1698644.373	2572963.18	1163957.305	2801238.216	1925713.31	1838581.623	1366134.437	1421322.229
TEFM	transcription elongation factor, mitochondrial [Homo sapiens]	110681.4276	99636.21975	196202.1824	0	213908.5876	186378.2757	0	153171.0789	109377.3248
TELO2	telomere length regulation protein TEL2 homolog [Homo sapiens]	153659.3801	195329.2754	248328.9129	251720.4068	222687.912	264483.4068	180286.43	144373.1866	157485.5658
TERF2	telomeric repeat-binding factor 2 [Homo sapiens]	47048.92096	50814.24124	42997.37351	24656.77284	42375.04382	49876.5957	0	0	0
TERF2IP	telomeric repeat-binding factor 2-interacting protein 1 [Homo sapiens]	326577.3953	895736.7263	289470.0178	365249.1607	325033.8213	375026.4353	341902.2564	265125.3216	684164.2618
TES	testin isoform 1 [Homo sapiens]	1291731.148	1636931.07	1116286.532	1294769.634	1178740.181	1532866.878	1368491.399	1393400.617	1445792.477
TEX10	testis-expressed sequence 10 protein isoform 1 [Homo sapiens]	355003.4725	551626.9881	450004.8266	338027.217	448031.5854	389625.8827	624502.6707	462599.2934	355921.1597
TEX264	testis-expressed sequence 264 protein isoform 1 precursor [Homo sapiens]	239189.4769	570830.8427	502876.5316	8563025.663	434811.427	0	0	0	0
TEX30	testis-expressed sequence 30 protein [Homo sapiens]	0	0	0	0	0	0	185750.2371	56561.52771	0
TFAM	transcription factor A, mitochondrial isoform 1 precursor [Homo sapiens]	1361530.679	1478133	2361658.797	1301558.607	1924932.24	1694238.623	1653085.816	1109414.141	1834963.928
TFAP2A	transcription factor AP-2-alpha isoform a [Homo sapiens]	530065.279	463648.4694	649999.7951	308778.0926	607170.4777	313681.9856	309952.7037	391868.957	414546.4344
TFAP4	transcription factor AP-4 [Homo sapiens]	0	0	0	0	0	0	0	179852.4286	119639.1306
TFB1M	dimethyladenosine transferase 1, mitochondrial [Homo sapiens]	109683.3722	159649.7688	212626.2898	136494.2896	229883.2635	213952.3773	140056.4718	167459.7193	203640.9299
TFB2M	dimethyladenosine transferase 2, mitochondrial [Homo sapiens]	466076.6609	535241.8861	488636.9438	694579.5751	529574.3257	829810.7341	842857.0151	673112.0995	780306.3405
TFCP2	alpha-globin transcription factor CP2 isoform 1 [Homo sapiens]	583859.9912	549427.3687	728633.689	533696.4567	961194.0032	594774.5521	603256.6481	996555.9342	763225.2613
TFDP1	transcription factor Dp-1 [Homo sapiens]	0	31942.98674	253571.6184	252360.7841	302961.4962	235983.1723	61494.27652	88264.86553	262464.9972
TFG	protein TFG isoform 1 [Homo sapiens]	6448241.904	8673090.078	10504294.83	7221844.681	10880816.21	10035327.2	6586296.759	7287081.035	4280243.656
TFIP11	tuftelin-interacting protein 11 [Homo sapiens]	122318.9456	140391.5089	128842.534	111896.7761	495453.4429	853657.1429	102471.9926	7266374.901	8615485.328
TFPT	TCF3 fusion partner [Homo sapiens]	84842.64211	0	153613	0	154058.3632	190598.4579	0	0	0
TFRC	transferrin receptor protein 1 [Homo sapiens]	6198447.514	7924505.057	9137543.014	8014071.819	8245444.085	8856151.138	9088685.262	7671944.032	9285187.404
TGFB1	transforming growth factor beta-1 precursor [Homo sapiens]	410908.7413	598282.8017	750454.2127	503629.7004	529792.6603	719955.5924	603282.0657	459525.2163	545808.398
TGFB1I1	transforming growth factor beta-1-induced transcript 1 protein isoform 1 [H	21239.50279	10697.69745	36439.34136	0	27768.09713	34032.35748	23796.17994	14714.04876	10231.33261
TGFBRAP1	transforming growth factor-beta receptor-associated protein 1 [Homo sapiens	0	0	0	0	0	0	23612.11525	16717.9911	19437.56314
TGM2	protein-glutamine gamma-glutamyltransferase 2 isoform a [Homo sapiens]	1179877.138	1193009.227	1501835.022	1201585.739	1488246.964	1299810.77	1331974.932	1105594.289	1461038.976
TGM3	protein-glutamine gamma-glutamyltransferase E [Homo sapiens]	207293.5111	391029.6366	459108.7479	17836.46008	235990.7224	235593.0914	26197.2072	18104.35977	0
TGS1	trimethylguanosine synthase [Homo sapiens]	44691.79912	63049.31211	59754.2949	88921.36091	76101.04708	62959.86188	69232.46997	31978.54058	53678.57315
THADA	thyroid adenoma-associated protein isoform a [Homo sapiens]	24640.84495	22730.79792	55624.55038	27069.09132	40723.11979	27667.40407	24436.77618	26315.59727	35102.00176
THEM4	acyl-coenzyme A thioesterase THEM4 [Homo sapiens]	581940.9455	469844.2871	614499.1567	484053.1931	485369.8523	520721.8874	685408.2429	486380.5446	2096629.994
THEM6	protein THEM6 precursor [Homo sapiens]	581943.0076	704213.654	952848.5676	863290.0887	467908.4946	955916.101	926884.9862	775075.466	716412.1524
THG1L	probable tRNA(His) guanylyltransferase [Homo sapiens]	191514.7581	454811.0865	575275.1815	520078.8936	610716.9556	534960.4769	527585.9627	614068.0323	478673.244
THNSL1	threonine synthase-like 1 [Homo sapiens]	72004.78418	27696.25611	30817.5459	0	0	76431.13281	84928.23047	66103.06738	0
THOC1	THO complex subunit 1 [Homo sapiens]	340349.7532	340464.2747	440839.5891	310799.3369	429541.0853	407691.812	390118.3612	371717.6794	416929.8368
THOC2	THO complex subunit 2 [Homo sapiens]	177965.7105	195950.4654	206210.4892	183007.6332	200165.2613	204266.0715	194668.7937	169503.6235	205914.8219
THOC3	THO complex subunit 3 [Homo sapiens]	1222293.632	1342702.572	1569347.202	1530582.608	1394206.228	1219610.212	1440094.24	1256541.773	1420812.454
THOC5	THO complex subunit 5 homolog [Homo sapiens]	343021.8267	317864.2661	434519.0043	372444.0089	444862.0577	346497.8642	318868.1896	165307.1107	308236.2938
THOC6	THO complex subunit 6 homolog isoform 1 [Homo sapiens]	660223.0327	813065.9653	954619.715	834099.523	1294390.794	776399.0649	750379.8875	463788.01	781162.6771
THOC7	THO complex subunit 7 homolog [Homo sapiens]	171461.6328	554494.6491	408767.6997	213870.6394	433997.1441	140351.9952	278421.0675	141101.6791	163993.2734
THOP1	thimet oligopeptidase [Homo sapiens]	1051416.814	1130308.943	1349797.346	1113748.219	1504595.873	1212572.447	1015606.075	962827.5072	1246534.083
THRAP3	thyroid hormone receptor-associated protein 3 [Homo sapiens]	741943.4942	828970.0153	967557.8097	855885.0911	993224.9821	876057.8137	827673.3916	728057.7393	876953.4213
THTPA	thiamine-triphosphatase isoform 1 [Homo sapiens]	303240.4611	124149.1125	430154.324	385778.6921	881982.4948	371683.9848	408031.3118	315887.9456	150373.1794
THUMPD1	THUMP domain-containing protein 1 [Homo sapiens]	1027897.418	928652.5323	1105103.691	753227.2646	1111571.755	749329.797	914181.6067	680678.0628	1160944.345
THUMPD3	THUMP domain-containing protein 3 [Homo sapiens]	366726.142	497145.5084	636725.4284	547626.034	632403.4975	589388.8873	541942.3442	456853.986	377584.9539
THYN1	thymocyte nuclear protein 1 isoform 1 [Homo sapiens]	396580.1019	311038.4004	297791.2579	147352.4274	205848.0548	144891.5716	133833.3969	422101.2938	391642.7938
TIA1	nucleolysin TIA-1 isoform p40 isoform 1 [Homo sapiens]	0	551890.9581	708320.0766	675325.973	0	607089.365	528635.5409	481107.2606	514625.9459
TIAL1	nucleolysin TIAR isoform 2 [Homo sapiens]	1319854.76	740085.6087	1627004.018	1235903.315	1579207.407	1726957.168	1045326.327	801571.5848	469377.188
TIMELESS	protein timeless homolog [Homo sapiens]	79466.67017	20996.05484	91400.09544	72742.46374	90989.38351	42883.01398	50571.38202	33559.2792	51822.70619
TIMM10	mitochondrial import inner membrane translocase subunit Tim10 [Homo sapien	1316554.597	0	1882867.539	1737028.733	1577403.883	2671871.889	2843939.789	1563855.519	597302.774
TIMM13	mitochondrial import inner membrane translocase subunit Tim13 [Homo sapien	15064209.4	15992136.95	13353791.77	16622332.75	17128599.64	17313471.25	15991436.07	13989596.43	16047206.54
TIMM17A	mitochondrial import inner membrane translocase subunit Tim17-A [Homo sapi	1319711.696	2390734.841	1495905.087	1316794.666	1682650.511	964958.6413	886279.7826	777936.3261	828395.4946
TIMM17B	mitochondrial import inner membrane translocase subunit Tim17-B isoform 1	631836.9633	813307	943390.8938	804420.6541	1468499.864	804696.4746	1199664.221	634530.8514	881905.3152
TIMM22	mitochondrial import inner membrane translocase subunit Tim22 [Homo sapien	0	0	0	0	0	0	0	0	0
TIMM23	mitochondrial import inner membrane translocase subunit Tim23 [Homo sapien	4226936.384	4252394.29	5027704.095	3670600.307	4510070.366	4288662.212	3568098.759	2573795.428	1846673.129
TIMM44	mitochondrial import inner membrane translocase subunit TIM44 [Homo sapien	837748.4167	612121.6614	1036330.805	642210.436	1144598.396	942204.589	949799.8636	812808.6702	952713.0702
TIMM50	mitochondrial import inner membrane translocase subunit TIM50 [Homo sapien	981517.2958	1483400.809	1130529.753	1438645.08	1582545.462	1273791.133	1546458.802	935933.4895	1517451.745
TIMM8A	mitochondrial import inner membrane translocase subunit Tim8 A isoform 1 [	5824280.467	5607280.125	7413543.3	6102449.553	7686031.806	6688474.842	5950149.786	6824107.689	7667527.131
TIMM8B	mitochondrial import inner membrane translocase subunit Tim8 B [Homo sapie	5339780.995	6794439.227	8227550.614	6862708.386	6973226.886	7235866.2	6082990.466	5097020.87	5891003.684
TIMM9	mitochondrial import inner membrane translocase subunit Tim9 [Homo sapiens	3437137.71	3329573.315	3167480.578	2241102.103	5178393.508	2529185.095	2952358.26	1663605.1	4153054.948
TIMMDC1	translocase of inner mitochondrial membrane domain-containing protein 1 pr	495986.6032	439061.7717	572893.1376	565369.9563	583070.8157	559112.682	453586.9892	239297.3954	847032.0553
TIPRL	TIP41-like protein isoform 1 [Homo sapiens]	2409061.773	2029317.886	3328724.083	1950191.459	3458816.839	2232016.606	2345266.587	2064460.371	2344272.884
TJAP1	tight junction-associated protein 1 isoform a [Homo sapiens]	49910.11547	59064.9535	94367.98206	55993.96637	110410.2904	69607.09166	0	44044.47618	32807.463
TJP1	tight junction protein ZO-1 isoform b [Homo sapiens]	100090.5064	123319.9366	36061.71308	19516.12244	41371.55252	127950.3633	124085.9273	102819.48	7893.868135
TJP1	tight junction protein ZO-1 isoform a [Homo sapiens]	100090.5064	123319.9366	36061.71308	19516.12244	41371.55252	127950.3633	124085.9273	102819.48	7893.868135
TJP2	tight junction protein ZO-2 isoform 3 [Homo sapiens]	109704.4969	12808.23426	180631.437	146620.5146	0	142031.7701	140545.8934	125713.7591	0
TK1	thymidine kinase, cytosolic [Homo sapiens]	1301073.577	1606832.736	2362174.217	1939135.205	2426526.474	1823803.781	1268289.644	951648.6478	2414333.068
TKT	transketolase isoform 2 [Homo sapiens]	30876700.92	32554279.35	38511758.14	32673100.26	38859852.37	34005280.97	33100610.16	28217183.65	35503195.9
TLCD1	TLC domain-containing protein 1 isoform 1 precursor [Homo sapiens]	275247.4303	199054.5797	0	294115.8043	0	274536.7971	208543.5955	114223.192	0
TLE3	transducin-like enhancer protein 3 isoform a [Homo sapiens]	33380.67695	82903.15465	0	240918.9506	13876.85237	340815.8519	38303.92901	111395.1461	190747.3772
TLK1	serine/threonine-protein kinase tousled-like 1 isoform 1 [Homo sapiens]	43123.59328	37097.23627	187486.3701	27495.37462	53259.52298	26970	48108.88611	0	43677.44919
TLL1	tolloid-like protein 1 isoform 1 precursor [Homo sapiens]	0	0	0	0	0	0	0	0	0
TLN1	talin-1 [Homo sapiens]	274870.2802	303565.2858	370616.3837	320310.4018	376240.2517	334032.8848	295485.9935	264842.2149	296005.9425
TM4SF1	transmembrane 4 L6 family member 1 [Homo sapiens]	158427.7822	89760.40204	173140.8678	0	420804.2223	120185.1824	125563.0235	155312.1869	0
TM7SF3	transmembrane 7 superfamily member 3 precursor [Homo sapiens]	9203.718754	34222.68288	29101.03059	35319.3169	22725.82658	36987.02993	0	0	0
TM9SF1	transmembrane 9 superfamily member 1 isoform a precursor [Homo sapiens]	241345.472	266859.6444	218984.3364	244272.3866	309129.3385	182022.4299	238136.6623	212292.2084	0
TM9SF2	transmembrane 9 superfamily member 2 precursor [Homo sapiens]	1298565.15	1362986.942	1818318.815	1122042.774	1250008.34	1184660.339	1326110.447	1014869.515	1295640.421
TM9SF3	transmembrane 9 superfamily member 3 precursor [Homo sapiens]	1085380.392	645349.379	1584512.328	885823.5222	1533174.839	720014.5818	1007887.466	652896.2852	1001526.656
TM9SF4	transmembrane 9 superfamily member 4 precursor [Homo sapiens]	378736.6002	303787.0892	164050.6366	373917.8511	325542.8922	278600.265	399388.9908	409026.9005	338012.4505
TMA16	translation machinery-associated protein 16 [Homo sapiens]	252026.1466	468980.7455	732688.7785	898308.8773	621161.8944	510863.4023	751241.7773	753183.0364	666918.9364
TMBIM6	bax inhibitor 1 isoform 2 [Homo sapiens]	0	1299507.831	1022895.165	1014180.018	989599.9983	1096964.338	945898.9718	791963.0176	0
TMCO1	transmembrane and coiled-coil domain-containing protein 1 isoform a [Homo s	2697614.577	2623306.349	2214577.667	3592971.493	2410586.421	2256256.023	3359460.824	2574971.306	3631607.845
TMCO7	transport and Golgi organization 6 homolog [Homo sapiens]	38104.41814	69601.52886	0	18996.46362	28905.95745	31279.01652	18726.30325	58014.73915	60823.12955
TMED1	transmembrane emp24 domain-containing protein 1 precursor [Homo sapiens]	947515.61	1109967.173	1165960.357	1377057.198	1665021.373	823532.5513	1196579.452	1298453.23	1228923.691
TMED10	transmembrane emp24 domain-containing protein 10 precursor [Homo sapiens]	5745550.706	6904533.424	7422187.847	6697096.223	7489155.293	7119171.275	7010048.814	5756953.609	6552975.575
TMED2	transmembrane emp24 domain-containing protein 2 precursor [Homo sapiens]	3086974.012	2902659.269	3821821.694	4095441.679	4522265.157	2552883.169	1787248.696	2634058.704	3146271.03
TMED4	transmembrane emp24 domain-containing protein 4 precursor [Homo sapiens]	1446659.287	1548423.491	2151269.39	2369278.743	3035128.537	2516525.743	2044865.314	2072488.801	2459432.801
TMED5	transmembrane emp24 domain-containing protein 5 isoform 1 precursor [Homo s	1783607.611	2420246.629	2320179.204	2333773.329	2455126.284	1723254.367	2446943.5	2141382.786	2739165.007
TMED7-TICAM2	TRAM adaptor with GOLD domain isoform 1 precursor [Homo sapiens]	894886.4049	1311301.633	1058155.564	1274723.472	1206830.149	1067533.671	1208959.124	918943.6625	1299110.072
TMED8	protein TMED8 [Homo sapiens]	0	0	0	0	0	0	0	145465.8333	161256.4329
TMED9	transmembrane emp24 domain-containing protein 9 precursor [Homo sapiens]	1668540.433	2158822.219	2960989.857	1972924.896	3259457.902	3106593.463	3172882.111	2584675.735	2050977.809
TMEM106B	transmembrane protein 106B [Homo sapiens]	1326171.012	1106345.216	534397.8354	776847.1163	714877.4414	491034.3642	524578.4259	984948.2222	685517.267
TMEM106C	transmembrane protein 106C isoform a [Homo sapiens]	530501.8788	775189.2765	573450.5455	672659.303	151220.8485	624084.0303	531330.447	584842.1364	352382.1061
TMEM109	transmembrane protein 109 precursor [Homo sapiens]	6881718.567	10134466.42	8280873.668	16995804.58	8284438.395	9802723.201	10019226.93	8107460.836	7074909.486
TMEM11	transmembrane protein 11, mitochondrial [Homo sapiens]	880897.5525	591686.5159	929512.7606	920104.123	916695.6806	769468.5846	942785.7063	796020.3298	1430518.379
TMEM115	transmembrane protein 115 [Homo sapiens]	86290.68681	0	384761.989	322290.6484	446460.9066	68092.31181	0	0	0
TMEM120A	transmembrane protein 120A [Homo sapiens]	0	0	0	0	0	0	0	109306.3934	288116.2664
TMEM126A	transmembrane protein 126A isoform 1 [Homo sapiens]	1810278.229	1516001.428	1704914.117	1510033.446	1478850.388	1441531.234	1530712.307	1256393.996	1691823.971
TMEM126B	transmembrane protein 126B isoform a [Homo sapiens]	387182.7089	392051.6627	702436.0813	416157.4543	660464.3786	347811.6973	372427.4792	0	362147.372
TMEM147	transmembrane protein 147 isoform 1 [Homo sapiens]	1476261.804	288730.2466	2065952.464	199282.1988	2022380.357	1430675.839	1143688.446	640519.7411	244208.125
TMEM14B	transmembrane protein 14B isoform a [Homo sapiens]	669622.1936	765339.3776	1099618.545	1038041.18	1635029.02	766295.42	573160.7688	689901.01	1069770.999
TMEM14C	transmembrane protein 14C [Homo sapiens]	15814776.38	15446187.73	25231332.7	16854319.5	26079826.78	18916883.27	17579843.7	13639025.71	16453050.6
TMEM160	transmembrane protein 160 precursor [Homo sapiens]	2139644.321	2121369.536	608454.2345	2190246.268	369623.8571	2278249.554	2243136.75	891732.8493	0
TMEM161A	transmembrane protein 161A isoform 1 precursor [Homo sapiens]	281656.9379	135140.5826	304021.4483	144275.4	114752.6927	326880.4914	210559.6147	45285.83793	55224.75561
TMEM164	transmembrane protein 164 isoform b [Homo sapiens]	65593.7916	157749.7771	0	18337.49066	0	51589.26705	110190.6649	234207.2322	0
TMEM165	transmembrane protein 165 precursor [Homo sapiens]	1697305.724	1753386.003	1917300.005	1601245.071	1974661.446	2494935.078	930605.2526	1475267.666	2226489.805
TMEM167A	protein kish-A precursor [Homo sapiens]	4890644.12	4362959.74	6813173.616	5977941.784	5543238	4821854.912	4929244.28	5114027.364	7268205.34
TMEM173	stimulator of interferon genes protein [Homo sapiens]	152130.6691	201961.8675	590074.9402	236216.3259	524702.84	128970.2196	401539.6239	215343.2832	216034.4509
TMEM189-UBE2V1	TMEM189-UBE2V1 fusion protein [Homo sapiens]	2315155.42	444579.65	2935835.117	1921005.549	6147643.117	259456.8063	1954803.296	2484856.803	3558107.838
TMEM192	transmembrane protein 192 [Homo sapiens]	281653.4655	0	330245.6024	3143526.161	471138.5977	1468810.461	963897.3276	766618.0992	0
TMEM2	transmembrane protein 2 isoform a [Homo sapiens]	12284.59685	33033.16098	34492.6929	59419.82575	31069.33114	27520.34388	41060.29996	33866.55759	62702.79933
TMEM201	transmembrane protein 201 isoform 1 [Homo sapiens]	197857.5493	112246.3572	233726.1935	171394.7653	144575.7606	110664.6611	77355.58488	119894.6174	132134.7465
TMEM205	transmembrane protein 205 [Homo sapiens]	2136237.667	1754275.745	2424897.706	2252223.165	3013726.686	2214955.527	2163972.908	1704730.05	2392123.176
TMEM208	transmembrane protein 208 [Homo sapiens]	0	2112121.528	3083404.409	1745720.693	3605202.086	1955987.617	1802039.091	1553055.96	0
TMEM209	transmembrane protein 209 [Homo sapiens]	251215.029	289874.338	361286.0409	274546.0875	329627.474	275150.1427	212509.9871	129949.9112	255669.9945
TMEM214	transmembrane protein 214 isoform 1 [Homo sapiens]	592180.7979	591860.2588	602964.8239	538924.016	747042.0527	787309.9683	626319.1337	471114.1354	840209.0774

TMEM223	transmembrane protein 223 [Homo sapiens]	416248.8109	397149.8622	504529.7821	645225.0962	849020.109	730099.2805	814624.109	464292.2949	571110.8074
TMEM230	transmembrane protein 230 isoform 1 [Homo sapiens]	0	0	0	0	0	0	253554.1452	688022.3589	0
TMEM237	transmembrane protein 237 isoform a [Homo sapiens]	0	0	334700.2904	314911.0462	406444.9558	163502.6145	332272.5241	94792.44423	295013.6577
TMEM245	transmembrane protein 245 [Homo sapiens]	0	53210.78788	68343.27805	110342.9589	155968.0422	105605.6523	61672.50866	78498.67532	94988.57792
TMEM256	transmembrane protein 256 precursor [Homo sapiens]	749458.8138	6532376.269	15097914.23	1657759.75	15466061.92	6751824.069	6452997.192	5033878.223	7860059.731
TMEM258	transmembrane protein 258 [Homo sapiens]	8860895.313	1547582.977	9111276.844	0	10156108.4	10967228.99	10562967.09	9310559.225	10444499.19
TMEM259	membralin isoform 1 [Homo sapiens]	185996.5078	213429.8781	227638.2691	195198.776	159630.0738	206799.8672	237496.5085	167446.6736	226490.9514
TMEM30A	cell cycle control protein 50A isoform 1 [Homo sapiens]	592735.1563	547907.8998	683034.8887	363431.0052	521203.9177	243335.808	375711.3012	84890.98148	473689.0748
TMEM33	transmembrane protein 33 [Homo sapiens]	2925738.156	3740669.356	3463317.552	3483534.239	3839263.569	3423744.126	4100313.945	3767568.184	4404271.223
TMEM41A	transmembrane protein 41A precursor [Homo sapiens]	0	0	0	0	0	0	598283.8253	281323.5042	532508.3014
TMEM43	transmembrane protein 43 [Homo sapiens]	2270716.728	2478633.46	2721760.642	2684629.993	2774978.534	2550429.689	2840661.51	2284107.411	2807069.776
TMEM48	nucleoporin NDC1 isoform 1 [Homo sapiens]	371517.6641	576569.7884	517881.3749	482151.8654	539292.4421	470537.1767	515824.4438	334058.0511	701645.2458
TMEM50A	transmembrane protein 50A [Homo sapiens]	180366.0987	331567.4803	285205.76	191308.4803	273092.2697	213351.8421	0	0	0
TMEM55B	transmembrane protein 55B isoform 1 [Homo sapiens]	297246.4789	0	395861.0908	344140.304	437838.5678	331316.8154	272453.6549	250653.8789	48009.81514
TMEM65	transmembrane protein 65 [Homo sapiens]	420009.0513	534991.7692	453223.859	604736.585	161186.9038	550696.5946	623995.621	500255.4103	398747.4615
TMEM70	transmembrane protein 70, mitochondrial isoform a [Homo sapiens]	1398570.172	1484316.654	1876007.94	2758794.569	1912055.966	2643806.42	2777606.586	1526910.391	2702118.356
TMEM87A	transmembrane protein 87A isoform 1 precursor [Homo sapiens]	132654.1953	87301.36124	161185.1986	136667.8747	131403.968	72297.89171	84408.62565	96351.22798	158588.742
TMEM9	transmembrane protein 9 precursor [Homo sapiens]	315731.8125	0	524549.3832	319025.4093	555623.9168	357450.535	309331.0828	167626.046	328284.1553
TMEM97	transmembrane protein 97 [Homo sapiens]	1192109.617	1586006.056	2718743.438	1670478.744	2733312.533	1805136.202	3505108.609	2686780.456	2676082.561
TMF1	TATA element modulatory factor [Homo sapiens]	38686.25846	58023.96104	63838.63712	47877.24254	53396.97859	70847.64006	56135.44957	19624.90414	47052.44136
TMOD3	tropomodulin-3 [Homo sapiens]	1593813.79	1804093.321	2111364.819	2308067.908	1820627.456	2058316.146	1930751.538	1534555.981	1875219.099
TMPO	thymopoietin isoform beta [Homo sapiens]	1475186.894	1772779.079	1982169.726	1844710.758	1977855.484	1900516.426	1724683.466	1583955.628	1674033.771
TMPO	thymopoietin isoform alpha [Homo sapiens]	1475186.894	1772779.079	1982169.726	1844710.758	1977855.484	1900516.426	1724683.466	1583955.628	1674033.771
TMPPE	transmembrane protein with metallophosphoesterase domain isoform 1 [Homo sa	0	0	0	0	0	0	153604.6396	104172.2877	327872.4612
TMPRSS13	transmembrane protease serine 13 isoform 1 [Homo sapiens]	0	0	0	0	0	0	44040.12073	51948.15585	0
TMSB10	thymosin beta-10 [Homo sapiens]	145891869.2	137841472.5	331671434.4	338537145.5	163056113.6	153495901.7	307891192.2	155223429.1	336291698.4
TMSB4X	thymosin beta-4 [Homo sapiens]	189968974.7	5109358.54	219332261.8	193202845.7	158832664.6	180058285	169121667.1	171941323.6	183725761.6
TMTC3	transmembrane and TPR repeat-containing protein 3 [Homo sapiens]	120334.1404	90633.45531	144892.8799	0	101511.5119	98287.0817	77112.72888	83908.92494	103714.8866
TMUB2	transmembrane and ubiquitin-like domain-containing protein 2 isoform b [Hor	0	0	0	0	0	0	0	0	0
TMX1	thioredoxin-related transmembrane protein 1 precursor [Homo sapiens]	4091553.114	5127431.196	5222472.326	5225713.727	5535178.182	4303439.295	5078839.557	4418476.06	4286639.99
TMX2	thioredoxin-related transmembrane protein 2 isoform 1 precursor [Homo sapie	979806.6892	1043689.707	1250807.173	1444598.931	1341125.215	1045486.813	918065.9333	814821.791	869649.3925
TMX3	protein disulfide-isomerase TMX3 precursor [Homo sapiens]	555371.8753	562447.463	583121.7924	626220.2807	515467.2158	619608.4048	697767.1584	513274.4865	455864.5337
TMX4	thioredoxin-related transmembrane protein 4 precursor [Homo sapiens]	303930.7813	0	259780.0534	454312.2774	352540.3802	391956.4167	381912.2682	325250.1563	0
TNFAIP2	tumor necrosis factor alpha-induced protein 2 [Homo sapiens]	208558.8747	164622.7212	236343.7667	199714.6852	184748.2509	214955.129	239457.3987	192424.4754	158701.1255
TNFAIP8	tumor necrosis factor alpha-induced protein 8 isoform a [Homo sapiens]	0	204788.7972	1089731.226	883527.1716	722593.8694	0	835749.6917	794239.107	319249.0686
TNFRSF10A	tumor necrosis factor receptor superfamily member 10A [Homo sapiens]	96237.63874	73166.26101	53502.57154	158625.7353	103181.3247	168832.9065	129682.6051	125759.288	178837.8586
TNIP2	TNFAIP3-interacting protein 2 isoform 1 [Homo sapiens]	72599.4623	0	60039.9574	96944.76657	59634.47794	68092.56069	70399.99049	66227.6526	78139.39461
TNKS1BP1	182 kDa tankyrase-1-binding protein [Homo sapiens]	126474.2065	118408.4917	160889.0884	142668.7611	184134.9354	141940.1825	119316.7074	165007.7455	170140.2996
TNPO1	transportin-1 isoform 2 [Homo sapiens]	5688566.029	2647506.186	3290902.277	2566349.769	3201300.256	2850413.257	2925196.941	2566085.232	3136423.232
TNPO2	transportin-2 isoform 2 [Homo sapiens]	209919.2424	350721.4878	397702.4587	299485.5578	524521.6739	280506.5997	314334.5696	247422.635	379064.1136
TNPO3	transportin-3 isoform 1 [Homo sapiens]	649530.1246	566113.1189	682016.1698	614966.4534	807543.5527	612626.5256	622929.2011	526937.1071	658515.1181
TNRC6B	trinucleotide repeat-containing gene 6B protein isoform 1 [Homo sapiens]	31583.31574	14418.8201	2045.336612	0	1904.154091	40243.60845	0	0	0
TNS1	tensin-1 [Homo sapiens]	0	32817.21096	14598.60166	69899.52444	16098.75144	6847.334998	6802.827403	0	10147.98336
TNS3	tensin-3 [Homo sapiens]	279636.6776	185544.7409	258479.9895	217851.7757	154385.4314	124887.9295	191464.8286	170539.1404	161727.2348
TOE1	target of EGR1 protein 1 [Homo sapiens]	407408.6834	434492.5402	562153.1382	371972.1125	553621.4768	468752.5593	325555.4259	331223.8598	430212.4115
TOLLIP	toll-interacting protein [Homo sapiens]	572744.8097	0	855924.8506	486625.477	870507.0762	575008.4248	647246.4943	621226.4949	337512.5417
TOM1	target of Myb protein 1 isoform 2 [Homo sapiens]	1230356.833	1063221.997	675900.6	678135.3966	713677.7466	590179.0816	529997.8105	853466.6561	1071217.091
TOM1L1	TOM1-like protein 1 [Homo sapiens]	189943.3231	289483.0229	287729.5585	281082.2277	298429.0082	238122.9037	273552.8874	173423.9182	274676.7061
TOM1L2	TOM1-like protein 2 isoform 3 [Homo sapiens]	103250.3333	79730.09513	126746.7463	105737.6094	136697.2365	121684.5207	112815.3514	85811.33738	44823.97071
TOMM20	mitochondrial import receptor subunit TOM20 homolog [Homo sapiens]	12497743.31	9652647.082	12572023.03	11929068.48	11417257.23	13265284.25	10800774.07	8522316.945	10807303.78
TOMM22	mitochondrial import receptor subunit TOM22 homolog [Homo sapiens]	26717108.3	26852007.18	35844252.55	28448493.2	36637592.93	29653946.63	28829591.9	21927932	25754898.53
TOMM34	mitochondrial import receptor subunit TOM34 [Homo sapiens]	3428351.688	4188366.015	4456225.751	4037053.224	4624482.693	3986599.948	3989493.209	3613571.177	4227160.908
TOMM40	mitochondrial import receptor subunit TOM40 homolog [Homo sapiens]	7420931.58	8762893.484	9819047.762	8507398.19	9629995.817	9037752.057	9227945.813	7415714.366	9686859.366
TOMM5	mitochondrial import receptor subunit TOM5 homolog isoform 3 [Homo sapiens]	4191591.474	3800434.021	4472814.674	4542877.01	4623274.462	4239656.872	4743758.659	4388812.646	3551270.882
TOMM6	mitochondrial import receptor subunit TOM6 homolog [Homo sapiens]	0	21253212.67	26710548.81	0	24933053.57	17583132.14	20293206.05	20274274.48	20849802.57
TOMM70A	mitochondrial import receptor subunit TOM70 [Homo sapiens]	1717311.343	1804288.922	2111322.32	1898542.173	2012654.095	1851908.106	1844479.061	1566545.449	2022898.268
TONSL	tonsoku-like protein [Homo sapiens]	32211.65261	27406.09053	0	17367.89843	0	9814.510715	0	0	0
TOP1	DNA topoisomerase 1 [Homo sapiens]	605711.2043	949459.6563	1154839.458	974366.87	1373626.56	1116267.914	1129000.367	938864.8831	1174289.94
TOP2A	DNA topoisomerase 2-alpha [Homo sapiens]	498745.2159	707000.5047	690778.1061	584113.3632	645390.0212	551331.0102	404203.6306	381475.1217	386357.5865
TOP2B	DNA topoisomerase 2-beta [Homo sapiens]	203522.885	249487.106	190695.0928	227959.8326	194304.5157	142288.3403	125285.363	158832.1773	236023.8299
TOP3A	DNA topoisomerase 3-alpha [Homo sapiens]	56388.18826	47439.03301	75897.75032	23284.3394	77137.15125	53161.4132	32453.94076	29004.44254	0
TOP3B	DNA topoisomerase 3-beta-1 [Homo sapiens]	20578.47983	25580.87791	51017.88395	12568.65589	20431.27471	14787.34557	20815.76453	0	21650.91497
TOPBP1	DNA topoisomerase 2-binding protein 1 [Homo sapiens]	0	34247.23924	16089.94923	34876.03981	73643.83635	36933.91632	49476.0657	60949.30465	34204.36489
TOR1A	torsin-1A precursor [Homo sapiens]	444036.7103	521988.5083	748279.4246	321936.6683	489981.5708	548285.9438	533859.2834	325836.8344	451698.4379
TOR1AIP1	torsin-1A-interacting protein 1 isoform 2 [Homo sapiens]	922235.7551	1132644.355	978014.993	915140.523	947958.3785	1033226.866	1035530.79	1064631.403	1476457.449
TOR1AIP2	torsin-1A-interacting protein 2 isoform b [Homo sapiens]	874564.726	810407.3452	1010361.722	903655.9129	1008718.825	977383.212	773457.1068	683195.4747	727372.3453
TOR1AIP2	interferon alpha responsive protein isoform a [Homo sapiens]	874564.726	810407.3452	1010361.722	903655.9129	1008718.825	977383.212	773457.1068	683195.4747	727372.3453
TOR1B	torsin-1B precursor [Homo sapiens]	151815.6657	261015.5213	224200.6443	8709.380986	216719.4628	85537.3822	19125.29277	173380.3085	0
TOR3A	torsin-3A precursor [Homo sapiens]	0	0	0	0	0	0	96117.65301	15302.78617	120038.2038
TOX	thymocyte selection-associated high mobility group box protein TOX [Homo sa	0	0	0	0	0	0	0	0	0
TOX4	TOX high mobility group box family member 4 [Homo sapiens]	348084.437	0	245696.2137	391824.4276	262023.0962	420238.6267	349975.7002	464975.8637	372455.8472
TP53BP1	tumor suppressor p53-binding protein 1 isoform 1 [Homo sapiens]	75852.3669	82053.77572	0	82750.56995	113441.2258	88215.71857	78077.25444	71770.44611	104272.4213
TP53BP2	apoptosis-stimulating of p53 protein 2 isoform 1 [Homo sapiens]	17371.9345	13003.46809	20926.64275	56702.73697	21175.11029	38976.94374	58829.18932	88528.93842	22005.01638
TP53I3	quinone oxidoreductase PIG3 isoform 1 [Homo sapiens]	755598.3023	581228.3039	558541.2247	585947.6369	663129.973	594824.3322	795936.8473	350127.3722	856028.714
TP53RK	TP53-regulating kinase [Homo sapiens]	772715.5561	328475.5264	750398.4182	430157.1639	765645.9159	425924.525	366366.5958	332119.4179	588647.9393
TPBG	trophoblast glycoprotein precursor [Homo sapiens]	200537.7022	215539.9961	83347.14453	162600.752	0	162353.3377	185767.8041	0	176766.0479
TPCN1	two pore calcium channel protein 1 isoform 1 [Homo sapiens]	0	0	0	0	0	0	34463.07825	0	40942.28469
TPD52	tumor protein D52 isoform 1 [Homo sapiens]	5403228.815	6720472.573	7825258.4	7171861.046	7744063.464	7427251.356	6979451.223	6726770.988	7168830.056
TPD52L1	tumor protein D53 isoform 1 [Homo sapiens]	1677155.519	1155497.419	1717316.1	3196640.572	2076653.272	1916237.48	1875274.558	2096595.441	822378.8301
TPD52L2	tumor protein D54 isoform a [Homo sapiens]	2235361.843	3524580.013	3614359.462	3371816.13	626808.4865	2391132.553	2432567.337	2283268.853	2860594.38
TPD52L2	tumor protein D54 isoform b [Homo sapiens]	2235361.843	3524580.013	3614359.462	3371816.13	626808.4865	2391132.553	2432567.337	2283268.853	2860594.38
TPI1	triosephosphate isomerase isoform 2 [Homo sapiens]	79137838.76	77500179.76	99870247	86769971.18	98753678.09	84628571.84	80460048.33	73974749.68	84228349.96
TPM1	tropomyosin alpha-1 chain isoform 6 [Homo sapiens]	7004573.07	7708688.448	10200416.91	6515825.677	9275376.455	7165691.14	6570896.652	3235239.936	7869831.818
TPM2	tropomyosin beta chain isoform 2 [Homo sapiens]	3129795.204	6064248.741	3340462.925	5605968.052	4101882.054	5882912.313	6052938.125	5054910.926	6534352.393
TPM3	tropomyosin alpha-3 chain isoform 2 [Homo sapiens]	17861066.7	16759429.32	873654.9828	17617592.78	25804480.07	18768592.03	17271852.83	14380547.31	18309812.97
TPM4	tropomyosin alpha-4 chain isoform 2 [Homo sapiens]	4533134.159	4482547.361	5869173.304	4662697.33	5958420.508	4733851.401	4751797.057	3192020.518	5095083.678
TPM4	tropomyosin alpha-4 chain isoform 1 [Homo sapiens]	4533134.159	4482547.361	5869173.304	4662697.33	5958420.508	4733851.401	4751797.057	3192020.518	5095083.678
TPMT	thiopurine S-methyltransferase [Homo sapiens]	969994.4798	673008.6523	1271778.632	956637.7098	1325867.037	917920.4897	1100414.985	566089.4604	735115.7744
TPP1	tripeptidyl-peptidase 1 preproprotein [Homo sapiens]	1174035.995	1330473.381	1522814.951	1364748.499	1682630.568	1308921.173	1153031.024	958234.5518	1263712.526
TPP2	tripeptidyl-peptidase 2 [Homo sapiens]	619497.1828	487944.6405	850849.1559	434746.7007	937307.0676	586847.1517	563904.023	493160.8202	525056.3425
TPR	nucleoprotein TPR [Homo sapiens]	308506.9792	297248.0642	397139.2461	1075533.482	405667.1477	342135.0431	311163.7909	1003655.667	307712.789
TPRKB	TP53RK-binding protein [Homo sapiens]	544286.5462	866465.5768	681994.068	721377.9494	688904.0658	591834.0642	802369.2042	611508.4243	475490.9885
TPT1	translationally-controlled tumor protein [Homo sapiens]	37342628.56	39554113.19	47673376.44	23590534.22	48448422.08	36060378.45	32109151.16	35673335.33	46450212.28
TPX2	targeting protein for Xklp2 [Homo sapiens]	102543.8842	1284015.515	1250540.213	127200.5052	1435742.441	74905.72631	56798.98783	54382.16446	1399841.516
TRA2A	transformer-2 protein homolog alpha [Homo sapiens]	1573181.067	1133872.574	0	0	861867.0123	2615.133178	0	0	0
TRA2B	transformer-2 protein homolog beta isoform 1 [Homo sapiens]	3569312.43	4541754.396	4387152.769	4334470.549	4143072.632	4641120.95	4442745.959	3395593.92	4381071.352
TRABD	traB domain-containing protein [Homo sapiens]	900295.0311	802914.6709	1176765.495	900054.1391	1367211.672	946840.5073	621200.2667	721128.4503	784259.4106
TRAF2	TNF receptor-associated factor 2 [Homo sapiens]	49242.87411	0	149605.5759	36694.16589	93691.45327	0	52798.35514	51554.88435	0
TRAF4	TNF receptor-associated factor 4 [Homo sapiens]	0	149840.2998	147640.2279	0	178451.2132	148098.0882	204593.7993	0	155757.1397
TRAFD1	TRAF-type zinc finger domain-containing protein 1 [Homo sapiens]	111010.211	90759.26186	119178.5884	122143.0605	119474.3868	104587.0829	135195.239	25349.75628	105025.0659
TRAM1	translocating chain-associated membrane protein 1 [Homo sapiens]	709497.8532	1056203.457	1468171.534	1377115.299	1481589.709	1263951.762	1401335.561	1123484.275	1928656.009
TRAP1	heat shock protein 75 kDa, mitochondrial isoform 1 precursor [Homo sapiens]	29178582.76	38328542.16	35596860.42	39360084.48	36280646.09	39312335.26	36503324.56	32368112.75	41734341.48
TRAPPC1	trafficking protein particle complex subunit 1 [Homo sapiens]	454226.9505	423126.4467	593074.0794	579417.7348	512223.1692	561627.2842	394328.3603	359680.0156	644113.0859
TRAPPC11	trafficking protein particle complex subunit 11 isoform a [Homo sapiens]	13285.5446	0	17891.02198	22141.89848	23186.80528	21761.94158	18579.67274	0	79448.24749
TRAPPC2	trafficking protein particle complex subunit 2 isoform 2 [Homo sapiens]	0	0	0	0	0	0	85694.99206	0	984194.137
TRAPPC2L	trafficking protein particle complex subunit 2-like protein [Homo sapiens]	794688.0741	984830.4444	431714.0904	1041234.593	476591.3287	1025984.657	0	0	0
TRAPPC3	trafficking protein particle complex subunit 3 isoform 2 [Homo sapiens]	1439361.472	1685277.47	1651714.052	1711801.731	1746423.19	1781316.43	865017.8955	1354781.003	1764320.994
TRAPPC4	trafficking protein particle complex subunit 4 [Homo sapiens]	483626.4753	626958.6127	748937.9043	712754.6631	912736.1481	912268.4136	796584.4846	804497.3364	732545.0422
TRAPPC5	trafficking protein particle complex subunit 5 [Homo sapiens]	513379.8678	181423.1977	295042.1479	351374.843	499307.3372	285639.7035	483294.3634	770209.8231	755218.222
TRAPPC6B	trafficking protein particle complex subunit 6B isoform 1 [Homo sapiens]	428340.213	693927.9195	909832.8339	855144.398	413950.9557	1157077.056	1011458.197	396083.773	746283.6053
TRIAP1	TP53-regulated inhibitor of apoptosis 1 [Homo sapiens]	776906.2195	753518.2044	880577.2561	623903.5671	840321.9085	539803.7837	754224.5427	264195.7317	430860.0732
TRIM11	E3 ubiquitin-protein ligase TRIM11 [Homo sapiens]	68159.76622	84894.57838	162878.3865	68521.67568	164591.6649	114206.8766	77930.85811	60425.38378	0
TRIM16	tripartite motif-containing protein 16 [Homo sapiens]	694807.2029	692243.0804	908828.3364	758931.3364	951738.311	751626.0756	821222.5469	705587.9091	783590.7895
TRIM21	E3 ubiquitin-protein ligase TRIM21 [Homo sapiens]	63626.67154	137666.0532	142151.6762	105173.1436	198441.3112	79218.88963	0	108473.3431	173702.0376
TRIM24	transcription intermediary factor 1-alpha isoform a [Homo sapiens]	47880.93304	144230.2931	48335.75971	107653.4468	141483.8209	130135.0849	64554.37602	43527.90322	118021.3301
TRIM25	E3 ubiquitin/ISG15 ligase TRIM25 [Homo sapiens]	1375863.033	1440342.947	1806447.12	1565941.456	1882979.452	1574682.441	1519476.351	1308368.211	1425060.329
TRIM26	tripartite motif-containing protein 26 [Homo sapiens]	326436.2768	379186.2836	436452.2203	423916.4653	402921.9098	391809.7397	403327.0056	359020.3643	440030.7013
TRIM27	zinc finger protein RFP [Homo sapiens]	176017.2169	185698.768	232138.8721	199847.6277	246880.3706	197140.182	197899.0277	90515.84907	176460.4813
TRIM28	transcription intermediary factor 1-beta [Homo sapiens]	4679971.294	4801085.824	5845292.771	5149015.276	6029659.588	5698161.333	5448981.082	4845894.466	5624994.125
TRIM29	tripartite motif-containing protein 29 [Homo sapiens]	0	36853.19869	44270.86754	79433.69869	0	62111.18097	0	0	0

TRIM33	E3 ubiquitin-protein ligase TRIM33 isoform alpha [Homo sapiens]	71157.3227	56144.3464	34120.60933	123411.4362	32698.87875	128853.0416	95398.14315	81743.7063	66003.33625
TRIM33	E3 ubiquitin-protein ligase TRIM33 isoform beta [Homo sapiens]	71157.3227	56144.3464	34120.60933	123411.4362	32698.87875	128853.0416	95398.14315	81743.7063	66003.33625
TRIM38	tripartite motif-containing protein 38 [Homo sapiens]	0	0	0	0	0	0	400813.233	199818.3306	0
TRIM47	tripartite motif-containing protein 47 [Homo sapiens]	282245.0225	340245.2513	297285.467	322327.9538	406061.3074	411759.2204	333568.5339	290371.3667	354330.788
TRIM56	E3 ubiquitin-protein ligase TRIM56 [Homo sapiens]	0	21940.02778	0	8097.820707	23117.72559	6796.87437	31838.57597	25749.37458	39061.69633
TRIM65	tripartite motif-containing protein 65 isoform 1 [Homo sapiens]	154186.0774	163873.6316	190158.4327	168243.4656	163622.2558	168842.6552	0	0	0
TRIO	triple functional domain protein [Homo sapiens]	17403.66997	8860.828619	17057.68702	19722.53886	24903.00829	18384.82594	42732.69921	9283.705737	12937.41686
TRIP10	cdc42-interacting protein 4 [Homo sapiens]	262079.9923	232613.9262	473603.3367	348616.5493	471817.5354	350606.4826	380355.8917	354450.6278	382867.2217
TRIP11	thyroid receptor-interacting protein 11 [Homo sapiens]	15211.49417	16665.47567	13628.30099	7039.039678	70000.85386	8063.316454	24435.43804	28113.80959	18113.20552
TRIP12	E3 ubiquitin-protein ligase TRIP12 [Homo sapiens]	126810.1232	127463.1732	144080.5765	143486.5317	102064.5713	80825.14747	133202.2484	118670.1828	111359.3061
TRIP13	pachytene checkpoint protein 2 homolog isoform 1 [Homo sapiens]	1837622.288	2260341.673	2390147.701	2275387.854	2460561.482	2299103.243	2296224.799	2064568.915	2484331.463
TRIP4	activating signal cointegrator 1 [Homo sapiens]	123664.9072	39578.06625	58793.6617	566981.7232	66918.2249	95560.98197	235276.9961	46906.83849	750018.727
TRIP6	thyroid receptor-interacting protein 6 [Homo sapiens]	1691326.353	1442754.028	2062624.937	1770419.406	1875990.499	1415071.537	1638363.292	1509070.699	1771397.546
TRMT1	tRNA (guanine(26)-N(2))-dimethyltransferase isoform 1 [Homo sapiens]	385487.2799	457605.4247	473060.4653	440640.9301	467013.8174	505875.1541	480600.783	373271.5566	441888.9266
TRMT10A	tRNA methyltransferase 10 homolog A [Homo sapiens]	103943.4833	139458.2	138603.2633	119664.88	178046.0133	121704.35	131174.4759	118112.0563	0
TRMT10C	mitochondrial ribonuclease P protein 1 precursor [Homo sapiens]	1358603.272	1451758.368	1822744.951	1579092.252	1799409.88	1635398.789	1584366.569	1282926.771	1491477.028
TRMT11	tRNA (guanine(10)-N2)-methyltransferase homolog [Homo sapiens]	175349.4667	184463.8625	202322.9931	180415.5444	297518.1104	186903.4787	132530.2341	250819.7306	72624.05694
TRMT112	tRNA methyltransferase 112 homolog [Homo sapiens]	13546787	14429054.41	16902816.97	14777895.91	17150335.41	15428181.62	79329757.65	65684194.3	92937322.33
TRMT1L	TRMT1-like protein isoform 1 [Homo sapiens]	152377.3293	176181.1505	212558.2896	161923.9092	221609.1237	194557.0789	162880.1632	97782.53677	204877.521
TRMT2A	tRNA (uracil-5-)-methyltransferase homolog A isoform a [Homo sapiens]	258624.0658	272958.4253	321326.0532	295343.4013	299033.444	267554.6837	284517.1681	248116.0648	258217.6961
TRMT5	tRNA (guanine(37)-N1)-methyltransferase [Homo sapiens]	175032.2946	181508.0099	244795.2237	151221.1571	180625.0341	181943.0205	172433.1196	136598.342	181759.094
TRMT6	tRNA (adenine(58)-N(1))-methyltransferase non-catalytic subunit TRM6 [Homo	375781.0737	184089.0404	471736.5435	246102.3301	475161.9088	269091.1061	237782.9006	153858.2236	202721.9654
TRMT61A	tRNA (adenine(58)-N(1))-methyltransferase catalytic subunit TRMT61A [Homo s	338025.7971	301673.5833	514986.0918	249992.8488	380290.992	296878.9208	171835.1439	265709.629	468177.353
TRMU	mitochondrial tRNA-specific 2-thiouridylase 1 [Homo sapiens]	61814.33	62085.01923	0	54029.28074	47026.64252	0	59483.97802	51131.25549	0
TRNT1	CCA tRNA nucleotidyltransferase 1, mitochondrial [Homo sapiens]	638836.3082	794410.488	639080.5582	855478.1418	789677.8716	879351.3389	900902.1231	651411.463	806336.6
TROVE2	60 kDa SS-A/Ro ribonucleoprotein isoform 2 [Homo sapiens]	1058741.495	1005417.548	1394323.164	1148497.507	1488164.848	1116413.067	1162763.342	857764.2825	1199362.779
TRPC4AP	short transient receptor potential channel 4-associated protein isoform a	0	25116.54553	22027.42343	18561.92964	31068.47061	0	0	0	0
TRPT1	tRNA 2'-phosphotransferase 1 isoform 3 [Homo sapiens]	208888.8415	247294.2565	239000.6254	97490.38918	334918.0651	0	0	0	0
TRRAP	transformation/transcription domain-associated protein isoform 1 [Homo sap	13906.15328	20923.00559	17444.97317	18469.46142	16306.42277	17275.07768	142212.9884	14387.85062	20314.92128
TRUB1	probable tRNA pseudouridine synthase 1 [Homo sapiens]	83297.49404	131870.2934	170927.637	58691.1113	142952.6199	155515.2757	181015.542	91584.93836	0
TSC2	tuberin isoform 1 [Homo sapiens]	0	0	10353.31587	19620.33821	12532.71629	22939.41507	0	0	0
TSC22D1	TSC22 domain family protein 1 isoform 1 [Homo sapiens]	626036.4262	1143000.804	1026307.228	1234826.851	1065414.574	1284271.127	1055251.016	863976.3492	1265281.332
TSC22D2	TSC22 domain family protein 2 [Homo sapiens]	255200.9841	218076.8095	209285.9187	194320.5536	264651.6116	245198.5258	228336.0397	33211.06151	261589.2624
TSC22D4	TSC22 domain family protein 4 [Homo sapiens]	0	0	181731.0216	95578.76079	182135.1781	119487.4591	0	0	0
TSEN15	tRNA-splicing endonuclease subunit Sen15 isoform 1 [Homo sapiens]	1856176.446	1972695.676	2343750.831	1734394.378	2543975.973	1814698.216	0	893642.7568	1700068.392
TSEN2	tRNA-splicing endonuclease subunit Sen2 isoform 1 [Homo sapiens]	72571.1091	105431.2091	169078.0148	65570.72377	132725.0035	143704.0132	178926.6224	55075.80316	117357.5781
TSEN34	tRNA-splicing endonuclease subunit Sen34 [Homo sapiens]	491355.264	375625.1674	580614.87	515951.528	619272.9865	534096.0625	466772.194	399882.292	398732.5157
TSEN54	tRNA-splicing endonuclease subunit Sen54 [Homo sapiens]	0	0	0	0	0	0	88223.17662	46904.30224	0
TSFM	elongation factor Ts, mitochondrial isoform 2 precursor [Homo sapiens]	2161438.476	2703581.679	2768320.727	2463225.625	2510035.659	2249291.766	2560073.855	2107565.007	3196071.895
TSG101	tumor susceptibility gene 101 protein [Homo sapiens]	382800.4632	297449.8354	454333.2435	537701.4261	455222.5437	554799.3261	312739.3819	326992.7726	311071.1248
TSN	translin isoform 1 [Homo sapiens]	2838456.751	3152840.925	6484628.204	3330240.344	6753916.651	3511824.568	3524174.348	2764850.972	6317884.127
TSNAX	translin-associated protein X [Homo sapiens]	1202362.046	1613147.139	2067130.354	1498185.185	1992106.185	1619544.262	1856098.138	1961063.047	2027000.99
TSPAN4	tetraspanin-4 isoform a [Homo sapiens]	740203.6979	0	1384140.714	802348.2527	1144747.849	0	1040060.969	642418.9219	0
TSPAN6	tetraspanin-6 [Homo sapiens]	617665.784	558355.821	901342.5401	708767.7716	921650.8642	601742.2948	470762.0715	258481.0054	782204.9352
TSPO	translocator protein [Homo sapiens]	0	0	0	0	0	0	54287.90255	0	625712.4835
TSPYL1	testis-specific Y-encoded-like protein 1 [Homo sapiens]	192170.7243	0	19978.75786	175961.2639	283364.6857	0	0	0	0
TSR1	pre-rRNA-processing protein TSR1 homolog [Homo sapiens]	900501.1365	1019726.879	1188738.717	1100170.487	1141125.676	1325843.68	1300488.918	906115.7116	1072577.169
TSR2	pre-rRNA-processing protein TSR2 homolog [Homo sapiens]	1566437.153	2821390.759	1633539.553	2073123.813	2965647.463	0	718108.7037	1702943.496	718316.1111
TSSC1	protein TSSC1 [Homo sapiens]	315287.0703	496887.1886	631110.8278	430579.2243	645530.7835	445324.5811	469630.9082	0	531668.7614
TSSC4	protein TSSC4 [Homo sapiens]	413799.6982	413583.0762	413891.0337	412432.6742	0	398623.9129	428415.3961	428771.3951	455632.7865
TST	thiosulfate sulfurtransferase [Homo sapiens]	742950.814	620939.3058	970772.8835	989474.4298	789478.7961	1255554.135	1383495.89	750411.2641	1000969.093
TSTA3	GDP-L-fucose synthase [Homo sapiens]	1974004.656	1669727.159	2295670.066	2300658.403	2467829.359	1714628.425	2230657.679	1807065.389	2321948.549
TTC1	tetratricopeptide repeat protein 1 [Homo sapiens]	1150098.413	1370481.018	1426327.527	1530500.468	1547019.421	1428691.124	1475214.574	1301132.59	1547923.825
TTC27	tetratricopeptide repeat protein 27 isoform 1 [Homo sapiens]	123112.6042	169046.3076	224776.3495	291930.2891	213932.2038	330462.9984	317652.1021	185515.0597	383598.5436
TTC28	tetratricopeptide repeat protein 28 [Homo sapiens]	13613.59027	0	4727.447121	3433.643939	24693.20545	4839.623371	3372.613967	25932.18364	0
TTC33	tetratricopeptide repeat protein 33 [Homo sapiens]	424043.9063	352817.8156	422477.5202	418931.4284	509464.5213	464572.1503	386862.1211	252123.964	410433.9008
TTC37	tetratricopeptide repeat protein 37 [Homo sapiens]	62070.72263	74237.74583	78074.93801	67941.89314	88586.85461	84815.38376	60749.35345	53308.35461	75619.48864
TTC38	tetratricopeptide repeat protein 38 [Homo sapiens]	416117.4594	412854.4805	423393.5779	423032.967	543914.0914	438545.7955	323564.2779	244332.3373	391020.5904
TTC39C	tetratricopeptide repeat protein 39C isoform 1 [Homo sapiens]	120595.2945	179667.2207	180094.7061	247109.7759	206336.8204	251865.857	121137.2013	136386.1335	0
TTC4	tetratricopeptide repeat protein 4 [Homo sapiens]	624279.9295	287721.7949	283994.2821	321653.6785	323152.8201	263570.8179	245606.3339	94466.05837	314884.358
TTC5	tetratricopeptide repeat protein 5 [Homo sapiens]	0	0	0	0	0	0	30704.83485	0	28902.35152
TTC7A	tetratricopeptide repeat protein 7A [Homo sapiens]	0	29901.47969	7233.062695	1585.522975	0	39106.6252	47714.82673	0	27363.95911
TTC9C	tetratricopeptide repeat protein 9C [Homo sapiens]	2001016.153	2191876.938	1033135.028	2268936.424	1526180.789	1663036.007	911235.4097	960116.1293	1407366.117
TTF2	transcription termination factor 2 [Homo sapiens]	15031.08564	17602.06782	21007.79152	17827.64751	19136.74901	18816.45347	4298.145422	8793.831111	14154.18806
TTI1	TELO2-interacting protein 1 homolog [Homo sapiens]	142702.995	103723.1306	152976.1431	140366.1676	147895.514	148172.6711	134502.9434	114884.9243	103111.6967
TTI2	TELO2-interacting protein 2 [Homo sapiens]	67388.65228	139835.5007	81537.41944	51773.9702	102936.0482	57460.26142	0	0	0
TTK	dual specificity protein kinase TTK isoform 1 [Homo sapiens]	82112.55229	28362.47851	93743.1508	58867.00799	72761.62728	67240.74235	65000.26129	57772.53582	50978.13521
TTLL12	tubulin--tyrosine ligase-like protein 12 [Homo sapiens]	1519659.243	1700767.735	2290048.941	1838374.694	2032591.591	2070501.259	1832519.744	1621583.534	2091334.386
TTN	titin isoform IC [Homo sapiens]	0	0	0	0	0	0	0	2371.66224	674.2008612
TTYH3	protein tweety homolog 3 [Homo sapiens]	50616.22421	239405.2983	60252.39187	262124.6793	52571.68452	194884.6981	281272.5965	166023.0688	352889.7537
TUBA1C	tubulin alpha-1C chain [Homo sapiens]	154592527.4	174015541.4	179527572.9	180657348.4	188230877	200705136.1	176373179	161501251.1	197649804.4
TUBA4A	tubulin alpha-4A chain isoform 1 [Homo sapiens]	70007593.83	81469891.83	94141981.59	83075612.89	91681990.23	92649284.28	90480145.86	74233864.21	100336767.2
TUBAL3	tubulin alpha chain-like 3 isoform 1 [Homo sapiens]	16999618.68	21598384.12	25477902.89	20486090.5	24268610.5	19617909.33	19915559.21	18548488.61	23743407.46
TUBB	tubulin beta chain [Homo sapiens]	155246374.1	158315017.6	207870249.7	163888996.7	218356580.9	188262487.4	174213336.5	155075178	179105905.6
TUBB2A	tubulin beta-2A chain [Homo sapiens]	1696114.604	2304724.216	0	2235176.211	0	2270453.254	2350480.16	2057538.69	2540590.79
TUBB3	tubulin beta-3 chain isoform 1 [Homo sapiens]	16373283.48	731186.9602	1025086.108	959321.2144	1152641.675	751828.3023	882456.4583	809774.1311	905002.7765
TUBB4A	tubulin beta-4A chain [Homo sapiens]	26294459.93	28987987.82	37329484.31	0	37074013.2	0	32195778.84	0	29791560.38
TUBB4B	tubulin beta-4B chain [Homo sapiens]	30603977.94	27361944.24	38403293.82	44439260.66	42250392.97	44963572.25	26828525.7	37632839.26	31341441.03
TUBB6	tubulin beta-6 chain [Homo sapiens]	4848194.236	4647411.656	5445618.332	14347514.19	6164461.147	14104624.06	14693339.62	12399790.45	5306968.268
TUBG1	tubulin gamma-1 chain [Homo sapiens]	1506528.386	1668034.806	1691305.607	1565486.539	1772068.034	1783328.379	1526672.131	1362661.507	1893612.801
TUBGCP2	gamma-tubulin complex component 2 isoform 1 [Homo sapiens]	105481.4497	146839.1965	147936.3493	148830.4047	154625.8226	172291.8275	182788.3487	126246.324	108441.0158
TUBGCP3	gamma-tubulin complex component 3 [Homo sapiens]	77772.86708	125206.3428	111959.9838	118907.2435	142566.6055	123274.4486	121641.5377	95523.95464	119047.5986
TUBGCP4	gamma-tubulin complex component 4 [Homo sapiens]	249091.5841	206817.4103	284854.0538	234962.2034	304901.6556	284183.1035	231309.327	67312.53626	203316.6991
TUBGCP5	gamma-tubulin complex component 5 isoform a [Homo sapiens]	0	6461.109543	14694.75459	12235.93898	24495.11155	13581.00935	0	7194.162052	10470.22014
TUFM	elongation factor Tu, mitochondrial precursor [Homo sapiens]	9848199.477	10063782.28	13010081.02	10231725.19	13043105.61	10633912.49	10893235.04	9280620.114	11327414.04
TUSC2	tumor suppressor candidate 2 [Homo sapiens]	0	0	0	0	0	0	638970.1973	0	539493.0389
TVP23C	Golgi apparatus membrane protein TVP23 homolog C isoform 1 [Homo sapiens]	156720.375	0	134976.6606	159024.5138	186550.2948	0	0	0	0
TWF1	twinfilin-1 isoform 2 [Homo sapiens]	2862013.811	3489511.539	4194433.404	3411489.005	4377861.477	3318848.562	3172958.112	2784159.839	3682926.095
TWF2	twinfilin-2 [Homo sapiens]	1281635.22	1178000.983	1458148.81	855612.2685	1391152.252	857952.7644	1242852.044	1088769.181	1574324.17
TWISTNB	DNA-directed RNA polymerase I subunit RPA43 [Homo sapiens]	465268.9556	508275.1187	653825.059	263205.6989	549043.2756	585786.691	506388.3614	648025.9463	502451.8915
TXLNA	alpha-taxilin [Homo sapiens]	728577.007	729997.3472	837948.1723	779763.5446	966412.5723	801081.9262	775372.5908	705194.0993	862012.2694
TXLNG	gamma-taxilin isoform 1 [Homo sapiens]	352373.6003	450893.8675	380942.6627	335607.5459	401248.4336	395177.1149	424047.5981	379279.2981	438609.6317
TXN	thioredoxin isoform 1 [Homo sapiens]	149572732.3	170300507.6	162669519.7	167142249.9	171690598.1	175796292.4	170540181	143183057.4	183454721.3
TXN2	thioredoxin, mitochondrial precursor [Homo sapiens]	2224238.474	5398837.492	4158639.332	4725904.75	2929907.22	1935795.811	4282075.706	2441018.964	3135881.676
TXNDC12	thioredoxin domain-containing protein 12 precursor [Homo sapiens]	4731505.008	3103564.256	6751562.179	3747226.563	6510162.519	4411394.217	4234361.894	5137528.916	4290881.897
TXNDC15	thioredoxin domain-containing protein 15 precursor [Homo sapiens]	275997.8689	347233.9986	402382.1435	359375.3146	400184.5261	394999.5518	351102.3776	198587.5232	421663.6653
TXNDC17	thioredoxin domain-containing protein 17 [Homo sapiens]	18294054.25	23444929.67	22009479.82	23026867.08	27171048.63	23029087.8	23440480.55	18803856.33	24739586.29
TXNDC5	thioredoxin domain-containing protein 5 isoform 1 precursor [Homo sapiens]	5546639.647	5976483.795	7367961.882	5969877.719	7399912.752	6315091.236	6435774.492	5404814.69	6664883.176
TXNDC9	thioredoxin domain-containing protein 9 [Homo sapiens]	1002420.556	1042573.934	1178692.746	796253.3646	1186161.187	840346.805	908843.0197	740607.5155	640696.8864
TXNIP	thioredoxin-interacting protein [Homo sapiens]	0	0	0	0	0	0	0	0	0
TXNL1	thioredoxin-like protein 1 [Homo sapiens]	3358914.078	4923326.987	4670183.311	4516976.036	4807990.64	4658907.748	4776427.148	3931526.154	5154250.645
TXNL4A	thioredoxin-like protein 4A [Homo sapiens]	1182219.279	1786737.439	1563543.468	838108.5926	1601146.697	993102.0154	1073159.338	1286832.463	1634745.313
TXNRD1	thioredoxin reductase 1, cytoplasmic isoform 2 [Homo sapiens]	255033.8207	863847.4593	781656.1521	885685.7078	885164.2004	916298.5135	863869.2342	682022.244	869780.0585
TXNRD1	thioredoxin reductase 1, cytoplasmic isoform 3 [Homo sapiens]	255033.8207	863847.4593	781656.1521	885685.7078	885164.2004	916298.5135	863869.2342	682022.244	869780.0585
TXNRD2	thioredoxin reductase 2, mitochondrial precursor [Homo sapiens]	629791.3368	569489.7897	788229.867	607107.0908	799727.8649	622719.7632	721108.2595	473300.3349	305504.5781
TYMP	thymidine phosphorylase isoform 2 proprotein [Homo sapiens]	104621.5416	113358.6129	284007.5828	153382.2484	156257.7134	124018.4203	132467.0253	0	108660.3005
TYMS	thymidylate synthase [Homo sapiens]	2466205.75	2680283.287	3541093.929	2183584.171	3124095.116	3128625.679	1335470.918	1912465.56	2217077.932
TYSND1	peroxisomal leader peptide-processing protease isoform a [Homo sapiens]	501858.8393	218549.8651	420890.6113	226974.1512	372796.9098	205439.7786	173561.8891	206591.4469	274008.7694
TYW3	tRNA wybutosine-synthesizing protein 3 homolog isoform 1 [Homo sapiens]	0	0	0	0	0	0	0	0	0
U2AF1	splicing factor U2AF 35 kDa subunit isoform a [Homo sapiens]	5374376.093	6770350.691	5810754.301	6624897.702	2697781.66	6492037.265	5980722.942	5386644.406	440601.335
U2AF2	splicing factor U2AF 65 kDa subunit isoform a [Homo sapiens]	7323505.352	8738190.239	10332146.56	8779240.176	10620592.99	9047563.899	8497431.019	7542525.245	9149283.836
U2SURP	U2 snRNP-associated SURP motif-containing protein [Homo sapiens]	274397.7393	355937.1384	531194.4288	476948.1681	573533.1119	459837.6223	368102.374	369419.8573	381845.6687
UACA	uveal autoantigen with coiled-coil domains and ankyrin repeats isoform 1 [	133315.7348	130287.6902	159192.9507	423133.5784	162741.7123	394846.5765	433162.8495	87078.76035	150780.7362
UAP1	UDP-N-acetylhexosamine pyrophosphorylase [Homo sapiens]	775396.4779	867076.859	1752397.925	1199241.671	972269.6169	987500.8872	916975.4436	857607.7195	1322394.702
UAP1L1	UDP-N-acetylhexosamine pyrophosphorylase-like protein 1 [Homo sapiens]	0	0	0	0	0	0	64164.20602	56580.67245	67897.92537
UBA1	ubiquitin-like modifier-activating enzyme 1 [Homo sapiens]	7552643.318	8344009.676	10459241.35	8662027.568	10755390.08	9522886.244	8822637.622	7532000.298	8891381.188
UBA2	SUMO-activating enzyme subunit 2 [Homo sapiens]	2139192.915	2781531.163	3114365.146	2649688.369	3214592.011	2793731.908	2730733.362	2299200.033	2879098.228
UBA3	NEDD8-activating enzyme E1 catalytic subunit isoform 1 [Homo sapiens]	946809.6314	1026490.425	993330.3686	1097335.621	1152540.286	943605.0935	855055.7536	906020.8797	1289182.459
UBA5	ubiquitin-like modifier-activating enzyme 5 isoform 1 [Homo sapiens]	1354299.838	1413622.738	1987719.702	1255644.395	2024986.188	1477610.002	1266284.269	1134990.814	1520018.386
UBA6	ubiquitin-like modifier-activating enzyme 6 [Homo sapiens]	260010.1959	309955.0161	363531.2897	291366.9066	362739.0759	315681.2513	312253.8831	242107.348	304775.1513

UBAC1	ubiquitin-associated domain-containing protein 1 [Homo sapiens]	250759.7297	200327.1038	419769.2419	77252.31045	406647.0284	229422.0272	176253.7471	202229.4816	346795.681
UBAC2	ubiquitin-associated domain-containing protein 2 isoform 1 precursor [Homo	314567.2547	465028.5564	525679.3727	357944.7381	539300.8006	486295.1381	440210.4262	291037.5941	516187.0802
UBAP2	ubiquitin-associated protein 2 [Homo sapiens]	113104.603	114032.0583	128020.8757	112887.6292	63726.10714	135579.3013	96023.31828	135728.8309	230024.9309
UBAP2L	ubiquitin-associated protein 2-like isoform b [Homo sapiens]	2273884.957	2605932.165	3267560.274	2916823.4	3198587.445	2917252.991	2648412.507	2309185.381	2629118.573
UBE2A	ubiquitin-conjugating enzyme E2 A isoform 1 [Homo sapiens]	8177806.647	8054058.32	10632542.09	7610366.789	10066359.27	7243703.72	7143626.22	4065873.831	9024221.091
UBE2B	ubiquitin-conjugating enzyme E2 B [Homo sapiens]	576834.619	865481.4524	1022009.333	1126299.762	4040170.952	3750371.524	2999184.217	2818511.524	1005122.143
UBE2C	ubiquitin-conjugating enzyme E2 C isoform 1 [Homo sapiens]	3343439.833	3670380.961	3771522.859	3448912.506	2694307.817	3245167.936	2672040.4	2479786.52	2519955.48
UBE2D3	ubiquitin-conjugating enzyme E2 D3 isoform 3 [Homo sapiens]	3585587.957	23833612.87	9489994.613	5434510.087	17287569.59	4636433.891	5339032.424	1027424.043	816890.6576
UBE2E1	ubiquitin-conjugating enzyme E2 E1 isoform 1 [Homo sapiens]	483317.8204	1366896.48	2435000	0	2158630.48	0	0	1941265.56	2770022.227
UBE2E3	ubiquitin-conjugating enzyme E2 E3 [Homo sapiens]	804589.5686	1524027.006	1083414.62	1415922.897	969720.7571	3091016.649	3158632.262	1867824.949	1497651.08
UBE2G1	ubiquitin-conjugating enzyme E2 G1 [Homo sapiens]	2470431.36	2200312.009	4057919.816	1784806.551	3806724.812	3418181.956	2766547.115	2300923.491	2931656.701
UBE2G2	ubiquitin-conjugating enzyme E2 G2 isoform 1 [Homo sapiens]	1702101.656	3029637.99	2834372.756	2674231.042	2734019.213	1779024.437	2881440.26	1986620.358	3382413.308
UBE2H	ubiquitin-conjugating enzyme E2 H isoform 1 [Homo sapiens]	2147503.393	2491752.755	2538612.603	3545341.552	2428365.859	3109626.409	3890085.112	1787880.434	2098063.605
UBE2I	SUMO-conjugating enzyme UBC9 [Homo sapiens]	6647012.933	10055904.7	9936172.884	10070898.29	7751205.798	7388440.356	7182575.484	5561729.02	9883941.442
UBE2J1	ubiquitin-conjugating enzyme E2 J1 [Homo sapiens]	116974.7528	123982.8687	43929.65482	61568.49728	0	133503.9478	117784.6664	0	141619.482
UBE2K	ubiquitin-conjugating enzyme E2 K isoform 1 [Homo sapiens]	4742090.917	5198239.548	6553594.98	5865120.631	7164662.409	5428181.095	5355715.541	5023630.472	5443098.215
UBE2L3	ubiquitin-conjugating enzyme E2 L3 isoform 4 [Homo sapiens]	15979434.24	17119673.77	17904692.19	17842680.57	0	18810018.88	15771525.69	16865822.13	15683625.77
UBE2M	NEDD8-conjugating enzyme Ubc12 [Homo sapiens]	5968383.48	7585018.207	8275235.265	6851280.263	8106961.081	5837450.072	7471818.8	6150081.731	7575170.815
UBE2N	ubiquitin-conjugating enzyme E2 N [Homo sapiens]	19701762.13	17861482.29	27398587.37	22874922.19	27255851.71	24483389.89	18568623.65	20609220.65	23997930.92
UBE2O	ubiquitin-conjugating enzyme E2 O [Homo sapiens]	935080.725	479947.0761	402155.0118	345306.7755	748502.7098	347895.3155	352557.2418	273340.8636	366114.4841
UBE2Q1	ubiquitin-conjugating enzyme E2 Q1 [Homo sapiens]	641092.9411	876751.9553	838225.0411	923267.3546	785411.5298	950857.0504	890057.6319	725544.5929	532729.2346
UBE2R2	ubiquitin-conjugating enzyme E2 R2 [Homo sapiens]	977573.0612	895235.2184	1231879.19	833113.5479	947556.9868	554104.6256	341547.1896	797487.0274	249255.6575
UBE2S	ubiquitin-conjugating enzyme E2 S [Homo sapiens]	1045884.61	1031153.946	1226696.212	1134966.456	1120261.654	1133969.372	1021764.342	742473.5908	929659.481
UBE2T	ubiquitin-conjugating enzyme E2 T [Homo sapiens]	2959600.014	2957722.967	3969926.07	2359341.903	4169898.576	3011605.597	2997732.733	2975389.081	2875529.087
UBE2V2	ubiquitin-conjugating enzyme E2 variant 2 [Homo sapiens]	4842756.227	4923805.959	10956133.63	5393188.781	8753402.562	4548305.221	4596398.322	3600769.787	4957395.706
UBE2Z	ubiquitin-conjugating enzyme E2 Z [Homo sapiens]	1846813.02	2185423.937	2609602.573	1939540.838	1999844.914	2230845.224	2221244.791	1674993.357	2132101.486
UBE3A	ubiquitin-protein ligase E3A isoform 2 [Homo sapiens]	49639.76244	77776.58052	67163.98684	59618.7396	114958.6116	55975.77049	46557.58405	148689.9425	42021.83537
UBE3C	ubiquitin-protein ligase E3C [Homo sapiens]	94366.0606	95045.08766	84339.8074	91284.75605	118938.9179	78024.28935	90167.95545	73343.20174	88306.38242
UBE4A	ubiquitin conjugation factor E4 A isoform 1 [Homo sapiens]	202000.3136	190808.8895	229633.8009	257202.691	263069.6248	894593.5345	226261.1397	206839.302	155312.3724
UBE4B	ubiquitin conjugation factor E4 B isoform 1 [Homo sapiens]	1183543.009	205878.0406	1450399.557	1309539.843	1479963.221	1204763.6	1116120.333	177626.4107	222071.4302
UBFD1	ubiquitin domain-containing protein UBFD1 [Homo sapiens]	758261.8171	826396.6954	1050611.974	1022340.384	1231960.313	949902.6372	941309.7496	1238089.56	1375285.242
UBIAD1	ubiA prenyltransferase domain-containing protein 1 [Homo sapiens]	0	0	171126.236	292857.7374	341252.5393	297585.8596	0	0	0
UBL4A	ubiquitin-like protein 4A [Homo sapiens]	835714.8859	1150250.45	1594357.617	1176607.069	1365739.036	1378587.942	1367826.833	1194742.54	1084658.897
UBL5	ubiquitin-like protein 5 [Homo sapiens]	3371104.166	4724282.546	1321192.15	4420110.566	1439194.261	3595600.351	4336470.543	4080433.74	681925.7929
UBL7	ubiquitin-like protein 7 [Homo sapiens]	418895.1914	628553.4489	631031.3508	753508.914	891881.4734	608186.3305	539550.7629	408706.7591	631289.9242
UBLCP1	ubiquitin-like domain-containing CTD phosphatase 1 [Homo sapiens]	465759.4883	438580.6311	598959.4787	463429.8669	563001.1443	505750.4245	414048.8076	398983.5057	393107.7528
UBP1	upstream-binding protein 1 isoform LBP-1b [Homo sapiens]	158105.0055	64207.35088	243302.0128	189161.4488	291556.4561	244382.4678	0	0	0
UBQLN1	ubiquilin-1 isoform 1 [Homo sapiens]	3660150.841	3884424.729	4377585.451	4395412.633	4911679.19	4350824.106	3659727.72	2892577.176	3471312.718
UBQLN2	ubiquilin-2 [Homo sapiens]	1732953.554	2063973.197	2598724.196	1991907.454	2663268.887	1675159.494	1174939.732	1481081.309	1674670.032
UBQLN4	ubiquilin-4 [Homo sapiens]	1445165.814	1577966.107	983875.5508	1013318.008	2096026.911	1681684.904	829303.8242	799273.1144	907320.2674
UBR1	E3 ubiquitin-protein ligase UBR1 [Homo sapiens]	23206.32427	16007.2466	19264.31691	0	28027.24884	476168.9864	26688.28336	23897.14097	6820.565546
UBR2	E3 ubiquitin-protein ligase UBR2 isoform 1 [Homo sapiens]	108914.6021	89784.42841	150332.2568	89411.84263	145421.2576	85215.04586	82430.52381	64111.65824	302934.1282
UBR3	E3 ubiquitin-protein ligase UBR3 [Homo sapiens]	15434.18429	16650.73958	24649.50521	25982.91737	31343.9412	15973.32492	24030.34335	21899.36519	18213.23928
UBR4	E3 ubiquitin-protein ligase UBR4 [Homo sapiens]	119404.6989	92479.33945	175839.2611	114828.7384	145728.2399	109643.8312	99164.99224	96708.69271	117765.6759
UBR5	E3 ubiquitin-protein ligase UBR5 [Homo sapiens]	69333.92172	73601.58431	113863.9881	201051.5056	109746.9458	115869.0468	87748.96256	68553.53455	94152.16357
UBR7	putative E3 ubiquitin-protein ligase UBR7 [Homo sapiens]	286013.2101	230140.8555	431290.1783	244746.0737	188178.6405	249941.1718	217893.142	336022.4224	256895.8384
UBTF	nucleolar transcription factor 1 isoform a [Homo sapiens]	404413.3244	505629.9275	603262.2077	457506.6739	568562.4203	495182.0949	450811.1568	306734.0043	477074.3449
UBXN1	UBX domain-containing protein 1 [Homo sapiens]	1894451.909	2359466.646	2798267.11	2364228.132	2772943.168	2554104.625	2421149.403	1763265.216	2505109.482
UBXN4	UBX domain-containing protein 4 [Homo sapiens]	506130.9276	673396.0467	708840.4894	672026.7628	763373.705	531379.2543	693703.7362	254968.8664	759680.0291
UBXN6	UBX domain-containing protein 6 isoform 1 [Homo sapiens]	172205.3115	89506.6669	255561.6003	103922.9499	148050.9646	127847.2139	166130.5925	65334.53209	145289.5909
UBXN7	UBX domain-containing protein 7 [Homo sapiens]	679870.3598	749166.5644	901969.5897	857098.6379	740216.2379	897173.4437	806146.0057	741625.046	1433973.047
UBXN8	UBX domain-containing protein 8 [Homo sapiens]	151484.1755	137203.078	236465.5069	123318.3544	178223.9416	136312.6626	132500.7351	75522.30677	107515.281
UCHL3	ubiquitin carboxyl-terminal hydrolase isozyme L3 isoform 2 [Homo sapiens]	4237806.943	4323442.558	5208002.217	3913531.056	3214438.03	2431483.83	2372073.512	3682717.029	5084467.153
UCHL5	ubiquitin carboxyl-terminal hydrolase isozyme L5 isoform 2 [Homo sapiens]	177432.6758	419194.9821	2401872.521	1657572.242	1543625.072	2376095.971	349244.904	466113.0849	451983.3862
UCHL5	ubiquitin carboxyl-terminal hydrolase isozyme L5 isoform 1 [Homo sapiens]	177432.6758	419194.9821	2401872.521	1657572.242	1543625.072	2376095.971	349244.904	466113.0849	451983.3862
UCK2	uridine-cytidine kinase 2 [Homo sapiens]	3235416.315	3992698.08	5102280.528	3809574.058	4885322.787	4570140.994	4007510.172	3848486.999	4236695.678
UCKL1	uridine-cytidine kinase-like 1 isoform 1 [Homo sapiens]	46274.11824	56151.29471	11211.46126	58705.961	0	60671.2257	44954.36425	0	28430.57155
UEVLD	ubiquitin-conjugating enzyme E2 variant 3 isoform a [Homo sapiens]	39893.82298	128519.087	93944.62733	98910.73683	140637.4309	148654.9612	75277.49379	0	28256.26572
UFC1	ubiquitin-fold modifier-conjugating enzyme 1 [Homo sapiens]	2350317.574	2535359.255	4390804.788	2572738.732	4535298.59	1001782.313	1181840.731	2495882.615	2445896.205
UFD1L	ubiquitin fusion degradation protein 1 homolog isoform A [Homo sapiens]	2439045.136	2362055.731	3222610.572	2616187.557	3211998.174	2964068.213	2679953.982	2225512.335	3058907.666
UFL1	E3 UFM1-protein ligase 1 [Homo sapiens]	210422.5215	219839.7317	273171.4469	201989.4861	242724.2936	235153.0499	224970.4134	157417.9221	205034.8517
UFM1	ubiquitin-fold modifier 1 precursor [Homo sapiens]	21870846.93	20620189.29	26850881.48	13949869.12	27743543.1	24234855.84	22283185.15	12066191.58	23349565.67
UFSP2	ufm1-specific protease 2 [Homo sapiens]	68534.353	232570.8989	195884.4695	194211.615	300709.1578	0	159935.6871	213091.385	215684.2681
UGDH	UDP-glucose 6-dehydrogenase isoform 1 [Homo sapiens]	4391775.883	5164064.771	5867481.353	4973927.462	6272797.532	5348688.1	5128270.199	4283475.272	5425442.274
UGGT1	UDP-glucose:glycoprotein glucosyltransferase 1 precursor [Homo sapiens]	270785.5023	253202.3025	359221.958	520056.9792	354597.2578	269277.1144	272478.1619	439038.8007	229373.6425
UGGT2	UDP-glucose:glycoprotein glucosyltransferase 2 precursor [Homo sapiens]	12646.61782	0	15897.89452	0	19289.73736	10203.71438	0	0	0
UGP2	UTP--glucose-1-phosphate uridylyltransferase isoform b [Homo sapiens]	570109.243	525264.146	766514.1875	78447.15745	689785.4565	0	260820.729	502337.7	223472.0839
UGP2	UTP--glucose-1-phosphate uridylyltransferase isoform a [Homo sapiens]	570109.243	525264.146	766514.1875	78447.15745	689785.4565	0	260820.729	502337.7	223472.0839
UHRF1	E3 ubiquitin-protein ligase UHRF1 isoform 1 [Homo sapiens]	0	0	0	0	0	0	405277.3452	336259.3866	342686.8403
UHRF1	E3 ubiquitin-protein ligase UHRF1 isoform 2 [Homo sapiens]	0	0	0	0	0	0	405277.3452	336259.3866	342686.8403
UHRF1BP1	UHRF1-binding protein 1 [Homo sapiens]	60828.27132	47106.93841	47717.01439	60308.34726	63206.2194	53610.34639	66502.99674	0	45318.31606
UHRF2	E3 ubiquitin-protein ligase UHRF2 [Homo sapiens]	0	0	0	0	0	0	8608.062796	0	7157.784668
UIMC1	BRCA1-A complex subunit RAP80 [Homo sapiens]	18857.43006	0	33464.52073	0	65724.38891	38322.92697	68753.12265	54793.99745	0
ULK3	serine/threonine-protein kinase ULK3 [Homo sapiens]	58714.44828	0	149329.2512	0	221938.3547	31071.70813	18764.22445	22140.38147	48336.22906
UMPS	uridine 5'-monophosphate synthase [Homo sapiens]	1844459.101	1894645.961	2593466.402	2407156.777	2876518.854	2250835.685	2295272.751	1995138.922	2792783.931
UNC119	protein unc-119 homolog A isoform a [Homo sapiens]	0	0	0	0	0	0	200941.8736	186804.011	183841.511
UNC119B	protein unc-119 homolog B [Homo sapiens]	279235.7905	202074.6324	0	167408.6042	325991.1127	253908.3554	312578.6422	283219.2353	322334.9081
UNC13D	protein unc-13 homolog D [Homo sapiens]	200333.3784	270124.0674	477144.4932	247854.8503	495411.5945	326409.6413	208335.388	149299.0173	246344.3081
UNC45A	protein unc-45 homolog A isoform 2 [Homo sapiens]	0	750761.907	982443.897	0	918219.021	805453.384	689241.041	548998.8455	0
UNC45A	protein unc-45 homolog A isoform 3 [Homo sapiens]	0	750761.907	982443.897	0	918219.021	805453.384	689241.041	548998.8455	0
UNC93B1	protein unc-93 homolog B1 [Homo sapiens]	176102.1236	219091.3328	234970.5579	80287.24874	178178.2506	222404.8634	214164.8191	0	223257.9499
UNG	uracil-DNA glycosylase isoform UNG2 [Homo sapiens]	0	311375.3941	464834.8361	2602089.439	420039.4792	377481.2025	2478141.55	226111.6209	283089.0699
UPF1	regulator of nonsense transcripts 1 [Homo sapiens]	546288.7472	694758.8312	640303.3363	603356.4541	687303.7672	653712.5408	564360.3501	548237.9267	693943.5432
UPF2	regulator of nonsense transcripts 2 [Homo sapiens]	370120.5462	43256.94618	67870.01273	54647.43859	75031.85273	45825.11212	41393.12297	1541836.522	49640.28148
UPF3B	regulator of nonsense transcripts 3B isoform 1 [Homo sapiens]	90284.879	76377.33452	113964.6999	75419.69352	0	129906.9893	75572.30359	80135.14769	101808.4297
UPP1	uridine phosphorylase 1 [Homo sapiens]	0	0	0	0	0	0	362970.2455	0	1077401.404
UQCC	ubiquinol-cytochrome c reductase complex chaperone CBP3 homolog isoform a	106075.2439	0	0	98076.03385	151658.0134	85498.41189	0	0	0
UQCR10	cytochrome b-c1 complex subunit 9 isoform a [Homo sapiens]	7510505.865	12264832.37	12428795.57	11357142.23	13903058.37	11704670.57	12173118.72	8382400.669	7741222.096
UQCRB	cytochrome b-c1 complex subunit 7 isoform 1 [Homo sapiens]	0	0	833394.3675	1036708.515	875208.2555	870758.6655	0	1426997.866	2020791.08
UQCRB	cytochrome b-c1 complex subunit 7 isoform 3 [Homo sapiens]	0	0	833394.3675	1036708.515	875208.2555	870758.6655	0	1426997.866	2020791.08
UQCRC1	cytochrome b-c1 complex subunit 1, mitochondrial precursor [Homo sapiens]	3071923.208	4237307.1	4999729.046	4122482.514	5255685.693	3754729.9	4018256.123	2707320.675	4113587.623
UQCRC2	cytochrome b-c1 complex subunit 2, mitochondrial precursor [Homo sapiens]	3468504.396	4323334.079	5062909.331	3820610.974	5975398.326	4046564.981	4067658.652	2954542.723	4729135.682
UQCRFS1	cytochrome b-c1 complex subunit Rieske, mitochondrial [Homo sapiens]	3105343.47	3871593.352	3642581.055	3358953.948	3640659.949	3446475.694	3108594.658	2697141.162	3819017.023
UQCRH	cytochrome b-c1 complex subunit 6, mitochondrial [Homo sapiens]	9690258.931	13231500.93	6888592.566	14056738.91	5900675.857	3073061.043	7323553.526	13285928.69	12772114.03
UQCRQ	cytochrome b-c1 complex subunit 8 [Homo sapiens]	1267616.851	1201567.88	1220966.549	1586919.586	1904441.994	1214214.611	1314020.695	1133451.299	0
URB1	nucleolar pre-ribosomal-associated protein 1 [Homo sapiens]	61078.74519	94401.83519	83295.32241	55027.74529	91502.33809	57583.18588	63634.96865	34712.81919	72941.65094
URB2	unhealthy ribosome biogenesis protein 2 homolog [Homo sapiens]	59112.01979	38876.83083	143640.3546	56668.79746	152771.1135	125706.6288	104395.2954	61673.69295	92214.09875
URGCP	up-regulator of cell proliferation isoform 3 [Homo sapiens]	60917.24059	72856.4741	91136.88395	81575.41548	58417.08658	96549.77777	60634.40607	74623.82709	63098.72881
URI1	unconventional prefoldin RPB5 interactor 1 isoform a [Homo sapiens]	105540.7787	0	192907.8629	31384.33739	259761.6119	223932.6357	146059.6798	87080.39662	119719.5952
URM1	ubiquitin-related modifier 1 homolog isoform b [Homo sapiens]	3715096.424	4687861.132	4641314.622	4401284.18	4251210.168	4669434.178	3972588.159	3595677.707	7290260.976
UROD	uroporphyrinogen decarboxylase [Homo sapiens]	1124387.965	1532332.763	1527172.097	1484110.84	1436673.28	1600198.885	1499412.715	1149438.14	1702259.408
UROS	uroporphyrinogen-III synthase [Homo sapiens]	0	0	0	0	0	0	74514.72068	0	151837.2794
USE1	vesicle transport protein USE1 [Homo sapiens]	0	0	0	0	0	0	0	50999.20079	130723.6014
USF1	upstream stimulatory factor 1 isoform 1 [Homo sapiens]	145993.3103	0	0	197170.8333	647535.046	224953.1207	170810.9195	179255.2874	394738.6897
USMG5	up-regulated during skeletal muscle growth protein 5 [Homo sapiens]	25772893.03	31108872.26	31200043.12	28809698.85	31382758.3	27687263.3	28261534.31	21393162.58	48526721.88
USO1	general vesicular transport factor p115 [Homo sapiens]	495963.2681	567689.1114	781730.524	577599.9293	812389.8593	534525.8041	603719.0924	423925.417	550010.8341
USP1	ubiquitin carboxyl-terminal hydrolase 1 [Homo sapiens]	35273.78261	13607.03067	36781.65879	49942.29037	18334.82337	46653.20031	41853.35811	16255.41896	0
USP10	ubiquitin carboxyl-terminal hydrolase 10 isoform 2 [Homo sapiens]	479654.9621	0	674174.656	585117.7238	763691.0548	599434.2	0	494866.7859	537731.7681
USP11	ubiquitin carboxyl-terminal hydrolase 11 [Homo sapiens]	275528.5159	285799.8299	287093.5194	293012.956	318849.2216	328096.1542	263414.5713	220071.5292	388424.3886
USP13	ubiquitin carboxyl-terminal hydrolase 13 [Homo sapiens]	30100.05551	110379.9443	0	0	25349.2156	40668.52467	122952.5519	0	79712.5
USP14	ubiquitin carboxyl-terminal hydrolase 14 isoform a [Homo sapiens]	2054472.679	2275616.742	2155266.084	2348364.902	2321371.817	2336071.248	2372804.839	2055485.297	2344910.68
USP15	ubiquitin carboxyl-terminal hydrolase 15 isoform 1 [Homo sapiens]	258104.9829	293920.9809	318627.3202	297386.7497	376742.3817	322414.7567	287486.2234	284377.9384	320404.1581
USP16	ubiquitin carboxyl-terminal hydrolase 16 isoform a [Homo sapiens]	15784.07213	22073.44985	17894.72166	11441.28634	17650.6657	0	24559.85102	21665.38072	43874.07849
USP19	ubiquitin carboxyl-terminal hydrolase 19 isoform 1 [Homo sapiens]	93849.04304	82615.20818	169841.1605	98068.69905	247389.6095	106944.614	90523.94824	127272.3286	89655.75614
USP22	ubiquitin carboxyl-terminal hydrolase 22 [Homo sapiens]	33369.55729	62230.8288	74711.05599	51317.36914	49001.15299	55303.15042	54817.02214	0	186702.2856
USP24	ubiquitin carboxyl-terminal hydrolase 24 [Homo sapiens]	54752.6446	53559.18032	67648.98905	60268.15368	77037.8676	55036.13348	48666.78606	45404.72856	53420.33414
USP25	ubiquitin carboxyl-terminal hydrolase 25 [Homo sapiens]	29120.90435	33792.46645	21726.971	22951.35619	59604.21336	29197.59206	27575.16884	29613.94695	21478.61126
USP28	ubiquitin carboxyl-terminal hydrolase 28 [Homo sapiens]	45099.405	634629.2487	57377.85817	46326.15146	59284.11479	48003.68304	51355.21066	52518.94479	730502.5651
USP3	ubiquitin carboxyl-terminal hydrolase 3 isoform 1 [Homo sapiens]	68061.46635	71944.94172	110660.7587	92633.46875	120945.9709	108910.5743	78108.28026	104600.3112	51856.02669
USP34	ubiquitin carboxyl-terminal hydrolase 34 [Homo sapiens]	9706.906615	8426.39391	10699.91062	10241.09032	6803.78198	7566.271007	9718.286325	4033.942739	13741.22049

USP36	ubiquitin carboxyl-terminal hydrolase 36 [Homo sapiens]	19602.32378	23558.40799	51102.62173	21614.10545	52799.73706	31468.85188	23611.14879	0	32594.66205
USP39	U4/U6.U5 tri-snRNP-associated protein 2 isoform 1 [Homo sapiens]	832512.5518	980751.2114	1048913.194	995613.042	1151549.303	1104618.414	983166.0057	801398.5029	990522.0073
USP4	ubiquitin carboxyl-terminal hydrolase 4 isoform a [Homo sapiens]	22602.31772	39891.62608	0	40789.57242	32096.96291	0	49612.82997	9515.478026	0
USP47	ubiquitin carboxyl-terminal hydrolase 47 [Homo sapiens]	102758.0015	122879.3217	119841.7391	132351.5164	128941.4876	151596.3035	122563.5629	83160.22452	118219.6599
USP48	ubiquitin carboxyl-terminal hydrolase 48 isoform a [Homo sapiens]	303894.9139	74687.24559	102420.7453	82779.62481	109966.75	336273.4759	315388.2979	277650.3179	315527.4915
USP5	ubiquitin carboxyl-terminal hydrolase 5 isoform 2 [Homo sapiens]	1420016.77	1267152.971	1827609.908	1422926.897	1798571.012	1462362.727	1251810.945	1134660.694	1464970.915
USP7	ubiquitin carboxyl-terminal hydrolase 7 [Homo sapiens]	433518.4728	487786.1382	518747.4393	529510.6045	630708.5254	534177.0897	520837.7951	474557.6033	467988.0967
USP8	ubiquitin carboxyl-terminal hydrolase 8 [Homo sapiens]	135920.7223	152858.1332	149591.4752	156169.8907	113707.8427	97619.50595	163730.1946	115262.8741	80701.30204
USP9X	probable ubiquitin carboxyl-terminal hydrolase FAF-X isoform 3 [Homo sapien	139967.3686	149528.7087	197841.8313	150977.069	204501.7997	156942.9514	158121.1185	149106.373	171944.9072
UTP11L	probable U3 small nucleolar RNA-associated protein 11 [Homo sapiens]	0	0	0	0	0	0	43754.04217	0	111047.4797
UTP14A	U3 small nucleolar RNA-associated protein 14 homolog A isoform 1 [Homo sap	246037.9698	269578.6785	347776.2384	233461.2723	302071.5249	305404.4046	262524.7759	212338.762	246727.3799
UTP15	U3 small nucleolar RNA-associated protein 15 homolog [Homo sapiens]	659926.2951	725513.196	907741.4386	788840	963036.2323	855691.3381	804754.0359	644841.4327	782235.0502
UTP18	U3 small nucleolar RNA-associated protein 18 homolog [Homo sapiens]	1135164.807	1390340.077	1434577.904	535357.963	623938.0861	1284707.689	518326.3617	983532.267	1449382.102
UTP20	small subunit processome component 20 homolog [Homo sapiens]	28326.96936	43066.60752	37979.85671	49079.42066	38918.12292	37381.95907	33580.27475	28446.84002	47644.92993
UTP23	rRNA-processing protein UTP23 homolog [Homo sapiens]	0	0	0	0	0	0	226077.5286	0	276662.7818
UTP3	something about silencing protein 10 [Homo sapiens]	260602.2562	219830.1636	381898.9758	153352.5641	430689.7823	310748.1887	172991.9677	253117.2479	310476.4574
UTP6	U3 small nucleolar RNA-associated protein 6 homolog [Homo sapiens]	703044.7483	324319.3659	674503.0404	368807.8685	302053.4349	375141.9774	254898.671	205161.0439	239856.7417
UXT	protein UXT isoform 1 [Homo sapiens]	0	0	0	0	0	0	0	0	0
VAC14	protein VAC14 homolog [Homo sapiens]	100209.0626	98524.97835	149199.9605	69342.07132	147877.0783	116911.2615	72436.18018	93209.53143	63162.7452
VAMP2	vesicle-associated membrane protein 2 [Homo sapiens]	595475.9875	2903304.973	652688.7313	0	1161631.35	2407956.802	74364.17188	841252.9125	2082728.735
VAMP3	vesicle-associated membrane protein 3 [Homo sapiens]	2280125.928	3397556.77	3608039.283	7111136.575	3834412.995	3985470.615	7025064.875	6549201.77	3778385.918
VAMP7	vesicle-associated membrane protein 7 isoform 1 [Homo sapiens]	644538.3352	928135.1452	807961.734	879044.661	669000.8502	889837.7969	890898.3706	685118.9028	622819.3989
VAMP8	vesicle-associated membrane protein 8 [Homo sapiens]	2058119.311	1392916.293	2197255.752	2019518.715	3420177.166	1862867.735	1522745.533	1406503.336	676679.2614
VANGL1	vang-like protein 1 isoform 1 [Homo sapiens]	72190.5008	82405.63557	53673.48975	87940.04831	71274.95353	85073.38547	123905.801	0	28799.73601
VAPA	vesicle-associated membrane protein-associated protein A isoform 2 [Homo sa	5377408.235	1840056.741	1194267.197	2164736.953	5639269.277	6954878.071	5983960.759	0	6057000.725
VAPB	vesicle-associated membrane protein-associated protein B/C isoform 1 [Homo	2144810.645	2012956.086	1861743.329	1833675.599	2059512.928	1303119.646	1260391.046	827127.2997	2136751.994
VARS	valine--tRNA ligase [Homo sapiens]	2185606.275	1981133.138	2569564.589	2239731.214	2425430.143	2372822.604	2265454.721	2072258.816	2170669.976
VASP	vasodilator-stimulated phosphoprotein [Homo sapiens]	1041990.419	1441724.144	1464927.153	1559184.63	1548263.44	1184300.018	1346352.271	1234006.857	1735343.751
VAT1	synaptic vesicle membrane protein VAT-1 homolog [Homo sapiens]	3133621.245	3282217.846	5352755.556	3179479.372	5367699.925	4236292.353	3531180.53	2717564.348	3497137.989
VAV2	guanine nucleotide exchange factor VAV2 isoform 1 [Homo sapiens]	66512.60763	52748.97847	44290.81337	49010.03161	78005.86066	30880.88048	38756.18184	39911.04758	60068.36523
VBP1	prefoldin subunit 3 [Homo sapiens]	6054558.258	6125891.399	7263611.384	5852552.466	7518469.438	5399119.143	6532486.844	5960110.174	6444501.965
VCL	vinculin isoform meta-VCL [Homo sapiens]	1974834.583	1912864.514	2836728.141	1995571.793	2766987.113	2066695.992	2082398.365	1774598.704	2292620.413
VCP	transitional endoplasmic reticulum ATPase [Homo sapiens]	3863835.588	4449363.918	5029297.344	4337424.109	4805096.832	4339390.909	4622560.912	3990891.974	4967228.494
VCPIP1	deubiquitinating protein VCIP135 [Homo sapiens]	112201.0132	128970.8113	105806.0118	77401.56054	133623.924	105448.6552	92667.6427	78744.51577	124744.6868
VDAC1	voltage-dependent anion-selective channel protein 1 [Homo sapiens]	25361176.75	25857698.66	33078800.99	28865863.13	33923898.24	28631834.54	29712810.65	24611736.68	29753655.19
VDAC2	voltage-dependent anion-selective channel protein 2 isoform 1 [Homo sapiens	12788327.19	17920390.87	14260373.71	17502940.04	18267055.79	17346198.67	19143325.1	14239331.73	9784405.794
VDAC3	voltage-dependent anion-selective channel protein 3 isoform 1 [Homo sapiens	10150329.41	13164929.94	14424649.38	13851503.47	14265601.12	14567136.37	14507198.61	10726576.58	14275336.43
VEZF1	vascular endothelial zinc finger 1 [Homo sapiens]	0	0	0	0	0	0	78815.09182	0	93745.87676
VHL	von Hippel-Lindau disease tumor suppressor isoform 1 [Homo sapiens]	279363.7246	238910.7464	261664.529	320739.529	249786.8804	297235.942	193248.5055	140190.1449	0
VIM	vimentin [Homo sapiens]	29895288.46	29726140.27	37895847.62	30403334.68	38900513.89	32481862.95	30140068.5	25941780.61	30096380
VKORC1	vitamin K epoxide reductase complex subunit 1 isoform 1 precursor [Homo sap	986524.352	1193536.735	1327886.852	801633.7143	1509036.671	1299323.991	1436595.551	857364.7092	1616550.179
VKORC1L1	vitamin K epoxide reductase complex subunit 1-like protein 1 [Homo sapiens]	826969.0173	743732.375	837856.6375	1300175.797	1229169.641	1317391.351	686306.5147	1135089.401	928537.7833
VMA21	vacuolar ATPase assembly integral membrane protein VMA21 [Homo sapiens]	5431501.704	7665370.664	8299734.832	9053123.746	11515311.06	6076318.214	8834274.45	5253512.689	15668555.51
VMP1	vacuole membrane protein 1 [Homo sapiens]	373035.7696	427620.4576	644663.5893	492585.2634	520042.7835	503682.6172	0	0	0
VPRBP	protein VPRBP isoform 1 [Homo sapiens]	94574.63558	114803.8762	96117.90269	106648.264	113426.1429	143582.4714	106052.8184	58258.03129	134290.8447
VPS11	vacuolar protein sorting-associated protein 11 homolog [Homo sapiens]	38568.86014	18864.27348	28488.05295	0	27500.02438	36697.07551	11169.98784	18229.57635	21360.07002
VPS13A	vacuolar protein sorting-associated protein 13A isoform A [Homo sapiens]	0	18273.64417	15716.86041	10651.33856	18998.24308	16216.96886	15785.44118	12577.91349	21986.86108
VPS13C	vacuolar protein sorting-associated protein 13C isoform 2A [Homo sapiens]	23613.11257	22533.90314	23432.36196	28557.37662	26405.26132	24429.41472	17839.52298	13303.70787	18580.0638
VPS16	vacuolar protein sorting-associated protein 16 homolog isoform 1 [Homo sap	57581.6929	95484.43605	124655.7896	96441.4892	132855.6798	114157.8107	112988.1402	105135.3349	128075.4577
VPS18	vacuolar protein sorting-associated protein 18 homolog [Homo sapiens]	79641.7553	56257.69519	136083.4863	110604.7945	117737.643	126895.0852	98770.7829	110763.9802	62607.11306
VPS25	vacuolar protein-sorting-associated protein 25 [Homo sapiens]	1344287.697	1153429.475	368456.5608	1882612.9	478090.191	1767809.614	1564905.094	993888.4068	1346610.225
VPS26A	vacuolar protein sorting-associated protein 26A isoform 1 [Homo sapiens]	1918287.515	1871299.818	2686152.41	1984681.938	2677128.718	2054524.557	1969264.908	1278175.799	1691450.067
VPS26B	vacuolar protein sorting-associated protein 26B [Homo sapiens]	417315.756	482519.4835	460801.3791	619270.7296	253881.6275	486450.7631	250061.9232	342009.9006	460878.5358
VPS28	vacuolar protein sorting-associated protein 28 homolog isoform 1 [Homo sap	0	0	0	0	0	0	1081566.866	918724.8921	0
VPS29	vacuolar protein sorting-associated protein 29 isoform 2 [Homo sapiens]	2764516.318	0	2420346.384	2914705.307	0	2834266.175	2546271.513	2085562.672	0
VPS33A	vacuolar protein sorting-associated protein 33A [Homo sapiens]	182334.7907	64290.70928	192223.6562	89857.24164	92443.243	98386.4476	54288.61972	39530.73	41142.63512
VPS33B	vacuolar protein sorting-associated protein 33B [Homo sapiens]	557587.1399	756038.4461	721710.5173	545250.0247	826329.8469	836390.341	503283.7867	513529.1517	672358.6613
VPS35	vacuolar protein sorting-associated protein 35 [Homo sapiens]	877499.2463	1390216.286	1507204.787	1270851.267	1698740.534	1256679.737	1203808.733	993010.018	1366928.892
VPS36	vacuolar protein-sorting-associated protein 36 [Homo sapiens]	116684.1546	240478.4653	287028.6763	150933.6488	274508.061	170931.8996	151094.8549	126454.2728	112274.3378
VPS37A	vacuolar protein sorting-associated protein 37A isoform 1 [Homo sapiens]	255620.2689	26861.13261	136874.0317	126133.6928	145565.6138	123078.6832	85975.92277	66353.66071	132171.5677
VPS37B	vacuolar protein sorting-associated protein 37B [Homo sapiens]	185498.2872	332707.4096	270933.8265	0	263349.6876	0	0	0	0
VPS37C	vacuolar protein sorting-associated protein 37C [Homo sapiens]	260784.0536	325675.1269	297444.8546	273530.1773	308874.102	302461.4796	326345.7474	234090.9388	298433.509
VPS39	vam6/Vps39-like protein [Homo sapiens]	19149.44573	38533.33123	33215.73599	53660.59297	0	15183.78274	34800.36521	56776.10224	27155.42227
VPS45	vacuolar protein sorting-associated protein 45 [Homo sapiens]	203191.2572	88919.15066	160907.7843	174438.6433	274333.6811	192866.734	174553.9286	165941.3362	248683.1102
VPS4A	vacuolar protein sorting-associated protein 4A [Homo sapiens]	210162.8658	442001.4033	306627.2348	228505.2083	444222.5815	197314.0635	247399.8731	221009.342	358674.1414
VPS4B	vacuolar protein sorting-associated protein 4B [Homo sapiens]	410960.3202	577944.385	565019.258	511512.3606	627936.8606	683463.4487	507801.9515	271673.6478	538197.3404
VPS51	vacuolar protein sorting-associated protein 51 homolog [Homo sapiens]	154600.8233	729517.189	264991.746	172521.6511	229809.4205	743675.4385	157647.8173	130319.6497	136128.3637
VPS52	vacuolar protein sorting-associated protein 52 homolog [Homo sapiens]	176197.3798	13265.89287	92425.70788	0	127913.2412	129929.9574	111528.7863	0	67556.51446
VPS53	vacuolar protein sorting-associated protein 53 homolog isoform 1 [Homo sap	113918.8726	98356.18991	141314.1147	111247.6526	152188.1553	122320.9738	75789.5538	59226.18323	119313.8301
VRK1	serine/threonine-protein kinase VRK1 [Homo sapiens]	397543.0165	661168.5181	490322.0677	668616.4011	522095.5721	543630.6699	526329.6841	397303.3892	490485.8968
VRK2	serine/threonine-protein kinase VRK2 isoform 1 [Homo sapiens]	72366.51605	62232.21312	116488.1366	0	161211.6268	0	52421.33902	0	79535.59698
VTA1	vacuolar protein sorting-associated protein VTA1 homolog [Homo sapiens]	2579268.592	2842800.224	2516640.568	2805046.179	2956523.072	3075252.877	3109919.733	2657636.087	2914882.195
VTI1B	vesicle transport through interaction with t-SNAREs homolog 1B [Homo sapien	293246.1572	153968.3363	471789.1766	443369.9725	308086.651	174477.3315	202903.214	194716.2449	339243.4128
VTN	vitronectin precursor [Homo sapiens]	1481494.931	0	2100509.273	1540878.577	1969345.086	1599091.545	2602937.527	2084792.243	2035645.497
VWA3B	von Willebrand factor A domain-containing protein 3B [Homo sapiens]	0	178581.7573	0	148199.712	164183.9774	174082.1712	0	0	0
VWA8	von Willebrand factor A domain-containing protein 8 isoform a precursor [Ho	0	0	0	0	0	0	17096.71789	0	10759.02556
VWA9	von Willebrand factor A domain-containing protein 9 isoform 4 [Homo sapiens	131003.5809	152451.1389	176792.0159	166474.5143	0	158595.1372	149626.2594	114640.9572	0
VWA9	von Willebrand factor A domain-containing protein 9 isoform 5 [Homo sapiens	131003.5809	152451.1389	176792.0159	166474.5143	0	158595.1372	149626.2594	114640.9572	0
WAC	WW domain-containing adapter protein with coiled-coil isoform 1 [Homo sapie	164020.4062	0	76920.40294	146958.9497	192757.9452	195879.7463	117923.9843	63203.27128	106254.5029
WAPAL	wings apart-like protein homolog [Homo sapiens]	82824.02058	103746.0437	93443.43905	122551.6335	135252.1233	130757.4053	121672.1874	106255.0937	104994.2478
WARS	tryptophan--tRNA ligase, cytoplasmic isoform a [Homo sapiens]	10008725.63	4071507.18	4705286.31	4146465.581	4863567.853	3717205.419	4081836.651	3397212.785	4208954.168
WARS2	tryptophan--tRNA ligase, mitochondrial isoform 1 precursor [Homo sapiens]	1117506.874	610537.2934	637717.8632	641286.2984	1335449.2	406299.958	1141413.924	491284.9707	1300641.013
WASF2	wiskott-Aldrich syndrome protein family member 2 isoform 1 [Homo sapiens]	418607.2516	375484.0957	501318.8015	429138.4618	527898.4788	502072.638	351219.1354	272995.3336	1064289.005
WASH1	WAS protein family homolog 1 [Homo sapiens]	62978.0866	68748.55705	0	236180.2811	0	82055.74835	314019.6467	198587.4283	27787.07467
WASH1	PREDICTED: uncharacterized protein LOC100287171 [Homo sapiens]	62978.0866	68748.55705	0	236180.2811	0	82055.74835	314019.6467	198587.4283	27787.07467
WASL	neural Wiskott-Aldrich syndrome protein [Homo sapiens]	265444.2432	299919.9591	472536.399	258471.2785	452534.9233	248255.3327	208597.246	249918.9683	289843.6137
WBP11	WW domain-binding protein 11 [Homo sapiens]	410402.5349	528133.4314	331781.3142	329063.9356	835687.9333	511208.8352	462737.2544	537413.3747	417626.0069
WBP2	WW domain-binding protein 2 [Homo sapiens]	1528627.796	1917986.973	1327399.451	1714855.413	1437964.597	1804466.929	1504528.45	664674.6424	1414810.946
WBP4	WW domain-binding protein 4 [Homo sapiens]	293256.6238	265642.8595	426144.0701	401783.2293	394315.8535	0	0	0	0
WBSCR16	Williams-Beuren syndrome chromosomal region 16 protein [Homo sapiens]	182344.4356	472697.5304	125004.8526	212187.7372	224895.8606	276064.1031	342936.5187	171475.7524	141896.1008
WBSCR22	uncharacterized methyltransferase WBSCR22 isoform 1 [Homo sapiens]	373242.4888	609603.8652	1182121.208	901715.9713	1298898.967	0	500350.2136	746377.4488	2462237.969
WDHD1	WD repeat and HMG-box DNA-binding protein 1 isoform 1 [Homo sapiens]	105554.6781	146039.3516	141647.6567	164137.2825	914301.6741	186779.9306	147401.3673	112253.0806	196653.1604
WDR1	WD repeat-containing protein 1 isoform 1 [Homo sapiens]	3373942.236	4065178.43	4755535.003	4164226.935	4892310.389	4045119.867	4293711.909	3441702.333	3939265.408
WDR11	WD repeat-containing protein 11 [Homo sapiens]	24127.26886	41144.53731	35487.77335	68212.71016	168765.1374	51815.16891	73596.94752	39143.13838	92725.74601
WDR12	ribosome biogenesis protein WDR12 [Homo sapiens]	1419815.951	1460591.107	1764167.875	1597532.529	1838066.407	1401857.827	1627482.015	1383966.511	1625898.268
WDR13	WD repeat-containing protein 13 isoform 1 [Homo sapiens]	53548.60914	0	173856.2405	108525.1053	196013.7029	91533.22843	84240.64279	67907.90482	111710.9715
WDR18	WD repeat-containing protein 18 [Homo sapiens]	1107166.338	1188805.979	964305.6856	1152041.077	1398077.034	959343.068	946328.6768	862747.024	724786.337
WDR26	WD repeat-containing protein 26 isoform a [Homo sapiens]	383018.7544	154360.4615	121242.9782	268507.9979	155194.0071	231545.5927	245188.1067	168254.0346	170700.6027
WDR3	WD repeat-containing protein 3 [Homo sapiens]	401020.8159	420520.9222	5570204.181	465110.9573	501426.4811	465750.6511	421145.3781	375460.1035	389393.603
WDR33	pre-mRNA 3' end processing protein WDR33 isoform 1 [Homo sapiens]	49608.22257	97877.56211	100879.46	110292.0844	50918.44923	104295.6939	57148.81458	67052.09217	74613.93188
WDR35	WD repeat-containing protein 35 isoform 1 [Homo sapiens]	20766.20792	51449.99222	0	18878.56944	0	36805.81944	43997.235	33046.96642	22727.93389
WDR36	WD repeat-containing protein 36 [Homo sapiens]	601554.0091	635375.4123	860628.7601	674403.2721	859815.7597	693591.0253	627591.8782	503724.9214	664094.1581
WDR4	tRNA (guanine-N(7)-)-methyltransferase subunit WDR4 isoform 1 [Homo sapiens	595871.6563	743509.7269	937074.5221	1072148.012	0	593980.0574	830604.4572	562963.9664	879914.9021
WDR41	WD repeat-containing protein 41 [Homo sapiens]	188223.0803	287179.182	300089.0363	206211.4253	316303.9428	288455.3192	227189.1441	163036.3737	272547.9027
WDR43	WD repeat-containing protein 43 [Homo sapiens]	785337.9212	823171.1278	730408.5054	675583.6772	667558.7386	684821.2079	504239.1531	752748.3199	912486.8299
WDR44	WD repeat-containing protein 44 isoform 1 [Homo sapiens]	209585.5365	176427.6843	215743.232	201144.868	260142.2064	145456.3539	141553.3329	116530.2071	190854.9217
WDR45	WD repeat domain phosphoinositide-interacting protein 4 isoform 1 [Homo sap	111781.1409	76718.40022	116459.1828	78346.79104	165165.6698	133410.1996	66225.19403	61626.58582	344368.5168
WDR45L	WD repeat domain phosphoinositide-interacting protein 3 [Homo sapiens]	0	0	0	0	0	0	0	533020.6637	205529.0597
WDR46	WD repeat-containing protein 46 isoform 1 [Homo sapiens]	377488.2837	448125.1613	483914.8379	428318.1805	457552.2732	345063.4643	415543.446	341709.7351	371614.3838
WDR48	WD repeat-containing protein 48 [Homo sapiens]	57062.56074	441751.0941	121504.3253	146670.3128	85205.23234	219757.6224	162956.5836	134014.2739	128259.6117
WDR5	WD repeat-containing protein 5 [Homo sapiens]	552508.0063	809323.7594	689794.9979	701683.1432	773824.5455	710209.7673	594170.7663	575095.5123	1091064.807
WDR55	WD repeat-containing protein 55 [Homo sapiens]	224492.4817	158393.225	301176.7181	221583.8643	0	297356.4643	137342.4911	182541.3621	253450.9512
WDR6	WD repeat-containing protein 6 [Homo sapiens]	126165.13	122868.4668	141893.5473	137536.7765	145293.2607	198808.8677	0	102229.361	179855.9327
WDR61	WD repeat-containing protein 61 [Homo sapiens]	4324463.256	4060701.998	5841693.463	4935404.563	5876157.023	5351424.026	4644523.483	3760810.27	4141782.72
WDR62	WD repeat-containing protein 62 isoform 1 [Homo sapiens]	45764.931	78554.51282	95870.55457	51764.56686	108428.1513	74819.43491	75806.88231	56131.93592	62448.89291
WDR66	WD repeat-containing protein 66 isoform 1 [Homo sapiens]	0	0	964060.0099	967849.7709	1059653.372	15141.25339	0	0	0
WDR7	WD repeat-containing protein 7 isoform 1 [Homo sapiens]	27284.66075	16831.44682	20829.40822	17410.0999	22379.80363	0	0	0	0
WDR70	WD repeat-containing protein 70 [Homo sapiens]	306367.5582	333288.7482	429191.3026	385938.3372	440691.9536	360450.2275	354520.3799	316120.8948	323288.283
WDR74	WD repeat-containing protein 74 [Homo sapiens]	1263220.428	695028.9628	796752.1397	384907.281	1343351.847	893529.5609	894148.241	1233573.394	1505829.133
WDR75	WD repeat-containing protein 75 [Homo sapiens]	363735.4596	350090.7906	393823.8074	472102.7401	387756.8596	392832.6084	410192.8704	385941.5205	316982.4433

WDR77	methylosome protein 50 [Homo sapiens]	4660894.353	5785320.696	7145670.737	5578102.814	6292140.545	5474508.272	5477653.714	4502242.199	5851669.55
WDR81	WD repeat-containing protein 81 isoform 1 [Homo sapiens]	8310.191189	33050.81476	52467.27236	29915.00467	63785.42274	41100.36892	0	0	0
WDR82	WD repeat-containing protein 82 [Homo sapiens]	758421.873	893915.5165	986686.6174	965531.3331	970853.9215	862680.2884	888542.8479	714780.0968	974575.214
WDR89	WD repeat-containing protein 89 [Homo sapiens]	0	0	0	0	0	0	72999.064	47414.414	0
WFS1	wolframin [Homo sapiens]	30228.32596	63191.40065	0	25520.17056	94609.55	31447.4752	44751.55365	27320.79597	62990.21458
WHSC1	histone-lysine N-methyltransferase NSD2 isoform 1 [Homo sapiens]	55521.83831	35620.53572	45984.54056	45818.27398	63982.70171	67599.43768	37570.75539	56411.66418	47079.06474
WHSC1L1	histone-lysine N-methyltransferase NSD3 isoform long [Homo sapiens]	23929.85025	24290.76878	25811.13493	24161.85027	30709.79971	26281.49812	23170.38622	0	23965.74497
WHSC2	negative elongation factor A [Homo sapiens]	313236.0417	336011.4325	393247.9487	381118.1219	433547.1195	343738.6267	320398.4489	312841.2255	345704.8162
WIBG	partner of Y14 and mago isoform 1 [Homo sapiens]	1905493.035	2156571.942	3086516.693	2691227.888	2728864.396	2755082.495	2701985.986	1748524.409	2570645.879
WIPF2	WAS/WASL-interacting protein family member 2 [Homo sapiens]	95835.8832	52908.14258	71067.76907	155847.3267	163181.7374	90139.01206	70950.47361	77583.04448	84261.62732
WIPI2	WD repeat domain phosphoinositide-interacting protein 2 isoform a [Homo sap	236078.2874	355676.815	285744.4647	0	182014.6423	103588.1276	57907.3069	102626.7586	349412.2549
WIZ	protein Wiz [Homo sapiens]	82629.77245	123856.0015	191890.1258	0	130022.8716	25229.21014	0	0	0
WLS	protein wntless homolog isoform 2 [Homo sapiens]	251399.8723	200503.8613	262340.359	275939.7109	109031.4634	293076.2729	292883.2229	175729.4349	253634.6578
WNK1	serine/threonine-protein kinase WNK1 isoform 4 [Homo sapiens]	96212.78543	99298.79238	131922.9702	114651.9219	131294.9426	115012.0492	117618.6095	106520.5541	135524.5108
WRAP53	telomerase Cajal body protein 1 [Homo sapiens]	870852.1625	445928.9577	974149.5547	742883.8968	439411.148	732924.875	553798.8691	523037.3955	447977.0084
WRNIP1	ATPase WRNIP1 isoform 1 [Homo sapiens]	126182.357	250111.5186	254501.5523	208099.1966	297250.974	148570.3012	170681.9535	190020.4757	300538.3267
WTAP	pre-mRNA-splicing regulator WTAP isoform 1 [Homo sapiens]	296797.8753	365169.2008	321642.8527	401519.4976	360888.3906	906535.6684	714974.5374	189496.2068	1019928.844
WWC1	protein KIBRA isoform 1 [Homo sapiens]	0	0	0	0	0	0	544108.913	480076.4348	0
XAB2	pre-mRNA-splicing factor SYF1 [Homo sapiens]	200335.4795	214995.7181	286706.4344	221568.6082	277511.3946	373802.4563	217994.0619	162049.9207	213822.2431
XIAP	E3 ubiquitin-protein ligase XIAP [Homo sapiens]	124839.1871	100874.1182	155008.2463	173349.4252	215103.7746	0	235183.8227	150847.2075	79492.95197
XPA	DNA repair protein complementing XP-A cells [Homo sapiens]	142772.415	45828.14037	58501.47593	53276.3791	71203.9416	56206.3125	46701.08094	50191.98361	48071.06148
XPC	DNA repair protein complementing XP-C cells isoform 1 [Homo sapiens]	46964.66975	41670.36201	61910.49062	63241.22921	27420.95395	61925.06965	18011.91109	8937.37861	35170.12023
XPNPEP1	xaa-Pro aminopeptidase 1 isoform 1 [Homo sapiens]	706661.8179	746364.3224	688960.1386	606919.148	678623.5553	835089.6272	803098.8211	534016.0366	633491.1581
XPNPEP3	probable Xaa-Pro aminopeptidase 3 isoform 1 [Homo sapiens]	5385389.872	5202300.883	4982053.105	5083147.109	4107700.997	284241.9887	5150716.058	14485632.98	6230993.2
XPO1	exportin-1 [Homo sapiens]	2564942.146	2478414.919	3322868.089	2793599.481	3273568.605	2654908.824	2494358.144	2387602.35	2577668.538
XPO4	exportin-4 [Homo sapiens]	109048.9341	214747.5611	182872.9271	122689.4527	261805	102446.7218	94598.10552	99239.87215	130222.5351
XPO5	exportin-5 [Homo sapiens]	948892.1049	945460.3068	1243805.794	1072618.367	1248755.983	1071268.002	1024894.572	851988.5316	1022201.652
XPO6	exportin-6 isoform 2 [Homo sapiens]	119064.4946	107617.522	79630.01186	139355.111	156829.1892	132739.1105	116409.0395	89873.23726	121548.0159
XPO7	exportin-7 [Homo sapiens]	235670.881	281365.5049	362590.9881	272577.6988	348603.8963	283529.3283	253762.9093	224352.3083	211756.6515
XPOT	exportin-T [Homo sapiens]	706044.1595	849553.1078	1124263.512	762168.4944	1157779.697	923471.5745	807629.5956	786214.0745	852565.7844
XPR1	xenotropic and polytropic retrovirus receptor 1 isoform 1 [Homo sapiens]	0	0	0	0	0	0	41828.88081	0	42445.76988
XRCC1	DNA repair protein XRCC1 [Homo sapiens]	292859.5256	155469.0804	367565.9872	318672.6689	395466.6605	295959.4238	277074.301	226766.6784	228375.0754
XRCC4	DNA repair protein XRCC4 isoform 2 [Homo sapiens]	184220.3129	755571.5007	265504.5468	1049300.228	205906.6978	277361.1043	988612.9748	1024942.62	1362968.742
XRCC5	X-ray repair cross-complementing protein 5 [Homo sapiens]	7249085.955	7576042.471	9763666.643	7509060.993	9614812.591	8339364.313	7287831.076	6202582.007	7503248.165
XRCC6	X-ray repair cross-complementing protein 6 [Homo sapiens]	6534264.121	8642304.475	9160518.129	8030593.839	8968670.589	8401047.254	8510339.689	7022801.939	9153897.264
XRN1	5'-3' exoribonuclease 1 isoform a [Homo sapiens]	50618.04023	42320.28454	63974.26845	51437.12002	75542.78036	163452.7598	151407.5295	48607.32202	51745.54732
XRN2	5'-3' exoribonuclease 2 [Homo sapiens]	456047.9071	563316.9377	657707.1579	430932.9901	658418.4558	568081.094	482807.8699	399341.5236	405496.3953
XXYLT1	xyloside xylosyltransferase 1 [Homo sapiens]	56584.03082	61670.04623	0	81504.00342	67684.59589	93993.66781	0	0	0
XYLB	xylulose kinase [Homo sapiens]	83352.81544	81536.80054	11647.4719	96877.28859	77128.31416	83777.59188	86621.27101	0	88968.63678
YAP1	yorkie homolog isoform 1 [Homo sapiens]	1153082.58	1310741.033	1532016.39	1369868.597	1624355.224	1007294.791	1254134.594	819269.439	1373654.532
YARS	tyrosine--tRNA ligase, cytoplasmic [Homo sapiens]	2920184.472	3528681.617	2948426.808	3544513.512	3311144.259	3644642.111	3475064.971	2878155.499	3514675.568
YARS2	tyrosine--tRNA ligase, mitochondrial precursor [Homo sapiens]	519007.3401	487337.8791	732092.4385	352421.6925	717996.4863	567382.0538	574430.2522	567671.5703	574379.8857
YBEY	putative ribonuclease isoform 1 [Homo sapiens]	0	0	0	0	0	0	0	130380.5357	824845.2103
YBX1	nuclease-sensitive element-binding protein 1 [Homo sapiens]	15083520.34	15193459.49	19892337.28	16251446.76	22010004.72	16602215.09	15408638.89	13962573.4	15013905.59
YBX3	Y-box-binding protein 3 isoform a [Homo sapiens]	2486200.234	2781369.386	3436198.105	3143991.014	2980287.336	3286895.021	2198814.297	1766080.695	2225742.277
YEATS4	YEATS domain-containing protein 4 [Homo sapiens]	273128.9123	0	0	185667.7061	488125.8038	247808.7873	156496.0768	0	247524.5099
YES1	tyrosine-protein kinase Yes [Homo sapiens]	260191.5001	181170.0312	372867.6023	234220.0832	262608.485	281175.5017	267940.8517	251080.8479	242298.339
YIF1A	protein YIF1A [Homo sapiens]	1533814.546	1556332.365	1156773.966	1143543.908	1787581.42	1147216.108	1219874.7	972660.273	1108508.254
YIPF2	protein YIPF2 [Homo sapiens]	0	0	0	0	0	0	0	86070.65524	396820.99
YIPF3	protein YIPF3 [Homo sapiens]	430676.5472	224026.4111	0	740871.1	312585.0944	0	236649.6167	0	1159017.689
YIPF5	protein YIPF5 isoform a [Homo sapiens]	1596914.973	3562695.159	953608.255	2805144.832	0	2693501.265	3075107.038	2810571.5	1928383.438
YIPF6	protein YIPF6 isoform A [Homo sapiens]	284835.5373	1482136.582	389901.0597	1356676.627	430147.209	1635425.813	324725.4851	1374539.463	904120.2799
YKT6	synaptobrevin homolog YKT6 [Homo sapiens]	1330935.73	1800421.326	1718181.557	1809574.122	1906098.337	1674199.868	1699171.471	1566181.19	2456286.205
YLPM1	YLP motif-containing protein 1 [Homo sapiens]	74173.84418	84917.56895	58759.0884	73659.32615	63011.0642	51308.74495	55128.04501	61597.11552	79158.1117
YME1L1	ATP-dependent zinc metalloprotease YME1L1 isoform 1 [Homo sapiens]	331937.1721	379064.3105	403352.8536	387766.9687	391080.9495	438508.5037	442821.196	328472.0285	410655.7533
YOD1	ubiquitin thioesterase OTU1 [Homo sapiens]	191071.0079	0	205404.4162	163191.5635	192280.3234	200219.7302	175895.9583	211442.0595	0
YRDC	yrdC domain-containing protein, mitochondrial precursor [Homo sapiens]	804422.7582	733440.1566	1089223.048	775906.4849	1078645.619	749098.7377	725851.0595	631272.8301	744328.134
YTHDC1	YTH domain-containing protein 1 isoform 1 [Homo sapiens]	104501.0264	208277.9593	284304.6138	130667.484	286595.3844	221762.454	204783.5646	36554.15494	225631.8729
YTHDC2	probable ATP-dependent RNA helicase YTHDC2 [Homo sapiens]	267833.9374	240997.0223	394486.0768	252803.7922	329237.4487	113725.1155	99135.24365	82652.15729	82089.90274
YTHDF1	YTH domain family protein 1 [Homo sapiens]	308962.6013	279657.6978	223430.8509	248571.9781	286462.0619	243954.7968	50505.12573	163017.0322	192218.5444
YTHDF2	YTH domain family protein 2 isoform 1 [Homo sapiens]	980416.3129	1019822.947	1262914.376	1108996.18	1676208.111	1111714.56	932342.2246	789031.1474	805497.8234
YTHDF3	YTH domain family protein 3 isoform b [Homo sapiens]	1042042.44	1204973.946	1139616.176	1245995.184	999753.7331	1213554.974	740585.7718	764499.4258	774096.0147
YWHAB	14-3-3 protein beta/alpha [Homo sapiens]	17986474.1	20972403.01	22956196.64	21099159.77	22757927.41	21077895.87	21462340.35	17317406.92	22187379
YWHAE	14-3-3 protein epsilon [Homo sapiens]	45248231.06	46337830.16	57188980.76	45638446.5	56009555.49	48187448.69	46656931.48	38665454.34	49818650.63
YWHAG	14-3-3 protein gamma [Homo sapiens]	18208132.05	13701156.63	24432204.75	18746116.48	23933115.13	20682341.97	18194643.57	16068128.26	16844614.69
YWHAH	14-3-3 protein eta [Homo sapiens]	5968424.81	5894987.372	4901041.377	5832961.043	7375975.817	6131240.507	4851020.622	5187412.067	6543305.649
YWHAQ	14-3-3 protein theta [Homo sapiens]	20643396.81	19974669.45	25765891.23	20502136.05	26241755.6	20922726.62	19176762.37	14999734.75	18778793.19
YWHAZ	14-3-3 protein zeta/delta [Homo sapiens]	38906729.3	44584295.18	50308810.64	42462019.66	55124757.7	45979347.59	43160379.49	35290296.21	46853614.8
YY1	transcriptional repressor protein YY1 [Homo sapiens]	561986.9435	609276.1276	775323.393	695245.4984	721392.2483	721296.2154	596198.3511	463624.9844	681738.1428
ZADH2	zinc-binding alcohol dehydrogenase domain-containing protein 2 [Homo sapien	0	0	0	0	0	0	276781.4213	248895.2441	24753.61122
ZAK	mitogen-activated protein kinase kinase kinase MLT isoform 1 [Homo sapiens]	222690.1366	174454.4605	290425.9744	155220.0465	307655.2942	74109.50435	28274.67255	61808.22655	64328.31625
ZAK	mitogen-activated protein kinase kinase kinase MLT isoform 2 [Homo sapiens]	222690.1366	174454.4605	290425.9744	155220.0465	307655.2942	74109.50435	28274.67255	61808.22655	64328.31625
ZBED1	zinc finger BED domain-containing protein 1 [Homo sapiens]	114561.6172	101190.2314	121589.2617	63240.85145	51893.89746	17765.47461	0	77449.61965	50359.10406
ZBTB7A	zinc finger and BTB domain-containing protein 7A [Homo sapiens]	89711.5509	138053.7844	89198.42754	398658.0389	0	0	212103.2725	194984.6093	158230.991
ZBTB7B	zinc finger and BTB domain-containing protein 7B isoform 2 [Homo sapiens]	0	0	0	0	0	0	95878.56164	0	77602.5411
ZBTB8OS	protein archease [Homo sapiens]	0	0	0	0	0	0	0	0	0
ZC3H11A	zinc finger CCCH domain-containing protein 11A [Homo sapiens]	136846.4066	170696.4703	213699.399	136603.6418	178814.2913	147178.4222	122267.8721	97191.88482	175712.5832
ZC3H13	zinc finger CCCH domain-containing protein 13 [Homo sapiens]	21571.90943	18328.35864	7796.741965	11153.61043	32389.00329	19017.33848	15398.22077	5101.849714	23186.44338
ZC3H14	zinc finger CCCH domain-containing protein 14 isoform 1 [Homo sapiens]	290141.9892	620437.3107	315821.5119	301919.1766	333359.1406	353178.2346	288395.4321	332698.2003	336195.7802
ZC3H15	zinc finger CCCH domain-containing protein 15 [Homo sapiens]	703977.7748	704368.6911	1000845.223	851950.4617	1066990.805	853534.7089	654676.7547	663703.7379	842695.385
ZC3H18	zinc finger CCCH domain-containing protein 18 [Homo sapiens]	140138.265	224242.1127	283626.3976	139339.5371	305201.0728	230514.378	213269.8506	59111.14422	150532.4306
ZC3H4	zinc finger CCCH domain-containing protein 4 [Homo sapiens]	127058.9156	151381.5498	148064.1037	30582.57155	129098.2364	133133.9234	114192.9557	130132.7994	143188.2369
ZC3H7A	zinc finger CCCH domain-containing protein 7A [Homo sapiens]	71685.55447	63431.60789	60558.57237	35973.0625	67931.53926	51867.68092	46373.33718	51110.37237	52405.87797
ZC3H7B	zinc finger CCCH domain-containing protein 7B [Homo sapiens]	49142.32173	65178.3672	63484.44975	63975.45802	61628.22817	66615.86323	57995.31743	47232.8855	43599.75573
ZC3H8	zinc finger CCCH domain-containing protein 8 [Homo sapiens]	390339.9457	540504.1414	507513.5436	470304.7385	426915.4309	421775.4211	0	0	0
ZC3HAV1	zinc finger CCCH-type antiviral protein 1 isoform 1 [Homo sapiens]	665458.6911	877053.0919	759068.1203	671421.4754	989197.7959	755378.9468	833119.2841	686616.7319	772015.3305
ZC3HAV1L	zinc finger CCCH-type antiviral protein 1-like [Homo sapiens]	809849.9193	475217.7421	682420.1882	493380.807	719768.4562	509264.34	408163.462	311748.1451	8577004.987
ZC3HC1	nuclear-interacting partner of ALK [Homo sapiens]	109595.6029	140727.4549	216982.585	152136.0721	230030.2582	162170.9642	150002.2029	0	178986.1134
ZCCHC10	zinc finger CCHC domain-containing protein 10 [Homo sapiens]	205711.4576	1070490.432	285018.428	186996.0593	0	314271.7627	0	249096.5508	351088.1695
ZCCHC11	terminal uridylyltransferase 4 isoform a [Homo sapiens]	8294.872109	12452.21029	10510.62507	8487.965505	11123.68764	9455.907335	15310.62521	0	8127.740562
ZCCHC8	zinc finger CCHC domain-containing protein 8 [Homo sapiens]	133322.7422	146344.8216	474672.6983	304102.8721	542981.3938	154819.6504	218035.6773	138243.7091	129963.2783
ZCRB1	zinc finger CCHC-type and RNA-binding motif-containing protein 1 [Homo sap:	202325.6358	721893.5428	410669.7449	433754.6811	362243.6837	246528.8069	280674.7015	71894.15816	584082.8061
ZDHHC20	probable palmitoyltransferase ZDHHC20 [Homo sapiens]	37392.37394	52898.02754	39590.81462	0	0	113209.2119	0	0	0
ZDHHC5	palmitoyltransferase ZDHHC5 [Homo sapiens]	36131.86719	27506.8447	35431.63542	37230.45147	23378.64773	41782.71212	0	0	0
ZER1	protein zer-1 homolog [Homo sapiens]	0	0	0	0	0	0	0	18304.14344	10549.47077
ZFAND1	AN1-type zinc finger protein 1 isoform a [Homo sapiens]	179935.4377	322725.7635	318901.3568	145750.8712	330955.9423	257138.5827	175973.0404	138606.7043	291643.1256
ZFAND6	AN1-type zinc finger protein 6 isoform a [Homo sapiens]	317991.0795	644323.0871	1176306.837	0	1247336.924	0	0	0	0
ZFC3H1	zinc finger C3H1 domain-containing protein [Homo sapiens]	1922.977052	23422.09968	3342.941415	11323.51885	176860.6983	14584.00381	8024.713621	0	11575.68145
ZFP36L2	zinc finger protein 36, C3H1 type-like 2 [Homo sapiens]	0	0	0	0	0	0	0	0	0
ZFP91	E3 ubiquitin-protein ligase ZFP91 isoform 1 [Homo sapiens]	150338.6064	134580.5181	234805.2065	143340.2074	266974.0905	171885.604	120544.7484	162156.556	175401.8127
ZFPL1	zinc finger protein-like 1 [Homo sapiens]	399657.7209	0	377104.8907	610206.4429	677611.4791	348944.4289	426733.8253	270816.4395	153322.5489
ZFR	zinc finger RNA-binding protein [Homo sapiens]	637735.4623	438756.3727	555830.2067	423347.1025	416368.6819	501854.535	509851.7543	304385.5516	646048.8181
ZFYVE1	zinc finger FYVE domain-containing protein 1 isoform 1 [Homo sapiens]	16556.02528	24959.43949	18542.41103	19732.87858	0	25646.63694	18687.71049	11681.53105	27639.10224
ZFYVE16	zinc finger FYVE domain-containing protein 16 [Homo sapiens]	72648.7707	83854.15024	70932.89669	61582.48185	98156.84296	66017.82588	66756.72641	71636.66867	94323.73601
ZFYVE19	zinc finger FYVE domain-containing protein 19 isoform 1 [Homo sapiens]	0	72034.99455	78559.96258	91320.47626	0	85986.61051	0	0	0
ZGPAT	zinc finger CCCH-type with G patch domain-containing protein isoform a [Hom	69750.54515	62351.7799	0	53138.8156	0	84679.683	61990.17585	41590.84584	41906.01017
ZGPAT	zinc finger CCCH-type with G patch domain-containing protein isoform c [Hom	69750.54515	62351.7799	0	53138.8156	0	84679.683	61990.17585	41590.84584	41906.01017
ZHX1	zinc fingers and homeoboxes protein 1 [Homo sapiens]	67035.73922	29681.82605	21188.94579	0	24082.96845	0	0	0	0
ZMAT2	zinc finger matrin-type protein 2 [Homo sapiens]	151877.1417	167140.9537	179837.1056	103344.0291	120057.4289	251249.6185	608877.7718	122595.9666	1086132.746
ZMPSTE24	CAAX prenyl protease 1 homolog [Homo sapiens]	1913588.959	1708574.035	2043356.228	1665090.87	2164123.604	1817156.946	2266363.013	1396967.147	1995224.721
ZMYM1	zinc finger MYM-type protein 1 [Homo sapiens]	0	14019.15941	0	15509.30824	15305.1	11268.97176	18628.42176	0	14061.06676
ZMYM2	zinc finger MYM-type protein 2 [Homo sapiens]	0	0	23828.62449	15554.58281	36734.34848	25437.11211	28473.25055	5708.26202	51331.88184
ZMYM3	zinc finger MYM-type protein 3 isoform 1 [Homo sapiens]	55126.41079	53117.71669	61512.09577	38863.82669	61731.28075	53322.42365	54590.36317	44376.52431	48239.82284
ZMYM4	zinc finger MYM-type protein 4 [Homo sapiens]	6264.921007	17458.81983	33350.96036	28934.42068	9928.014866	15646.54644	18131.57099	0	10275.64366
ZNF131	zinc finger protein 131 [Homo sapiens]	22923.72101	13348.35688	0	73995.34541	51168.86473	46533.30676	0	0	0
ZNF148	zinc finger protein 148 [Homo sapiens]	328030.8333	218876.2152	0	97840.44117	419349.3491	356354.6163	394957.0473	135711.4753	34495.86722
ZNF207	zinc finger protein 207 isoform c [Homo sapiens]	2644255.067	2924089.113	3540968.583	2669297.275	3326195.074	2827813.524	2672704.37	2196096.488	2767728.172
ZNF259	zinc finger protein ZPR1 [Homo sapiens]	805446.7717	840061.2217	1090919.999	709739.1967	1125655.23	906917.4895	941807.2053	806455.7934	746210.5776
ZNF277	zinc finger protein 277 [Homo sapiens]	0	0	0	0	0	0	0	0	0
ZNF280C	zinc finger protein 280C [Homo sapiens]	13679.57362	13371.57381	52386.80291	34276.88172	58580.46472	11810.14695	18685.54431	0	36790.54083

ZNF281	zinc finger protein 281 [Homo sapiens]	127911.6758	8653.87368	0	0	57821.20423	71710.98901	0	0	0
ZNF295	zinc finger and BTB domain-containing protein 21 isoform L [Homo sapiens]	27908.59546	20516.31236	0	11419.86244	25299.58105	14215.46747	0	31467.02169	22862.38699
ZNF326	DBIRD complex subunit ZNF326 isoform 1 [Homo sapiens]	994218.2074	1171117.155	1277742.103	1190923.514	1300857.504	1204833.703	1088000.276	936743.1173	1108489.81
ZNF330	zinc finger protein 330 [Homo sapiens]	0	99360.528	207607.03	133614.926	0	132037.829	69317.262	0	44562.96
ZNF384	zinc finger protein 384 isoform d [Homo sapiens]	70596.26286	0	220981.8014	174898.0005	224099.4353	175974.2636	0	0	0
ZNF428	zinc finger protein 428 [Homo sapiens]	709029.8567	1359227.026	1629577.874	0	1722540.526	1551292.458	983382.9488	594530.4975	1298210.549
ZNF512	zinc finger protein 512 isoform a [Homo sapiens]	46211.86177	36939.68021	0	140911.4541	0	127638.4167	0	0	0
ZNF512B	zinc finger protein 512B [Homo sapiens]	27259.51088	16348.39732	18824.35203	18517.70064	19567.59404	18759.04151	14903.69427	25370.33712	18801.56646
ZNF592	zinc finger protein 592 [Homo sapiens]	26427.12394	25674.53051	70822.70106	29107.88038	0	26527.37002	7081.961502	26787.60319	52585.53357
ZNF593	zinc finger protein 593 [Homo sapiens]	0	768819.4768	275161.4407	865543.1653	686208.7119	887912.6737	918258.1483	0	994464.0508
ZNF598	zinc finger protein 598 [Homo sapiens]	71751.21799	137405.8077	172210.6637	124309.3788	123367.4285	159745.9871	103541.8659	64162.65538	77308.29316
ZNF609	zinc finger protein 609 [Homo sapiens]	8990.455541	18626.1616	0	11333.94009	0	15818.57521	16992.49665	10740.04923	15446.54716
ZNF622	zinc finger protein 622 [Homo sapiens]	510049.0009	607994.5952	644136.733	564880.178	655446.689	631462.1767	573645.2181	442473.2317	587396.3379
ZNF638	zinc finger protein 638 isoform 1 [Homo sapiens]	49010.24481	46898.62451	158472.0802	129439.8912	180298.7069	157150.7831	111407.81	165757.0239	29799.65913
ZNF668	zinc finger protein 668 isoform b [Homo sapiens]	18401.89323	0	20042.51623	13811.45778	16288.22636	12655.65285	0	0	0
ZNF687	zinc finger protein 687 [Homo sapiens]	0	0	0	0	0	0	0	37605.96062	37589.15573
ZNF740	zinc finger protein 740 [Homo sapiens]	0	177457.463	265427.9806	249227.6505	242402.6456	0	0	0	0
ZNF787	zinc finger protein 787 [Homo sapiens]	147505.9353	138646.1147	217503.3082	133122.6216	116408.2646	152792.2588	47726.56721	36233.06849	128178.7264
ZNF830	zinc finger protein 830 [Homo sapiens]	69508.65027	64058.98842	0	70487.19399	0	73081.19945	77951.52579	60364.20989	0
ZNFX1	NFX1-type zinc finger-containing protein 1 [Homo sapiens]	8217.551158	8076.92451	25336.70106	16356.77967	23537.33311	10620.19631	15317.87611	0	25011.58601
ZNHIT2	zinc finger HIT domain-containing protein 2 [Homo sapiens]	320510.3769	570902.8475	185127.5923	447619.5485	181073.9481	501957.0215	617104.8968	486464.4395	700786.8923
ZNHIT3	zinc finger HIT domain-containing protein 3 [Homo sapiens]	0	0	0	0	0	0	3720231.4	0	4105427.593
ZNHIT6	box C/D snoRNA protein 1 isoform 1 [Homo sapiens]	0	32888.95291	103423.5776	78266.32825	73755.31301	65643.70122	75836.70988	0	67600.20935
ZNRD1	DNA-directed RNA polymerase I subunit RPA12 [Homo sapiens]	0	0	0	0	0	0	0	618033.5116	587211.3192
ZRANB2	zinc finger Ran-binding domain-containing protein 2 isoform 1 [Homo sapiens	710454.9201	552962.1778	686509.3572	904181.018	762654.2747	939245.8268	735790.2407	686593.718	918550.4289
ZW10	centromere/kinetochore protein zw10 homolog [Homo sapiens]	301168.274	287301.9001	442479.5613	233260.5842	390301.3149	258956.763	260454.1736	221170.4185	254581.7653
ZWILCH	protein zwilch homolog [Homo sapiens]	117498.1534	188766.2142	132059.5891	77599.8669	186226.5261	69358.835	0	69442.5719	212549.514
ZWINT	ZW10 interactor isoform a [Homo sapiens]	0	0	0	0	0	0	0	145079.564	185947.0269
ZYG11B	protein zyg-11 homolog B [Homo sapiens]	0	0	0	0	0	0	72867.23352	0	52316.92752
ZYX	zyxin [Homo sapiens]	1899761.252	2037092.557	2841075.259	2407089.562	2672733.165	2309033.777	2264532.814	2210705.636	2093968.601
ZZEF1	zinc finger ZZ-type and EF-hand domain-containing protein 1 [Homo sapiens]	31187.7768	35405.55848	35726.03042	76690.25146	93031.64481	27013.82267	90799.12273	19848.45359	34480.57838
