# Supplementary material for: The neddylation of the RNA-dependent RNA polymerase 3D of Coxsackievirus B3 promotes viral replication
Source: J Virol. 2025 Oct 31;99(11):e01535-25. doi: 10.1128/jvi.01535-25 (PMC12646006; doi:10.1128/jvi.01535-25)
Supplement: Table S2 — Data of mass spectrometry for the proteins precipitated with 3Dpol. [file jvi.01535-25-s0003.pdf]

Table S2. Data of mass spectrometry for the proteins precipitated with $3D^{pol}$. HEK293T cells were transfected with the construct expressing Flag-3D for 36 h, followed by CVB3 infection (MOI = 5) or mock-infection for 18 h. Total cellular proteins were extracted by NP40 lysis buffer supplemented with 1% protease inhibitor and subjected to denatured Co-IP with anti-Flag antibody. The proteins precipitated with $3D^{pol}$ were analyzed by mass spectrometry (MS).

Accession	Gene Name	Description	Mass	Score	Matches	Sequences	emPAI	Coverage	
sp|P62987|RL40_HUMAN	UBA52	Ubiquitin-ribosomal protein eL40 fusion protein OS=Homo sapiens OX=960	15004	1221	66(51)	10(8)	13.27	60%	P62987_uniprot
sp|P05787|K2C8_HUMAN	KRT8	Keratin, type II cytoskeletal 8 OS=Homo sapiens OX=9606 GN=KRT8 PE=1	53671	232	22(13)	10(5)	0.52	14%	P05787_uniprot
sp|A0A8I5KQE6|RPSA2_HUMAN	RPSA2	Small ribosomal subunit protein uS2B OS=Homo sapiens OX=9606 GN=RP	33002	124	6(4)	4(3)	0.33	16%	A0A8I5KQE6_uniprot
sp|Q8N257|H2B3B_HUMAN	H2BC26	Histone H2B type 3-B OS=Homo sapiens OX=9606 GN=H2BC26 PE=1 SV	13900	119	7(4)	4(2)	0.55	30%	Q8N257_uniprot
sp|P05783|K1C18_HUMAN	KRT18	Keratin, type I cytoskeletal 18 OS=Homo sapiens OX=9606 GN=KRT18 PE	48029	94	5(4)	2(2)	0.14	3%	P05783_uniprot
sp|Q9UGM3|DMBT1_HUMAN	DMBT1	Deleted in malignant brain tumors 1 protein OS=Homo sapiens OX=9606 GN	268039	85	2(1)	2(1)	0.01	0%	Q9UGM3_uniprot
sp|Q01130|SRSF2_HUMAN	SRSF2	Serine/arginine-rich splicing factor 2 OS=Homo sapiens OX=9606 GN=SRSI	25461	74	4(2)	3(2)	0.28	14%	Q01130_uniprot
sp|P13667|PDIA4_HUMAN	PDIA4	Protein disulfide-isomerase A4 OS=Homo sapiens OX=9606 GN=PDIA4 PE	73229	67	4(2)	4(2)	0.09	6%	P13667_uniprot
sp|P01591|IGJ_HUMAN	JCHAIN	Immunoglobulin J chain OS=Homo sapiens OX=9606 GN=JCHAIN PE=1 S	18543	66	1(1)	1(1)	0.18	7%	P01591_uniprot
sp|P02788|TRFL_HUMAN	LTF	Lactotransferrin OS=Homo sapiens OX=9606 GN=LTF PE=1 SV=6	80014	59	3(3)	3(3)	0.13	4%	P02788_uniprot
sp|P37837|TALDO_HUMAN	TALDO1	Transaldolase OS=Homo sapiens OX=9606 GN=TALDO1 PE=1 SV=2	37688	48	3(2)	3(2)	0.18	8%	P37837_uniprot
sp|Q8WUM4|PDC6I_HUMAN	PDCD6IP	Programmed cell death 6-interacting protein OS=Homo sapiens OX=9606 GN	96590	47	4(2)	4(2)	0.07	4%	Q8WUM4_uniprot
sp|P47756|CAPZB_HUMAN	CAPZB	F-actin-capping protein subunit beta OS=Homo sapiens OX=9606 GN=CAP	30952	47	2(1)	1(1)	0.11	5%	P47756_uniprot
sp|P11908|PRPS2_HUMAN	PRPS2	Ribose-phosphate pyrophosphokinase 2 OS=Homo sapiens OX=9606 GN=PI	35146	46	3(1)	3(1)	0.20	10%	P11908_uniprot
sp|P35580|MYH10_HUMAN	MYH10	Myosin-10 OS=Homo sapiens OX=9606 GN=MYH10 PE=1 SV=3	229827	45	12(2)	7(2)	0.03	2%	P35580_uniprot
sp|P47929|LEG7_HUMAN	LGALS7	Galectin-7 OS=Homo sapiens OX=9606 GN=LGALS7 PE=1 SV=2	15123	44	1(1)	1(1)	0.23	8%	P47929_uniprot
sp|Q9NTK5|OLA1_HUMAN	OLA1	Obg-like ATPase 1 OS=Homo sapiens OX=9606 GN=OLA1 PE=1 SV=2	44943	43	3(1)	3(1)	0.07	8%	Q9NTK5_uniprot
sp|Q6P2Q9|PRP8_HUMAN	PRPF8	Pre-mRNA-processing-splicing factor 8 OS=Homo sapiens OX=9606 GN=PI	274738	42	6(2)	6(2)	0.02	1%	Q6P2Q9_uniprot
sp|Q15517|CDSN_HUMAN	CDSN	Corneodesmosin OS=Homo sapiens OX=9606 GN=CDSN PE=1 SV=3	52288	41	1(1)	1(1)	0.06	3%	Q15517_uniprot
sp|P54819|KAD2_HUMAN	AK2	Adenylate kinase 2, mitochondrial OS=Homo sapiens OX=9606 GN=AK2 PI	26689	40	4(1)	3(1)	0.13	13%	P54819_uniprot
sp|P78347|GTF2I_HUMAN	GTF2I	General transcription factor II-I OS=Homo sapiens OX=9606 GN=GTF2I PI	112859	39	10(1)	8(1)	0.03	5%	P78347_uniprot
sp|Q9ULH7|MRTFB_HUMAN	MRTFB	Myocardin-related transcription factor B OS=Homo sapiens OX=9606 GN=N	118282	39	5(3)	3(1)	0.03	1%	Q9ULH7_uniprot
sp|Q01081|U2AF1_HUMAN	U2AF1	Splicing factor U2AF 35 kDa subunit OS=Homo sapiens OX=9606 GN=U2A	28368	37	1(1)	1(1)	0.12	5%	Q01081_uniprot
sp|Q06210|GFPT1_HUMAN	GFPT1	Glutamine--fructose-6-phosphate aminotransferase [isomerizing] 1 OS=Homo	79555	36	2(1)	2(1)	0.04	2%	Q06210_uniprot
sp|Q15843|NEDD8_HUMAN	NEDD8	NEDD8 OS=Homo sapiens OX=9606 GN=NEDD8 PE=1 SV=1	9066	36	2(1)	1(1)	0.38	17%	Q15843_uniprot
sp|Q08J23|NSUN2_HUMAN	NSUN2	RNA cytosine C(5)-methyltransferase NSUN2 OS=Homo sapiens OX=9606	87214	36	4(1)	4(1)	0.04	3%	Q08J23_uniprot
sp|P61221|ABCE1_HUMAN	ABCE1	ATP-binding cassette sub-family E member 1 OS=Homo sapiens OX=9606	68240	36	4(1)	3(1)	0.05	4%	P61221_uniprot
sp|O60841|IF2P_HUMAN	EIF5B	Eukaryotic translation initiation factor 5B OS=Homo sapiens OX=9606 GN=	139198	36	5(1)	5(1)	0.02	2%	O60841_uniprot
sp|Q9GZT3|SLIRP_HUMAN	SLIRP	SRA stem-loop-interacting RNA-binding protein, mitochondrial OS=Homo sa	12398	36	1(1)	1(1)	0.28	9%	Q9GZT3_uniprot
sp|Q9BPW8|NIPS1_HUMAN	NIPSNAP1	Protein NipSnap homolog 1 OS=Homo sapiens OX=9606 GN=NIPSNAP1 P	33460	35	2(1)	2(1)	0.10	5%	Q9BPW8_uniprot
sp|Q5BKZ1|ZN326_HUMAN	ZNF326	DBIRD complex subunit ZNF326 OS=Homo sapiens OX=9606 GN=ZNF32	65955	34	7(1)	6(1)	0.05	8%	Q5BKZ1_uniprot
sp|Q9UI30|TR112_HUMAN	TRMT112	Multifunctional methyltransferase subunit TRM112-like protein OS=Homo sa	14304	34	2(1)	2(1)	0.24	15%	Q9UI30_uniprot
sp|P04844|RPN2_HUMAN	RPN2	Dolichyl-diphosphooligosaccharide--protein glycosyltransferase subunit 2 OS	69355	34	2(1)	2(1)	0.05	3%	P04844_uniprot
sp|Q96HN2|SAHH3_HUMAN	AHCYL2	Adenosylhomocysteinase 3 OS=Homo sapiens OX=9606 GN=AHCYL2 PE=	67705	34	4(1)	3(1)	0.05	4%	Q96HN2_uniprot
sp|P61289|PSME3_HUMAN	PSME3	Proteasome activator complex subunit 3 OS=Homo sapiens OX=9606 GN=P	29602	34	6(3)	4(3)	0.38	15%	P61289_uniprot
sp|Q5BJH7|YIF1B_HUMAN	YIF1B	Protein YIF1B OS=Homo sapiens OX=9606 GN=YIF1B PE=1 SV=1	34527	33	1(1)	1(1)	0.10	4%	Q5BJH7_uniprot
sp|P55769|NH2L1_HUMAN	SNU13	NHP2-like protein 1 OS=Homo sapiens OX=9606 GN=SNU13 PE=1 SV=3	14393	33	1(1)	1(1)	0.24	9%	P55769_uniprot
sp|Q15029|U5S1_HUMAN	EFTUD2	116 kDa U5 small nuclear ribonucleoprotein component OS=Homo sapiens O	110336	33	6(3)	4(2)	0.06	4%	Q15029_uniprot
sp|P14866|HNRPL_HUMAN	HNRNPL	Heterogeneous nuclear ribonucleoprotein L OS=Homo sapiens OX=9606 GN	64720	32	1(1)	1(1)	0.05	1%	P14866_uniprot
sp|P62888|RL30_HUMAN	RPL30	Large ribosomal subunit protein eL30 OS=Homo sapiens OX=9606 GN=RPI	12947	32	2(1)	2(1)	0.26	12%	P62888_uniprot
sp|P25786|PSA1_HUMAN	PSMA1	Proteasome subunit alpha type-1 OS=Homo sapiens OX=9606 GN=PSMA1	29822	32	6(1)	4(1)	0.11	12%	P25786_uniprot
sp|Q13257|MD2L1_HUMAN	MAD2L1	Mitotic spindle assembly checkpoint protein MAD2A OS=Homo sapiens OX	23666	32	1(1)	1(1)	0.14	3%	Q13257_uniprot
sp|Q15181|IPYR_HUMAN	PPA1	Inorganic pyrophosphatase OS=Homo sapiens OX=9606 GN=PPA1 PE=1 S	33095	32	2(1)	2(1)	0.10	12%	Q15181_uniprot
sp|P50750|CDK9_HUMAN	CDK9	Cyclin-dependent kinase 9 OS=Homo sapiens OX=9606 GN=CDK9 PE=1 S	43149	32	3(2)	2(2)	0.16	3%	P50750_uniprot
sp|Q8NB16|MLKL_HUMAN	MLKL	Mixed lineage kinase domain-like protein OS=Homo sapiens OX=9606 GN=N	55015	32	4(1)	3(1)	0.06	3%	Q8NB16_uniprot
sp|Q8WXF1|PSPC1_HUMAN	PSPC1	Paraspeckle component 1 OS=Homo sapiens OX=9606 GN=PSPC1 PE=1 S	58820	31	2(2)	2(2)	0.11	3%	Q8WXF1_uniprot
sp|P23284|PPIB_HUMAN	PPIB	Peptidyl-prolyl cis-trans isomerase B OS=Homo sapiens OX=9606 GN=PPIE	23785	31	3(1)	2(1)	0.14	7%	P23284_uniprot
sp|P61160|ARP2_HUMAN	ACTR2	Actin-related protein 2 OS=Homo sapiens OX=9606 GN=ACTR2 PE=1 SV=	45017	30	1(1)	1(1)	0.07	1%	P61160_uniprot
sp|Q9NTU7|CBLN4_HUMAN	CBLN4	Cerebellin-4 OS=Homo sapiens OX=9606 GN=CBLN4 PE=1 SV=1	21908	30	2(1)	1(1)	0.15	3%	Q9NTU7_uniprot
sp|P31689|DNJA1_HUMAN	DNAJA1	DnaJ homolog subfamily A member 1 OS=Homo sapiens OX=9606 GN=DN	45581	30	3(1)	2(1)	0.07	7%	P31689_uniprot
sp|P59998|ARPC4_HUMAN	ARPC4	Actin-related protein 2/3 complex subunit 4 OS=Homo sapiens OX=9606 GN	19768	29	2(1)	2(1)	0.17	11%	P59998_uniprot
sp|Q07283|TRHY_HUMAN	TCHH	Trichohyalin OS=Homo sapiens OX=9606 GN=TCHH PE=1 SV=2	254233	29	4(1)	4(1)	0.01	1%	Q07283_uniprot
sp|P55265|DSRAD_HUMAN	ADAR	Double-stranded RNA-specific adenosine deaminase OS=Homo sapiens OX=	137178	29	4(2)	3(2)	0.05	2%	P55265_uniprot
sp|Q8TDI0|CHD5_HUMAN	CHD5	Chromodomain-helicase-DNA-binding protein 5 OS=Homo sapiens OX=960	224506	28	1(1)	1(1)	0.01	0%	Q8TDI0_uniprot
sp|P30085|KCY_HUMAN	CMPK1	UMP-CMP kinase OS=Homo sapiens OX=9606 GN=CMPK1 PE=1 SV=3	22436	28	1(1)	1(1)	0.15	4%	P30085_uniprot
sp|P63208|SKP1_HUMAN	SKP1	S-phase kinase-associated protein 1 OS=Homo sapiens OX=9606 GN=SKP1	18817	28	1(1)	1(1)	0.18	7%	P63208_uniprot
sp|Q8N4U5|T11L2_HUMAN	TCP11L2	T-complex protein 11-like protein 2 OS=Homo sapiens OX=9606 GN=TCP1	58511	28	1(1)	1(1)	0.06	1%	Q8N4U5_uniprot
sp|A8MWD9|RUXGL_HUMAN	SNRPGP15	Putative small nuclear ribonucleoprotein G-like protein 15 OS=Homo sapiens	8595	27	1(1)	1(1)	0.41	9%	A8MWD9_uniprot
sp|Q15424|SAFB1_HUMAN	SAFB	Scaffold attachment factor B1 OS=Homo sapiens OX=9606 GN=SAFB PE=	103036	27	4(1)	4(1)	0.03	2%	Q15424_uniprot
sp|Q9HAV7|GRPE1_HUMAN	GRPEL1	GrpE protein homolog 1, mitochondrial OS=Homo sapiens OX=9606 GN=GI	24492	27	2(1)	2(1)	0.14	9%	Q9HAV7_uniprot
sp|Q9C037|TRIM4_HUMAN	TRIM4	E3 ubiquitin-protein ligase TRIM4 OS=Homo sapiens OX=9606 GN=TRIM	58280	26	3(1)	2(1)	0.06	3%	Q9C037_uniprot
sp|P15153|RAC2_HUMAN	RAC2	Ras-related C3 botulinum toxin substrate 2 OS=Homo sapiens OX=9606 GN	21814	26	1(1)	1(1)	0.15	4%	P15153_uniprot
sp|P59665|DEF1_HUMAN	DEFA1	Neutrophil defensin 1 OS=Homo sapiens OX=9606 GN=DEFA1 PE=1 SV=1	10536	26	1(1)	1(1)	0.33	9%	P59665_uniprot
sp|Q01844|EWS_HUMAN	EWSR1	RNA-binding protein EWS OS=Homo sapiens OX=9606 GN=EWSR1 PE=1	68721	25	2(1)	2(1)	0.05	3%	Q01844_uniprot
sp|O75339|CILP1_HUMAN	CILP	Cartilage intermediate layer protein 1 OS=Homo sapiens OX=9606 GN=CILI	134761	25	5(1)	2(1)	0.02	2%	O75339_uniprot
sp|Q7Z417|NUFP2_HUMAN	NUFIP2	FMR1-interacting protein NUFIP2 OS=Homo sapiens OX=9606 GN=NUFIF	76132	25	1(1)	1(1)	0.04	1%	Q7Z417_uniprot
sp|Q16531|DDB1_HUMAN	DDB1	DNA damage-binding protein 1 OS=Homo sapiens OX=9606 GN=DDB1 PE	128142	25	3(0)	3(0)	0.03	3%	Q16531_uniprot
sp|P48735|IDHP_HUMAN	IDH2	Isocitrate dehydrogenase [NADP], mitochondrial OS=Homo sapiens OX=960	51333	25	1(1)	1(1)	0.06	1%	P48735_uniprot
sp|P62847|RS24_HUMAN	RPS24	Small ribosomal subunit protein eS24 OS=Homo sapiens OX=9606 GN=RPS	15413	24	6(1)	3(1)	0.22	24%	P62847_uniprot
sp|Q9BY44|EIF2A_HUMAN	EIF2A	Eukaryotic translation initiation factor 2A OS=Homo sapiens OX=9606 GN=	65519	24	2(1)	2(1)	0.05	3%	Q9BY44_uniprot
sp|Q9NZM6|PK2L2_HUMAN	PKD2L2	Polycystin-2-like protein 2 OS=Homo sapiens OX=9606 GN=PKD2L2 PE=2	74256	24	1(1)	1(1)	0.04	0%	Q9NZM6_uniprot
sp|Q8IVF4|DYH10_HUMAN	DNAH10	Dynein axonemal heavy chain 10 OS=Homo sapiens OX=9606 GN=DNAH1	517705	24	11(1)	9(1)	0.01	1%	Q8IVF4_uniprot
sp|Q9NVI7|ATD3A_HUMAN	ATAD3A	ATPase family AAA domain-containing protein 3A OS=Homo sapiens OX=9	71610	24	2(1)	2(1)	0.05	2%	Q9NVI7_uniprot
sp|Q9H0D2|ZN541_HUMAN	ZNF541	Zinc finger protein 541 OS=Homo sapiens OX=9606 GN=ZNF541 PE=1 SV	147776	23	2(1)	2(1)	0.02	0%	Q9H0D2_uniprot
sp|O14929|HAT1_HUMAN	HAT1	Histone acetyltransferase type B catalytic subunit OS=Homo sapiens OX=96	49908	23	3(1)	3(1)	0.07	3%	O14929_uniprot
sp|O95197|RTN3_HUMAN	RTN3	Reticulon-3 OS=Homo sapiens OX=9606 GN=RTN3 PE=1 SV=2	113169	23	2(0)	2(0)	0.03	2%	O95197_uniprot
sp|Q7L014|DDX46_HUMAN	DDX46	Probable ATP-dependent RNA helicase DDX46 OS=Homo sapiens OX=960	117803	23	3(1)	3(1)	0.03	3%	Q7L014_uniprot
sp|Q96I15|SCLY_HUMAN	SCLY	Selenocysteine lyase OS=Homo sapiens OX=9606 GN=SCLY PE=1 SV=4	48404	22	2(1)	1(1)	0.07	1%	Q96I15_uniprot
sp|Q12792|TWF1_HUMAN	TWF1	Twinfilin-1 OS=Homo sapiens OX=9606 GN=TWF1 PE=1 SV=3	40429	22	1(0)	1(0)	0.08	3%	Q12792_uniprot
sp|P29374|ARI4A_HUMAN	ARID4A	AT-rich interactive domain-containing protein 4A OS=Homo sapiens OX=96	143634	22	3(1)	3(1)	0.02	1%	P29374_uniprot
sp|P55084|ECHB_HUMAN	HADHB	Trifunctional enzyme subunit beta, mitochondrial OS=Homo sapiens OX=96	51547	22	2(1)	2(1)	0.06	3%	P55084_uniprot
sp|Q9BYE4|SPR2G_HUMAN	SPRR2G	Small proline-rich protein 2G OS=Homo sapiens OX=9606 GN=SPRR2G PI	8779	21	1(1)	1(1)	0.40	12%	Q9BYE4_uniprot
sp|Q8N5K1|CISD2_HUMAN	CISD2	CDGSH iron-sulfur domain-containing protein 2 OS=Homo sapiens OX=960	15497	21	2(0)	2(0)	0.22	14%	Q8N5K1_uniprot
sp|O15354|GPR37_HUMAN	GPR37	Prosaposin receptor GPR37 OS=Homo sapiens OX=9606 GN=GPR37 PE=1	68724	21	1(1)	1(1)	0.05	1%	O15354_uniprot
sp|P35237|SPB6_HUMAN	SERPINB6	Serpin B6 OS=Homo sapiens OX=9606 GN=SERPINB6 PE=1 SV=3	42936	21	2(0)	2(0)	0.08	6%	P35237_uniprot
sp|A6NIK2|LR10B_HUMAN	LRRC10B	Leucine-rich repeat-containing protein 10B OS=Homo sapiens OX=9606 GN	32864	20	4(1)	2(1)	0.10	5%	A6NIK2_uniprot
sp|A6NCE7|MP3B2_HUMAN	MAP1LC3B2	Microtubule-associated proteins 1A/1B light chain 3 beta 2 OS=Homo sapien	14676	20	1(1)	1(1)	0.23	5%	A6NCE7_uniprot
sp|P62750|RL23A_HUMAN	RPL23A	Large ribosomal subunit protein uL23 OS=Homo sapiens OX=9606 GN=RPI	17684	19	3(0)	3(0)	0.19	19%	P62750_uniprot
sp|Q9BPX5|ARP5L_HUMAN	ARPC5L	Actin-related protein 2/3 complex subunit 5-like protein OS=Homo sapiens O	16931	19	1(1)	1(1)	0.20	7%	Q9BPX5_uniprot
sp|Q9Y2F5|ICE1_HUMAN	ICE1	Little elongation complex subunit 1 OS=Homo sapiens OX=9606 GN=ICE1	250759	19	5(1)	3(1)	0.01	0%	Q9Y2F5_uniprot
sp|Q16576|RBBP7_HUMAN	RBBP7	Histone-binding protein RBBP7 OS=Homo sapiens OX=9606 GN=RBBP7 P	48132	17	2(1)	2(1)	0.07	4%	Q16576_uniprot
sp|Q6ZU15|SEP14_HUMAN	SEPTIN14	Septin-14 OS=Homo sapiens OX=9606 GN=SEPTIN14 PE=1 SV=2	50449	16	3(1)	2(1)	0.07	3%	Q6ZU15_uniprot
sp|P26640|SYVC_HUMAN	VARS1	Valine--tRNA ligase OS=Homo sapiens OX=9606 GN=VARS1 PE=1 SV=4	141642	16	2(1)	2(1)	0.02	2%	P26640_uniprot
